# Molecular pathogenesis of Alzheimer's disease onset in a mouse model: effects of cannabidiol treatment

**DOI:** 10.3389/fnins.2025.1667585

**Published:** 2025-09-05

**Authors:** Mary A. Bishara, Phoebe P. Chum, Fritz E. L. Miot, Ankita Hooda, Richard E. Hartman, Erik J. Behringer

**Affiliations:** Department of Basic Sciences, Loma Linda University, Loma Linda, CA, United States

**Keywords:** cannabinoids, neuroinflammation, oxidative signaling, lipid metabolism, carbohydrate metabolism, cognitive function, *3xTg-AD* model

## Abstract

**Introduction:**

Alzheimer's disease (AD) is a common neurodegenerative condition involving a complex blend of disturbances in synaptic development and maintenance, neurovascular cross-talk, ionic and nutrient transport, and mitochondrial metabolism. The precise molecular profile of AD onset with insight for major pathological contributors remains unclear with corresponding impedances in therapeutic development. The current study sought two objectives, as (i) to resolve the molecular pathogenesis from cognitive impairment to the onset of AD-like neuropathology and (ii) whether the novel agent cannabidiol (CBD), noted for its neuroprotective effects, influences the molecular transition associated with AD onset.

**Methods:**

Dietary CBD was administered daily (80–100 mg/kg/day) in male *3xTg-AD* mice and wild-type B6129SF2/J animals from 4.5 to 6.5 mo of age with inclusion of vehicle controls. RNA sequencing encompassed longitudinal and cross-sectional blood and brain samples, respectively. Metabolomics and behavioral analyses examined brain regions (cortex, hippocampus) and associated integrated neurocircuitry.

**Results and discussion:**

There were >1,000 differentially expressed markers of AD onset, whereby >75% were either eliminated or reversed in the direction of expression in response to CBD. Signaling pathways encompassed synaptic development and plasticity (e.g., Foxp2), neurovascular interactions (Smad9, Angptl6), receptors and ion channels (Gria4, Chrna2, Rgs7/Rgs7bp), mitochondrial genes (Ndufa7, Cox7a2), immunity (Ncr1), oxidation-reduction (Esr1), lipid synthesis (Fasn, ApoE), and carbohydrate metabolism (Mafa, Mlxipl). As potentially addressable with CBD treatment, AD onset represents molecular integration of neurovascular interactions, channelopathies, metabolic disturbances, and aberrations in developmental genes with involvement of major pathological contributors such as inflammation, oxidative signaling, dyslipidemia, and insulin resistance.

## Introduction

Alzheimer's disease (AD) is a multifactorial neurodegenerative disorder that currently impacts ≈6.7 million Americans with a drug development pipeline in place that primarily targets abnormalities in neurotransmission, inflammation, and amyloid burden (Cummings et al., 2025). To help expand capabilities for diagnosis and therapy of AD, fundamental applications of comprehensive molecular analyses such as transcriptomics and proteomics have been recognized over the past decade (Rahimzadeh et al., 2024; Sutherland et al., 2011). As a result, we now have a clearer view of the molecular “signatures” of major pathological contributors to AD as inflammation (Amelimojarad et al., 2024), oxidative stress (Bhandari et al., 2024), dyslipidemia (de Oliveira et al., 2024), and insulin resistance (Kale et al., 2024). However, outside of simplified annotation tools, there remains a challenge to resolve large, untargeted data sets while equipped with a physiological perspective to optimally locate and integrate significant biological markers into healthy cerebral perfusion and cognition. Furthermore, there is a need to enhance mechanistic insight into the early development of AD and, in particular, the critical and costly transition from mild cognitive impairment (MCI) to AD (Frech et al., 2024).

In tandem with experimentally comprehensive tools that best capture molecular pathogenesis, there remains a critical need for refining effective AD therapeutic strategies, particularly regarding the application of single, or combinations of, pharmacological agents (Cummings et al., 2025). From 2019 to the end of 2023, the use of cannabidiol (CBD) in particular has increased from 14% to 21% among adults in the United States (Wilson-Poe et al., 2023) to alleviate symptoms of a wide range of neurological conditions (e.g., anxiety, chronic pain, migraines, epilepsy, and schizophrenia; Mallick et al., 2024). The encompassing health effects of CBD are not surprising as it is known to target the primary cannabinoid receptors (CB1R & CB2R) in addition to a plethora of other G-protein coupled receptors (e.g., GPCR3/6/12/55, μ/δ opioid, adenosine A1, 5-HT1A, and dopamine D2), ligand-gated receptors (e.g., AMPA and GABA), and ion channels (e.g., TRPV1-4, TRPA1, TRPM8, Na_v_1.1-1.7, Ca_v_1.1-1.4/3.1-3.3, and K_v_7.2-7.3; Wright, 2024) with several more transmembrane targets yet to be tested. It is also worth noting that three clinical trials of CBD treatment for MCI to mild/moderate AD pathology have begun as of January 2021 (NCT04075435, Phase 1), February 2021 (NCT04436081, Phase 2), and January 2024 (NCT05822362, Phase 2; Cummings et al., 2025). In addition, CBD potentially presents a novel experimental (e.g., cyclodextrins) and therapeutic (e.g., statins) alternative to managing membrane cholesterol homeostasis (Guard et al., 2022) as relevant to the AD risk factor apolipoprotein E ε4 allele (APOE4; Sun et al., 2023b) while central to cardiovascular and cognitive health (Rashid et al., 2023). Altogether, CBD may be harnessed for treating a broad spectrum of neurodegenerative diseases; however, a clear mechanistic understanding of how CBD modulates molecular pathways specifically associated with AD-like pathogenesis remains incomplete.

Using the *3xTg-AD* mouse model, the current study sought two objectives as (i) to resolve the molecular pathogenesis from cognitive impairment to the onset of AD-like neuropathology and (ii) determine whether CBD could influence the molecular transition associated with MCI to that of AD. For longitudinal molecular measurements, whole blood samples were examined from male mice during the cognitive impairment (4.5 mo, wk 0) and AD-like neuropathology (6.5 mo, wk 8) phases of the animal's lifespan using bulk RNA sequencing (CBD-treated vs. vehicle). We used transcriptomic and metabolomic profiling to identify molecular changes at the earliest stages of AD, as these methods provide comprehensive insight into gene expression and metabolic disturbances preceding the onset of clinical symptoms. Cross-sectional comparisons entailed bulk RNA sequencing and metabolomics of whole brain samples. The same animals, along with sex- and age-matched wild-type B6129SF2/J (now hereby referred to as B6129) mice, were assessed using behavioral assays [Morris water maze (MWM), open field test (OFT), and nest building test (NBT)] at ages 4.5 and 6.5 mo. Our baseline expectation was that CBD would disrupt the expression of key biomarkers of AD pathogenesis involving neuroinflammation and amyloid-β metabolism. In brief, we found >900 differentially expressed genes (DEGs) in the blood associated with the onset of AD-like neuropathology in *3xTg-AD* mice, whereby ~240 DEGs have previously been identified as AD-associated markers in human subjects. Furthermore, dietary CBD treatment removed respective DEGs (or reversed their direction of expression) in at least 75% of these AD-selective genes. Using the *3xTg-AD* animal model as a surrogate for studying molecular mechanisms underlying AD pathogenesis, these data have implications for the early-stage pathogenesis of AD while reinforcing dietary CBD as a robust therapeutic option.

## Materials and methods

### General animal care and use

All animal care use and experimental protocols for this study were approved by the Institutional Animal Care and Use Committee of Loma Linda University and performed in accordance with the National Research Council's “Guide for the Care and Use of Laboratory Animals” (8th Edition, 2011). Experiments were performed using male B6129 mice (*n* = 10) [The Jackson Laboratory (Wilmington, MA, USA), strain#: 101045] and male *3xTg-AD* mice (*n* = 10) [(B6;129-Tg (APP-Swe, tauP301L) 1Lfa Psen1tm1Mpm/Mmjax); Mutant Mouse Resource and Research Center (MMRRC) stock #034830]. The *3xTg-AD* mouse model was selected due to its robust expression of hallmark AD pathology, including amyloid-β plaques, tau neurofibrillary tangles, and cognitive deficits, making it suitable for investigating effects of early intervention. At 4–5 mo of age, *3xTg-AD* mice generally exhibit cognitive impairment but minimal extracellular amyloid-β (Aβ) plaques, whereas the presence of neuropathology in the form of extracellular Aβ plaques is generally noted by 6–8 mo of age. All 20 mice were at 4.5 mo of age in the beginning of the study and 6.5 mo at the end (Oddo et al., 2003; Belfiore et al., 2019; Chum et al., 2022). All animals were housed on a 12:12-h light–dark cycle at 22–24 °C with fresh water and food available *ad libitum*.

### Housing and dietary training for *ad libitum* ingestion of CBD in raspberry-flavored gelatin relative to vehicle

To closely monitor the complete consumption of food, water, and a Jello-type raspberry-flavored gelatin [Item model #:4300020072; Sun Maid, USA (vehicle for dietary CBD dissolved in 95% ethanol)] of individual animals, mice were single-housed for 11 days prior to handling. Animals were single-housed to closely monitor CBD administration while ensuring intake on an individual level. Observation of any anxiety (e.g., rapid chewing of food and excessive grooming) during this period was addressed using additional enrichment (toys) added to the cage. Five days prior to the start of the gelatin training period, each mouse was handled for 5 min per day. Procedures for habituating and reducing stress in mice were performed in accordance with a “three-dimensional handling technique” (Marcotte et al., 2021), whereby the identity of the handler/experimenter (one to two people at most) to individual animals was kept as consistent as possible.

With water remaining available throughout, mice were fasted for 12 and 16 h prior to the first and second days of gelatin presentation, respectively. After the mice completed the gelatin training procedure for the first 2 days, they were presented with their regular food and water *ad libitum* until the start of the next fasting period. The mice were fasted for only 2 out of the 5 days of gelatin training to encourage ingestion of the gelatin upon presentation. For each gelatin feeding period, the gelatin was provided on a weighing boat as a tray in a clean empty cage without any bedding or enrichment for a maximum of 1 h. If a mouse consumed the prepared gelatin cube within the hour, they were placed back into their home cage immediately to encourage eating as quickly as possible. Note that two wild-type B6129 mice designated in the vehicle group did not respond with eating the raspberry-flavored gelatin or an alternative as an unflavored gelatin (Knox, Item model number: 10043000048679; Kraft-Heinz, USA) and thus were excluded from the core analyses of the study as presented here in the manuscript.

CBD was obtained from Cayman Chemical Company (Ann Arbor, MI, USA) as 2-[1R-3-methyl-6R-(1-methylethenyl)-2-cyclohexen-1-yl]-5-pentyl-1,3-benzenediol (Catalog #90080). With the limitation of low bioavailability (≈9%) relative to parenteral intravenous administration (Xu et al., 2019), the oral route was chosen based on its non-invasiveness and representation of use in the human population (Arnold et al., 2023; Jha et al., 2024). The time frame (8 wks) and frequency (once per day) of administration was chosen in accord with a consistent and chronic treatment period encompassing the transition from pre- to post-plaques in the brains of *3xTg-AD* animals. With consideration of prior studies of mouse models of neurodegenerative disease (Dearborn et al., 2022; Kreilaus et al., 2022; Coles et al., 2020; Hao and Feng, 2021; Watt et al., 2020; Cheng et al., 2014; Long et al., 2010) combined with untested effects on *3xTg-AD* animals in particular, we first provided CBD samples as 80 mg/kg/day for 4 wks and monitored for any signs of overt toxicity. With none observed, we proceeded with 100 mg/kg/day as a “high” therapeutic dose (Xu et al., 2019) for the final 4 wks of the treatment period.

### Blood sample collection

Blood was collected from all animals via tail clipping prior to the CBD administration; then, trunk blood was collected at the end of the study. Tail clipping was performed, while the mouse was under anesthesia. To ensure the comfort of the mice during this process, they were placed in an airtight container and anesthetically induced with isoflurane at 3% for 3 min. Afterward, they were fitted into a nose cone and the isoflurane was lowered to 1.5% for the remainder of the process, which averaged an additional 20 min. Trunk blood was collected while the mouse was under anesthesia prior to brain and organ collection, and the procedure was terminal. A 150–200 μl blood sample was obtained from the tail, and 500–750 μl of blood from the trunk was collected from each mouse. A 1:1 ratio of RNA/DNA Shield 2X Concentrate (R1200-25; Zymo Research, Irvine, CA, USA) was added to each blood solution to preserve the samples, which were then sent to Zymo Research for RNA sequencing analysis.

### Brain and organ collection

On the final day of the project, animals were euthanized after the completion of the OFT experiment. The brain was extracted from each mouse and stored in the −80 °C freezer for further analysis. Half of the brain was snap-frozen in liquid nitrogen and ground to powder using mortar and pestle; then, the powder was divided in half for RNA sequencing and metabolomics analysis, respectively.

### RNA sequencing

A powdered brain sample per animal (80–127 mg) was stored in 1X RNA/DNA Shield (R1100; Zymo Research) according to the manufacture instructions and stored in −80 °C freezer prior to shipment. RNA extraction, sequencing, and bioinformatics analysis were done by Zymo Research on Illumina NovaSeq X Plus platform with 30 million read pairs per sample for both blood and brain samples. For differentially expressed genes (DEGs) calculations, RNAseq pipeline (v2.1.0) developed by Zymo Research with the DESeq2 package (v1.28.0) was employed for calculation of DEGs. We defined significant DEGs as those fulfilling *p*-value < 0.05 and an absolute value of log2 fold change > 1.

### Metabolomics

A powdered brain sample per animal (98–150 mg) was stored dry in −80 °C freezer prior to shipment. The Untargeted Metabolomic Service was performed by Creative Proteomics (Shirley, NY, USA) on the Thermo Q Exactive UPLC-MS/MS platform. A list of comprehensive metabolites in both positive and negative mode was obtained as part of the analysis report provided by Creative Proteomics.

### Morris water maze

Learning and memory (general associative and spatial) were tested using the MWM, a plastic circular pool (85 cm in diameter) filled with water (25 ± 2 °C) made opaque using non-toxic tempera paint (Handy Art, Inc. Milton, WI, USA). The mice had to find and climb onto an escape platform (11 cm in diameter), the surface of which was either 1.5 cm above the water's surface for the “cued” task or 1.5 cm below the water's surface for the “spatial” task. The test was performed prior to the CBD exposure and after 8 weeks of daily CBD exposure.

On the first day of MWM testing, each mouse was trained to locate the platform during the cued trials, in which the platform's location changed every trial, but remained visible to the mice. For the subsequent 3 days of the spatial navigation testing, mice were trained to locate a submerged (hidden) platform that remained in the same location for all the trials of that day and before changing to a different location on the following day. Five trials were administered per day. For each trial, the mouse was placed into the water pool at different start locations (E, S, W, and N) and allowed to locate the hidden platform. If the mouse was unable to locate the platform within 60 s, it was gently guided to the platform by the experimenter. Once on the platform, it was allowed to remain for 15s. A “probe” trial, in which the platform was removed and the mouse was allowed to swim freely for 60 s, was performed at the end of the day on the spatial performance days (24 h after the last training trial). The position of each mouse was tracked by a camera above the center of the pool and was connected to an automatic photographic recording and analysis system (Noldus, EthoVision XT 11.5, Leesburg, VA, USA). The escape latency (i.e., the time required to locate the hidden platform), latency of the first entrance to the target zone, and the time spent in the target zone (% of the total time in all the four zones) during the 4-day acquisition training, the swimming paths, and the number of crossings into the target quadrant during the probe trial were all recorded.

### Open field test

The OFT was used to measure the exploratory behavior of the *3xTg-AD* and B6129 mice. The test was conducted the day after the MWM was completed. An hour prior to the start of the test, the mice were relocated to the behavioral testing room to acclimate to the room's lighting and temperature conditions. The test was conducted in a box that is 76.2 cm × 76.2 cm. The floor of the box was covered with white butcher paper that is the exact dimensions of the box. Mice were released into the middle of the OFT maze and allowed to explore freely for 30 min with no interruptions. At the end of the 30 min, the mice were removed from the box and new white butcher paper was placed. This procedure was repeated for each mouse, and the mouse tracking data were collected and analyzed with the EthoVision XT 11.5 Software system.

### Nest building test

NBT was performed 3 days prior to the gelatin training period during the animal handling week on day 3 of the handling. Each mouse was given one-third of a paper towel (Georgia Pacific 20204 Acclaim Multifold Paper Towels, White, Poly-Bag Protected). Each paper towel was cut into 1 cm × 8 cm strips and was evenly distributed across the width of each clean cage before putting the mouse into the cage. The nesting materials were presented to the mice after the third handling session, and the mice were left undisturbed for 24 h until the next handling session. A picture of the nest was taken after the nesting materials were presented at 12, 36, and 60 h. All nesting materials were removed after 60 h, and the mice were given their regular enrichment and cotton bedding. At the end of the 60 h, the pictures from the three nesting days were sent to three experimenters who were blind to the study groups. The scoring criteria were designated from a score of 1–5 as follows: (1) nest materials remained scattered throughout the cage, untouched, or entirely disorganized; (2) material was collected near the edges and corners of the cage and but remain scattered; (3) most of the material primarily in one quadrant of the cage; (4) material not shredded but packed into one corner; (5) material shredded and packed into one corner as an identifiable nest (Neely et al., 2019). The three experimenters rated the state of each nest from each picture, and then, the scores were averaged over each day for each mouse. The process was repeated after CBD treatment. Photos of the cages showing the nest state were once again taken after 12, 36, and 60 h.

### Statistical analysis

For behavioral assays, all statistical analyses were performed using GraphPad Prism (Version 10.1.2; GraphPad Software, La Jolla, CA). Analysis included a two-way analysis of variance (Tukey's *post-hoc*). Differences between groups were accepted as statistically significant with p < 0.05. All summary data are presented as the mean ± SEM.

## Results

The aims of the current study were to resolve molecular pathogenesis throughout the range of cognitive impairment associated with development of AD-like neuropathology and to determine whether oral administration of CBD could influence this molecular transition. In addition, we endeavored to identify novel biomarkers of AD pathogenesis as well. Using the *3xTg-AD* animal model (Chum et al., 2022; Stevens and Brown, 2015; Jullienne et al., 2022; Stover et al., 2015), untargeted transcriptomic and metabolomic analyses were employed in combination with behavioral assays. For within group comparisons (e.g., longitudinal blood analyses, wk 8 vs. wk 0), study groups are presented first in the following order: *3xTg-AD* vehicle, wild-type vehicle, *3xTg-AD* CBD-treated, and wild-type CBD-treated. For cross-group comparisons (e.g., cross-sectional brain analyses at wk 8), the order of presentation is *3xTg-AD* vehicle vs. wild-type vehicle, *3xTg-AD* CBD-treated vs. *3xTg-AD* vehicle, wild-type CBD-treated vs. wild-type vehicle, and *3xTg-AD* CBD-treated vs. wild-type CBD-treated. Due to the extensive nature of the datasets, not all results are thoroughly discussed here; therefore, readers are referred to the Supplementary Tables 1–34 for comprehensive lists of DEGs and pathway interactions across study groups.

### Transcriptomic analyses: longitudinal blood samples with AD onset

**Figure 1:**
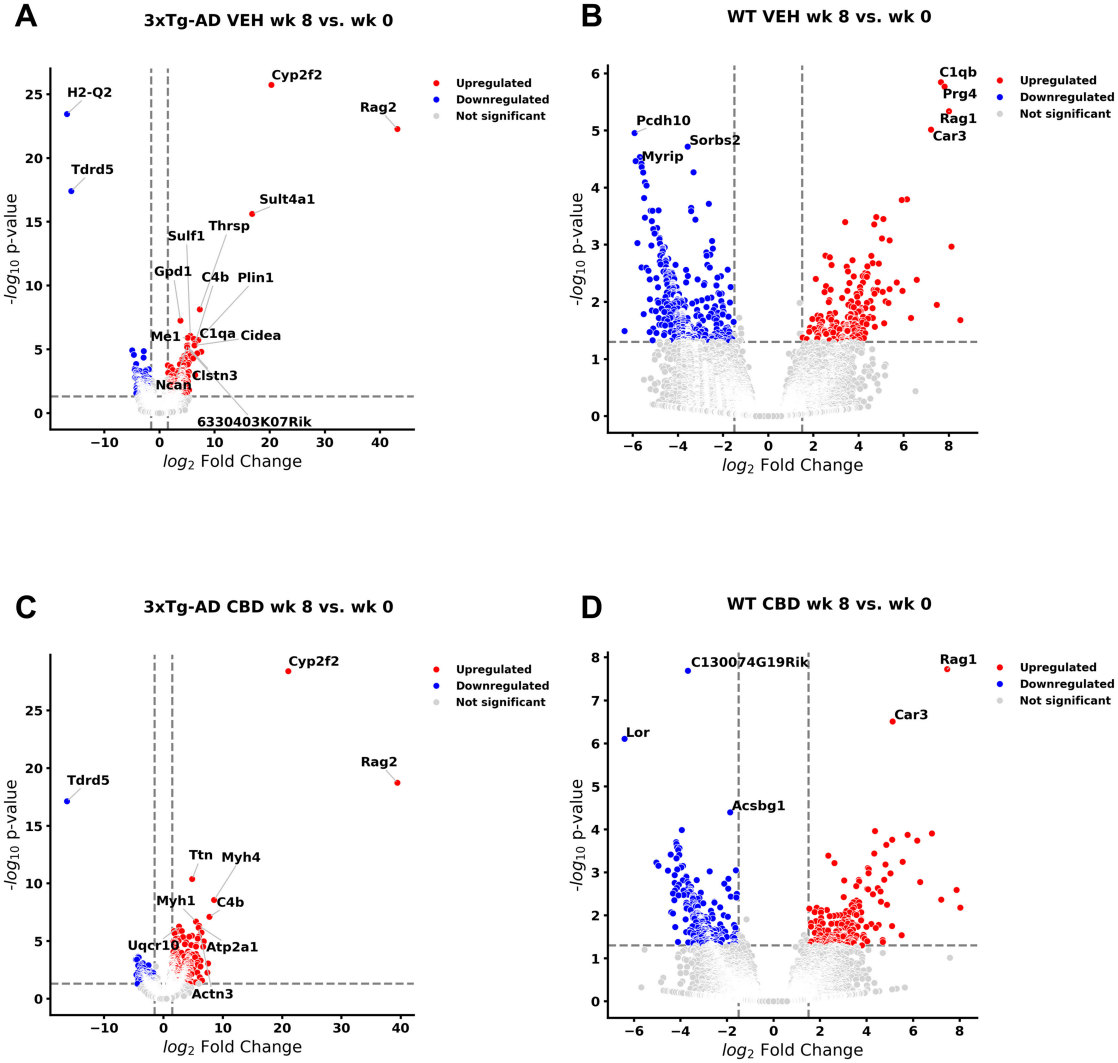
Volcano plots of longitudinal changes of gene profiles collected from whole blood: effect of Alzheimer's disease onset and cannabidiol. Genes that were upregulated (red), downregulated (blue), and were not significantly altered (light gray) from 4.5 mo (0 wks) to 6.5 mo (8 wks) **(A)** in *3xTg-AD* animals; 447 genes were downregulated, and 471 genes were upregulated (see Supplementary Table 1). **(B)** In wild-type B6129 mice, 593 genes were downregulated and 198 genes were upregulated (see Supplementary Table 2). **(C)** In cannabidiol (CBD)-treated *3xTg-AD* mice, 180 genes were downregulated and 663 genes were upregulated (see Supplementary Table 6). **(D)** In CBD-treated B6129 mice, 338 genes were downregulated and 198 genes were upregulated (see Supplementary Table 9). Data were obtained from *n* = 3–5 male mice per group. For complete reports on overlap of genes across respective groups, see Supplementary Tables 3, 7, 10, 11. For complete reports on reversal of significant gene profiles across groups (e.g., CBD-treated *3xTg-AD* vs. vehicle *3xTg-AD*), see Supplementary Tables 4, 5, 8, 12, 13, 14. This figure was generated through the use of QIAGEN IPA (QIAGEN Inc., https://digitalinsights.qiagen.com/IPA) (Krämer et al., 2014).

Comprehensive blood- and/or brain-based analyses of RNA biomarkers can track development of MCI and AD-associated neuropathology in mouse models (Li et al., 2023; Barisano et al., 2022) and human subjects (Li et al., 2023; Shigemizu et al., 2020; Wang et al., 2024b). With comparison of whole blood of *3xTg-AD* animals at AD onset (6.5 mo, 8 wks vehicle treatment) vs. cognitive impairment (4.5 mo, 0 wks vehicle treatment), there were 447 and 471 genes significantly downregulated and upregulated, respectively (Figure 1A, Supplementary Table 1). The most extreme expression alterations (|log2 fold change| > 15) include Histocompatibility 2, Q region locus 2 (H2-Q2) and Tudor domain containing 5 (Tdrd5) genes for downregulation and Sulfotransferase family 4A member 1 (Sult4a1), Cytochrome P450, family 2, subfamily f, polypeptide 2 (Cyp2f2), and Recombination Activating 2 (Rag2) genes for upregulation (Figure 1A, Supplementary Table 1). DEGs for non-coding RNAs include 94 long non-coding RNAs (lncRNAs; 66 downregulated, 28 upregulated), 8 microRNAs (miRNAs; 7 downregulated, 1 upregulated), 2 small nuclear RNAs (snRNAs; both downregulated), and 7 small nucleolar RNAs (snoRNAs; 6 downregulated, 1 upregulated). Note that 206 DEGs are not annotated (unknown or uncharacterized) for pathway analysis, whereby 89% were downregulated (=183) vs. 11% upregulated (=23). For DEGs previously identified for AD pathology, 40 and 200 genes were downregulated (9% of 447 genes) and upregulated (42% of 471 genes), respectively (Table 1). As not necessarily selective for AD pathogenesis *per se*, other select DEGs of interest have also been tracked throughout study groups as shown in Table 2.

**Table 1:** Differentially expressed genes (*P* < 0.05) in whole blood of *3xTg-AD* vehicle (weeks 8 vs. 0) animals that are recognized with Alzheimer's disease pathology.

**Category**	**Genes**
Downregulated (40 genes)	5S_rRNA, Acvr1, Apex1, Cacna2d4, Crmp1, Dock9, Efna1, Egfl7, Esr1, F12, Fam167a, Fam222a, Gprc5b, Il18bp, Iqck, Itgb8, Kcnip3, Map2k3os, Mir342, Mybpc3, Ncr1, Peg3, Prss36, Ramp3, Rapgef4, Rin1, Scg5, Sema4c, Sesn2, Slc22a5, Snx33, Sparcl1, Tagln, Tagln3, Tamalin, Tchh, Unc5c, Vangl2, Wfs1, Wnt10a
Upregulated (200 genes)	Acacb, Acss3, Acy3, Adamts13, Adora1, Adra1a, Aebp1, Agbl2, Ak9, Aldoc, Angptl2, Ankrd36, Antxr1, Aoc3, Apba2, Atp1a2, Bcam, Bche, Bgn, Bmp4, Bmp6, C1qa, C1qb, C1qc, C1s1, C4b, Cabcoco1, Cacna2d3, Calcb, Camp, Cav1, Ccdc81, Cckbr, Cd163, Cd209b, Cdk18, Cdkn2a, Cgnl1, Chadl, Chmp4c, Chrna2, Chst1, Chst7, Cldn10, Clstn3, Cnnm1, Col18a1, Col6a2, Coro2b, Cox7a2, Cox8b, Cplx2, Cpxm1, Csmd1, Cyp1b1, Dab1, Dab2, Dagla, Dcn, Ddit4l, Ddr2, Dkk3, Dock3, Eda2r, Ednrb, Efemp1, Elovl4, Etv4, Fabp3, Fabp7, Fam20a, Fasn, Fbln1, Fbxo15, Fcrls, Fgf14, Fgf2, Fgfr3, Fkbp14, Flrt2, Fn1, Foxc1, Foxp2, Fras1, Fstl1, Gas1, Gas6, Ghr, Ghrl, Gls2, Gpr6, Grb14, Gria4, Hapln2, Hbegf, Homer1, Ica1l, Igfbp5, Ighg1, Il33, Ildr2, Insm1, Kank1, Kcnk2, Kcnn3, Kndc1, Lag3, Lin7a, Lpar1, Lrfn5, Ltc4s, Map1lc3b, Mei1, Mertk, Mgat3, Mgp, Mir144, Mlxipl, Mme, Mrc1, Mroh8, Ms4a7, mt-Nd6, Mt2, Myh4, Myt1l, Nap1l2, Nbea, Ncan, Ndufa7, Ninj2, Nos1, Npsr1, Ntrk2, Ntsr1, Oprd1, P4ha3, Pcdh9, Pcdhgc5, Pck1, Pcsk2, Pcsk5, Pdcd1, Pdia5, Pdk4, Pfdn5, Pgr, Phf24, Pld6, Plekhh1, Pm20d1, Ppp1r3c, Prelp, Prkar1b, Prok2, Prox1, Ptgis, Pth1r, Pth2r, Ptpn5, Ptprd, Rarres2, Rbm24, Rbms3, Rbp4, Rgs7, Rgs7bp, Rorb, Rpl3l, Saa3, Scara3, Serping1, Sfrp1, Slc17a7, Slc4a4, Smad9, Snap91, Spint1, Srgap1, St8sia3, Stard13, Synpo2, Syt10, Tacr3, Thbs2, Thbs4, Tmem119, Tmem176b, Tmem63c, Tnc, Trpc3, Tspan6, Tspan7, Uqcr10, Uqcrh, Vcam1, Vgf, Wnt5a, Zbtb7c, Zic1

See Supplementary Table 1 for citations.

**Table 2:** A list of 53 differentially expressed genes (*P* < 0.05) of interest (neurological and cardiovascular conditions) and their relevant functions in the whole blood of *3xTg-AD* vehicle (weeks 8 vs. 0) animals that are tracked throughout all study groups in parallel with AD-selective DEGs, including wild-type animals with and without CBD treatment.

**Gene symbol**	**Gene name**	**Relevant functions**	**Citation(s)**
Sgtb	Small glutamine rich tetratricopeptide repeat co-chaperone beta	Associated with cognitive resilience	Yu et al., 2020
Phf24	PHD finger protein 24	Underlies GABA_B_ receptor-driven synaptic transmission, whereby its deficiency is associated with increased seizure sensitivity and cognitive impairment	Serikawa et al., 2019
lncRNA C920006O11Rik	–	Involved in Parkinson's disease	Jia et al., 2020
Tox3	TOX high mobility group box family member 3		Xie et al., 2022
Timm8b	Translocase of inner mitochondrial membrane 8 homolog B		Wang et al., 2024a
Necab2	N-terminal EF-hand calcium binding protein 2		Xie et al., 2022
Pak6	P21 (RAC1) activated kinase 6		Giusto et al., 2024
Gucy2c	Guanylate cyclase 2c		Cheslow et al., 2024
Tgm1	Transglutaminase 1	Risk genes for Huntington's disease	O'Day, 2022
Fam171b	Family with sequence similarity 171 member B		Tran et al., 2021
Gprasp2	G protein-coupled receptor associated sorting protein 2	Associated with neurodevelopmental disorders such as autism spectrum disorder	Edfawy et al., 2019; Piton et al., 2011
Susd4	Sushi domain containing 4		Tu et al., 2010; Zhu et al., 2020
Cdh11	Cadherin 11		Wu et al., 2022; Crepel et al., 2014
Cradd	CASP2 and RIPK1 domain containing adaptor with death domain	Associated with intellectual disability	Di Donato et al., 2016
Slc45a1	Solute carrier family 45 member 1	Associated with autosomal recessive intellectual disability	Anazi et al., 2017
Rai1	Retinoic acid induced 1	Indicative of Smith-Magenis syndrome	Turco et al., 2022
Asxl3	Additional Sex Combs-Like transcription regulator 3	Associated with Bainbridge-Ropers syndrome	Schirwani et al., 2023
Sufu	Suppressor of fused homolog	Variants of SUFU negative regulator of hedgehog signaling are associated with Joubert syndrome	Siegert et al., 2024
Kcng2	K^+^ voltage-gated channel modifier subfamily G member 2	Schizophrenia-risk genes	Guo et al., 2023
Kcnq5	K^+^ voltage-gated channel subfamily Q member 5		Baird et al., 2021
Nt5dc2	5'-nucleotidase domain containing 2		Chen et al., 2024
Btbd9	BTB domain containing 9	Involved in Restless Legs Syndrome and adult attention-deficit/hyperactivity disorder	Gan-Or et al., 2015; Alemany et al., 2015
Tmsb4x	Thymosin beta-4 X-linked	Involved in major depressive and bipolar disorder	Kim et al., 2021
Kcnt2	K^+^-Na^+^-activated channel subfamily T member 2	Involved in developmental epileptic encephalopathy	Cioclu et al., 2023
Scn7a	Na^+^ voltage-gated channel alpha subunit 7	Increased and persistent expression contributes to epilepsy in the rodent and human hippocampus	Gorter et al., 2010
Kif1a	Kinesin family member 1A	Associated with neurological disorder	Nair et al., 2023
Asic4	Acid sensing ion channel subunit family member 4	Modulates innate fear and anxiety	Lin et al., 2015
Chmp4c	Charged multivesicular body protein 4C	Associated with the pathogenesis of spinal and bulbar muscular atrophy	Malik et al., 2019
Kcnq4	K^+^ voltage-gated channel subfamily Q member 4	Contribute to non-syndromic hearing loss	Lee et al., 2021
Abcc9	ATP-binding cassette, sub-family C member 9	Associated with hippocampal sclerosis of aging pathology	Nelson et al., 2014
Postn	Periostin	An indicator of the decline of physical and cognitive capacity in the elderly (≥70 years of age)	Sánchez-Sánchez et al., 2023
Zcchc14	Zinc finger CCHC-type containing 14	Associated with small vessel stroke	Traylor et al., 2017
Jam3	Junctional adhesion molecule 3	Underly hemorrhagic destruction of the blood brain barrier	Akawi et al., 2013
Tmem100	Transmembrane protein 100	Selective for pulmonary vascular endothelium development and morphogenesis	Liu et al., 2022
Lockd	lncRNA downstream of Cdkn1b	Modulator of vascular structure and function	Sung et al., 2018
EphB4	Ephrin receptor B4	Regulate angiogenesis	Chen et al., 2022
Angptl6	Angiopoietin like 6	Carbone et al., 2018
Kcnk3	Potassium channel, subfamily K, member 3	Drives hereditary pulmonary arterial hypertension	West et al., 2021
Des	Desmin	Promotes toxic amyloid aggregates outside of the brain in cardiac and skeletal muscle	Sanbe et al., 2005; Kedia et al., 2019
Pdpn	Podoplanin	Indicates the presence of meningeal lymphatic vessels that may help clear amyloid from the brain parenchyma during AD	Goodman et al., 2018
Klhdc7a	Kelch domain containing 7A	Associated with regulation of circadian rhythm during diabetic retinopathy	Ling et al., 2023
Stk36	Serine/threonine kinase 36	Involved in primary ciliary dyskinesia	Edelbusch et al., 2017
Rsph1	Radial spoke head component 1		Knowles et al., 2014
Sidt1	SID1 transmembrane family member 1	Involved in transports of RNA and cholesterol	Méndez-Acevedo et al., 2017
Abca8	ATP-binding cassette, sub-family A member 8a	Regulates cholesterol efflux and high-density lipoprotein cholesterol levels	Trigueros-Motos et al., 2017
Acat3	Acetyl-coenzyme A acetyltransferase 3 (human ortholog is ACAT2)	Located in mitochondria; involved in hypercholesterolemia and coronary artery disease	Rudel et al., 2005
Alox8	Arachidonate 8-lipoxygenase	Metabolizes arachidonic acid to 8-hydroxyeicosatetraenoic acid (8-HETE), a pro-inflammatory metabolite	Furstenberger et al., 2002
Gpd1	Glycerol-3-phosphate dehydrogenase 1	Regulate lipid metabolism	Kawamura et al., 2022
Lgals12	Galectin 12		Tsao et al., 2023
Plin1	Perilipin 1		Griseti et al., 2024
Plin4	Perilipin 4		Ge et al., 2019
Thrsp	Thyroid hormone responsive		Li et al., 2024
Cidec	Cell death inducing DFFA like effector c	Promotes lipid droplet formation; upregulation associated with hypercholesterolemia	Loke et al., 2017

### AD and select DEGs in blood of wild-type animals: longitudinal analysis

In wild-type controls (Figure 1B, Supplementary Table 2), 21 AD-associated DEGs (Table 1) were regulated in the same direction (Egfl7, Efna1, Unc5c, Fn1, Ddit1, Efemp1, Fabp3, Pdk4, Ltc4s, C1qb, Il33, Bcam, Cgnl1, C1qc, Aebp1, Ptgis, Saa3, Fcrls, C1qa, C4b, and Ednrb) as in *3xTg-AD* animals and thus are not distinct for neuropathology onset in *3xTg-AD* animals. One AD-marked DEG (5S_rRNA) went from upregulated in wild-type to downregulated in *3xTg-AD* mice. In contrast, 17 AD-associated DEGs (Table 1) went from upregulated in *3xTg-AD* mice to downregulated in wild-type at 6.5 mo (Srgap1, Lrfn5, Myt1l, Kndc1, Pcsk2, Cacna2d3, Prkar1b, Pcdh9, Mei, Foxp2, Lin7a, Pth2r, St8sia3, Slc17a7, Rbp4, Etv4, and Ncan), which serve as potential blood biomarkers during the MCI phase of AD pathology.

Of those DEGs not necessarily selective for AD pathology (Table 2), Sgtb, Pak6, Kcnk3, Des, Ltc4s, and Gpd1 were commonly regulated in the same direction for both *3xTg-AD* and wild-type mice. However, Kcnq4 for hearing loss (Lee et al., 2021) was upregulated in *3xTg-AD* mice and downregulated in wild-type mice. The majority (89%) of these randomly selected DEGs in *3xTg-AD* mice (Figure 1A, Supplementary Table 1) did not appear as DEGs for wild-type mice (Figure 1B, Supplementary Table 2) including those for Parkinson's (e.g., Tox3, Pak6, and Gucy2c) and Huntington's (e.g., Tgm1 and Fam171b) pathology, aging (e.g., Abcc9 and Postn), potential destruction of the blood brain barrier (Jam3), vascular remodeling (e.g., EphB4 and Angptl6), lipid disorders (e.g., Acat3 and Cidec), and inflammation (e.g., Alox8). In addition, note that the most extreme DEGs in *3xTg-AD* animals (log2 fold change > 15) such as downregulated H2-Q2 [“non-classical” Major Histocompatibility Complex Class 1 molecule (Huh et al., 2000)] and Tdrd5 [processes small non-coding RNAs for spermatogenesis (Ding et al., 2018)] and upregulated Sult4a1 [brain-specific sulfotransferase involved in neuronal development & function (Culotta et al., 2020)], Cyp2f2 [cytochrome P450 enzyme highly expressed in lungs (Li et al., 2011)], and Rag2 [crucial for immune development via V(D)J recombination for generation of antigen receptors on B & T lymphocytes (Gennery, 2019)] did not overlap as DEGs with wild-type animals (Figure 1B, Supplementary Table 2).

For a complete list of overlapping genes and directional regulation of DEG expression among *3xTg-AD* and wild-type B6129 mice, see Supplementary Table 3. For characterized non-coding RNAs differentially regulated among groups, small RNAs include snoRNAs Snora21 & C/D box 59A (Snord59a) and Gm54761 miRNA. LncRNAs regulated in opposite directions among groups include 9530022L04Rik and Gm13270. In addition, note that a total of 431 DEGs in *3xTg-AD* mice were “reversed” in expression in wild-type mice (Supplementary Table 4). Of all the AD DEGs (Table 1), 103 (43%) were included in this list, with Srgap1, Lrfn5, Myt1l, Kndc1, Pcsk2, Cacna2d3, Prkar1b, Pcdh9, Mei1, Foxp2, Lin7a, Pth2r, St8sia3, Slc17a7, Rbp4, and Etv4 as significantly reversed in the opposite direction of expression in wild-type mice relative to *3xTg-AD*. Other significantly reversed coding genes include Frs3, IQ motif and Sec7 domain 3 (Iqsec3), Masp1, Shisa2, Hhatl, Lmcd1, Ppfia2, Spink10, Slc13a4, and Kcnq4 (Supplementary Table 4). Conversely, a total of 478 DEGs in wild-type mice were reversed in the opposite direction of expression in *3xTg-AD* mice with inclusion of significant gene markers indicated in the *vice versa* analysis (Supplementary Table 5).

### AD and select DEGs in blood of *3xTg-AD* animals treated with CBD: longitudinal analysis

Relative to *3xTg-AD* animals (Figure 1A, Supplementary Table 1), 57 AD-selective DEGs were regulated in the same direction (Map2k3os lncRNA, Map1lc3b, Uqcr10, Pfdn5, Cox7a2, Uqcrh, Mrc1, Ndufa7, Igfbp5, Lpar1, Mertk, Rbms3, Ddr2, Slc4a4, Synpo2, Serping1, Dagla, Grb14, Eda2r, Dab2, C1qb, Cyp1b1, Sfrp1, Fgf2, Pth1r, Myh4, Gas6, Mgp, Ptprd, Kcnn3, Tmem119, Dcn, Acss3, Fbln1, Scara3, Rarres2, C1s1, Mme, Ppp1r3c, Prox1, Pck1, Prelp, Atp1a2, and C1qa; common with wild-type: Efemp1, Pdk4, Ltc4s, Il33, Bcam, Cgnl1, C1qc, Aebp1, Ptgis, Saa3, C4b, Ednrb) in CBD-treated *3xTg-AD* animals (Figure 1C, Supplementary Table 6). Three AD-marked DEGs (5s_rRNA, Peg3, and Tagln3) went from downregulated in *3xTg-AD* mice to upregulated in CBD-treated *3xTg-AD* mice. In contrast, 2 AD-marked DEGs went from upregulated in *3xTg-AD* mice to downregulated in CBD-treated *3xTg-AD* mice (Smad9 and Rgs7bp). Note that most (=178, 75%) of the remaining AD-marked DEGs (Figure 1A, Table 1) were no longer DEGs in *3xTg-AD* animals following CBD treatment (Figure 1C, Supplementary Table 6).

Of those DEGs indicated in conditions independent of or in addition to AD pathology, Timm8b, Susd4, Tmsb4x, Kcnt2, Lockd lncRNA, Jam3, Kcnk3, Des, Klhdc7a, Abca8a, Gpd1, Plin1, and Thrsp were commonly regulated in the same direction for both *3xTg-AD* and CBD-treated *3xTg-AD* mice. The majority (76%) of these randomly selected DEGs in *3xTg-AD* mice (Figure 1A, Table 2) did not appear as DEGs following CBD treatment (Figure 1C, Supplementary Table 6), noting absence of some select markers for neurological aging (e.g., Abcc9 and Postn), hypercholesterolemia (e.g., Acat3), and inflammation (e.g., Alox8). In addition, note that the most extreme DEGs in *3xTg-AD* animals (log2 fold change > 15) as H2-Q2 and Sult4a1 were no longer DEGs in comparison with the CBD-treated *3xTg-AD* group, whereas Tdrd5, Cyp2f2, and Rag2 remained (Figure 1C, Supplementary Table 6). For select endocannabinoid-related genes, the CBD receptor gene Gpr6 (Laun et al., 2019) was no longer indicated as a DEG but Dagla enzyme gene [for 2-arachidonoglycerol (2-AG) production; Schuele et al., 2022] remained upregulated regardless following CBD treatment in *3xTg-AD* mice.

For a complete list of overlapping genes and directional regulation of DEG expression among CBD-treated *3xTg-AD* and *3xTg-AD* mice, see Supplementary Table 7. A non-coding RNA regulated in opposite directions among groups includes the snRNA 7SK (or RN7SK). In addition, note that a total of 284 DEGs in *3xTg-AD* mice were reversed in the direction of expression in CBD-treated *3xTg-AD* mice (Supplementary Table 8). Of all the AD DEGs, 72 (30%) were included in this list, with Peg3, Tagln3, Smad9, and Rgs7bp as significantly expressed in the opposite direction in CBD-treated *3xTg-AD* mice relative to *3xTg-AD* mice.

### AD and select DEGs in blood of wild-type animals treated with CBD: longitudinal analysis

Of the DEGs marked with AD in *3xTg-AD* animals as common with wild-type animals (Figures 1A, B, Supplementary Tables 1, 2) and CBD-treated *3xTg-AD* animals (Figure 1C, Supplementary Table 6), 6 were regulated in the same direction as Pdk4, C1qb, C1qc, Ptgis, C4b, and Ednrb in CBD-treated wild-type animals (Figure 1D, Supplementary Table 9). Fabp3 was commonly upregulated among wild-type (Figure 1B, Supplementary Table 2) and CBD-treated wild-type animals (Figure 1D, Supplementary Table 9). As upregulated genes in *3xTg-AD* mice, Srgap1 and Myt1l remained downregulated regardless of CBD treatment in wild-type animals, whereas these genes were no longer DEGs in CBD-treated *3xTg-AD* animals. For genes under other classifications independent of AD pathology, Gpd1 for lipid metabolism was commonly upregulated among wild-type animals and CBD-treated wild-type animals.

In comparison with CBD-treated *3xTg-AD* animals (Figure 1C, Supplementary Table 6), nine AD DEGs as C1qa, C1qb, Cyp1b1, Sfrp1, Myh4, C1s1, Ppp1r3c, Pck1, and Atp1a2 were regulated in the same direction in CBD-treated wild-type animals. One AD DEG, Pth1r, was upregulated in CBD-treated *3xTg-AD* animals but downregulated in CBD-treated wild-type animals. As downregulated in *3xTg-AD* animals but upregulated in CBD-treated *3xTg-AD* animals, Peg3 is downregulated in CBD-treated wild-type animals (Figure 1D, Supplementary Table 9). For other select genes not necessarily related to AD pathology, Gpd1 is commonly upregulated across all groups regardless of AD pathology and CBD treatment. Susd4, Thrsp, and Cidec genes are commonly regulated among all groups except for wild-type mice without CBD where they are not indicated as DEGs. Nt5dc, Angptl6, Plin1, and Plin4 are commonly regulated among *3xTg-AD* mice and the CBD-treated wild-type group. Tox3 and Postn are downregulated and upregulated, respectively, in *3xTg-AD* mice but, conversely, upregulated and downregulated, respectively, in the CBD-treated wild-type group. The majority (81%) of the randomly selected DEGs in *3xTg-AD* mice (Figure 1A, Supplementary Table 1) did not appear as DEGs in CBD-treated wild-type animals (Figure 1D, Supplementary Table 9). Of the most extreme DEGs as identified in *3xTg-AD* animals (|log2 fold change| > 15; downregulated H2-Q2 and Tdrd5 and upregulated Sult4a1, Cyp2f2, and Rag2), H2-Q2, Tdrd5, and Cyp2f2 were upregulated DEGs in CBD-treated wild-type animals.

For a complete list of overlapping genes and directional regulation of DEG expression among CBD-treated wild-type and untreated wild-type mice, see Supplementary Table 10. One lncRNA showing opposite regulation between these groups was Gm43868. For overlapping DEG expression among CBD-treated *3xTg-AD* mice and CBD-treated wild-type mice, see Supplementary Table 11. Non-coding RNAs regulated in opposite directions among these groups include the miRNA Gm56228 and lncRNAs Gm43868 and Gm27252. In addition, note that a total of 238 DEGs in wild-type mice were reversed in direction of expression in CBD-treated wild-type mice (Supplementary Table 12). Significantly reversed genes following CBD treatment of wild-type mice include Rbp4, S100 calcium binding protein B (S100b), protein kinase D1 (Prkd1), tumor necrosis factor alpha induced protein 6 (Tnfaip6), and the lncRNA Gm43868. To help ascertain how CBD may differentially impact *3xTg-AD* mice vs. wild-type mice, an analysis revealed a total of 347 DEGs in CBD-treated *3xTg-AD* that were reversed in the direction of expression in CBD-treated wild-type mice (Supplementary Table 13). Significantly reversed genes in CBD-treated wild-type mice relative to CBD-treated *3xTg-AD* mice include the miRNA Gm56228, lncRNAs 2010310C07Rik and Gm27252, Tdrd5, F-box and leucine rich repeat protein 15 (Fbxl15), 5-hydroxytryptamine (serotonin) receptor 1B (Htr1b), multiple PDZ domain crumbs cell polarity complex component (Mpdz), ADCYAP receptor type I (Adcyap1r1), FYVE, RhoGEF and PH domain containing 1 (Fgd1), protein interacting with cyclin A1 (Proca1), Pth1r, Tmem132a/e, Sorbin and SH3 domain containing 2 (Sorbs2), Peg3, Formin homology 2 domain containing 3 (Fhod3), Peroxisomal biogenesis factor 11 gamma (Pex11g), and Slc26a1. Conversely, 300 DEGs in CBD-treated wild-type mice were reversed in CBD-treated *3xTg-AD* mice with inclusion of significant gene markers indicated in the *vice versa* analysis (Supplementary Table 14).

### Pathways of AD onset: longitudinal blood analysis

**Figure 2:**
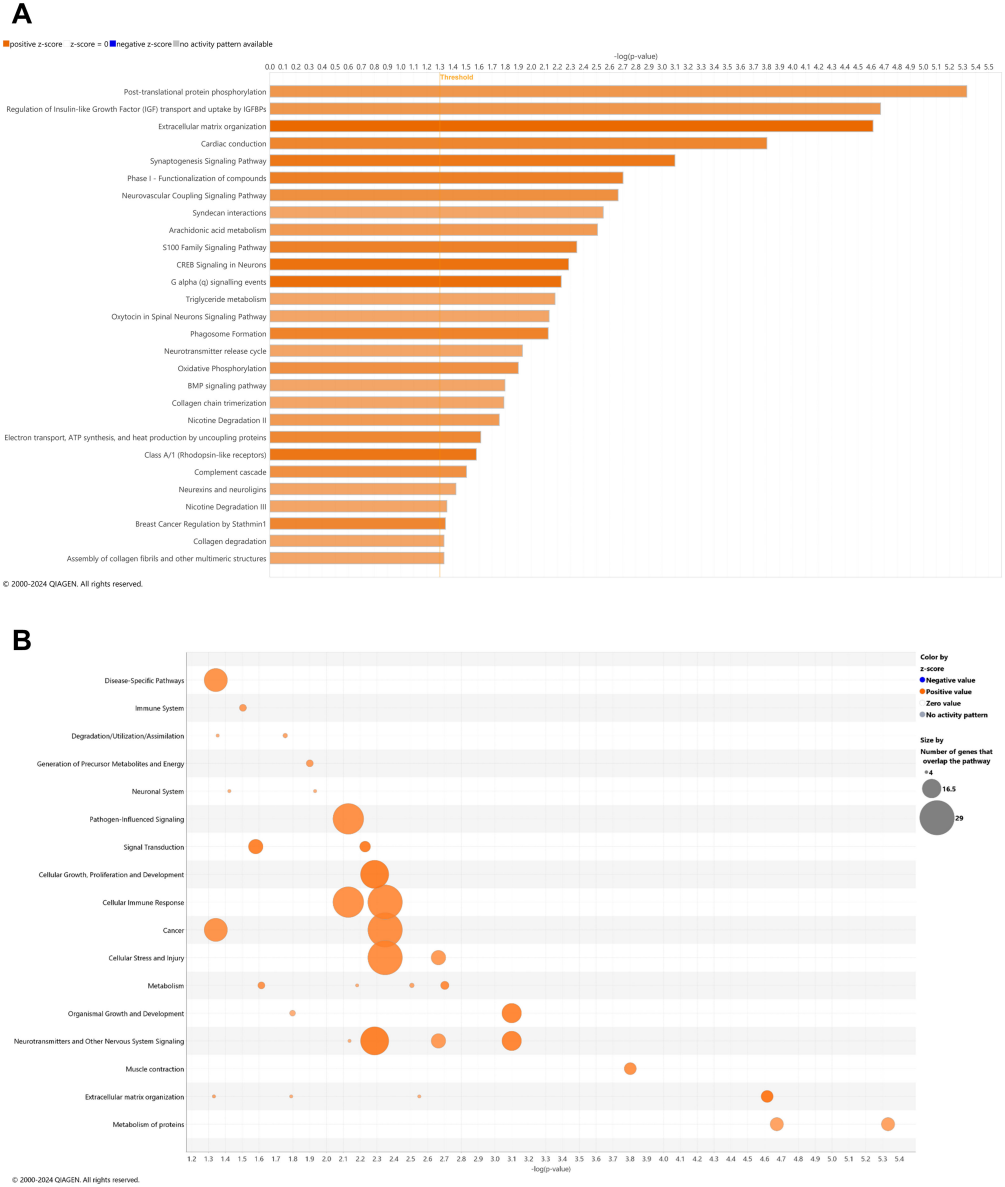
Cell signaling pathways and categories in whole blood marking Alzheimer's disease onset. **(A)** Canonical pathways that significantly increased (orange, 28) or decreased (blue, 0) from 4.5 mo (0 wks) to 6.5 mo (8 wks) in *3xTg-AD* animals. **(B)** Bubble plots of the number of genes that overlap with major pathway categories with size of bubble directly indicating the amount of overlap; increase = orange and decrease = blue. The Ingenuity pathway analysis setting was set at a log2 fold change cutoff at 1.0 up and −1.0 down (*p*-value ≤ 0.05). The significance of canonical pathways was determined at a –log(*p*-value) >1.3 and absolute *z*-score > 2.0. Data were obtained from *n* = 5 male mice. This figure was generated through the use of QIAGEN IPA (QIAGEN Inc., https://digitalinsights.qiagen.com/IPA) (Krämer et al., 2014).

With comparison of whole blood of *3xTg-AD* animals at AD onset (6.5 mo, 8 wks vehicle treatment) vs. cognitive impairment (4.5 mo, 0 wks vehicle treatment; Figure 1A, Supplementary Table 1), 28 canonical pathways were upregulated (Figure 2A; –log *p*-value >1.3 and absolute *z*-score > 2.0) Using a bubble plot analysis with consideration of the abundance of gene overlap with various pathways (Figure 2B), the most prominent categories are disease-specific pathways; pathogen-influenced signaling; cellular growth proliferation and development; cellular immune response; cancer; cellular stress and injury; and neurotransmitters and other nervous system signaling. At least in part, the lack of downregulated pathways in *3xTg-AD* animals may be attributed to 183 (41%) of downregulated DEGs (Figure 1A) that have not been sufficiently characterized and annotated yet.

**Figure 3:**
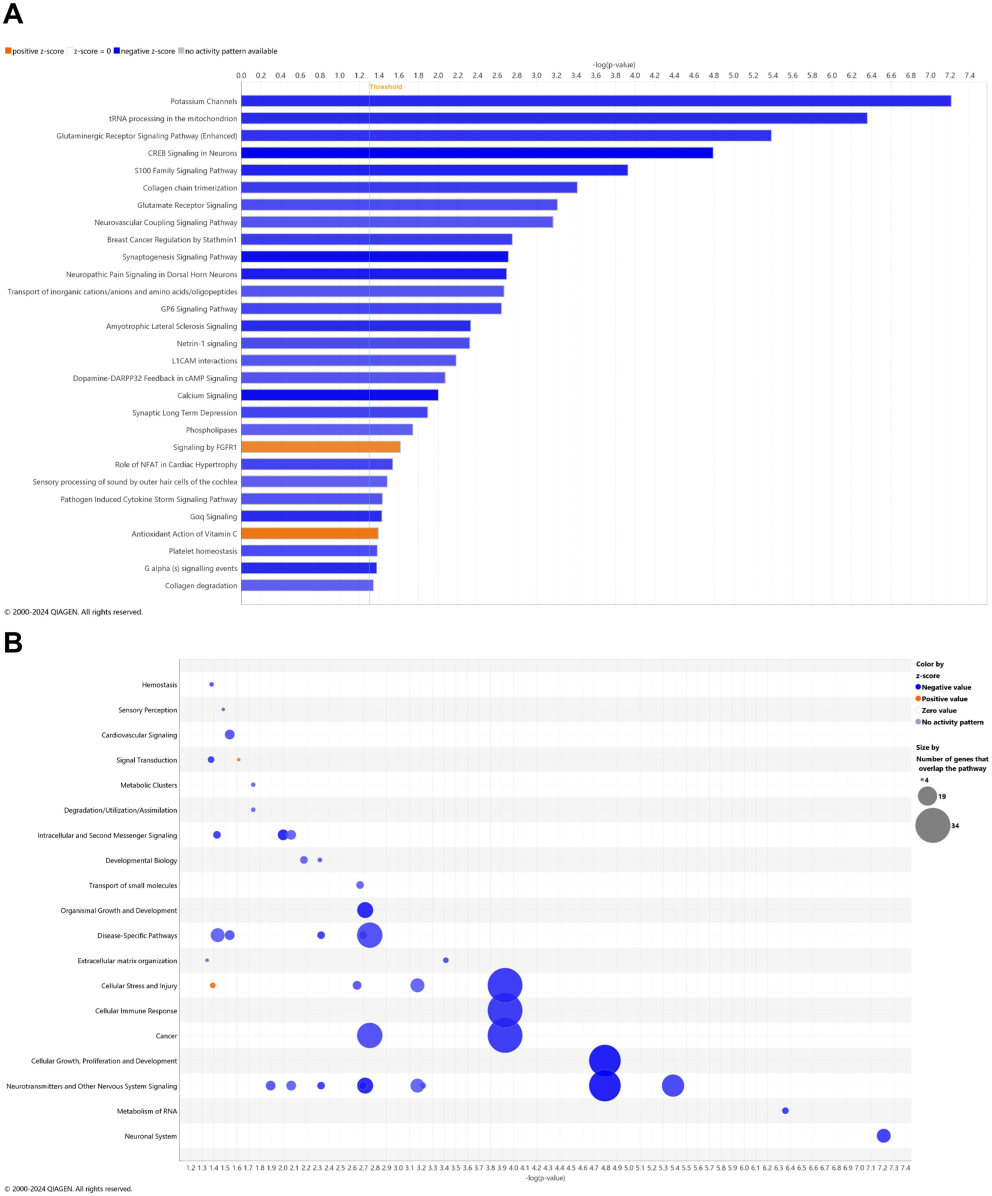
Cell signaling pathways and categories in whole blood of age-matched, wild-type mice. **(A)** Canonical pathways that significantly increased (orange, 2) or decreased (blue, 27) from 4.5 mo (0 wks) to 6.5 mo (8 wks) in wild-type B6129 animals. **(B)** Bubble plots of the number of genes that overlap with major pathway categories with size of bubble directly indicating the amount of overlap; increase = orange and decrease = blue. The Ingenuity pathway analysis setting was set at a log2 fold change cutoff at 1.0 up and −1.0 down (*p*-value ≤ 0.05). The significance of canonical pathways was determined at a –log *p*-value greater >1.3 and absolute *z*-score of >2.0. Data were obtained from *n* = 3 male mice. For a report on reversal of significant pathways in wild-type B6129 vs. *3xTg-AD* mice, see Supplementary Table 15 (*vice versa* as Supplementary Table 16). This figure was generated through the use of QIAGEN IPA (QIAGEN Inc., https://digitalinsights.qiagen.com/IPA) (Krämer et al., 2014).

Young adult aging from 4.5 to 6.5 mo in the wild-type mice is not likely a substantial shift in the animal's genome regulation toward pathology (Quintana et al., 2021; Lourenco et al., 2017), whereby it was suspected that only pathways of development (e.g., neurogenesis and skeletal muscle growth) would be relevant, if anything. Surprisingly, 27 pathways were downregulated in wild-type mice including synaptogenesis, CREB signaling, and glutamate receptor signaling (Figure 3A) with only two upregulated pathways as FGFR1 signaling and antioxidant action of vitamin C (Figure 3A). Accordingly, downregulated DEGs indicate a prominent decrease in disease-specific pathways; cellular stress and injury; cellular immune response, cancer; cellular growth, proliferation, and development; and neurotransmitters and other nervous system signaling (Figure 3B). Pathways that are significantly reversed in wild-type relative to *3xTg-AD* mice (Supplementary Table 15) and *vice versa* (Supplementary Table 16) include endocannabinoid neuronal synapse pathway; breast cancer regulation by Stathmin 1; neurotransmitter release cycle; K^+^ channels; molecular mechanisms of cancer; CREB signaling in neurons, S100 family signaling; neurovascular coupling signaling; synaptogenesis signaling; and extracellular matrix organization.

### Pathways of AD onset relative to wild-type animals with CBD treatment: longitudinal blood analysis

**Figure 4:**
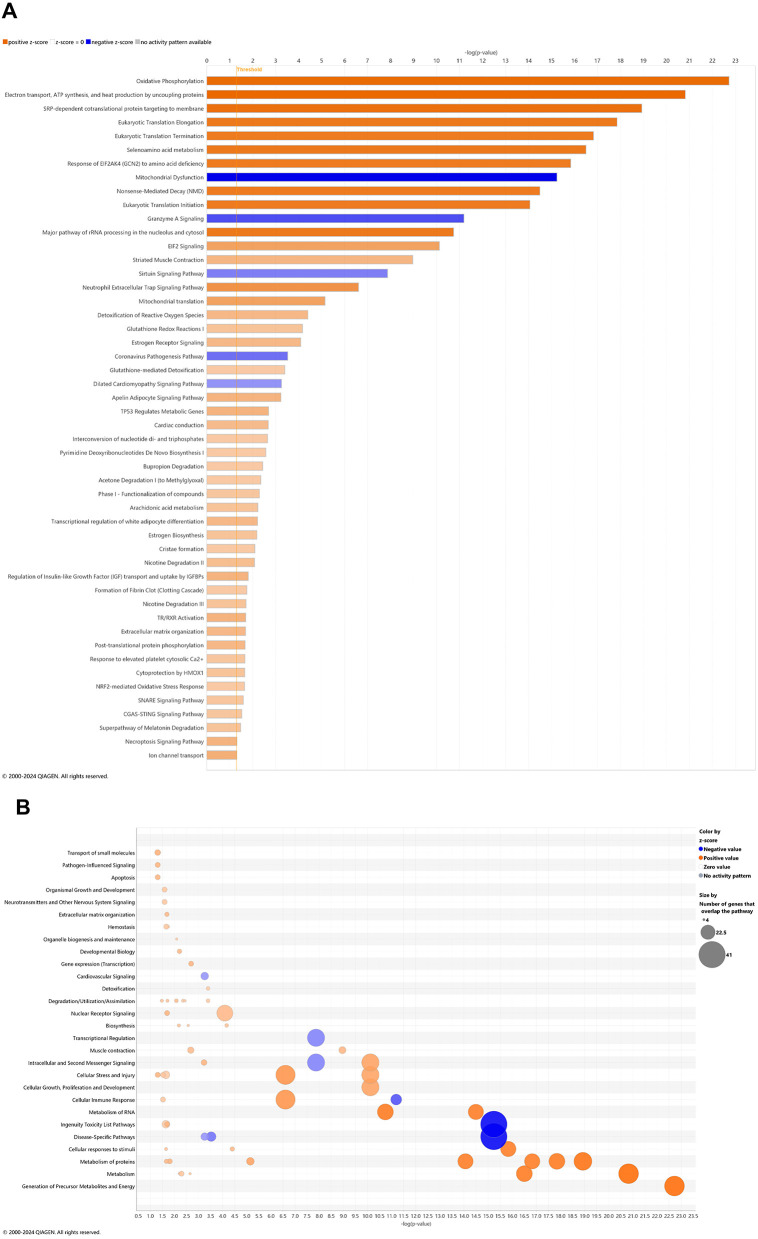
Cell signaling pathways and categories in whole blood with cannabidiol treatment of Alzheimer's disease onset. **(A)** Canonical pathways that significantly increased (orange, 45) or decreased (blue, 5) from 4.5 mo (0 wks) to 6.5 mo (8 wks) in cannabidiol (CBD)-treated *3xTg-AD* animals. **(B)** Bubble plots of the number of genes that overlap with major pathway categories with size of bubble directly indicating the amount of overlap; increase = orange and decrease = blue. The Ingenuity pathway analysis setting was set at a log2 fold change cutoff at 1.0 up and −1.0 down (*p*-value ≤ 0.05). The significance of canonical pathways was determined at a –log(*p*-value) greater >1.3 and absolute *z*-score of >2.0. Data were obtained from *n* = 5 male mice. For a report on reversal of significant pathways in CBD-treated *3xTg-AD* vs. *3xTg-AD* vehicle mice, see Supplementary Table 17. This figure was generated through the use of QIAGEN IPA (QIAGEN Inc., https://digitalinsights.qiagen.com/IPA) (Krämer et al., 2014).

There is a reasonable premise that CBD may address known pathways of Alzheimer disease pathogenesis (Cummings et al., 2025; Liu, 2024). In whole blood of CBD-treated *3xTg-AD* animals, 45 and 5 pathways were significantly upregulated and downregulated, respectively (Figure 4A). For the most prominent upregulated pathways with –log *p*-value > 10, oxidative phosphorylation; electron transport, ATP synthesis, and heat production by uncoupling proteins; SRP-dependent co-translational protein targeting to membrane; eukaryotic translation initiation, elongation, and termination; seleno-amino acid metabolism, response of EIF2AK4 (GCN2) to amino acid deficiency; non-sense-mediated decay; major Pathway of rRNA processing in the nucleolus and cytosol; and EIF2 signaling. The abundance of gene overlap for upregulated pathways is prominent for nuclear receptor signaling, cellular immune response, and metabolism of protein (Figure 4B). Downregulated gene overlap appears for ingenuity toxicity list and disease-specific pathways (Figure 4B). Pathways muted in CBD-treated *3xTg-AD* relative to untreated *3xTg-AD* mice primarily center on the Smad9 gene involved in angiogenesis and tumor development (Supplementary Table 17).

**Figure 5:**
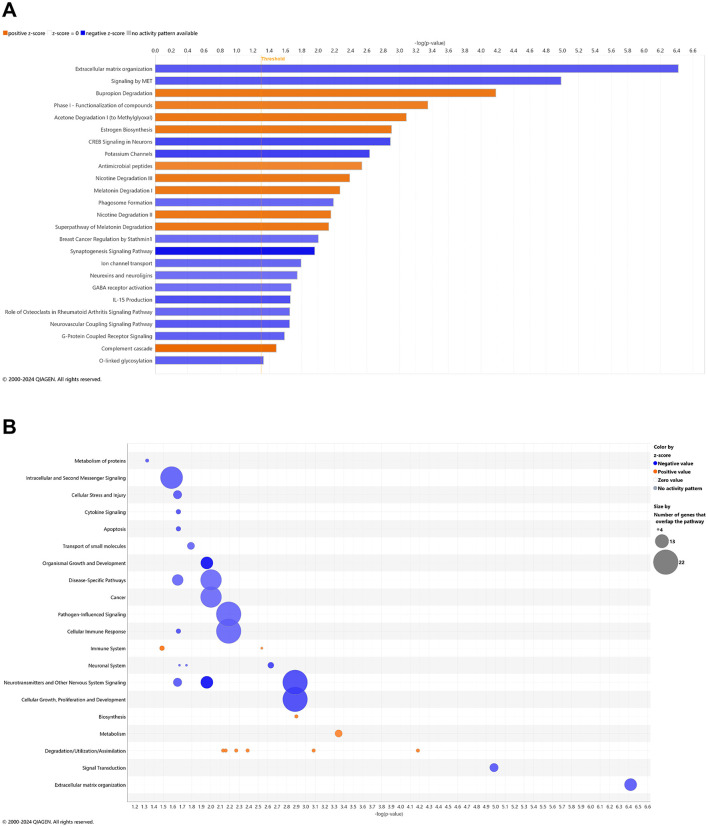
Cell signaling pathways and categories in whole blood of age-matched, wild-type mice treated with cannabidiol. **(A)** Canonical pathways that significantly increased (orange, 10) or decreased (blue, 15) from 4.5 mo (0 wks) to 6.5 mo (8 wks) in cannabidiol (CBD)-treated wild-type B6129 animals. **(B)** Bubble plots of the number of genes that overlap with major pathway categories with size of bubble directly indicating the amount of overlap; increase = orange and decrease = blue. The Ingenuity pathway analysis setting was set at a log2 fold change cutoff at 1.0 up and −1.0 down (*p*-value ≤ 0.05). The significance of canonical pathways was determined at a –log *p*-value greater >1.3 and absolute *z*-value score of >2.0. Data were obtained from *n* = 5 male mice per group. For a report on pathways that were flipped in the opposite direction of regulation in CBD-treated vs. untreated wild-type mice, see Supplementary Table 18. For a report on downregulated pathways that emerged with CBD treatment in wild-type but not *3xTg-AD* mice, see Supplementary Table 19. For pathways that were commonly upregulated in both CBD-treated groups to a similar extent [–log(*p*-value) ≈ 2] but with a relatively enhanced *z*-score in *3xTg-AD* animals, see Supplementary Table 20. This figure was generated through the use of QIAGEN IPA (QIAGEN Inc., https://digitalinsights.qiagen.com/IPA) (Krämer et al., 2014).

In whole blood of CBD-treated wild-type animals, 10 and 15 pathways were significantly upregulated and downregulated, respectively (Figure 5A). With a commonly upregulated pathway as complement cascade signaling, downregulated pathways in CBD-treated wild-type animals that were upregulated in untreated *3xTg-AD* animals include extracellular matrix organization; CREB signaling in neurons; synaptogenesis signaling; neurexins and neuroligins; neurovascular coupling; and breast cancer regulation by Stathmin 1 (Figures 2A, 5A). As absent for significance (P>0.05) in untreated wild-type mice (Figure 3A), upregulated pathways in CBD-treated wild-type mice (Figure 5A) include melatonin and nicotine degradation and estrogen biosynthesis as similar to CBD-treated *3xTg-AD* mice (Figure 4A). Downregulated pathways in untreated wild-type mice that remained downregulated with CBD treatment include K^+^ channels, CREB signaling in neurons, neurovascular coupling, synaptogenesis signaling, and breast cancer regulation by Stathmin 1 (Figures 3A, 5A). Relative to increases in CBD-treated *3xTg-AD* mice (Figure 4A), pathways of extracellular matrix organization and ion transport signaling were decreased in CBD-treated wild-type mice (Figure 5A). Within CBD-treated wild-type animals, patterns of overlapping genes were primarily downregulated as intracellular second messenger signaling; disease specific pathways; cancer; pathogen-influenced signaling; cellular immune response; neurotransmitters and other nervous system signaling; and cellular growth, proliferation, and development (Figure 5B). Pathways that were flipped in the opposite direction of regulation in CBD-treated relative to untreated wild-type mice include hepatic fibrosis and neutrophil extracellular trap signaling (Supplementary Table 18). Downregulated pathways that emerged with CBD treatment in wild-type but not *3xTg-AD* include phagosome formation; CREB signaling in neurons; S100 family signaling; G-protein coupled receptor signaling; breast cancer regulation by stathmin 1; molecular mechanisms of cancer; and class B/2 (secretin family receptors; Supplementary Table 19). Pathways that were commonly upregulated in both CBD-treated groups to a similar extent [–log(*p*-value) ≈ 2] but with a relatively enhanced *z*-score in *3xTg-AD* animals include regulation of IGF transport and uptake by IGFBPs and post-translational protein phosphorylation, primarily based on the expression of the Tmem132a gene (Supplementary Table 20).

### Select DEGs in blood of *3xTg-AD* relative to wild-type animals with and without CBD treatment: cross-sectional analysis

With longitudinal comparisons among groups being the most rigorous for analysis, we also sought to analyze what DEGs may distinguish groups at the 6.5 mo timepoint following 8 wks of vehicle treatment or CBD. Cross-sectional comparison of whole blood from *3xTg-AD* animals at AD onset vs. wild-type animals (both at 6.5 mo following 8 weeks of vehicle treatment; Supplementary Table 21) identified 523 significantly downregulated and 494 significantly upregulated DEGs. The most extreme expression alterations (log2 fold change ≥ 20) are the same as the downregulated H2-Q2 and Tdrd5 genes marked for the longitudinal (6.5 vs. 4.5 mo) *3xTg-AD* analysis (Figure 1A, Supplementary Table 1). Exact matches for AD-selective genes as determined in the longitudinal *3xTg-AD* analyses for onset of AD (Figure 1A, Supplementary Table 1) include Ramp3, Tamalin, Sema4c, the lncRNA Map2k3os, Rin1, F12, Acvr1, Iqck, Tagln3, Scg5, Wfs1, Cacna2d4, Ncr1, Esr1, Ndufa7, Cox7a2, Acacb, Gls2, Bmp4, Mgat3, Ppp1r3c, Vgf, Gpr6, Hapln2, Oprd1, Ntsr1, Lrfn5, Nap1l2, Kndc1, Pcsk2, Cckbr, Tmem63c, Prkar1b, the miRNA Mir144, Mei1, Tacr3, Lin7a, Gria4, Npsr1, Scara3, Ankrd36, Sfrp1, Insm1, Snap91, St8sia3, Pcdh9, Rgs7, Rbp4, Chrna2, Etv4, Pld6, Adamts13, Kcnk2, Slc17a7, Prok2, and Ncan. In CBD-treated *3xTg-AD* vs. vehicle mice, all of these genes marked for AD pathology were either reversed in expression (downregulated to upregulated: Ramp3, Sema4c, Rin1, Acvr1, Iqck, Tagln3, Scg5, and Cacna2d4; upregulated to downregulated: Mgat3, Tmem63c, Prkar1b, Kcnk2, and Ncan) or no longer appeared as a DEG in *3xTg-AD* mice (Supplementary Table 22). Another noteworthy finding is that apolipoprotein E (Apoe; Pendse et al., 2009) was a downregulated DEG in *3xTg-AD* mice relative to wild-type (Supplementary Table 21), an observation no longer apparent with CBD treatment (Supplementary Table 22).

With continuing consideration of DEGs not necessarily selective for AD pathology originally identified in longitudinal analyses of *3xTg-AD* mice (Figure 1A, Supplementary Table 1), C920006O11Rik, Timm8b, Necab2, Gprasp2, Kcng2, Asic4, Kcnq4, Lockd, Angptl6, Sidt1, and Acat3 also appeared as DEGs in the cross-sectional analysis of vehicle *3xTg-AD* vs. wild-type mice (Supplementary Table 21). Of these DEGs, only two remained (C920006O11Rik and Angptl6) and were reversed in direction of expression from downregulated to upregulated in CBD-treated *3xTg-AD* animals (Supplementary Table 22). For DEGs that overlapped in longitudinal blood analyses of *3xTg-AD* vs. wild-type B6129 vehicle (week 0–8; age, 4.5 to 6.5 mo) mice while opposite in direction of expression among groups (Supplementary Table 3), five ncRNAs (Gm13270, Gm17096, Gm42462, Gm5067, and Snora21), A830018L16Rik, Hhat1, Iqsec3, Limcd1, Masp1, Ppfia2, Slc13a4, and Spink10 appeared again as DEGs in cross-sectional analyses of *3xTg-AD* mice vs. wild-type (6.5 mo; Supplementary Table 21). Only two of these genes (Gm17096 and Slc13a4) remained in the CBD-treated *3xTg-AD* group vs. *3xTg-AD* vehicle, whereby CBD treatment again reversed their direction of expression relative to vehicle (Supplementary Table 22). Of all the genes common to longitudinal and cross-sectional analyses in *3xTg-AD* vs. wild-type animals above, note that Kndc1, Sfrp1, Rbp4, and Etv4 also appear as upregulated DEGs in CBD-treated wild-type vs. vehicle animals at 6.5 mo of age (Supplementary Table 23). For CBD-treated *3xTg-AD* vs. CBD-treated wild-type mice cross-sectional analyses in blood, downregulated DEGs include F12, Ncr1, and Apoe and upregulated DEGs include Ndufa7, Cox7a2, miR144, Ankrd36, Prok2, Timm8b, Lockd, Iqsec3, and Spink10 (Supplementary Table 24).

### Select DEGs in brains of *3xTg-AD* relative to wild-type animals with and without CBD treatment: cross-sectional analysis

**Figure 6:**
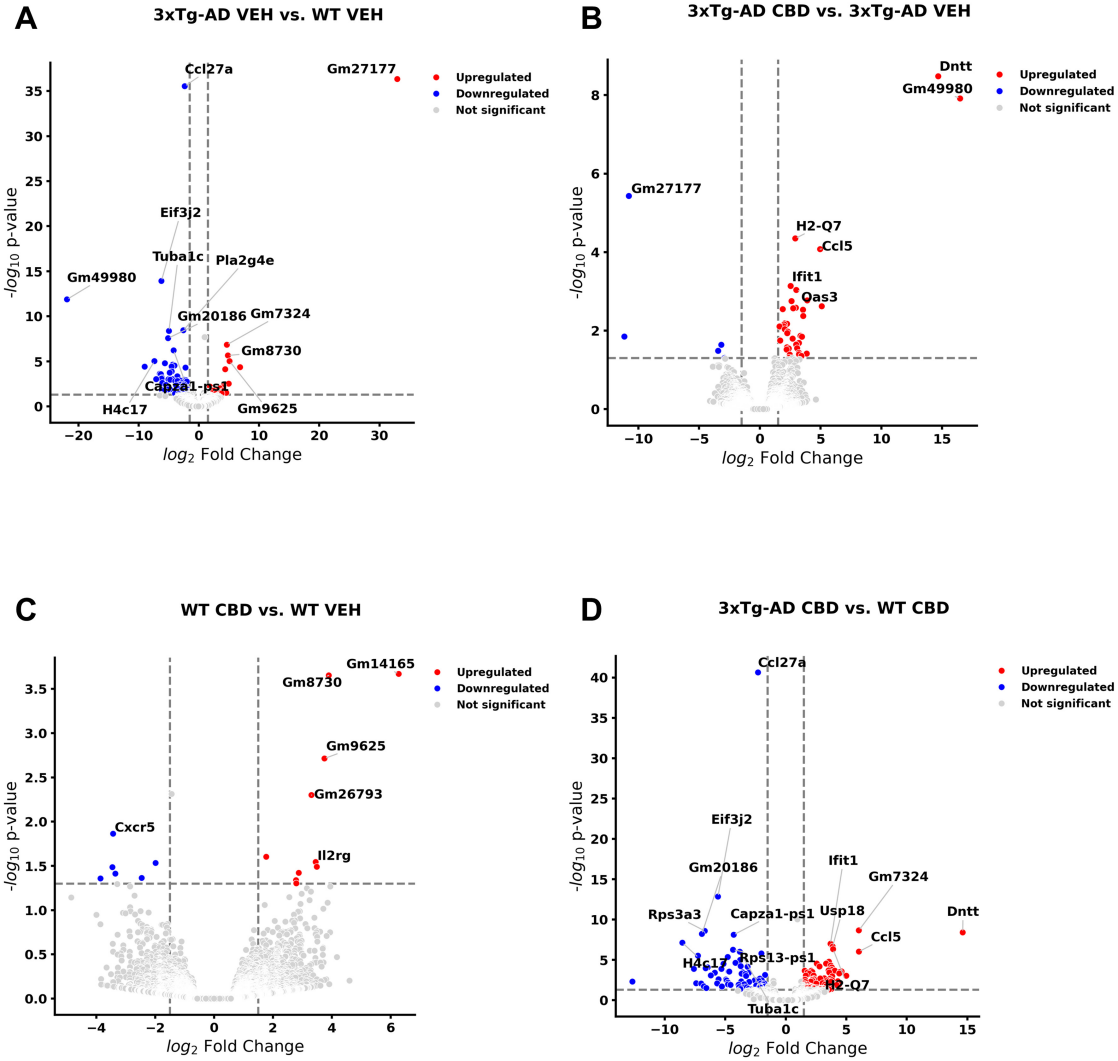
Volcano plots of cross-sectional profiles collected from whole brain: effect of Alzheimer's disease onset and cannabidiol. Genes that were upregulated (red), downregulated (blue), and were not significantly altered (light gray). **(A)** Genes that were altered in 6.5 mo *3xTg-AD* vs. age-matched B6129 mice; 72 and 28 genes were less and more in expression, respectively (see Supplementary Table 25). **(B)** Cannabidiol (CBD)-treated *3xTg-AD* vs. *3xTg-AD* vehicle mice; 5 and 38 genes were less and more in expression, respectively (see Supplementary Table 26). **(C)** CBD-treated B6129 vs. B6129 vehicle mice; 7 and 10 genes were less and more in expression, respectively (see Supplementary Table 27). **(D)** CBD-treated *3xTg-AD* vs. CBD-treated B6129 mice; 73 and 98 genes were less and more in expression, respectively (see Supplementary Table 28). Data were obtained from *n* = 3–5 male mice per group. For corresponding cross-sectional comparisons of blood samples across respective groups, see Supplementary Tables 21–24. For cross-sectional of gene markers that were matched across blood and brain compartments, see Supplementary Tables 29–32. This figure was generated through the use of QIAGEN IPA (QIAGEN Inc., https://digitalinsights.qiagen.com/IPA) (Krämer et al., 2014).

As a basis of central nervous function, we also examined cross-sectional analyses of DEGs among all study groups at the 6.5 mo timepoint following 8 wks of vehicle treatment or CBD in brain samples. With cross-sectional comparison of brains of *3xTg-AD* animals at AD onset relative to wild-type (6.5 mo, 8 wks vehicle treatment; Figure 6A, Supplementary Table 25), 72 and 28 DEGs were significantly downregulated and upregulated, respectively. AD-selective genes include ribonuclease A family member 6 (Rnase6; Seto et al., 2022; Bolivar et al., 2024), membrane-spanning 4-domains, subfamily A, member 1 (Ms4a1; Deming et al., 2019), oxidative stress-induced growth inhibitor 1 (Osgin1; Kang et al., 2023), C-C motif chemokine receptor 1 & 6 (Ccr1/6; Halks-Miller et al., 2003; Subramanian et al., 2010; D'Angelo et al., 2020), complement c5a receptor 2 (C5ar2; Carvalho et al., 2022), phospholipase A2 group IVE (Pla2g4e; Perez-Gonzalez et al., 2020), NLR family, CARD domain containing 4 (Nlrc4; Saadi et al., 2020), Ubc (Nguyen et al., 2024), serine (or cysteine) peptidase inhibitor, clade A, member 3N (Serpina3n; Saroja et al., 2022), Il15 (Clark et al., 2021; Janelidze et al., 2018), Aqp6 (Amro et al., 2023), exocyst complex component 3-like 2 (Exoc3l2; Wu et al., 2017; Seshadri et al., 2010), ubiquitin specific peptidase 18 (Usp18; Widjaya et al., 2023; Xiang et al., 2018), Oncostatin m (Osm; Yu et al., 2023; Whelan et al., 2019), Kcnn4 (Kosoy et al., 2022; Maezawa et al., 2012), interferon-activated gene 204 (Ifi204; Green et al., 2022), and C-X-C motif chemokine ligand 13 (Cxcl13; Karaahmet et al., 2022; Figure 6A, Supplementary Table 25). In the corresponding cross-sectional analysis in blood for *3xTg-AD* vs. wild-type mice, Ubc is an exact match whereas other homolog DEGs appear as Rnase1, Ms4a4b, Ccr5/9, Aqp11, Usp46, and Cxcl14 (Supplementary Table 26). In addition, note that Ms4a7 and Kcnn3 appear as homolog genes in longitudinal analyses as AD onset in *3xTg-AD* mice (6.5 vs. 4.5 mo; Supplementary Table 1). Other notable genes commonly regulated among the blood and brain compartments include eukaryotic translation initiation factor 3, subunit J2 (Eif3j2), enolase 1b (Eno1b), guanylate binding protein 2 (Gbp2b), H4 clustered histone 17 (H4c17) mitochondrial ribosomal protein S12 (Mrps12), apolipoprotein L 11b (Apol11b), and budding uninhibited by benzimidazoles 1 mitotic checkpoint serine/threonine kinase B (Bub1b; Supplementary Table 26). Interestingly, one gene is regulated in opposite directions in brain (up) relative to blood (down) collagen, type VI, alpha 4 (Col6a4) in marking DEGs among *3xTg-AD* vs. wild-type mice (Supplementary Table 26).

In CBD-treated *3xTg-AD* animals for the brain (Figure 6B), none of the DEGs remained as marked in *3xTg-AD* relative to wild-type mice with the exception of a persistent upregulation of Usp18 regardless of CBD treatment in *3xTg-AD* mice (Supplementary Tables 25, 27). However, note that there were relevant gene homologs in CBD-treated animals such as opposing regulations of Nlrc5 and Gbp3 in CBD-treated *3xTg-AD* mice (Supplementary Table 27) vs. Nlrc4 and Gbp2b, respectively, in *3xTg-AD* vehicle vs. wild-type mice (Supplementary Table 25). Furthermore, homologs Ifi204 and CxCl13 (*3xTg-AD* vs. wild-type; Supplementary Table 25) and Ifi44/206/209/27l2a and Cxcl10 (CBD-treated vs. vehicle *3xTg-AD* mice; Supplementary Table 27) were commonly upregulated. Note that there is only one precise DEG match that is regulated in opposing directions among cross-sectional blood and brain compartments for CBD-treated vs. vehicle *3xTg-AD* mice as the lncRNA Gm49980 (Supplementary Table 28).

Relatively few DEGs were apparent for CBD-treated vs. vehicle wild-type mice as 7 downregulated [e.g., Cxcr5 and Midline 1 (Mid1)] and 10 upregulated [e.g., Nurim (Nrm), Lymphocyte transmembrane adaptor 1 (Lax1), SLAM family member 6 (Slamf6), Immunoglobulin kappa constant (Igkc) and Immunoglobulin kappa chain variable 12-41 (Igkv12-41)] genes while not overlapping with those of CBD-treated *3xTg-AD* animals (Figure 6C, Supplementary Table 29). Furthermore, overlapping DEGs (all upregulated) among the brain and blood compartments for the CBD-treated vs. vehicle wild-type animals are limited to three pseudogenes (Gm14165, Gm8730, and Gm96250; Supplementary Table 30).

For CBD-treated *3xTg-AD* vs. CBD-treated wild-type animals, 73 and 98 genes were upregulated and downregulated, respectively (Figure 6D, Supplementary Table 31). With relevance to notable genes marked in the brain for AD pathology and CBD treatment in *3xTg-AD* animals, Eif3j2, Eno1b, Apol11b, and Cxcl13 commonly mark both biological sample compartments with DNA nucleotidylexotransferase (Dntt) regulated in opposing directions in brain relative to blood (Supplementary Table 32).

### Pathways of AD onset influenced by CBD treatment: cross-sectional brain analyses

**Figure 7:**
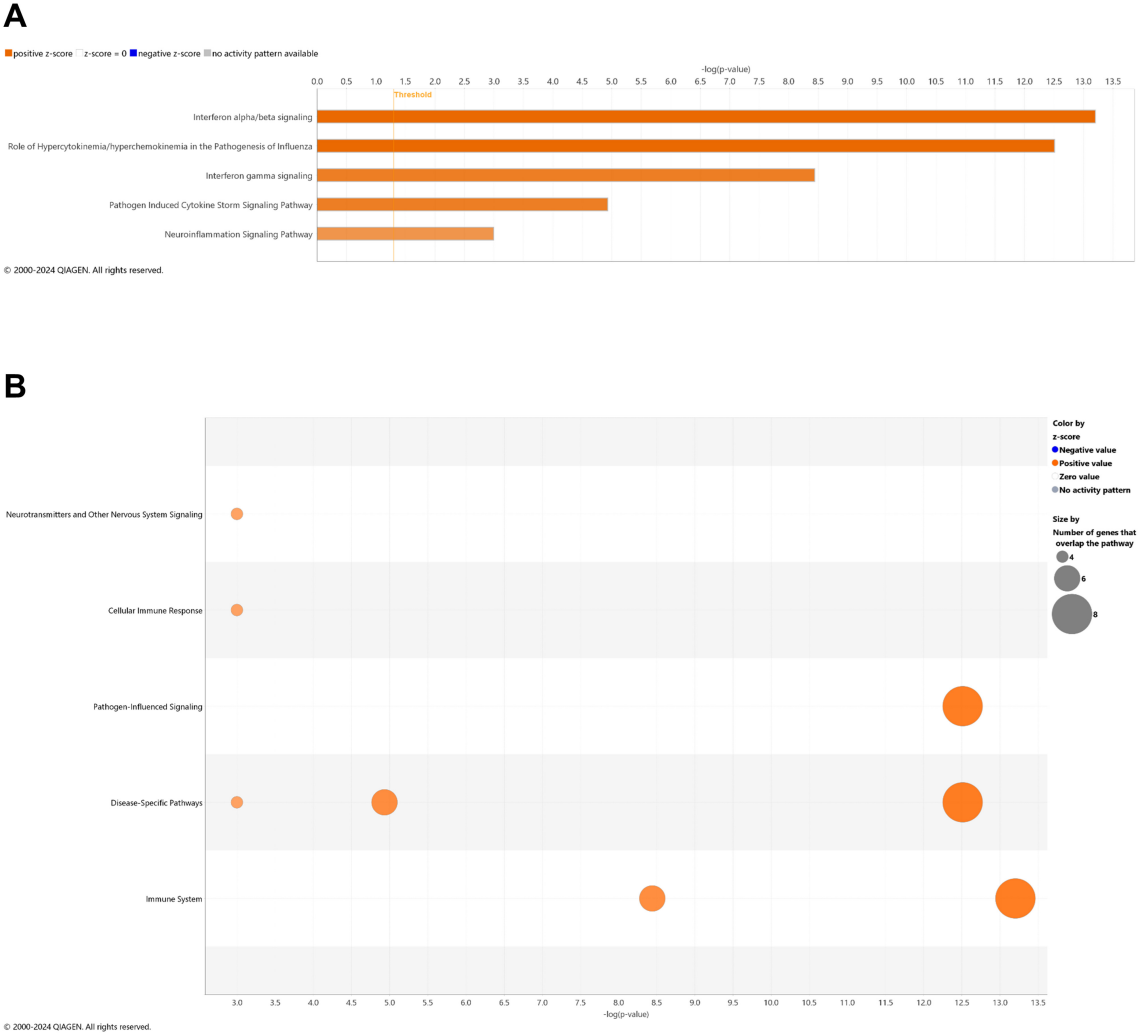
Cell signaling pathways and categories in whole brain in Alzheimer's disease animals treated with cannabidiol relative to vehicle controls. **(A)** Canonical pathways that were significantly more (orange, 5) or less (blue, 0) in 6.5 mo CBD-treated *3xTg-AD* vs. *3xTg-AD* vehicle mice. **(B)** Bubble plots of the number of genes that overlap with major pathway categories with size of bubble directly indicating the amount of overlap; more = orange and less = blue. The Ingenuity pathway analysis setting was set at a log2 fold change cutoff at 1.0 up and −1.0 down (*p*-value ≤ 0.05). The significance of canonical pathways was determined at a –log(*p*-value) greater >1.3 and absolute *z*-score of >2.0. Data were obtained from *n* = 5 male mice per group. Note that no significant pathways emerged in comparisons among brains of *3xTg-AD* vs. wild-type B6129 mice or CBD-treated wild-type B6129 vs. wild-type vehicle mice. This figure was generated through the use of QIAGEN IPA (QIAGEN Inc., https://digitalinsights.qiagen.com/IPA) (Krämer et al., 2014).

**Figure 8:**
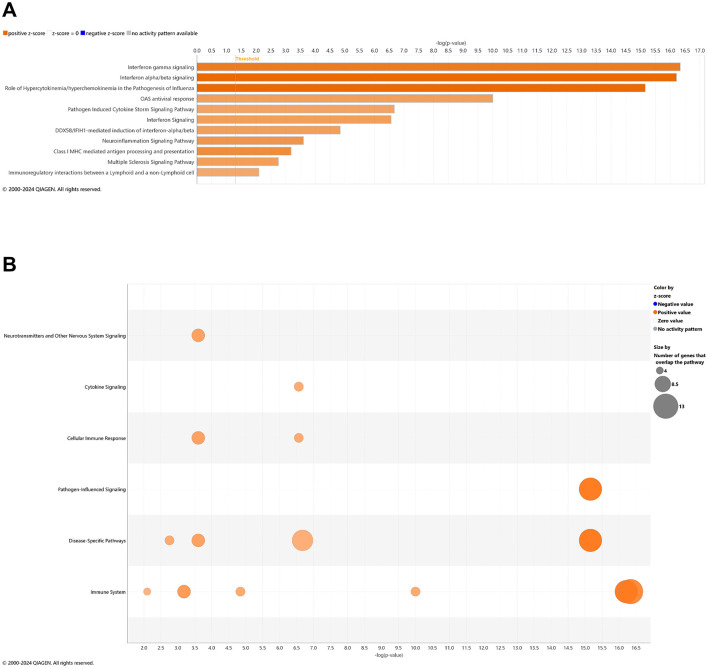
Cell signaling pathways and categories in whole brain in Alzheimer's disease animals treated with cannabidiol relative to vehicle controls. **(A)** Canonical pathways that were significantly more (orange, 11) or less (blue, 0) in 6.5 mo CBD-treated *3xTg-AD* vs. CBD-treated wild-type B6129 mice. **(B)** Bubble plots of the number of genes that overlap with major pathway categories with size of bubble directly indicating the amount of overlap; more = orange and less = blue. The Ingenuity pathway analysis setting was set at a log2 fold change cutoff at 1.0 up and −1.0 down (*p*-value ≤ 0.05). The significance of canonical pathways was determined at a –log(*p*-value) >1.3 and absolute *z*-score of >2.0. Data were obtained from *n* = 5 male mice per group. Note that no significant pathways emerged in comparisons among brains of *3xTg-AD* vs. wild-type B6129 mice or CBD-treated wild-type B6129 vs. wild-type vehicle mice. This figure was generated through the use of QIAGEN IPA (QIAGEN Inc., https://digitalinsights.qiagen.com/IPA) (Krämer et al., 2014).

With comparison of brain samples among all animal groups at 6.5 mo (8 wks vehicle or CBD treatment), there were no significant canonical pathways (cutoff: –log *p*-value > 1.3 & absolute *z*-score > 2.0) to distinguish *3xTg-AD* or CBD-treated wild-type mice from the wild-type vehicle group. Note that the relative scarcity in DEGs (≤10) among these groups for the brain may explain the absence of canonical pathways recognized among respective groups. Five upregulated pathways of the CBD-treated *3xTg-AD* vs. *3xTg-AD* vehicle group included (from greatest to least) interferon alpha/beta signaling, role of hypercytokinemia/hyperchemokinemia in the pathogenesis of influenza, interferon gamma signaling, pathogen induced cytokine storm signaling, and neuroinflammation signaling (Figure 7A). Gene overlap was most prominent for upregulation of overall pathogen-influenced signaling, disease-specific pathways, and the immune system (Figure 7B). For comparisons of CBD-treated *3xTg-AD* vs. CBD-treated wild-type, additional upregulated pathways included OAS antiviral response, class I MHC-mediated antigen processing and presentation, multiple sclerosis signaling, and immunoregulatory interactions between a lymphoid and a non-lymphoid cell (Figure 8A) with gene overlap patterns similar to CBD-treated *3xTg-AD* vs. vehicle (Figures 7B, 8B).

### Metabolites of AD onset influenced by CBD treatment: cross-sectional brain analyses

With knowing the general role of metabolomics, and particularly altered profiles of lipids (He et al., 2025) and immune markers (Ahmad et al., 2024) during Alzheimer's disease pathogenesis, we also sought to compare all study groups at 6.5 mo of age following 8 wks of vehicle treatment or CBD in brain samples. Using an untargeted screen, in positive mode, there were 73 significant metabolites whereas in negative mode, there were 39 metabolites. In total, there were 112 metabolites that marked *3xTg-AD* vs. wild-type vehicle mice (Supplementary Tables 33, 34).

Relative to *3xTg-AD* vehicle, all metabolites in CBD-treated *3xTg-AD* animals were not at a detectable level to show significance except for phosphate, which was increased in *3xTg-AD* vs. wild-type vehicle but decreased in CBD-treated *3xTg-AD* animals vs. vehicle (Supplementary Table 34). In wild-type animals, notable metabolites such as androstane, glutamate, palmitoyl ethanolamide, and malic acid were less in CBD-treated vs. vehicle animals, whereas 2-methylserine was higher. Furthermore, CBD-treated *3xTg-AD* animals show higher 13,16,19-docosatrienoicacid, glycero-3-phosphoethanolamine, N-dodecanoylsphinganine, 11-eicosenoic acid, 11,14,17-eicosatrienoic acid, 13,16,19-docosatrienoic acid, 2-hydroxyglutarate, 3-methylindole, 3b-hydroxy-5-cholenoic acid, oleic acid, carnitine, cis-5-tetradecenoylcarnitine, fructose 6-phosphate, glutaconate, glycerophosphoglycerol, eicosadienoic acid, leucine, pantetheine, phenylalanine, sn-glycero-3-phosphoethanolamine, stearic acid, tryptophan, and xanthosine levels but lesser 1-linoleoylglycerophosphocholine, 16-HETE, 2-arachidonyl-sn-glycero-3-phosphoethanolamine, 9-nitrooleate, arachidonic acid methyl ester, N-arachidonoyl taurine, lysophosphatidylethanolamine (22:6/0:0), 11(Guard et al., 2022)-EET, 2-aminomuconate, and guanosine relative to CBD-treated wild-type animals (Supplementary Tables 33, 34).

### Behavioral analyses

**Figure 9:**
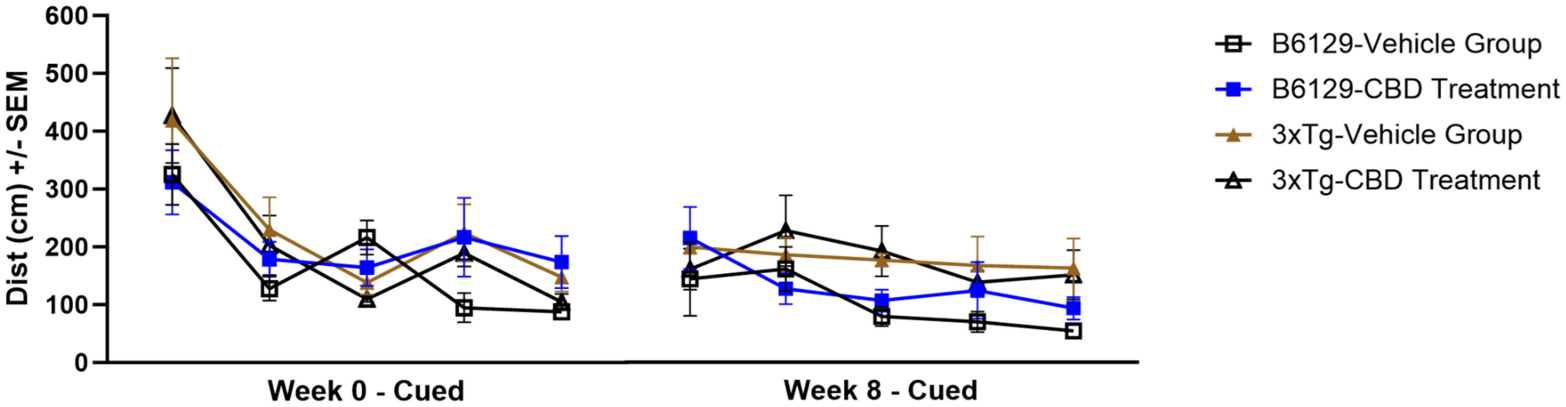
Cued characteristics of the water maze among wild-type and Alzheimer's disease animals with and without cannabidiol treatment. Animals were cued on Day 1 of each time period (five trials) as 0 wks (4.5 mo old) and 8 wks (6.5 mo) throughout the treatment period. A lower distance as marked on the y-axis implies greater learning ability. At week 8, the *3xTg-AD* mice (6.5 mo old) swam ≈2.9 times the distance relative to age-matched, wild-type B6129 mice. Both *3xTg-AD* and wild-type B6129 mice groups traveled less for total distance at wk 8 vs. wk 0, but the wild-type group showed a ≈40% reduction in travel relative to the *3xTg-AD* group (≈22%). The effects of cannabidiol (CBD) at wk 8 appear negligible among respective *3xTg-AD* and wild-type animal groups. Data were obtained from *n* = 3–5 mice per group. See Supplementary Figures 1, 2 for average distance data comparisons and individual block comparisons, respectively, among study groups.

**Figure 10:**
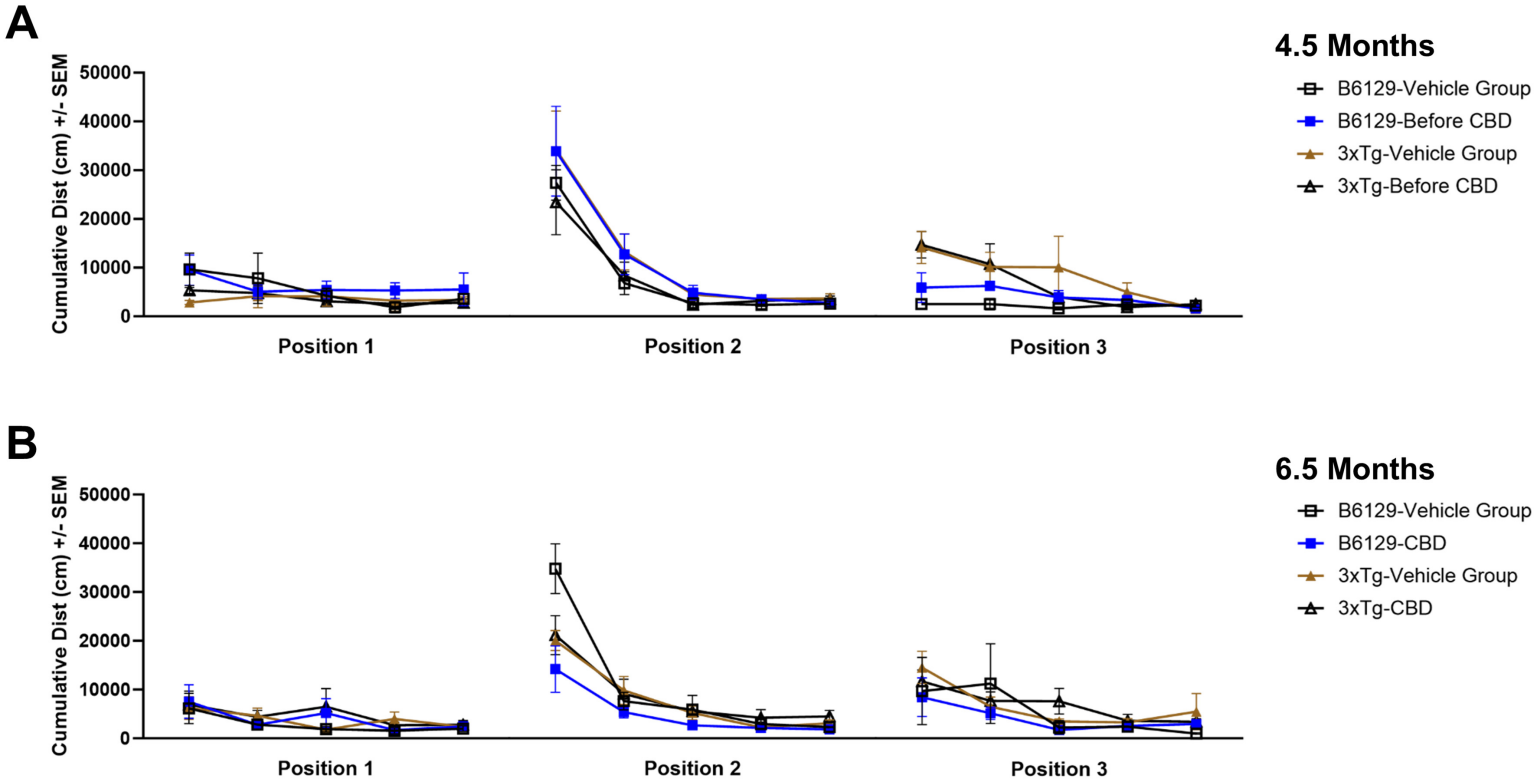
Distance traveled throughout three positions of the water maze among wild-type and Alzheimer's disease animals with and without cannabidiol treatment. **(A)** Cumulative distances traveled among groups at wk 0 (4.5 mo old) at Positions 1, 2, and 3. **(B)** As in A, at wk 8 (6.5 mo old). Note no significant differences overall among groups. Data were obtained from *n* = 3–5 mice per group. See Supplementary Figures 3–6 for additional details regarding individual spatial characteristics (number of target entries, percent time in target zone, and cumulative distance to target) among study groups.

Since AD is a cognitive disorder (Baerresen et al., 2015), we sought to assess learning, spatial, exploratory, and organizational behavior (Hartman et al., 2001; Rudobeck et al., 2017) longitudinally in the same wild-type and *3xTg-AD* animals used for the molecular analyses in the absence and presence of CBD treatment. In the MWM (cued visible platform phase), both *3xTg-AD* and wild-type mice groups exhibited reduced total distance traveled at 6.5 vs. 4.5 mo, but the reduction in the wild-type group was approximately double of that for the *3xTg-AD* mice (≈40% vs. ≈22%, respectively; Figure 9). *3xTg-AD* mice at AD onset (6.5 mo) swam ≈2.9 times greater distance relative to the age-matched, wild-type mice. The effects of CBD at 6.5 mo (following 8 wks of treatment) were negligible among respective *3xTg-AD* and wild-type animal groups (Figure 9). In addition, for the spatial submerged platform phase, note that there was a trend (P>0.05) of an increased average distance traveled by *3xTg-AD* vs. wild-type (Supplementary Figure 1). Overall, all study groups performed better at wk 8 (6.5 mo) relative to the starting point at wk 0 (4.5 mo) as indicated by a reduced travel distance (Supplementary Figure 2). There were no significant group differences among the cumulative distances to the target in the Spatial learning phase (Figure 10). During the Probe trials at wk 0, none of the groups spend more than 25% of the trial searching the correct target quadrant, suggesting a lack of memory for the escape platform's location. However, 8 wks later, the wild-type mice spent >25% of the trial searching the correct target quadrant (suggesting a memory for the escape platform's location; *P* < 0.05), whereas the *3xTg-AD* mice still did not (Supplementary Figure 3D). CBD treatment increased the average number of target zone entries by ≈22% in wild-type mice and ≈3% in *3xTg-AD* mice (Supplementary Figure 4). Furthermore, CBD increased the average time spent in the target zone by ≈20% in wild-type mice and ≈8% in *3xTg-AD* mice (Supplementary Figure 5). Finally, CBD treatment also decreased the average cumulative distance to target (i.e., improved performance) by ≈7% in wild-type mice and ≈4% in *3xTg-AD* mice (Supplementary Figure 6).

**Figure 11:**
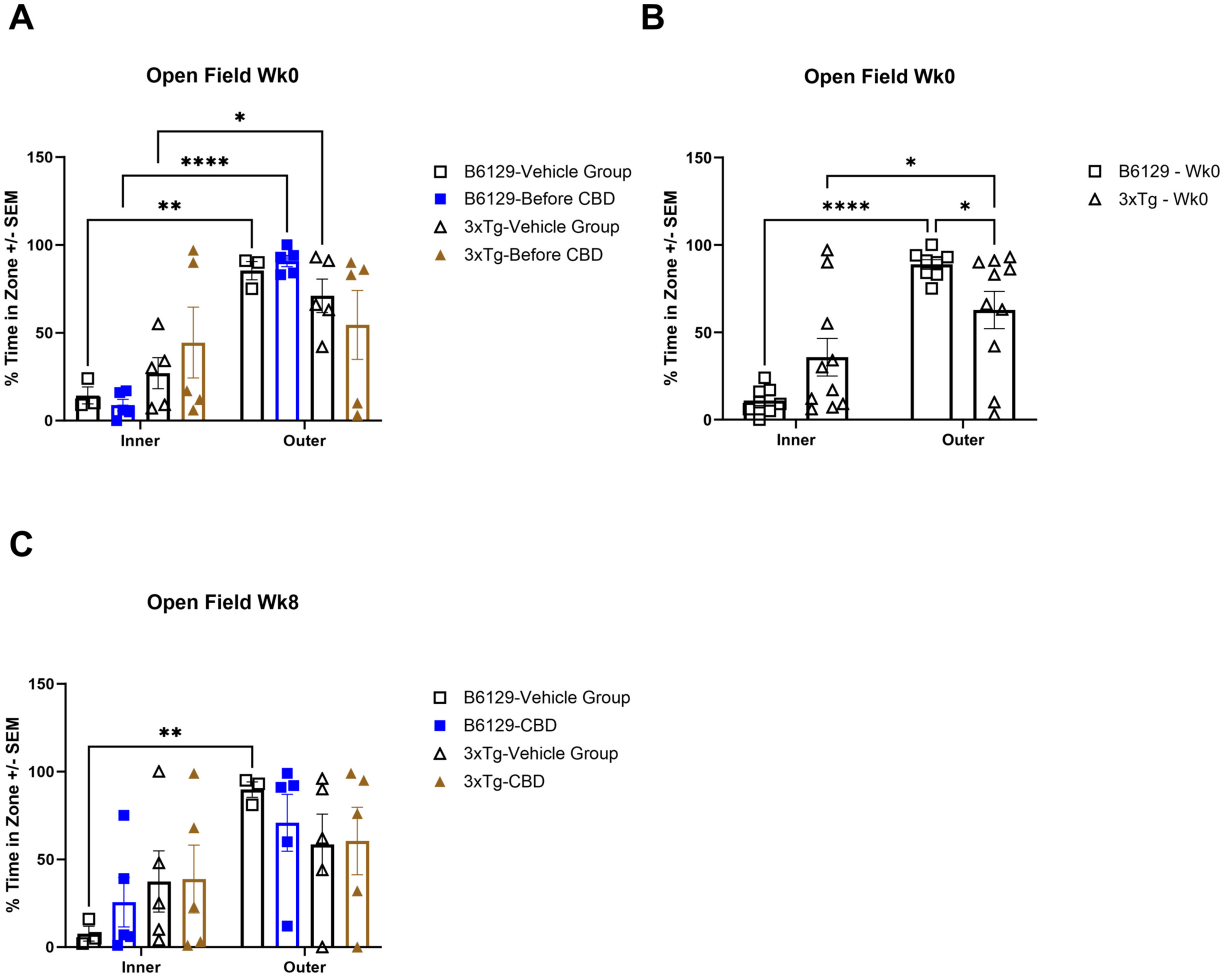
Time spent in the open field test among wild-type and Alzheimer's disease animals with and without cannabidiol treatment. **(A)** Travel times among inner (open central) and outer (periphery, edges, and corners) zones among all respective study groups at wk 0 (no exposure to CBD yet). Note that outer vs. inner zone times were generally higher in the wild-type but not *3xTg-AD* mice. **(B)** As in **(A)**, with data combined for wild-type B6129 and *3xTg-AD* mice, respectively, at wk 0. Although the time in the outer zone was higher relative to the inner zone in both groups, note the significantly less time in the outer zone for the *3xTg-AD* vs. wild-type B6129 mice. **(C)** As in **(A)**, with comparisons at wk 8. Note that the only group with a significantly higher outer vs. inner time zone value was wild-type B6129 vehicle. Data were obtained from *n* = 3–5 mice per group (*n* = 8–10 in combined CBD and vehicle comparisons); *P < 0.05, **P < 0.01, and ****P < 0.0001.

**Figure 12:**
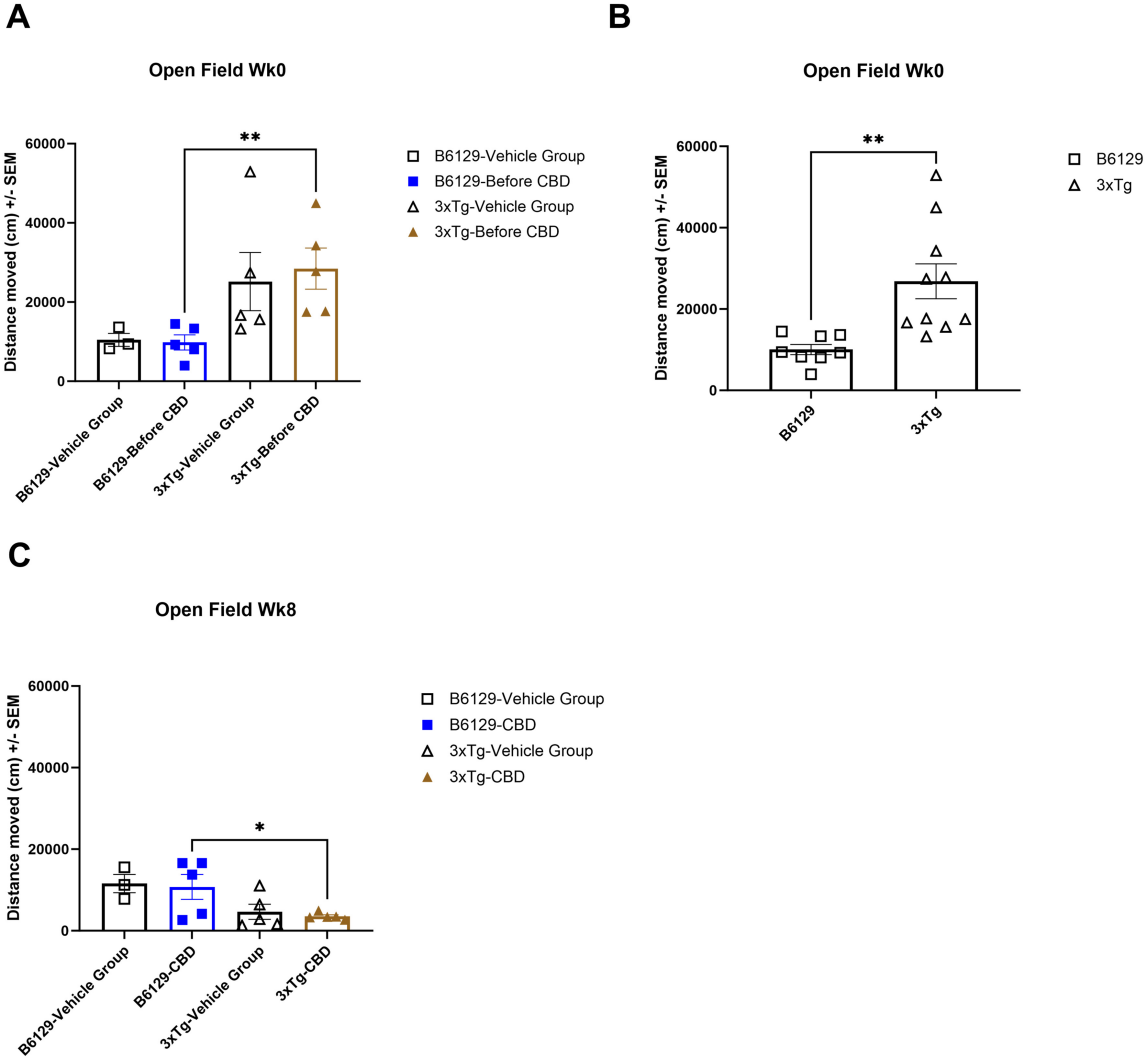
Average distance traveled in the open field test among wild-type and Alzheimer's disease animals with and without cannabidiol treatment. **(A)** The average distance traveled among all respective study groups at wk 0 (no exposure to CBD yet). The *3xTg-AD* animals indicate a trend of more distance traveled relative to wild-type B6129 animals. **(B)** As in A, with data combined for wild-type B6129 and *3xTg-AD* mice, respectively, at wk 0. Note significantly more distance traveled in the *3xTg-AD* group relative to wild-type B6129. **(C)** As in A, with comparisons at wk 8 following CBD vs. vehicle treatment. The distances traveled were generally less in the *3xTg-AD* vs. wild-type B6129 group with negligible effects of CBD. Data were obtained from *n* = 3–5 mice per group (*n* = 8–10 in combined CBD and vehicle comparisons); *P < 0.05, **P < 0.01. See Supplementary Figure 7 for the percentage of time spent in the center, parameter, and corners at wks 0 and 8 among study groups.

For the OFT test, all mice on average spent more time in the periphery (edges and corners) relative to the open central zone, but the *3xTg-AD* mice spent significantly more time in the open central zone, suggesting a lack of anxiety about potentially risky behavior (Figures 11A, B). By 6.5 mo, only the wild-type vehicle mice spent significantly more time in the periphery (Figure 11C). There were no significant differences among the percentage of time spent in the center, parameter, and corners at 4.5 and 6.5 mo among study groups (Supplementary Figure 7). During 4.5 mo, note that the *3xTg-AD* mice were hyperactive, traveling significantly more distance relative to wild-type animals (Figures 12A, B). By 6.5 mo, however, the *3xTg-AD* mice were hypoactive, traveling less than the wild-type B6129 group. The effects of CBD on both groups were negligible (Figure 12C).

For the NBT test for cognitive function, there was a trend for lower nesting scores among *3xTg-AD* relative to wild-type mice at both 4.5 and 6.5 mo (Supplementary Figure 8). With neuropathological onset at 6.5 mo, average nest scores among *3xTg-AD* animals were relatively flat across three nights of examination while not exceeding a score of 4 (≈3.5–3.8). Apparent effects of CBD were mild in 6.5 mo *3xTg-AD* animals but with correspondence to average scores of 4 on the second and third nights relative to less < 4 in the vehicle *3xTg-AD* group. In contrast, nesting scores among 6.5 mo wild-type animals progressively increased over the three-night period from ≈3.5–4.5, with similar (or lesser) scores during CBD treatment relative to vehicle across all three nights.

## Discussion

With priorities for clarifying molecular pathogenesis from mild cognitive impairment to the onset of Alzheimer's disease pathology (Frech et al., 2024), and potential mechanisms of therapeutic cannabidiol intervention (Cummings et al., 2025), we conducted a thorough blood transcriptomic analysis of early-stage pathogenesis of Alzheimer's disease using the *3xTg-AD* animal model. Furthermore, additional cross-sectional analyses were performed on paired brain and blood samples during Alzheimer's disease onset relative to wild-type controls to survey potential agreement among prominent biomarkers of the blood circulation and central nervous system. Although a limitation of the study, a focus on only male animals in the current study is consistent with the bulk of differences noted in the molecular profile (Chum et al., 2022, 2024) and structure (Jullienne et al., 2022) of cerebral vessels relative to females in aging *3xTg-AD* mice. In brief, over 900 DEGs marked AD onset in *3xTg-AD* mice relative to the timepoint of cognitive impairment. Approximately 240 of these genes were identified as AD-associated markers pertinent to human subjects, whereby at least 75% were either removed as statistically significant or reversed in the direction of expression as a result of dietary cannabidiol treatment. Altogether, these data provide insight into the early-stage molecular pathogenesis of AD, susceptible to disruption by a chronic (≈2 month) cannabidiol intervention. Given the extensive datasets, selected biomarkers are further discussed below concerning their biological mechanisms and clinical implications.

### Genes of Alzheimer's disease onset: sensitivity to cannabidiol treatment

Relative to age-matched wild-type mice, ApoE was downregulated in the blood of *3xTg-AD* mice during onset of AD, a downregulated DEG that disappeared following CBD treatment. This finding is significant as ApoE deficiency promotes atherosclerosis in mice (Pendse et al., 2009) as most commonly observed in human subjects with the presence of the APOE4 gene and increased risk for developing AD pathology (Sun et al., 2023b). Recent *in silico* (Choi et al., 2023) and cholesterol transport (Allende et al., 2024) analyses involving aberrant ApoE function have been suggestive of CBD's utility in this regard. There were also genes significant upon AD onset in *3xTg-AD* mice that were reversed in the direction of expression following CBD treatment. Ramp3, Sema4c, Rin1, Acvr1, Iqck, Tagln3, Scg5, Cacna2d4, and Peg3 were downregulated with AD onset and were reversed to upregulated in expression following CBD treatment. Mgat3, Tmem63c, Kcnk2, Prkar1b, Smad9, and Rgs7bp were upregulated with AD onset and were reversed to downregulated in expression following CBD treatment. Other AD genes that were obviated as DEGs in response to CBD at AD onset included those that were downregulated (e.g., Ramp3, Tamalin, Sema4c, the lncRNA Map2k3os, Rin1, F12, Acvr1, Iqck, Tagln3, Scg5, Wfs1, Cacna2d4, Ncr1, and Esr1) and upregulated (e.g., Ndufa7, Cox7a2, Acacb, Gls2, Bmp4, Mgat3, Ppp1r3c, Vgf, Gpr6, Hapln2, Oprd1, Ntsr1, Lrfn5, Nap1l2, Pcsk2, Cckbr, Tmem63c, Prkar1b, the miRNA Mir144, Mei1, Tacr3, Lin7a, Gria4, Npsr1, Scara3, Ankrd36, Insm1, Snap91, St8sia3, Pcdh9, Rgs7, Chrna2, Pld6, Adamts13, Kcnk2, Slc17a7, Prok2, and Ncan). With organization across synaptic plasticity and development; neurovascular interactions; ion channels, receptors, and transporters; mitochondrial genes; inflammation and oxidative stress; and lipid and carbohydrate metabolism, these particular genes are emphasized for further discussion below.

### Synaptic development and plasticity

In the current study, we found that numerous genes linked to AD pathology are involved in neuronal network development and remodeling with development and aging (Kalra et al., 2025). Genetic interactions among RAMP3 and SEMA3A are notable for human subjects with AD (Wang et al., 2021), whereby Ramp3 mechanistically acts as an amylin receptor and regulates clearance of amyloid from the brain to the blood as demonstrated in the Tg2576 mouse model (Mohamed et al., 2017). Note that Sema4c is also expressed across human brain regions as the entorhinal cortex, hippocampus, middle temporal gyrus, posterior cingulate cortex, superior frontal gyrus, and visual cortex in AD human subjects (Puthiyedth et al., 2016). As both semaphorins (Sema3a and Sema4c) are known to regulate nervous system development and plasticity (Carulli et al., 2021), it is possible that the murine version of Ramp3 to Sema3a interaction noted with AD pathology in humans (Wang et al., 2021) more precisely involves Sema4c (and not Sema3a) instead. Although CBD treatment has not been identified for modulation of Ramp3 and the semaphorin genes in the past per AD pathology, stimulation of some cannabinoid receptors (e.g., CB1R) is known to increase Ramp3 expression (Glenn et al., 2024). Rin1 regulates postsynaptic neuronal plasticity, whereby its deficiency leads to enhanced amygdala long-term potential and associated aversive memory (Dhaka et al., 2003). Rin1 has also been identified as a hub gene in late-onset AD patients but in non-carriers of APOE4 (Jiang et al., 2016). Although interaction of CBD with Rin1 *per se* has not been established in prior studies, its ability to bolster Rin1 expression is consistent with overall effects as a reduction in learned fear and aversive memory (Bitencourt and Takahashi, 2018). Acvr1 is a type I receptor for bone morphogenetic protein while associated with hippocampal volume (Horgusluoglu-Moloch et al., 2019). CBD is an inhibitor of the expression of the inhibitor of DNA binding 1 (Id1) gene as a downstream target of Acvr1 (Messinger et al., 2023). While a binding partner for EF-hand proteins such as calmodulin, Iqck is a genome-wide risk signal for AD (Kunkle et al., 2019) and also associated with obesity (Hinney et al., 2014). Tagln3 assists with actin filament organization and is downregulated in patients with sporadic AD while a target of APOE4 (Arnaud et al., 2022). Prkar1b is a regulatory subunit of cyclic AMP-dependent protein kinase (PKA) and is associated with neurodevelopmental disorders and neurodegeneration in general (Benjamin-Zukerman et al., 2024) including distinctions among symptomatic and asymptomatic forms of AD (Tandon et al., 2023). Ncan is a chrondroitin sulfate proteoglycan involved in synaptic plasticity while associated with amyloid levels (Mravinacova et al., 2024). Vgf is inducible by the presence of nerve growth factor and is associated with the onset and progression of AD (Lu et al., 2025; Beckmann et al., 2020; Busse et al., 2015). Lrfn5 mediates cell adhesion for synaptic plasticity and coincides with AD and major depressive disorder (Nho et al., 2015). Pcdh9 is protocadherin involved in cell–cell adhesion in the presence of Ca^2+^ while associated with neurofibrillary tangles and phosphorylated tau (Ghose et al., 2024). Snap91 is a synaptosome-associated protein involved in clathrin and phosphatidylinositol binding activity while having been identified as a hub gene for AD (Hu et al., 2020). As an AD-selective DEG eliminated by CBD treatment, Cplx2 (Nie et al., 2021) also modulates neuronal control of memory in patients with schizophrenia (Hass et al., 2015) and during frontotemporal dementia (FTD) pathogenesis (Ramos-Miguel et al., 2018). Furthermore, Foxp2 [fundamental to nervous system evolution and development (Usui et al., 2014)] was significantly up- and downregulated in longitudinal analyses of *3xTg-AD* and wild-type mice, respectively; CBD treatment removed Foxp2 as a DEG for both groups. In addition to AD (Oswald et al., 2017), note that Foxp2 is also integral to the development of a host of neurodegenerative diseases including FTD (Padovani et al., 2010).

For regulation of neuronal growth, Bmp4 is a ligand of bone morphogenetic receptors (can activate Acvr1), whereby its increased expression correlates to decreased hippocampal cell proliferation during AD (Li et al., 2008) and white matter destruction following chronic hypoperfusion of the brain (Uemura et al., 2018). Past evidence has demonstrated that CBD can downregulate Bmp4 expression (Gurgul et al., 2024). In addition to Nap1l5 (Wang et al., 2022b), histone chaperone Nap1l2 regulates neuronal proliferation by interacting with chromatin while associated with AD among other neurodegenerative diseases (Haenig et al., 2020). Mei1 is involved in meiosis I for germ cell development with potential association with AD (Li and De Muynck, 2021). Finally, Peg3 of the Kruppel C2H2-type zinc finger protein family is also involved with regulating neuronal growth and development, whereby its deficiency (as demonstrated in the current study with *3xTg-AD* animals) leads to apoptosis (Broad et al., 2009). Alterations in miRNAs that primarily target mRNAs for cellular growth proliferation and development were also a molecular characteristic of cerebral vessels of aging *3xTg-AD* mice (Chum et al., 2022, 2024).

### Neurovascular interactions

At least from a mechanistic pathogenesis perspective, it is clear that AD has now been recognized as a neurovascular disorder as well (Chum et al., 2024; Zhu et al., 2022; Santisteban et al., 2023), whereby cerebrovascular growth, permeability, and resistance/tone operate or disintegrate together in concert toward brain health or dementia, respectively. Ankrd36 is ankyrin repeat domain protein that regulates blood pressure by interaction with the transcription factor YY1 and thereby influencing epithelial Na^+^ channel (ENaC) expression (Yan et al., 2021). Ankrd36 expression can be correlated with Mini-Mental State Examination (MMSE) and Medial Temporal Atrophy (MTA) scores, particularly in Vietnamese AD patients (Cao et al., 2023). Adamts13 is metalloproteinase that regulates thrombosis by cleaving von Willebrand Factor (VWF) while comprising a vascular disease axis component of AD (Hanas et al., 2021). Hapln2 supports formation of the blood–nerve barrier but elevated levels may also contribute to neurodegeneration during AD (Tandon et al., 2023) or Parkinson's disease (Wang et al., 2016). Smad9 expression was completely reversed in the direction of the expression from *3xTg-AD* vehicle (upregulated by log2 fold change = 3) to CBD-treated *3xTg-AD* mice (downregulated by log2 fold change = 3). Furthermore, signaling pathways at AD onset that were primarily addressed with CBD treatment (e.g., embryonic stem cell pluripotency, adipogenesis, proliferation and myelination, and molecular mechanisms of cancer) centered on Smad9. As a target of miR-132 and miR-27a, Smad9 was also highlighted as a strong indicator of AD onset in our prior studies that had examined the molecular pathogenesis of cerebral vessels of aging *3xTg-AD* animals (Chum et al., 2022, 2024). Although CBD treatment did not eliminate Prelp (Li and De Muynck, 2021) in *3xTg-AD* animals, the extent of upregulation was decreased (log2 fold change in vehicle = 5.1 vs. CBD-treated = 2.7). Prelp is selectively expressed in vascular smooth muscle cells and pericytes and regulates cellular adhesion of integrity of the blood brain barrier (Davaapil et al., 2023). Although not as associated with AD *per se*, CBD also reversed expression of the angiogenic gene Angptl6 (Carbone et al., 2018) from down- to upregulated relative to *3xTg-AD* vehicle mice.

### Ion channels, receptors, and transporters

As with all chronic co-morbidities that ultimately develop from vascular aging and compromised perfusion of the central nervous system and periphery, AD is also a “channelopathy” in large part (Behringer, 2023). Cacna2d4 is an L-type voltage-dependent Ca^2+^ channel auxiliary subunit (α2/δ4) while a marker of AD and hyperhomocysteinemia (Wang et al., 2023). Tmem63c is an osmo-sensitive Ca^2+^-permeant cation channel and an early-stage biomarker of AD (Yaghoobi and Malekpour, 2024). Kcnk2 is a two-pore domain background K^+^ channel that can also mark brain atrophy per cognitive impairment and AD (Li and De Muynck, 2021; Le Guen et al., 2019). Gpr6 is an adenylate cyclase-activating GPCR (G_s_) with CBD as an inverse agonist and has been proposed as a therapeutic target of AD and Parkinson's disease (Laun et al., 2019; Benoit et al., 2013). Oprd1 is a delta-type opioid GPCR (G_i_/G_o_) and is associated with slowing of oscillatory brain activity per AD (Macedo et al., 2021). Ntsr1 is a promiscuous neurotensin GPCR (G_s_, G_q/11_, G_i/o_, and G_12/13_) with altered expression in concert with the appetite stimulant ghrelin during AD (Gahete et al., 2010). Cckbr is a GPCR (G_q_ and G_i_) for gastrin and cholecystokinin while integrated with the activities of several other receptors such as the AMPA ionotropic and metabotropic glutamate receptors and CB1Rs for governing excitatory long-term potentiation (Asim et al., 2024). Tacr3 is a GPCR (G_α*q*_) for neurokinin B that governs cholinergic activity underlying learning and memory (de Souza Silva et al., 2013). Gria4 is an AMPA ionotropic glutamate receptor and may contribute to excitotoxicity during AD (Jacob et al., 2007; Bereczki et al., 2018). As a negative regulator of Gria4, miR-27a coincidentally decreases in expression in cerebral vessels of overall AD vs. pre-AD pathology in *3xTg-AD* mice as well (Chum et al., 2022, 2024). Npsr1 is a neuropeptide GPCR (G_q_ and G_s_) of the vasopressin/oxytocin subfamily and is a target of early-stage AD (Gazestani et al., 2023; Wallace et al., 2024). As upregulated during AD onset in the absence of CBD treatment, both Rgs7 and its binding protein Rgs7bp play a role in opioid, dopamine, and adrenergic GPCRs as the G_α*i*/*o*_-type (Masuho et al., 2013). Rgs7 in particular has been associated with aberrant copper metabolism during AD (Squitti et al., 2023). Chrna2 is a nicotinic cholinergic receptor subunit and is a clinical target for AD (Xu et al., 2021), with specific polymorphisms noted for Chinese (Ding et al., 2023) and Korean (Kim et al., 2024) populations. As with α7-containing nicotinic cholinergic receptors, CBD may suppress Chrna2 expression or activity (Demontis et al., 2019). As a hub gene of early-stage AD (Wang et al., 2024c), Slc17a7 is a multifunctional transporter of glutamate and several ionic species as Na^+^, K^+^, H^+^, Cl^−^, and ${\text{PO}}_{4}^{3-}$ (Aihara et al., 2000). Other notable receptor and ion channel DEGs addressed by CBD treatment in *3xTg-AD* animals included regulatory proteins Necab2 [for adenosine A_2A_ and metabotropic glutamate type 5 receptors (Xie et al., 2022)] and Gprasp2 [for M1 muscarinic acetylcholine and calcitonin receptors (Edfawy et al., 2019)]; the H^+^-gated, Na^+^ permeant ion channel Asic4 (Lin et al., 2015); and the voltage-gated K^+^ channels Kcng2 (Guo et al., 2023) and Kcnq4 (Lee et al., 2021).

For scaffolding of plasma membrane proteins, Tamalin (or GRASP) is a molecular scaffold for group 1 metabotropic glutamate receptors and the guanine nucleotide exchange factor cohesins (Kitano et al., 2002). Tamalin is also required for the survival of neurons and oligodendrocytes (Seo et al., 2022). Lin7a is a synaptic protein involved in the distribution of receptors and ion channels in the plasma membrane, whereby its upregulation and downregulation in expression indicate early- and late-stage AD, respectively, as paired with progressive Braak stages (Hondius et al., 2016).

### Mitochondrial genes

In response to the increasing prevalence of AD (Cummings et al., 2025), mitochondrial biology and medicine is also developing rapidly for contemporary biomedical research (D'Alessandro et al., 2025; Mosharov et al., 2025). As an upregulated hub gene in AD patients (Liu et al., 2020) and the current study using *3xTg-AD* animals, Acacb catalyzes carboxylation of acetyl-CoA to malonyl-CoA as the rate-limiting step in fatty acid synthesis. Ndufa7 is the NADH; ubiquinone oxidoreductase subunit A7 in complex I of the mitochondrial electron transport chain (ETC), whereby its dysregulated expression may underlie metabolic disorders during AD (Haytural et al., 2021). Cox7a2 is cytochrome c oxidase subunit 7A2 in complex IV of the ETC and catalyzes electron transfer from reduced cytochrome c to oxygen, whereby its altered expression can correlate amyloid plaque burden per AD (Ji et al., 2022; Bi et al., 2018). Gls2 is a mitochondrial glutaminase enzyme that decomposes glutamine into glutamate and ammonia while potentially contributing to ferroptosis during AD (Wang et al., 2022a). Pld6 (or mitoPLD) is a mitochondrial cardiolipin hydrolase and a component of the dysregulated lipidome with AD (Jin et al., 2006; Chan et al., 2012). Another notable mitochondrial DEG addressed by CBD treatment in *3xTg-AD* animals included Timm8b as a translocase of the inner mitochondrial membrane (Wang et al., 2024a).

### Inflammation and oxidative stress

Inflammation and oxidative stress have been well-established as major pathological contributors of AD (Amelimojarad et al., 2024; Bhandari et al., 2024). The CBD upregulation of Tagln3 likely results in decreased inflammation by inhibiting nuclear factor kappa B (NF-κB) activation (Arnaud et al., 2022; Atalay Ekiner et al., 2022). Scg5 is a chaperone protein (and copper metabolism indicator) that prevents aggregation of other secreted proteins; expression decreases with severity of AD (Zhuang et al., 2024) or cerebral amyloid angiopathy (Vervuurt et al., 2024). Aberrant Wfs1 expression is an indicator of endoplasmic reticulum stress while associated with tau pathology (Chen et al., 2022b) and may be addressed by stimulation of CB1R (McDew-White et al., 2023). Ncr1 is an immune receptor that distinguishes cognitive non-resilience vs. resilience among APOE4 carriers, prone to development of AD (Walker et al., 2024). Esr1 is estrogen receptor 1 involved at the intersection of oxidative stress and AD (Zhou et al., 2024), while underlying agitation as a behavioral phenotype in particular (Fisher et al., 2024). As demonstrated in the current study for AD onset, CBD may prevent Esr1 downregulation in response to unpredictable chronic mild stress (Bright and Akirav, 2025). Scara3 is a macrophage scavenger receptor induced by oxidative stress while overlapping in prominent expression among AD and gastrointestinal disorders (e.g., gastroesophageal reflux disease; Adewuyi et al., 2022). Finally, F12 is coagulation factor XII that bridges circulating amyloid with inflammation via kallikrein-mediated cleavage of kininogen to produce bradykinin (Zamolodchikov et al., 2015).

### Lipid and carbohydrate metabolism

Dysregulated transport and metabolism of lipids and carbohydrates involving conditions such as atherosclerosis and type II diabetes are commonly integrated with the development of AD (de Oliveira et al., 2024; Kale et al., 2024). Mgat3 is an enzyme (N-acetylglucosaminyltransferase III or GnT-III) that stimulates lipid droplet growth and is involved in amyloid phagocytosis and may be up- or downregulated among subpopulations of AD patients (Fiala et al., 2011). Although not detected as a DEG in the cross-sectional analyses among *3xTg-AD* vs. wild-type mice (only longitudinal among respective groups), the fatty acid synthase gene Fasn (Ates et al., 2020) was also removed in response to CBD treatment. Pcsk2 (or PC2) is a serine endopeptidase known for converting precursor prohormones and peptides to active hormones or neurotransmitters (e.g., α-melanocyte stimulating hormone, glucagon, and insulin), whereby its dysfunction may link diabetic pathology with AD (Barranco et al., 2021). Insm1 is a zinc finger DNA-binding protein normally involved in neurogenesis and neuroendocrine cell differentiation (Welcker et al., 2013). Prok2 is a crucial neuropeptide component of the circadian clock that may link insulin resistance, cardiovascular disease, and AD (Tian et al., 2022; Mortreux et al., 2019). St8sia3 is a sialyltransferase that catalyzes transfer of sialic acid among glycoproteins and glycolipids while implicated in glycan modifications with AD pathology (Zhang et al., 2024). Ppp1r3c is a protein phosphatase regulatory subunit that activates glycogen synthase while preventing glycogen breakdown, upregulated in response to stress sensed by norepinephrine release from the locus coeruleus (Privitera et al., 2024) and AD (Noh et al., 2014). As primarily identified and characterized for aberrant metabolism during cancer, the carbohydrate-binding protein Ppp1r3c may be sensitive to CBD treatment (Sun et al., 2023a). Finally, other longitudinal blood DEGs such as Mafa [insulin gene expression in pancreatic β cells (Kataoka et al., 2002)] and Mlxipl [regulates glycolysis and lipogenesis (Abdul-Wahed et al., 2017); involved in both AD and coronary artery disease (Loika et al., 2023)] were eliminated in response to CBD in *3xTg-AD* animals.

### Non-coding RNAs

Non-coding RNA biomarkers continue the promise of innovative diagnosis and therapy for chronic diseases such as AD while stable in the blood circulation (Tijsen et al., 2012). Non-coding RNAs in blood that consistently mark AD onset while sensitive to CBD include the miRNA MiR144 and the lncRNA Map2k3os. As regulated by the AP1 transcription factor sensitive to oxidative signaling, an increase Mir144 expression increases amyloid production by inhibiting expression of Adamt10 (Cheng et al., 2013). Increased expression of Map2k3os coincides with the development of tau pathology and loss of serotonergic neuronal loss (Kolling et al., 2025). Other notable lncRNAs that coincided with AD onset in *3xTg-AD* animals and were addressed by CBD treatment include C920006O11Rik (Jia et al., 2020) and Lockd involved in the transcriptional regulation of the cyclin-dependent kinase inhibitor 1B (Cdkn1b) gene (Sung et al., 2018).

### Brain and blood transcriptome cross-sectional correlations

Cross-sectional transcriptomic correlations were also examined in blood and brain samples at AD onset in *3xTg-AD* mice relative to other age-matched study groups to ascertain relationships among biomarker DEGs present in the blood circulation and central nervous system. The AD-selective genes in brain samples during the onset of AD in *3xTg-AD* mice relative to age-matched wild-type animals primarily encompassed immunity as Rnase6 (Seto et al., 2022; Bolivar et al., 2024), Ms4a1 (Deming et al., 2019), Ccr1/6 (Halks-Miller et al., 2003; Subramanian et al., 2010; D'Angelo et al., 2020), Ifi204 (Green et al., 2022), Cxcl13 (Karaahmet et al., 2022), C5ar2 (Carvalho et al., 2022), Nlrc4 (Saadi et al., 2020), Serpina3n (Saroja et al., 2022), Il15 (Clark et al., 2021; Janelidze et al., 2018), and Osm (Yu et al., 2023; Whelan et al., 2019). In addition, there were oxidative stress genes indicated as Osgin1 as an apoptotic regulator via mitochondrial cytochrome c release (Kang et al., 2023) and Aqp6 as a transmembrane H_2_O channel also permeant to H_2_O_2_ (Amro et al., 2023). As downregulated in *3xTg-AD* animals, Pla2g4e is a cytosolic phospholipase known to confer cognitive resilience and resistance to development of AD (Perez-Gonzalez et al., 2020). As upregulated in *3xTg-AD* animals, Exoc3l2 is an endothelial factor involved in angiogenesis [upregulated by vascular endothelial growth factor (Vegfa)] and with gene mutations associated in AD pathology of human subjects (Wu et al., 2017; Seshadri et al., 2010). The Ca^2+^-activated K^+^ channel Kcnn4 (or K_Ca_3.1) was also upregulated as consistent with past observations of microglial activation and inflammation (Maezawa et al., 2012) and enhanced electrical dynamics of cerebrovascular endothelial cell function (Hakim and Behringer, 2020) per AD pathology. As a precise downregulated match among both blood and brain components of *3xTg-AD* relative to age-matched wild-type animals, the ubiquitin gene Ubc can mark AD in human subjects (Nguyen et al., 2024). However, note that other DEGs in blood appeared as homologs to the observed AD DEGs in the brains of *3xTg-AD* mice as Rnase1, Ms4a4b, Ccr5/9, Aqp11, Usp46, Cxcl14, Ms4a7, and Kcnn3. Remarkably, all AD-selective DEGs in the brain were addressed in the CBD-treated *3xTg-AD* group with the exception of the ubiquitin gene Usp18 (Widjaya et al., 2023; Xiang et al., 2018).

Other notable genes commonly regulated among the blood and brain compartments for *3xTg-AD* animals include Bub1b [cell division, sister chromatid to spindle microtubule attachment (Yang et al., 2017)], Eif3j2 [translation from mRNA to protein (Egorova et al., 2021)], H4c17 [chromatic packaging and function (Zhang et al., 2022)], Mrps12 [mitochondrial protein synthesis (Qiu et al., 2021)], Apol11b [or A330102K04Rik; lipid binding and Cl^−^ channel activity, very low- and high-density lipoprotein particles (Koury et al., 2007)], Gbp2b [host defense to bacterial infection (Yu et al., 2022)], and Eno1b as a pseudogene marker for α-enolase. Finally, one gene Col6a4 [collagen binding in the extracellular matrix (Andres-Benito et al., 2023)] was regulated in opposite directions in brain (up) relative to blood (down) in marking DEGs among *3xTg-AD* vs. wild-type mice.

### Brain metabolome

With blood samples dedicated to transcriptome analyses, we also sought to gather metabolite information from brain samples of all study groups, particularly with expectations in altered lipid (He et al., 2025) and immune (Ahmad et al., 2024) profiles per AD pathogenesis and CBD treatment. Notable fatty acid species in *3xTg-AD* mice include 4-(stearoylamino) butanoic acid (GABA derivative) and 11,14,17-eicosatrienoic acid [precursor of eicosanoid synthesis per inflammation; tracks AD pathogenesis (Nasaruddin et al., 2018)]. Cis-5-tetradecenoylcarnitine is an acylcarnitine species while a potential indicator of accelerated aging and co-morbidities such as atherosclerosis (Liu et al., 2018) and type II diabetes (Zhao et al., 2020). Known markers of AD include the bile acid lithocholic acid (Ehtezazi et al., 2023; Marksteiner et al., 2018), the ceramide n-dodecanoyl sphinganine (Filippov et al., 2012). Disruptions in brain metabolism during AD may also be indicated by elevated fructose 6-phosphate (Johnson et al., 2020, 2023), orthophosphate (Landfield et al., 1991), and the purine xanthosine (Ansoleaga et al., 2015; Kaddurah-Daouk et al., 2013). Alterations in the phospholipid species sn-glycero-3-phosphoethanolamine may also track AD (Ahsanul Haque et al., 2023). Finally, as a potential therapeutic for AD via general reduction of neurodegenerative oxidants, lipids, and inflammation (Baranger et al., 2019; Moiseenok and Kanunnikova, 2023), pantetheine (monomeric form of pantethine) is an analog of pantothenic acid while an intermediate in the catabolism of coenzyme A.

Downregulated metabolites in *3xTg-AD* animals vs. wild-type include a neuroprotective omega-3 fatty acid such as DHA (de Wilde et al., 2017) and its metabolite 14-hydroxy DHA (Do et al., 2023; Borkowski et al., 2021). As marked in *3xTg-AD* animals, the nitro fatty acid 10-nitrooleate was also less in AD subjects relative to cognitively healthy subjects (Morris et al., 2019). Downregulated phospholipid species in *3xTg-AD* animals include 1-(-docosahexaenoyl)-sn-glycero-3-phosphocholine, 1-Linoleoylglycerophosphocholine, lysophosphatidylethanolamine (Llano and Devanarayan, 2021), and a key component as choline (Yuan et al., 2022). Arachidonic acid metabolites (Shinto et al., 2022) include methyl arachidonate, 16-HETE, 18-HETE, 5-deoxy-J2-IsoP, 19-Hydroxy prostaglandin F2 (Ahmad et al., 2024), and epoxy-eicosatetraenoic acid (Fiala et al., 2025). Purines and respective metabolites include guanosine (Ugarte et al., 2015), inosine (Teixeira et al., 2020), and5′-S-methyl5′-thioinosine. Amino acid metabolites include 3-methylcrotonylglycine (leucine metabolite), 2-aminomuconate (tryptophan), 5-hydroxytryptophan (Kaddurah-Daouk et al., 2013; Tohgi et al., 1992), 4-hydroxyphenyllactic acid (tyrosine; Kaddurah-Daouk et al., 2013), reduced and oxidized glutathione (cysteine glutamate and glycine; Chen et al., 2022a), DL-methionine (Kaddurah-Daouk et al., 2013), N-acetylaspartic acid (Chen et al., 2000), and N-acetyl-L-2-aminoadipic acid (Rahimzadeh et al., 2024). A notable ketone body includes 3-hydroxybutanoate (also known as β-hydroxybutyrate; Han et al., 2025). Finally, decreased metabolites of pyruvate and the tricarboxylic acid cycle include acetylphosphate and 2-(acetamidomethylene)succinate, respectively. Relative to *3xTg-AD* vehicle, all ≈100 metabolites that differentiated *3xTg-AD* vs. wild-type vehicle mice were absent in CBD-treated *3xTg-AD* animals with exception of phosphate, which was increased in *3xTg-AD* relative wild-type vehicle but decreased in CBD-treated *3xTg-AD* animals relative to *3xTg-AD* vehicle.

### Behavior

Although naturally a pathological feature of human subjects and not wild-type rodents (Baerresen et al., 2015), AD is indeed a cognitive disorder and, thus, we also assessed learning, spatial, exploratory, and organizational behavior (Hartman et al., 2001; Rudobeck et al., 2017). Overall, the *3xTg-AD* mice demonstrated stunted spatial learning and memory patterns relative to age-matched wild-type progressively from 4.5 mo (cognitive impairment) to 6.5 mo (AD onset). With similar recognition of the overall MWM environment, the *3xTg-AD* mice required more time and distance to travel through the apparatus with favoring long-term over short-term memory, whereby CBD treatment improved the latter. A deficient memory of the target zone in the maze is also mildly restored in response to CBD. For the OFT, distribution of time spent in outer vs. inner zones is highly variable in a randomized pattern among *3xTg-AD* mice at both 4.5 and 6.5 mo, whereby the difference in the average percent time among respective areas is minimal. While also variable among individual animals, the overall distance traveled was higher in the *3xTg-AD* relative to wild-type mice. Thus, the *3xTg-AD* mice indicate signs of agitation and anxiety that is resistant to CBD treatment. As suggested by the nesting protocol, the organizational score is also slightly worse in *3xTg-AD* mice with little to no apparent effect of CBD. Altogether, in our hands using a mouse model, dietary CBD has the potential to address deficiencies in spatial memory and learning but not necessarily anxiety-like or executive decision-making behavior. The relatively short, subtle 2-month time window from 4.5 to 6.5 mo, even in *3xTg-AD* mice (Chum et al., 2022; Hakim and Behringer, 2020), should be a consideration for the mild phenotypical shifts observed throughout integrative behavioral analyses.

### Experimental considerations

Note that the current study involved several limitations that should be taken into account as findings are interpreted by the reader. First, the *3xTg-AD* mouse carries familial mutations of AD from conception as transgenes in amyloid precursor protein (APP; KM670/671NL) and microtubule-associated protein tau (MAPT; P301L) in combination with a knock-in mutation of presenilin 1 (PSEN1; M164V; Oddo et al., 2003; Javonillo et al., 2022). In contrast, >95% of human AD cases reflect sporadic or late-onset development of dementia pathology independent of the inheritance of rare, autosomal dominant gene mutations (Ulaganathan and Pitchaimani, 2023). Second, the role of biological sex for both *3xTg-AD* and age-matched wild-type mice was not examined as female animals were not included. Third, histopathological analyses have not been included and paired with respective findings for -omics and behavioral analyses. Fourth, there remains a need for in-depth analyses of pharmacokinetic profiling (absorption, distribution, metabolism, and excretion) of CBD among multiple concentrations and dietary treatment durations. Fifth, as an untargeted, comprehensive molecular study, the main narrative of the manuscript does not completely unpack ambiguous findings for age-matched wild-type mice (e.g., decrease in annotated neurovascular coupling pathway in 6.5 mo relative to 4.5 mo) with and without CBD treatment. In turn, precise mechanisms underlying the CBD-sensitive downregulation of neuroprotective fatty acids (e.g., omega-3s) in the brains of *3xTg-AD* mice relative to wild-type animals remain to be explored. Finally, immense investigative follow-up will be required for quantitation of corresponding functional changes per select differentially-expressed markers and their associated pathways.

## Summary and conclusion

Alzheimer's disease is the most widely recognized form of neurodegenerative disease (Cummings et al., 2025) involving genome-wide alterations in synaptic development, maintenance, and remodeling (Oswald et al., 2017; Hondius et al., 2016); overlap and integration of the cardiovascular and nervous systems (Chum et al., 2024; Santisteban et al., 2023); cell receptors and ionic transport (Behringer, 2023; Joshi et al., 2024); and mitochondrial structure and metabolism (D'Alessandro et al., 2025; Mosharov et al., 2025). Furthermore, all major pathological contributors are integral to Alzheimer's disease pathogenesis as inflammation (Amelimojarad et al., 2024), oxidative stress (Bhandari et al., 2024), dyslipidemia (de Oliveira et al., 2024), and insulin resistance (Kale et al., 2024). Given these complex molecular underpinnings, the challenges of accurately diagnosing and effectively treating Alzheimer's disease remain immense, requiring comprehensive multi-omics approaches to understand its molecular pathogenesis. Our prior work examined non-coding and coding RNA markers of cerebrovascular remodeling Alzheimer's disease in *3xTg-AD* animals with utility for tracking early-stage disease in particular (Chum et al., 2022, 2024). Thus, while ambitious, our current effort was to resolve significant transcriptomic and metabolomic shifts in a 2-month window of the animal's life and health span from the cognitive impairment phase to the onset of Alzheimer's disease (Oddo et al., 2003; Billings et al., 2005). In turn, we attempted to quantitate the molecular effects of daily dietary cannabidiol during this pathological shift as a broadly acting neurological therapeutic (Mallick et al., 2024) while concurrently in clinical trials for treatment of Alzheimer's disease in human subjects (Cummings et al., 2025). With concomitant analysis of blood and brain samples in a longitudinal or cross-sectional manner among study groups (with age-matched wild-type animals), ~1,000 genes and 100 metabolites marked the onset of Alzheimer's disease, whereby cannabidiol intake effectively eliminated or reversed expression of over 75% of significant markers. Based on our observations, we also maintain that the *3xTg-AD* study model is a suitable surrogate for illuminating the molecular landscape of Alzheimer's disease, despite the disease itself manifested as a human condition and not of rodents. Altogether, with all details enclosed in the primary manuscript and Supplementary Files, we hereby conclude that the onset of Alzheimer's disease represents a molecular integration of neurovascular interactions, channelopathies, metabolic disturbances, and developmental genes gone awry with notable overlap among other neurological (e.g., Parkinson's and frontotemporal dementia) and non-neurological (e.g., cancer) conditions. Remarkably, chronic cannabidiol treatment has the potential to widely address and almost completely disrupt molecular signatures of the onset of Alzheimer's disease.

## Data Availability

The transcriptome data discussed in this publication have been deposited in NCBI's Gene Expression Omnibus (Edgar et al., 2002; Barrett et al., 2013) and are accessible through GEO Series accession number GSE304212 (https://www.ncbi.nlm.nih.gov/geo/query/acc.cgi?acc=GSE304212).

## References

[B1] Abdul-Wahed A. Guilmeau S. Postic C. (2017). Sweet sixteenth for ChREBP: established roles and future goals. Cell Metab. 26, 324–341. 10.1016/j.cmet.2017.07.004 28768172

[B2] Adewuyi E. O. O'Brien E. K. Nyholt D. R. Porter T. Laws S. M. A. (2022). large-scale genome-wide cross-trait analysis reveals shared genetic architecture between Alzheimer's disease and gastrointestinal tract disorders. Commun. Biol. 5:691. 10.1038/s42003-022-03607-2 35851147 PMC9293965

[B3] Ahmad S. Yang W. Orellana A. Frolich L. de Rojas I. Cano A.. (2024). Association of oxidative stress and inflammatory metabolites with Alzheimer's disease cerebrospinal fluid biomarkers in mild cognitive impairment. Alzheimers Res. Ther. 16:171. 10.1186/s13195-024-01542-4 39080778 PMC11287840

[B4] Ahsanul Haque M. Omori N. Md Sheikh A. Yano S. Osago H. Mitaki S.. (2023). Analysis of the time-dependent changes of phospholipids in the brain regions of a mouse model of Alzheimer's disease. Brain Res. 1800:148197. 10.1016/j.brainres.2022.148197 36481236

[B5] Aihara Y. Mashima H. Onda H. Hisano S. Kasuya H. Hori T.. (2000). Molecular cloning of a novel brain-type Na(+)-dependent inorganic phosphate cotransporter. J. Neurochem. 74, 2622–2625. 10.1046/j.1471-4159.2000.0742622.x 10820226

[B6] Akawi N. A. Canpolat F. E. White S. M. Quilis-Esquerra J. Morales Sanchez M. Gamundi M. J.. (2013). Delineation of the clinical, molecular and cellular aspects of novel JAM3 mutations underlying the autosomal recessive hemorrhagic destruction of the brain, subependymal calcification, and congenital cataracts. Hum. Mutat. 34, 498–505. 10.1002/humu.22263 23255084 PMC3951164

[B7] Alemany S. Ribasés M. Vilor-Tejedor N. Bustamante M. Sánchez-Mora C. Bosch R.. (2015). New suggestive genetic loci and biological pathways for attention function in adult attention-deficit/hyperactivity disorder. Am. J. Med. Genet. B Neuropsychiatr. Genet. 168, 459–470. 10.1002/ajmg.b.32341 26174813

[B8] Allende L. G. Natali L. Cragnolini A. B. Bollo M. Musri M. M. de Mendoza D.. (2024). Lysosomal cholesterol accumulation in aged astrocytes impairs cholesterol delivery to neurons and can be rescued by cannabinoids. Glia 72, 1746–1765. 10.1002/glia.24580 38856177

[B9] Amelimojarad M. Amelimojarad M. Cui X. (2024). The emerging role of brain neuroinflammatory responses in Alzheimer's disease. Front. Aging Neurosci. 16:1391517. 10.3389/fnagi.2024.1391517 39021707 PMC11253199

[B10] Amro Z. Ryan M. Collins-Praino L. E. Yool A. J. (2023). Unexpected classes of aquaporin channels detected by transcriptomic analysis in human brain are associated with both patient age and Alzheimer's disease status. Biomedicines 11:770. 10.3390/biomedicines11030770 36979749 PMC10045580

[B11] Anazi S. Maddirevula S. Faqeih E. Alsedairy H. Alzahrani F. Shamseldin H. E.. (2017). Clinical genomics expands the morbid genome of intellectual disability and offers a high diagnostic yield. Mol. Psychiatry 22, 615–624. 10.1038/mp.2016.113 27431290

[B12] Andres-Benito P. Flores A. Busquet-Areny S. Carmona M. Ausin K. Cartas-Cejudo P.. (2023). Deregulated transcription and proteostasis in adult mapt knockout mouse. Int. J. Mol. Sci. 24:6559. 10.3390/ijms24076559 37047532 PMC10095510

[B13] Ansoleaga B. Jove M. Schluter A. Garcia-Esparcia P. Moreno J. Pujol A.. (2015). Deregulation of purine metabolism in Alzheimer's disease. Neurobiol. Aging 36, 68–80. 10.1016/j.neurobiolaging.2014.08.004 25311278

[B14] Arnaud L. Benech P. Greetham L. Stephan D. Jimenez A. Jullien N.. (2022). APOE4 drives inflammation in human astrocytes via TAGLN3 repression and NF-kappaB activation. Cell Rep. 40:111200. 10.1016/j.celrep.2022.111200 35977506

[B15] Arnold J. C. McCartney D. Suraev A. McGregor I. S. (2023). The safety and efficacy of low oral doses of cannabidiol: an evaluation of the evidence. Clin. Transl. Sci. 16, 10–30. 10.1111/cts.13425 36259271 PMC9841308

[B16] Asim M. Wang H. Waris A. Qianqian G. Chen X. (2024). Cholecystokinin neurotransmission in the central nervous system: insights into its role in health and disease. Biofactors 50, 1060–1075. 10.1002/biof.2081 38777339 PMC11627476

[B17] Atalay Ekiner S. Gegotek A. Skrzydlewska E. (2022). The molecular activity of cannabidiol in the regulation of Nrf2 system interacting with NF-kappaB pathway under oxidative stress. Redox Biol. 57:102489. 10.1016/j.redox.2022.102489 36198205 PMC9535304

[B18] Ates G. Goldberg J. Currais A. Maher P. (2020). CMS121, a fatty acid synthase inhibitor, protects against excess lipid peroxidation and inflammation and alleviates cognitive loss in a transgenic mouse model of Alzheimer's disease. Redox Biol. 36:101648. 10.1016/j.redox.2020.101648 32863221 PMC7394765

[B19] Baerresen K. M. Miller K. J. Hanson E. R. Miller J. S. Dye R. V. Hartman R. E.. (2015). Neuropsychological tests for predicting cognitive decline in older adults. Neurodegener. Dis. Manag. 5, 191–201. 10.2217/nmt.15.7 26107318 PMC4511118

[B20] Baird D. A. Liu J. Z. Zheng J. Sieberts S. K. Perumal T. Elsworth B.. (2021). Identifying drug targets for neurological and psychiatric disease via genetics and the brain transcriptome. PLoS Genet. 17:e1009224. 10.1371/journal.pgen.1009224 33417599 PMC7819609

[B21] Baranger K. van Gijsel-Bonnello M. Stephan D. Carpentier W. Rivera S. Khrestchatisky M.. (2019). Long-term pantethine treatment counteracts pathologic gene dysregulation and decreases Alzheimer's disease pathogenesis in a transgenic mouse model. Neurotherapeutics 16, 1237–1254. 10.1007/s13311-019-00754-z 31267473 PMC6985318

[B22] Barisano G. Kisler K. Wilkinson B. Nikolakopoulou A. M. Sagare A. P. Wang Y.. (2022). A “multi-omics” analysis of blood-brain barrier and synaptic dysfunction in APOE4 mice. J. Exp. Med. 219:e20221137. 10.1084/jem.20221137 36040482 PMC9435921

[B23] Barranco N. Pla V. Alcolea D. Sanchez-Dominguez I. Fischer-Colbrie R. Ferrer I.. (2021). Dense core vesicle markers in CSF and cortical tissues of patients with Alzheimer's disease. Transl. Neurodegener. 10:37. 10.1186/s40035-021-00263-0 34565482 PMC8466657

[B24] Barrett T. Wilhite S. E. Ledoux P. Evangelista C. Kim I. F. Tomashevsky M.. (2013). NCBI GEO: archive for functional genomics data sets–update. Nucleic Acids Res. 41:D991–5. 10.1093/nar/gks1193 23193258 PMC3531084

[B25] Beckmann N. D. Lin W. J. Wang M. Cohain A. T. Charney A. W. Wang P.. (2020). Multiscale causal networks identify VGF as a key regulator of Alzheimer's disease. Nat. Commun. 11:3942. 10.1038/s41467-020-17405-z 32770063 PMC7414858

[B26] Behringer E. J. (2023). Impact of aging on vascular ion channels: perspectives and knowledge gaps across major organ systems. Am. J. Physiol. Heart Circul. Physiol. 325, H1012–H38. 10.1152/ajpheart.00288.2023 37624095 PMC10908410

[B27] Belfiore R. Rodin A. Ferreira E. Velazquez R. Branca C. Caccamo A.. (2019). Temporal and regional progression of Alzheimer's disease-like pathology in 3xTg-AD mice. Aging Cell 18:e12873. 10.1111/acel.12873 30488653 PMC6351836

[B28] Benjamin-Zukerman T. Shimon G. Gaine M. E. Dakwar A. Peled N. Aboraya M.. (2024). A mutation in the PRKAR1B gene drives pathological mechanisms of neurodegeneration across species. Brain 147, 3890–3905. 10.1093/brain/awae154 38743596 PMC11531844

[B29] Benoit M. E. Hernandez M. X. Dinh M. L. Benavente F. Vasquez O. Tenner A. J.. (2013). C1q-induced LRP1B and GPR6 proteins expressed early in Alzheimer disease mouse models, are essential for the C1q-mediated protection against amyloid-beta neurotoxicity. J. Biol. Chem. 288, 654–665. 10.1074/jbc.M112.400168 23150673 PMC3537064

[B30] Bereczki E. Branca R. M. Francis P. T. Pereira J. B. Baek J. H. Hortobagyi T.. (2018). Synaptic markers of cognitive decline in neurodegenerative diseases: a proteomic approach. Brain 141, 582–595. 10.1093/brain/awx352 29324989 PMC5837272

[B31] Bhandari U. R. Danish S. M. Ahmad S. Ikram M. Nadaf A. Hasan N.. (2024). New opportunities for antioxidants in amelioration of neurodegenerative diseases. Mech. Ageing Dev. 221:111961. 10.1016/j.mad.2024.111961 38960099

[B32] Bi R. Zhang W. Zhang D. F. Xu M. Fan Y. Hu Q. X.. (2018). Genetic association of the cytochrome c oxidase-related genes with Alzheimer's disease in Han Chinese. Neuropsychopharmacology 43, 2264–2276. 10.1038/s41386-018-0144-3 30054583 PMC6135758

[B33] Billings L. M. Oddo S. Green K. N. McGaugh J. L. LaFerla F. M. (2005). Intraneuronal Abeta causes the onset of early Alzheimer's disease-related cognitive deficits in transgenic mice. Neuron 45, 675–688. 10.1016/j.neuron.2005.01.040 15748844

[B34] Bitencourt R. M. Takahashi R. N. (2018). Cannabidiol as a therapeutic alternative for post-traumatic stress disorder: from bench research to confirmation in human trials. Front. Neurosci. 12:502. 10.3389/fnins.2018.00502 30087591 PMC6066583

[B35] Bolivar D. A. Mosquera-Heredia M. I. Vidal O. M. Barcelo E. Allegri R. Morales L. C.. (2024). Exosomal mRNA signatures as predictive biomarkers for risk and age of onset in Alzheimer's disease. Int. J. Mol. Sci. 25:12293. 10.3390/ijms252212293 39596356 PMC11594294

[B36] Borkowski K. Pedersen T. L. Seyfried N. T. Lah J. J. Levey A. I. Hales C. M.. (2021). Association of plasma and CSF cytochrome P450, soluble epoxide hydrolase, and ethanolamide metabolism with Alzheimer's disease. Alzheimers Res. Ther. 13, 149. 10.1186/s13195-021-00893-6 34488866 PMC8422756

[B37] Bright U. Akirav I. (2025). Cannabidiol modulates neuroinflammatory and estrogen-related pathways in a sex-specific manner in a chronic stress model of depression. Cells. 14:99. 10.3390/cells14020099 39851527 PMC11763596

[B38] Broad K. D. Curley J. P. Keverne E. B. (2009). Increased apoptosis during neonatal brain development underlies the adult behavioral deficits seen in mice lacking a functional paternally expressed gene 3 (Peg3). Dev. Neurobiol. 69, 314–325. 10.1002/dneu.20702 19224563

[B39] Busse S. Steiner J. Glorius S. Dobrowolny H. Greiner-Bohl S. Mawrin C.. (2015). VGF expression by T lymphocytes in patients with Alzheimer's disease. Oncotarget 6, 14843–14851. 10.18632/oncotarget.3569 26142708 PMC4558119

[B40] Cao T. H. M. Le A. P. H. Tran T. T. Huynh V. K. Pham B. H. Le T. M.. (2023). Plasma cell-free RNA profiling of Vietnamese Alzheimer's patients reveals a linkage with chronic inflammation and apoptosis: a pilot study. Front. Mol. Neurosci. 16:1308610. 10.3389/fnmol.2023.1308610 38178908 PMC10764507

[B41] Carbone C. Piro G. Merz V. Simionato F. Santoro R. Zecchetto C.. (2018). Angiopoietin-like proteins in angiogenesis, inflammation and cancer. Int. J. Mol. Sci. 19:431. 10.3390/ijms19020431 29389861 PMC5855653

[B42] Carulli D. de Winter F. Verhaagen J. (2021). Semaphorins in adult nervous system plasticity and disease. Front. Synaptic Neurosci. 13:672891. 10.3389/fnsyn.2021.672891 34045951 PMC8148045

[B43] Carvalho K. Schartz N. D. Balderrama-Gutierrez G. Liang H. Y. Chu S. H. Selvan P.. (2022). Modulation of C5a-C5aR1 signaling alters the dynamics of AD progression. J. Neuroinflamm. 19:178. 10.1186/s12974-022-02539-2 35820938 PMC9277945

[B44] Chan R. B. Oliveira T. G. Cortes E. P. Honig L. S. Duff K. E. Small S. A.. (2012). Comparative lipidomic analysis of mouse and human brain with Alzheimer disease. J. Biol. Chem. 287, 2678–2688. 10.1074/jbc.M111.274142 22134919 PMC3268426

[B45] Chen D. Hughes E. D. Saunders T. L. Wu J. Vasquez M. N. H. Makinen T.. (2022). Angiogenesis depends upon EPHB4-mediated export of collagen IV from vascular endothelial cells. JCI Insight 7:e156928. 10.1172/jci.insight.156928 35015735 PMC8876457

[B46] Chen J. G. Charles H. C. Barboriak D. P. Doraiswamy P. M. (2000). Magnetic resonance spectroscopy in Alzheimer's disease: focus on N-acetylaspartate. Acta Neurol. Scand,. Supplc. 176, 20–26. 10.1034/j.1600-0404.2000.00303.x 11261801

[B47] Chen J. J. Thiyagarajah M. Song J. Chen C. Herrmann N. Gallagher D.. (2022a). Altered central and blood glutathione in Alzheimer's disease and mild cognitive impairment: a meta-analysis. Alzheimers Res. Ther. 14:23. 10.1186/s13195-022-00961-5 35123548 PMC8818133

[B48] Chen S. Acosta D. Li L. Liang J. Chang Y. Wang C.. (2022b). Wolframin is a novel regulator of tau pathology and neurodegeneration. Acta Neuropathol. 143, 547–569. 10.1007/s00401-022-02417-4 35389045 PMC13262681

[B49] Chen Y. Liu S. Ren Z. Wang F. Liang Q. Jiang Y.. (2024). Cross-ancestry analysis of brain QTLs enhances interpretation of schizophrenia genome-wide association studies. Am. J. Hum. Genet. 111, 2444–2457. 10.1016/j.ajhg.2024.09.001 39362218 PMC11568756

[B50] Cheng C. Li W. Zhang Z. Yoshimura S. Hao Q. Zhang C.. (2013). MicroRNA-144 is regulated by activator protein-1 (AP-1) and decreases expression of Alzheimer disease-related a disintegrin and metalloprotease 10 (ADAM10). J. Biol. Chem. 288, 13748–13761. 10.1074/jbc.M112.381392 23546882 PMC3650412

[B51] Cheng D. Low J. K. Logge W. Garner B. Karl T. (2014). Chronic cannabidiol treatment improves social and object recognition in double transgenic APPswe/PS1ΔE9 mice. Psychopharmacology 231, 3009–3017. 10.1007/s00213-014-3478-5 24577515

[B52] Cheslow L. Byrne M. Kopenhaver J. S. Iacovitti L. Smeyne R. J. Snook A. E.. (2024). GUCY2C signaling limits dopaminergic neuron vulnerability to toxic insults. NPJ Parkinsons Dis. 10:83. 10.1038/s41531-024-00697-z 38615030 PMC11016112

[B53] Choi K. Lee Y. Kim C. (2023). An *in silico* study for expanding the utility of cannabidiol in Alzheimer's disease therapeutic development. Int. J. Mol. Sci. 24:16013. 10.3390/ijms242116013 37959001 PMC10648567

[B54] Chum P. P. Bishara M. A. Solis S. R. Behringer E. J. (2024). Cerebrovascular miRNAs track early development of Alzheimer's disease and target molecular markers of angiogenesis and blood flow regulation. J. Alzheimers Dis. 99, S187–S234. 10.3233/JAD-230300 37458037 PMC10787821

[B55] Chum P. P. Hakim M. A. Behringer E. J. (2022). Cerebrovascular microRNA expression profile during early development of Alzheimer's disease in a mouse model. J. Alzheimers Dis. 85, 91–113. 10.3233/JAD-215223 34776451 PMC9169494

[B56] Cioclu M. C. Mosca I. Ambrosino P. Puzo D. Bayat A. Wortmann S. B.. (2023). KCNT2-related disorders: phenotypes, functional, and pharmacological properties. Ann. Neurol. 94, 332–349. 10.1002/ana.26662 37062836 PMC13007567

[B57] Clark C. Dayon L. Masoodi M. Bowman G. L. Popp J. (2021). An integrative multi-omics approach reveals new central nervous system pathway alterations in Alzheimer's disease. Alzheimers Res. Ther. 13:71. 10.1186/s13195-021-00814-7 33794997 PMC8015070

[B58] Coles M. Watt G. Kreilaus F. Karl T. (2020). Medium-dose chronic cannabidiol treatment reverses object recognition memory deficits of APP (Swe)/PS1DeltaE9 transgenic female mice. Front. Pharmacol. 11:587604. 10.3389/fphar.2020.587604 33424597 PMC7789874

[B59] Crepel A. De Wolf V. Brison N. Ceulemans B. Walleghem D. Peuteman G.. (2014). Association of CDH11 with non-syndromic ASD. Am. J. Med. Genet. B Neuropsychiatr. Genet. 165B, 391–398. 10.1002/ajmg.b.32243 24839052

[B60] Culotta L. Scalmani P. Vinci E. Terragni B. Sessa A. Broccoli V.. (2020). SULT4A1 modulates synaptic development and function by promoting the formation of PSD-95/NMDAR complex. J. Neurosci. 40, 7013–7026. 10.1523/JNEUROSCI.2194-19.2020 32801157 PMC7480244

[B61] Cummings J. L. Zhou Y. Lee G. Zhong K. Fonseca J. Leisgang-Osse A. M.. (2025). Alzheimer's disease drug development pipeline: 2025. Alzheimers Dement. 11:e70098. 10.1002/trc2.70098 40463637 PMC12131090

[B62] D'Alessandro M. C. B. Kanaan S. Geller M. Pratico D. Daher J. P. L. (2025). Mitochondrial dysfunction in Alzheimer's disease. Ageing Res. Rev. 107:102713. 10.1016/j.arr.2025.102713 40023293

[B63] D'Angelo C. Goldeck D. Pawelec G. Gaspari L. Di Iorio A. Paganelli R.. (2020). Exploratory study on immune phenotypes in Alzheimer's disease and vascular dementia. Eur. J. Neurol. 27, 1887–1894. 10.1111/ene.14360 32441872

[B64] Davaapil H. Hopkins J. Bonnin N. Papadaki V. Leung A. Kosuge H.. (2023). PRELP secreted from mural cells protects the function of blood brain barrier through regulation of endothelial cell-cell integrity. Front. Cell Dev. Biol. 11:1147625. 10.3389/fcell.2023.1147625 37936982 PMC10626469

[B65] de Oliveira J. Moreira E. L. G. de Bem A. F. (2024). Beyond cardiovascular risk: implications of Familial hypercholesterolemia on cognition and brain function. Ageing Res. Rev. 93:102149. 10.1016/j.arr.2023.102149 38056504

[B66] de Souza Silva M. A. Lenz B. Rotter A. Biermann T. Peters O. Ramirez A.. (2013). Neurokinin3 receptor as a target to predict and improve learning and memory in the aged organism. Proc. Natl. Acad. Sci. U.S.A. 110, 15097–15102. 10.1073/pnas.1306884110 23983264 PMC3773732

[B67] de Wilde M. C. Vellas B. Girault E. Yavuz A. C. Sijben J. W. (2017). Lower brain and blood nutrient status in Alzheimer's disease: results from meta-analyses. Alzheimers Dement. 3, 416–431. 10.1016/j.trci.2017.06.002 29067348 PMC5651428

[B68] Dearborn J. T. Nelvagal H. R. Rensing N. R. Takahashi K. Hughes S. M. Wishart T. M.. (2022). Effects of chronic cannabidiol in a mouse model of naturally occurring neuroinflammation, neurodegeneration, and spontaneous seizures. Sci. Rep. 12:11286. 10.1038/s41598-022-15134-5 35789177 PMC9253004

[B69] Deming Y. Filipello F. Cignarella F. Cantoni C. Hsu S. Mikesell R.. (2019). The MS4A gene cluster is a key modulator of soluble TREM2 and Alzheimer's disease risk. Sci. Transl. Med. 11:eaau2291. 10.1126/scitranslmed.aau2291 31413141 PMC6697053

[B70] Demontis D. Rajagopal V. M. Thorgeirsson T. E. Als T. D. Grove J. Leppala K.. (2019). Genome-wide association study implicates CHRNA2 in cannabis use disorder. Nat. Neurosci. 22, 1066–1074. 10.1038/s41593-019-0416-1 31209380 PMC7596896

[B71] Dhaka A. Costa R. M. Hu H. Irvin D. K. Patel A. Kornblum H. I.. (2003). The RAS effector RIN1 modulates the formation of aversive memories. J. Neurosci. 23, 748–757. 10.1523/JNEUROSCI.23-03-00748.2003 12574403 PMC6741936

[B72] Di Donato N. Jean Y. Y. Maga A. M. Krewson B. D. Shupp A. B. Avrutsky M. I.. (2016). Mutations in CRADD result in reduced caspase-2-mediated neuronal apoptosis and cause megalencephaly with a rare lissencephaly variant. Am. J. Hum. Genet. 99, 1117–1129. 10.1016/j.ajhg.2016.09.010 27773430 PMC5097945

[B73] Ding D. Liu J. Midic U. Wu Y. Dong K. Melnick A.. (2018). TDRD5 binds piRNA precursors and selectively enhances pachytene piRNA processing in mice. Nat. Commun. 9:127. 10.1038/s41467-017-02622-w 29317670 PMC5760656

[B74] Ding Y. Chen H. Yan Y. Qiu Y. Zhao A. Li B.. (2023). Relationship between FERMT2, CELF1, COPI, CHRNA2, and ABCA7 genetic polymorphisms and Alzheimer's disease risk in the Southern Chinese population. J. Alzheimers Dis. Rep. 7, 1247–1257. 10.3233/ADR-230072 38025799 PMC10657721

[B75] Do K. V. Hjorth E. Wang Y. Jun B. Kautzmann M. I. Ohshima M.. (2023). Cerebrospinal fluid profile of lipid mediators in Alzheimer's disease. Cell. Mol. Neurobiol. 43, 797–811. 10.1007/s10571-022-01216-5 35362880 PMC9957874

[B76] Edelbusch C. Cindrić S. Dougherty G. W. Loges N. T. Olbrich H. Rivlin J.. (2017). Mutation of serine/threonine protein kinase 36 (STK36) causes primary ciliary dyskinesia with a central pair defect. Hum. Mutat. 38, 964–969. 10.1002/humu.23261 28543983

[B77] Edfawy M. Guedes J. R. Pereira M. I. Laranjo M. Carvalho M. J. Gao X.. (2019). Abnormal mGluR-mediated synaptic plasticity and autism-like behaviours in Gprasp2 mutant mice. Nat. Commun. 10:1431. 10.1038/s41467-019-09382-9 30926797 PMC6440958

[B78] Edgar R. Domrachev M. Lash A. E. (2002). Gene Expression Omnibus: NCBI gene expression and hybridization array data repository. Nucleic Acids Res. 30, 207–210. 10.1093/nar/30.1.207 11752295 PMC99122

[B79] Egorova T. Biziaev N. Shuvalov A. Sokolova E. Mukba S. Evmenov K.. (2021). eIF3j facilitates loading of release factors into the ribosome. Nucleic Acids Res. 49, 11181–11196. 10.1093/nar/gkab854 34591963 PMC8565342

[B80] Ehtezazi T. Rahman K. Davies R. Leach A. G. (2023). The pathological effects of circulating hydrophobic bile acids in Alzheimer's disease. J. Alzheimers Dis. Rep. 7, 173–211. 10.3233/ADR-220071 36994114 PMC10041467

[B81] Fiala M. Hammock B. D. Hwang S. H. Whitelegge J. Paul K. Kaczor-Urbanowicz K. E.. (2025). Inhibitors of soluble epoxide hydrolase and cGAS/STING repair defects in amyloid-beta clearance underlying vascular complications of Alzheimer's disease. J. Alzheimers Dis. 104, 150–157. 10.1177/13872877241305965 39962970 PMC11969680

[B82] Fiala M. Mahanian M. Rosenthal M. Mizwicki M. T. Tse E. Cho T.. (2011). MGAT3 mRNA: a biomarker for prognosis and therapy of Alzheimer's disease by vitamin D and curcuminoids. J. Alzheimers Dis. 25, 135–144. 10.3233/JAD-2011-101950 21368380

[B83] Filippov V. Song M. A. Zhang K. Vinters H. V. Tung S. Kirsch W. M.. (2012). Increased ceramide in brains with Alzheimer's and other neurodegenerative diseases. J. Alzheimers Dis. 29, 537–547. 10.3233/JAD-2011-111202 22258513 PMC3643694

[B84] Fisher D. W. Dunn J. T. Keszycki R. Rodriguez G. Bennett D. A. Wilson R. S.. (2024). Unique transcriptional signatures correlate with behavioral and psychological symptom domains in Alzheimer's disease. Transl. Psychiatry 14:178. 10.1038/s41398-024-02878-z 38575567 PMC10995139

[B85] Frech F. H. Li G. Juday T. Ding Y. Mattke S. Khachaturian A.. (2024). Economic impact of progression from mild cognitive impairment to Alzheimer disease in the United States. J Prev Alzheimers Dis. 11, 983–991. 10.14283/jpad.2024.68 39044509 PMC11266270

[B86] Furstenberger G. Marks F. Krieg P. (2002). Arachidonate 8(S)-lipoxygenase. Prostaglandins Other Lipid Mediat. 68–69, 235–243. 10.1016/S0090-6980(02)00033-3 12432921

[B87] Gahete M. D. Rubio A. Cordoba-Chacon J. Gracia-Navarro F. Kineman R. D. Avila J.. (2010). Expression of the ghrelin and neurotensin systems is altered in the temporal lobe of Alzheimer's disease patients. J. Alzheimers Dis. 22, 819–828. 10.3233/JAD-2010-100873 20858966

[B88] Gan-Or Z. Alcalay R. N. Bar-Shira A. Leblond C. S. Postuma R. B. Ben-Shachar S.. (2015). Genetic markers of Restless Legs Syndrome in Parkinson disease. Parkinsonism Relat. Disord. 21, 582–585. 10.1016/j.parkreldis.2015.03.010 25817513 PMC4441838

[B89] Gazestani V. Kamath T. Nadaf N. M. Dougalis A. Burris S. J. Rooney B.. (2023). Early Alzheimer's disease pathology in human cortex involves transient cell states. Cell 186, 4438–4453.e23. 10.1016/j.cell.2023.08.005 37774681 PMC11107481

[B90] Ge X. Zhang Y. Zuo Y. Israr M. Li B. Yu P.. (2019). Transcriptomic analysis reveals the molecular mechanism of Alzheimer-related neuropathology induced by sevoflurane in mice. J. Cell. Biochem. 120, 17555–17565. 10.1002/jcb.29020 31134678

[B91] Gennery A. (2019). Recent advances in understanding RAG deficiencies. F1000Research. 8, F1000 Faculty Rev-148. 10.12688/f1000research.17056.1 30800289 PMC6364374

[B92] Ghose U. Sproviero W. Winchester L. Amin N. Zhu T. Newby D.. (2024). Genome-wide association neural networks identify genes linked to family history of Alzheimer's disease. Brief. Bioinformatics 26:bbae704. 10.1093/bib/bbae704 39775791 PMC11707606

[B93] Giusto E. Maistrello L. Iannotta L. Giusti V. Iovino L. Bandopadhyay R.. (2024). Prospective Role of PAK6 and 14-3-3gamma as Biomarkers for Parkinson's Disease. J. Parkinsons. Dis. 14, 495–506. 10.3233/JPD-230402 38640169 PMC11091598

[B94] Glenn N. A. K. Finlay D. B. Carruthers E. R. Mountjoy K. G. Walker C. S. Grimsey N. L. (2024). RAMP and MRAP accessory proteins have selective effects on expression and signalling of the CB(1), CB(2), GPR18 and GPR55 cannabinoid receptors. Br. J. Pharmacol. 181, 2212–2231. 10.1111/bph.16095 37085333

[B95] Goodman J. R. Adham Z. O. Woltjer R. L. Lund A. W. Iliff J. J. (2018). Characterization of dural sinus-associated lymphatic vasculature in human Alzheimer's dementia subjects. Brain Behav. Immun. 73, 34–40. 10.1016/j.bbi.2018.07.020 30055243 PMC6149215

[B96] Gorter J. A. Zurolo E. Iyer A. Fluiter K. van Vliet E. A. Baayen J. C.. (2010). Induction of sodium channel Na(x) (SCN7A) expression in rat and human hippocampus in temporal lobe epilepsy. Epilepsia 51, 1791–1800. 10.1111/j.1528-1167.2010.02678.x 20738386

[B97] Green R. Mayilsamy K. McGill A. R. Martinez T. E. Chandran B. Blair L. J.. (2022). SARS-CoV-2 infection increases the gene expression profile for Alzheimer's disease risk. Mol. Ther. Methods Clin. Dev. 27, 217–229. 10.1016/j.omtm.2022.09.007 36187720 PMC9508696

[B98] Griseti E. Bello A. A. Bieth E. Sabbagh B. Iacovoni J. S. Bigay J.. (2024). Molecular mechanisms of perilipin protein function in lipid droplet metabolism. FEBS Lett. 598, 1170–1198. 10.1002/1873-3468.14792 38140813

[B99] Guard S. E. Chapnick D. A. Poss Z. C. Ebmeier C. C. Jacobsen J. Nemkov T.. (2022). Multiomic analysis reveals disruption of cholesterol homeostasis by cannabidiol in human cell lines. Mol. Cell. Proteomics 21:100262. 10.1016/j.mcpro.2022.100262 35753663 PMC9525918

[B100] Guo L. K. Su Y. Zhang Y. Y. Yu H. Lu Z. Li W. Q.. (2023). Prediction of treatment response to antipsychotic drugs for precision medicine approach to schizophrenia: randomized trials and multiomics analysis. Mil. Med. Res. 10:24. 10.1186/s40779-023-00459-7 37269009 PMC10236828

[B101] Gurgul A. Zurowski J. Szmatola T. Kucharski M. Sawicki S. Semik-Gurgul E.. (2024). Cannabidiol (CBD) modulates the transcriptional profile of ethanol-exposed human dermal fibroblast cells. J. Appl. Genet. 65, 773–796. 10.1007/s13353-024-00915-7 39466591 PMC11561130

[B102] Haenig C. Atias N. Taylor A. K. Mazza A. Schaefer M. H. Russ J.. (2020). Interactome mapping provides a network of neurodegenerative disease proteins and uncovers widespread protein aggregation in affected brains. Cell Rep. 32:108050. 10.1016/j.celrep.2020.108050 32814053

[B103] Hakim M. A. Behringer E. J. (2020). Development of Alzheimer's disease progressively alters sex-dependent K_Ca_ and sex-independent K_IR_ channel function in cerebrovascular endothelium. J. Alzheimers Dis. 76, 1423–1442. 10.3233/JAD-200085 32651315 PMC7709862

[B104] Halks-Miller M. Schroeder M. L. Haroutunian V. Moenning U. Rossi M. Achim C.. (2003). CCR1 is an early and specific marker of Alzheimer's disease. Ann. Neurol. 54, 638–646. 10.1002/ana.10733 14595653

[B105] Han W. Zhang B. Zhao W. Zhao W. He J. Qiu X.. (2025). Ketogenic beta-hydroxybutyrate regulates beta-hydroxybutyrylation of TCA cycle-associated enzymes and attenuates disease-associated pathologies in Alzheimer's mice. Aging Cell 24:e14368. 10.1111/acel.14368 39411885 PMC11709107

[B106] Hanas J. S. Hocker J. R. S. Vannarath C. A. Lerner M. R. Blair S. G. Lightfoot S. A.. (2021). Distinguishing Alzheimer's disease patients and biochemical phenotype analysis using a novel serum profiling platform: potential involvement of the VWF/ADAMTS13 axis. Brain Sci. 11:583. 10.3390/brainsci11050583 33946285 PMC8145311

[B107] Hao F. Feng Y. (2021). Cannabidiol (CBD) enhanced the hippocampal immune response and autophagy of APP/PS1 Alzheimer's mice uncovered by RNA-seq. Life Sci. 264:118624. 10.1016/j.lfs.2020.118624 33096116

[B108] Hartman R. E. Wozniak D. F. Nardi A. Olney J. W. Sartorius L. Holtzman D. M.. (2001). Behavioral phenotyping of GFAP-apoE3 and -apoE4 transgenic mice: apoE4 mice show profound working memory impairments in the absence of Alzheimer's-like neuropathology. Exp. Neurol. 170, 326–344. 10.1006/exnr.2001.7715 11476599

[B109] Hass J. Walton E. Kirsten H. Turner J. Wolthusen R. Roessner V.. (2015). Complexin2 modulates working memory-related neural activity in patients with schizophrenia. Eur. Arch. Psychiatry Clin. Neurosci. 265, 137–145. 10.1007/s00406-014-0550-4 25297695 PMC4342303

[B110] Haytural H. Benfeitas R. Schedin-Weiss S. Bereczki E. Rezeli M. Unwin R. D.. (2021). Insights into the changes in the proteome of Alzheimer disease elucidated by a meta-analysis. Sci. Data 8:312. 10.1038/s41597-021-01090-8 34862388 PMC8642431

[B111] He S. Xu Z. Han X. (2025). Lipidome disruption in Alzheimer's disease brain: detection, pathological mechanisms, and therapeutic implications. Mol. Neurodegener. 20:11. 10.1186/s13024-025-00803-6 39871348 PMC11773937

[B112] Hinney A. Albayrak O. Antel J. Volckmar A. L. Sims R. Chapman J.. (2014). Genetic variation at the CELF1 (CUGBP, elav-like family member 1 gene) locus is genome-wide associated with Alzheimer's disease and obesity. Am. J. Med. Genet. B Neuropsychiatr. Genet. 165B, 283–293. 10.1002/ajmg.b.32234 24788522

[B113] Hondius D. C. van Nierop P. Li K. W. Hoozemans J. J. van der Schors R. C. van Haastert E. S.. (2016). Profiling the human hippocampal proteome at all pathologic stages of Alzheimer's disease. Alzheimers Dement. 12, 654–668. 10.1016/j.jalz.2015.11.002 26772638

[B114] Horgusluoglu-Moloch E. Risacher S. L. Crane P. K. Hibar D. Thompson P. M. Saykin A. J.. (2019). Genome-wide association analysis of hippocampal volume identifies enrichment of neurogenesis-related pathways. Sci. Rep. 9:14498. 10.1038/s41598-019-50507-3 31601890 PMC6787090

[B115] Hu R. T. Yu Q. Zhou S. D. Yin Y. X. Hu R. G. Lu H. P.. (2020). Co-expression network analysis reveals novel genes underlying Alzheimer's disease pathogenesis. Front. Aging Neurosci. 12:605961. 10.3389/fnagi.2020.605961 33324198 PMC7725685

[B116] Huh G. S. Boulanger L. M. Du H. Riquelme P. A. Brotz T. M. Shatz C. J.. (2000). Functional requirement for class I MHC in CNS development and plasticity. Science 290, 2155–2159. 10.1126/science.290.5499.2155 11118151 PMC2175035

[B117] Jacob C. P. Koutsilieri E. Bartl J. Neuen-Jacob E. Arzberger T. Zander N.. (2007). Alterations in expression of glutamatergic transporters and receptors in sporadic Alzheimer's disease. J. Alzheimers Dis. 11, 97–116. 10.3233/JAD-2007-11113 17361039

[B118] Janelidze S. Mattsson N. Stomrud E. Lindberg O. Palmqvist S. Zetterberg H.. (2018). CSF biomarkers of neuroinflammation and cerebrovascular dysfunction in early Alzheimer disease. Neurology 91, e867–e877. 10.1212/WNL.0000000000006082 30054439 PMC6133624

[B119] Javonillo D. I. Tran K. M. Phan J. Hingco E. Kramár E. A. da Cunha C.. (2022). Systematic phenotyping and characterization of the 3xTg-AD mouse model of Alzheimer's disease. Front. Neurosci. 15:785276. 10.3389/fnins.2021.785276 35140584 PMC8818877

[B120] Jha S. K. Nelson V. K. Suryadevara P. R. Panda S. P. Pullaiah C. P. Nuli M. V.. (2024). Cannabidiol and neurodegeneration: from molecular mechanisms to clinical benefits. Ageing Res. Rev. 100:102386. 10.1016/j.arr.2024.102386 38969143

[B121] Ji W. An K. Wang C. Wang S. (2022). Bioinformatics analysis of diagnostic biomarkers for Alzheimer's disease in peripheral blood based on sex differences and support vector machine algorithm. Hereditas 159:38. 10.1186/s41065-022-00252-x 36195955 PMC9531459

[B122] Jia E. Pan M. Liu Z. Zhou Y. Zhao X. Dong J.. (2020). Transcriptomic profiling of differentially expressed genes and related pathways in different brain regions in Parkinson's disease. Neurosci. Lett. 732:135074. 10.1016/j.neulet.2020.135074 32446776

[B123] Jiang S. Tang L. Zhao N. Yang W. Qiu Y. Chen H. Z. A.. (2016). Systems view of the differences between APOE epsilon4 carriers and non-carriers in Alzheimer's disease. Front. Aging Neurosci. 8:171. 10.3389/fnagi.2016.00171 27462267 PMC4941795

[B124] Jin J. K. Kim N. H. Lee Y. J. Kim Y. S. Choi E. K. Kozlowski P. B.. (2006). Phospholipase D1 is up-regulated in the mitochondrial fraction from the brains of Alzheimer's disease patients. Neurosci. Lett. 407, 263–267. 10.1016/j.neulet.2006.08.062 16973278

[B125] Johnson R. J. Gomez-Pinilla F. Nagel M. Nakagawa T. Rodriguez-Iturbe B. Sanchez-Lozada L. G.. (2020). Cerebral fructose metabolism as a potential mechanism driving Alzheimer's disease. Front. Aging Neurosci. 12:560865. 10.3389/fnagi.2020.560865 33024433 PMC7516162

[B126] Johnson R. J. Tolan D. R. Bredesen D. Nagel M. Sanchez-Lozada L. G. Fini M.. (2023). Could Alzheimer's disease be a maladaptation of an evolutionary survival pathway mediated by intracerebral fructose and uric acid metabolism? Am. J. Clin. Nutr. 117, 455–466. 10.1016/j.ajcnut.2023.01.002 36774227 PMC10196606

[B127] Joshi N. Vaidya B. Sharma S. S. (2024). Transient receptor potential channels as an emerging target for the treatment of Alzheimer's disease: unravelling the potential of pharmacological interventions. Basic Clin. Pharmacol. Toxicol. 135, 375–400. 10.1111/bcpt.14073 39209323

[B128] Jullienne A. Quan R. Szu J. I. Trinh M. V. Behringer E. J. Obenaus A.. (2022). Progressive vascular abnormalities in the aging 3xTg-AD mouse model of Alzheimer's disease. Biomedicines 10:1967. 10.3390/biomedicines10081967 36009514 PMC9405684

[B129] Kaddurah-Daouk R. Zhu H. Sharma S. Bogdanov M. Rozen S. G. Matson W.. (2013). Alterations in metabolic pathways and networks in Alzheimer's disease. Transl. Psychiatry 3:e244. 10.1038/tp.2013.18 23571809 PMC3641405

[B130] Kale M. B. Bhondge H. M. Wankhede N. L. Shende P. V. Thanekaer R. P. Aglawe M. M.. (2024). Navigating the intersection: diabetes and Alzheimer's intertwined relationship. Ageing Res. Rev. 2024:102415. 10.1016/j.arr.2024.102415 39002642

[B131] Kalra P. Grewal A. K. Khan H. Singh T. G. (2025). Unscrambling the cellular and molecular threads of neuroplasticity: insights into Alzheimer's disease pathogenesis. Neuroscience 571, 74–88. 10.1016/j.neuroscience.2025.02.037 39970983

[B132] Kang M. Ang T. F. A. Devine S. A. Sherva R. Mukherjee S. Trittschuh E. H.. (2023). A genome-wide search for pleiotropy in more than 100,000 harmonized longitudinal cognitive domain scores. Mol. Neurodegener. 18:40. 10.1186/s13024-023-00633-4 37349795 PMC10286470

[B133] Karaahmet B. Le L. Mendes M. S. Majewska A. K. O'Banion M. K. (2022). Repopulated microglia induce expression of Cxcl13 with differential changes in Tau phosphorylation but do not impact amyloid pathology. J. Neuroinflamm. 19:173. 10.1186/s12974-022-02532-9 35787714 PMC9252071

[B134] Kataoka K. Han S. I. Shioda S. Hirai M. Nishizawa M. Handa H.. (2002). MafA is a glucose-regulated and pancreatic beta-cell-specific transcriptional activator for the insulin gene. J. Biol. Chem. 277, 49903–49910. 10.1074/jbc.M206796200 12368292

[B135] Kawamura M. Goda N. Hariya N. Kimura M. Ishiyama S. Kubota T.. (2022). Medium-chain fatty acids enhance expression and histone acetylation of genes related to lipid metabolism in insulin-resistant adipocytes. Biochem. Biophys. Rep. 29:101196. 10.1016/j.bbrep.2021.101196 35028437 PMC8741418

[B136] Kedia N. Arhzaouy K. Pittman S. K. Sun Y. Batchelor M. Weihl C. C.. (2019). Desmin forms toxic, seeding-competent amyloid aggregates that persist in muscle fibers. Proc. Natl. Acad. Sci. U.S.A. 116, 16835–16840. 10.1073/pnas.1908263116 31371504 PMC6708308

[B137] Kim B. H. Seo S. W. Park Y. H. Kim J. Kim H. J. Jang H.. (2024). Clinical application of sparse canonical correlation analysis to detect genetic associations with cortical thickness in Alzheimer's disease. Front. Neurosci. 18:1428900. 10.3389/fnins.2024.1428900 39381682 PMC11458562

[B138] Kim H. Rhee S. J. Lee H. Han D. Lee T. Y. Kim M.. (2021). Identification of altered protein expression in major depressive disorder and bipolar disorder patients using liquid chromatography-tandem mass spectrometry. Psychiatry Res. 299:113850. 10.1016/j.psychres.2021.113850 33711561

[B139] Kitano J. Kimura K. Yamazaki Y. Soda T. Shigemoto R. Nakajima Y.. (2002). Tamalin, a PDZ domain-containing protein, links a protein complex formation of group 1 metabotropic glutamate receptors and the guanine nucleotide exchange factor cytohesins. J. Neurosci. 22, 1280–1289. 10.1523/JNEUROSCI.22-04-01280.2002 11850456 PMC6757580

[B140] Knowles M. R. Ostrowski L. E. Leigh M. W. Sears P. R. Davis S. D. Wolf W. E.. (2014). Mutations in RSPH1 cause primary ciliary dyskinesia with a unique clinical and ciliary phenotype. Am. J. Respir. Crit. Care Med. 189, 707–717. 10.1164/rccm.201311-2047OC 24568568 PMC3983840

[B141] Kolling L. J. Chimenti M. S. Marcinkiewcz C. A. (2025). Spatial differences in gene expression across the dorsal raphe nucleus in a model of early Alzheimer's disease. J. Alzheimers Dis. 103, 133–148. 10.1177/13872877241299119 39584353 PMC12047055

[B142] Kosoy R. Fullard J. F. Zeng B. Bendl J. Dong P. Rahman S.. (2022). Genetics of the human microglia regulome refines Alzheimer's disease risk loci. Nat. Genet. 54, 1145–1154. 10.1038/s41588-022-01149-1 35931864 PMC9388367

[B143] Koury S. Yarlagadda S. Moskalik-Liermo K. Popli N. Kim N. Apolito C.. (2007). Differential gene expression during terminal erythroid differentiation. Genomics 90, 574–582. 10.1016/j.ygeno.2007.06.010 17764892 PMC2205530

[B144] Krämer A. Green J. Pollard Jr. J. Tugendreich S. (2014). Causal analysis approaches in Ingenuity Pathway Analysis. Bioinformatics. 30, 523–530. 10.1093/bioinformatics/btt703 24336805 PMC3928520

[B145] Kreilaus F. Przybyla M. Ittner L. Karl T. (2022). Cannabidiol (CBD) treatment improves spatial memory in 14-month-old female TAU58/2 transgenic mice. Behav. Brain Res. 425:113812. 10.1016/j.bbr.2022.113812 35202719

[B146] Kunkle B. W. Grenier-Boley B. Sims R. Bis J. C. Damotte V. Naj A. C.. (2019). Genetic meta-analysis of diagnosed Alzheimer's disease identifies new risk loci and implicates Abeta, tau, immunity and lipid processing. Nat. Genet. 51, 414–430. 10.1038/s41588-019-0358-2 30820047 PMC6463297

[B147] Landfield P. W. Applegate M. D. Schmitzer-Osborne S. E. Naylor C. E. (1991). Phosphate/calcium alterations in the first stages of Alzheimer's disease: implications for etiology and pathogenesis. J. Neurol. Sci. 106, 221–229. 10.1016/0022-510X(91)90261-5 1802970

[B148] Laun A. S. Shrader S. H. Brown K. J. Song Z. H. (2019). GPR3, GPR6, and GPR12 as novel molecular targets: their biological functions and interaction with cannabidiol. Acta Pharmacol. Sin. 40, 300–308. 10.1038/s41401-018-0031-9 29941868 PMC6460361

[B149] Le Guen Y. Philippe C. Riviere D. Lemaitre H. Grigis A. Fischer C.. (2019). eQTL of KCNK2 regionally influences the brain sulcal widening: evidence from 15,597 UK Biobank participants with neuroimaging data. Brain Struct. Funct. 224, 847–857. 10.1007/s00429-018-1808-9 30519892 PMC6420450

[B150] Lee S. Y. Choi H. B. Park M. Choi I. S. An J. Kim A.. (2021). Novel KCNQ4 variants in different functional domains confer genotype- and mechanism-based therapeutics in patients with nonsyndromic hearing loss. Exp. Mol. Med. 53, 1192–1204. 10.1038/s12276-021-00653-4 34316018 PMC8333092

[B151] Li D. Tang J. Xu H. Fan X. Bai Y. Yang L.. (2008). Decreased hippocampal cell proliferation correlates with increased expression of BMP4 in the APPswe/PS1DeltaE9 mouse model of Alzheimer's disease. Hippocampus 18, 692–698. 10.1002/hipo.20428 18398851

[B152] Li L. Wei Y. Van Winkle L. Zhang Q. Y. Zhou X. Hu J.. (2011). Generation and characterization of a Cyp2f2-null mouse and studies on the role of CYP2F2 in naphthalene-induced toxicity in the lung and nasal olfactory mucosa. J. Pharmacol. Exp. Ther. 339, 62–71. 10.1124/jpet.111.184671 21730012 PMC3186285

[B153] Li Q. S. De Muynck L. (2021). Differentially expressed genes in Alzheimer's disease highlighting the roles of microglia genes including OLR1 and astrocyte gene CDK2AP1. Brain Behav. Immun. Health. 13:100227. 10.1016/j.bbih.2021.100227 34589742 PMC8474442

[B154] Li X. Zhang D. F. Bi R. Tan L. W. Chen X. Xu M.. (2023). Convergent transcriptomic and genomic evidence supporting a dysregulation of CXCL16 and CCL5 in Alzheimer's disease. Alzheimers Res. Ther. 15:17. 10.1186/s13195-022-01159-5 36670424 PMC9863145

[B155] Li Y. Xu K. Zhou A. Xu Z. Wu J. Peng X.. (2024). Integrative transcriptomics and proteomics analysis reveals THRSP's role in lipid metabolism. Genes 15:1562. 10.3390/genes15121562 39766829 PMC11675175

[B156] Lin S. H. Chien Y. C. Chiang W. W. Liu Y. Z. Lien C. C. Chen C. C.. (2015). Genetic mapping of ASIC4 and contrasting phenotype to ASIC1a in modulating innate fear and anxiety. Eur. J. Neurosci. 41, 1553–1568. 10.1111/ejn.12905 25828470

[B157] Ling F. Zhang C. Zhao X. Xin X. Zhao S. (2023). Identification of key genes modules linking diabetic retinopathy and circadian rhythm. Front. Immunol. 14:1260350. 10.3389/fimmu.2023.1260350 38124748 PMC10730663

[B158] Liu B. Yi D. Yu Z. Pan J. Ramirez K. Li S.. (2022). TMEM100, a lung-specific endothelium gene. Arterioscler. Thromb. Vasc. Biol. 42, 1495–1497. 10.1161/ATVBAHA.122.317683 36252125 PMC9691553

[B159] Liu L. Wu Q. Zhong W. Chen Y. Zhang W. Ren H.. (2020). Microarray analysis of differential gene expression in Alzheimer's disease identifies potential biomarkers with diagnostic value. Med. Sci. Monit. 26:e919249. 10.12659/MSM.919249 31984950 PMC7001516

[B160] Liu W. Liu Y. Yang Y. Ou W. Chen X. Huang B.. (2018). Metabolic biomarkers of aging and aging-related diseases in Chinese middle-aged and elderly men. J. Nutr. Health Aging 22, 1189–1197. 10.1007/s12603-018-1062-0 30498825 PMC12280475

[B161] Liu Y. (2024). Alzheimer's disease, aging, and cannabidiol treatment: a promising path to promote brain health and delay aging. Mol. Biol. Rep. 51:121. 10.1007/s11033-023-09162-1 38227160

[B162] Llano D. A. Devanarayan V. (2021). Alzheimer's disease neuroimaging I. Serum phosphatidylethanolamine and lysophosphatidylethanolamine levels differentiate Alzheimer's disease from controls and predict progression from mild cognitive impairment. J. Alzheimers Dis. 80, 311–319. 10.3233/JAD-201420 33523012

[B163] Loika Y. Loiko E. Feng F. Stallard E. Yashin A. I. Arbeev K.. (2023). Exogenous exposures shape genetic predisposition to lipids, Alzheimer's, and coronary heart disease in the MLXIPL gene locus. Aging 15, 3249–3272. 10.18632/aging.204665 37074818 PMC10449285

[B164] Loke S. Y. Wong P. T. Ong W. Y. (2017). Global gene expression changes in the prefrontal cortex of rabbits with hypercholesterolemia and/or hypertension. Neurochem. Int. 102, 33–56. 10.1016/j.neuint.2016.11.010 27890723

[B165] Long L. E. Chesworth R. Huang X. F. McGregor I. S. Arnold J. C. Karl T. (2010). A behavioural comparison of acute and chronic Delta9-tetrahydrocannabinol and cannabidiol in C57BL/6JArc mice. Int. J. Neuropsychopharmacol. 13, 861–876. 10.1017/S1461145709990605 19785914

[B166] Lourenco C. F. Ledo A. Barbosa R. M. Laranjinha J. (2017). Neurovascular uncoupling in the triple transgenic model of Alzheimer's disease: impaired cerebral blood flow response to neuronal-derived nitric oxide signaling. Exp. Neurol. 291, 36–43. 10.1016/j.expneurol.2017.01.013 28161255

[B167] Lu Y. Li D. Yu Y. Wang Q. Li A. Quan Y.. (2025). Cerebrospinal fluid VGF is associated with the onset and progression of Alzheimer's disease. J. Alzheimers Dis. 104, 1235–1242. 10.1177/13872877251323002 40095667

[B168] Macedo A. Gomez C. Rebelo M. A. Poza J. Gomes I. Martins S.. (2021). Risk variants in three Alzheimer's disease genes show association with EEG endophenotypes. J. Alzheimers Dis. 80, 209–223. 10.3233/JAD-200963 33522999 PMC8075394

[B169] Maezawa I. Jenkins D. P. Jin B. E. Wulff H. (2012). Microglial KCa3.1 channels as a potential therapeutic target for Alzheimer's disease. Int. J. Alzheimers Dis. 2012:868972. 10.1155/2012/868972 22675649 PMC3364551

[B170] Malik B. Devine H. Patani R. La Spada A. R. Hanna M. G. Greensmith L. (2019). Gene expression analysis reveals early dysregulation of disease pathways and links Chmp7 to pathogenesis of spinal and bulbar muscular atrophy. Sci. Rep. 9:3539. 10.1038/s41598-019-40118-3 30837566 PMC6401132

[B171] Mallick K. Khan M. F. Banerjee S. (2024). The anxiolytic effects of cannabinoids: a comprehensive review. Pharmacol. Biochem. Behav. 243:173828. 10.1016/j.pbb.2024.173828 39032530

[B172] Marcotte M. Bernardo A. Linga N. Perez-Romero C. A. Guillou J. L. Sibille E.. (2021). Handling techniques to reduce stress in mice. J. Vis. Exp. e62593. 10.3791/62593 34633376

[B173] Marksteiner J. Blasko I. Kemmler G. Koal T. Humpel C. (2018). Bile acid quantification of 20 plasma metabolites identifies lithocholic acid as a putative biomarker in Alzheimer's disease. Metabolomics 14:1. 10.1007/s11306-017-1297-5 29249916 PMC5725507

[B174] Masuho I. Xie K. Martemyanov K. A. (2013). Macromolecular composition dictates receptor and G protein selectivity of regulator of G protein signaling (RGS) 7 and 9-2 protein complexes in living cells. J. Biol. Chem. 288, 25129–25142. 10.1074/jbc.M113.462283 23857581 PMC3757177

[B175] McDew-White M. Lee E. Premadasa L. S. Alvarez X. Okeoma C. M. Mohan M.. (2023). Cannabinoids modulate the microbiota-gut-brain axis in HIV/SIV infection by reducing neuroinflammation and dysbiosis while concurrently elevating endocannabinoid and indole-3-propionate levels. J. Neuroinflamm. 20:62. 10.1186/s12974-023-02729-6 36890518 PMC9993397

[B176] Méndez-Acevedo K. M. Valdes V. J. Asanov A. Vaca L. (2017). A novel family of mammalian transmembrane proteins involved in cholesterol transport. Sci. Rep. 7:7450. 10.1038/s41598-017-07077-z 28785058 PMC5547113

[B177] Messinger D. Harris M. K. Cummings J. R. Thomas C. Yang T. Sweha S. R.. (2023). Therapeutic targeting of prenatal pontine ID1 signaling in diffuse midline glioma. Neuro-oncology 25, 54–67. 10.1093/neuonc/noac141 35605606 PMC9825316

[B178] Mohamed L. A. Zhu H. Mousa Y. M. Wang E. Qiu W. Q. Kaddoumi A.. (2017). Amylin enhances amyloid-beta peptide brain to blood efflux across the blood-brain barrier. J. Alzheimers Dis. 56, 1087–1099. 10.3233/JAD-160800 28059785 PMC5466167

[B179] Moiseenok A. G. Kanunnikova N. P. (2023). Brain CoA and acetyl CoA metabolism in mechanisms of neurodegeneration. Biochemistry 88, 466–480. 10.1134/S000629792304003X 37080933

[B180] Morris J. K. Piccolo B. D. John C. S. Green Z. D. Thyfault J. P. Adams S. H.. (2019). Oxylipin profiling of Alzheimer's disease in nondiabetic and type 2 diabetic elderly. Metabolites 9:177. 10.3390/metabo9090177 31491971 PMC6780570

[B181] Mortreux M. Foppen E. Denis R. G. Montaner M. Kassis N. Denom J.. (2019). New roles for prokineticin 2 in feeding behavior, insulin resistance and type 2 diabetes: studies in mice and humans. Mol. Metab. 29, 182–196. 10.1016/j.molmet.2019.08.016 31668389 PMC6812023

[B182] Mosharov E. V. Rosenberg A. M. Monzel A. S. Osto C. A. Stiles L. Rosoklija G. B.. (2025). A human brain map of mitochondrial respiratory capacity and diversity. Nature 641, 749–758. 10.1038/s41586-025-08740-6 40140564 PMC12770858

[B183] Mravinacova S. Alanko V. Bergstrom S. Bridel C. Pijnenburg Y. Hagman G.. (2024). CSF protein ratios with enhanced potential to reflect Alzheimer's disease pathology and neurodegeneration. Mol. Neurodegener. 19:15. 10.1186/s13024-024-00705-z 38350954 PMC10863228

[B184] Nair A. Greeny A. Rajendran R. Abdelgawad M. A. Ghoneim M. M. Raghavan R. P.. (2023). KIF1A-associated neurological disorder: an overview of a rare mutational disease. Pharmaceuticals 16:147. 10.3390/ph16020147 37259299 PMC9962247

[B185] Nasaruddin M. L. Pan X. McGuinness B. Passmore P. Kehoe P. G. Holscher C.. (2018). Evidence that parietal lobe fatty acids may be more profoundly affected in moderate Alzheimer's disease (AD) pathology than in severe AD pathology. Metabolites 8:69. 10.3390/metabo8040069 30373213 PMC6316131

[B186] Neely C. L. Pedemonte K. A. Boggs K. N. Flinn J. M. (2019). Nest building behavior as an early indicator of behavioral deficits in mice. J. Vis. Exp. e60139. 10.3791/60139 31680688

[B187] Nelson P. T. Estus S. Abner E. L. Parikh I. Malik M. Neltner J. H.. (2014). ABCC9 gene polymorphism is associated with hippocampal sclerosis of aging pathology. Acta Neuropathol. 127, 825–843. 10.1007/s00401-014-1282-2 24770881 PMC4113197

[B188] Nguyen H. D. Kim W. K. Huong Vu G. (2024). Molecular mechanisms implicated in protein changes in the Alzheimer's disease human hippocampus. Mech. Ageing Dev. 219:111930. 10.1016/j.mad.2024.111930 38554950

[B189] Nho K. Ramanan V. K. Horgusluoglu E. Kim S. Inlow M. H. Risacher S. L.. (2015). Comprehensive gene- and pathway-based analysis of depressive symptoms in older adults. J. Alzheimers Dis. 45, 1197–1206. 10.3233/JAD-148009 25690665 PMC4398648

[B190] Nie L. He K. Xie F. Xiao S. Li S. Xu J.. (2021). Loganin substantially ameliorates molecular deficits, pathologies and cognitive impairment in a mouse model of Alzheimer's disease. Aging 13, 23739–23756. 10.18632/aging.203646 34689137 PMC8580356

[B191] Noh H. Park C. Park S. Lee Y. S. Cho S. Y. Seo H.. (2014). Prediction of miRNA-mRNA associations in Alzheimer's disease mice using network topology. BMC Genom. 15:644. 10.1186/1471-2164-15-644 25086961 PMC4132902

[B192] O'Day D. H. (2022). Calmodulin binding domains in critical risk proteins involved in neurodegeneration. Curr. Issues Mol. Biol. 44, 5802–5814. 10.3390/cimb44110394 36421678 PMC9689381

[B193] Oddo S. Caccamo A. Shepherd J. D. Murphy M. P. Golde T. E. Kayed R.. (2003). Triple-transgenic model of Alzheimer's disease with plaques and tangles: intracellular Abeta and synaptic dysfunction. Neuron 39, 409–421. 10.1016/S0896-6273(03)00434-3 12895417

[B194] Oswald F. Kloble P. Ruland A. Rosenkranz D. Hinz B. Butter F.. (2017). The FOXP2-driven network in developmental disorders and neurodegeneration. Front. Cell. Neurosci. 11:212. 10.3389/fncel.2017.00212 28798667 PMC5526973

[B195] Padovani A. Cosseddu M. Premi E. Archetti S. Papetti A. Agosti C.. (2010). The speech and language FOXP2 gene modulates the phenotype of frontotemporal lobar degeneration. J. Alzheimers Dis. 22, 923–931. 10.3233/JAD-2010-101206 20858950

[B196] Pendse A. A. Arbones-Mainar J. M. Johnson L. A. Altenburg M. K. Maeda N. (2009). Apolipoprotein E knock-out and knock-in mice: atherosclerosis, metabolic syndrome, and beyond. J. Lipid Res. 50(Suppl):S178–182. 10.1194/jlr.R800070-JLR200 19060252 PMC2674752

[B197] Perez-Gonzalez M. Mendioroz M. Badesso S. Sucunza D. Roldan M. Espelosin M.. (2020). PLA2G4E, a candidate gene for resilience in Alzheimer s disease and a new target for dementia treatment. Prog. Neurobiol. 191:101818. 10.1016/j.pneurobio.2020.101818 32380223

[B198] Piton A. Gauthier J. Hamdan F. F. Lafrenière R. G. Yang Y. Henrion E.. (2011). Systematic resequencing of X-chromosome synaptic genes in autism spectrum disorder and schizophrenia. Mol. Psychiatry 16, 867–880. 10.1038/mp.2010.54 20479760 PMC3289139

[B199] Privitera M. von Ziegler L. M. Floriou-Servou A. Duss S. N. Zhang R. Waag R.. (2024). Noradrenaline release from the locus coeruleus shapes stress-induced hippocampal gene expression. Elife 12:RP88559. 10.7554/eLife.88559.3.sa2 38477670 PMC10937036

[B200] Puthiyedth N. Riveros C. Berretta R. Moscato P. (2016). Identification of differentially expressed genes through integrated study of Alzheimer's disease affected brain regions. PLoS ONE 11:e0152342. 10.1371/journal.pone.0152342 27050411 PMC4822961

[B201] Qiu X. Guo D. Du J. Bai Y. Wang F. (2021). A novel biomarker, MRPS12 functions as a potential oncogene in ovarian cancer and is a promising prognostic candidate. Medicine 100:e24898. 10.1097/MD.0000000000024898 33663122 PMC7909224

[B202] Quintana D. D. Anantula Y. Garcia J. A. Engler-Chiurazzi E. B. Sarkar S. N. Corbin D. R.. (2021). Microvascular degeneration occurs before plaque onset and progresses with age in 3xTg AD mice. Neurobiol. Aging 105, 115–128. 10.1016/j.neurobiolaging.2021.04.019 34062487 PMC9703920

[B203] Rahimzadeh N. Srinivasan S. S. Zhang J. Swarup V. (2024). Gene networks and systems biology in Alzheimer's disease: insights from multi-omics approaches. Alzheimers Dement. 20, 3587–3605. 10.1002/alz.13790 38534018 PMC11095483

[B204] Ramos-Miguel A. Jones A. A. Sawada K. Barr A. M. Bayer T. A. Falkai P.. (2018). Frontotemporal dysregulation of the SNARE protein interactome is associated with faster cognitive decline in old age. Neurobiol. Dis. 114, 31–44. 10.1016/j.nbd.2018.02.006 29496544 PMC6483375

[B205] Rashid B. Glasser M. F. Nichols T. Van Essen D. Juttukonda M. R. Schwab N. A.. (2023). Cardiovascular and metabolic health is associated with functional brain connectivity in middle-aged and older adults: results from the Human Connectome Project-Aging study. Neuroimage 276:120192. 10.1016/j.neuroimage.2023.120192 37247763 PMC10330931

[B206] Rudel L. L. Lee R. G. Parini P. (2005). ACAT2 is a target for treatment of coronary heart disease associated with hypercholesterolemia. Arterioscler. Thromb. Vasc. Biol. 25, 1112–1118. 10.1161/01.ATV.0000166548.65753.1e 15831806

[B207] Rudobeck E. Bellone J. A. Szucs A. Bonnick K. Mehrotra-Carter S. Badaut J.. (2017). Low-dose proton radiation effects in a transgenic mouse model of Alzheimer's disease - Implications for space travel. PLoS ONE 12:e0186168. 10.1371/journal.pone.0186168 29186131 PMC5706673

[B208] Saadi M. Karkhah A. Pourabdolhossein F. Ataie A. Monif M. Nouri H. R.. (2020). Involvement of NLRC4 inflammasome through caspase-1 and IL-1beta augments neuroinflammation and contributes to memory impairment in an experimental model of Alzheimer's like disease. Brain Res. Bull. 154, 81–90. 10.1016/j.brainresbull.2019.10.010 31715312

[B209] Sanbe A. Osinska H. Villa C. Gulick J. Klevitsky R. Glabe C. G.. (2005). Reversal of amyloid-induced heart disease in desmin-related cardiomyopathy. Proc. Natl. Acad. Sci. U.S.A. 102, 13592–13597. 10.1073/pnas.0503324102 16155124 PMC1224623

[B210] Sánchez-Sánchez J. L. Ader I. Jeanson Y. Planat-Benard V. Vellas B. Casteilla L.. (2023). Periostin plasma levels and changes on physical and cognitive capacities in community-dwelling older adults. J. Gerontol. A Biol. Sci. Med. Sci. 78, 424–432. 10.1093/gerona/glac226 36373873 PMC9977245

[B211] Santisteban M. M. Iadecola C. Carnevale D. (2023). Hypertension, neurovascular dysfunction, and cognitive impairment. Hypertension 80, 22–34. 10.1161/HYPERTENSIONAHA.122.18085 36129176 PMC9742151

[B212] Saroja S. R. Sharma A. Hof P. R. Pereira A. C. (2022). Differential expression of tau species and the association with cognitive decline and synaptic loss in Alzheimer's disease. Alzheimers Dement. 18, 1602–1615. 10.1002/alz.12518 34873815 PMC9170833

[B213] Schirwani S. Woods E. Koolen D. A. Ockeloen C. W. Lynch S. A. Kavanagh K.. (2023). Familial Bainbridge-Ropers syndrome: report of familial ASXL3 inheritance and a milder phenotype. Am. J. Med. Genet. A 191, 29–36. 10.1002/ajmg.a.62981 36177608 PMC10087684

[B214] Schuele L. L. Schuermann B. Bilkei-Gorzo A. Gorgzadeh S. Zimmer A. Leidmaa E.. (2022). Regulation of adult neurogenesis by the endocannabinoid-producing enzyme diacylglycerol lipase alpha (DAGLa). Sci. Rep. 12:633. 10.1038/s41598-021-04600-1 35022487 PMC8755832

[B215] Seo Y. Mo S. Kim S. Kim H. Park H. C. (2022). Tamalin function is required for the survival of neurons and oligodendrocytes in the CNS. Int. J. Mol. Sci. 23:13395. 10.3390/ijms232113395 36362204 PMC9654138

[B216] Serikawa T. Kunisawa N. Shimizu S. Kato M. Alves Iha H. Kinboshi M.. (2019). Increased seizure sensitivity, emotional defects and cognitive impairment in PHD finger protein 24 (Phf24)-null rats. Behav. Brain Res. 369:111922. 10.1016/j.bbr.2019.111922 31039378

[B217] Seshadri S. Fitzpatrick A. L. Ikram M. A. DeStefano A. L. Gudnason V. Boada M.. (2010). Genome-wide analysis of genetic loci associated with Alzheimer disease. JAMA 303, 1832–1840. 10.1001/jama.2010.574 20460622 PMC2989531

[B218] Seto M. Weiner R. L. Dumitrescu L. Mahoney E. R. Hansen S. L. Janve V.. (2022). RNASE6 is a novel modifier of APOE-epsilon4 effects on cognition. Neurobiol. Aging 118, 66–76. 10.1016/j.neurobiolaging.2022.06.011 35896049 PMC9721357

[B219] Shigemizu D. Mori T. Akiyama S. Higaki S. Watanabe H. Sakurai T.. (2020). Identification of potential blood biomarkers for early diagnosis of Alzheimer's disease through RNA sequencing analysis. Alzheimers Res. Ther. 12:87. 10.1186/s13195-020-00654-x 32677993 PMC7367375

[B220] Shinto L. H. Raber J. Mishra A. Roese N. Silbert L. C. A. (2022). Review of oxylipins in Alzheimer's disease and related dementias (ADRD): potential therapeutic targets for the modulation of vascular tone and inflammation. Metabolites 12:826. 10.3390/metabo12090826 36144230 PMC9501361

[B221] Siegert S. Grisold A. Pal-Handl K. Lilja S. Kepa S. Silvaieh S.. (2024). Developmental, cognitive, ocular motor, and neuroimaging findings related to SUFU haploinsufficiency: unraveling subtle and highly variable phenotypes. Pediatr. Neurol. 160, 38–44. 10.1016/j.pediatrneurol.2024.07.015 39181021

[B222] Squitti R. Catalli C. Gigante L. Marianetti M. Rosari M. Mariani S.. (2023). Non-ceruloplasmin copper identifies a subtype of Alzheimer's disease (CuAD): characterization of the cognitive profile and case of a CuAD patient carrying an RGS7 stop-loss variant. Int. J. Mol. Sci. 24:6377. 10.3390/ijms24076377 37047347 PMC10094789

[B223] Stevens L. M. Brown R. E. (2015). Reference and working memory deficits in the 3xTg-AD mouse between 2 and 15-months of age: a cross-sectional study. Behav. Brain Res. 278, 496–505. 10.1016/j.bbr.2014.10.033 25446812

[B224] Stover K. R. Campbell M. A. Van Winssen C. M. Brown R. E. (2015). Early detection of cognitive deficits in the 3xTg-AD mouse model of Alzheimer's disease. Behav. Brain Res. 289, 29–38. 10.1016/j.bbr.2015.04.012 25896362

[B225] Subramanian S. Ayala P. Wadsworth T. L. Harris C. J. Vandenbark A. A. Quinn J. F.. (2010). CCR6: a biomarker for Alzheimer's-like disease in a triple transgenic mouse model. J. Alzheimers Dis. 22, 619–629. 10.3233/JAD-2010-100852 20847401 PMC2988888

[B226] Sun D. Li X. Nie S. Liu J. Wang S. (2023a). Disorders of cancer metabolism: the therapeutic potential of cannabinoids. Biomed. Pharmacother. 157:113993. 10.1016/j.biopha.2022.113993 36379120

[B227] Sun Y. Y. Wang Z. Huang H. C. (2023b). Roles of ApoE4 on the pathogenesis in Alzheimer's disease and the potential therapeutic approaches. Cell. Mol. Neurobiol. 43, 3115–3136. 10.1007/s10571-023-01365-1 37227619 PMC10211310

[B228] Sung Y. J. Winkler T. W. de las Fuentes L. Bentley A. R. Brown M. R. Kraja A. T.. (2018). A large-scale multi-ancestry genome-wide study accounting for smoking behavior identifies multiple significant loci for blood pressure. Am. J. Hum. Genet. 102, 375–400. 10.1016/j.ajhg.2018.01.015 29455858 PMC5985266

[B229] Sutherland G. T. Janitz M. Kril J. J. (2011). Understanding the pathogenesis of Alzheimer's disease: will RNA-Seq realize the promise of transcriptomics? J. Neurochem. 116, 937–946. 10.1111/j.1471-4159.2010.07157.x 21175619

[B230] Tandon R. Levey A. I. Lah J. J. Seyfried N. T. Mitchell C. S. (2023). Machine learning selection of most predictive brain proteins suggests role of sugar metabolism in Alzheimer's disease. J. Alzheimers Dis. 92, 411–424. 10.3233/JAD-220683 36776048 PMC10041447

[B231] Teixeira F. C. Gutierres J. M. Soares M. S. P. da Siveira de Mattos B. Spohr L. do Couto C. A. T.. (2020). Inosine protects against impairment of memory induced by experimental model of Alzheimer disease: a nucleoside with multitarget brain actions. Psychopharmacology 237, 811–823. 10.1007/s00213-019-05419-5 31834453

[B232] Tian X. Qin Y. Tian Y. Ge X. Cui J. Han H.. (2022). Identification of vascular dementia and Alzheimer's disease hub genes expressed in the frontal lobe and temporal cortex by weighted co-expression network analysis and construction of a protein-protein interaction. Int. J. Neurosci. 132, 1049–1060. 10.1080/00207454.2020.1860966 33401985

[B233] Tijsen A. J. Pinto Y. M. Creemers E. E. (2012). Circulating microRNAs as diagnostic biomarkers for cardiovascular diseases. Am. J. Physiol. Heart Circ. Physiol. 303, H1085–H1095. 10.1152/ajpheart.00191.2012 22942181

[B234] Tohgi H. Abe T. Takahashi S. Kimura M. Takahashi J. Kikuchi T.. (1992). Concentrations of serotonin and its related substances in the cerebrospinal fluid in patients with Alzheimer type dementia. Neurosci. Lett. 141, 9–12. 10.1016/0304-3940(92)90322-X 1508406

[B235] Tran Q. Sudasinghe A. Jones B. Xiong K. Cohen R. E. Sharlin D. S.. (2021). FAM171B is a novel polyglutamine protein widely expressed in the mammalian brain. Brain Res. 1766:147540. 10.1016/j.brainres.2021.147540 34052262

[B236] Traylor M. Malik R. Nalls M. A. Cotlarciuc I. Radmanesh F. Thorleifsson G.. (2017). Genetic variation at 16q24.2 is associated with small vessel stroke. Ann. Neurol. 81, 383–394. 10.1002/ana.24840 27997041 PMC5366092

[B237] Trigueros-Motos L. van Capelleveen J. C. Torta F. Castaño D. Zhang L. H. Chai E. C.. (2017). ABCA8 regulates cholesterol efflux and high-density lipoprotein cholesterol levels. Arterioscler. Thromb. Vasc. Biol. 37, 2147–2155. 10.1161/ATVBAHA.117.309574 28882873

[B238] Tsao C. H. Hsieh W. C. Lin F. J. Yang R. Y. Chang M. T. Apaya M. K.. (2023). The critical role of galectin-12 in modulating lipid metabolism in sebaceous glands. J. Invest. Dermatol. 143, 913–924. 10.1016/j.jid.2022.11.012 36535362

[B239] Tu Z. Cohen M. Bu H. Lin F. (2010). Tissue distribution and functional analysis of Sushi domain-containing protein 4. Am. J. Pathol. 176, 2378–2384. 10.2353/ajpath.2010.091036 20348246 PMC2861102

[B240] Turco E. M. Giovenale A. M. G. Sireno L. Mazzoni M. Cammareri A. Marchioretti C.. (2022). Retinoic acid-induced 1 gene haploinsufficiency alters lipid metabolism and causes autophagy defects in Smith-Magenis syndrome. Cell Death Dis. 13:981. 10.1038/s41419-022-05410-7 36411275 PMC9678881

[B241] Uemura M. T. Ihara M. Maki T. Nakagomi T. Kaji S. Uemura K.. (2018). Pericyte-derived bone morphogenetic protein 4 underlies white matter damage after chronic hypoperfusion. Brain Pathol. 28, 521–535. 10.1111/bpa.12523 28470822 PMC6099372

[B242] Ugarte A. Gil-Bea F. Garcia-Barroso C. Cedazo-Minguez A. Ramirez M. J. Franco R.. (2015). Decreased levels of guanosine3′,5′-monophosphate (cGMP) in cerebrospinal fluid (CSF) are associated with cognitive decline and amyloid pathology in Alzheimer's disease. Neuropathol. Appl. Neurobiol. 41, 471–482. 10.1111/nan.12203 25488891

[B243] Ulaganathan S. Pitchaimani A. (2023). Spontaneous and familial models of Alzheimer's disease: challenges and advances in preclinical research. Life Sci. 328:121918. 10.1016/j.lfs.2023.121918 37422070

[B244] Usui N. Co M. Konopka G. (2014). Decoding the molecular evolution of human cognition using comparative genomics. Brain Behav. Evol. 84, 103–116. 10.1159/000365182 25247723 PMC4174362

[B245] Vervuurt M. Schrader J. M. de Kort A. M. Kersten I. Wessels H. Klijn C. J. M.. (2024). Cerebrospinal fluid shotgun proteomics identifies distinct proteomic patterns in cerebral amyloid angiopathy rodent models and human patients. Acta Neuropathol. Commun. 12:6. 10.1186/s40478-023-01698-4 38191511 PMC10775534

[B246] Walker K. A. An Y. Moghekar A. Moaddel R. Duggan M. R. Peng Z.. (2024). Proteomic analysis of APOEepsilon4 carriers implicates lipid metabolism, complement and lymphocyte signaling in cognitive resilience. Mol. Neurodegener. 19:81. 10.1186/s13024-024-00772-2 39482741 PMC11526661

[B247] Wallace C. H. Oliveros G. Xie L. Serrano P. Rockwell P. Figueiredo-Pereira M.. (2024). Potential Alzheimer's early biomarkers in a transgenic rat model and benefits of diazoxide/dibenzoylmethane co-treatment on spatial memory and AD-pathology. Sci. Rep. 14:3730. 10.1038/s41598-024-54156-z 38355687 PMC10867006

[B248] Wang B. Fu C. Wei Y. Xu B. Yang R. Li C.. (2022a). Ferroptosis-related biomarkers for Alzheimer's disease: identification by bioinformatic analysis in hippocampus. Front. Cell. Neurosci. 16:1023947. 10.3389/fncel.2022.1023947 36467613 PMC9709107

[B249] Wang B. Liu W. Sun F. (2022b). Nucleosome assembly protein 1-like 5 alleviates Alzheimer's disease-like pathological characteristics in a cell model. Front. Mol. Neurosci. 15:1034766. 10.3389/fnmol.2022.1034766 36568274 PMC9773259

[B250] Wang H. Bennett D. A. De Jager P. L. Zhang Q. Y. Zhang H. Y. (2021). Genome-wide epistasis analysis for Alzheimer's disease and implications for genetic risk prediction. Alzheimers Res. Ther. 13:55. 10.1186/s13195-021-00794-8 33663605 PMC7934265

[B251] Wang L. Qin Y. Song J. Xu J. Quan W. Su H.. (2024a). Integrated analysis of single-cell RNA sequencing and bulk transcriptome data identifies a pyroptosis-associated diagnostic model for Parkinson's disease. Sci. Rep. 14:28548. 10.1038/s41598-024-80185-9 39558055 PMC11574289

[B252] Wang Q. Zhou Q. Zhang S. Shao W. Yin Y. Li Y.. (2016). Elevated Hapln2 expression contributes to protein aggregation and neurodegeneration in an animal model of Parkinson's disease. Front. Aging Neurosci. 8:197. 10.3389/fnagi.2016.00197 27601993 PMC4993759

[B253] Wang T. Zhang W. Maclin J. M. A. Xu H. Hong B. Yan F.. (2024b). Novel panel of long noncoding RNAs as diagnostic biomarkers for amnestic mild cognitive impairment in peripheral blood. J. Alzheimers Dis. 99, 1385–1396. 10.3233/JAD-231446 38788072

[B254] Wang W. Lu J. Pan N. Zhang H. Dai J. Li J.. (2024c). Identification of early Alzheimer's disease subclass and signature genes based on PANoptosis genes. Front. Immunol. 15:1462003. 10.3389/fimmu.2024.1462003 39650656 PMC11621049

[B255] Wang X. Liu L. Jiang X. Saredy J. Xi H. Cueto R.. (2023). Identification of methylation-regulated genes modulating microglial phagocytosis in hyperhomocysteinemia-exacerbated Alzheimer's disease. Alzheimers Res. Ther. 15:164. 10.1186/s13195-023-01311-9 37789414 PMC10546779

[B256] Watt G. Shang K. Zieba J. Olaya J. Li H. Garner B.. (2020). Chronic treatment with 50 mg/kg cannabidiol improves cognition and moderately reduces Abeta40 levels in 12-month-old male AbetaPPswe/PS1DeltaE9 transgenic mice. J. Alzheimers Dis. 74, 937–950. 10.3233/JAD-191242 32116258

[B257] Welcker J. E. Hernandez-Miranda L. R. Paul F. E. Jia S. Ivanov A. Selbach M.. (2013). Insm1 controls development of pituitary endocrine cells and requires a SNAG domain for function and for recruitment of histone-modifying factors. Development 140, 4947–4958. 10.1242/dev.097642 24227653

[B258] West J. D. Austin E. D. Rizzi E. M. Yan L. Tanjore H. Crabtree A. L.. (2021). KCNK3 mutation causes altered immune function in pulmonary arterial hypertension patients and mouse models. Int. J. Mol. Sci. 22:5014. 10.3390/ijms22095014 34065088 PMC8126011

[B259] Whelan C. D. Mattsson N. Nagle M. W. Vijayaraghavan S. Hyde C. Janelidze S.. (2019). Multiplex proteomics identifies novel CSF and plasma biomarkers of early Alzheimer's disease. Acta Neuropathol. Commun. 7:169. 10.1186/s40478-019-0795-2 31694701 PMC6836495

[B260] Widjaya M. A. Cheng Y. J. Kuo Y. M. Liu C. H. Cheng W. C. Lee S. D.. (2023). Transcriptomic analyses of exercise training in Alzheimer's disease cerebral cortex. J. Alzheimers Dis. 93, 349–363. 10.3233/JAD-221139 36970901

[B261] Wilson-Poe A. R. Smith T. Elliott M. R. Kruger D. J. Boehnke K. F. (2023). Past-year use prevalence of cannabidiol, cannabigerol, cannabinol, and Delta8-tetrahydrocannabinol among US adults. JAMA Netw. Open 6:e2347373. 10.1001/jamanetworkopen.2023.47373 38091045 PMC10719758

[B262] Wright N. J. D. (2024). A review of the direct targets of the cannabinoids cannabidiol, Delta9-tetrahydrocannabinol, N-arachidonoylethanolamine and 2-arachidonoylglycerol. AIMS Neurosci. 11, 144–165. 10.3934/Neuroscience.2024009 38988890 PMC11230856

[B263] Wu N. Wang Y. Jia J. Y. Pan Y. H. Yuan X. B. (2022). Association of CDH11 with autism spectrum disorder revealed by matched-gene co-expression analysis and mouse behavioral studies. Neurosci. Bull. 38, 29–46. 10.1007/s12264-021-00770-0 34523068 PMC8783018

[B264] Wu Q. J. Sun S. Y. Yan C. J. Cheng Z. C. Yang M. F. Li Z. F.. (2017). EXOC3L2 rs597668 variant contributes to Alzheimer's disease susceptibility in Asian population. Oncotarget 8, 20086–20091. 10.18632/oncotarget.15380 28423615 PMC5386745

[B265] Xiang S. Huang Z. Wang T. Han Z. Yu C. Y. Ni D.. (2018). Condition-specific gene co-expression network mining identifies key pathways and regulators in the brain tissue of Alzheimer's disease patients. BMC Med. Genom. 11:115. 10.1186/s12920-018-0431-1 30598117 PMC6311927

[B266] Xie S. Yang J. Huang S. Fan Y. Xu T. He J.. (2022). Disrupted myelination network in the cingulate cortex of Parkinson's disease. IET Syst. Biol. 16, 98–119. 10.1049/syb2.12043 35394697 PMC9290774

[B267] Xu C. Chang T. Du Y. Yu C. Tan X. Pharmacokinetics L.. (2019). of oral and intravenous cannabidiol and its antidepressant-like effects in chronic mild stress mouse model. Environ. Toxicol. Pharmacol. 70:103202. 10.1016/j.etap.2019.103202 31173966

[B268] Xu Y. Kong J. Hu P. (2021). Computational drug repurposing for Alzheimer's disease using risk genes from GWAS and single-cell RNA sequencing studies. Front. Pharmacol. 12:617537. 10.3389/fphar.2021.617537 34276354 PMC8277916

[B269] Yaghoobi A. Malekpour S. A. (2024). Unraveling the genetic architecture of blood unfolded p-53 among non-demented elderlies: novel candidate genes for early Alzheimer's disease. BMC Genom. 25:440. 10.1186/s12864-024-10363-6 38702606 PMC11067101

[B270] Yan Y. Wang J. Yu L. Cui B. Wang H. Xiao X.. (2021). ANKRD36 is involved in hypertension by altering expression of ENaC genes. Circ. Res. 129, 1067–1081. 10.1161/CIRCRESAHA.121.319883 34615377

[B271] Yang Z. Jun H. Choi C. I. Yoo K. H. Cho C. H. Hussaini S. M. Q.. (2017). Age-related decline in BubR1 impairs adult hippocampal neurogenesis. Aging Cell 16, 598–601. 10.1111/acel.12594 28383136 PMC5418205

[B272] Yu H. Wang F. Wu J. J. Gong J. Bi S. Mao Y.. (2023). Integrated transcriptomics reveals the brain and blood biomarkers in Alzheimer's disease. CNS Neurosci. Ther. 29, 3943–3951. 10.1111/cns.14316 37334737 PMC10651972

[B273] Yu L. Tasaki S. Schneider J. A. Arfanakis K. Duong D. M. Wingo A. P.. (2020). Cortical proteins associated with cognitive resilience in community-dwelling older persons. JAMA Psychiatry 77, 1172–1180. 10.1001/jamapsychiatry.2020.1807 32609320 PMC7330835

[B274] Yu Y. Pan J. Liu M. Jiang H. Xiong J. Tao L.. (2022). Guanylate-Binding protein 2b regulates the AMPK/mTOR/ULK1 signalling pathway to induce autophagy during Mycobacterium bovis infection. Virulence 13, 875–889. 10.1080/21505594.2022.2073024 35531887 PMC9132469

[B275] Yuan J. Liu X. Liu C. Ang A. F. Massaro J. Devine S. A.. (2022). Is dietary choline intake related to dementia and Alzheimer's disease risks? Results from the Framingham Heart Study. Am. J. Clin. Nutr. 116, 1201–1207. 10.1093/ajcn/nqac193 37208066 PMC9630864

[B276] Zamolodchikov D. Chen Z. L. Conti B. A. Renne T. Strickland S. (2015). Activation of the factor XII-driven contact system in Alzheimer's disease patient and mouse model plasma. Proc. Natl. Acad. Sci. U.S.A. 112, 4068–4073. 10.1073/pnas.1423764112 25775543 PMC4386355

[B277] Zhang Q. Ma C. Chin L. S. Pan S. Li L. (2024). Human brain glycoform coregulation network and glycan modification alterations in Alzheimer's disease. Sci. Adv. 10:eadk6911. 10.1126/sciadv.adk6911 38579000 PMC10997212

[B278] Zhang X. Wu X. Peng J. Sun A. Guo Y. Fu P.. (2022). Cis- and trans-regulation by histone H4 basic patch R17/R19 in metazoan development. Open Biol. 12:220066. 10.1098/rsob.220066 36382370 PMC9667139

[B279] Zhao S. Feng X. F. Huang T. Luo H. H. Chen J. X. Zeng J.. (2020). The association between acylcarnitine metabolites and cardiovascular disease in Chinese patients with type 2 diabetes mellitus. Front. Endocrinol. 11:212. 10.3389/fendo.2020.00212 32431666 PMC7214635

[B280] Zhou M. Jiao Q. Wu Z. Li W. Liu G. Wang R.. (2024). Uncovering the oxidative stress mechanisms and targets in Alzheimer's disease by integrating phenotypic screening data and polypharmacology networks. J. Alzheimers Dis. 99, S139–S56. 10.3233/JAD-220727 36744334

[B281] Zhu H. Meissner L. E. Byrnes C. Tuymetova G. Tifft C. J. Proia R. L. (2020). The complement regulator susd4 influences nervous-system function and neuronal morphology in mice. iScience 23:100957. 10.1016/j.isci.2020.100957 32179479 PMC7075988

[B282] Zhu W. M. Neuhaus A. Beard D. J. Sutherland B. A. DeLuca G. C. (2022). Neurovascular coupling mechanisms in health and neurovascular uncoupling in Alzheimer's disease. Brain 145, 2276–2292. 10.1093/brain/awac174 35551356 PMC9337814

[B283] Zhuang X. Xia Y. Liu Y. Guo T. Xia Z. Wang Z.. (2024). SCG5 and MITF may be novel markers of copper metabolism immunorelevance in Alzheimer's disease. Sci. Rep. 14:13619. 10.1038/s41598-024-64599-z 38871989 PMC11176367

