# Supplementary material for: Molecular pathogenesis of Alzheimer's disease onset in a mouse model: effects of cannabidiol treatment
Source: Front Neurosci. 2025 Sep 5;19:1667585. doi: 10.3389/fnins.2025.1667585 (PMC12446314; doi:10.3389/fnins.2025.1667585)
Supplement: Supplementary file 1 [file Data_Sheet_1.pdf]

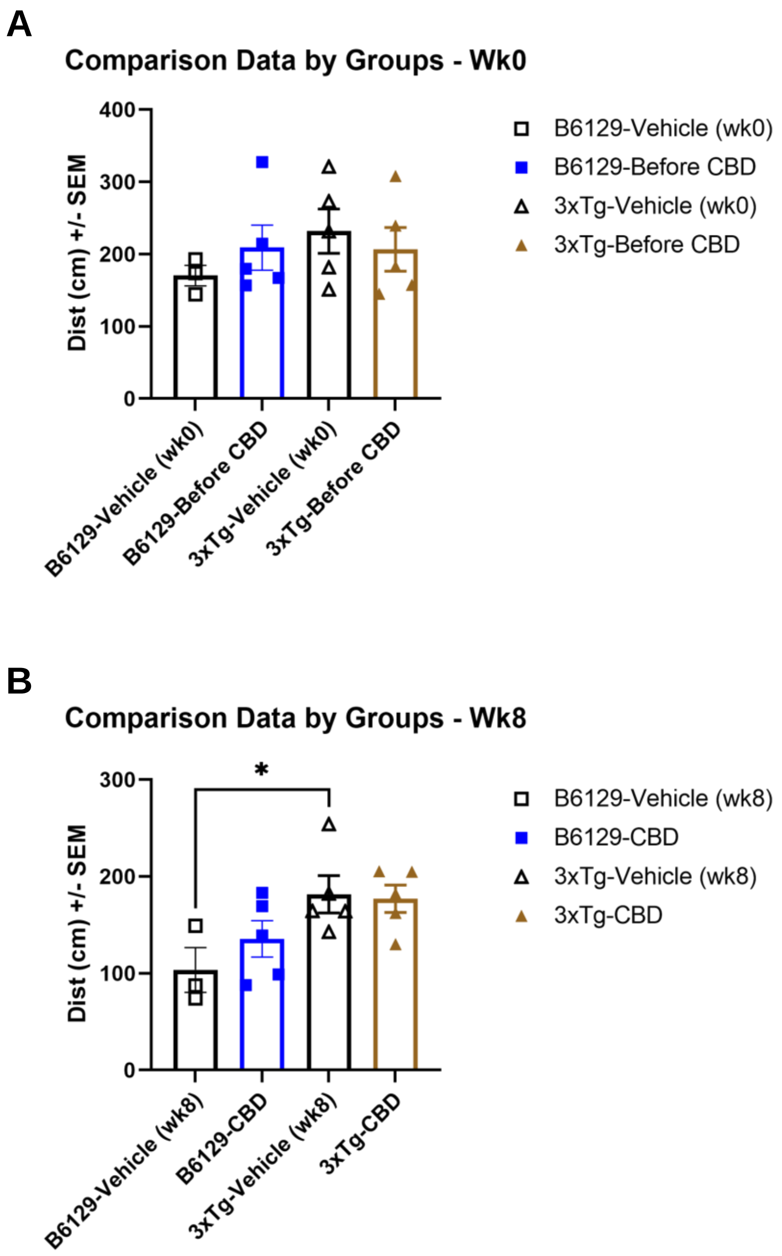
A
Comparison Data by Groups - Wk0
Dist (cm) +/- SEM
400
300
200
100
0
B6129-Vehicle (wk0)
B6129-Before CBD
3xTg-Vehicle (wk0)
3xTg-Before CBD
B
Comparison Data by Groups - Wk8
Dist (cm) +/- SEM
300
200
100
0
*
B6129-Vehicle (wk8)
B6129-CBD
3xTg-Vehicle (wk8)
3xTg-CBD

Figure 1

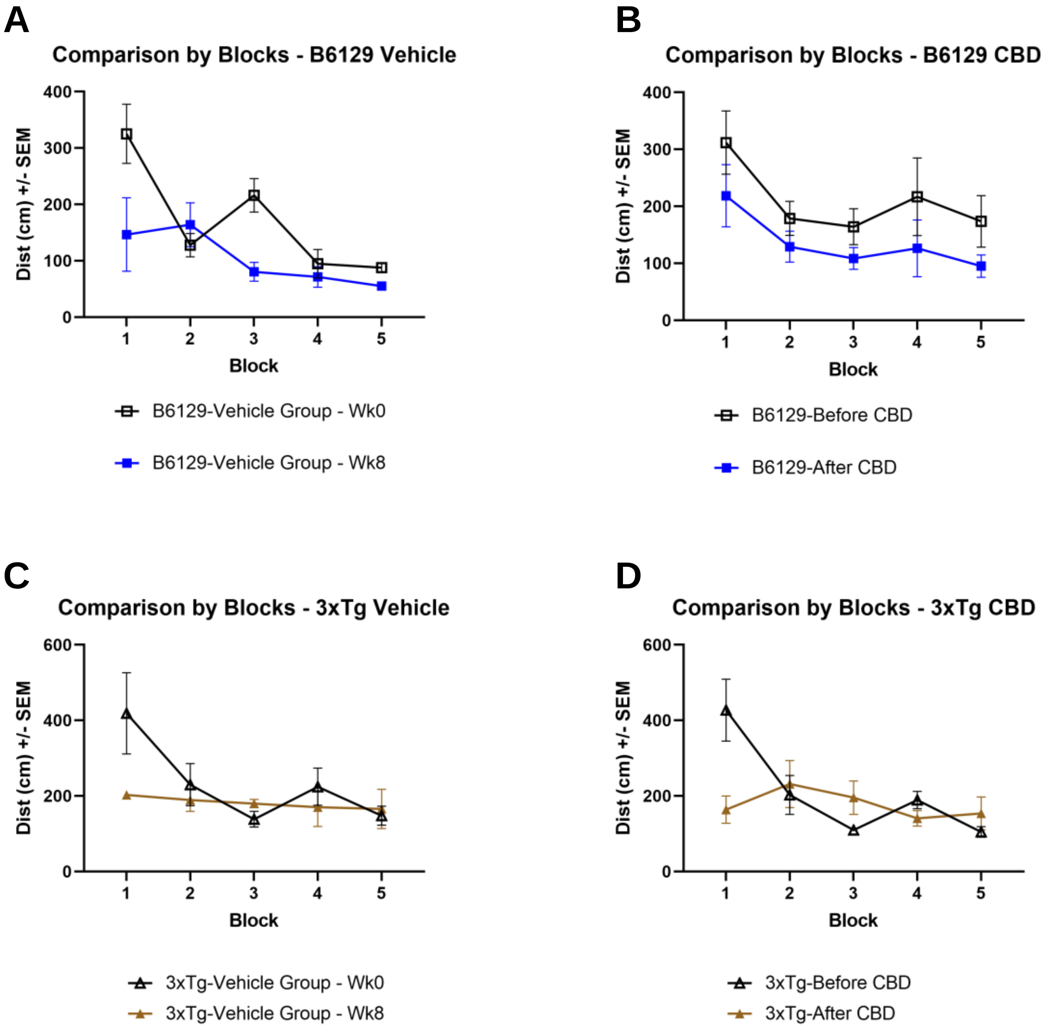
A
Comparison by Blocks - B6129 Vehicle
Dist (cm) +/- SEM
Block
B6129-Vehicle Group - Wk0
B6129-Vehicle Group - Wk8
B
Comparison by Blocks - B6129 CBD
Dist (cm) +/- SEM
Block
B6129-Before CBD
B6129-After CBD
C
Comparison by Blocks - 3xTg Vehicle
Dist (cm) +/- SEM
Block
3xTg-Vehicle Group - Wk0
3xTg-Vehicle Group - Wk8
D
Comparison by Blocks - 3xTg CBD
Dist (cm) +/- SEM
Block
3xTg-Before CBD
3xTg-After CBD

Figure 2

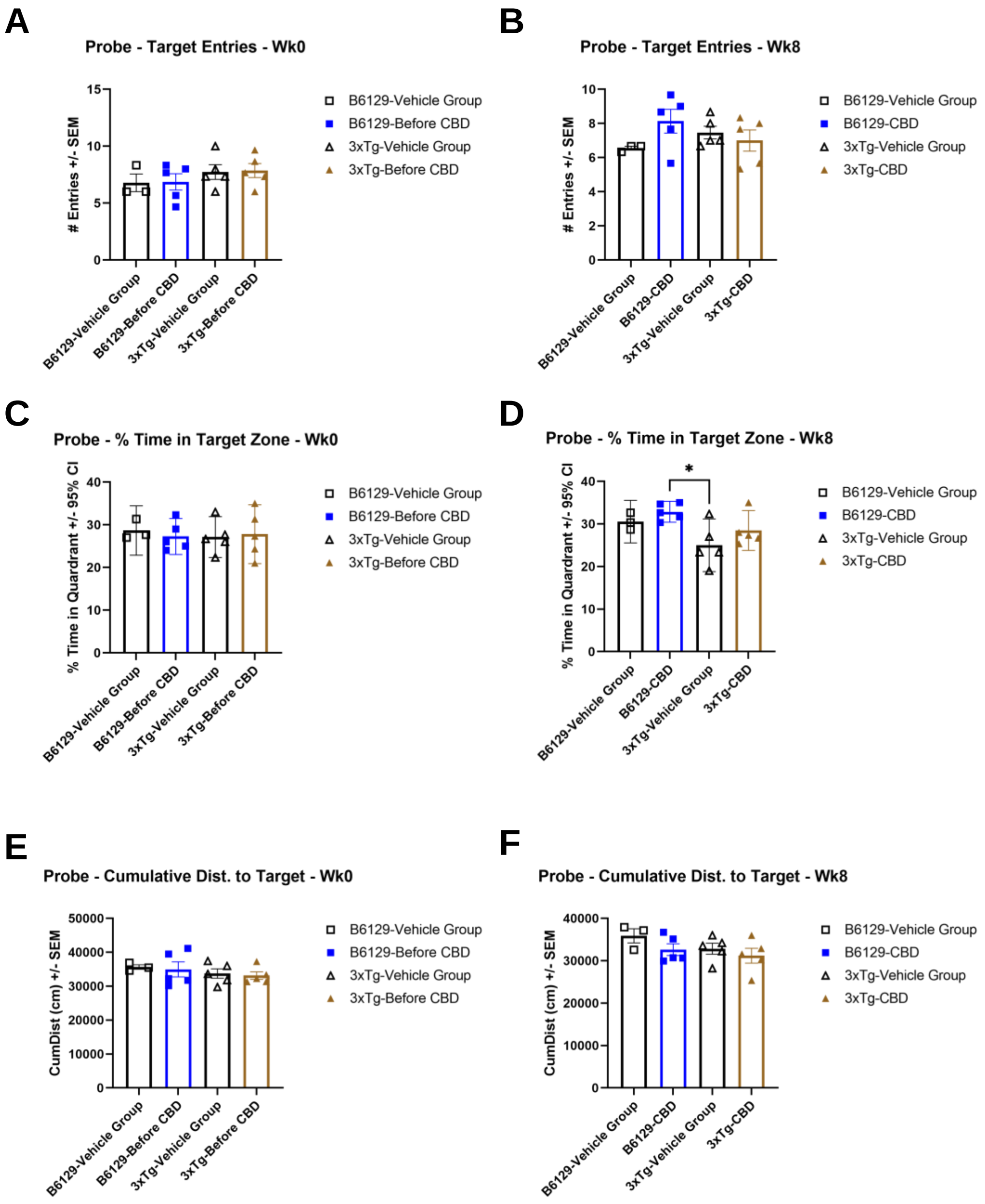
A
Probe - Target Entries - Wk0
Entries +/- SEM
0
5
10
15
B6129-Vehicle Group
B6129-Before CBD
3xTg-Vehicle Group
3xTg-Before CBD
B
Probe - Target Entries - Wk8
Entries +/- SEM
0
2
4
6
8
10
B6129-Vehicle Group
B6129-CBD
3xTg-Vehicle Group
3xTg-CBD
C
Probe - % Time in Target Zone - Wk0
% Time in Quardrant +/- 95% CI
0
10
20
30
40
B6129-Vehicle Group
B6129-Before CBD
3xTg-Vehicle Group
3xTg-Before CBD
D
Probe - % Time in Target Zone - Wk8
*
% Time in Quardrant +/- 95% CI
0
10
20
30
40
B6129-Vehicle Group
B6129-CBD
3xTg-Vehicle Group
3xTg-CBD
E
Probe - Cumulative Dist. to Target - Wk0
CumDist (cm) +/- SEM
0
10000
20000
30000
40000
50000
B6129-Vehicle Group
B6129-Before CBD
3xTg-Vehicle Group
3xTg-Before CBD
F
Probe - Cumulative Dist. to Target - Wk8
CumDist (cm) +/- SEM
0
10000
20000
30000
40000
B6129-Vehicle Group
B6129-CBD
3xTg-Vehicle Group
3xTg-CBD

Figure 3

A

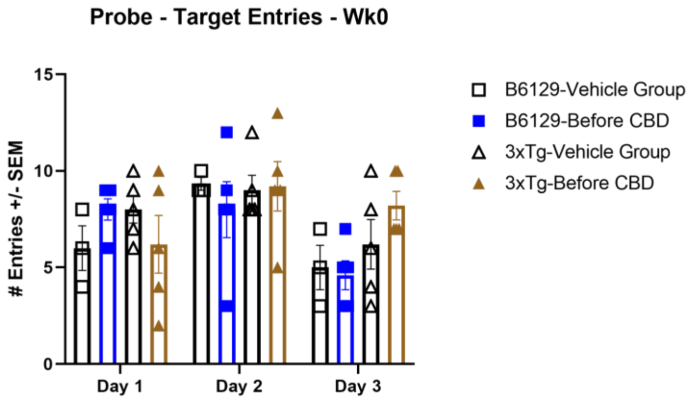

B

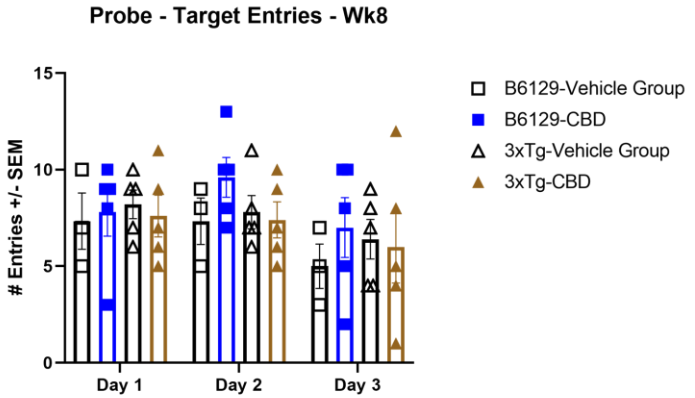

C

Group	# of Target Entries Fraction Average of Day 1-3 (Wk8 vs Wk0)
B6-Veh	1.07
B6-CBD	1.31
3x-Veh	1.05
3x-CBD	1.08

Figure 4

A

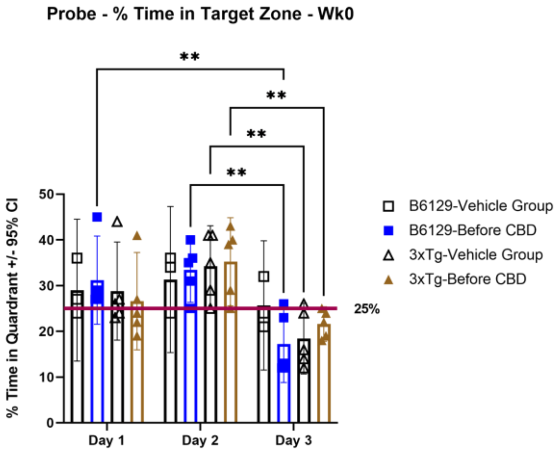

B

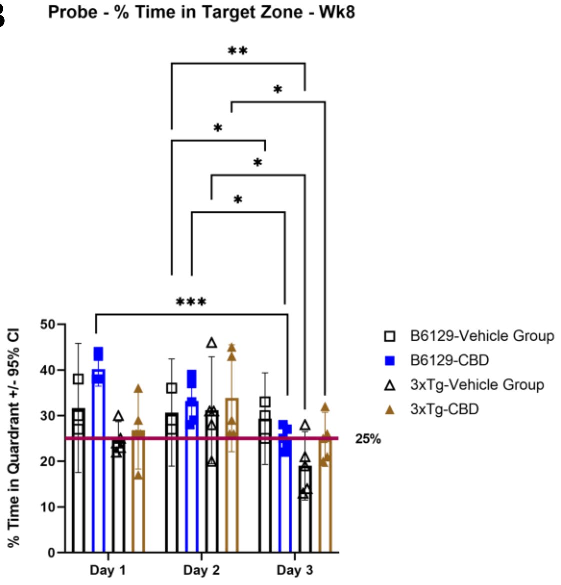

C

Group	% Time Fraction Average of Day 1-3 (Wk8 vs Wk0)
B6-Veh	1.09
B6-CBD	1.31
3x-Veh	1.01
3x-CBD	1.09

Figure 5

A

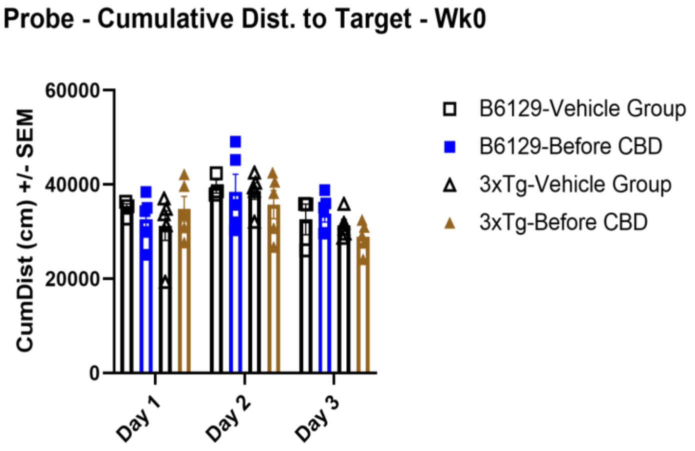

B

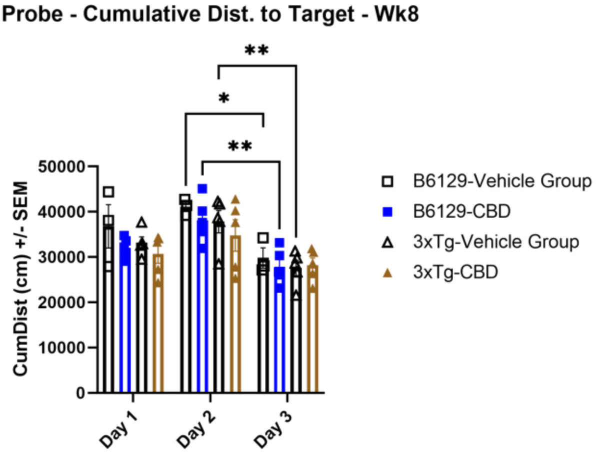

C

**Group**	**CumDist Fraction Average of Day 1-3 (Wk8 vs Wk0)**
B6-Veh	1.01
B6-CBD	0.94
3x-Veh	1.00
3x-CBD	0.96

Figure 6

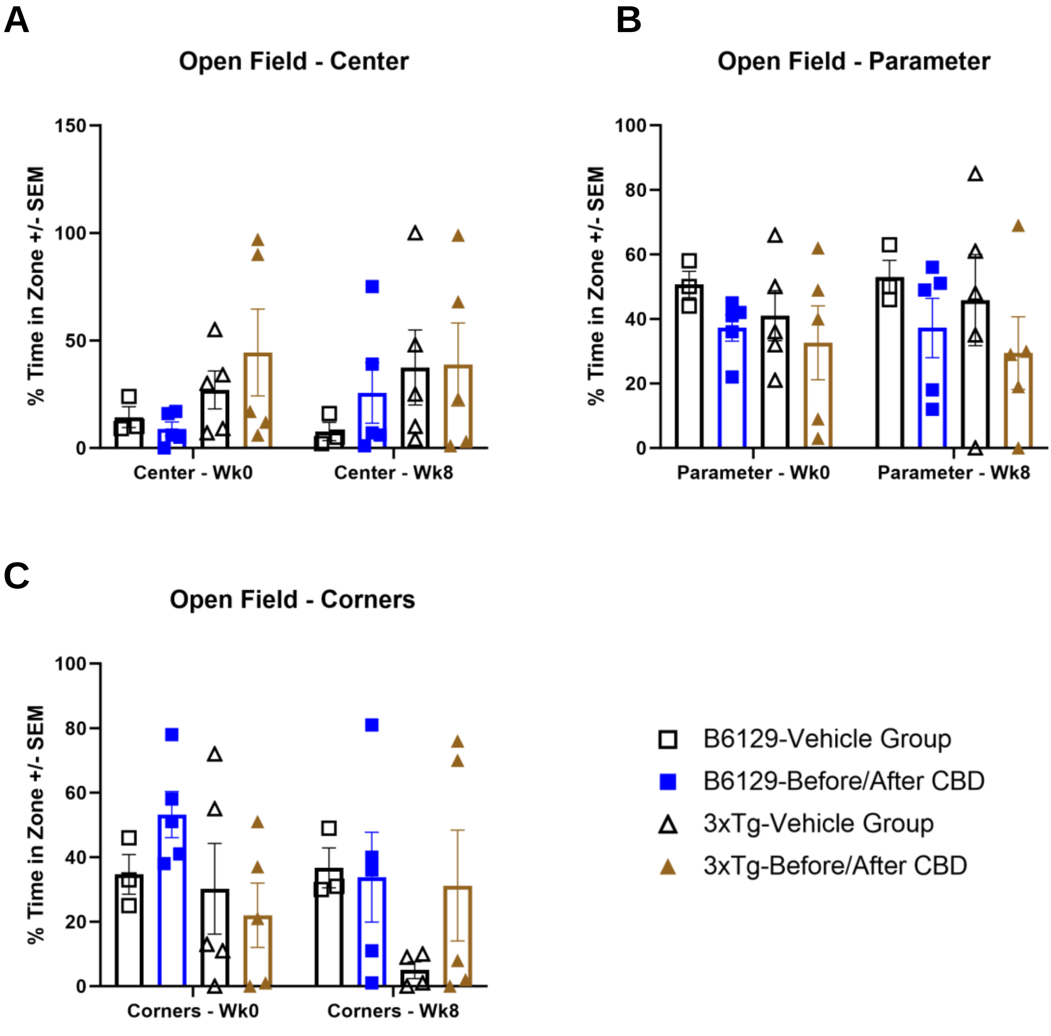
A
Open Field - Center
% Time in Zone +/- SEM
150
100
50
0
Center - Wk0
Center - Wk8
B
Open Field - Parameter
% Time in Zone +/- SEM
100
80
60
40
20
0
Parameter - Wk0
Parameter - Wk8
C
Open Field - Corners
% Time in Zone +/- SEM
100
80
60
40
20
0
Corners - Wk0
Corners - Wk8
B6129-Vehicle Group
B6129-Before/After CBD
3xTg-Vehicle Group
3xTg-Before/After CBD

Figure 7

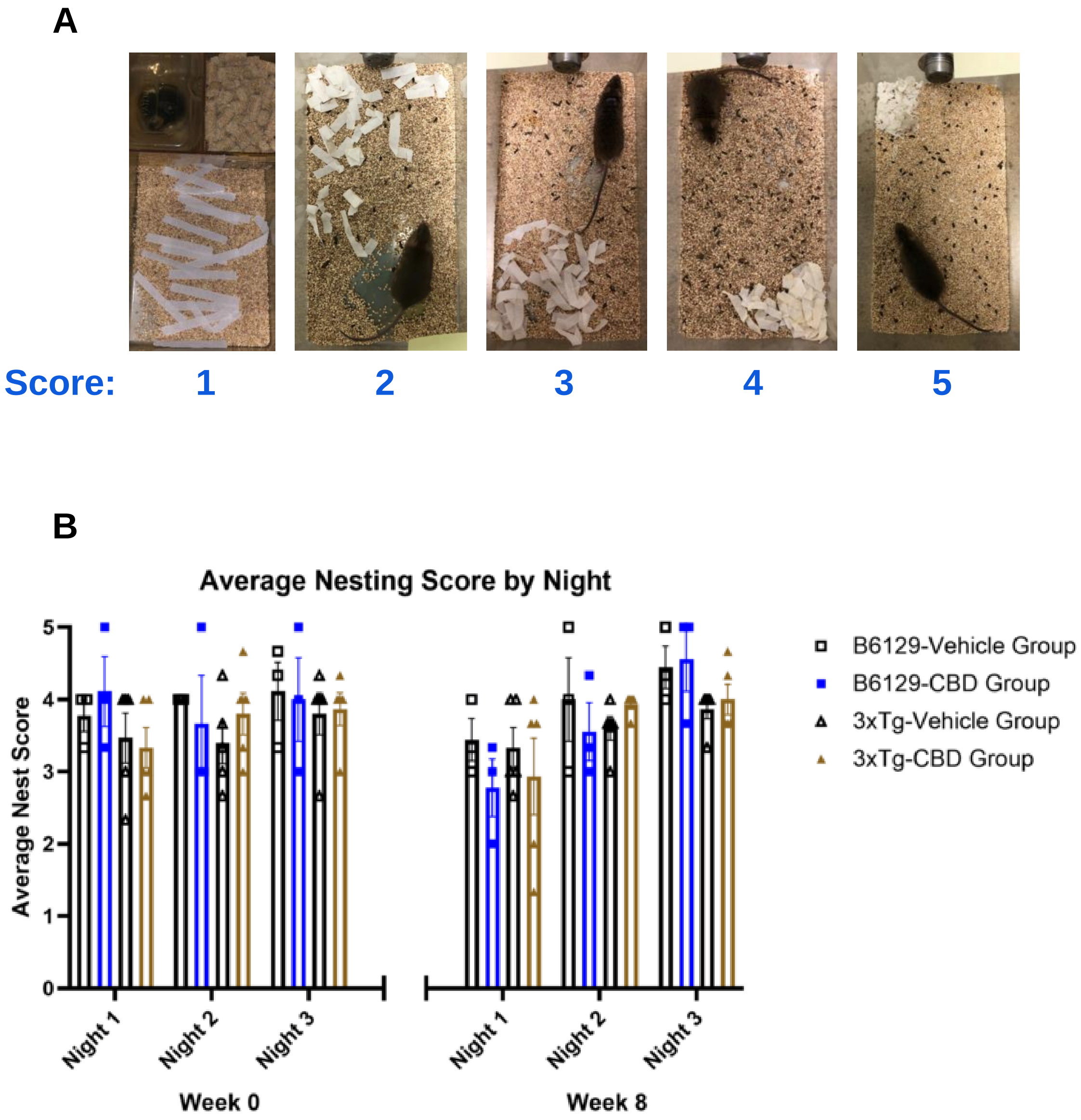
A
Score:
1
2
3
4
5
B
Average Nesting Score by Night
Average Nest Score
0
1
2
3
4
5
Night 1
Night 2
Night 3
Week 0
Night 1
Night 2
Night 3
Week 8
B6129-Vehicle Group
B6129-CBD Group
3xTg-Vehicle Group
3xTg-CBD Group

Figure 8
