# Supplementary material for: Molecular pathogenesis of Alzheimer's disease onset in a mouse model: effects of cannabidiol treatment
Source: Front Neurosci. 2025 Sep 5;19:1667585. doi: 10.3389/fnins.2025.1667585 (PMC12446314; doi:10.3389/fnins.2025.1667585)
Supplement: Supplementary file 2 [file Data_Sheet_2.pdf]

Supplemental Figure Legends

Supplemental Figure 1. ***Average distance traveled in the water maze among wild-type and Alzheimer's disease animals with and without cannabidiol treatment.*** (A) Average distance traveled at 0 wks [4.5 mo old, no cannabidiol (CBD) treatment yet] among study groups. (B) As in A, at 8 wks (6.5 mo old) following CBD vs vehicle treatment period. Note a significant increase in increased distance traveled in *3xTg-AD* vs. wild-type B6129 mice with negligible effects of CBD. Data were obtained from n = 3 to 5 mice per group.

Supplemental Figure 2. ***Individual block distance traveled in the water maze among wild-type and Alzheimer's disease animals with and without cannabidiol treatment.*** (A) Individual distance traveled in the wild-type B6129 vehicle group throughout Blocks 1 to 5 at 0 wks (4.5 mo old) and 8 wks (6.5 mo old). (B) As in A, but with wild-type B6129 animals treated with CBD. (C & D) As in A & B respectively, but with *3xTg-AD* mice. Note that, overall, all groups performed better at wk 8 relative to the starting point at wk 0. Further, at wk 8, the *3xTg-AD* animals did not express the same learning pattern relative to wild-type B6129 animals as marked by an apparent motivation to escape the body of water as soon as possible. Thus, a reduced traveling distance is shown for the *3xTg-AD* mice during Block 1 in particular. Data were obtained from n = 3 to 5 mice per group.

Supplemental Figure 3. ***Averaged probe characteristics of the water maze among wild-type and Alzheimer's disease animals with and without cannabidiol treatment.*** (A) Number of target entries at 0 wks [4.5 mo old, no cannabidiol (CBD) treatment yet] among study groups. (B) As in

A, but at 8 wks following CBD vs. vehicle treatment. (C & D) As in A & B respectively, but with the percentage of time spent at target zone. (E & F) As in A & B respectively, but with cumulative distance to target. A favorable learning profile is indicated by a higher number of target entries, >25% of time in the target zone, and lesser cumulative distance to the target zone. Note that the mean of *3xTg-AD* vehicle animals approximated the 25% threshold value for time in the target zone with three out of the five animals performing < 25%. Following CBD treatment, four out of five *3xTgAD* animals performed >25%. All wild-type B6129 animals performed >25% regardless of CBD treatment. Data were obtained from n = 3 to 5 mice per group; *P<0.05.

Supplemental Figure 4. ***Number of target entries over three days in the water maze among wild-type and Alzheimer's disease animals with and without cannabidiol treatment.*** (A) Number of target entries at 0 wks [4.5 mo old, no cannabidiol (CBD) treatment yet] among study groups over three days. (B) As in A, but at 8 wks following CBD vs. vehicle treatment. (C) Average ratio of number of target entries for wk 8 versus wk 0 among groups. Note that, overall, CBD treatment increased the average number of target entries by ≈22% and ≈3% in wild-type B6129 and *3xTg-AD* mice respectively. Data were obtained from n = 3 to 5 mice per group.

Supplemental Figure 5. ***Percentage of time spent in the target zone over three days in the water maze among wild-type and Alzheimer's disease animals with and without cannabidiol treatment.*** (A) Percentage of time spent in the target zone at 0 wks [4.5 mo old, no cannabidiol (CBD) treatment yet] among study groups over three days. (B) As in A, but at 8 wks following CBD vs. vehicle treatment. (C) Average ratio of percentage of time spent at target zone for wk 8 versus wk 0 among groups. With a general "rundown" in performance among groups from Days 1 & 2 to

Day 3, the *3xTg-AD* group performed < 25% during Day 3 at wk 0 with a mild improvement to the ≈25% threshold value at wk 8 following CBD treatment. Note that, overall, CBD treatment increased the average time spent in the target zone by ≈20% and ≈8% in wild-type B6129 and *3xTg-AD* mice respectively. Data were obtained from n = 3 to 5 mice per group; *P<0.05, **P<0.01, ***P<0.001.

Supplemental Figure 6. ***Cumulative distance traveled to the target zone over three days in the water maze among wild-type and Alzheimer's disease animals with and without cannabidiol treatment.*** (A) Cumulative distance traveled to the target zone at 0 wks [4.5 mo old, no cannabidiol (CBD) treatment yet] among study groups over three days. (B) As in A, but at 8 wks following CBD vs. vehicle treatment. (C) Average ratio of cumulative distance traveled to the target zone for wk 8 versus wk 0 among groups. A significant decrease in distance values was noted from Day 2 to Day 3 of all groups except for CBD-treated *3xTgAD* animals at wk 8. Note that, overall, CBD treatment decreased the average cumulative distance by ≈7% and ≈4% in wild-type B6129 and *3xTg-AD* mice respectively. Data were obtained from n = 3 to 5 mice per group; *P<0.05, **P<0.01.

Supplemental Figure 7. ***Time spent in the center, parameter, and corners in the open field test among wild-type and Alzheimer's disease animals with and without cannabidiol treatment.*** (A) Percent of time spent in the center among all respective study groups at wk 0 (4.5 mo old, before cannabidiol (CBD) treatment] and wk 8 (6.5 mo old, following CBD vs. vehicle treatment). (B) As in A, for the parameter. (C) As in A for the corners. Data were obtained from n = 3 to 5 mice per group.

Supplemental Figure 8. ***Nesting behavior among wild-type and Alzheimer's disease animals with and without cannabidiol treatment.*** (A) Representative images illustrating nesting profile scores from 1 (=worst, poorly organized) to 5 (=best, highly organized). (B) Average nesting scores across individual nights examined for all respective study groups at wk 0 (4.5 mo old, before cannabidiol (CBD) treatment] and wk 8 (6.5 mo old, following CBD vs. vehicle treatment). Although not statistically significant, note a lower nesting score among *3xTg-AD* animals with a negligible effect of CBD. Data were obtained from n = 3 to 5 mice per group.
