# Supplementary material for: Molecular pathogenesis of Alzheimer's disease onset in a mouse model: effects of cannabidiol treatment
Source: Front Neurosci. 2025 Sep 5;19:1667585. doi: 10.3389/fnins.2025.1667585 (PMC12446314; doi:10.3389/fnins.2025.1667585)
Supplement: Supplementary file 3 [file Data_Sheet_3.pdf]

Table of Contents

Supplemental Table 1. 3
Supplemental Table 2. 27
Supplemental Table 3. 45
Supplemental Table 4. 47
Supplemental Table 5. 58
Supplemental Table 6. 70
Supplemental Table 7. 89
Supplemental Table 8. 94
Supplemental Table 9. 101
Supplemental Table 10. 113
Supplemental Table 11. 116
Supplemental Table 12. 119
Supplemental Table 13. 125
Supplemental Table 14. 134
Supplemental Table 15. 141
Supplemental Table 16. 142
Supplemental Table 17. 143
Supplemental Table 18. 144
Supplemental Table 19. 145
Supplemental Table 20. 146
Supplemental Table 21. 147
Supplemental Table 22. 184
Supplemental Table 23. 201
Supplemental Table 24. 214
Supplemental Table 25. 265
Supplemental Table 26. 278
Supplemental Table 27. 281
Supplemental Table 28. 288
Supplemental Table 29. 289
Supplemental Table 30. 292
Supplemental Table 31. 293
Supplemental Table 32. 313
Supplemental Table 33. 316

Supplemental Table 34. 320

Supplemental Table 1. List of all significant (P < 0.05) differentially expressed genes in whole blood of 3xTg-AD vehicle (week 8) versus 3xTg-AD vehicle (week 0) animals; 447 genes downregulated & 471 genes upregulated. Italicized genes have a recognized association with Alzheimer's disease pathology; #unknown identity/role

Gene Name	Log2FoldChange	P-value	Regulation
H2-Q2	-16.7756034	3.64E-24	Downregulated
Tdrd5	-15.9992625	3.96E-18	Downregulated
Gm42866#	-4.94768186	1.20E-05	Downregulated
Gm37607#	-4.64510456	2.69E-05	Downregulated
Gm28941; lncRNA	-4.50541624	0.00033357	Downregulated
Snora41; snoRNA	-4.48117105	0.00042263	Downregulated
Gm19552#	-4.44538324	0.00165102	Downregulated
Gm44834#	-4.37977713	0.00147299	Downregulated
Zfp951	-4.349788	0.00120878	Downregulated
ENSMUSG00002075672 Gm54761; miRNA	-4.31484818	0.00138943	Downregulated
Gm9754; lncRNA	-4.28409245	0.00060642	Downregulated
BC016579	-4.26504701	0.00184264	Downregulated
Gm38146#	-4.25213641	0.00084838	Downregulated
3110080O07Rik; uncharacterized protein LOC73241	-4.25007361	0.00065027	Downregulated
Sema4c	-4.24253536	0.00040565	Downregulated
Rnf227	-4.2346848	0.00014041	Downregulated
Trbj1-7	-4.20364531	0.02770301	Downregulated
2510017J16Rik; uncharacterized protein LOC66574	-4.19734243	0.00036031	Downregulated
Gm5521; pseudogene, mortality factor 4 like 2	-4.18094398	0.01084889	Downregulated
Gm43707#; protein-coding	-4.17704818	0.00196176	Downregulated
Gm19046; pseudogene, non-POU-domain-containing, octamer binding protein	-4.11398053	0.00645746	Downregulated
1600022D10Rik; lncRNA	-4.10301518	0.01728084	Downregulated
Gm49703#	-4.0415279	0.00086937	Downregulated
Mir342; miRNA	-4.0283531	0.01029355	Downregulated
Gm17096; GM17096 gene product from transcript GM17096-RA	-4.02812035	0.00172693	Downregulated
Gm47594#	-4.02171308	0.00825084	Downregulated
Trbj1-6	-4.01590509	0.015917	Downregulated
Gm44975#	-3.97628417	0.00198411	Downregulated

Gm44704#	-3.96254663	0.00115684	Downregulated
Gm19427; pseudogene, 60S ribosomal protein L38	-3.9545539	0.00508826	Downregulated
Gm8899; pseudogene	-3.93006459	0.02554252	Downregulated
Gm7054; pseudogene, establishment of cohesion 1 homolog 1	-3.92935023	0.01337869	Downregulated
Gm37766#	-3.92394797	0.00346037	Downregulated
Gm29156; lncRNA	-3.90760215	0.0015669	Downregulated
Mir670hg; miRNA	-3.90654666	0.00183108	Downregulated
Pcdhgb7	-3.8869869	0.00062713	Downregulated
Gm42568#	-3.86404065	0.00138184	Downregulated
Gm43609#	-3.83570222	0.00178935	Downregulated
4833412K13Rik; uncharacterized protein LOC74607	-3.82078958	0.00143695	Downregulated
2900078I11Rik; uncharacterized protein LOC73004	-3.81891065	0.00169833	Downregulated
Gm49706#	-3.81756573	0.00183807	Downregulated
Gm37675#	-3.81500475	0.00423674	Downregulated
Traj22	-3.79770035	0.04778866	Downregulated
Gm45570#	-3.79737735	0.00345367	Downregulated
Gm24992; snoRNA	-3.79084576	0.00834251	Downregulated
Gm44174; lncRNA	-3.76189327	0.02202438	Downregulated
Gm11725; lncRNA	-3.7591443	0.00547631	Downregulated
Gm26064; snRNA	-3.7582433	0.00388827	Downregulated
Gm29112#	-3.7569683	0.0052204	Downregulated
Gm37062#	-3.74175131	0.0020335	Downregulated
Gm38252#	-3.73626651	0.00660428	Downregulated
Gm49169#	-3.72678365	0.00889314	Downregulated
Gm43513#	-3.71943912	0.00419938	Downregulated
Gm38257#	-3.71914662	0.00178216	Downregulated
A3galt2	-3.71316128	0.00447512	Downregulated
Gm29055; lncRNA	-3.70706594	0.00786498	Downregulated
Gm16113; lncRNA	-3.70495018	0.01225259	Downregulated
Gm44888#	-3.70161402	0.00221414	Downregulated
2610028D06Rik; uncharacterized protein LOC76359	-3.70150566	0.00964542	Downregulated
Tamalin; also GRASP	-3.68979254	0.00151291	Downregulated
Gm37509#	-3.6829141	0.01011844	Downregulated
Gm48335#	-3.68180246	0.00195167	Downregulated
Gm16794; lncRNA	-3.67638032	0.00646959	Downregulated
Gstp-ps; pseudogene, glutathione S-transferase, pi	-3.66876442	0.01663692	Downregulated

Gm29596; pseudogene	-3.66095139	0.01999903	Downregulated
Gm43062; lncRNA	-3.66042521	0.00319684	Downregulated
Gm25128; snoRNA	-3.64631241	0.03089848	Downregulated
Gm37080[#]	-3.64475249	0.00647639	Downregulated
Gm6044; pseudogene, peptidylprolyl isomerase A	-3.63740715	0.00951724	Downregulated
Gm27184; lncRNA	-3.6326755	0.00942412	Downregulated
Peg3	-3.62871752	0.00170206	Downregulated
Amt	-3.61618667	0.00149381	Downregulated
Gm11440[#]	-3.61018256	0.00524207	Downregulated
Gm8437[#]	-3.60718739	0.00601979	Downregulated
Gm42702; lncRNA	-3.60311345	0.00716119	Downregulated
Gm37160[#]	-3.60259912	0.00503278	Downregulated
Gm43075[#]	-3.59934321	0.01087383	Downregulated
Gm37672[#]	-3.59632094	0.00515536	Downregulated
Gm47374[#]	-3.59397818	0.01767785	Downregulated
Mir7212; miRNA	-3.58695602	0.01692628	Downregulated
Ccdc106	-3.57576582	0.00990544	Downregulated
Gm45407; lncRNA	-3.5751183	0.00510212	Downregulated
Gm17981; pseudogene	-3.57378282	0.0075524	Downregulated
F12	-3.5713001	0.01644385	Downregulated
Tchh	-3.5577284	0.02674963	Downregulated
Gm14286; lncRNA	-3.54309328	0.00860356	Downregulated
Gm37519[#]	-3.53498716	0.00050259	Downregulated
Gm17072; pseudogene, voltage-dependent anion channel 1	-3.53077388	0.01001017	Downregulated
Syt15	-3.52800291	0.04082895	Downregulated
3110062G12Rik; uncharacterized protein LOC73188	-3.51569936	0.00776598	Downregulated
Gm37490[#]	-3.51507927	0.02294849	Downregulated
Gm13710; lncRNA	-3.51180277	0.04633139	Downregulated
Gm12902 or Agpat2; 1-Acylglycerol-3-phosphate O-acyltransferase 2	-3.51169318	0.01883749	Downregulated
Stk36	-3.51127562	0.00959452	Downregulated
Gm15859; lncRNA	-3.50785567	0.00704066	Downregulated
Gm28053[#]; protein-coding	-3.50351444	0.03371813	Downregulated
Tmem150b	-3.49120092	0.03879828	Downregulated
1700028J19Rik; lncRNA	-3.49057243	0.01395799	Downregulated
1810012K16Rik; lncRNA	-3.48720857	0.02016887	Downregulated
Kcnip3; or Calsenilin	-3.48698629	0.00302711	Downregulated
Rasd1	-3.48216278	0.00459423	Downregulated
Synm	-3.47875815	0.0025495	Downregulated

D130019J16Rik; lncRNA	-3.47473456	0.01362365	Downregulated
Gm14830; pseudogene, proteasome subunit beta type-1	-3.46947222	0.03120912	Downregulated
Sox7	-3.44469164	0.02035518	Downregulated
Gm15708; lncRNA	-3.43855823	0.01123739	Downregulated
4930579K19Rik; lncRNA	-3.42021764	0.01004634	Downregulated
Gm37342[#]	-3.4140781	0.00114642	Downregulated
Olfr1393	-3.41338819	0.01527775	Downregulated
Fam171a1	-3.40997151	0.00379768	Downregulated
Pbx4	-3.40968696	0.00622042	Downregulated
Stmn1-rs1; pseudogene, stathmin 1, related sequence 1	-3.40849698	0.02166136	Downregulated
Angptl6	-3.40800968	0.00523055	Downregulated
Polr2k-ps; pseudogene, polymerase (RNA) II (DNA directed) polypeptide K	-3.39167827	0.02670449	Downregulated
Ppnr	-3.3897507	0.00817668	Downregulated
Hspa12b	-3.37545292	0.01531904	Downregulated
Gm15549; pseudogene, Ribosomal protein 10	-3.37415645	0.0297595	Downregulated
Gm26109; snoRNA	-3.3552507	0.01386469	Downregulated
Iqck	-3.35299091	0.01020175	Downregulated
Gm44835[#]	-3.34955373	0.02642691	Downregulated
Gm4787; Adam4b or EG214321	-3.32271458	0.00675383	Downregulated
Gm43024[#]	-3.31913912	0.00341917	Downregulated
Gm44037[#]	-3.31844977	0.02339961	Downregulated
Tagln3	-3.31669466	0.00539579	Downregulated
Crmp1	-3.31600977	0.02998959	Downregulated
Gm49086; lncRNA	-3.31478589	0.01786362	Downregulated
Mir421; miRNA	-3.3123907	0.00747025	Downregulated
Gm14049; pseudogene, high mobility group box 1	-3.30968435	0.0185243	Downregulated
Gm38125[#]	-3.28629307	0.01011514	Downregulated
Gm42632; lncRNA	-3.28054054	0.03899295	Downregulated
Gm17228; pseudogene	-3.27592265	0.01541891	Downregulated
Gm43215[#]	-3.2599839	0.03324644	Downregulated
Gm44008; lncRNA	-3.25786421	0.02170605	Downregulated
Gm47113; lncRNA	-3.25105466	0.02162417	Downregulated
Gm8738; pseudogene, CDGSH iron-sulfur domain-containing protein 1	-3.2447932	0.0247234	Downregulated
Hmgb1-ps6; pseudogene, high mobility group box 1	-3.24291838	0.01874777	Downregulated
Gm49326; pseudogene	-3.24246062	0.01586205	Downregulated

Gm38021#	-3.23937343	0.02157033	Downregulated
Fuom	-3.23725793	0.00735465	Downregulated
Gm37485#	-3.234102	0.02011113	Downregulated
Rnft2	-3.23253147	0.00630479	Downregulated
Gm18537; pseudogene, DENN/MADD domain containing 4C	-3.23213558	0.02194293	Downregulated
Gm36501; pseudogene, uncharacterized protein C1orf43 homolog	-3.22197986	0.04020865	Downregulated
Gm43420#	-3.21759884	0.01593653	Downregulated
Rin1	-3.21093224	0.01890095	Downregulated
Gm15672; lncRNA	-3.20356525	0.04025405	Downregulated
Gm17530 or LOC6618995; serine/arginine repetitive matrix protein 2	-3.1954435	0.01614702	Downregulated
Gm19269#	-3.1891713	0.01442808	Downregulated
7SK or RN7SK; snRNA, RNA component of 7SK nuclear ribonucleoprotein	-3.1872732	0.0435933	Downregulated
Gm25821; snoRNA Snora21	-3.18515155	0.00666781	Downregulated
Gm16074; pseudogene, ring finger protein 4	-3.18105401	0.03517554	Downregulated
Gm4734; pseudogene, 60S ribosomal protein L7	-3.17487966	0.04406198	Downregulated
Gm43727; lncRNA	-3.17244213	0.02565384	Downregulated
Tmem240	-3.16463879	0.00691936	Downregulated
Gm42874#	-3.16436853	0.03791596	Downregulated
A130050O07Rik; lncRNA	-3.15877975	0.03697641	Downregulated
Prss36	-3.15723052	0.00961726	Downregulated
Zkscan16	-3.13874553	0.008779	Downregulated
Smarcd3	-3.13457261	0.02082746	Downregulated
Rac3	-3.13390553	0.01137161	Downregulated
Gm15971; pseudogene	-3.13052676	0.01342614	Downregulated
Gm43072#	-3.12693392	0.01393901	Downregulated
Gm49058#	-3.11845668	0.01973029	Downregulated
D7Bwg0826e; DNA, Chr 7, Brigham & Women's Genetics 0826	-3.11081111	0.04320737	Downregulated
Gm17893; pseudogene, nucleoporin 37	-3.10443158	0.02036709	Downregulated
Gm49201#	-3.10398611	0.01799879	Downregulated
Gm7666; pseudogene, prohibitin	-3.1027247	0.00203614	Downregulated
Zfp82	-3.09226668	0.03095556	Downregulated
Gm48670#	-3.08670433	0.00844107	Downregulated

Dhrs13	-3.08381133	0.0045567	Downregulated
Runx2os1	-3.07282767	0.04191261	Downregulated
Rimkla	-3.06772987	0.01300072	Downregulated
Slc25a2	-3.0658756	0.02376778	Downregulated
9530022L04Rik; lncRNA	-3.05698593	0.01907501	Downregulated
C78859; lncRNA	-3.05478493	0.0139059	Downregulated
Prox2os; lncRNA	-3.05095358	0.01250894	Downregulated
A930007I19Rik; lncRNA	-3.03999888	0.01178034	Downregulated
Gm3081; pseudogene, adaptor-related protein complex 2, beta 1 subunit	-3.03913634	0.00261583	Downregulated
Gm50387[#]; protein-coding	-3.03684344	0.03600396	Downregulated
4930481B07Rik; lncRNA	-3.03496073	0.01386861	Downregulated
Gm15445; lncRNA	-3.03404852	0.03109521	Downregulated
Gm43389[#]	-3.03063388	0.01928211	Downregulated
Gm23608; snoRNA	-3.02947306	0.03797379	Downregulated
Mamdc2	-3.02914759	0.02773977	Downregulated
6430571L13Rik; uncharacterized protein C3orf18 homolog	-3.02625394	0.01788001	Downregulated
Gm42869[#]	-3.02159297	0.00426092	Downregulated
Gm45532[#]	-3.0180196	0.04219113	Downregulated
Ablim3	-3.01656935	0.01882789	Downregulated
Gm28707; lncRNA	-3.01294276	0.04680249	Downregulated
Wnt10a	-3.00898024	0.03503082	Downregulated
Tagln	-3.00737965	0.04216386	Downregulated
Srd5a1	-3.00439761	0.01794335	Downregulated
Gm38376[#]	-3.00181183	0.03058371	Downregulated
8430422M14Rik; uncharacterized protein LOC109319	-3.00026967	0.01632661	Downregulated
Mir6374; miRNA	-2.98444967	0.04436022	Downregulated
Mmp28	-2.97845889	0.02145725	Downregulated
5S_rRNA	-2.97754595	0.02936569	Downregulated
Acvr1	-2.97201273	0.01746395	Downregulated
Gm43061; lncRNA	-2.97174478	0.00403341	Downregulated
Gm49197	-2.95763482	0.03402459	Downregulated
Ramp3	-2.95500019	0.02111837	Downregulated
6430710M23Rik; uncharacterized protein LOC76214	-2.95266444	0.0270321	Downregulated
Gm45234[#]; protein-coding	-2.95074586	0.04436555	Downregulated
7SK	-2.94486585	0.0210191	Downregulated
Gm45534[#]	-2.94191689	0.03047183	Downregulated
Daam2	-2.93872233	0.01884153	Downregulated

Sgtb	-2.93692427	0.01895896	Downregulated
Gm47615[#]	-2.93365058	0.0059451	Downregulated
Pcdhga9	-2.9261337	0.02005675	Downregulated
Gm43807[#]	-2.91948381	0.03228729	Downregulated
Gm38142[#]	-2.91922657	0.0369589	Downregulated
Gm44041[#]	-2.9155837	0.01205276	Downregulated
Gm17191; uncharacterized LOC6614398	-2.91158613	0.02688172	Downregulated
Galnt15	-2.90849756	0.03907497	Downregulated
Gm37132[#]	-2.90405547	0.04933455	Downregulated
Lbhd1	-2.88951677	0.00533887	Downregulated
Gm42611[#]	-2.88520881	4.44E-05	Downregulated
Gm48114[#]	-2.88239334	0.01944081	Downregulated
Gm49413[#]	-2.87989263	0.02680405	Downregulated
Egfl7	-2.87242173	0.01344857	Downregulated
C920006O11Rik; lncRNA	-2.87085873	0.03072954	Downregulated
Gm37383[#]	-2.87081323	0.026895	Downregulated
B130021K23Rik; uncharacterized protein LOC319672	-2.86961122	0.03283763	Downregulated
Gm15538 or EG664851[#]; pseudogene	-2.86956473	0.03677101	Downregulated
Gm37255[#]	-2.86877103	0.00956452	Downregulated
Gm47980[#]	-2.86230524	0.04492169	Downregulated
Egfl6	-2.85917971	0.02919461	Downregulated
Nscme3l	-2.84913385	0.04706366	Downregulated
Gm38365[#]	-2.84828525	1.38E-05	Downregulated
Mettl5os; lncRNA	-2.84002295	0.04448921	Downregulated
Gm13868; pseudogene, 60S ribosomal protein L37	-2.83817504	0.03710876	Downregulated
Gm36757; lncRNA	-2.83610197	0.04388005	Downregulated
Fam110d	-2.83159039	0.0435027	Downregulated
Gm11175[#]	-2.81830959	0.00107895	Downregulated
A530064N14Rik; lncRNA	-2.81647623	0.01872909	Downregulated
Gm37537[#]	-2.80971377	0.04609321	Downregulated
Gm50055[#]	-2.79998236	0.03615935	Downregulated
Igkv3-12	-2.7990235	0.00932467	Downregulated
Dach2	-2.78302974	0.03558374	Downregulated
Gm13270; lncRNA	-2.7696026	0.04524065	Downregulated
Fth-ps2; pseudogene, ferritin heavy chain	-2.755194	0.02434211	Downregulated
Itgb8	-2.75396439	0.02815743	Downregulated
Stxbp6	-2.752129	0.00907972	Downregulated
Map2k3os; lncRNA	-2.74194882	0.04562118	Downregulated
Gm42872[#]	-2.73936778	0.0433152	Downregulated

Tox3	-2.73638881	0.0295528	Downregulated
Gm15545; lncRNA	-2.71103313	0.03358989	Downregulated
6820402A03Rik; uncharacterized protein LOC75742	-2.70447118	0.02473174	Downregulated
Rian; lncRNA	-2.70268272	0.0366955	Downregulated
Gm12743; lncRNA	-2.70146019	0.04392769	Downregulated
Gm38262#	-2.70092519	0.04222142	Downregulated
Mir1955; miRNA	-2.69199692	0.03634722	Downregulated
Pcdhga4	-2.69175319	0.03901101	Downregulated
Efna1	-2.69153061	0.04876087	Downregulated
Gm48420#	-2.68388089	0.04424085	Downregulated
Gm43775#	-2.68131403	0.03753032	Downregulated
Gm43111#	-2.67789621	0.04604064	Downregulated
Gm42867#	-2.67591937	0.02554151	Downregulated
Gm44053#	-2.67028136	0.03824857	Downregulated
Gm37115#	-2.65764685	0.00154946	Downregulated
Gm45206#	-2.65391962	0.0046699	Downregulated
Gm48889#	-2.64991034	0.04622524	Downregulated
Mrnip	-2.6425576	0.03234913	Downregulated
Gm44695#	-2.62275371	0.04975625	Downregulated
C430019N01Rik; protein-coding, RIKEN cDNA C430019N01 gene	-2.62138139	0.01735147	Downregulated
Gm47585#	-2.61892243	0.04861102	Downregulated
Gm44152#	-2.61279603	0.01442768	Downregulated
Gm42883#; pseudogene	-2.6101864	0.04254847	Downregulated
9930024M15Rik; lncRNA	-2.60974367	0.01583268	Downregulated
C030017G13Rik; uncharacterized protein LOC319334	-2.597854	0.01446627	Downregulated
Ptk7	-2.5943701	0.04317565	Downregulated
Gm48701#	-2.59147036	0.01091616	Downregulated
7SK or RN7SK; snRNA	-2.58998013	0.02844859	Downregulated
Gm43852#	-2.58359009	0.04690839	Downregulated
Gm36933#	-2.57848936	0.00081162	Downregulated
1810014B01Rik; protein-coding, RIKEN cDNA C430019N01 gene	-2.5773301	0.01440111	Downregulated
Cpa3	-2.57615356	0.0241646	Downregulated
Gprasp2	-2.5598349	0.04622947	Downregulated
Gm43571#	-2.55188163	0.01206745	Downregulated
6230400D17Rik; lncRNA	-2.53375395	0.00608504	Downregulated
Frs3	-2.52693998	0.04693188	Downregulated
Gm37106#	-2.52079522	0.01807082	Downregulated
Gm28693#	-2.51954176	0.0005036	Downregulated

Gm44699; lncRNA	-2.51345136	0.02746491	Downregulated
Susd4	-2.48747446	0.04921572	Downregulated
Gm37390[#]	-2.48699655	0.02587706	Downregulated
Gm38346[#]	-2.48679896	0.00042937	Downregulated
Gm37465[#]	-2.47922466	0.00612206	Downregulated
Sparcl1	-2.47835848	0.01304178	Downregulated
Gm31024; lncRNA	-2.47566946	0.00263887	Downregulated
Gm48139[#]	-2.47441282	0.02790023	Downregulated
Spef2	-2.4739546	0.01933858	Downregulated
Gm43753[#]	-2.45580346	0.00401122	Downregulated
Gm47246[#]	-2.45481378	0.04127946	Downregulated
Gm49783[#]	-2.45377999	0.00987782	Downregulated
Gm45477[#]	-2.43015102	0.0016286	Downregulated
Gm43052[#]	-2.41633574	0.00768747	Downregulated
Gm5511; pseudogene, heat shock protein 90, alpha (cytosolic), class A member 1	-2.40838048	0.02270694	Downregulated
Scg5	-2.4037173	0.03752514	Downregulated
4930556H04Rik; uncharacterized protein LOC75842 or lncRNA	-2.37966001	0.04390214	Downregulated
Ighj2	-2.37952369	0.00313653	Downregulated
Xrcc3	-2.37629808	0.02877933	Downregulated
Gm26779; lncRNA	-2.35236447	0.02849346	Downregulated
Gm37105[#]	-2.34742308	0.01407452	Downregulated
Gm37366[#]	-2.34542953	0.00983071	Downregulated
Wfs1	-2.32945639	0.02171714	Downregulated
Gm44434[#]	-2.31572434	0.008634	Downregulated
Gm45719[#]	-2.30665754	0.01361266	Downregulated
Gm42688[#]	-2.30131182	0.03756555	Downregulated
Gm37529[#]	-2.29028346	0.00681589	Downregulated
A630081D01Rik; uncharacterized protein LOC98754	-2.28783244	0.00856385	Downregulated
Crip3	-2.28420466	0.02612479	Downregulated
Gm42462[#]	-2.27938907	0.01049722	Downregulated
Gm38034[#]	-2.27924938	0.00196471	Downregulated
Gm43484[#]	-2.26201287	0.00244071	Downregulated
Gm38055[#]	-2.248477	0.01908757	Downregulated
Gm43051[#]	-2.24750661	0.0041004	Downregulated
Gm38014[#]	-2.24697647	0.02545435	Downregulated
Gm37139[#]	-2.23739144	0.00971873	Downregulated
Rgs11	-2.22562383	0.027943	Downregulated
Gm38374[#]	-2.19230141	0.02429497	Downregulated
Gm37702[#]	-2.18805064	0.04955438	Downregulated

Gm48624[#]	-2.17844335	0.00145277	Downregulated
Igkv8-19	-2.17046173	0.02792479	Downregulated
Gm43099[#]	-2.1609261	0.04138911	Downregulated
Tmie	-2.15519489	0.00273462	Downregulated
4833421G17Rik; uncharacterized protein LOC73757	-2.15286372	0.03709136	Downregulated
Gm47664; lncRNA	-2.14845385	0.01550942	Downregulated
Unc5c	-2.13189283	0.04605247	Downregulated
4933421O10Rik; lncRNA	-2.13117261	0.00708522	Downregulated
5930430L01Rik; lncRNA	-2.12635405	0.04267193	Downregulated
Gm49396[#]	-2.10278435	0.04210642	Downregulated
Krtcap3	-2.08583467	0.00703302	Downregulated
Zfp202	-2.08061361	0.01197018	Downregulated
Nr6a1	-2.06940351	0.03539911	Downregulated
Zfp775	-2.06855503	0.00594152	Downregulated
Adora3	-2.0616519	0.04554277	Downregulated
Cacna2d4	-2.05418789	0.01876856	Downregulated
Gm36931[#]	-2.04266103	0.00304902	Downregulated
Gm36527[#]	-2.04034508	0.00725616	Downregulated
Gm49705[#]	-2.02139707	0.01098998	Downregulated
Gm37053; lncRNA	-2.02059809	0.01671692	Downregulated
Tlcd3b	-2.0202277	0.00718857	Downregulated
Gm38120[#]	-2.01490094	0.00364117	Downregulated
2700062C07Rik; UPF0711 protein C18orf21 homolog	-2.01012105	0.00293546	Downregulated
Gm44033[#]	-2.00966479	0.04135796	Downregulated
Gm37176[#]	-2.00951508	0.02314031	Downregulated
Gm37524[#]	-2.00798089	0.02243074	Downregulated
Kcng2	-2.00493052	0.02822789	Downregulated
Gm37639[#]	-1.98907613	0.01365365	Downregulated
Gm48904[#]	-1.98780782	0.04666542	Downregulated
1700012D14Rik or Zbed5-ps; pseudogene, zinc finger, BED type containing 5	-1.98375993	0.03837935	Downregulated
Gm37019[#]	-1.97113296	0.01645938	Downregulated
Bach2it1; lncRNA, sense intronic	-1.96628378	0.01055812	Downregulated
Gm38082[#]	-1.95822659	0.0171524	Downregulated
Gm38253[#]	-1.95816325	0.00543375	Downregulated
4933407K13Rik; lncRNA	-1.95142326	0.04147745	Downregulated
Gm43721[#]	-1.94057924	0.04865487	Downregulated
Zswim9	-1.93936677	0.0003638	Downregulated
Gm44027[#]	-1.93631671	0.00500521	Downregulated
Adamts14	-1.93609269	0.00679256	Downregulated

Gm43196#	-1.93098728	0.02090616	Downregulated
Ust	-1.93097915	0.00424883	Downregulated
Zfp599	-1.92487711	0.0214287	Downregulated
Gm43329#	-1.92084946	0.02043655	Downregulated
Gm37677#	-1.91557194	0.02954419	Downregulated
Arl4d	-1.91310276	0.02770572	Downregulated
Gm37663#	-1.90556528	0.04575183	Downregulated
Gm48225#	-1.90424391	0.01299058	Downregulated
Gm11400; pseudogene, elongation factor 2	-1.89973457	0.01055056	Downregulated
Gm9796#	-1.89745013	0.04653486	Downregulated
Gm38220#	-1.88005707	0.03524498	Downregulated
Mybpc3	-1.86694001	0.04209173	Downregulated
Gm28373; lncRNA	-1.86668924	0.01632772	Downregulated
Gm37488#	-1.85616425	0.00363836	Downregulated
Gm17690; lncRNA	-1.85289296	0.01593369	Downregulated
C130074G19Rik; Required for drug-induced death protein 1	-1.85288822	0.01541173	Downregulated
Esr1	-1.85183885	0.02892676	Downregulated
Nat8f4	-1.85182033	0.03067127	Downregulated
Gm49839#	-1.84462461	0.0432547	Downregulated
Chic1	-1.83720179	0.01540856	Downregulated
Gm20696#	-1.83603474	0.00181986	Downregulated
C130089K02Rik; lncRNA	-1.78251998	0.01783401	Downregulated
Gm44891#	-1.77695086	0.02273249	Downregulated
Vangl2	-1.77050953	0.01441936	Downregulated
Gm38366; lncRNA	-1.76094735	0.01147436	Downregulated
Phxr4; lncRNA	-1.75782015	0.04235715	Downregulated
Gm45342#	-1.75702795	0.03348447	Downregulated
5330406M23Rik; uncharacterized protein LOC76671	-1.73266546	0.00646605	Downregulated
Gm37159#	-1.73085728	0.03056069	Downregulated
Gm19337#	-1.73073542	0.01736956	Downregulated
Gm42856#	-1.72411127	0.04178432	Downregulated
Gm44153#	-1.71851083	0.03408554	Downregulated
Slc22a5	-1.71316847	0.00277434	Downregulated
Gm43274#	-1.70622436	0.04537835	Downregulated
Gm29488#	-1.69907447	0.04741986	Downregulated
Gm38372#	-1.69525851	0.00374519	Downregulated
Gm43330#	-1.68232856	0.03173485	Downregulated
Gm37906#	-1.67303847	0.01083565	Downregulated
Gm44745#	-1.66327898	0.02797594	Downregulated
Gm37943; lncRNA	-1.66012451	0.01824981	Downregulated
Zfp28	-1.65480151	0.01034808	Downregulated

Ncr1	-1.64854802	0.02545373	Downregulated
Gm43457[#]	-1.64136878	0.02310023	Downregulated
Fam167a	-1.60865394	0.03613398	Downregulated
C230085N15Rik; lncRNA	-1.60546764	0.02993112	Downregulated
Dph2	-1.56916333	0.00662794	Downregulated
Gm37589[#]	-1.54428361	0.01416321	Downregulated
Gm44664[#]	-1.53903737	0.04011815	Downregulated
Il18bp	-1.53757162	0.0460668	Downregulated
2410004B18Rik; UPF0690 protein C1orf52 homolog	-1.53606004	0.00559344	Downregulated
Gm43300[#]	-1.53148443	0.0327369	Downregulated
Gm37593[#]	-1.52002498	0.026604	Downregulated
Fam222a	-1.51255435	0.04030467	Downregulated
Gm37296[#]	-1.5110516	0.03546071	Downregulated
5330432J10Rik; uncharacterized protein LOC320151	-1.48192167	0.03633083	Downregulated
Gm15675; lncRNA	-1.48139286	0.01585504	Downregulated
Gm21781 or LOC6609604; protein 5NUC	-1.43634072	0.01893351	Downregulated
Gm38190[#]	-1.4319401	0.04990458	Downregulated
Gm37206[#]	-1.42593761	0.0384095	Downregulated
Zfp707	-1.42262441	0.04407231	Downregulated
Dock6	-1.42169728	0.03326772	Downregulated
Rapgef4	-1.40929493	0.01551628	Downregulated
Acsf3	-1.40578004	0.03278462	Downregulated
Zfp157	-1.38462357	0.00431051	Downregulated
Gm6712; zinc finger protein 431-like	-1.37266071	0.02800755	Downregulated
Sesn2	-1.36929473	0.02923999	Downregulated
Abcc10	-1.36254868	0.03469282	Downregulated
Gprc5b	-1.36220936	0.04783021	Downregulated
Apex1	-1.3542316	0.01958232	Downregulated
Snapc2	-1.35407332	0.04942986	Downregulated
Zfp729a	-1.35277717	0.00291218	Downregulated
Rnf122	-1.33915389	0.02577825	Downregulated
Cradd	-1.31425054	0.03191825	Downregulated
BE692007; lncRNA	-1.29858271	0.03339695	Downregulated
Snx33	-1.27908315	0.04675242	Downregulated
Dock9	-1.22747017	0.01224732	Downregulated
Mief2	-1.22605171	0.03681332	Downregulated
Rai1	-1.21187901	0.03494247	Downregulated
Sidt1	-1.20606678	0.01873775	Downregulated
Sufu	-1.19775764	0.00694994	Downregulated
Gvin-ps6; pseudogene,	-1.18235264	0.02183154	Downregulated

GTPase, very large interferon inducible			
Kcnq5	-1.17190895	0.04712438	Downregulated
Btbd9	-1.11304107	0.03381874	Downregulated
Btla	-1.09421794	0.04566481	Downregulated
Chst3	-1.08015993	0.03806637	Downregulated
Asb7	-1.04871203	0.02264448	Downregulated
Dph3	1.01568264	0.02757446	Upregulated
Cript	1.06449422	0.02367574	Upregulated
mt-Nd6	1.08477013	0.04200423	Upregulated
Sf3b6	1.1877662	0.01715849	Upregulated
Pyurf	1.1976408	0.04755457	Upregulated
Ccdc167	1.19973307	0.02799452	Upregulated
Tsen15	1.20041638	0.00621684	Upregulated
Acot13	1.22831256	0.03301922	Upregulated
Fam151b	1.27587112	0.03017983	Upregulated
Ndufb4	1.29261126	0.04845882	Upregulated
Rnf26	1.30903513	0.03822103	Upregulated
Ccsap	1.32325737	0.04528318	Upregulated
Homer1	1.32549202	0.03424507	Upregulated
Nbea	1.33625258	0.04196336	Upregulated
Cdkn2c	1.35671936	0.00800239	Upregulated
Fasn	1.35681788	0.03175944	Upregulated
Atp5mpl or Atp5mj	1.37560571	0.02929179	Upregulated
Gm7536; pseudogene, ribosomal protein L27A	1.41621973	0.02216735	Upregulated
Cplx2	1.46132714	0.0456128	Upregulated
Naa38	1.47537019	0.00231291	Upregulated
Tmsb4x	1.48727237	0.00323896	Upregulated
Micos13	1.49283018	0.00649107	Upregulated
Atp5md; or Atp5mk	1.4985063	0.03640157	Upregulated
Map1lc3b	1.51621049	0.00017746	Upregulated
Gm10076; pseudogene, ribosomal protein L41	1.52709091	0.0349835	Upregulated
Uqcr10	1.53909266	0.01869224	Upregulated
Zcchc14	1.54299416	0.04754277	Upregulated
Atp5j2 or Atp5mf	1.57445414	0.00733128	Upregulated
Tmem176b	1.58330555	0.01536034	Upregulated
Pfdn5	1.58509992	0.00068634	Upregulated
Rpl17-ps8; pseudogene 8, ribosomal protein L17	1.58671717	0.03900027	Upregulated
Lockd; lncRNA, downstream of Cdkn1b	1.59020242	0.03274153	Upregulated
Aldoc	1.60286407	0.00523754	Upregulated
Ghr	1.62055236	0.00493294	Upregulated
Cox7a2	1.63717151	0.01709797	Upregulated

Uqcrh	1.6665213	0.01399229	Upregulated
Gm9517; pseudogene, predicted gene 9517	1.67434027	0.03634912	Upregulated
Kitl	1.67982577	0.03622905	Upregulated
Aifm2	1.68451786	0.00409272	Upregulated
Mrc1	1.69678284	0.03305663	Upregulated
Rab11fip5	1.69987105	0.01020185	Upregulated
Timm8b	1.7237302	0.00207083	Upregulated
Gm5617; uncharacterized protein C11orf71 homolog	1.7285371	0.02353546	Upregulated
Asxl3	1.7371097	0.0228066	Upregulated
Fam174c	1.73938529	0.02560718	Upregulated
Rpl17-ps5; pseudogene 5, ribosomal protein L17	1.74937555	0.04832058	Upregulated
Snrnp25	1.7654248	0.00188754	Upregulated
Psma8	1.78406926	0.04196773	Upregulated
Gm10257; pseudogene, H3 histone, family 3A	1.79168871	0.01443836	Upregulated
Wfdc17	1.79270287	0.04707712	Upregulated
Gm40578; lncRNA	1.82005248	0.03140526	Upregulated
Tmem91	1.82733252	0.02976611	Upregulated
Acacb	1.83733886	0.01946944	Upregulated
Cav1	1.84595769	0.04932736	Upregulated
Ndufa7	1.84631856	0.00515975	Upregulated
Spats2	1.91168022	0.00751465	Upregulated
1700007L15Rik; lncRNA	1.91770118	0.04103793	Upregulated
Gm34961; lncRNA	1.93032169	0.03235016	Upregulated
Pdia5	1.95041439	0.00348555	Upregulated
Rgs8	1.95125843	0.03032019	Upregulated
Tmem202	1.95720425	0.02784101	Upregulated
Nt5dc2	1.95839729	0.03662578	Upregulated
Igfbp5	1.99831895	0.02465982	Upregulated
Lrrc69	2.014642	0.00561914	Upregulated
Gm46209; pseudogene	2.06706048	0.02125875	Upregulated
Tm4sf1	2.07305101	0.00127354	Upregulated
Gm6055; pseudogene 2, Microtubule-associated protein 1 light chain 3	2.07559555	0.00024087	Upregulated
Gm50046[#]	2.11125938	0.02682915	Upregulated
Lpar1	2.11630662	0.04945094	Upregulated
Fam13a	2.12150586	0.00499128	Upregulated
Gm5909; pseudogene, zinc finger CCCH-type containing 15	2.13972695	0.04782638	Upregulated
Fn1	2.14115071	0.01197175	Upregulated
Acot6	2.14382561	0.02024047	Upregulated

Gls2	2.16635767	0.01190299	Upregulated
Snord59a; snoRNA	2.20848419	0.04232525	Upregulated
Arhgef5	2.21282106	0.04751733	Upregulated
Nmbr	2.23101183	0.00105576	Upregulated
Grhl1	2.2526463	0.00846257	Upregulated
Ephb4	2.27974677	0.00225697	Upregulated
Enpp3	2.28495617	0.02193265	Upregulated
Cyp2u1	2.30562795	0.00471953	Upregulated
Dmd	2.31651329	0.04028832	Upregulated
Parva	2.31882129	0.00379754	Upregulated
Adamts17	2.32131085	0.0447583	Upregulated
Rpl3l	2.32417269	0.03265499	Upregulated
Sh3pxd2b	2.32939654	0.00350382	Upregulated
Plin4	2.33891657	0.03121805	Upregulated
BC065403; lncRNA	2.34119091	0.02215663	Upregulated
Plekhh2	2.34178858	0.01177346	Upregulated
Ahnak2	2.34186491	0.02794245	Upregulated
Mst1r	2.36836786	0.0413388	Upregulated
Sdc2	2.39534784	0.01917512	Upregulated
Phf24	2.40072097	0.00041071	Upregulated
Rgs22	2.40111439	0.04120367	Upregulated
Cabcoco1	2.41721523	0.04871757	Upregulated
Tbr1	2.47881347	0.00739021	Upregulated
Col18a1	2.48596291	0.01873295	Upregulated
Tmem88b	2.49427063	0.00262997	Upregulated
Col14a1	2.49530205	0.04633999	Upregulated
Mcrip2	2.49567118	0.00222472	Upregulated
Ica1l	2.49838925	0.00122891	Upregulated
Mertk	2.51544427	0.03354794	Upregulated
Calr3	2.51691892	0.01955833	Upregulated
Lag3	2.51954092	0.00572438	Upregulated
Gm10925 or Atp6-ps; pseudogene, ATP synthase membrane subunit 6	2.5602195	0.00279738	Upregulated
Xkr5	2.56522287	0.04228146	Upregulated
Sytl5	2.5785197	0.01633973	Upregulated
Islr2	2.58829391	0.0352156	Upregulated
Gm47493[#]	2.62060319	0.02918403	Upregulated
Thbs2	2.62363954	0.0143933	Upregulated
Mir144; miRNA	2.62412133	0.02509313	Upregulated
Mroh8	2.62521964	0.03730571	Upregulated
6530409C15Rik; lncRNA	2.65190834	0.02186629	Upregulated
Car3	2.65417538	0.01109728	Upregulated
Cnnm1	2.66393609	0.01139041	Upregulated
Hbegf	2.66604999	0.01155263	Upregulated

Chst7	2.67786685	0.01046785	Upregulated
Tgm1	2.74614833	0.04767715	Upregulated
Syt16	2.78718385	0.03234579	Upregulated
P4ha3	2.8020347	0.03407933	Upregulated
Bmp4	2.83181867	0.00254845	Upregulated
Cerox1; lncRNA	2.848941	0.01446181	Upregulated
Rbms3	2.85626956	0.00508287	Upregulated
Vcam1	2.87496866	0.01906384	Upregulated
Bche	2.91464055	0.0491629	Upregulated
Vgf	2.91555458	0.04872643	Upregulated
Ccdc159	2.91834812	0.04741915	Upregulated
Gpr6	2.91854331	0.04732926	Upregulated
Gm16701; lncRNA	2.91958085	0.04685383	Upregulated
Hapln2	2.9197576	0.04677301	Upregulated
A830018L16Rik; uncharacterized protein C8orf34 homolog	2.92220956	0.04567082	Upregulated
Oprd1	2.92227043	0.04724499	Upregulated
Pfn4	2.92312388	0.04825818	Upregulated
Srgap1	2.92327551	0.0452003	Upregulated
Iqsec3	2.92339216	0.04514877	Upregulated
Cdh11	2.92379313	0.04497353	Upregulated
Trpc3	2.92381771	0.04496292	Upregulated
Ntsr1	2.92391219	0.0480595	Upregulated
Necab2	2.92437372	0.04529775	Upregulated
Wnk3	2.92540363	0.04451862	Upregulated
Masp1	2.92552561	0.04556479	Upregulated
Lrfn5	2.9258004	0.04469722	Upregulated
Nap1l2	2.92580563	0.04471201	Upregulated
Myt1l	2.92589971	0.04452923	Upregulated
Kndc1	2.92593149	0.04506825	Upregulated
Rnf208	2.9259364	0.04508009	Upregulated
Pcsk2	2.92597031	0.04518004	Upregulated
Fam171b	2.92601346	0.04532322	Upregulated
Nos1	2.92603069	0.04535353	Upregulated
Cdh12	2.92608898	0.04552234	Upregulated
Cckbr	2.9261087	0.04557945	Upregulated
Mab21l1	2.92634098	0.04627783	Upregulated
Pcsk5	2.92681282	0.04508358	Upregulated
Syt10	2.92714658	0.04895901	Upregulated
Gm48822[#]	2.92720921	0.04918386	Upregulated
Gm5067; pseudogene, ribosome biogenesis regulatory protein	2.92726876	0.0494049	Upregulated
Zic1	2.92778619	0.04469125	Upregulated
Abca8a	2.92787299	0.03264613	Upregulated

Cfap44	2.92946111	0.04933025	Upregulated
Gm973; uncharacterized protein KIAA2012 homolog	2.92987382	0.03225765	Upregulated
Cacna2d3	2.93031334	0.0315364	Upregulated
Snap91	2.93375995	0.03081305	Upregulated
Sat2	2.93377619	0.04474912	Upregulated
Fam110c	2.93487927	0.01231589	Upregulated
Ddr2	2.93537453	0.00261164	Upregulated
Actn3	2.93558425	0.04007628	Upregulated
Fstl1	2.94269726	0.01978995	Upregulated
Gm15663; lncRNA	2.9439747	0.03133728	Upregulated
Prkar1b	2.94595921	0.02015241	Upregulated
Ddit4l	2.94624768	0.0475433	Upregulated
Cpxm1	2.94751733	0.04576664	Upregulated
Zdbf2	2.95019559	0.01976171	Upregulated
Ppp1r26	2.95090207	0.01960385	Upregulated
Camp	2.95698985	0.03538351	Upregulated
Ptgr1	2.96258207	0.0094119	Upregulated
Jam3	2.96762761	0.00923992	Upregulated
Spire2	2.96836968	0.02109313	Upregulated
Tspan7	2.96862344	0.0092264	Upregulated
Fabp7	2.97166287	0.04164056	Upregulated
4933412O06Rik; lncRNA	2.97196149	0.04780999	Upregulated
Cldn10	2.97948879	0.01939987	Upregulated
Cyp3a59	2.99354059	0.02431157	Upregulated
Shisal2b	3.00006685	0.03223532	Upregulated
Abcc9	3.05711352	0.04465934	Upregulated
Agbl2	3.0607908	0.03401224	Upregulated
Slc4a4	3.07657795	0.00632101	Upregulated
Cd163	3.08248489	0.00415343	Upregulated
Efemp1	3.08697686	0.00701593	Upregulated
Synpo2	3.09845703	0.01385532	Upregulated
Tspan6	3.10734647	0.00287881	Upregulated
Acy3	3.11646447	0.02580866	Upregulated
Adra1a	3.11953145	0.02255363	Upregulated
Gm9353; pseudogene, bgcn homolog	3.14049339	0.03172466	Upregulated
Kcnt2	3.14679	0.01705247	Upregulated
Rsph1	3.15006684	0.01917545	Upregulated
Coro2b	3.15437091	0.01605287	Upregulated
Serping1	3.15553087	0.033103	Upregulated
Mafa	3.15581809	0.00628341	Upregulated
Pcdh9	3.15608723	0.01576061	Upregulated
Dock3	3.15623935	0.01570661	Upregulated
Foxc1	3.16136925	0.01934937	Upregulated

Gm49894[#]; protein-coding	3.16388636	0.02901816	Upregulated
Aspn	3.19051058	0.03356235	Upregulated
Catsper3	3.19112169	0.01924021	Upregulated
1700016P03Rik; lncRNA	3.21734342	0.02437947	Upregulated
Lyplal1	3.22609753	0.0275801	Upregulated
Mei1	3.23661196	0.02224389	Upregulated
Gm19935; lncRNA	3.23699784	0.03700562	Upregulated
Cfap91	3.23776093	0.02263139	Upregulated
Tacr3	3.2385529	0.02023489	Upregulated
Fabp3	3.24115562	0.01880177	Upregulated
Postn	3.24148224	0.04718158	Upregulated
Eps8l2	3.24424158	0.02188339	Upregulated
Dagla	3.24502234	0.01798881	Upregulated
Foxp2	3.24581829	0.01767172	Upregulated
Pygo1	3.24587649	0.01759645	Upregulated
Wnt5a	3.24619343	0.0182497	Upregulated
Gucy2c	3.2463797	0.04560783	Upregulated
Fgf14	3.24645659	0.01740567	Upregulated
Ptpn5	3.24674173	0.01751781	Upregulated
Smad9	3.24724135	0.01764666	Upregulated
Lurap1	3.24769942	0.01798393	Upregulated
Lin7a	3.24775875	0.01746454	Upregulated
Gria4	3.24779104	0.0173481	Upregulated
Apba2	3.2478591	0.01759526	Upregulated
Slc45a1	3.24808179	0.01789106	Upregulated
Shisa2	3.24843036	0.01838181	Upregulated
Hhatl	3.24955394	0.01852515	Upregulated
Gm15666; pseudogene, Keratin 19	3.25039725	0.02204596	Upregulated
Npsr1	3.25125394	0.02443066	Upregulated
Grb14	3.2512744	0.01745674	Upregulated
Tox2	3.25282426	0.01629714	Upregulated
6030407O03Rik; lncRNA	3.25342309	0.04340987	Upregulated
Lmcd1	3.26025528	0.01874183	Upregulated
Gm30329; lncRNA	3.26516465	0.04721636	Upregulated
Tmem100	3.27117593	0.01796491	Upregulated
Medag	3.27303224	0.01847833	Upregulated
Spint1	3.28199325	0.02504392	Upregulated
Pdk4	3.28698254	0.00891382	Upregulated
Pth2r	3.29334274	0.01970181	Upregulated
Kif1a	3.29367618	0.03895249	Upregulated
Ltc4s	3.29606015	0.03799365	Upregulated
Eda2r	3.31081873	0.04462331	Upregulated
Ghrl	3.32003514	0.01072745	Upregulated
Scin	3.3307771	0.02333569	Upregulated
Ccdc169	3.33078524	0.02554742	Upregulated

Fblim1	3.3427163	0.03516748	Upregulated
Tmem63c	3.34722833	0.00851936	Upregulated
Ms4a7	3.34775037	0.04027269	Upregulated
Cdk18;<br>or Pctk3 or Pctaire3	3.34784286	0.00887325	Upregulated
Smarca5-ps; pseudogene, SNF2 related chromatin remodeling ATPase 5	3.35282865	0.04235302	Upregulated
Slc4a10	3.35347732	0.00835051	Upregulated
Spaca6	3.35868028	0.00067775	Upregulated
Pld6	3.35975872	0.01654296	Upregulated
Prg2	3.36106387	0.01172811	Upregulated
Gm28050; lncRNA	3.36966165	0.03889794	Upregulated
Rpl36-ps2; pseudogene 2, Ribosomal protein L36	3.37084684	0.02474516	Upregulated
Dab2	3.37518257	0.002353	Upregulated
C1qb	3.3841623	0.00050406	Upregulated
Gm11868; pseudogene, Cytochrome b5 reductase 4	3.39029712	0.03588234	Upregulated
Gm48684; lncRNA	3.39060379	0.0250466	Upregulated
Cbr2	3.42399216	0.045309	Upregulated
Gldn	3.43224507	0.020271	Upregulated
Aoc3	3.44772859	0.01031606	Upregulated
Ankrd36	3.45350536	0.01529275	Upregulated
Gm14327; Zfp1003	3.47037746	0.01758575	Upregulated
Gm11653; pseudogene, Snurportin 1	3.47165029	0.01172964	Upregulated
Chrna1os; lncRNA, Cholinergic receptor nicotinic alpha 1 subunit, opposite strand	3.47782627	0.01771632	Upregulated
Gm44997; lncRNA	3.4858424	0.00971353	Upregulated
Col12a1	3.4867168	0.00398426	Upregulated
Zfp449	3.49114031	0.00507104	Upregulated
Il33	3.49184482	0.01059742	Upregulated
Cyp1b1	3.49430648	0.00730909	Upregulated
C230031I18Rik[#]; protein-coding	3.49514332	0.00860375	Upregulated
Rsph14	3.49568758	0.03452086	Upregulated
Elovl4	3.4963317	0.01479776	Upregulated
Pm20d1	3.49718661	0.00864316	Upregulated
Zbtb7c	3.49723696	0.00863641	Upregulated
Pak6	3.49876224	0.00121671	Upregulated
Rpl36a-ps1; pseudogene 1, Ribosomal protein L36A	3.49877703	0.0326291	Upregulated
Thbs4	3.50155566	0.00894573	Upregulated
Cyp2j9	3.50157712	0.00791961	Upregulated

Gm30015; lncRNA	3.50660758	0.04320216	Upregulated
Sfrp1	3.50664638	0.00748991	Upregulated
Adora1	3.50709501	0.00738136	Upregulated
Insm1	3.50753132	0.00803434	Upregulated
Sema6a	3.50845171	0.0072555	Upregulated
Chst1	3.50901872	0.00739062	Upregulated
Gm37363[#]	3.51257092	0.00845152	Upregulated
Bmp6	3.51281256	0.00506109	Upregulated
Gm8173; pseudogene, DEAD (Asp-Glu-Ala-Asp) box polypeptide 18	3.51352496	0.00941642	Upregulated
St8sia3	3.51362533	0.00712115	Upregulated
Asic4	3.51550291	0.00822739	Upregulated
Spry4	3.51623821	0.00468829	Upregulated
Gm42899[#]	3.51685533	0.00814504	Upregulated
Fkbp14	3.51776265	0.00715938	Upregulated
Ccdc81	3.51991978	0.01042328	Upregulated
Kcnk12	3.52044951	0.00501353	Upregulated
Gm44848[#]	3.5205902	0.00894753	Upregulated
Flrt3	3.52082815	0.00457836	Upregulated
Fgf2	3.52111908	0.0083226	Upregulated
Alox8	3.52412873	0.01568929	Upregulated
Gm15910; lncRNA	3.5269488	0.03110819	Upregulated
Rbm24	3.52702775	0.02800208	Upregulated
Angptl2	3.54330542	0.00853589	Upregulated
4933424G06Rik; uncharacterized protein C2orf92 homolog	3.55402508	0.03866349	Upregulated
Gm37953[#]	3.56953795	0.0068249	Upregulated
9630001P10Rik; lncRNA	3.57483468	0.00980709	Upregulated
Acat3	3.58963155	0.00209791	Upregulated
Sec14l3	3.59608096	0.04271492	Upregulated
Hopxos; lncRNA	3.61402271	0.02343563	Upregulated
Spdya	3.6200739	0.00333874	Upregulated
Ninj2	3.62263294	0.01242707	Upregulated
Olfr267	3.63681596	0.01335908	Upregulated
Gm42669[#]; protein-coding	3.63804506	0.04854248	Upregulated
Bicc1	3.65103465	0.01441191	Upregulated
Pth1r	3.65148111	0.00337377	Upregulated
Slc17a7	3.67003073	0.0025799	Upregulated
Kank1	3.67033878	0.00261927	Upregulated
Pdcd1	3.67489803	0.01313062	Upregulated
Ttll9	3.68140368	0.00640056	Upregulated
Bcam	3.69576029	0.00555234	Upregulated
Myh4	3.70560963	0.01764567	Upregulated
Gas6	3.70814696	0.00014776	Upregulated

Mgp	3.72038965	0.00853749	Upregulated
Depdc7	3.72321201	0.00402971	Upregulated
Gm49069[#]	3.7264238	0.00367745	Upregulated
Ppfia2	3.72969255	0.00320076	Upregulated
Pcdhb22	3.73234888	0.00328544	Upregulated
Tnc	3.73246548	0.00344705	Upregulated
Dab1	3.73280782	0.00312567	Upregulated
Flrt2	3.73367014	0.00325571	Upregulated
Gas1	3.7589899	0.00205408	Upregulated
Stard13	3.76075754	0.00930754	Upregulated
Ptprd	3.77868366	0.0009033	Upregulated
Ccdc142	3.78309905	0.04293782	Upregulated
Fbl-ps2; pseudogene 2, Fibrillarin	3.78436276	0.01048783	Upregulated
Plekhd1	3.80619726	0.00206732	Upregulated
Ttc26; or Ift56	3.80986304	0.00157113	Upregulated
Gpd1	3.81152725	5.60E-08	Upregulated
Pdcd5-ps; pseudogene, Programmed cell death 5	3.82089171	0.00581712	Upregulated
8430432A02Rik; lncRNA, Translation initiation factor IF-2-like	3.8703945	0.00669613	Upregulated
Tpbgl	3.87493048	0.00287916	Upregulated
Tmem45b	3.88175256	0.03401544	Upregulated
Col6a2	3.88580689	0.00662966	Upregulated
Tmem37	3.8890042	0.0092904	Upregulated
Rbp4	3.89666168	0.00253917	Upregulated
Scn7a	3.90411687	0.00241406	Upregulated
Ighg1	3.90516998	0.01848865	Upregulated
Gm44237[#]	3.90737351	0.00630019	Upregulated
Chrna2	3.90832532	0.02722709	Upregulated
Gm20661[#]; protein-coding	3.91048699	0.01847879	Upregulated
Fras1	3.91497865	0.00174012	Upregulated
Gm50341; lncRNA	3.91498378	0.03116899	Upregulated
Kcnn3	3.91613487	0.0074333	Upregulated
Pcdhgc5	3.92142109	0.00155133	Upregulated
Rgs7bp	3.92293195	0.00144918	Upregulated
Chadl	3.92696427	0.00158669	Upregulated
Etv4	3.92753059	0.00168859	Upregulated
Pgr	3.92764998	0.00147267	Upregulated
Gm14863[#]	3.93336494	0.04083815	Upregulated
Gm13479; lncRNA	3.93550893	0.04498302	Upregulated
Bgn	3.93674159	0.00011563	Upregulated
Nr2f2	3.93676418	0.00095894	Upregulated
Inmt	3.93768859	0.01450098	Upregulated

4930505A04Rik; or Ciliary microtubule inner protein 6 (Cimip6)	3.94570171	0.01898165	Upregulated
Tc2n	3.95029909	0.00788298	Upregulated
Prok2	3.95077804	0.03803395	Upregulated
Pdpn	3.95357677	0.00155825	Upregulated
Tmem119	3.95552409	0.00223845	Upregulated
5330439K02Rik; lncRNA	3.96303007	0.00335813	Upregulated
G530011O06Rik; lncRNA	3.96770283	0.01732117	Upregulated
Kif28	3.97076196	0.04231423	Upregulated
Alx4	4.01204121	0.00411266	Upregulated
Mgl2	4.03282383	0.00605871	Upregulated
Grin3b	4.04443331	0.01395156	Upregulated
Cgnl1	4.06132881	6.35E-05	Upregulated
Ankef1	4.07317265	0.00105875	Upregulated
Dynlt2a2	4.08389316	0.04623194	Upregulated
Kcnk3	4.08832365	0.00076578	Upregulated
Mt2	4.09122858	0.00080193	Upregulated
Csmd1	4.09186187	0.0007359	Upregulated
Kcnk2	4.09339444	0.00073142	Upregulated
Dcn	4.09475899	8.21E-05	Upregulated
Ntrk2	4.09479994	0.00068052	Upregulated
Cox8b	4.09670732	0.01315877	Upregulated
Rorb	4.09741694	0.00070821	Upregulated
Gm16364; lncRNA	4.09894061	0.01289222	Upregulated
Fgfr3	4.10407044	0.00069486	Upregulated
Acss3	4.11659524	0.00098132	Upregulated
Gm48894[#]	4.12913713	0.0087542	Upregulated
Fam20a	4.13358374	0.00308327	Upregulated
9130008F23Rik; protein coding, uncharacterized protein C6orf141 homolog	4.14505464	0.02937752	Upregulated
Des	4.15234411	0.01032613	Upregulated
Spink10	4.15478601	0.00192496	Upregulated
Rgs7	4.15485177	0.00032341	Upregulated
Slc13a4	4.15607357	0.0006362	Upregulated
Gm37634[#]	4.16474696	0.00779487	Upregulated
Ebf2	4.16649006	0.00100154	Upregulated
B3gnt9	4.18307574	0.00128875	Upregulated
Calcb	4.18584508	0.00416712	Upregulated
Tepp	4.18849997	0.0213333	Upregulated
Fbln1	4.19525572	0.00073494	Upregulated
Gm15843; pseudogene, Ribosomal protein L17	4.19789093	0.04624568	Upregulated
Scara3	4.24091371	0.00040618	Upregulated
Clmn	4.24550944	0.00036871	Upregulated

Mgat3	4.24811361	0.00034232	Upregulated
Plekhh1	4.24877939	0.000372	Upregulated
Gucy2g	4.24937156	0.00063446	Upregulated
Kcnq4	4.25098926	0.00050213	Upregulated
Tspan11	4.25208287	0.0005071	Upregulated
Dkk3	4.25536556	0.00034632	Upregulated
Chmp4c	4.26406844	0.01126682	Upregulated
Rarres2	4.27005037	0.00238918	Upregulated
Lgals12	4.28813344	0.00251777	Upregulated
Gm9830; pseudogene, Family with sequence similarity 103, member A1	4.30649033	0.01309473	Upregulated
Ak9	4.33424718	0.00098707	Upregulated
C1qc	4.33783215	0.00038043	Upregulated
Cdkn2a	4.34299636	0.03969282	Upregulated
C1s1	4.37938247	0.00554031	Upregulated
Ildr2	4.38350792	0.0001789	Upregulated
Adh7	4.42333696	0.00271367	Upregulated
Aebp1	4.42468418	0.00030152	Upregulated
Slc36a2	4.42605609	0.00220023	Upregulated
Klhdc7a	4.46709432	0.00165777	Upregulated
4930544I03Rik; protein coding, uncharacterized protein LOC78197	4.49153474	0.00308694	Upregulated
Mme; or Neprilysin	4.50667291	0.00011392	Upregulated
Ppp1r3c	4.51533497	3.30E-05	Upregulated
Cilp	4.51582229	0.02155228	Upregulated
Vamp9	4.51704571	0.00407962	Upregulated
Pde6a	4.53100857	0.00287622	Upregulated
Timd4	4.55627474	0.02588775	Upregulated
Trarg1	4.57358904	0.00195956	Upregulated
Antxr1	4.59087857	4.12E-05	Upregulated
Cd248; or Endosialin	4.59605836	0.00180727	Upregulated
Fam217a	4.61176146	0.00438875	Upregulated
Prox1	4.61629116	5.98E-05	Upregulated
Srl	4.67066268	4.58E-05	Upregulated
Ptgis	4.70952397	0.00385078	Upregulated
Marco	4.77306241	0.03625825	Upregulated
Zmynd15	4.86497538	0.00367385	Upregulated
Adamts13	4.94291946	0.00511921	Upregulated
Clstn3	4.98819699	5.15E-06	Upregulated
Saa3	4.98901495	0.02566068	Upregulated
Pck1	5.00138481	0.00072092	Upregulated
6330403K07Rik; protein coding, uncharacterized protein LOC103712	5.02016431	6.79E-06	Upregulated

Ncan	5.08377985	4.50E-06	Upregulated
Prelp	5.09217585	2.43E-05	Upregulated
Ces1d	5.11158095	0.00129462	Upregulated
Me1	5.12916536	1.24E-06	Upregulated
Fbxo15	5.1356014	0.00015201	Upregulated
Cd209b	5.1457267	0.00320511	Upregulated
Dpt	5.29776216	0.00011474	Upregulated
Mlxipl	5.31746683	0.00057196	Upregulated
Cidec	5.37138184	0.01517998	Upregulated
Prg4	5.42059907	3.30E-05	Upregulated
Sulf1	5.59341198	8.54E-07	Upregulated
Fcrls; or Fcrl2	5.63382481	2.65E-05	Upregulated
Atp1a2	5.66732667	1.99E-05	Upregulated
Fcna	6.11179816	5.08E-05	Upregulated
C1qa	6.22640146	1.50E-06	Upregulated
Cidea	6.3566198	4.90E-06	Upregulated
C4b	6.44759989	3.40E-06	Upregulated
Retnla; or Fizz1	6.48248328	0.00102751	Upregulated
Ednrb	6.8518195	2.06E-05	Upregulated
Plin1	7.04321453	1.88E-06	Upregulated
Thrsp; or Spot14	7.31940107	7.41E-09	Upregulated
Ucp1	7.58505474	1.55E-05	Upregulated
Sult4a1	16.8141827	2.42E-16	Upregulated
Cyp2f2	20.3068344	1.88E-26	Upregulated
Rag2	43.1629737	5.38E-23	Upregulated

Table of Contents Top of Current Table

Supplemental Table 2. List of all significant (P < 0.05) differentially expressed genes in whole blood of wild-type B6129 vehicle (week 8) versus wild-type B6129 vehicle (week 0) animals; 593 genes downregulated & 198 genes upregulated.

Gene Name	Log2FoldChange	P-value	Regulation
mt-Th	-6.36664471	0.032487	Downregulated
Pcdh10	-5.92268016	1.11E-05	Downregulated
Sgsm1	-5.87532903	3.44E-05	Downregulated
Depp1	-5.79666335	0.000936	Downregulated
Myrip	-5.68570937	2.92E-05	Downregulated
Cadps	-5.6176868	3.81E-05	Downregulated
Acot12	-5.6147316	0.0025	Downregulated
Col19a1	-5.60815399	4.37E-05	Downregulated
Bcat1	-5.54023556	5.44E-05	Downregulated
mt-Nd4l	-5.51811108	0.016414	Downregulated
C130073E24Rik	-5.49993699	0.000152	Downregulated
Robo4	-5.46528101	0.000336	Downregulated
Oprm1	-5.46110536	8.09E-05	Downregulated
Gm23330	-5.40488666	0.00253	Downregulated
Atp2b2	-5.39210105	9.21E-05	Downregulated
Gm19461	-5.30807496	0.002849	Downregulated
Slc22a12	-5.25409823	0.033818	Downregulated
Dipk2b	-5.25051819	0.004051	Downregulated
Loxhd1	-5.24480148	0.009003	Downregulated
Dazl	-5.20872692	0.000254	Downregulated
Otogl	-5.17689858	0.001034	Downregulated
Mir421	-5.15647134	0.000386	Downregulated
ENSMUSG00002075991	-5.15613717	0.014172	Downregulated
Gpld1	-5.13307675	0.000253	Downregulated
Grin2a	-5.13193968	0.000248	Downregulated
Dpp6	-5.1295553	0.000245	Downregulated
Dscam	-5.12798726	0.000255	Downregulated
Dytn	-5.12335361	0.047095	Downregulated
Mir6399	-5.0968447	0.029353	Downregulated
Stac	-5.09365663	0.000533	Downregulated
Gm48871	-5.0358498	0.000636	Downregulated
Rxfp1	-5.00123382	0.000631	Downregulated
3425401B19Rik	-4.98214822	0.014587	Downregulated
Frem1	-4.95677767	0.003841	Downregulated
Gm16372	-4.95239322	0.035086	Downregulated
Akap6	-4.92430689	0.000498	Downregulated
Sntg1	-4.92329916	0.000505	Downregulated
Astn1	-4.92276178	0.000511	Downregulated
Chil5	-4.90062481	0.009472	Downregulated
Mycbpap	-4.88290458	0.005561	Downregulated
Gm4813	-4.86581478	0.010345	Downregulated

Gm2106	-4.86523243	0.018891	Downregulated
mt-Ty	-4.85916462	0.000251	Downregulated
Alox12e	-4.84730956	0.01921	Downregulated
Pkd1l2	-4.82125785	0.025147	Downregulated
Plpp7	-4.81896488	0.000799	Downregulated
Grm5	-4.80668987	0.000766	Downregulated
A830009L08Rik	-4.80408868	0.001993	Downregulated
BC034090	-4.79462434	0.00096	Downregulated
Corin	-4.77937175	0.001548	Downregulated
Cnmd	-4.76762729	0.002203	Downregulated
Sfta3-ps	-4.73384696	0.00988	Downregulated
Cbx3-ps6	-4.7225902	0.013126	Downregulated
Umodl1	-4.71958512	0.025188	Downregulated
D630023F18Rik	-4.69659582	0.001487	Downregulated
Stum	-4.68286834	0.001163	Downregulated
Nav3	-4.68232875	0.001141	Downregulated
Lrp1b	-4.68171835	0.001153	Downregulated
Clstn2	-4.68115264	0.001165	Downregulated
Gabrg3	-4.68115111	0.001165	Downregulated
Slc6a17	-4.68060933	0.001176	Downregulated
B230334C09Rik	-4.67758596	0.001241	Downregulated
Ppp1r1b	-4.67514925	0.001298	Downregulated
Gm13446	-4.66345088	0.001642	Downregulated
Dcdc5	-4.64063729	0.002955	Downregulated
Xntrpc	-4.62163283	0.009887	Downregulated
Scn10a	-4.61723582	0.038139	Downregulated
Tpo	-4.60454768	0.015045	Downregulated
Ptprt	-4.60377566	0.020313	Downregulated
1700001G11Rik	-4.60296309	0.01508	Downregulated
Olfr78	-4.59770648	0.025035	Downregulated
Csmd2	-4.59382891	0.035198	Downregulated
Ccl27a	-4.58810638	0.001115	Downregulated
Kcnq2	-4.57222294	0.025981	Downregulated
Gm44144	-4.56874691	0.002827	Downregulated
Gm31107	-4.55020812	0.00821	Downregulated
Csrnp3	-4.54593218	0.001748	Downregulated
Shc3	-4.54475567	0.001826	Downregulated
Negr1	-4.54472329	0.001784	Downregulated
Usp13	-4.54447574	0.001792	Downregulated
A930017M01Rik	-4.54230727	0.001868	Downregulated
Gm9801	-4.54019695	0.001933	Downregulated
Kcng1	-4.53772763	0.002099	Downregulated
Col11a1	-4.52764914	0.002469	Downregulated
Nkd2	-4.52105751	0.002855	Downregulated
Gm49192	-4.51893636	0.014879	Downregulated
Otx1	-4.51867069	0.003018	Downregulated

Lama1	-4.51333087	0.003444	Downregulated
Myo16	-4.5115585	0.003606	Downregulated
Tdrd6	-4.50683191	0.004104	Downregulated
Gm5535	-4.50525487	0.004293	Downregulated
Gm47950	-4.49550502	0.01092	Downregulated
Gm20535	-4.49547605	0.019495	Downregulated
Gm42597	-4.49094695	0.006873	Downregulated
Gm26756	-4.48230534	0.009762	Downregulated
A330015K06Rik	-4.47278503	0.004951	Downregulated
Plb1	-4.46710392	0.021797	Downregulated
Gm9768	-4.46233303	0.014724	Downregulated
Piezo2	-4.43881715	0.012504	Downregulated
Sox18	-4.4323449	0.003239	Downregulated
Rian	-4.43130846	0.001765	Downregulated
ENSMUSG00002076530	-4.42779419	0.010646	Downregulated
Fam110d	-4.41045178	0.006786	Downregulated
Doc2b	-4.39570075	0.00282	Downregulated
Fibcd1	-4.39452118	0.003347	Downregulated
Lsamp	-4.39411571	0.0028	Downregulated
Mapk10	-4.3934087	0.002834	Downregulated
Syt4	-4.39327144	0.002841	Downregulated
Slc17a7	-4.39271933	0.002868	Downregulated
Kcnab1	-4.39143416	0.002932	Downregulated
A830018L16Rik	-4.39046704	0.002982	Downregulated
Cyp46a1	-4.38960622	0.003028	Downregulated
Pde9a	-4.38869577	0.003165	Downregulated
Cd109	-4.38337282	0.003527	Downregulated
Srd5a1	-4.38050335	0.00377	Downregulated
Synpr	-4.3756541	0.003958	Downregulated
Gm48743	-4.37240094	0.004805	Downregulated
Scube2	-4.37151552	0.005832	Downregulated
Sostdc1	-4.36628952	0.007322	Downregulated
Avp	-4.33807495	0.01655	Downregulated
9330158H04Rik	-4.336178	0.011904	Downregulated
Baiap2l2	-4.33538328	0.019129	Downregulated
Cilp2	-4.32637811	0.033682	Downregulated
Ttc6	-4.31922675	0.025445	Downregulated
Egflam	-4.31491737	0.032414	Downregulated
Tnni3k	-4.30879585	0.048026	Downregulated
3110067C02Rik	-4.27620435	0.018692	Downregulated
Mir100hg	-4.25532669	0.003109	Downregulated
Mgat4c	-4.25145183	0.003263	Downregulated
D330050G23Rik	-4.23726123	0.007095	Downregulated
Fbxl21	-4.23719304	0.005155	Downregulated
Cdh10	-4.22472424	0.004814	Downregulated
Ntm	-4.22436552	0.004643	Downregulated

Nalcn	-4.22420984	0.004655	Downregulated
Brinp3	-4.2238049	0.004687	Downregulated
Igsf11	-4.22337597	0.004721	Downregulated
Lrfn5	-4.22315668	0.004739	Downregulated
Slc8a3	-4.22281928	0.004766	Downregulated
Iqsec3	-4.22251203	0.004791	Downregulated
Shisa4	-4.22231583	0.004807	Downregulated
Igdcc4	-4.22211317	0.004823	Downregulated
Gm35040	-4.22187509	0.004843	Downregulated
Daam2	-4.22187038	0.004843	Downregulated
Kndc1	-4.22163886	0.004863	Downregulated
Matn4	-4.21974791	0.005053	Downregulated
Fut9	-4.21972821	0.005027	Downregulated
Tfap2b	-4.21859764	0.005128	Downregulated
Galntl6	-4.21774074	0.005207	Downregulated
Unc13c	-4.21718198	0.005259	Downregulated
Krt77	-4.21561765	0.005411	Downregulated
Fam107a	-4.21499122	0.005682	Downregulated
Gm26777	-4.21416807	0.00667	Downregulated
2900027M19Rik	-4.21375793	0.005599	Downregulated
Etv4	-4.21355592	0.005621	Downregulated
Kcns3	-4.21031224	0.005979	Downregulated
Adamts18	-4.20543252	0.006591	Downregulated
Glp1r	-4.20464577	0.006699	Downregulated
Clec18a	-4.20266648	0.006983	Downregulated
Mei1	-4.20176567	0.007118	Downregulated
Rbp4	-4.19940301	0.007493	Downregulated
Ptges3l	-4.18724064	0.011365	Downregulated
Gm45470	-4.18479	0.010688	Downregulated
Gm38187	-4.18261749	0.011339	Downregulated
Tmprss5	-4.17655479	0.013509	Downregulated
Gm12522	-4.1730982	0.01504	Downregulated
Rpl29-ps2	-4.16849501	0.040292	Downregulated
Rab3c	-4.16836516	0.003816	Downregulated
Clmp	-4.16393812	0.003993	Downregulated
Lypd1	-4.15902693	0.032276	Downregulated
Nfatc4	-4.15113274	0.035288	Downregulated
Dmkn	-4.14853256	0.040062	Downregulated
Meltf	-4.1483199	0.040477	Downregulated
4930568G15Rik	-4.11914644	0.024001	Downregulated
Gm10801	-4.10878366	0.002252	Downregulated
Mir7212	-4.07575213	0.035063	Downregulated
Cfap65	-4.0743175	0.032189	Downregulated
Trhr2	-4.06930598	0.009759	Downregulated
Drd1	-4.06668619	0.00555	Downregulated
Tmco5	-4.05203011	0.022464	Downregulated

Gm29340	-4.04744017	0.012835	Downregulated
Vmn2r53	-4.04285439	0.018114	Downregulated
Hepacam	-4.03453208	0.008198	Downregulated
Nhs	-4.0331516	0.008136	Downregulated
Abcc8	-4.03134238	0.008758	Downregulated
Csmd3	-4.03096174	0.008133	Downregulated
Myt1	-4.03076263	0.008309	Downregulated
Sox11	-4.03063271	0.008272	Downregulated
Slitrk1	-4.03062529	0.008178	Downregulated
Pcdhb16	-4.03060612	0.008344	Downregulated
Adra1b	-4.0301798	0.008539	Downregulated
Rab15	-4.03017253	0.00824	Downregulated
Nrxn3	-4.0299199	0.008275	Downregulated
Cntnap5b	-4.02977947	0.008295	Downregulated
Ncan	-4.02963294	0.008375	Downregulated
Zfp423	-4.02872048	0.008443	Downregulated
Tmem200a	-4.02851527	0.008473	Downregulated
Nkain3	-4.02830895	0.008503	Downregulated
Ptprn2	-4.02802992	0.008543	Downregulated
Sema3e	-4.02781978	0.008574	Downregulated
S100b	-4.0274489	0.008895	Downregulated
Zfp786	-4.0268236	0.008722	Downregulated
2810459M11Rik	-4.02669478	0.008741	Downregulated
A330008L17Rik	-4.02589886	0.008862	Downregulated
Six3	-4.02583789	0.009517	Downregulated
A2m	-4.02581952	0.008874	Downregulated
Zic5	-4.02468621	0.009052	Downregulated
Hhatl	-4.0242953	0.009114	Downregulated
Gm44022	-4.02163973	0.009558	Downregulated
Gm5067	-4.01623718	0.010571	Downregulated
Cda	-4.01314731	0.016019	Downregulated
Igdcc3	-4.01083042	0.013088	Downregulated
Gm12122	-4.00439877	0.015671	Downregulated
Slc6a5	-4.00110714	0.018976	Downregulated
Dlx6os1	-3.99739877	0.015814	Downregulated
Gpr179	-3.99737269	0.01578	Downregulated
Gm37894	-3.99717411	0.0159	Downregulated
Calcr	-3.99566101	0.016492	Downregulated
Slc2a10	-3.99483124	0.016831	Downregulated
1110002E22Rik	-3.99474563	0.016866	Downregulated
Lncenc1	-3.99338245	0.017449	Downregulated
Gm23995	-3.99320829	0.036988	Downregulated
Col24a1	-3.99196567	0.018085	Downregulated
Wif1	-3.99181297	0.018157	Downregulated
Frmpd2	-3.99179217	0.018166	Downregulated
Lif	-3.99125355	0.019812	Downregulated

Erich6	-3.98993516	0.019063	Downregulated
Esyt3	-3.98840444	0.019853	Downregulated
Gm48996	-3.98569628	0.026086	Downregulated
Gm29508	-3.98514209	0.02171	Downregulated
Cd63-ps	-3.98339211	0.028339	Downregulated
AU022754	-3.98289758	0.023143	Downregulated
Gm10717	-3.98004081	0.039204	Downregulated
Gm45496	-3.97867321	0.026251	Downregulated
Trp53cor1	-3.97580206	0.028731	Downregulated
Otx2os1	-3.97460592	0.029867	Downregulated
Klhl35	-3.97249276	0.03695	Downregulated
B4galnt3	-3.96775867	0.037838	Downregulated
Spag16	-3.96613388	0.040183	Downregulated
Tomm6os	-3.96332927	0.044763	Downregulated
Unc79	-3.96223002	0.006735	Downregulated
Gm5454	-3.96169841	0.047787	Downregulated
Gm9530	-3.96165715	0.047865	Downregulated
Myt1l	-3.96147749	0.006792	Downregulated
Fgfbp1	-3.96101508	0.049136	Downregulated
Kcnq3	-3.96075402	0.006855	Downregulated
Faim2	-3.95955551	0.006942	Downregulated
Dpyd	-3.95708459	0.007164	Downregulated
Plch2	-3.95506561	0.00732	Downregulated
Mir7052	-3.93245992	0.048629	Downregulated
Gm26555	-3.93174264	0.047953	Downregulated
Gm37541	-3.92625982	0.043274	Downregulated
Gm5814	-3.89698783	0.020722	Downregulated
Nkpd1	-3.87796439	0.030946	Downregulated
A330094K24Rik	-3.87000859	0.038642	Downregulated
Zfp973	-3.86681517	0.047083	Downregulated
Gcat	-3.86410157	0.020098	Downregulated
Gm19196	-3.86163967	0.034853	Downregulated
Gm44907	-3.85950003	0.022359	Downregulated
Cdr1os	-3.84583591	0.009474	Downregulated
Prkar1b	-3.84346167	0.009522	Downregulated
Dscaml1	-3.84097064	0.009803	Downregulated
A730098A19Rik	-3.83973117	0.042752	Downregulated
Gm3764	-3.83552595	0.010476	Downregulated
Fsip1	-3.83149927	0.034231	Downregulated
Gm43508	-3.8279883	0.016507	Downregulated
Slc38a3	-3.81209003	0.014653	Downregulated
Cdh8	-3.81019982	0.01465	Downregulated
Dok5	-3.81004107	0.015217	Downregulated
Rem2	-3.80989759	0.015778	Downregulated
Grid1	-3.80957866	0.014614	Downregulated
Dcc	-3.80956582	0.01462	Downregulated

Arfgef3	-3.80948758	0.01459	Downregulated
Lin7a	-3.80902007	0.01467	Downregulated
Gm37805	-3.80900908	0.020789	Downregulated
Plcxd3	-3.80895923	0.014634	Downregulated
Enho	-3.80883242	0.01496	Downregulated
Pnmal1	-3.8086412	0.014708	Downregulated
Ajap1	-3.8084798	0.014747	Downregulated
Tmem178	-3.80840628	0.014764	Downregulated
Nyap2	-3.80821189	0.01481	Downregulated
Frrs1l	-3.80818256	0.014816	Downregulated
Tmem74	-3.80812509	0.014831	Downregulated
Lmo3	-3.80803181	0.014853	Downregulated
Pcdha12	-3.80761645	0.015519	Downregulated
Pcdhb20	-3.80738188	0.015131	Downregulated
Doc2a	-3.80737014	0.015013	Downregulated
Pcsk2	-3.80715703	0.015064	Downregulated
Tenm3	-3.80694608	0.015179	Downregulated
Zkscan2	-3.80692806	0.015121	Downregulated
Mro	-3.80657717	0.015207	Downregulated
Masp1	-3.80620308	0.015301	Downregulated
Pou3f1	-3.806161	0.015311	Downregulated
3632454L22Rik	-3.80562348	0.016508	Downregulated
Zfp133-ps	-3.8055112	0.01645	Downregulated
Pcdhb14	-3.80500635	0.015605	Downregulated
Kirrel2	-3.80442397	0.015756	Downregulated
Vmn2r84	-3.80434332	0.016231	Downregulated
Slc35f4	-3.80422826	0.015808	Downregulated
Pigz	-3.80246449	0.016283	Downregulated
Cdh15	-3.80121813	0.018847	Downregulated
Lmcd1	-3.80023485	0.017148	Downregulated
Gm15606	-3.80001509	0.016981	Downregulated
Acot4	-3.79977879	0.017051	Downregulated
Gm11855	-3.79934395	0.017181	Downregulated
Gm43789	-3.79876589	0.017355	Downregulated
Gm37928	-3.798758	0.017357	Downregulated
Vmn2r85	-3.79819609	0.01753	Downregulated
Gm14413	-3.79716065	0.01859	Downregulated
9630014M24Rik	-3.7965002	0.018066	Downregulated
Prcd	-3.79498762	0.02013	Downregulated
Gm43175	-3.79407885	0.018876	Downregulated
Pla2g4e	-3.7935645	0.019056	Downregulated
Gm44021	-3.7928973	0.019292	Downregulated
Gm37818	-3.78476695	0.022576	Downregulated
B430319G15Rik	-3.78467078	0.024775	Downregulated
Slc9a4	-3.78330205	0.02742	Downregulated
Kcp	-3.78240355	0.023691	Downregulated

Efna1	-3.78092503	0.016097	Downregulated
4921539H07Rik	-3.77969325	0.025075	Downregulated
Tdgf1	-3.7768325	0.043911	Downregulated
Gm42981	-3.77669759	0.026753	Downregulated
Gm29674	-3.77650514	0.026865	Downregulated
Gm45737	-3.77223434	0.044792	Downregulated
Gm49942	-3.77113587	0.030348	Downregulated
Ido1	-3.76950244	0.031543	Downregulated
Col9a1	-3.76939415	0.031625	Downregulated
Gm10710	-3.76572908	0.034585	Downregulated
Gm4875	-3.76536321	0.049644	Downregulated
Dll4	-3.76513463	0.035103	Downregulated
Mettl24	-3.76422916	0.035917	Downregulated
Rps27a-ps3	-3.75813653	0.021218	Downregulated
Erich2	-3.75810231	0.042228	Downregulated
En1	-3.75759657	0.042814	Downregulated
Gm41836	-3.75645422	0.044206	Downregulated
4930507D05Rik	-3.75619531	0.044529	Downregulated
Gm49077	-3.75544518	0.04548	Downregulated
Il22b	-3.75234326	0.049772	Downregulated
D630045J12Rik	-3.72101796	0.012822	Downregulated
Chp2	-3.70313739	0.009441	Downregulated
Mab21l2	-3.69121389	0.018825	Downregulated
C030005K06Rik	-3.67827884	0.029944	Downregulated
Gm10722	-3.67802883	0.012077	Downregulated
Gm10718	-3.64082451	0.002759	Downregulated
mt-Te	-3.63820987	0.004565	Downregulated
Gm43690	-3.61065917	0.046759	Downregulated
Pax3	-3.59007947	0.024736	Downregulated
Shisa2	-3.58967377	0.018978	Downregulated
Tmem144	-3.58839528	0.018505	Downregulated
2310040G24Rik	-3.58792675	0.034861	Downregulated
Kcnmb4os2	-3.58709767	0.03424	Downregulated
Kcnma1	-3.58334005	0.01794	Downregulated
Srgap1	-3.58178598	0.018247	Downregulated
Gfra4	-3.58142149	0.018538	Downregulated
Rnft2	-3.58035696	0.018872	Downregulated
Aifm3	-3.580315	0.018547	Downregulated
Spink10	-3.57980965	0.04186	Downregulated
Rasgef1c	-3.57698258	0.019263	Downregulated
Gm5628	-3.57473549	0.035149	Downregulated
Grhl3	-3.57297628	0.04588	Downregulated
Sorbs2	-3.57009449	1.92E-05	Downregulated
Gm7901	-3.56573399	0.035427	Downregulated
Kcnq4	-3.56417888	0.022877	Downregulated
Gm10800	-3.56266932	0.003527	Downregulated

Htr4	-3.55297031	0.02816	Downregulated
Gm44883	-3.55242839	0.033592	Downregulated
Grin3a	-3.54751813	0.028096	Downregulated
Cdh18	-3.54655286	0.027708	Downregulated
Foxb1	-3.54647873	0.036281	Downregulated
Pcdh9	-3.54642225	0.027762	Downregulated
St8sia3	-3.5462493	0.027875	Downregulated
Hecw1	-3.54619053	0.027859	Downregulated
Lnx1	-3.54617569	0.02813	Downregulated
Tmem178b	-3.54611697	0.02789	Downregulated
Grik2	-3.5460787	0.027907	Downregulated
Kcna1	-3.54604526	0.027969	Downregulated
Jph1	-3.54596089	0.027957	Downregulated
Slitrk3	-3.54594186	0.027965	Downregulated
Phkg1	-3.5458895	0.028641	Downregulated
Lrtm2	-3.54583393	0.02836	Downregulated
Nrg3	-3.545823	0.028015	Downregulated
Phyhipl	-3.54582293	0.028015	Downregulated
Mlip	-3.54580668	0.028717	Downregulated
Slc13a5	-3.54577779	0.028171	Downregulated
Prkg2	-3.54567228	0.02808	Downregulated
Dgkb	-3.54565771	0.028854	Downregulated
Hmgcll1	-3.54565314	0.028088	Downregulated
Galnt18	-3.54562074	0.02836	Downregulated
Bok	-3.54560061	0.02811	Downregulated
Pcdhac2	-3.54556722	0.028125	Downregulated
Sorcs3	-3.54555928	0.028128	Downregulated
Eef1a2	-3.54545269	0.028172	Downregulated
Sgpp2	-3.5453684	0.02821	Downregulated
Svop	-3.54535887	0.028213	Downregulated
Cntn6	-3.54531601	0.028312	Downregulated
Actl6b	-3.54529634	0.028241	Downregulated
Snhg11	-3.54525314	0.028339	Downregulated
St6gal2	-3.54519085	0.028286	Downregulated
Cdh7	-3.54510631	0.028522	Downregulated
Cbln2	-3.54506978	0.028338	Downregulated
Cux2	-3.54506148	0.028341	Downregulated
Slc7a10	-3.54504743	0.028348	Downregulated
Msi1	-3.54498119	0.028376	Downregulated
Shisa6	-3.54482857	0.028443	Downregulated
Tafa2	-3.54482755	0.028443	Downregulated
Pcdh11x	-3.54472565	0.028487	Downregulated
Zfp804a	-3.54446382	0.028602	Downregulated
Atp13a5	-3.54437065	0.028917	Downregulated
Fam189a2	-3.54390321	0.029171	Downregulated
Mfsd2a	-3.54368183	0.029293	Downregulated

Nkd1	-3.54361674	0.029133	Downregulated
A930004D18Rik	-3.54344004	0.029221	Downregulated
Gabra5	-3.54323023	0.029151	Downregulated
Frmd3	-3.54299608	0.031552	Downregulated
Agt	-3.54258724	0.029647	Downregulated
Vwa5b2	-3.54235223	0.029551	Downregulated
Sox1ot	-3.54221278	0.029615	Downregulated
Gm50462	-3.54219259	0.029625	Downregulated
Xkr7	-3.54208187	0.029676	Downregulated
Chrna4	-3.54190063	0.02976	Downregulated
Oprk1	-3.54182974	0.029793	Downregulated
B230206I08Rik	-3.54172195	0.029844	Downregulated
Eva1a	-3.54171452	0.029847	Downregulated
Pcdhga1	-3.54100328	0.033863	Downregulated
Gm38260	-3.54041967	0.030462	Downregulated
Nsun7	-3.54038807	0.030477	Downregulated
Gm48542	-3.54034144	0.0305	Downregulated
Gm37345	-3.53843537	0.031443	Downregulated
Gm37051	-3.53839363	0.031463	Downregulated
Gm46123	-3.53831147	0.031505	Downregulated
Ecel1	-3.53781401	0.031758	Downregulated
Gm45516	-3.53770716	0.031813	Downregulated
Gm37069	-3.5376407	0.031848	Downregulated
Gm49411	-3.53704043	0.03216	Downregulated
Gm43953	-3.53703351	0.032164	Downregulated
1700086D15Rik	-3.53626854	0.032568	Downregulated
Gm42443	-3.53616727	0.036273	Downregulated
Gm16268	-3.53532096	0.042288	Downregulated
Gm28756	-3.53392452	0.033854	Downregulated
B130024G19Rik	-3.53386121	0.03389	Downregulated
Fibin	-3.53294459	0.034415	Downregulated
Gm37984	-3.53277863	0.034512	Downregulated
Pgf	-3.53260304	0.037984	Downregulated
Capsl	-3.53193169	0.04911	Downregulated
Gm45200	-3.53183858	0.035066	Downregulated
Sfrp5	-3.53134913	0.035359	Downregulated
Gm37393	-3.53129843	0.045141	Downregulated
Oxtr	-3.53105253	0.035538	Downregulated
Slc26a7	-3.53018621	0.036072	Downregulated
Gm49937	-3.52926977	0.036648	Downregulated
Slc13a4	-3.52851165	0.037134	Downregulated
Gm45257	-3.52828846	0.037281	Downregulated
Abhd12b	-3.52351889	0.042348	Downregulated
Pitx2	-3.51793724	0.045084	Downregulated
Gm23787	-3.51756726	0.045406	Downregulated
Slc5a5	-3.51655969	0.046302	Downregulated

9530036O11Rik	-3.51636729	0.046476	Downregulated
Cyp3a13	-3.51589357	0.04691	Downregulated
Optc	-3.51525559	0.047503	Downregulated
Pax7	-3.51281987	0.049868	Downregulated
Mir3473g	-3.49509184	0.033987	Downregulated
Cpxm2	-3.44388807	0.029037	Downregulated
Kcnj6	-3.43267953	0.025653	Downregulated
Fstl4	-3.42973182	0.026286	Downregulated
Wasf3	-3.42914914	0.026403	Downregulated
Pcdhga3	-3.42868131	0.027536	Downregulated
Vwc2	-3.42600327	0.027273	Downregulated
Col4a1	-3.41663665	0.000258	Downregulated
Zfp469	-3.41567432	0.027611	Downregulated
Pcdh17	-3.41539417	0.000226	Downregulated
Aox3	-3.41450828	0.049384	Downregulated
mt-Tr	-3.39217583	0.040708	Downregulated
Wnk2	-3.36665018	0.010669	Downregulated
Dnah14	-3.3427711	0.044829	Downregulated
Rnu12	-3.31030052	5.40E-05	Downregulated
Jakmip2	-3.26210912	0.037455	Downregulated
Pknox2	-3.26205547	0.03772	Downregulated
Ano4	-3.26171252	0.037607	Downregulated
Cacna2d3	-3.2615947	0.037976	Downregulated
Ppfia2	-3.26114155	0.037827	Downregulated
Adamts20	-3.25891735	0.039058	Downregulated
Ccn2	-3.25712955	0.039759	Downregulated
Ccdc13	-3.25185384	0.041794	Downregulated
Gm3739	-3.25139899	0.043114	Downregulated
Esrrb	-3.25005548	0.042657	Downregulated
Gm44220	-3.24020337	0.049805	Downregulated
Snord118	-3.2281513	0.000364	Downregulated
Cntnap3	-3.20016444	0.048885	Downregulated
Gm20544	-3.190043	0.030401	Downregulated
B4galt2	-3.18490995	0.01764	Downregulated
Gm22488	-3.17893088	0.009823	Downregulated
mt-Co3	-3.15355139	0.013853	Downregulated
A4galt	-3.13414299	0.04068	Downregulated
Abi3bp	-3.13080969	0.004048	Downregulated
Gm26109	-3.1114867	0.016485	Downregulated
Pth2r	-3.09785278	0.032812	Downregulated
Gm42568	-3.08030635	0.036314	Downregulated
Dpp10	-3.06909789	0.024674	Downregulated
Cntn4	-3.06780987	0.025157	Downregulated
Ephb3	-3.06062192	0.026407	Downregulated
Gm25813	-2.99268091	0.017925	Downregulated
H1f10	-2.97285249	0.033086	Downregulated

Prkd1	-2.95549091	0.033332	Downregulated
Ccdc148	-2.95519033	0.032742	Downregulated
Hcn1	-2.95492135	0.032693	Downregulated
Sgtb	-2.95368117	0.032933	Downregulated
Grik1	-2.94940399	0.00937	Downregulated
Gm43513	-2.89444683	0.021172	Downregulated
Myo5c	-2.86939896	0.020967	Downregulated
Gm25926	-2.85826447	0.030442	Downregulated
Dcbld1	-2.85042233	0.005057	Downregulated
Eno2	-2.83003501	0.044201	Downregulated
Caln1	-2.82858179	0.044172	Downregulated
Adgrb2	-2.82669636	0.004703	Downregulated
Lhx6	-2.82039473	0.049615	Downregulated
Gm49871	-2.79134234	0.037508	Downregulated
9430060I03Rik	-2.78747093	0.031767	Downregulated
Arhgef15	-2.74167096	0.001363	Downregulated
Gm43628	-2.73852453	0.012324	Downregulated
Egfl7	-2.72980586	0.001555	Downregulated
5_8S_rRNA	-2.69951509	0.002249	Downregulated
Ndrg4	-2.68771512	0.008676	Downregulated
Fbxl13	-2.63391962	0.01836	Downregulated
Gm24265	-2.63350661	0.000192	Downregulated
Gm17017	-2.61565095	0.049744	Downregulated
Zfp697	-2.58935093	0.002509	Downregulated
Efemp2	-2.56658447	0.001496	Downregulated
Igsf9b	-2.53794547	0.016046	Downregulated
Il34	-2.5199069	0.008427	Downregulated
Gm43807	-2.51866792	0.041056	Downregulated
Slco5a1	-2.48957399	0.043964	Downregulated
C130074G19Rik	-2.48810435	0.000861	Downregulated
Fam135b	-2.48745868	0.040283	Downregulated
Apc2	-2.48425499	0.042429	Downregulated
Anks1b	-2.45436724	0.00117	Downregulated
mt-Tp	-2.4194395	0.013824	Downregulated
Mir1955	-2.38811072	0.019678	Downregulated
Snord13	-2.38648241	0.036737	Downregulated
Nrk	-2.35535087	0.01193	Downregulated
Mir6236	-2.32735589	0.047563	Downregulated
Tmod2	-2.31858104	0.005094	Downregulated
Bcl6b	-2.30018712	0.021011	Downregulated
Snord49b	-2.29436177	0.005171	Downregulated
Gm22973	-2.28948344	0.017903	Downregulated
Snora31	-2.28948067	0.02496	Downregulated
mt-Ta	-2.28269304	0.030004	Downregulated
Foxp2	-2.2782032	0.039239	Downregulated
Gm23297	-2.2766164	0.018788	Downregulated

Bnip5	-2.25641819	0.035143	Downregulated
Gm23444	-2.25037821	0.003757	Downregulated
Snord59a	-2.22348925	0.040858	Downregulated
5_8S_rRNA	-2.22265583	0.01553	Downregulated
Gm23472	-2.20334198	0.007009	Downregulated
Bmp7	-2.16339499	0.040558	Downregulated
Gm15444	-2.15364038	0.007284	Downregulated
Unc5b	-2.12579635	0.042637	Downregulated
Dnm3	-2.10878674	0.023805	Downregulated
Gm43627	-2.10772832	0.034384	Downregulated
Snord15a	-2.09660696	0.003556	Downregulated
Kcna6	-2.07976756	0.009689	Downregulated
mt-Tq	-2.06856938	0.04469	Downregulated
Gm24119	-2.0649554	0.007155	Downregulated
Adgrb3	-2.05036489	0.048454	Downregulated
Col4a2	-2.04452545	0.037256	Downregulated
Disp2	-2.03017257	0.041279	Downregulated
Nr1i2	-2.02017954	0.016645	Downregulated
Fzd3	-2.01904771	0.016047	Downregulated
Snora23	-2.0152505	0.012426	Downregulated
Dixdc1	-2.00779476	0.011845	Downregulated
BC051226	-1.98292808	0.014873	Downregulated
Map9	-1.95453812	0.047526	Downregulated
Zfp618	-1.9358605	0.046044	Downregulated
Cadps2	-1.91114024	0.031381	Downregulated
Snora21	-1.90319001	0.041976	Downregulated
Gm26493	-1.86060505	0.028803	Downregulated
Unc5c	-1.84086106	0.030764	Downregulated
Gm37194	-1.80796637	0.02793	Downregulated
Elp6	-1.79713203	0.002735	Downregulated
2810025M15Rik	-1.75944413	0.018173	Downregulated
A630072L19Rik	-1.74313121	0.037159	Downregulated
Reep1	-1.72393903	0.04743	Downregulated
Nsg1	-1.72179125	0.03319	Downregulated
Gm49420	-1.69968901	0.044191	Downregulated
Mtrfr	-1.69650599	0.010266	Downregulated
Borcs5	-1.6650685	0.005504	Downregulated
Gm24407	-1.64969655	0.042651	Downregulated
Gm11175	-1.59217892	0.04504	Downregulated
Gm38366	-1.53329813	0.022438	Downregulated
Sccpdh	-1.49359082	0.039104	Downregulated
Gm26397	-1.28589978	0.048305	Downregulated
Manbal	-1.28348766	0.018994	Downregulated
Mief2	-1.2148535	0.029038	Downregulated
Mydgf	-1.21451881	0.04658	Downregulated
Gm23119	-1.1923229	0.035947	Downregulated

2310015A10Rik	1.39090757	0.010421	Upregulated
Tbc1d19	1.39681375	0.035998	Upregulated
Lzts3	1.52408272	0.04216	Upregulated
Senp8	1.70307933	0.044873	Upregulated
Rundc3a	1.80710829	0.033038	Upregulated
Efnb2	1.81618652	0.033409	Upregulated
Gm43313	1.85210304	0.028376	Upregulated
Gm19721	1.95731539	0.019984	Upregulated
Fam169a	2.04063436	0.029938	Upregulated
Cfp	2.10725383	0.003972	Upregulated
2900005J15Rik	2.17221423	0.043823	Upregulated
Reep6	2.17305428	0.034898	Upregulated
Cfh	2.18419709	0.017394	Upregulated
Myrf	2.21917292	0.039291	Upregulated
Gpd1	2.22887581	0.028463	Upregulated
Dmpk	2.22901221	0.02032	Upregulated
Wfdc17	2.2369093	0.037588	Upregulated
Gm42462	2.29467879	0.025147	Upregulated
1300014J16Rik	2.31957333	0.017749	Upregulated
Hspa12a	2.33768707	0.026869	Upregulated
Gm43857	2.35268134	0.029151	Upregulated
Maoa	2.39005733	0.022163	Upregulated
Gm38235	2.41607604	0.030691	Upregulated
1700086O06Rik	2.484105	0.031623	Upregulated
Nthl1	2.49458313	0.00675	Upregulated
Nmnat2	2.49856916	0.031868	Upregulated
Kcnip2	2.50373699	0.033122	Upregulated
Fgfr1	2.53587317	0.001554	Upregulated
Sdc3	2.56099782	0.0053	Upregulated
Colec12	2.56505362	0.036383	Upregulated
Igfbp6	2.68142957	0.01009	Upregulated
P3h4	2.68862236	0.019004	Upregulated
Gm43145	2.71001724	0.009345	Upregulated
Slc7a2	2.71227638	0.047147	Upregulated
Trpv5	2.72788957	0.016181	Upregulated
Sorbs3	2.728912	0.001661	Upregulated
ENSMUSG00002075338	2.75809401	0.030935	Upregulated
Gm38357	2.76320436	0.033162	Upregulated
Pak6	2.76490349	0.006114	Upregulated
Gm12905	2.76789624	0.034084	Upregulated
Gm25821	2.79197981	0.020593	Upregulated
1700029I15Rik	2.79453833	0.018557	Upregulated
Plxdc2	2.80508749	0.002267	Upregulated
Pdk4	2.84194019	0.026262	Upregulated
Cplane2	2.86427942	0.027505	Upregulated
Mt1	2.87762514	0.034486	Upregulated

E230020D15Rik	2.89503987	0.044264	Upregulated
Osbpl6	2.9399017	0.025991	Upregulated
Garnl3	2.95948568	0.015638	Upregulated
Gm44967	3.01606323	0.028517	Upregulated
Htra1	3.02311707	0.022957	Upregulated
Hmgb1-ps5	3.02999224	0.049885	Upregulated
Ltc4s	3.1454493	0.037957	Upregulated
D630044L22Rik	3.16036509	0.030315	Upregulated
Bco2	3.22239477	0.041864	Upregulated
Ddit4l	3.26898269	0.046144	Upregulated
Fbln2	3.27305567	0.008559	Upregulated
Kcnk3	3.28227518	0.039552	Upregulated
Gm13689	3.28424044	0.048021	Upregulated
Gm48623	3.28447324	0.0383	Upregulated
Irak1bp1	3.28548744	0.038261	Upregulated
Ecm2	3.2854907	0.038993	Upregulated
Frs3	3.28685345	0.039951	Upregulated
Ednra	3.28811117	0.039763	Upregulated
Rgs4	3.28935821	0.038524	Upregulated
1700001L19Rik	3.29118379	0.040513	Upregulated
Ccdc153	3.29216742	0.049889	Upregulated
Gm49968	3.29719994	0.043339	Upregulated
Gm45073	3.30232532	0.044908	Upregulated
Gm13270	3.31147507	0.029779	Upregulated
Slco1c1	3.32150025	0.028265	Upregulated
Snord7	3.33904723	0.024316	Upregulated
Fn1	3.40874978	0.000402	Upregulated
Mpzl2	3.43791604	0.031142	Upregulated
Gm43868	3.46382321	0.04797	Upregulated
Pcdhgb4	3.47089913	0.026922	Upregulated
Apol10a	3.47497446	0.031418	Upregulated
Tnfaip6	3.47615763	0.025582	Upregulated
Enpp2	3.47743226	0.002423	Upregulated
Gm47701	3.47801817	0.027958	Upregulated
Plppr2	3.47978105	0.024758	Upregulated
Pex11a	3.48276978	0.027964	Upregulated
Vgll3	3.48399441	0.029242	Upregulated
Sox2	3.48564322	0.024939	Upregulated
Tsku	3.48705877	0.031629	Upregulated
Il17rc	3.48937286	0.029239	Upregulated
Gm17893	3.49247928	0.045548	Upregulated
Itga11	3.50248588	0.04762	Upregulated
Gm43073	3.50696594	0.044955	Upregulated
Zfp811	3.50763766	0.006418	Upregulated
ENSMUSG00002076189	3.50784934	0.032292	Upregulated
Nbl1	3.52464809	0.018415	Upregulated

AV099323	3.52489439	0.045765	Upregulated
Tgfb2	3.53211686	0.00293	Upregulated
Gm28068	3.54872823	0.031494	Upregulated
Adamtsl5	3.54965633	0.011253	Upregulated
Gm19935	3.55488735	0.047101	Upregulated
Flrt2	3.5962399	0.004677	Upregulated
9530022L04Rik	3.63410335	0.024414	Upregulated
Gjc1	3.64025167	0.018924	Upregulated
Gm12353	3.64593529	0.023753	Upregulated
Zfp37	3.6510458	0.016398	Upregulated
Gm43800	3.65911481	0.019396	Upregulated
Gm48582	3.67418134	0.009767	Upregulated
Myl6b	3.68438903	0.012059	Upregulated
Ocln	3.68889664	0.016295	Upregulated
Hsd17b1	3.71075796	0.041744	Upregulated
Cgnl1	3.73430517	0.00187	Upregulated
Wfikkn1	3.75788958	0.028053	Upregulated
Nhlrc1	3.78890111	0.012443	Upregulated
Fzd1	3.78900019	0.00359	Upregulated
Gm9929	3.7968359	0.038321	Upregulated
2310002F09Rik	3.80584402	0.04487	Upregulated
ENSMUSG00002075672	3.82477807	0.031362	Upregulated
Gm11745	3.82789004	0.024745	Upregulated
Ccdc169	3.8295773	0.023956	Upregulated
Gm48673	3.84399282	0.016294	Upregulated
Gm43365	3.8482896	0.020648	Upregulated
Gm43545	3.87530123	0.019901	Upregulated
Gm39121	3.90631657	0.049746	Upregulated
Ackr3	3.9132137	0.007287	Upregulated
Gm1604a	3.92423177	0.026421	Upregulated
Grem2	3.94014666	0.008125	Upregulated
Gm20125	3.95632655	0.03701	Upregulated
Tex29	3.96424032	0.039624	Upregulated
Ogn	3.97346359	0.010465	Upregulated
Gm12713	3.97599442	0.049819	Upregulated
Gm42633	3.9789773	0.033422	Upregulated
5S_rRNA	4.00945942	0.01473	Upregulated
Gm47765	4.02392841	0.028845	Upregulated
Trarg1	4.03894639	0.034229	Upregulated
C1rl	4.03915317	0.030488	Upregulated
Des	4.0489039	0.046341	Upregulated
Chtf8	4.05697145	0.014414	Upregulated
Prss57	4.05834267	0.037708	Upregulated
Gnao1	4.06379224	0.005455	Upregulated
Gabra3	4.06684885	0.00552	Upregulated
Gdpd2	4.07406514	0.00716	Upregulated

Smtnl2	4.08553992	0.013825	Upregulated
Il33	4.08708434	0.006504	Upregulated
Gm13786	4.10113765	0.014384	Upregulated
Nox1	4.13274389	0.021488	Upregulated
Gm17096	4.1455452	0.012781	Upregulated
Gm37886	4.15907617	0.034476	Upregulated
Gm38215	4.17136899	0.023114	Upregulated
Fgf1	4.18410826	0.003537	Upregulated
Adamts2	4.19455588	0.004585	Upregulated
Mypn	4.20123291	0.018237	Upregulated
Gm42780	4.20182405	0.030928	Upregulated
Gm48639	4.2111689	0.021699	Upregulated
Plaat5	4.23724057	0.042994	Upregulated
Gm42433	4.28838734	0.003158	Upregulated
Pcdhgb8	4.29120306	0.003576	Upregulated
Gm12708	4.29480751	0.035442	Upregulated
Gm22067	4.30232129	0.01288	Upregulated
Tcaf1	4.35512741	0.024636	Upregulated
Hspb8	4.3687084	0.003641	Upregulated
Islr	4.37054458	0.010415	Upregulated
Hyal1	4.38539532	0.003158	Upregulated
Fabp3	4.39123604	0.002393	Upregulated
4833408A19Rik	4.39973941	0.00324	Upregulated
Rpl17-ps9	4.41492202	0.019698	Upregulated
Loxl2	4.51380774	0.018308	Upregulated
Gm20768	4.52789127	0.009636	Upregulated
Efemp1	4.5682893	0.001568	Upregulated
9630028I04Rik	4.621601	0.0129	Upregulated
Junos	4.6270257	0.002102	Upregulated
U1	4.63458815	0.019149	Upregulated
Snord43	4.66773673	0.006155	Upregulated
Aebp1	4.69509628	0.000441	Upregulated
Ednrb	4.70921901	0.007189	Upregulated
Gm9830	4.72360522	0.027379	Upregulated
Gm8850	4.7414533	0.006249	Upregulated
C1qa	4.78597349	0.000328	Upregulated
Mgl2	4.82770695	0.006255	Upregulated
Kcnk13	4.85090342	0.00452	Upregulated
Fcrls	4.90065272	0.002141	Upregulated
Gm5242	5.03916902	0.000778	Upregulated
Mrps36-ps1	5.07255797	0.006686	Upregulated
C1qc	5.10316102	0.000355	Upregulated
Gm47326	5.13785924	0.023739	Upregulated
Ceacam10	5.1994587	0.009808	Upregulated
Cbr2	5.32676787	0.010465	Upregulated
Fcna	5.37537883	0.005997	Upregulated

Gm12279	5.3787699	0.000841	Upregulated
Ptgis	5.6964851	0.004578	Upregulated
C4b	5.91640513	0.000165	Upregulated
Cxcl13	5.94989974	0.006429	Upregulated
Bcam	6.14888097	0.00016	Upregulated
Gata6	6.32635178	0.01919	Upregulated
Ucp1	6.58108592	0.004119	Upregulated
Car3	7.21330467	9.68E-06	Upregulated
Saa3	7.46958827	0.011294	Upregulated
C1qb	7.65202286	1.43E-06	Upregulated
Prg4	7.81210321	1.71E-06	Upregulated
Rag1	8.00900395	4.61E-06	Upregulated
Retnla	8.1192498	0.001081	Upregulated
Cd5l	8.51232257	0.020896	Upregulated

Table of Contents Top of Current Table

Supplemental Table 3. List of all overlap of significant ($P < 0.05$) differentially expressed genes in whole blood of 3xTg-AD vehicle (week 0 to 8) and wild-type B6129 vehicle (week 0 to 8) animals. [&]Commonly downregulated in 3xTg-AD (vehicle only) and B6129 (vehicle & CBD); [#]Commonly downregulated in 3xTg-AD (vehicle & CBD) but upregulated in B6129 (vehicle only); *Commonly upregulated in 3xTg-AD (vehicle & CBD) and B6129 groups (vehicle & CBD); [†]Commonly upregulated in 3xTg-AD (vehicle & CBD) and B6129 (vehicle only); [‡]Commonly upregulated in 3xTg-AD (vehicle only) and B6129 groups (vehicle & CBD); [^]Commonly upregulated in 3xTg-AD (vehicle only) and B6129 (CBD only) but downregulated in B6129 (vehicle only); [+]Commonly upregulated in 3xTg-AD (vehicle only) but downregulated in B6129 (vehicle & CBD); [$]Commonly upregulated in 3xTg-AD (vehicle and CBD) but downregulated in the B6129 (vehicle only).

Common Genes Downregulated	Common Genes Upregulated	Common but Opposite: Downregulated in 3xTg-AD & Upregulated in B6129	Common but Opposite: Upregulated in 3xTg-AD & Downregulated in B6129
C130074G19Rik[&]	Aebp1[†]	ENSMUSG00002075672; Gm54761, miRNA	A830018L16Rik
Daam2	Bcam[†]	5S_rRNA[#]	Cacna2d3
Efna1	C1qa*	9530022L04Rik; lncRNA	Etv4
Egfl7	C1qb*	Frs3	Foxp2
Fam110d[&]	C1qc*	Gm13270; lncRNA	Gm5067; pseudogene of ribosome biogenesis regulatory protein
Gm11175	C4b*	Gm17096	Hhatl
Gm26109	Car3*	Gm17893; pseudogene of nucleoporin 37	Iqsec3
Gm38366	Cbr2[‡]	Gm25821; Snora21	Kcnq4
Gm42568	Ccdc169	Gm42462	Kndc1
Gm43513	Cgnl1[†]		Lin7a
Gm43807	Ddit4l		Lmcd1
Mief2	Des[†]		Lrfn5
Mir1955	Ednrb*		Masp1
Mir421	Efemp1[†]		Mei1
Mir7212	Fabp3[‡]		Myt1l[+]
Rian	Fcna[†]		Ncan
Rnft2	Fcrls		Pcdh9
Sgtb	Flrt2[†]		Pcsk2

Srd5a1	Fn1		Ppfia2
Unc5c	Gm19935[†]		Prkar1b
	Gm9830		Pth2r
	Gpd1*		Rbp4[^]
	Il33[†]		Shisa2[$]
	Kcnk3[†]		Slc13a4
	Ltc4s[†]		Slc17a7
	Mgl2[†]		Snord59a
	Pak6		Spink10
	Pdk4*		Srgap1[+]
	Prg4*		St8sia3
	Ptgis*		
	Retnla*		
	Saa3[†]		
	Trarg1		
	Ucp1*		
	Wfdc17		

Table of Contents Top of Current Table

Supplemental Table 4. List of all differentially expressed genes (P<0.05) in whole blood of 3xTg-AD vehicle (week 0 to 8) that were reversed in expression in wild-type B6129 vehicle (week 0 to 8) animals in comparison. *P<0.05, significant expression in the opposite direction in wild-type B6129; Italicized genes have a recognized association with Alzheimer's disease pathology

DEG (P<0.05): 3xTg-AD	Log2FoldChange: 3xTg-AD	Log2FoldChange: B6129	P-value: B6129
H2-Q2	-16.77560335	2.234356116	0.311528743
Tdrd5	-15.99926249	4.046070353	0.065260045
Gm19552	-4.445383242	1.608224583	0.500672505
ENSMUSG00002075672*; Gm54761; miRNA	-4.314848177	3.82477807	0.031362062
Gm38146	-4.252136409	1.193550892	0.656405102
3110080O07Rik	-4.250073615	1.786161885	0.247101188
Rnf227	-4.234684796	0.176067604	1
Trbj1-7	-4.20364531	1.108669373	0.773532727
2510017J16Rik	-4.197342434	0.226190774	1
Gm5521	-4.180943981	0.653458665	0.968054065
1600022D10Rik	-4.103015179	0.499984725	1
Gm49703	-4.041527897	0.785660322	0.805597774
Mir342	-4.028353097	0.422818764	1
Gm17096*; GM17096 gene product from transcript GM17096-RA	-4.028120351	4.145545202	0.012780639
Trbj1-6	-4.015905086	1.401722533	0.582278601
Gm44975	-3.976284171	0.08474035	1
Gm19427	-3.954553904	2.94215338	0.099789104
Gm8899	-3.930064587	2.449473727	0.314528968
Gm37766	-3.923947967	0.328689894	1
4833412K13Rik	-3.820789576	2.106027268	0.105211304
2900078I11Rik	-3.818910655	1.0993327	0.735342676
Gm37675	-3.815004745	3.462094943	0.054412739
Traj22	-3.797700348	1.297692153	0.683401889
Gm45570	-3.797377352	3.225770645	0.062972156
Gm11725	-3.759144304	1.832416278	0.424256815
Gm26064	-3.758243305	0.934745709	0.746468867
Gm29112	-3.756968302	0.025622229	1
Gm37062	-3.741751314	0.666823822	0.909324418
Gm49169	-3.726783647	0.747332577	0.899154486
Gm38257	-3.71914662	0.509089139	1
A3galt2	-3.713161281	0.124940404	1
Gm29055	-3.707065942	2.456099349	0.251714787
Gm44888	-3.701614023	0.491901489	1
2610028D06Rik	-3.701505661	0.096069292	1
Tamalin	-3.689792539	0.924514018	0.504408663

Gm37509	-3.682914104	1.472234814	0.475286797
Gm16794	-3.676380325	0.543667714	1
Gm29596	-3.660951393	3.553744248	0.086026892
Gm43062	-3.660425205	2.900005887	0.051826134
Gm27184	-3.632675504	1.103859913	0.668339085
Gm8437	-3.607187388	0.08918582	1
Gm42702	-3.603113451	0.640904278	0.958854194
Gm37160	-3.60259912	1.011951101	0.63117542
Gm43075	-3.599343211	3.482701248	0.060878819
Gm37672	-3.59632094	0.802946374	0.825712231
Gm47374	-3.593978179	0.084513247	1
F12	-3.571300097	0.385909553	1
Gm37490	-3.515079271	3.131726482	0.124456583
Gm12902	-3.511693182	3.111338841	0.12664302
1700028J19Rik	-3.490572427	0.587493774	0.998704424
1810012K16Rik	-3.48720857	0.377283334	1
Rasd1	-3.482162783	0.58835838	0.998437547
Synm	-3.478758154	0.192912875	1
D130019J16Rik	-3.474734558	1.273426478	0.477143296
Olfr1393	-3.41338819	3.233180426	0.085667027
Pbx4	-3.40968696	1.688350523	0.141289154
Stmn1-rs1	-3.408496978	1.370417229	0.609725381
Angptl6	-3.408009683	0.060032415	1
Polr2k-ps	-3.391678273	0.851488797	0.841278158
Ppnr	-3.3897507	1.790436979	0.389673772
Iqck	-3.35299091	0.415623778	1
Gm44835	-3.349553731	0.735185503	0.896871492
Gm4787	-3.322714576	0.275332692	1
Tagln3	-3.31669466	0.088675272	1
Gm49086	-3.314785886	0.623673825	0.97208742
Gm14049	-3.309684347	3.498169147	0.053314764
Gm38125	-3.286293074	0.660972849	0.954766917
Gm42632	-3.280540537	1.197757946	0.647272276
Gm17228	-3.275922652	1.416301843	0.487261999
Gm44008	-3.257864209	1.028519184	0.763497475
Gm47113	-3.251054658	2.230787905	0.228527482
Gm37485	-3.234101999	2.080039854	0.362837198
Gm18537	-3.232135581	3.452619394	0.06066097
Gm36501	-3.221979864	0.842772775	0.862280153
Gm43420	-3.217598844	1.420168462	0.403149959
Rin1	-3.210932244	0.583390299	1
Gm15672	-3.203565253	2.528107845	0.260887729
Gm17530	-3.195443498	2.816112646	0.127592054
7SK	-3.187273204	2.82951698	0.190855639

Gm25821*; snoRNA Snora21	-3.185151552	2.791979812	0.02059253
Gm43727	-3.172442133	1.518952827	0.425820884
A130050O07Rik	-3.15877975	1.919949075	0.229920461
Gm15971	-3.130526756	0.442651405	1
Gm43072	-3.12693392	0.639027025	0.951128321
D7Bwg0826e	-3.110811106	2.361054649	0.27468172
Gm17893*; pseudogene, nucleoporin 37	-3.104431581	3.492479278	0.045548261
Gm7666	-3.102724703	1.377875243	0.2105494
Dhrs13	-3.083811334	0.475095638	1
Runx2os1	-3.072827666	1.687439616	0.402840834
Rimkla	-3.067729871	1.149875589	0.391389794
Slc25a2	-3.065875599	0.270934964	1
9530022L04Rik*; lncRNA	-3.056985934	3.634103346	0.024413917
A930007I19Rik	-3.039998876	1.069323929	0.568472129
Gm50387	-3.036843437	3.498210862	0.053491077
Gm15445	-3.034048525	1.810512733	0.420736846
Mamdc2	-3.029147589	2.144759875	0.301245608
Gm45532	-3.018019597	1.629277015	0.463794719
Gm28707	-3.012942755	0.395748251	1
Wnt10a	-3.00898024	0.538207374	1
Gm38376	-3.001811831	1.029354492	0.657058656
Mir6374	-2.984449674	0.980847137	0.796132439
Mmp28	-2.978458893	1.977263357	0.240087467
Acvr1	-2.972012735	0.342748576	1
Ramp3	-2.955000191	0.988033195	0.45080112
6430710M23Rik	-2.952664441	0	1
Gm45234	-2.950745862	0.575651886	1
7SK	-2.944865848	2.722935261	0.070118868
Gm45534	-2.941916887	1.156162402	0.573439558
Gm47615	-2.933650581	0.086562405	1
Pcdhga9	-2.926133701	0.896221213	0.692814578
Gm38142	-2.919226574	0.690139885	0.92000912
Gm44041	-2.915583698	0.17688872	1
Gm17191	-2.911586128	2.562993762	0.089757147
Gm37132	-2.904055473	0.396912151	1
Lbhd1	-2.889516774	1.13603329	0.405203766
Gm49413	-2.879892628	0.655054517	0.939172968
Gm15538	-2.869564726	1.792280377	0.354708659
Nscme3l	-2.849133853	2.126703641	0.20024422
Mettl5os	-2.840022955	0	1
Gm13868	-2.838175041	1.454935218	0.603604777
A530064N14Rik	-2.816476227	0.423540741	1

Gm37537	-2.80971377	1.209239922	0.545317797
Igkv3-12	-2.799023497	0.04058399	1
Gm13270*; lncRNA	-2.7696026	3.311475068	0.029779006
Fth-ps2	-2.755194004	2.474913867	0.125288918
Map2k3os; lncRNA	-2.741948821	0.74374656	0.835143442
Gm42872	-2.739367784	1.221738973	0.537754344
Tox3	-2.736388812	1.832467673	0.357730093
Gm15545	-2.711033131	0.00710562	1
6820402A03Rik	-2.704471181	0.8797604	0.790223021
Gm38262	-2.700925191	1.211956278	0.618962594
Gm42867	-2.675919367	0.772813534	0.845763233
Gm44053	-2.670281355	1.161758976	0.527319037
Gm37115	-2.657646848	0.076511393	1
Gm44695	-2.62275371	0.415918488	1
C430019N01Rik	-2.62138139	1.462901998	0.327403505
Gm42883	-2.610186398	1.513834934	0.284423496
Ptk7	-2.594370101	0.512915516	1
Gm48701	-2.591470364	0.046326741	1
7SK	-2.589980134	0.025225582	1
Gm36933	-2.57848936	0.102094975	1
Gm43571	-2.551881633	0.183377099	1
Frs3*	-2.526939977	3.286853452	0.039951213
Gm37390	-2.486996548	1.881224226	0.189004308
Gm38346	-2.486798961	0.339017951	1
Gm37465	-2.479224664	0.11782289	1
Gm31024	-2.47566946	1.322489185	0.215560719
Gm48139	-2.474412817	0.692309927	0.902495756
Gm47246	-2.454813779	0.461980386	1
Gm49783	-2.453779994	0.314962764	1
4930556H04Rik	-2.379660011	0.256631783	1
Ighj2	-2.379523695	0.628953249	0.951479394
Wfs1	-2.329456394	0.699208546	0.84061096
Crip3	-2.284204663	1.023346339	0.658214628
Gm42462*	-2.279389068	2.294678793	0.02514737
Gm38034	-2.279249381	0.160858357	1
Gm37139	-2.237391437	0.186592841	1
Rgs11	-2.225623827	0.281690857	1
Gm43099	-2.160926104	0.128568203	1
Tmie	-2.15519489	0.238037658	1
4833421G17Rik	-2.152863723	0.812019173	0.776780207
Gm47664	-2.148453848	1.302550061	0.380032533
5930430L01Rik	-2.126354054	0.722748662	0.86012419
Gm49396	-2.102784354	0.512635162	1
Nr6a1	-2.069403506	0.438448887	1

Zfp775	-2.068555033	0.654448762	0.867571991
Cacna2d4	-2.054187887	0.120605984	1
Gm36931	-2.042661034	0.619338077	0.953913507
Gm36527	-2.040345083	1.168643533	0.330720762
Gm37053	-2.020598091	0.047138567	1
2700062C07Rik	-2.01012105	0.220591657	1
Gm44033	-2.009664786	0.49084251	1
Gm37176	-2.009515081	0.604764117	0.978127036
Gm37524	-2.007980894	0.716137662	0.892504852
Gm37019	-1.971132956	0.008609393	1
Bach2it1	-1.966283782	0.172953325	1
Gm43721	-1.940579243	0.000250989	1
Gm44027	-1.936316712	0.133195127	1
Gm43196	-1.930987277	0.093954371	1
Ust	-1.930979154	0.673877629	0.794311952
Gm11400	-1.899734574	0.410036539	1
Gm38220	-1.880057065	0.427549622	1
Gm28373	-1.86668924	0.357977913	1
Gm17690	-1.852892955	0.079262228	1
Esr1	-1.851838849	0.218764177	1
Gm49839	-1.84462461	0.770409092	0.812609215
Gm20696	-1.836034741	0.743686772	0.752997095
C130089K02Rik	-1.782519983	0.243846299	1
Gm44891	-1.776950859	1.099346883	0.441733123
Gm45342	-1.757027947	1.856146571	0.066463241
5330406M23Rik	-1.732665459	0.902993042	0.529105206
Gm37159	-1.730857284	0.480656788	1
Gm44153	-1.718510833	1.754878019	0.077007949
Gm43274	-1.706224361	0.179510087	1
Gm29488	-1.699074466	0.770182741	0.778831707
Gm44745	-1.663278982	0.986393704	0.519016944
Gm37943	-1.66012451	0.850593824	0.645369368
Zfp28	-1.654801514	0.07660056	1
Gm43457	-1.641368781	0.261659268	1
Fam167a	-1.608653944	0.0396204	1
C230085N15Rik	-1.605467642	0.996535452	0.484093703
Dph2	-1.569163326	0.216031641	1
Il18bp	-1.537571621	0.40246144	1
Gm37593	-1.520024982	0.322243182	1
Gm37296	-1.511051603	0.610806426	0.955900542
5330432J10Rik	-1.481921667	0.744308612	0.767969042
Gm15675	-1.481392862	0.081587618	1
Acsf3	-1.405780036	0.004381534	1
Zfp157	-1.384623573	0.081989575	1

Gm6712	-1.372660705	1.226303462	0.100547573
Abcc10	-1.362548676	0.306841155	1
Gprc5b	-1.362209364	0.315752937	1
Rai1	-1.211879012	0.005483918	1
Dph3	1.015682637	-0.202221832	1
mt-Nd6	1.08477013	-0.101871919	1
Fam151b	1.275871117	-0.067517298	1
Cplx2	1.461327138	-0.449054819	1
Naa38	1.47537019	-0.091029989	1
Zcchc14	1.542994165	-0.81240484	0.662078345
Kitl	1.679825767	-0.30226572	1
Gm5617	1.728537103	-0.208676684	1
Asxl3	1.737109699	-1.246924746	0.214361594
Fam174c	1.739385286	-1.051380116	0.386008798
Gm40578	1.82005248	-1.371783281	0.36141593
Tmem91	1.827332523	-1.097526919	0.45882549
1700007L15Rik	1.917701178	-0.657767654	0.920482437
Rgs8	1.951258432	-0.375995762	1
Tmem202	1.957204249	-0.827541104	0.728428482
Tm4sf1	2.073051007	-0.163921478	1
Lpar1	2.116306621	-0.374600218	1
Fam13a	2.121505864	-1.01158123	0.493655374
Gls2	2.166357672	-0.449444515	1
Snord59a*; snoRNA	2.208484186	-2.223489252	0.040857815
Grhl1	2.252646301	-0.379144977	1
Ephb4	2.279746766	-0.601416914	0.979158719
Enpp3	2.284956173	-0.05722285	1
Parva	2.318821291	-1.303518075	0.185500843
Adamts17	2.321310853	-1.606023318	0.321680771
BC065403	2.341190906	-0.075645071	1
Plekhh2	2.341788579	-2.243107948	0.082512134
Sdc2	2.395347845	-1.89997277	0.073565678
Rgs22	2.401114393	-0.35632351	1
Cabcoco1	2.417215228	-0.76917228	0.860969833
Tmem88b	2.494270634	-0.957152781	0.778651218
Ica1l	2.498389246	-0.411916373	1
Calr3	2.516918922	-1.708726862	0.235934319
Lag3	2.519540916	-0.778987392	0.816092382
Sytl5	2.578519696	-0.651678417	0.94997968
Islr2	2.588293913	-0.213344396	1
Gm47493	2.620603191	-3.51294097	0.053238524
Thbs2	2.623639538	-1.695219995	0.135471838
Mroh8	2.625219635	-0.351791905	1
6530409C15Rik	2.65190834	-1.773170436	0.319084957

Cnnm1	2.663936088	-0.784069379	0.800016557
Hbegf	2.666049994	-0.372930505	1
Syt16	2.787183848	-2.109760417	0.111617616
Cerox1	2.848941	-0.956554634	0.779178524
Vgf	2.915554576	-3.21895723	0.06115584
Gpr6	2.918543314	0	1
Hapln2	2.919757598	-2.223436101	0.297066875
A830018L16Rik*; uncharacterized protein C8orf34 homolog	2.922209565	-4.390467042	0.00298195
Oprd1	2.92227043	-2.806345331	0.128780313
*Srgap1**	2.923275512	-3.581785979	0.018247108
Iqsec3*	2.923392163	-4.222512026	0.004790631
Trpc3	2.92381771	-0.370982637	1
Ntsr1	2.923912187	-2.221691362	0.301324507
Necab2	2.924373722	0	1
Masp1*	2.925525611	-3.806203079	0.015300864
*Lrfn5**	2.925800396	-4.223156676	0.004738656
Nap1l2	2.925805633	-1.335232282	0.653128532
*Myt1l**	2.925899708	-3.961477493	0.006791694
*Kndc1**	2.925931487	-4.22163886	0.004862742
Rnf208	2.925936398	-1.335232277	0.653594566
*Pcsk2**	2.925970312	-3.807157027	0.015063852
Fam171b	2.926013462	-0.954665605	0.780464836
Nos1	2.926030692	-2.582396815	0.139841173
Cdh12	2.926088981	-1.33523227	0.654150617
Cckbr	2.926108699	-1.33523227	0.654222141
Mab21l1	2.926340976	0	1
Syt10	2.927146583	-1.25866975	0.672229216
Gm48822	2.927209212	0	1
Gm5067*; pseudogene, ribosome biogenesis regulatory protein	2.927268756	-4.016237182	0.01057147
Zic1	2.927786188	-0.373436927	1
Cfap44	2.929461114	-0.948141825	0.664635608
Gm973	2.929873821	-1.215812425	0.4444086
*Cacna2d3**	2.930313345	-3.261594697	0.037976281
Snap91	2.933759951	-3.224749279	0.056389062
Sat2	2.933776193	0	1
Gm15663	2.943974702	-1.694788427	0.125402781
*Prkar1b**	2.945959207	-3.84346167	0.009521833
Cpxm1	2.947517328	-1.591929486	0.334298059
Zdbf2	2.950195591	-0.788478962	0.854370233
Ppp1r26	2.950902072	-2.224797924	0.293795036

Ptgr1	2.962582071	-1.149902725	0.550772458
Spire2	2.968369684	-2.258889698	0.235457739
Tspan7	2.968623442	-0.053365322	1
4933412O06Rik	2.971961494	0	1
Cyp3a59	2.993540589	-0.711124515	0.914334796
Shisal2b	3.00006685	-0.07855666	1
Abcc9	3.057113521	-3.097569366	0.060646229
Agbl2	3.060790798	-2.907388038	0.050290019
Slc4a4	3.076577947	-1.464407474	0.072669882
Synpo2	3.098457034	-0.386204028	1
Acy3	3.116464471	-1.867062192	0.284121418
Adra1a	3.119531449	-2.520322445	0.106830788
Gm9353	3.140493394	0	1
Coro2b	3.154370908	-2.177766779	0.170819152
*Pcdh9**	3.156087226	-3.546422248	0.027761889
Dock3	3.15623935	-1.634927278	0.173515351
Foxc1	3.161369251	-0.117358499	1
1700016P03Rik	3.217343421	-2.567032912	0.160071388
Lyplal1	3.226097526	-0.320134886	1
*Mei1**	3.23661196	-4.201765675	0.007117956
Cfap91	3.237760929	-1.558861072	0.365491312
Tacr3	3.238552898	-1.33523223	0.657594598
Postn	3.24148224	-1.555694683	0.465128084
Eps8l2	3.244241581	-1.42589462	0.275881641
Dagla	3.245022338	-0.045445464	1
*Foxp2**	3.245818293	-2.278203201	0.039238982
Pygo1	3.24587649	-0.373436928	1
Gucy2c	3.246379704	-0.045643834	1
Ptpn5	3.246741735	-1.831189395	0.142124821
Lurap1	3.247699418	-0.8867014	0.680148195
*Lin7a**	3.247758746	-3.809020071	0.014670221
Gria4	3.247791036	-2.225032361	0.293234022
Apba2	3.247859102	-0.371442532	1
Shisa2*	3.248430357	-3.589673774	0.018978437
Hhatl*	3.249553938	-4.024295303	0.009114388
Gm15666	3.250397255	-1.722765919	0.234016896
Npsr1	3.251253935	-2.794144316	0.149320286
Grb14	3.251274404	-0.955713341	0.779401253
6030407O03Rik	3.253423094	0	1
Lmcd1*	3.260255278	-3.800234851	0.017147758
Gm30329	3.265164651	-2.767762865	0.212388355
Spint1	3.281993248	-0.917234608	0.817717827
*Pth2r**	3.293342737	-3.097852777	0.032811562
Kif1a	3.293676175	-1.240182636	0.622585967

Tmem63c	3.347228335	-3.068252622	0.056918295
Smarca5-ps	3.352828655	-0.80669258	0.887211675
Slc4a10	3.353477318	-0.053800932	1
Spaca6	3.358680284	-0.614242753	0.953181754
Pld6	3.359758722	0	1
Gm28050	3.369661653	-0.969949517	0.830705891
Gm48684	3.390603787	-3.550466672	0.054489106
Aoc3	3.447728588	-0.51507925	1
Ankrd36	3.45350536	-1.573259119	0.520061546
Gm11653	3.471650294	0	1
Gm44997	3.485842397	-1.257908003	0.673648227
Col12a1	3.486716795	-0.348569585	1
Zfp449	3.491140313	-0.949483552	0.794415688
C230031I18Rik	3.495143317	-1.104970471	0.633063369
Rsph14	3.495687577	-1.263714935	0.642785923
Elovl4	3.496331702	-0.391142094	1
Pm20d1	3.497186611	-0.636202394	0.955610739
Thbs4	3.501555664	-0.507778907	1
Cyp2j9	3.501577122	-1.845565921	0.387021529
Sfrp1	3.506646385	-2.80870996	0.125257813
Insm1	3.507531315	-2.222521708	0.299290038
Sema6a	3.508451711	-1.785098229	0.08531362
Gm37363	3.512570918	-1.335232227	0.657804848
Gm8173	3.513524959	-0.348706893	1
*St8sia3**	3.513625332	-3.546249302	0.027875337
Asic4	3.515502906	-2.804354185	0.131853163
Spry4	3.516238208	-0.539015416	1
Gm42899	3.516855333	0	1
Fkbp14	3.517762645	-0.046270732	1
Ccdc81	3.519919778	-0.373436889	1
Gm44848	3.520590204	-2.21889947	0.308285731
Flrt3	3.520828146	-0.956121998	0.778985802
Angptl2	3.543305415	-0.465952171	1
9630001P10Rik	3.574834685	-2.212707296	0.324421643
Acat3	3.589631553	-0.105519574	1
Spdya	3.620073896	-0.250466178	1
Pth1r	3.651481106	-1.165508768	0.541204186
*Slc17a7**	3.670030732	-4.39271933	0.002867587
Ttll9	3.681403676	-3.205601819	0.074670663
Gm49069	3.726423796	-2.230920621	0.29670905
Ppfia2*	3.729692553	-3.261141548	0.037826814
Pcdhb22	3.732348883	-0.953277117	0.587269501
Dab1	3.732807823	-0.373436929	1
Stard13	3.760757542	-1.538116346	0.366360941

Ptprd	3.77868366	-0.785620632	0.720020295
Ccdc142	3.783099052	-1.677918638	0.533246257
Fbl-ps2	3.784362761	-1.846686515	0.452210715
Ttc26	3.809863043	-0.565935484	1
Tpbgl	3.874930479	-0.557713874	1
Tmem37	3.889004204	-0.089548376	1
*Rbp4**	3.896661684	-4.199403011	0.007493371
Scn7a	3.904116868	-0.377511579	1
Chrna2	3.908325318	-2.748996536	0.282491984
Gm20661	3.910486989	-0.20239016	1
Gm50341	3.914983778	-0.507203262	1
Rgs7bp	3.922931953	-0.201800656	1
*Etv4**	3.927530593	-4.21355592	0.005620853
Pgr	3.927649977	-0.635069725	0.954940132
Nr2f2	3.936764179	-1.369609905	0.207134236
4930505A04Rik	3.945701708	-1.239760552	0.744799801
Prok2	3.95077804	-2.74033376	0.326562364
Pdpn	3.95357677	-0.944990849	0.711139352
G530011O06Rik	3.967702827	-2.200541008	0.386768959
Kif28	3.970761956	-2.621462258	0.287713802
Alx4	4.012041212	-3.504684567	0.059175667
Grin3b	4.044433307	-2.181642314	0.422889161
Csmd1	4.09186187	-2.53732755	0.086441873
Kcnk2	4.093394437	-3.222968366	0.057796376
Rorb	4.097416943	-1.828550004	0.145675292
Gm16364	4.098940607	-1.392853252	0.650700912
Spink10*	4.154786005	-3.579809647	0.04185983
Rgs7	4.154851766	-2.262843887	0.225369712
Slc13a4*	4.156073569	-3.528511655	0.037134315
Ebf2	4.166490058	0	1
B3gnt9	4.183075741	-0.098113815	1
Mgat3	4.248113614	-0.958310925	0.777476922
Gucy2g	4.249371563	-1.335232169	0.662601652
Kcnq4*	4.250989261	-3.564178883	0.022877073
Tspan11	4.252082871	-1.33523221	0.659221975
Chmp4c	4.264068442	-0.345861472	1
Adh7	4.42333696	-0.641218606	0.959267423
Klhdc7a	4.467094316	-2.314495155	0.192724222
Cilp	4.515822288	-3.144805255	0.258580514
Vamp9	4.517045715	-1.219274649	0.751237133
Pde6a	4.53100857	-0.373436698	1
Fam217a	4.611761457	-1.300528058	0.690804863
Prox1	4.616291162	-1.190554539	0.329973817
Srl	4.670662681	-0.373631834	1

Zmynd15	4.864975383	-0.480952667	1
Adamts13	4.942919463	-2.189165513	0.442860108
Clstn3	4.988196991	-0.359200746	1
*Ncan**	5.083779853	-4.029632935	0.008374539
Prelp	5.092175851	-0.151323937	1
Sult4a1	16.81418274	-0.809375178	0.90948469

Table of Contents Top of Current Table

Supplemental Table 5. List of all differentially expressed genes (P<0.05) in whole blood of wild-type B6129 vehicle (week 0 to 8) that were reversed in expression in 3xTg-AD vehicle (week 0 to 8) animals in comparison. *P<0.05, significant expression in the opposite direction in 3xTg-AD vehicle

DEG (P<0.05): B6129	Log2FoldChange: B6129	Log2FoldChange: 3xTg-AD	P-value: 3xTg-AD
mt-Th	-6.366644712	1.571167701	0.613483879
Pcdh10	-5.922680158	0	1
Myrip	-5.685709372	1.403305643	0.526358477
Col19a1	-5.608153994	1.980394771	0.281352824
mt-Nd4l	-5.518111078	1.101152045	0.759857922
C130073E24Rik	-5.499936994	0.25208807	1
Gm23330	-5.404886663	0.280634084	1
Gm19461	-5.308074964	0	1
Loxhd1	-5.244801483	0.524441017	1
Dazl	-5.208726918	1.403307488	0.53169544
Otogl	-5.176898575	1.403582741	0.55524268
Grin2a	-5.131939683	1.98034901	0.280543591
Dscam	-5.127987264	1.936847185	0.272759708
Dytn	-5.123353609	1.403305965	0.677947289
Stac	-5.093656629	0	1
Gm48871	-5.035849795	0.466869818	1
Gm16372	-4.952393223	0.32135223	1
Sntg1	-4.923299155	1.403307518	0.526562346
Chil5	-4.90062481	1.461157613	0.446934928
Mycbpap	-4.882904577	0.249152386	1
Gm4813	-4.865814784	0	1
Gm2106	-4.865232434	0	1
mt-Ty	-4.859164617	0.530319122	1
Grm5	-4.806689868	1.403307516	0.52690979
A830009L08Rik	-4.804088683	1.743685853	0.344476264
BC034090	-4.794624338	1.40334497	0.530377188
Corin	-4.779371754	0	1
Cnmd	-4.767627288	0	1
Umodl1	-4.719585121	0.82622953	0.884713816
D630023F18Rik	-4.696595823	0	1
Nav3	-4.682328754	1.936029768	0.27242917
Lrp1b	-4.681718345	2.348838212	0.130378658
Clstn2	-4.681152638	0	1
Gabrg3	-4.681151108	1.403307515	0.526997528
Slc6a17	-4.680609327	1.403312029	0.52700927
B230334C09Rik	-4.677585956	0	1
Ppp1r1b	-4.675149252	0	1
Gm13446	-4.663450885	0	1
Dcdc5	-4.640637287	0.262868104	1

Scn10a	-4.61723582	1.403306597	0.635593643
Tpo	-4.604547678	0	1
Ptprt	-4.603775661	1.350968159	0.614332218
Olfr78	-4.597706481	0	1
Csmd2	-4.593828907	2.580442009	0.207188159
Ccl27a	-4.588106381	0.249152448	1
Kcnq2	-4.572222937	2.610173152	0.200866685
Gm31107	-4.550208116	1.403307246	0.568345126
Csrnp3	-4.545932182	0	1
Usp13	-4.544475741	0	1
A930017M01Rik	-4.542307269	0	1
Gm9801	-4.540196949	2.512503522	0.118996265
Kcng1	-4.53772763	0.249152445	1
Col11a1	-4.527649144	1.403369674	0.532914025
Nkd2	-4.521057514	0	1
Gm49192	-4.518936364	1.22545767	0.537346604
Otx1	-4.51867069	0	1
Lama1	-4.513330869	0.401770329	1
Myo16	-4.511558498	1.980527806	0.297576923
Tdrd6	-4.506831909	1.980557431	0.301302612
Gm47950	-4.495505022	0	1
Gm20535	-4.495476055	1.310743302	0.624600426
Gm42597	-4.490946951	0	1
Gm26756	-4.482305341	0.739215455	0.86771583
A330015K06Rik	-4.472785032	2.931806552	0.058339991
Piezo2	-4.438817146	0.528082368	1
Sox18	-4.432344903	0.760145346	0.883451539
Fibcd1	-4.394521182	0	1
Lsamp	-4.394115714	0	1
Syt4	-4.393271443	0	1
Slc17a7*	-4.39271933	3.670030732	0.002579895
Kcnab1	-4.391434163	1.403307514	0.527297717
A830018L16Rik*; uncharacterized protein C8orf34 homolog	-4.390467042	2.922209565	0.045670819
Cyp46a1	-4.389606221	0.835712579	0.844667927
Pde9a	-4.388695767	1.403307504	0.529013268
Cd109	-4.383372819	1.40330749	0.531393221
Synpr	-4.375654099	1.980454608	0.288557303
Gm48743	-4.372400939	0	1
Sostdc1	-4.366289522	0.249152422	1
Avp	-4.338074954	0	1
9330158H04Rik	-4.336178002	2.894965369	0.078604213
Baiap2l2	-4.335383279	0.24915238	1

Cilp2	-4.326378105	1.780846423	0.42317016
Ttc6	-4.319226751	0.249152363	1
Egflam	-4.31491737	1.403306929	0.605421993
Tnni3k	-4.308795855	0	1
3110067C02Rik	-4.276204345	0	1
Mir100hg	-4.255326692	1.403316803	0.527496144
Mgat4c	-4.251451831	0	1
D330050G23Rik	-4.237261231	0	1
Cdh10	-4.22472424	0.267406532	1
Ntm	-4.224365523	1.403307519	0.526344114
Nalcn	-4.224209835	2.512900181	0.117086124
Brinp3	-4.223804902	0.826229983	0.851910548
Igsf11	-4.223375974	0	1
Lrfn5*	-4.223156676	2.925800396	0.044697219
Iqsec3*	-4.222512026	2.923392163	0.04514877
Gm35040	-4.221875088	0	1
Kndc1*	-4.22163886	2.925931487	0.045068252
Matn4	-4.219747906	0	1
Fut9	-4.219728211	0	1
Tfap2b	-4.218597644	0	1
Galntl6	-4.217740736	1.403307504	0.52902686
Krt77	-4.215617651	2.682000375	0.063639805
Fam107a	-4.214991225	2.688746421	0.063772479
Gm26777	-4.214168075	0	1
2900027M19Rik	-4.213757926	0	1
Etv4*	-4.21355592	3.927530593	0.001688593
Kcns3	-4.21031224	0	1
Adamts18	-4.205432519	0	1
Glp1r	-4.20464577	0.260487113	1
Clec18a	-4.202666477	0.249152438	1
Mei1*	-4.201765675	3.23661196	0.022243888
Rbp4*	-4.199403011	3.896661684	0.002539173
Gm45470	-4.184789995	0	1
Gm38187	-4.182617488	0	1
Rpl29-ps2	-4.168495009	0.249152345	1
Rab3c	-4.168365165	0.855337508	0.816183243
Lypd1	-4.159026933	2.339262375	0.144425385
Nfatc4	-4.151132736	1.402982425	0.582055756
Meltf	-4.1483199	0.804183852	0.888337754
4930568G15Rik	-4.119146435	2.981915848	0.074580978
Gm10801	-4.108783656	1.660378899	0.234583613
Cfap65	-4.074317496	1.403855311	0.585067576
Drd1	-4.066686194	0	1
Tmco5	-4.052030107	1.805721855	0.2266782

Gm29340	-4.047440174	1.690480456	0.240078672
Vmn2r53	-4.042854391	0.249152415	1
Nhs	-4.033151597	0	1
Abcc8	-4.031342382	0.249152446	1
Csmd3	-4.030961742	2.513505244	0.1172635
Sox11	-4.03063271	0	1
Slitrk1	-4.03062529	0.826229982	0.852023449
Pcdhb16	-4.030606122	1.980393142	0.281158801
Adra1b	-4.030179796	0	1
Rab15	-4.03017253	2.347621187	0.130994638
Cntnap5b	-4.029779469	0	1
Ncan*	-4.029632935	5.083779853	4.50E-06
Zfp423	-4.02872048	0.531357497	1
Tmem200a	-4.028515268	1.403307511	0.527834944
Nkain3	-4.028308947	1.40330751	0.527930247
Sema3e	-4.027819778	1.403307509	0.528156598
S100b	-4.027448898	1.403307502	0.52932678
Zfp786	-4.026823603	0.249152447	1
2810459M11Rik	-4.026694781	0	1
A330008L17Rik	-4.025898863	1.941313444	0.273344786
Six3	-4.025837893	1.932241066	0.280180667
A2m	-4.025819517	0	1
Zic5	-4.024686209	0	1
Hhatl*	-4.024295303	3.249553938	0.018525151
Gm5067*	-4.016237182	2.927268756	0.049404895
Cda	-4.013147309	0	1
Igdcc3	-4.010830415	0	1
Gm12122	-4.004398767	0.249152426	1
Slc6a5	-4.001107145	0	1
Dlx6os1	-3.99739877	0	1
Gpr179	-3.997372695	1.922451612	0.317350336
Gm37894	-3.997174113	2.92923566	0.058840942
Calcr	-3.995661008	1.98017035	0.3065437
Slc2a10	-3.994831238	0.249152424	1
1110002E22Rik	-3.994745628	1.980605136	0.307370717
Lncenc1	-3.993382452	0	1
Wif1	-3.991812966	0	1
Lif	-3.991253546	0.842532381	0.85136217
Erich6	-3.989935165	0	1
Esyt3	-3.988404436	0.249152416	1
Gm48996	-3.985696281	0.817680205	0.869465025
Gm29508	-3.985142094	0	1
Gm10717	-3.980040807	1.717536083	0.397568669
Gm45496	-3.978673205	1.403691168	0.566926647

Otx2os1	-3.974605919	0	1
Spag16	-3.966133883	0	1
Unc79	-3.96223002	1.78032132	0.296964404
Gm5454	-3.961698414	0	1
Gm9530	-3.961657145	2.252910292	0.156731537
Myt1l*	-3.961477493	2.925899708	0.044529227
Fgfbp1	-3.96101508	0	1
Faim2	-3.959555508	0	1
Dpyd	-3.957084593	1.980326943	0.281447493
Plch2	-3.955065614	0.249152447	1
A330094K24Rik	-3.870008591	2.215084791	0.204917994
Zfp973	-3.866815167	1.978996218	0.354802198
Gm19196	-3.861639674	0.325058715	1
Cdr1os	-3.845835906	1.9803913	0.280939394
Prkar1b*	-3.84346167	2.945959207	0.020152406
Dscaml1	-3.840970641	1.068238054	0.55139647
A730098A19Rik	-3.839731168	0.628099819	0.95910307
Gm43508	-3.827988297	0	1
Slc38a3	-3.812090028	0	1
Cdh8	-3.810199824	0	1
Dok5	-3.810041073	1.980312299	0.282051219
Lin7a*	-3.809020071	3.247758746	0.017464536
Gm37805	-3.809009081	0	1
Plcxd3	-3.808959235	0	1
Pnmal1	-3.808641196	0	1
Ajap1	-3.808479795	1.403307516	0.526901925
Tmem178	-3.80840628	0	1
Nyap2	-3.808211888	1.980388437	0.280598733
Tmem74	-3.808125087	0.858789875	0.765163156
Pcdha12	-3.807616448	0	1
Doc2a	-3.807370136	0	1
Pcsk2*	-3.807157027	2.925970312	0.04518004
Tenm3	-3.806946081	2.512508336	0.118621507
Zkscan2	-3.806928064	0	1
Mro	-3.806577165	0	1
Masp1*	-3.806203079	2.925525611	0.045564795
3632454L22Rik	-3.80562348	0	1
Pcdhb14	-3.805006355	0	1
Kirrel2	-3.804423967	1.403330666	0.528912521
Vmn2r84	-3.804343318	1.403307497	0.530224725
Slc35f4	-3.804228262	0.249152446	1
Pigz	-3.802464494	2.507336351	0.122162456
Cdh15	-3.80121813	1.376106268	0.525520618
Lmcd1*	-3.800234851	3.260255278	0.01874183

Gm15606	-3.800015095	0	1
Gm43789	-3.798765891	0	1
Gm37928	-3.798757996	0	1
Vmn2r85	-3.798196086	0	1
9630014M24Rik	-3.796500198	0	1
Prcd	-3.794987622	0	1
Gm43175	-3.794078855	0	1
Pla2g4e	-3.793564497	0.830924392	0.850459457
Gm44021	-3.792897297	0	1
Gm37818	-3.784766946	2.574068672	0.07245082
Slc9a4	-3.783302053	1.403538833	0.550572315
Kcp	-3.782403555	1.403307413	0.543984339
4921539H07Rik	-3.779693246	1.979837979	0.303620087
Tdgf1	-3.776832505	0	1
Gm42981	-3.776697594	0	1
Gm29674	-3.776505144	0	1
Gm49942	-3.771135866	0	1
Gm10710	-3.765729085	0.249152411	1
Gm4875	-3.765363206	1.403307152	0.580444296
Mettl24	-3.76422916	0.249152408	1
Erich2	-3.758102308	0	1
En1	-3.75759657	0	1
Gm41836	-3.756454221	0	1
4930507D05Rik	-3.75619531	2.52416743	0.165330941
Gm49077	-3.755445185	1.979229966	0.338501504
Il22b	-3.75234326	0	1
C030005K06Rik	-3.678278842	0.826229874	0.862260717
Gm10722	-3.678028826	0.921149325	0.733255364
Gm10718	-3.640824514	1.124468043	0.497826025
mt-Te	-3.638209871	0.803505442	0.794607241
Pax3	-3.590079465	0.249152435	1
Shisa2*	-3.589673774	3.248430357	0.01838181
Tmem144	-3.588395275	0.249152447	1
2310040G24Rik	-3.587926753	0.249152417	1
Srgap1*	-3.581785979	2.923275512	0.045200305
Spink10*	-3.579809647	4.154786005	0.00192496
Grhl3	-3.572976285	2.47672331	0.157521883
Kcnq4*	-3.564178883	4.250989261	0.000502127
Gm10800	-3.562669317	1.183860619	0.449118542
Htr4	-3.552970308	0.834327451	0.845483031
Gm44883	-3.552428386	1.939580401	0.283849546
Cdh18	-3.546552863	1.980382265	0.279865522
Foxb1	-3.546478731	1.365117145	0.535363649
Pcdh9*	-3.546422248	3.156087226	0.015760612

St8sia3*	-3.546249302	3.513625332	0.007121147
Lnx1	-3.546175689	0.249152448	1
Tmem178b	-3.546116965	0.249152449	1
Grik2	-3.546078701	1.403307517	0.526695829
Kcna1	-3.546045256	0	1
Jph1	-3.54596089	0	1
Lrtm2	-3.545833931	2.592727492	0.055361173
Nrg3	-3.545823	0	1
Phyhipl	-3.545822931	1.403307516	0.52685316
Mlip	-3.545806678	0.323626333	1
Slc13a5	-3.545777785	0	1
Dgkb	-3.54565771	0	1
Pcdhac2	-3.54556722	0	1
Sorcs3	-3.545559277	0	1
Cntn6	-3.54531601	0	1
Snhg11	-3.545253143	0.322270681	1
St6gal2	-3.545190846	0	1
Cdh7	-3.545106315	0.321062626	1
Slc7a10	-3.54504743	2.512350275	0.118134292
Msi1	-3.544981193	1.936889354	0.272995505
Shisa6	-3.544828574	0	1
Tafa2	-3.54482755	0	1
Pcdh11x	-3.544725654	1.403307512	0.527533642
Zfp804a	-3.544463816	0.249152448	1
Atp13a5	-3.54437065	0	1
Nkd1	-3.543616743	0	1
A930004D18Rik	-3.543440041	0	1
Gabra5	-3.543230231	0.249152447	1
Frmd3	-3.54299608	0	1
Agt	-3.542587242	0	1
Gm50462	-3.542192589	1.98040903	0.28305684
Xkr7	-3.542081871	2.508590594	0.120914858
Chrna4	-3.541900634	1.403307502	0.529341602
Oprk1	-3.541829739	0.249152446	1
B230206I08Rik	-3.541721949	0	1
Eva1a	-3.541714519	0	1
Pcdhga1	-3.541003275	1.933465053	0.285263315
Gm38260	-3.54041967	0	1
Nsun7	-3.540388067	1.980420634	0.284449905
Gm48542	-3.540341445	0	1
Gm37345	-3.538435366	0	1
Gm37051	-3.538393631	0	1
Gm46123	-3.53831147	0	1
Ecel1	-3.537814013	0	1

Gm45516	-3.537707157	0	1
Gm37069	-3.537640702	0	1
Gm43953	-3.537033511	0	1
1700086D15Rik	-3.536268542	0	1
Gm42443	-3.536167273	0	1
Gm16268	-3.535320957	0.350951609	1
Gm28756	-3.533924515	0	1
B130024G19Rik	-3.533861212	0	1
Fibin	-3.532944586	2.530205591	0.122235108
Pgf	-3.532603035	0.301137583	1
Gm45200	-3.531838576	0.253385701	1
Sfrp5	-3.53134913	0	1
Gm37393	-3.531298431	0.249152423	1
Oxtr	-3.531052527	0	1
Slc26a7	-3.530186215	0.249152437	1
Gm49937	-3.529269769	0	1
Slc13a4*	-3.528511655	4.156073569	0.000636196
Gm45257	-3.528288456	0	1
Abhd12b	-3.523518894	0	1
Pitx2	-3.517937243	0	1
Gm23787	-3.517567259	0	1
Cyp3a13	-3.515893574	0	1
Optc	-3.515255586	0	1
Pax7	-3.512819869	0	1
Kcnj6	-3.432679529	0	1
Fstl4	-3.429731818	0.377809569	1
Pcdhga3	-3.428681308	0	1
Vwc2	-3.426003269	0	1
Col4a1	-3.416636648	0.40050469	1
Zfp469	-3.415674325	1.403307315	0.55881653
Jakmip2	-3.262109123	1.403305837	0.526378224
Pknox2	-3.262055475	0.249152448	1
Ano4	-3.261712521	1.980384872	0.280175052
Cacna2d3*	-3.261594697	2.930313345	0.031536404
Ppfia2*	-3.261141548	3.729692553	0.003200758
Adamts20	-3.258917345	0.257091595	1
Ccn2	-3.25712955	2.172563527	0.067062235
Ccdc13	-3.251853839	0	1
Gm3739	-3.251398987	0.249152443	1
Esrrb	-3.250055483	0	1
Gm44220	-3.240203365	0	1
B4galt2	-3.18490995	2.588369607	0.055497814
mt-Co3	-3.153551386	2.216809427	0.056470908
A4galt	-3.134142991	0.249152422	1

Abi3bp	-3.130809693	0.702491496	0.874640753
Pth2r*	-3.097852777	3.293342737	0.019701807
Dpp10	-3.069097886	1.403307513	0.527463288
Cntn4	-3.067809869	0	1
Ephb3	-3.060621917	2.513904624	0.119475213
H1f10	-2.972852488	0.256645668	1
Prkd1	-2.955490911	0	1
Ccdc148	-2.955190332	1.403307514	0.527311893
Grik1	-2.949403986	0	1
Myo5c	-2.869398956	1.30581411	0.30108093
Eno2	-2.830035009	0.444350639	1
Caln1	-2.828581788	1.934578337	0.273416219
Lhx6	-2.820394726	0.255923703	1
5_8S_rRNA	-2.699515087	0.756101217	0.747885966
Ndrg4	-2.687715117	1.980390582	0.280853962
Gm24265	-2.633506607	0.185452876	1
Zfp697	-2.589350926	1.157067069	0.297656151
Igsf9b	-2.537945472	1.670551171	0.234528656
Il34	-2.5199069	0.341524319	1
Slco5a1	-2.489573989	0.935220454	0.637847284
Fam135b	-2.48745868	2.076064103	0.130360735
Apc2	-2.484254987	0.610850367	0.976633596
mt-Tp	-2.419439502	1.238552811	0.264643644
Nrk	-2.355350872	1.26831571	0.413848717
Mir6236	-2.327355888	1.36296867	0.253425308
Tmod2	-2.318581042	1.392035787	0.200487667
Snord49b	-2.294361774	0.388568159	1
mt-Ta	-2.28269304	1.371891323	0.199393364
Foxp2*	-2.278203201	3.245818293	0.017671724
Bnip5	-2.256418193	0.389894971	1
Snord59a*	-2.223489252	2.208484186	0.042325254
5_8S_rRNA	-2.222655827	0.907992325	0.541205727
Unc5b	-2.125796354	1.403307505	0.528822537
Gm43627	-2.107728321	0.561476796	1
Kcna6	-2.07976756	0.919204983	0.508444847
mt-Tq	-2.06856938	1.470003955	0.128569101
Adgrb3	-2.050364887	0.249152449	1
Nr1i2	-2.020179539	1.001005322	0.3669254
Dixdc1	-2.007794761	1.556549924	0.138351464
Map9	-1.95453812	0.853942016	0.748403217
Zfp618	-1.9358605	0	1
Gm37194	-1.807966369	0.665683416	0.85001949
Elp6	-1.797132033	0.03905941	1
A630072L19Rik	-1.743131214	1.133407952	0.20200476

Nsg1	-1.721791252	0.17810715	1
Gm49420	-1.699689014	0.921605838	0.431305423
Mtrfr	-1.696505991	0.139095813	1
Gm24407	-1.649696552	0.594183122	0.982333665
Sccpdh	-1.493590818	0.083691881	1
Gm26397	-1.285899782	0.527557237	1
2310015A10Rik	1.390907568	-0.232663746	1
Lzts3	1.524082717	-0.33446111	1
Senp8	1.70307933	-0.558468416	1
Gm43313	1.852103038	-1.00189855	0.348158361
2900005J15Rik	2.172214233	-0.056425825	1
Gm42462*	2.294678793	-2.279389068	0.010497215
1300014J16Rik	2.319573334	-0.307955715	1
Gm43857	2.35268134	-0.243728992	1
Gm38235	2.416076036	-0.741729938	0.782830557
1700086O06Rik	2.484105004	-0.335976357	1
Nthl1	2.494583132	-0.337782123	1
Kcnip2	2.503736986	-0.107787942	1
Colec12	2.56505362	-0.133195293	1
P3h4	2.688622357	-0.144899045	1
Gm43145	2.710017242	-1.316338653	0.166994469
Slc7a2	2.712276377	-0.249513354	1
Gm38357	2.763204358	-0.513321279	1
Gm12905	2.767896236	-0.903764108	0.6473129
Gm25821*	2.791979812	-3.185151552	0.00666781
1700029I15Rik	2.79453833	-0.630346877	0.947361123
E230020D15Rik	2.895039865	0	1
Gm44967	3.016063234	-2.052421132	0.052569506
Bco2	3.222394769	-2.228419332	0.115553866
Fbln2	3.273055669	-0.391754793	1
Gm13689	3.284240438	-1.270498548	0.569609166
Irak1bp1	3.285487442	0	1
Frs3*	3.286853452	-2.526939977	0.046931881
1700001L19Rik	3.291183791	-0.816463948	0.852294024
Gm49968	3.297199942	0	1
Gm45073	3.302325319	-2.186558764	0.143446864
Gm13270*	3.311475068	-2.7696026	0.045240648
Snord7	3.33904723	-0.878071522	0.759712389
Mpzl2	3.437916035	-0.710032577	0.918075343
Gm43868	3.463823206	-1.735427672	0.325257645
Pcdhgb4	3.470899129	-0.790842889	0.787369747
Tnfaip6	3.476157632	-1.686366305	0.275758964
Plppr2	3.479781049	-0.253864376	1
Pex11a	3.482769776	-0.084010797	1

Sox2	3.485643218	0	1
Tsku	3.487058771	0	1
Il17rc	3.489372862	0	1
Gm17893*	3.492479278	-3.104431581	0.02036709
Itga11	3.502485884	0	1
Gm43073	3.506965942	-2.119430052	0.19644664
AV099323	3.524894389	-1.53993204	0.274403426
9530022L04Rik*	3.634103346	-3.056985934	0.019075006
Gjc1	3.640251673	-0.313828081	1
Gm48582	3.674181341	-0.40748576	1
Wfikkn1	3.757889582	-1.284340626	0.453269257
ENSMUSG00002075672*	3.82477807	-4.314848177	0.001389429
Gm11745	3.827890041	-0.466992219	1
Gm48673	3.843992817	-0.414979916	1
Gm43365	3.848289597	-0.534948295	1
Gm39121	3.906316567	-0.670306486	0.95546521
Ackr3	3.913213705	-1.13252641	0.56022761
Grem2	3.940146657	-0.319981008	1
Gm20125	3.956326551	-0.665337946	0.93699433
Tex29	3.96424032	-2.570750017	0.130299641
Gm42633	3.978977299	-0.513512563	1
5S_rRNA	4.009459421	-1.833028821	0.17039594
Gm47765	4.023928411	-0.342618274	1
Chtf8	4.056971445	-0.499190629	1
Prss57	4.058342674	-0.349953462	1
Gabra3	4.066848848	0	1
Gm13786	4.101137649	-2.539702777	0.056928975
Nox1	4.132743889	-0.577747228	1
Gm17096*	4.145545202	-4.028120351	0.001726932
Gm37886	4.159076169	-1.295339193	0.52263511
Gm38215	4.171368992	-1.245964685	0.577633133
Mypn	4.201232907	-0.62475287	0.964604219
Gm42780	4.201824051	-1.431778951	0.528431175
Gm42433	4.288387336	-1.229204523	0.583587739
Pcdhgb8	4.291203059	-1.423931152	0.421387685
Tcaf1	4.355127415	-1.844995007	0.353433615
Hyal1	4.385395322	-1.287854524	0.289317804
4833408A19Rik	4.399739409	-1.322015376	0.444769079
Loxl2	4.51380774	0	1
U1	4.634588152	-2.465845296	0.127367442
Snord43	4.667736727	-1.325881892	0.443659931
Gm8850	4.741453304	-0.070096392	1
Gm5242	5.039169017	-1.597688458	0.253134746
Gm47326	5.137859244	-0.264039164	1

Ceacam10	5.199458698	-2.961165482	0.0613703

Supplemental Table 6. List of all significant ($P < 0.05$) differentially expressed genes in whole blood of 3xTgAD CBD (week 8) versus 3xTgAD CBD (week 0) animals; 180 genes downregulated & 663 upregulated

Gene Name	Log2FoldChange	P-value	Regulation
Tdrd5	-16.2632141	7.59E-18	Downregulated
Gm49376	-4.64582962	0.0004011	Downregulated
Nphs1	-4.56314223	0.00830129	Downregulated
Mir1931	-4.4722091	0.0004016	Downregulated
Gm34342	-4.3933903	0.04995725	Downregulated
Gm13350	-4.36623484	0.00556391	Downregulated
A1cf	-4.31044002	0.01910557	Downregulated
Gm37844	-4.26745836	0.00130943	Downregulated
Jazf1	-4.2620144	0.00022681	Downregulated
Gm20276	-4.23659331	0.0235195	Downregulated
Btnl7-ps	-4.19541001	0.00917428	Downregulated
Gm42433	-4.17531992	0.00026053	Downregulated
Gm43728	-4.14889621	0.00667862	Downregulated
Lrrc38	-4.12653989	0.01418324	Downregulated
Gm42975	-4.12386076	0.01745944	Downregulated
Gm42728	-3.99833282	0.00291135	Downregulated
Septin5	-3.97418447	0.01001719	Downregulated
Arl11	-3.96210307	0.00118281	Downregulated
Gm10044	-3.9133281	0.02153937	Downregulated
Gm12543	-3.90508672	0.03277098	Downregulated
Kdm4d	-3.89938354	0.0236746	Downregulated
Bmerb1	-3.84278992	0.0010089	Downregulated
Gm9870	-3.84127371	0.01184885	Downregulated
Gm38223	-3.80416305	0.00609382	Downregulated
Gm12737	-3.80307492	0.0015484	Downregulated
Gm6630	-3.80278764	0.02216146	Downregulated
Robo4	-3.74762698	0.00393826	Downregulated
Rasl11b	-3.74245267	0.00203044	Downregulated
Mir15a	-3.70663791	0.04889024	Downregulated
Gm12971	-3.67904647	0.00582663	Downregulated
Gm14021	-3.66495989	0.0174895	Downregulated
Gm37772	-3.65679652	0.01434756	Downregulated
Gm12846	-3.65048751	0.00989978	Downregulated
9330162012Rik	-3.61915234	0.00291398	Downregulated
Gm11440	-3.58040109	0.00731989	Downregulated
Gm45148	-3.56736337	0.00468956	Downregulated
Ldhal6b	-3.54507709	0.01235394	Downregulated
Prokr2	-3.53380971	0.01019159	Downregulated
Itpa-ps1	-3.53197688	0.00521969	Downregulated
Gm47075	-3.53021749	0.00613332	Downregulated
Gm43413	-3.50515773	0.00868497	Downregulated

D630003M21Rik	-3.50425273	0.02776329	Downregulated
Car12	-3.49040541	0.00903449	Downregulated
Gm37529	-3.47800266	0.00083733	Downregulated
ENSMUSG00002074935	-3.47116179	0.02067095	Downregulated
Gm26737	-3.44831288	0.04455496	Downregulated
Gm43909	-3.4317702	0.01309172	Downregulated
Gm49086	-3.4152609	0.01161787	Downregulated
Galnt15	-3.40144607	0.01715535	Downregulated
Gm44265	-3.37735886	0.03190558	Downregulated
Pip5kl1	-3.37701389	0.00794481	Downregulated
Gm47594	-3.37590099	0.03912041	Downregulated
Gm19046	-3.3506407	0.03672387	Downregulated
Gm42866	-3.33377062	0.00209439	Downregulated
Gm49616	-3.33289139	0.02598517	Downregulated
Mycl	-3.3279459	0.00372919	Downregulated
A930035D04Rik	-3.31043704	0.03084739	Downregulated
Spata6l	-3.28825973	0.01436986	Downregulated
Tmem47	-3.28479933	0.03338259	Downregulated
Gm23887	-3.27547471	0.02646768	Downregulated
Lrp11	-3.24920769	0.00927608	Downregulated
5830487J09Rik	-3.24671281	0.00759416	Downregulated
ENSMUSG00002075075	-3.24236293	0.01378081	Downregulated
Sox17	-3.24075821	0.02478381	Downregulated
2810407A14Rik	-3.22969762	0.03595118	Downregulated
Gm44830	-3.21077139	0.02339643	Downregulated
Nostrin	-3.19993968	0.02259532	Downregulated
Gm48254	-3.19893244	0.04101152	Downregulated
Gm43437	-3.19472434	0.00679608	Downregulated
Gm45807	-3.19147882	0.01395945	Downregulated
Cracr2b	-3.1858264	0.00544504	Downregulated
Map2k3os	-3.16312321	0.01532442	Downregulated
Tigd3	-3.15255185	0.01868079	Downregulated
9930014A18Rik	-3.11188917	0.01704683	Downregulated
Gm20732	-3.10310493	0.01758218	Downregulated
Susd4	-3.10050137	0.01502277	Downregulated
Gm43690	-3.08952093	0.02837805	Downregulated
Gm37536	-3.07904413	0.03853896	Downregulated
Gm44695	-3.07426028	0.016876	Downregulated
C030032O16Rik	-3.07229061	0.04912039	Downregulated
Ldha-ps2	-3.07098738	0.02897197	Downregulated
5S_rRNA	-3.06255389	0.04183002	Downregulated
Ushbp1	-3.05758952	0.0212031	Downregulated
Rgs7bp	-3.05626557	0.01327191	Downregulated
Gm44899	-3.04848095	0.00702745	Downregulated
Gm48673	-3.0302333	0.01551252	Downregulated
Gm17473	-3.00706292	0.03103357	Downregulated

Trav14d-3-dv8	-2.97186727	0.0343982	Downregulated
Gm14443	-2.96577547	0.01897711	Downregulated
Gm32679	-2.95671311	0.04457391	Downregulated
Smad9	-2.95377573	0.02386153	Downregulated
Pcdhga1	-2.95121103	0.02907978	Downregulated
Gm17509	-2.93979351	0.0325369	Downregulated
Creg2	-2.93775147	0.02532238	Downregulated
Tmod2	-2.93688248	0.01923966	Downregulated
Tmem215	-2.92825292	0.02737055	Downregulated
Kcnj12	-2.92302153	0.02648513	Downregulated
Gm43072	-2.92106317	0.03247635	Downregulated
Gm13868	-2.91319313	0.03612655	Downregulated
Aldob	-2.90376867	0.03671439	Downregulated
Spx	-2.88758449	0.04241002	Downregulated
Tas1r3	-2.86011782	0.00696823	Downregulated
Gm38241	-2.85865285	0.03755285	Downregulated
Gm38010	-2.85009928	0.03754985	Downregulated
Gm45203	-2.84883836	0.04358431	Downregulated
Gm45823	-2.84535782	0.038697	Downregulated
Grm1	-2.81598535	0.02771679	Downregulated
Paqr5	-2.78384266	0.03744373	Downregulated
Pdlim4	-2.77674758	0.00343666	Downregulated
Rab15	-2.74646982	0.04266463	Downregulated
Fjx1	-2.74581962	0.04301795	Downregulated
Galnt16	-2.73945511	0.04323737	Downregulated
Gria2	-2.73537198	0.0438443	Downregulated
Smim17	-2.73248789	0.04495099	Downregulated
Syde1	-2.71755267	0.0257198	Downregulated
Gm43412	-2.70642819	0.03825518	Downregulated
Gm12216	-2.69643244	0.0161776	Downregulated
Gm44045	-2.67345527	0.01642427	Downregulated
Fuom	-2.64138705	0.01676243	Downregulated
Pwwp2b	-2.63928876	0.00677437	Downregulated
8430422M14Rik	-2.61406415	0.02403043	Downregulated
Hic2	-2.59038215	0.00434354	Downregulated
Slc12a5	-2.56223092	0.02154684	Downregulated
Gm37663	-2.55638273	0.00471774	Downregulated
Ankrd24	-2.51949128	0.00125106	Downregulated
Reep1	-2.5018989	0.045725	Downregulated
Slc16a9	-2.48837585	0.00752783	Downregulated
C130013H08Rik	-2.46094754	0.02102498	Downregulated
Gm47324	-2.4590615	0.03145114	Downregulated
2010310C07Rik	-2.44292756	0.01068956	Downregulated
B930036N10Rik	-2.41984722	0.03522517	Downregulated
Edn3	-2.37706192	0.02891381	Downregulated
Gm49201	-2.376692	0.04552445	Downregulated

Gm42878	-2.36106785	0.03262766	Downregulated
Zfyve28	-2.35727561	0.00777848	Downregulated
Gm43006	-2.34773292	0.03066092	Downregulated
Gm17203	-2.3461798	0.01770293	Downregulated
Gm47121	-2.32030904	0.0262259	Downregulated
6230400D17Rik	-2.31840201	0.01108726	Downregulated
1700030J22Rik	-2.31659991	0.04877778	Downregulated
Gm45293	-2.30505625	0.04273599	Downregulated
Tuba-rs1	-2.30443819	0.03953177	Downregulated
Gm27019	-2.28085758	0.03187638	Downregulated
Ston1	-2.2201485	0.0147555	Downregulated
C230037L18Rik	-2.200248	0.00809117	Downregulated
Gm37570	-2.19628007	0.0416401	Downregulated
Cited4	-2.14923668	0.0081131	Downregulated
Gm48961	-2.14638624	0.04777067	Downregulated
A730081D07Rik	-2.13100525	0.04622445	Downregulated
Gm44888	-2.08370178	0.02732997	Downregulated
Gm43254	-2.08047407	0.02329156	Downregulated
Myadml2	-2.07471053	0.04205205	Downregulated
Gm12346	-2.06404344	0.02065782	Downregulated
Trim62	-2.04369907	0.01684949	Downregulated
Adamts14	-1.98468137	0.00893232	Downregulated
Aldh1b1	-1.98019189	0.04825964	Downregulated
Efna5	-1.97994858	0.00442384	Downregulated
Gm13357	-1.97989828	0.03855317	Downregulated
Gm10933	-1.94408199	0.03232134	Downregulated
Snord118	-1.89075075	0.02276076	Downregulated
Rpusd3	-1.81202182	0.04572235	Downregulated
Trp53i13	-1.79778316	0.01989459	Downregulated
Gm40309	-1.77184925	0.04503563	Downregulated
Ndnf	-1.72602702	0.00495642	Downregulated
Gm43329	-1.72382277	0.03929927	Downregulated
Hdhd2	-1.716683	0.00582405	Downregulated
Gm44664	-1.66711179	0.02219075	Downregulated
Amot	-1.65373781	0.00936861	Downregulated
Tnfsf13b	-1.58784371	0.03856306	Downregulated
Ptpdc1	-1.54599945	0.02623878	Downregulated
Bean1	-1.49321548	0.01976832	Downregulated
Zfp653	-1.49157375	0.02505352	Downregulated
BC048403	-1.43402286	0.01511339	Downregulated
Armcx1	-1.29786576	0.02994604	Downregulated
Ssbp4	-1.26836206	0.00158459	Downregulated
4833413G10Rik	-1.19564591	0.04506935	Downregulated
Zfp984	-1.17048193	0.04188329	Downregulated
Gata2	-1.14404181	0.03956275	Downregulated
Plxna4	-1.13168074	0.02743217	Downregulated

Phactr2	-1.00190365	0.01551599	Downregulated
Med6	1.00600647	0.04841807	Upregulated
Dph3	1.0092383	0.03142077	Upregulated
mt-Nd5	1.04420919	0.03819092	Upregulated
Commd6	1.04667223	0.04921798	Upregulated
Med28	1.06229588	0.03163909	Upregulated
Mtch2	1.06453666	0.02341875	Upregulated
Ap4s1	1.07492168	0.02064135	Upregulated
Commd2	1.07696991	0.04998693	Upregulated
Hikeshi	1.09483395	0.00872962	Upregulated
Hint3	1.10945271	0.03896365	Upregulated
Selenok	1.10945982	0.0218282	Upregulated
Cdc34	1.11049101	0.02285791	Upregulated
Atp6v0b	1.12476022	0.01858899	Upregulated
Rps15	1.13811223	0.03064237	Upregulated
Pdcd10	1.14345064	0.03698807	Upregulated
Rnf7	1.16230087	0.02728224	Upregulated
Cript	1.16244808	0.00653251	Upregulated
mt-Nd4	1.16885181	0.00473812	Upregulated
Rpl22l1	1.16953793	0.04703854	Upregulated
Ift20	1.17363857	0.03700454	Upregulated
Mrpl46	1.17404529	0.01556311	Upregulated
Mrpl30	1.17827676	0.04102503	Upregulated
Naa38	1.18096294	0.03165831	Upregulated
Bola2	1.18303422	0.04307192	Upregulated
Coq10a	1.18383492	0.01730876	Upregulated
Mien1	1.18579962	0.02644843	Upregulated
Use1	1.19242626	0.03667732	Upregulated
Rpl32	1.20292618	0.03511986	Upregulated
Snrpe	1.21028137	0.01637551	Upregulated
Coa3	1.21369037	0.03026798	Upregulated
Glrx3	1.21836282	0.01371171	Upregulated
Lsm7	1.21847918	0.03467359	Upregulated
Tmem208	1.226341	0.03543097	Upregulated
Mrpl41	1.23343153	0.03264834	Upregulated
Ufc1	1.23520243	0.01586921	Upregulated
Mrps14	1.23614321	0.03516818	Upregulated
Rpl13a	1.24766054	0.02760806	Upregulated
Sec11c	1.25898372	0.0107523	Upregulated
Dbi	1.26015736	0.04691221	Upregulated
Ccdc167	1.26081281	0.01698813	Upregulated
Trmt61b	1.26118766	0.01870965	Upregulated
Lancl1	1.26664961	0.04572441	Upregulated
Pycard	1.27133306	0.04750094	Upregulated
Spcs1	1.28083229	0.04742678	Upregulated
Rpl9	1.28137643	0.01902589	Upregulated

Chrac1	1.28361854	0.02442762	Upregulated
Rps13	1.28577904	0.0170956	Upregulated
Rps9	1.29120272	0.00396028	Upregulated
Gm23442	1.29353274	0.03664415	Upregulated
Sf3b6	1.30843294	0.00439809	Upregulated
Ndufa5	1.30848941	0.03900046	Upregulated
Ndufb1	1.31444262	0.02512336	Upregulated
Rps21	1.31686128	0.01925452	Upregulated
Psmb4	1.31693936	0.00784157	Upregulated
Rpl5	1.32126917	0.00503466	Upregulated
Rpl35a	1.32200089	0.01203497	Upregulated
Srp14	1.32538019	0.01827064	Upregulated
Rack1	1.34627359	0.01519435	Upregulated
Ssr4	1.34651882	0.01143669	Upregulated
Med11	1.34926798	0.01002212	Upregulated
Rps19	1.34988727	0.01983832	Upregulated
Nudt14	1.35069169	0.0486485	Upregulated
Vps29	1.35089667	0.00492101	Upregulated
Cox7c	1.35816573	0.03250078	Upregulated
mt-Cytb	1.35951306	0.00071488	Upregulated
Mrps24	1.36427952	0.006987	Upregulated
Nit2	1.36488153	0.01870689	Upregulated
Rpl17-ps10	1.3693708	0.03085811	Upregulated
Rpl10	1.37188302	0.02084485	Upregulated
mt-Nd1	1.37231583	0.00077822	Upregulated
Zfas1	1.3728527	0.01144115	Upregulated
Atp6v1f	1.37629425	0.00317671	Upregulated
Cenpx	1.37774078	0.02342842	Upregulated
Mrpl20	1.38319686	0.03997697	Upregulated
Polr2k	1.3858305	0.02483744	Upregulated
Bud31	1.38635958	0.02397773	Upregulated
Txn1	1.38920082	0.03519196	Upregulated
Mea1	1.39913998	0.02117698	Upregulated
Ubl4a	1.40065523	0.03128445	Upregulated
Mettl5	1.4054953	0.04837345	Upregulated
Gm7536	1.40662378	0.02798189	Upregulated
Cox4i1	1.40833727	0.02147498	Upregulated
Hsd17b10	1.40854748	0.00915375	Upregulated
H4c14	1.42280877	0.02954332	Upregulated
Rpsa-ps10	1.42684321	0.04113784	Upregulated
Ccdc141	1.4308671	0.03472323	Upregulated
Kbtbd3	1.43276225	0.0091292	Upregulated
Fmc1	1.43817182	0.0358076	Upregulated
Cyba	1.43951379	0.00533441	Upregulated
Cox16	1.44502316	0.03374031	Upregulated
Itgb1bp1	1.44596596	0.00063765	Upregulated

Fundc2	1.4524421	0.00338972	Upregulated
Tesc	1.45464357	0.03548601	Upregulated
Micos13	1.45534977	0.00900297	Upregulated
Psma5	1.45773224	0.03791278	Upregulated
Rpl17	1.45837112	0.0114798	Upregulated
Ube2b	1.45993585	0.01608433	Upregulated
Ndufa3	1.4641392	0.01951258	Upregulated
Rpl9-ps6	1.46986803	0.01299072	Upregulated
Rps3	1.474603	0.00685948	Upregulated
Sar1b	1.48197964	0.00286651	Upregulated
Fam162a	1.48278722	0.01796422	Upregulated
Rps18	1.48302002	0.00263504	Upregulated
Snhg1	1.48542256	0.00909565	Upregulated
Gm10076	1.48775954	0.04333893	Upregulated
Hadh	1.4883137	0.0128479	Upregulated
Supt4a	1.49321686	0.03330594	Upregulated
Gm25117	1.50165592	0.00557985	Upregulated
Rps16	1.50313682	0.00159826	Upregulated
Sptssa	1.50531422	0.00102928	Upregulated
Tm4sf1	1.51055117	0.0478712	Upregulated
Atp5h	1.51170332	0.01030759	Upregulated
Rps25	1.51244395	0.00076568	Upregulated
Tbca	1.52190903	0.00794765	Upregulated
Ppia	1.52276793	0.00027038	Upregulated
Gm48226	1.52416281	0.03449665	Upregulated
A430005L14Rik	1.52466212	0.00534247	Upregulated
Ndufb2	1.53437797	0.01758233	Upregulated
Lsm4	1.53966443	0.01046406	Upregulated
mt-Nd2	1.5435232	0.00044104	Upregulated
Echs1	1.54600205	0.00227779	Upregulated
Mrps36	1.54889157	0.00826293	Upregulated
Bloc1s1	1.5512985	0.0485968	Upregulated
Pfdn1	1.5542542	0.0236333	Upregulated
Rps27rt	1.55996026	0.01397347	Upregulated
Acot13	1.56056834	0.00205326	Upregulated
Ndufa4	1.56180035	0.00389488	Upregulated
Mrpl21	1.56206881	0.01812636	Upregulated
Pafah1b3	1.56336305	0.00494117	Upregulated
Rps12	1.56601499	0.00304973	Upregulated
Elof1	1.5687184	0.0003728	Upregulated
Swi5	1.59243383	0.00048027	Upregulated
Gm6421	1.59385688	0.01913684	Upregulated
Rpl27	1.59693612	0.00080878	Upregulated
Hspb11	1.59998014	0.02037581	Upregulated
Nsmce1	1.60278885	0.00158541	Upregulated
Gm10269	1.60283895	0.04177581	Upregulated

Rpl41	1.6030956	0.018555	Upregulated
Lncppara	1.61014841	0.0122108	Upregulated
Rpl27a	1.61191761	0.00030042	Upregulated
AI413582	1.61403065	0.01326527	Upregulated
Atp5o	1.61664188	0.0143198	Upregulated
Zswim7	1.61866071	0.02117415	Upregulated
Uqcrq	1.62084345	0.02026871	Upregulated
Dctn3	1.62502379	0.00123313	Upregulated
Flnb	1.62691565	0.02472439	Upregulated
Gm19585	1.64019385	0.04064226	Upregulated
Lockd	1.64043688	0.02502776	Upregulated
Paox	1.64518047	0.02520755	Upregulated
Bbln	1.64635946	0.01905297	Upregulated
Snrpd2	1.65183426	0.00266731	Upregulated
Ramp1	1.65446508	0.00292223	Upregulated
Mrpl14	1.65597793	0.01357127	Upregulated
Fau	1.65710356	1.15E-05	Upregulated
Gm10736	1.65923019	0.01895095	Upregulated
Rps15a-ps6	1.66811781	0.02261481	Upregulated
Tmsb4x	1.67069967	0.00039629	Upregulated
Selenof	1.67158228	0.00303142	Upregulated
Hint1	1.67160135	0.00444712	Upregulated
Pih1d2	1.67201675	0.04250409	Upregulated
Gm9843	1.67228257	0.00125038	Upregulated
Ndufs6	1.672467	0.00210677	Upregulated
Uqcr11	1.67307307	0.00108749	Upregulated
Phf19	1.67432159	0.02071807	Upregulated
Ciao2a	1.67642833	0.00061657	Upregulated
Rps16-ps2	1.677486	0.00752096	Upregulated
Gm10123	1.68102613	8.46E-05	Upregulated
Ndufa7	1.6813642	0.01495745	Upregulated
Pfdn5	1.68331656	0.00019539	Upregulated
Mrpl13	1.68614954	0.00035405	Upregulated
Med31	1.68862072	0.00678724	Upregulated
Romo1	1.68904219	0.0017695	Upregulated
Tomm5	1.70407223	0.00083169	Upregulated
Cmtm3	1.70441003	0.01768818	Upregulated
Gm4600	1.706122	0.0350648	Upregulated
1810037I17Rik	1.70652145	0.00957256	Upregulated
Zfp85	1.70750279	0.00508074	Upregulated
Atp5e	1.71028682	0.00792922	Upregulated
Cox7b	1.71566385	0.0112706	Upregulated
Nop10	1.71578619	0.00367633	Upregulated
Dctpp1	1.71763155	0.04860289	Upregulated
Atp5k	1.72506879	0.00040431	Upregulated
Cox6b1	1.72804598	0.00269747	Upregulated

Slirp	1.72902898	0.00144299	Upregulated
Tma7	1.73190952	0.00041911	Upregulated
Timm8b	1.73301644	0.00191682	Upregulated
Mtln	1.74080566	0.00553097	Upregulated
Gpx4	1.74261719	0.01026049	Upregulated
3110001I22Rik	1.74443997	0.01520681	Upregulated
Atp5j2	1.75905908	0.00147835	Upregulated
Prdx2	1.76323136	0.01197537	Upregulated
Mrpl28	1.76333103	0.00115634	Upregulated
Snrnp25	1.77485619	0.00175467	Upregulated
Ndufb4	1.77638183	0.00103919	Upregulated
Rps12-ps3	1.78142382	0.00168295	Upregulated
BC028528	1.78375359	0.00494604	Upregulated
Map1lc3b	1.78514603	1.36E-06	Upregulated
Rpl36	1.78991238	1.32E-05	Upregulated
Sv2c	1.79378523	0.02746114	Upregulated
Rps12-ps9	1.80189625	0.00190727	Upregulated
Gm6055	1.8098285	0.00286346	Upregulated
Snx21	1.81030008	0.04154754	Upregulated
Fzd1	1.81582892	0.03596965	Upregulated
Sorbs3	1.82235732	0.02995861	Upregulated
Dpy30	1.82501813	2.37E-05	Upregulated
Gm10250	1.82945762	0.0219836	Upregulated
Prdx1	1.84523572	0.00088747	Upregulated
Wfdc3	1.84616516	0.03810724	Upregulated
Pcbp4	1.84922411	0.00606828	Upregulated
Ndufa11	1.85507201	0.00424519	Upregulated
Ndufa2	1.86056914	0.00015327	Upregulated
Tspo	1.86530993	0.0160127	Upregulated
Fabp4	1.86777338	0.02359477	Upregulated
Ano2	1.87116233	0.02654795	Upregulated
Isca2	1.87485053	2.12E-06	Upregulated
Tyms	1.87509824	0.00827436	Upregulated
Rpp38	1.87551592	0.02944953	Upregulated
Plac8	1.88982452	0.03088472	Upregulated
Rps12-ps4	1.89323136	0.00057377	Upregulated
Gm10180	1.89464496	0.02370662	Upregulated
Gstm5	1.89785665	0.00122886	Upregulated
Aspa	1.9062915	0.03401708	Upregulated
Fbxl15	1.91433806	0.02050553	Upregulated
1500026H17Rik	1.9172024	0.01814877	Upregulated
mt-Nd3	1.92210432	0.0127345	Upregulated
H3f3a-ps2	1.92282344	0.01973891	Upregulated
Atp8b5	1.92380295	0.04263294	Upregulated
Rps27l	1.92594699	5.81E-05	Upregulated
Tmem147	1.92711514	3.06E-05	Upregulated

Rps24	1.93046699	0.00012008	Upregulated
Mansc1	1.93713164	0.04376102	Upregulated
Smim4	1.93768928	0.00366934	Upregulated
Polr2j	1.94875821	0.00082951	Upregulated
Gm15500	1.96974638	0.00136009	Upregulated
Gm28437	1.97185604	0.02311248	Upregulated
Tceal9	1.98469052	1.74E-05	Upregulated
Timm10	1.98508189	1.06E-05	Upregulated
Rpl10a	1.98802472	1.32E-05	Upregulated
Gm10221	1.99105424	0.0120981	Upregulated
Rps24-ps3	1.99262613	0.01352635	Upregulated
Uqcrh	1.99950299	0.00131181	Upregulated
Uxt	2.00145303	0.00075696	Upregulated
Gm8186	2.04167649	0.01030351	Upregulated
4933431K14Rik	2.04235341	0.01262184	Upregulated
Atp5mpl	2.04322029	5.99E-05	Upregulated
Rpl35	2.05096089	0.00011273	Upregulated
Htr1b	2.05148781	0.03683642	Upregulated
Tmtc1	2.05235275	0.01263765	Upregulated
Axl	2.05791615	0.01368865	Upregulated
Gm49024	2.07030396	0.04424842	Upregulated
Rab3a	2.07687884	0.00307171	Upregulated
Rps10-ps2	2.08028479	0.00110827	Upregulated
Snora78	2.08223084	0.01690537	Upregulated
Gulp1	2.08263826	0.04113095	Upregulated
Rpl39-ps	2.08403176	0.04220699	Upregulated
Cox5b	2.09077196	6.38E-06	Upregulated
Mrpl57	2.11169795	2.00E-05	Upregulated
Pls3	2.12540058	0.00319421	Upregulated
Gatm	2.12654768	0.00729616	Upregulated
Cox7a2	2.12812138	0.00047005	Upregulated
Rexo2	2.133784	2.81E-05	Upregulated
Rps19-ps6	2.13949275	0.00129661	Upregulated
Gm23346	2.14782969	0.02111045	Upregulated
Hcfc1r1	2.15320562	0.00026113	Upregulated
Fabp5	2.16373164	0.00525012	Upregulated
Cops9	2.16596555	3.60E-05	Upregulated
Cdo1	2.16874937	0.00385409	Upregulated
Gm12319	2.1732303	0.02219935	Upregulated
Rpl17-ps8	2.17950593	0.00117196	Upregulated
Ggh	2.18720068	0.00032827	Upregulated
Hlf	2.2025092	0.02254544	Upregulated
Gm5905	2.20391634	0.00110771	Upregulated
Gm13611	2.21048142	0.03805164	Upregulated
Acot6	2.21236863	0.04111011	Upregulated
Cacna2d1	2.21258309	0.04988203	Upregulated

Cks1b	2.22141509	0.00218184	Upregulated
Ackr3	2.22354397	0.01501349	Upregulated
Lage3	2.22411852	1.65E-05	Upregulated
Ndufb6	2.24352526	0.00110255	Upregulated
Dennd2a	2.24888875	0.02213334	Upregulated
Gpx4-ps2	2.24987619	0.00069275	Upregulated
Ttc8	2.25755515	0.03597704	Upregulated
Dctd	2.27305017	0.01542412	Upregulated
Lyrm1	2.29745133	0.00716305	Upregulated
Gm2000	2.30902948	0.00022117	Upregulated
Mgst3	2.31221555	0.00755706	Upregulated
Ak1	2.31299698	0.04472716	Upregulated
Map6	2.31421434	0.04683835	Upregulated
1700094M23Rik	2.32840666	0.02480378	Upregulated
Rps10-ps1	2.34044503	0.01464674	Upregulated
Uqcc2	2.34765271	6.01E-06	Upregulated
Uchl1	2.35750689	0.04374981	Upregulated
Tfpi	2.3702366	0.01755464	Upregulated
Bcl2a1d	2.37831137	0.02451372	Upregulated
Cxcl12	2.38148474	0.01945534	Upregulated
Cib2	2.39439559	0.01952658	Upregulated
Car5b	2.39967096	0.00057139	Upregulated
Mapk8ip1	2.42878625	0.01621269	Upregulated
Gm9531	2.4517493	0.02106528	Upregulated
7SK	2.45223095	0.00600758	Upregulated
Btf3-ps1	2.46044917	0.04337761	Upregulated
Snrpert	2.46080252	0.02485097	Upregulated
Vegfb	2.46344484	0.00088789	Upregulated
Lamb2	2.46771324	0.01499552	Upregulated
Map1b	2.483108	0.01370719	Upregulated
Akr1e1	2.49232916	0.00478853	Upregulated
Evpl	2.50912027	0.04472401	Upregulated
Ddr2	2.52296375	0.01920124	Upregulated
Parm1	2.52635264	0.01525152	Upregulated
Avpi1	2.52701709	0.0083117	Upregulated
A930009A15Rik	2.53340231	0.01543676	Upregulated
Lrrc49	2.53683577	0.00495627	Upregulated
Cstb	2.53861714	9.53E-06	Upregulated
BC034090	2.54800837	0.04595251	Upregulated
Rangrf	2.56395589	0.04569084	Upregulated
Mpdz	2.61586254	0.04968144	Upregulated
Rbms3	2.61722565	0.04960298	Upregulated
Kcnk10	2.6283484	0.04896437	Upregulated
Mrc1	2.62842923	0.00124905	Upregulated
Spats2	2.62844562	0.04846753	Upregulated
Uqcr10	2.63015568	5.54E-07	Upregulated

Dagla	2.63119155	0.01688517	Upregulated
Nme4	2.6343116	0.0081566	Upregulated
Gm42456	2.63641569	0.00839466	Upregulated
Ptprd	2.65049284	0.01522102	Upregulated
Atp13a5	2.65216767	0.04654258	Upregulated
Mok	2.65637614	0.01111716	Upregulated
Sntg2	2.65949982	0.04811842	Upregulated
Gm25432	2.67858931	0.04559567	Upregulated
Prelp	2.67970836	0.01643926	Upregulated
Aox1	2.68012986	0.03741679	Upregulated
Lonrf3	2.68852915	0.02881438	Upregulated
Ryr1	2.69972699	9.47E-05	Upregulated
Ndufa12	2.70623518	1.32E-06	Upregulated
Tnfsf13	2.70913011	0.02205716	Upregulated
Gm19391	2.71266502	0.04540859	Upregulated
Casp12	2.71950961	0.04272013	Upregulated
Lypd1	2.72426252	0.04652323	Upregulated
Copz2	2.74033147	0.03938389	Upregulated
Lurap1	2.7516451	0.03558986	Upregulated
Gm12254	2.76041269	0.00625319	Upregulated
Gm12669	2.76626273	0.04591074	Upregulated
Nol3	2.78085326	0.02209317	Upregulated
Cd34	2.78816483	0.01185506	Upregulated
Tbx3	2.80285757	0.04710596	Upregulated
Cdkn1c	2.8090971	0.03355738	Upregulated
Pnma3	2.81229921	0.03831899	Upregulated
Shisa2	2.81357303	0.03885229	Upregulated
Rxrg	2.81777145	0.03837785	Upregulated
Nhs	2.82627811	0.03582343	Upregulated
Cdh20	2.82854351	0.03552154	Upregulated
Podxl2	2.83142103	0.03562418	Upregulated
1700048O20Rik	2.83240221	0.04478834	Upregulated
Gm49601	2.83341223	0.0383577	Upregulated
Jam3	2.83664295	0.03462696	Upregulated
Sox21	2.8384603	0.03521486	Upregulated
Adcyap1r1	2.83875357	0.03430583	Upregulated
Pcolce2	2.83887626	0.0363443	Upregulated
Clip4	2.84113344	0.03417864	Upregulated
B3galt1	2.84160397	0.03403402	Upregulated
Cgref1	2.84597078	0.03539872	Upregulated
Fgd1	2.84660453	0.03482421	Upregulated
Rtkn	2.85106345	0.03444829	Upregulated
Gm17276	2.85418202	0.03482906	Upregulated
Tmc3	2.85441571	0.01227416	Upregulated
Npr3	2.86436033	0.0174554	Upregulated
Ntn5	2.87501708	0.03820343	Upregulated

Tnnt3	2.88164391	0.03106997	Upregulated
Proca1	2.88214499	0.0360858	Upregulated
Togaram2	2.88248179	0.0417386	Upregulated
Cfap45	2.88419067	0.03005584	Upregulated
4933412O06Rik	2.88637097	0.03915418	Upregulated
Tagln3	2.88672748	0.01537248	Upregulated
Bend6	2.9020098	0.01463983	Upregulated
Abca8a	2.90231443	0.02347753	Upregulated
Slc1a3	2.91054581	0.02191412	Upregulated
Mgl2	2.91426942	0.03294661	Upregulated
Fgf2	2.91816801	0.01692262	Upregulated
Kctd15	2.94326396	0.03124855	Upregulated
Wnt2b	2.94724705	0.04192225	Upregulated
1700007L15Rik	2.94997483	0.0052445	Upregulated
Col14a1	2.95055786	0.03664567	Upregulated
Ccbe1	2.9602869	0.04962948	Upregulated
Slc4a3	2.96120874	0.03928898	Upregulated
Pla2g5	2.96528045	0.04096413	Upregulated
Hbb-bh1	2.96889127	0.03178031	Upregulated
Junos	2.97900263	0.00211624	Upregulated
Gm32051	2.98009861	0.00315282	Upregulated
Mfsd4b1	2.99555632	0.03673741	Upregulated
Gm32282	2.99830849	0.03182516	Upregulated
Calr3	3.00596664	0.03377556	Upregulated
Gm15927	3.01161472	0.01000521	Upregulated
Kiss1r	3.01268222	0.01654045	Upregulated
Fblim1	3.01768005	0.03504093	Upregulated
Lgr5	3.01827668	0.04698853	Upregulated
Pdgfra	3.02114228	0.0205145	Upregulated
Slc2a13	3.02436762	0.02001765	Upregulated
Mageh1	3.02499059	0.0159705	Upregulated
Pth1r	3.02549133	0.02270807	Upregulated
Syn2	3.02843861	0.0197177	Upregulated
Marchf10	3.02886525	0.04286081	Upregulated
Ntm	3.0289341	0.01956273	Upregulated
Kif21a	3.03113705	0.01937764	Upregulated
Gm45605	3.03279484	0.02692184	Upregulated
St18	3.03473479	0.02012298	Upregulated
Bcas1	3.03590907	0.02069136	Upregulated
Lyz1	3.03956515	0.00694582	Upregulated
Pcdh18	3.04032028	0.02026921	Upregulated
Dclk1	3.04125331	0.01457886	Upregulated
Prox1	3.04125547	0.01508878	Upregulated
Snx7	3.04139491	0.01869682	Upregulated
Pgbd1	3.0453973	0.02370132	Upregulated
Nexn	3.04810585	0.01974424	Upregulated

Gm19935	3.048701	0.04209419	Upregulated
Slco2a1	3.04889682	0.0178242	Upregulated
4833417C18Rik	3.05003692	0.00402522	Upregulated
Gpd1	3.05158631	0.00010377	Upregulated
Gm36949	3.0551067	0.02980081	Upregulated
Scara3	3.06373481	0.01893715	Upregulated
Vwa5b2	3.06388515	0.01881159	Upregulated
Kank2	3.07667137	0.00223024	Upregulated
Gm44806	3.08432838	0.02962086	Upregulated
Dynlt1a	3.0893365	1.35E-06	Upregulated
Cftr	3.08992816	0.04728919	Upregulated
Sod3	3.09534174	0.01659596	Upregulated
Hs3st3a1	3.09573355	0.04690313	Upregulated
P2rx5	3.10333299	0.04062764	Upregulated
Gas6	3.10786419	0.00751197	Upregulated
Serping1	3.11082887	0.02876044	Upregulated
Gm8203	3.12203652	0.02150492	Upregulated
8030456M14Rik	3.12252537	0.04416467	Upregulated
Speg	3.12504072	0.04662447	Upregulated
Tead1	3.12543073	0.03360587	Upregulated
A730063M14Rik	3.12910967	0.01853609	Upregulated
Eda2r	3.13090077	0.03661283	Upregulated
Tmem132a	3.13182137	0.01241498	Upregulated
Pdk4	3.14236381	0.00457771	Upregulated
Tmem91	3.14266217	0.00827909	Upregulated
Myorg	3.14716583	0.01055311	Upregulated
Cox7a1	3.14801843	0.02094528	Upregulated
Frmpd4	3.15582764	0.00997757	Upregulated
Gm53065	3.1630476	0.0316676	Upregulated
Mme	3.16551999	0.01376109	Upregulated
Ckmt1	3.18108091	0.01249624	Upregulated
Gm16287	3.18293112	0.03697612	Upregulated
Grb14	3.18594175	0.01220521	Upregulated
Gm28586	3.19484407	0.04782745	Upregulated
Gm7429	3.19641068	0.03837207	Upregulated
Cntn1	3.20068488	0.01138159	Upregulated
Ppp1r3c	3.20191235	0.0123123	Upregulated
Schip1	3.20396432	0.01129236	Upregulated
Gm47079	3.21170352	0.02262637	Upregulated
Sorbs2	3.21341556	0.00516092	Upregulated
Tmem132e	3.21692252	0.01137913	Upregulated
Aif1	3.2276492	0.01000293	Upregulated
L3mbtl1	3.23047845	0.01167502	Upregulated
Trp73	3.23817502	0.02033561	Upregulated
Kcnn3	3.2468278	0.03123217	Upregulated
Acss3	3.24883649	0.01310281	Upregulated

Aldh3a1	3.26238565	0.03666484	Upregulated
Gm22192	3.26497307	0.0152156	Upregulated
Tnnc1	3.26719331	0.0364619	Upregulated
Gpr27	3.26915101	0.00704235	Upregulated
Arhgef26	3.27145971	0.00697179	Upregulated
Ano1	3.27200545	0.01357284	Upregulated
Gm49417	3.27520237	0.04077411	Upregulated
Igfbp6	3.27738427	0.00668722	Upregulated
Fcna	3.28210655	0.03146203	Upregulated
Sytl5	3.2821753	0.01842695	Upregulated
Unc45b	3.28493237	0.01342848	Upregulated
Gm5617	3.28557863	4.48E-06	Upregulated
Tmem119	3.29641502	0.02036272	Upregulated
Scn4b	3.29681849	0.02424147	Upregulated
Adamtsl3	3.29785719	0.02715575	Upregulated
A930003A15Rik	3.30211089	0.00155653	Upregulated
Ptges	3.30982142	0.02649426	Upregulated
Sapcd2	3.31050978	0.02828739	Upregulated
Pdlim3	3.31549665	0.03101567	Upregulated
Hyal2	3.31673575	0.00469965	Upregulated
Adgrb1	3.31921774	0.00630826	Upregulated
Kif26b	3.32597486	0.00818649	Upregulated
Camsap3	3.32696958	0.00771032	Upregulated
Slc36a2	3.33691777	0.03376737	Upregulated
Fxyd2	3.34293378	0.02180805	Upregulated
Bnc2	3.34463187	0.01864689	Upregulated
Gm4925	3.34762724	0.01686922	Upregulated
Astn1	3.35181852	0.00685062	Upregulated
Mmp15	3.35593409	0.0121334	Upregulated
Gnai1	3.35733578	0.00671133	Upregulated
Kcnj16	3.36588207	0.00745365	Upregulated
Cyyr1	3.36616744	0.01239582	Upregulated
9630028I04Rik	3.36728697	0.03486952	Upregulated
Aqp11	3.37092364	0.03528322	Upregulated
Klhl33	3.37324839	0.00680695	Upregulated
4930570G19Rik	3.37931101	0.00706765	Upregulated
Gpx7	3.38745278	0.00842792	Upregulated
Tcap	3.39167246	0.01516718	Upregulated
Synpo2	3.40122325	0.00516048	Upregulated
Gm25492	3.40595978	0.00337616	Upregulated
Gm4596	3.41503946	0.03293435	Upregulated
Sertad4	3.42569927	0.00424944	Upregulated
Lum	3.42706032	0.01697986	Upregulated
Gm13830	3.43153169	0.01034562	Upregulated
Jsrp1	3.43232925	0.00639544	Upregulated
Lrp2	3.43559418	0.03464527	Upregulated

Gm44524	3.43688904	0.00976809	Upregulated
Gm24920	3.45340472	0.01556835	Upregulated
6030407O03Rik	3.45790282	0.02057589	Upregulated
Kcnt2	3.4588258	0.0050073	Upregulated
Amotl1	3.46094736	0.00198629	Upregulated
B9d1	3.46505539	0.00389348	Upregulated
Cyp1b1	3.48077876	0.00662327	Upregulated
Peg3	3.48136998	0.00435436	Upregulated
Armh4	3.48896243	0.00415136	Upregulated
Eef1a2	3.49351339	0.00417137	Upregulated
Ndrg4	3.49458548	0.0041838	Upregulated
F3	3.50407742	0.00319271	Upregulated
Gm5621	3.51414232	0.00021212	Upregulated
Six4	3.5312255	0.00861332	Upregulated
Hjv	3.53190393	0.0023167	Upregulated
H19	3.53562363	0.04541173	Upregulated
Thrsp	3.5365315	0.00233619	Upregulated
Depdc7	3.53723258	0.00446244	Upregulated
Gm27252	3.54524538	0.01096577	Upregulated
Pter	3.55086404	0.00385449	Upregulated
Gm44907	3.5567072	0.00720718	Upregulated
Insl6	3.5658645	0.0098111	Upregulated
Col22a1	3.5678355	0.0313367	Upregulated
Flnc	3.57580943	0.00250649	Upregulated
Atoh8	3.5791917	0.00791916	Upregulated
2010001A14Rik	3.589423	0.00083247	Upregulated
Pln	3.59806648	0.00931781	Upregulated
Cma1	3.60942665	0.01207589	Upregulated
Igfbp5	3.61278595	1.87E-05	Upregulated
Ptgds	3.63451244	0.03805649	Upregulated
Kif17	3.6401914	0.00262649	Upregulated
Fhod3	3.64762053	0.00265211	Upregulated
F7	3.64839278	0.03616257	Upregulated
Tle6	3.64995668	0.00578715	Upregulated
Mcc	3.65261377	0.0231825	Upregulated
Cgnl1	3.66123538	0.00056494	Upregulated
Fbln1	3.67219317	0.00437441	Upregulated
Bdh2	3.70871627	0.00581929	Upregulated
Plin5	3.71496225	0.01355141	Upregulated
Gm48511	3.71781295	0.00470903	Upregulated
Slc23a1	3.72174285	0.00606158	Upregulated
Usp51	3.72784364	0.00268826	Upregulated
Gm12917	3.73760046	0.02615803	Upregulated
Zfp970	3.74194065	0.00196737	Upregulated
Tma7-ps	3.75702309	0.00304996	Upregulated
C530005A16Rik	3.76621752	0.00354073	Upregulated

1810019N24Rik	3.7801957	0.01268573	Upregulated
Msln	3.78813256	0.03530209	Upregulated
Wfdc2	3.79681566	0.04659133	Upregulated
D5Ertd605e	3.79732508	0.01533653	Upregulated
Gm4833	3.79910483	0.0299333	Upregulated
Gstt1	3.80130256	1.30E-05	Upregulated
Gm7846	3.8081942	0.00829437	Upregulated
Inmt	3.8203522	0.01555465	Upregulated
Efemp1	3.83351984	0.00137928	Upregulated
Ces1d	3.84409444	0.01341296	Upregulated
Mir7675	3.84528438	0.02212264	Upregulated
Sfrp1	3.85133025	0.00105064	Upregulated
Pex11g	3.85366483	0.00531649	Upregulated
Slc26a1	3.85437044	0.00149928	Upregulated
6530409C15Rik	3.85759273	0.00355969	Upregulated
Clec1a	3.85859565	0.00537613	Upregulated
Atp1a2	3.8694898	0.00316341	Upregulated
Gm47861	3.88919181	0.02697912	Upregulated
Klhdc7a	3.89080088	0.00697041	Upregulated
Lamb1	3.89652041	0.00173378	Upregulated
Kcnk3	3.9055762	0.00086926	Upregulated
Cyp2j6	3.910727	0.00447646	Upregulated
Mybpc1	3.96008847	0.00889954	Upregulated
Dpt	3.96696565	0.00424492	Upregulated
Hspb8	3.9764633	0.0010587	Upregulated
Bnip5	3.97761962	0.00084958	Upregulated
C1ra	3.99127447	0.00792441	Upregulated
Vmn1r6	4.01947877	0.02675917	Upregulated
Trpm1	4.03231228	0.01479812	Upregulated
Pgam2	4.03428588	0.00998089	Upregulated
Slc4a4	4.04809641	0.00045266	Upregulated
Des	4.051382	0.00294305	Upregulated
Podn	4.08190812	0.00132177	Upregulated
Clmn	4.08414453	0.00039219	Upregulated
Gm14327	4.09044204	0.00174516	Upregulated
Kcnab3	4.10545931	0.00035881	Upregulated
Mgp	4.10573841	0.00166429	Upregulated
Sema3a	4.11660039	0.00035161	Upregulated
Myl1	4.12054347	0.01091764	Upregulated
Usp13	4.12955578	0.0003002	Upregulated
Mertk	4.13785876	0.00196231	Upregulated
Tln2	4.15078302	0.00361334	Upregulated
Dcn	4.15769962	8.02E-05	Upregulated
Dnah9	4.16959687	0.00617985	Upregulated
Gm10687	4.17585564	0.00355508	Upregulated
Zfp385c	4.18109419	0.00132375	Upregulated

Flrt2	4.18464963	0.00024911	Upregulated
Lpar1	4.18564032	0.00025901	Upregulated
Gpx3	4.20184837	0.00089739	Upregulated
Cd248	4.21583052	0.00428356	Upregulated
Ltc4s	4.21752492	0.00453571	Upregulated
Cidea	4.24565208	0.00466259	Upregulated
Gm43698	4.27305893	0.00487119	Upregulated
Ptgr1	4.29493245	0.00073872	Upregulated
Pkia	4.30242897	0.00014745	Upregulated
Sec14l3	4.30836047	0.01033168	Upregulated
Car3	4.31546125	0.00059125	Upregulated
Nkain4	4.32029306	0.00013095	Upregulated
Cmya5	4.35407296	4.15E-06	Upregulated
Dnah5	4.38503085	0.00434727	Upregulated
Gm26670	4.39783616	0.00317719	Upregulated
Saa3	4.40226869	0.04639111	Upregulated
Sulf1	4.42289142	0.00019034	Upregulated
Mpo	4.45228185	0.03956098	Upregulated
Bcam	4.46985028	0.00102317	Upregulated
Aebp1	4.49526758	0.00020077	Upregulated
Il33	4.50808575	0.00011989	Upregulated
C1s1	4.53154435	0.00312267	Upregulated
Tgfb2	4.56156974	0.00062416	Upregulated
Rarres2	4.57210631	0.00064367	Upregulated
Myh6	4.63971197	0.00732063	Upregulated
Gpm6a	4.68502323	2.21E-05	Upregulated
Plin1	4.69108607	0.00290178	Upregulated
Cxcl13	4.72867802	0.00736591	Upregulated
Pck1	4.74724554	0.00161217	Upregulated
Arhgdig	4.75326578	0.00132432	Upregulated
Ighg2c	4.7685807	0.00209402	Upregulated
Pvalb	4.78413081	0.00203092	Upregulated
Acta1	4.79535526	3.44E-06	Upregulated
Cidec	4.80517594	0.03281801	Upregulated
Ttn	4.83878248	4.18E-11	Upregulated
Dab2	4.90195454	0.0001477	Upregulated
Rp1	5.05015043	0.00301724	Upregulated
Rapsn	5.32621318	0.0002536	Upregulated
Scgb3a1	5.42980377	0.03750871	Upregulated
Myh1	5.4800388	2.04E-07	Upregulated
Garnl3	5.48680958	2.97E-05	Upregulated
Scrn1	5.49658983	0.00013088	Upregulated
Calml4	5.55464287	2.74E-05	Upregulated
C1qa	5.55846331	2.19E-05	Upregulated
Prg4	5.65332038	2.13E-05	Upregulated
Xirp2	5.70099128	0.00114292	Upregulated

Cyp2a5	5.70151866	0.00534586	Upregulated
Mylk4	5.74207473	0.00072843	Upregulated
Ptgis	5.74852327	0.00024229	Upregulated
C1qb	5.77180007	6.10E-06	Upregulated
Actn3	5.9305274	6.95E-07	Upregulated
Me1	5.9534982	4.12E-06	Upregulated
Atp2a1	6.01006474	4.78E-07	Upregulated
Tnnc2	6.12418332	0.01362203	Upregulated
Retnla	6.23847516	0.00161603	Upregulated
Ucp1	6.24633044	0.00048831	Upregulated
C1qc	6.40860625	1.83E-06	Upregulated
Cd5l	6.49799301	0.02637509	Upregulated
Ednrb	6.70250639	3.08E-05	Upregulated
Myh2	6.79798068	1.06E-05	Upregulated
Bpifa1	7.400233	0.00542935	Upregulated
Ckm	7.51860901	0.00087074	Upregulated
C4b	7.74800507	7.92E-08	Upregulated
Myh4	8.52950324	2.74E-09	Upregulated
Cyp2f2	21.0425228	4.01E-29	Upregulated
Rag2	39.4371189	1.89E-19	Upregulated

Table of Contents Top of Current Table

Supplemental Table 7. List of all overlap of significant ($P < 0.05$) differentially expressed genes in whole blood of 3xTg-AD CBD (week 0 to 8) and 3xTg-AD vehicle (week 0 to 8) animals. [%]Commonly downregulated in 3xTg-AD (vehicle and CBD) and B6129 (CBD only); [#]Commonly downregulated in 3xTg-AD (vehicle & CBD) but upregulated in B6129 (vehicle only); [##]Commonly downregulated in the 3xTg-AD (vehicle & CBD) but upregulated in the B6129 (CBD only); *Commonly upregulated in 3xTg-AD (vehicle & CBD) and B6129 groups (vehicle & CBD); [†]Commonly upregulated in 3xTg-AD (vehicle & CBD) and B6129 (vehicle only); [††]Commonly upregulated in 3xTg-AD (vehicle & CBD) and B6129 (CBD only) groups; ‡Commonly upregulated in 3xTg-AD (vehicle only) and B6129 groups (vehicle & CBD); [+++]Commonly upregulated in 3xTg-AD (CBD only) and B6129 (CBD only) but downregulated in 3xTg-AD (vehicle only); [$]Commonly upregulated in 3xTg-AD (vehicle and CBD) but downregulated in the B6129 (vehicle only); [$$]Commonly upregulated in the 3xTg-AD animal groups (vehicle & CBD) but downregulated in the B6129 (CBD only)

Common Genes Downregulated	Common Genes Upregulated	Common but Opposite: Downregulated in 3xTg-AD CBD & Upregulated in 3xTg-AD Vehicle	Common but Opposite: Upregulated in 3xTg-AD CBD & Downregulated in 3xTg-AD Vehicle
5S_rRNA[#]	1700007L15Rik	Rgs7bp	7SK or RN7SK; snRNA, RNA component of 7SK nuclear ribonucleoprotein
6230400D17Rik	4933412O06Rik	Smad9	Peg3[+++]
8430422M14Rik	6030407O03Rik		Tagln3
Adamts14	6530409C15Rik		
Fuom[%]	Abca8a		
Galnt15	Acot13		
Gm11440	Acot6		
Gm13868	Acss3		
Gm19046	Actn3		
Gm37529	Aebp1[†]		
Gm37663	Atp1a2		
Gm43072	Atp5j2		
Gm43329	Atp5mpl		
Gm44664	Bcam[†]		
Gm44695	C1qa*		
Gm44888	C1qb*		
Gm47594	C1qc*		

Gm49086	C1s1		
Gm49201	C4b*		
Gm49616	Calr3		
Map2k3os	Car3*		
Susd4[%]	Ccdc167		
Tdrd5[##]	Cd248		
	Ces1d[††]		
	Cgnl1*		
	Cidea[††]		
	Cidec[††]		
	Clmn		
	Col14a1		
	Cox7a2		
	Cript		
	Cyp1b1[††]		
	Cyp2f2[††]		
	Dab2		
	Dagla		
	Dcn		
	Ddr2		
	Depdc7		
	Des[†]		
	Dph3		
	Dpt[††]		
	Eda2r		
	Ednrb*		
	Efemp1[†]		
	Fblim1		
	Fbln1		

Fcna[†]	
Fgf2	
Flrt2[†]	
Gas6	
Gm10076	
Gm14327	
Gm19935[†]	
Gm5617	
Gm6055	
Gm7536	
Gpd1*	
Grb14	
Igfbp5	
Il33[†]	
Inmt	
Jam3	
Kcnk3[†]	
Kcnn3	
Kcnt2	
Klhdc7a	
Lockd	
Lpar1	
Ltc4s[†]	
Lurap1	
Map1lc3b	
Me1	
Mertk	
Mgl2[†]	
Mgp	

Micos13
Mme
Mrc1
Myh4[††]
Naa38
Ndufa7
Ndufb4
Pck1[††]
Pdk4*
Pfdn5
Plin1[††]
Ppp1r3c
Prelp
Prg4*
Prox1
Ptgis*
Ptgr1
Pth1r[$$]
Ptprd
Rag2
Rarres2
Rbms3
Retnla*
Rpl17-ps8
Saa3[†]
Scara3
Sec14l3
Serping1
Sf3b6

	Sfrp1		
	Shisa2[$]		
	Slc36a2		
	Slc4a4		
	Snrnp25		
	Spats2		
	Sulf1		
	Synpo2		
	Sytl5		
	Thrsp		
	Timm8b		
	Tm4sf1		
	Tmem119		
	Tmem91		
	Tmsb4x		
	Ucp1*		
	Uqcr10		
	Uqcrh		

Supplemental Table 8. List of all differentially expressed genes (P<0.05) in whole blood of 3xTg-AD vehicle (week 0 to 8) that were reversed in expression in 3xTg-AD CBD (week 0 to 8) animals in comparison. Italicized genes have a recognized association with Alzheimer's disease pathology; [&]Genes commonly upregulated or downregulated in wild-type B6129 vehicle animals and may be omitted from interpretation as 3xTg-AD specific DEGs *P<0.05, significant expression in the opposite direction in 3xTg-AD CBD

DEG (P<0.05): 3xTg-AD	log2FoldChange: 3xTg-AD	log2FoldChange: 3xTg-AD CBD	P-value: 3xTg-AD CBD
H2-Q2	-16.77560335	0	1
Gm37607	-4.645104559	1.973449795	0.129190195
Gm28941	-4.505416239	0.798708074	0.781230119
ENSMUSG00002075672	-4.314848177	0.621120669	0.970194623
Gm9754	-4.284092449	0.42013514	1
BC016579	-4.265047007	1.080777057	0.565805043
Sema4c	-4.24253536	0.978277919	0.593650521
2510017J16Rik	-4.197342434	0.093816447	1
1600022D10Rik	-4.103015179	1.064588136	0.777738343
Mir342	-4.028353097	0.657264919	0.95097387
Gm17096	-4.028120351	0.429114556	1
Trbj1-6	-4.015905086	0.297669798	1
Gm44704	-3.962546629	0.424849276	1
Gm7054	-3.929350232	0.88837272	0.78453042
Pcdhgb7	-3.886986898	2.446184531	0.077447097
Gm43609	-3.835702221	1.513796893	0.447708571
2900078I11Rik	-3.818910655	1.752074749	0.324736593
Gm49706	-3.81756573	0.651431488	0.939159123
Traj22	-3.797700348	0.794326719	0.884531168
Gm11725	-3.759144304	2.047141524	0.181795997
Gm26064	-3.758243305	1.194674152	0.525284021
Gm37062	-3.741751314	1.323519115	0.41783508
Gm49169	-3.726783647	0.033797568	1
A3galt2	-3.713161281	0.485063986	1
Gm29055	-3.707065942	1.55167692	0.440741578
2610028D06Rik	-3.701505661	0.288360445	1
Gm37509	-3.682914104	0.595084071	0.991978049
Gm16794	-3.676380325	0.080113336	1
Gstp-ps	-3.668764423	0.491409543	1
Gm25128	-3.646312414	0.534199772	1
Gm6044	-3.637407146	1.912624286	0.306384725
*Peg3**	-3.628717522	3.481369977	0.004354363
Gm43075	-3.599343211	0.636055077	0.955982598
Tchh	-3.557728403	1.841640342	0.215459661
Gm37519	-3.534987162	0.531318206	1
Syt15	-3.528002907	0	1

Gm12902	-3.511693182	1.84424667	0.356581569
Stk36	-3.511275618	1.8836898	0.32323136
1810012K16Rik	-3.48720857	0.664251289	0.946599932
Synm	-3.478758154	1.270643279	0.242211122
4930579K19Rik	-3.420217645	0.328881315	1
Olfr1393	-3.41338819	0.54324178	1
Pbx4	-3.40968696	0.040407434	1
Gm15549	-3.374156447	0.03734304	1
Gm26109[&]; snoRNA	-3.355250696	0.025947245	1
Iqck	-3.35299091	0.686998745	0.901073103
Gm44835	-3.349553731	0.594616222	0.993401386
Gm43024	-3.319139122	0.355682542	1
*Tagln3**	-3.31669466	2.886727479	0.015372481
Crmp1	-3.316009774	0.965317315	0.689801099
Gm44008	-3.257864209	1.65835164	0.34120128
Gm47113	-3.251054658	0.217048306	1
Gm49326	-3.242460617	0.480242125	1
Gm38021	-3.239373428	0.080714771	1
Rnft2[&]	-3.232531474	0.613074436	0.982725396
Gm18537	-3.232135581	1.579333686	0.371468517
Rin1	-3.210932244	0.434045978	1
Gm15672	-3.203565253	0.825779146	0.813905019
Gm19269	-3.189171305	0.500623724	1
7SK	-3.187273204	1.725727814	0.435070389
Gm25821	-3.185151552	0.648768924	0.935260507
Gm16074	-3.181054009	0.722625841	0.903614632
Tmem240	-3.164638786	1.0012246	0.497216388
A130050O07Rik	-3.15877975	0.368427188	1
Prss36	-3.157230515	1.146265757	0.405692444
Rac3	-3.133905528	0.59171266	0.995800858
Gm7666	-3.102724703	0.895086082	0.673149982
Dhrs13	-3.083811334	0.046396899	1
Runx2os1	-3.072827666	0.781867717	0.874720895
9530022L04Rik	-3.056985934	0.380719631	1
C78859	-3.05478493	0.490486508	1
A930007I19Rik	-3.039998876	0.461680511	1
Gm3081	-3.039136337	0.285247195	1
Gm50387	-3.036843437	0.036847213	1
Gm15445	-3.034048525	0.449136754	1
Gm43389	-3.030633876	0.763584119	0.802007709
Mamdc2	-3.029147589	0.54126566	1
6430571L13Rik	-3.026253942	2.077528573	0.208849084
Gm45532	-3.018019597	0.6140377	0.98403455
Ablim3	-3.016569351	0.317006012	1

Gm28707	-3.012942755	1.41467443	0.428858421
Wnt10a	-3.00898024	0.716457516	0.918602573
Tagln	-3.007379653	0.238136219	1
Srd5a1[&]	-3.004397611	0.59364982	0.994616995
5S_rRNA	-2.977545949	1.975132898	0.268958156
Acvr1	-2.972012735	0.30913924	1
Ramp3	-2.955000191	2.189622818	0.076533925
6430710M23Rik	-2.952664441	2.110218686	0.154552263
Gm45234	-2.950745862	1.291153656	0.597570335
Sgtb[&]	-2.936924268	0.406309844	1
Pcdhga9	-2.926133701	0.017015773	1
Gm44041	-2.915583698	0.848672034	0.764689076
Gm17191	-2.911586128	0.049498353	1
Lbhd1	-2.889516774	0.515892646	1
Egfl7[&]	-2.872421728	1.304663614	0.434593409
Gm15538	-2.869564726	0.612606542	0.984061978
Gm37255	-2.868771027	0.964764526	0.558386575
Gm47980	-2.862305238	0.042753327	1
Egfl6	-2.859179712	1.183844696	0.596533418
Mettl5os	-2.840022955	2.416420307	0.116789537
Gm36757	-2.836101972	0	1
Fam110d[&]	-2.83159039	0	1
A530064N14Rik	-2.816476227	1.292180708	0.423616578
Igkv3-12	-2.799023497	0.100519876	1
Dach2	-2.783029745	1.887162014	0.0894604
Gm13270	-2.7696026	0.388079768	1
Fth-ps2	-2.755194004	0.366658668	1
Gm42872	-2.739367784	1.287187439	0.416165839
Tox3	-2.736388812	0.493275354	1
Gm15545	-2.711033131	0.006208487	1
Rian[&]*;* lncRNA	-2.702682719	0.586873989	0.998815378
Gm12743	-2.70146019	0.499884852	1
Mir1955[&]; miRNA	-2.69199692	2.016893152	0.157914847
Efna1[&]	-2.691530613	0.771108077	0.811831215
Gm48420	-2.683880892	1.068592937	0.694474944
Gm43111	-2.677896211	0.493132828	1
Gm44053	-2.670281355	0.852273865	0.779398124
Gm37115	-2.657646848	0.88441283	0.518575708
Gm45206	-2.653919623	0.570161212	1
Mrnip	-2.642557595	0.291368686	1
Ptk7	-2.594370101	1.648652613	0.380095013
Cpa3	-2.576153558	1.22924379	0.407884064
Frs3	-2.526939977	0.71797835	0.897270519
Gm37390	-2.486996548	0.713723266	0.864759299

Sparcl1	-2.478358481	1.054926297	0.550130928
Gm47246	-2.454813779	0.435464604	1
Scg5	-2.4037173	0.142115256	1
4930556H04Rik	-2.379660011	0.007907996	1
Ighj2	-2.379523695	0.169311094	1
Xrcc3	-2.376298084	0.589440577	0.995518245
Crip3	-2.284204663	0.40862119	1
Gm37139	-2.237391437	0.64510527	0.915519194
Igkv8-19	-2.170461732	0.782125526	0.776995506
Gm49396	-2.102784354	0.80456449	0.732592164
Krtcap3	-2.08583467	0.120933559	1
Zfp202	-2.080613612	0.558556363	1
Zfp775	-2.068555033	0.011865667	1
2700062C07Rik	-2.01012105	0.337921789	1
Gm37019	-1.971132956	0.495158752	1
Gm37677	-1.915571942	0.103137992	1
Arl4d	-1.913102764	0.896393976	0.597207107
Gm9796	-1.897450133	0.102650581	1
Gm17690	-1.852892955	0.61530769	0.949762958
Esr1	-1.851838849	0.036658249	1
C130089K02Rik	-1.782519983	0.141797152	1
Gm42856	-1.724111267	0.486169332	1
Gm29488	-1.699074466	0.483691216	1
Gm38372	-1.695258505	0.148181319	1
Gm37906	-1.673038466	0.170188006	1
Fam167a	-1.608653944	0.372443549	1
2410004B18Rik	-1.536060044	0.261829256	1
Gm6712	-1.372660705	0.340071047	1
Apex1	-1.354231598	0.624286479	0.900048648
Ccsap	1.323257374	-0.726971015	0.740811169
Rgs8	1.951258432	-0.215252267	1
Arhgef5	2.212821059	-1.025023646	0.548247404
Adamts17	2.321310853	-0.656722784	0.926301513
Sh3pxd2b	2.329396541	-0.329015562	1
Phf24	2.400720971	-1.150111434	0.426765314
Cabcoco1	2.417215228	-1.968135633	0.277720631
Tmem88b	2.494270634	-0.353076451	1
Ica1l	2.498389246	-1.175784335	0.261578042
Lag3	2.519540916	-0.609929084	0.972819557
Xkr5	2.565222871	-0.665010611	0.927639164
Gm47493	2.620603191	-0.811512702	0.838840279
Thbs2	2.623639538	-1.077750159	0.573971518
Mroh8	2.625219635	-0.705737577	0.882882916
Chst7	2.677866851	-1.398719672	0.481603029

Tgm1	2.746148327	-0.002786096	1
Syt16	2.787183848	0	1
Vgf	2.915554576	-0.541006444	1
Gpr6	2.918543314	0	1
Gm16701	2.919580847	-1.002202931	0.730220214
Oprd1	2.92227043	0	1
Trpc3	2.92381771	-0.541006453	1
Ntsr1	2.923912187	0	1
Wnk3	2.925403632	-0.541006454	1
Masp1	2.925525611	0	1
Nap1l2	2.925805633	0	1
Kndc1	2.925931487	-0.459735379	1
Pcsk2	2.925970312	0	1
Fam171b	2.926013462	0	1
Cckbr	2.926108699	0	1
Mab21l1	2.926340976	0	1
Pcsk5	2.926812822	-1.104380308	0.682255288
Syt10	2.927146583	0	1
Gm48822	2.927209212	0	1
Gm5067	2.927268756	0	1
Gm973	2.929873821	0	1
Sat2	2.933776193	0	1
Fam110c	2.934879266	-0.097705903	1
Gm15663	2.943974702	-1.8026746	0.233033852
Prkar1b	2.945959207	-1.581086729	0.408387364
Cldn10	2.979488787	-1.590689212	0.40667688
Agbl2	3.060790798	-0.420387214	1
Coro2b	3.154370908	-1.104856162	0.681535557
Dock3	3.15623935	-0.525277491	1
Foxc1	3.161369251	-0.443359899	1
Gm49894	3.163886357	-0.541006345	1
1700016P03Rik	3.217343421	0	1
Lyplal1	3.226097526	-1.715065421	0.261871302
Mei1	3.23661196	0	1
Cfap91	3.237760929	0	1
Tacr3	3.238552898	0	1
Fabp3[&]	3.241155622	0	1
Eps8l2	3.244241581	-2.144160322	0.159856606
Gucy2c	3.246379704	-2.151774103	0.222684031
Fgf14	3.246456591	0	1
*Smad9**	3.247241351	-2.953775734	0.023861531
Lin7a	3.247758746	0	1
Gria4	3.247791036	-0.541006454	1
Slc45a1	3.248081791	-2.26664598	0.132493186

Gm15666	3.250397255	-0.459274869	1
Tox2	3.25282426	-0.107687692	1
Lmcd1	3.260255278	-0.828358697	0.830758974
Medag	3.273032237	0	1
Pth2r	3.293342737	0	1
Tmem63c	3.347228335	0	1
Cdk18;<br>or Pctk3 or Pctaire3	3.347842856	0	1
Slc4a10	3.353477318	-0.201679282	1
Spaca6	3.358680284	-0.191009482	1
Gm28050	3.369661653	-0.918954645	0.827023501
Gldn	3.432245071	0	1
Aoc3	3.447728588	-0.74949111	0.850932445
Gm11653	3.471650294	0	1
Zfp449	3.491140313	-0.965063642	0.622386321
C230031I18Rik	3.495143317	0	1
Rsph14	3.495687577	0	1
Elovl4	3.496331702	0	1
Pm20d1	3.497186611	-0.541006442	1
Thbs4	3.501555664	-0.437475336	1
Gm30015	3.506607584	0	1
Insm1	3.507531315	0	1
Gm37363	3.512570918	0	1
Bmp6	3.51281256	-1.001554068	0.730791436
Gm8173	3.513524959	0	1
St8sia3	3.513625332	-0.528232954	1
Asic4	3.515502906	-1.180578807	0.54607504
Gm42899	3.516855333	0	1
Ccdc81	3.519919778	-1.116490095	0.686003192
Kcnk12	3.520449513	0	1
Gm44848	3.520590204	0	1
Flrt3	3.520828146	-0.541006453	1
Alox8	3.524128727	-2.165330062	0.117628814
Gm15910	3.526948804	-1.923775777	0.298401232
4933424G06Rik	3.554025081	0	1
Gm37953	3.569537952	-0.872312996	0.815138318
Gm42669	3.638045061	-0.192912873	1
Bicc1	3.651034645	-0.037264801	1
Ttll9	3.681403676	0	1
Gm49069	3.726423796	0	1
Ppfia2	3.729692553	-1.132867492	0.611665948
Dab1	3.732807823	0	1
Ccdc142	3.783099052	-0.943390952	0.796954107
Fbl-ps2	3.784362761	-0.506456969	1

Pdcd5-ps	3.820891708	-2.004471087	0.226064055
Tpbgl	3.874930479	-1.077675059	0.646998907
Scn7a	3.904116868	0	1
Chrna2	3.908325318	0	1
Gm50341	3.914983778	-0.268256317	1
*Rgs7bp**	3.922931953	-3.056265575	0.013271907
Chadl	3.926964273	-1.952316922	0.238693869
Gm14863	3.93336494	-1.200405024	0.739020178
Gm13479	3.935508933	-0.579491889	1
5330439K02Rik	3.963030069	-1.726475887	0.252112581
G530011O06Rik	3.967702827	0	1
Kcnk2	4.093394437	0	1
Rorb	4.097416943	-1.975380188	0.164400765
Fgfr3	4.104070437	0	1
Gm48894	4.129137133	-2.421728564	0.17959425
Slc13a4	4.156073569	0	1
B3gnt9	4.183075741	-1.024638309	0.671106096
Tepp	4.188499967	0	1
Mgat3	4.248113614	0	1
Tspan11	4.252082871	0	1
Chmp4c	4.264068442	0	1
Ak9	4.334247184	-1.350042845	0.544783246
Vamp9	4.517045715	-0.337106303	1
Fam217a	4.611761457	-0.541006185	1
Srl	4.670662681	-0.054474289	1
Zmynd15	4.864975383	-0.534093044	1
Clstn3	4.988196991	-2.260786468	0.087643819
Ncan	5.083779853	-0.52927144	1
Sult4a1	16.81418274	-1.018261458	0.827300197

Table of Contents Top of Current Table

Supplemental Table 9. List of all significant (P < 0.05) differentially expressed genes in whole blood of wild-type B6129 CBD (week 8) versus wild-type B6129 CBD (week 0) animals; 338 genes downregulated & 198 upregulated.

Gene Name	Log2FoldChange	P-value	Regulation
Lor	-6.40292788	7.85E-07	Downregulated
Krt1	-5.03378706	0.00059264	Downregulated
Krt2	-4.94572794	0.00070447	Downregulated
Ebf3	-4.53756449	0.00091177	Downregulated
Amn	-4.41903306	0.0003862	Downregulated
Krt25	-4.39586506	0.0085188	Downregulated
Postn	-4.32345583	0.0030933	Downregulated
Syt15	-4.32083938	0.00904604	Downregulated
Tchh	-4.26366687	0.00445793	Downregulated
Dnah14	-4.24914896	0.00176131	Downregulated
Gm38009	-4.24726226	0.00116259	Downregulated
Meox1	-4.23956455	0.00265386	Downregulated
ENSMUSG00000115801	-4.22463237	0.01876413	Downregulated
Arpin	-4.1853587	0.00019883	Downregulated
Grip2	-4.17179541	0.00047408	Downregulated
Fuom	-4.16966119	0.00021942	Downregulated
Trp63	-4.15322991	0.00230444	Downregulated
Gsg1l	-4.14434772	0.00026496	Downregulated
Galnt3	-4.1385622	0.00085547	Downregulated
Dcst1	-4.10590894	0.04169763	Downregulated
Csmd1	-4.09634137	0.00030306	Downregulated
Calcb	-4.08447699	0.00342805	Downregulated
Hmcn1	-4.0690068	0.00194031	Downregulated
Zfp786	-4.04913117	0.00039336	Downregulated
Pcdh17	-4.0432173	0.00027467	Downregulated
Slc12a8	-3.96444343	0.00076822	Downregulated
Pth1r	-3.95584178	0.00071165	Downregulated
Krt10	-3.94194311	0.00010379	Downregulated
A930029G22Rik	-3.89782971	0.0015304	Downregulated
Kcnk9	-3.89357709	0.00071214	Downregulated
Il20ra	-3.86550584	0.00264893	Downregulated
Gm44735	-3.86264319	0.00336076	Downregulated
1700120B22Rik	-3.77838951	0.01243792	Downregulated
Dnai3	-3.75539046	0.02458368	Downregulated
Susd4	-3.75505961	0.00122276	Downregulated
Pex11g	-3.75040977	0.00554073	Downregulated
Zfp13	-3.74716816	0.00127316	Downregulated
Gm46409	-3.74509179	0.00508133	Downregulated
A930037H05Rik	-3.73977646	0.00691198	Downregulated
Gm9967	-3.737715	0.00307921	Downregulated
Slc5a1	-3.73573009	0.00691957	Downregulated

Otoa	-3.70912847	0.00668392	Downregulated
Abcc8	-3.70686055	0.00167206	Downregulated
Cyp26b1	-3.70606364	0.00247281	Downregulated
Fam83h	-3.68438193	0.00604542	Downregulated
C130074G19Rik	-3.6798225	2.05E-08	Downregulated
4632428C04Rik	-3.67819733	0.00329892	Downregulated
Gm10719	-3.66711402	0.04271781	Downregulated
Asic1	-3.65684721	0.00185145	Downregulated
Gm28592	-3.60574031	0.02183387	Downregulated
Slc9a2	-3.60244108	0.00273911	Downregulated
Radil	-3.5926789	0.00285368	Downregulated
Fa2h	-3.58381573	0.0024513	Downregulated
Col2a1	-3.58212581	0.02520764	Downregulated
Adcyap1r1	-3.57019594	0.00252227	Downregulated
Lrrc18	-3.56282419	0.00753149	Downregulated
Dpp10	-3.56153905	0.00271526	Downregulated
Robo1	-3.54399198	0.00218176	Downregulated
Cdsn	-3.53482596	0.04254676	Downregulated
Dct	-3.5261141	0.02309615	Downregulated
Ecscr	-3.48193731	0.01795834	Downregulated
Rasd1	-3.47917515	0.00545301	Downregulated
Kcnk2	-3.47544916	0.00379339	Downregulated
Esyt3	-3.47518149	0.00982014	Downregulated
Sgsm1	-3.47428424	0.00546736	Downregulated
Adgrb3	-3.47409903	0.00359663	Downregulated
Fras1	-3.47253749	0.00495175	Downregulated
Gpld1	-3.47020914	0.00380398	Downregulated
Gpr63	-3.46917459	0.00388008	Downregulated
Ift43	-3.46573132	0.00410961	Downregulated
Gm43868	-3.46032639	0.01076696	Downregulated
Pkdrej	-3.45012305	0.00994169	Downregulated
Gm14279	-3.44452388	0.04441111	Downregulated
Gm26788	-3.43971357	0.02919135	Downregulated
Gm5086	-3.43721185	0.01182011	Downregulated
Grm1	-3.42670488	0.00344196	Downregulated
Gm38352	-3.42446928	0.02630425	Downregulated
Iqank1	-3.40764479	0.0100224	Downregulated
Fam110d	-3.39499404	0.01111032	Downregulated
Gm42440	-3.3946277	0.01185161	Downregulated
Kcnj12	-3.36825366	0.00558025	Downregulated
Gabrb1	-3.36785563	0.00529334	Downregulated
Tmem72	-3.36578493	0.03468861	Downregulated
Kl	-3.36494779	0.01102974	Downregulated
Tmem132e	-3.35244904	0.00601102	Downregulated
Chrm1	-3.34813501	0.00598383	Downregulated
Crhr1	-3.34212223	0.00621384	Downregulated

Gm25632	-3.33624797	0.0359857	Downregulated
Trpv3	-3.33169023	0.01445862	Downregulated
Zfhx2os	-3.31807101	0.00891006	Downregulated
Scel	-3.30978557	0.00879646	Downregulated
Vdr	-3.30693181	0.0361555	Downregulated
Nr2f2	-3.30144064	0.00151516	Downregulated
ENSMUSG00002076243	-3.30120238	0.04085074	Downregulated
Sncg	-3.2769212	0.01753609	Downregulated
Tymp	-3.27127258	0.03936834	Downregulated
Thsd1	-3.26079832	0.00410606	Downregulated
Barx2	-3.25960456	0.01173972	Downregulated
Col17a1	-3.258765	0.02531706	Downregulated
Gm10717	-3.25734749	0.0391932	Downregulated
Grip1	-3.25491608	0.00813204	Downregulated
Dlgap1	-3.25414451	0.00630352	Downregulated
Pcdhgc5	-3.25334159	0.00839499	Downregulated
Tmem132d	-3.25190728	0.00810865	Downregulated
Gabbr2	-3.25005989	0.00833922	Downregulated
Gm43182	-3.24819038	0.04280934	Downregulated
Gm48600	-3.24602439	0.01022046	Downregulated
Nrxn3	-3.24446854	0.008386	Downregulated
Pnma2	-3.23743504	0.00867798	Downregulated
Fgd1	-3.23712406	0.00891785	Downregulated
Gpr3	-3.23638276	0.01920338	Downregulated
Lhx6	-3.23606824	0.00967106	Downregulated
Mirg	-3.22535327	0.00933466	Downregulated
Scarf2	-3.22206956	0.01152184	Downregulated
Ccdc63	-3.21296943	0.01881873	Downregulated
Tnnt2	-3.20795425	0.00943269	Downregulated
Xlr3a	-3.2079095	0.0279111	Downregulated
Gm10801	-3.18429181	0.00394126	Downregulated
Zfp423	-3.18008978	0.00516806	Downregulated
Dnah7c	-3.16949136	0.01344301	Downregulated
Ror2	-3.16262421	0.0221671	Downregulated
Catsperg2	-3.14590912	0.0399192	Downregulated
Gm43609	-3.1432624	0.01117453	Downregulated
Papln	-3.14234135	0.02420858	Downregulated
Dipk1b	-3.1311318	0.01218898	Downregulated
Sox9	-3.13036578	0.01239137	Downregulated
Fbxo41	-3.12900046	0.01199456	Downregulated
Osbpl10	-3.12661997	0.00297344	Downregulated
Lrrc4	-3.12660807	0.01222059	Downregulated
Mapk10	-3.12392318	0.01227809	Downregulated
Grid2	-3.12349808	0.01230806	Downregulated
Bmper	-3.12260748	0.01282989	Downregulated
Wnt7b	-3.12223956	0.01256683	Downregulated

Gm37397	-3.12200178	0.01013477	Downregulated
Cemip	-3.12106661	0.01248083	Downregulated
Nell1	-3.11734882	0.01275	Downregulated
Frem2	-3.11714329	0.01123175	Downregulated
Gpc5	-3.11688843	0.01278378	Downregulated
Vat1l	-3.11508952	0.0131677	Downregulated
Gm5421	-3.11492443	0.03916389	Downregulated
Prr15	-3.11237085	0.02890162	Downregulated
C130075A20Rik	-3.11196511	0.01385765	Downregulated
Plekhb1	-3.11136216	0.01382511	Downregulated
Cntnap3	-3.11099622	0.03205382	Downregulated
Slco1a4	-3.1060579	0.01388058	Downregulated
Dgkb	-3.10535768	0.01366222	Downregulated
Pcdhga11	-3.10493764	0.01369556	Downregulated
Scn8a	-3.09197274	0.04309574	Downregulated
Sez6	-3.08964791	0.01130179	Downregulated
Alk	-3.08811218	0.01596726	Downregulated
Lama2	-3.08571311	0.01126068	Downregulated
Fhod3	-3.07972465	0.01257508	Downregulated
Tmem151a	-3.07448876	0.01179261	Downregulated
Frmd3	-3.06506644	0.01729	Downregulated
B230206H07Rik	-3.05872678	0.01794979	Downregulated
Gm47484	-3.05769318	0.01956634	Downregulated
L3mbtl1	-3.0517973	0.01395908	Downregulated
Aspn	-3.04106778	0.02970556	Downregulated
Gm16570	-3.04043366	0.04925424	Downregulated
Cldn10	-3.02015381	0.00524719	Downregulated
Gm27252	-3.01879574	0.03719442	Downregulated
Serinc2	-2.99328031	0.02445887	Downregulated
Proca1	-2.99279406	0.02283177	Downregulated
Myt1l	-2.99023759	0.01869658	Downregulated
Bcar1	-2.9877417	0.01963127	Downregulated
Dab1	-2.98756277	0.01884669	Downregulated
Myt1	-2.98660095	0.01931025	Downregulated
Asphd2	-2.98465156	0.01937115	Downregulated
Adgra1	-2.98215709	0.01940986	Downregulated
Lgi3	-2.97953104	0.01968993	Downregulated
Ntng1	-2.97882616	0.01976587	Downregulated
Igsf10	-2.97871649	0.02276842	Downregulated
Srrm4	-2.97803388	0.0198516	Downregulated
Hhipl1	-2.97765484	0.01989282	Downregulated
Celf6	-2.9769403	0.02004785	Downregulated
Nkain3	-2.97333903	0.02036797	Downregulated
Cdh12	-2.97125449	0.02060169	Downregulated
Col24a1	-2.96289488	0.03575411	Downregulated
Gm37411	-2.95869688	0.02605689	Downregulated

Slc26a1	-2.95411514	0.01586441	Downregulated
Nr2e1	-2.94287077	0.02409108	Downregulated
Pitpnm3	-2.92950819	0.011863	Downregulated
Ano5	-2.92700656	0.02631215	Downregulated
Mpdz	-2.92684489	0.01191911	Downregulated
Slc22a17	-2.92263578	0.01252206	Downregulated
Igkv1-133	-2.91740553	0.01094019	Downregulated
Serpina3f	-2.91515212	0.01764634	Downregulated
Peg3	-2.89717579	0.02017826	Downregulated
Gpr37	-2.89501121	0.02028982	Downregulated
Ephb1	-2.88623765	0.02099857	Downregulated
Npc1l1	-2.8850808	0.04043308	Downregulated
Gm20501	-2.87974092	0.03319526	Downregulated
Tead2	-2.87706565	0.03169133	Downregulated
Zfp133-ps	-2.87469068	0.02377775	Downregulated
Nkd1	-2.87307984	0.02250813	Downregulated
Gm48691	-2.87239347	0.04297276	Downregulated
Igfbp3	-2.87158018	0.03075685	Downregulated
Igdcc3	-2.86825629	0.03668413	Downregulated
Crb2	-2.85843773	0.0388117	Downregulated
Gm45380	-2.84433864	0.03481202	Downregulated
Apod	-2.84388226	0.03079271	Downregulated
Gpr62	-2.84162054	0.03041477	Downregulated
Tmem178b	-2.8383452	0.02939619	Downregulated
Trpm3	-2.83778389	0.02964083	Downregulated
Elfn1	-2.83735743	0.02996384	Downregulated
Kcnj6	-2.83490848	0.02969976	Downregulated
Rims1	-2.83297388	0.0301384	Downregulated
Omg	-2.83207612	0.03040401	Downregulated
Hpca	-2.82907815	0.03081471	Downregulated
Ano3	-2.82717452	0.03115049	Downregulated
Trank1	-2.82411281	0.03138865	Downregulated
Kcnh7	-2.82250027	0.03164963	Downregulated
P4ha2	-2.8219333	0.01183763	Downregulated
Tmem132c	-2.82162325	0.03234248	Downregulated
Elovl4	-2.82116061	0.01590926	Downregulated
Pcdhb13	-2.81755832	0.03524616	Downregulated
Pcdh8	-2.81666178	0.03261371	Downregulated
Tmem266	-2.81495573	0.03290128	Downregulated
Lca5l	-2.81331752	0.04351602	Downregulated
Slc35f4	-2.81219936	0.03337152	Downregulated
C2cd4c	-2.8100358	0.03374553	Downregulated
Wnt3	-2.80854762	0.03486542	Downregulated
Gm43517	-2.80455685	0.03544298	Downregulated
Fibcd1	-2.80407578	0.03533628	Downregulated
Sema5b	-2.80353552	0.03565176	Downregulated

Colgalt2	-2.80290397	0.03501037	Downregulated
Fkbp10	-2.79958433	0.03357217	Downregulated
Agbl4	-2.7904994	0.02796045	Downregulated
Snx24	-2.78746427	0.0293521	Downregulated
Rasgrf1	-2.78744136	0.02752611	Downregulated
Ppfia3	-2.78255993	0.01888787	Downregulated
Mtmr7	-2.78062569	0.01931318	Downregulated
Cabyr	-2.77960614	0.04309622	Downregulated
Rasgef1c	-2.77586964	0.02991785	Downregulated
Vasn	-2.7716671	0.03158458	Downregulated
Adamts18	-2.76671557	0.04224651	Downregulated
Mgat4c	-2.76373161	0.03080816	Downregulated
Musk	-2.75963066	0.04673261	Downregulated
Col1a1	-2.74436373	0.00095149	Downregulated
Paqr5	-2.73649879	0.02900161	Downregulated
Zfp57	-2.72516793	0.02509915	Downregulated
Gm47588	-2.71676664	0.04685665	Downregulated
Tfap2a	-2.70278352	0.0497301	Downregulated
Tubg2	-2.70243374	0.02515613	Downregulated
Grin1	-2.69371876	0.02587595	Downregulated
Gm10800	-2.68838831	0.00782484	Downregulated
Chrd	-2.68107727	0.0466079	Downregulated
Ccdc85a	-2.67852253	0.03709499	Downregulated
Fam189a1	-2.67465564	0.04662816	Downregulated
Fxyd7	-2.67235413	0.047604	Downregulated
Cobl	-2.67030213	0.04668356	Downregulated
Ajm1	-2.670247	0.03837018	Downregulated
Lrp1b	-2.66882836	0.03856224	Downregulated
Csrnp3	-2.66843687	0.04665682	Downregulated
Syt7	-2.6682486	0.04669836	Downregulated
Sox8	-2.66822226	0.04705609	Downregulated
Faim2	-2.66718589	0.03890468	Downregulated
Map6d1	-2.66686018	0.04706704	Downregulated
9330159F19Rik	-2.66646651	0.04709398	Downregulated
Mir100hg	-2.66634651	0.03948703	Downregulated
Pcdhgb1	-2.66624947	0.04780447	Downregulated
Phkg1	-2.6661671	0.04825559	Downregulated
Sh3gl2	-2.66531805	0.04735059	Downregulated
Tmem132a	-2.66367287	0.03057103	Downregulated
Lrrc7	-2.66353839	0.04775125	Downregulated
Cux2	-2.66273068	0.03960936	Downregulated
Tnfaip6	-2.66158091	0.04899922	Downregulated
Sez6l	-2.6583387	0.04911238	Downregulated
Dbx2	-2.65685401	0.04928797	Downregulated
Tenm3	-2.65589832	0.04951151	Downregulated
Gm15853	-2.64653764	0.04443046	Downregulated

Gm10718	-2.63526766	0.00971153	Downregulated
Lrrc24	-2.62062702	0.03193447	Downregulated
Lamc2	-2.61398371	0.01181219	Downregulated
Slc6a7	-2.60123425	0.02213398	Downregulated
Ak4	-2.56119405	0.02319289	Downregulated
Spsb4	-2.55027497	0.04107161	Downregulated
Aplp1	-2.54407139	0.00484485	Downregulated
Fat4	-2.53955626	0.00505673	Downregulated
Gm44067	-2.52598833	0.02126859	Downregulated
Rab15	-2.52597791	0.04106934	Downregulated
2810414N06Rik	-2.51443306	0.0411746	Downregulated
Myrip	-2.49782669	0.0277602	Downregulated
Gm10722	-2.49703027	0.0452163	Downregulated
Srgap1	-2.49688684	0.02856186	Downregulated
Wnk2	-2.4924464	0.02874812	Downregulated
Gm13205	-2.48796396	0.0389429	Downregulated
Rgs5	-2.4543269	0.0221256	Downregulated
Grik4	-2.449662	0.03378808	Downregulated
Robo4	-2.43970828	0.03635086	Downregulated
Zfp366	-2.41917106	0.02189554	Downregulated
Zfp618	-2.38480519	0.04428622	Downregulated
Cpne5	-2.36808801	0.02197162	Downregulated
mt-Co3	-2.2960907	0.04610717	Downregulated
Gm48870	-2.29332799	0.0065252	Downregulated
Kazn	-2.292521	0.01614343	Downregulated
Srcin1	-2.27806243	0.03033809	Downregulated
Galnt15	-2.27374816	0.03240613	Downregulated
Gli3	-2.26429806	0.03409293	Downregulated
Slc6a8	-2.22530774	0.0361666	Downregulated
Pxdn	-2.2161888	0.03716841	Downregulated
Lama4	-2.20847016	0.0277304	Downregulated
Snord13	-2.2036993	0.02030969	Downregulated
Col1a2	-2.14036292	0.00993738	Downregulated
Htr1b	-2.11656407	0.00183818	Downregulated
Angptl6	-2.07716595	0.02401609	Downregulated
Sncaip	-2.01238276	0.03174553	Downregulated
Zfp365	-1.96353351	0.01079829	Downregulated
Nuak1	-1.94739005	0.0023963	Downregulated
Tyro3	-1.9389291	0.00141161	Downregulated
Gm48632	-1.9116763	0.04922421	Downregulated
Acsbg1	-1.86266139	4.02E-05	Downregulated
Tshz3	-1.83784374	0.00366935	Downregulated
Gm2415	-1.76038643	0.03158659	Downregulated
Sox12	-1.73752751	0.04452413	Downregulated
Col4a2	-1.73122088	0.04630402	Downregulated
Nudt22	-1.70171385	0.04145837	Downregulated

Sorbs2	-1.69554468	0.01675749	Downregulated
Amt	-1.68576132	0.03374069	Downregulated
Thtpa	-1.66009381	0.02674399	Downregulated
Fndc10	-1.65794843	0.04354104	Downregulated
Ikzf4	-1.64510134	0.02908782	Downregulated
Gstm4	-1.6400397	0.04680749	Downregulated
Cdh22	-1.63746543	0.01958578	Downregulated
Commd9	-1.61580647	0.00089757	Downregulated
Igkv4-59	-1.60207827	0.0414786	Downregulated
Zfp593	-1.59623121	0.00383938	Downregulated
Arl4d	-1.57983654	0.02676256	Downregulated
Poll	-1.57594639	0.00316959	Downregulated
Vpreb3	-1.49331146	0.02913918	Downregulated
Fbxl15	-1.46567063	0.04398074	Downregulated
Dcxr	-1.43121455	0.02907862	Downregulated
Zswim1	-1.40448003	0.04227455	Downregulated
Zdhhc2	-1.3536593	0.0321474	Downregulated
Pald1	-1.32647249	0.04833169	Downregulated
Ddr1	-1.27992462	0.04189959	Downregulated
Suclg1	-1.16152062	0.0124494	Downregulated
Dtx1	-1.14292009	0.04681274	Downregulated
Tmtc3	1.19725857	0.04203167	Upregulated
Zc3h8	1.24169994	0.04754145	Upregulated
2210408I21Rik	1.30287691	0.02869258	Upregulated
B230369F24Rik	1.38748864	0.03395559	Upregulated
Poglut2	1.41603612	0.03728407	Upregulated
Snhg3	1.5394887	0.00699408	Upregulated
Sh3bp1	1.56382952	0.04726227	Upregulated
Myb	1.60928585	0.01510665	Upregulated
Gspt2	1.61098325	0.01828823	Upregulated
Lrrc69	1.62654569	0.04061716	Upregulated
H3c8	1.68007565	0.03999648	Upregulated
Lrrc27	1.70818111	0.04049097	Upregulated
Mcrip2	1.76992404	0.00842147	Upregulated
Stpg4	1.77577012	0.02548214	Upregulated
Gm37463	1.77703811	0.03757609	Upregulated
Gm50431	1.8130707	0.03651817	Upregulated
Arpp21	1.82580414	0.01363483	Upregulated
Coq7	1.85739385	0.00666146	Upregulated
5033421B08Rik	1.89192098	0.02167899	Upregulated
Nt5dc2	1.90338247	0.03879584	Upregulated
Rpl17-ps5	1.909003	0.03368424	Upregulated
Sdc2	1.9132472	0.01853382	Upregulated
Copz2	1.93252947	0.04533178	Upregulated
H2bc12	1.95521507	0.01901571	Upregulated
2010310C07Rik	1.95993563	0.02286772	Upregulated

Plin4	1.98216455	0.036988	Upregulated
Ackr3	1.98845586	0.04496919	Upregulated
Gm14399	1.99364695	0.0273381	Upregulated
Car13	2.01575548	0.04886988	Upregulated
Lama3	2.0200142	0.02891092	Upregulated
Sall2	2.03176403	0.01394144	Upregulated
Alms1-ps1	2.03583984	0.04150535	Upregulated
Gm24727	2.03826944	0.00859034	Upregulated
H1f1	2.0470961	0.01151282	Upregulated
Cfh	2.05112275	0.00761291	Upregulated
Spats2l	2.0652573	0.04469828	Upregulated
Gm4258	2.08476532	0.03755108	Upregulated
P3h3	2.11444068	0.01535387	Upregulated
Morc4	2.13699636	0.03839783	Upregulated
Smoc1	2.15764046	0.02258355	Upregulated
D130019J16Rik	2.16847621	0.03448681	Upregulated
Tmem141	2.28906364	0.04895633	Upregulated
Car8	2.29473168	0.00752395	Upregulated
Slc16a2	2.3033124	0.03052754	Upregulated
Smo	2.30765822	0.02201448	Upregulated
Spp1	2.33276476	0.04468049	Upregulated
Gm45027	2.34247257	0.02264425	Upregulated
Cdo1	2.35538323	0.0004097	Upregulated
Gm14093	2.3634911	0.02189596	Upregulated
Ephx2	2.36415314	0.04116413	Upregulated
Notch3	2.38526532	0.03922501	Upregulated
Gm37524	2.41479305	0.01046397	Upregulated
Cyp1b1	2.42769075	0.03624171	Upregulated
Vcam1	2.43132236	0.03573892	Upregulated
Tmem169	2.4326608	0.04405614	Upregulated
Gm6501	2.46374556	0.03488131	Upregulated
Rgs22	2.48359886	0.01576071	Upregulated
Gm14539	2.48614426	0.03207256	Upregulated
Lyz1	2.48831491	0.02940298	Upregulated
Gm44625	2.51482139	0.04658903	Upregulated
Gm48986	2.54466042	0.03117764	Upregulated
Gm20518	2.5691278	0.03694259	Upregulated
Pcdhga8	2.57121897	0.04945163	Upregulated
1700088E04Rik	2.58640824	0.0359642	Upregulated
Gm37534	2.58799505	0.0103339	Upregulated
H2ac12	2.59238991	0.01541791	Upregulated
Gpd1	2.61898451	0.0006098	Upregulated
Ltf	2.62591694	0.03817008	Upregulated
Gm44175	2.63388535	0.02803241	Upregulated
Gm37250	2.64516613	0.04097365	Upregulated
Dpt	2.69505282	0.02362008	Upregulated

Gpx3	2.72061576	0.01001656	Upregulated
Pcdh19	2.72610792	0.03063261	Upregulated
B3galt2	2.72627244	0.03104048	Upregulated
A130050O07Rik	2.7379515	0.01558758	Upregulated
Gm15663	2.74911116	0.03044937	Upregulated
ENSMUSG00002075075	2.7763802	0.02753864	Upregulated
Hmgcs2	2.78239421	0.02199122	Upregulated
Atp5l2-ps	2.78485998	0.03966938	Upregulated
Gm12669	2.80632544	0.04738441	Upregulated
Gm9042	2.81518363	0.04384809	Upregulated
Cacng4	2.82953819	0.0231673	Upregulated
F3	2.84063803	0.02305229	Upregulated
Atp1a2	2.84696821	0.01508794	Upregulated
Spag6l	2.86395874	0.02148384	Upregulated
Sfrp1	2.87172318	0.00994848	Upregulated
Gm16008	2.90831153	0.04354075	Upregulated
Gm21984	2.93303627	0.02255898	Upregulated
Islr	2.93383073	0.0165122	Upregulated
Gm15743	2.95290942	0.04611092	Upregulated
C1qa	2.95410757	0.02844747	Upregulated
Cfap300	2.97718567	0.02957697	Upregulated
Vsnl1	2.97916498	0.01440032	Upregulated
Gm14681	2.98409352	0.02767835	Upregulated
Prkd1	2.98819718	0.02801131	Upregulated
Gm44210	2.99325165	0.03091064	Upregulated
Rgs7	2.99429383	0.02679612	Upregulated
S100b	3.00611684	0.02743761	Upregulated
Gm14296	3.00735361	0.02984378	Upregulated
Cth	3.01598389	0.0037691	Upregulated
Zfp449	3.0367252	0.00948318	Upregulated
Gxylt2	3.03731968	0.02708458	Upregulated
Igfbp6	3.03771588	0.00154665	Upregulated
Ecrg4	3.03981853	0.02994419	Upregulated
C1qc	3.05048328	0.0124102	Upregulated
Rbm24	3.07156846	0.0475123	Upregulated
Col9a2	3.12676644	0.0198379	Upregulated
Apol10b	3.12796688	0.00795807	Upregulated
Cpxm1	3.15080652	0.01430796	Upregulated
Bche	3.15565212	0.01372409	Upregulated
Gm42729	3.17162396	0.04824938	Upregulated
Fabp3	3.19318835	0.01144757	Upregulated
Cstad	3.20237511	0.01560586	Upregulated
Nxf3	3.2057259	0.04096316	Upregulated
Tas2r126	3.22171828	0.03602035	Upregulated
Clec1a	3.24012221	0.01333537	Upregulated
Edn1	3.24717984	0.03888363	Upregulated

Tox3	3.27279824	0.00858204	Upregulated
Ptgis	3.27353574	0.0392978	Upregulated
Fbl-ps2	3.27915668	0.02137546	Upregulated
Gm26571	3.28756359	0.03276653	Upregulated
ENSMUSG00002075203	3.29892328	0.0240581	Upregulated
Ccdc153	3.32395096	0.01423479	Upregulated
Cyp4b1	3.34206091	0.03742145	Upregulated
4930466K18Rik	3.3586129	0.00574413	Upregulated
7SK	3.36603237	0.04136509	Upregulated
Chia1	3.37641504	0.01483017	Upregulated
Krt8	3.37920567	0.0171781	Upregulated
Gm37632	3.40211978	0.0411767	Upregulated
Lrrc3b	3.41080774	0.00724622	Upregulated
Gm38318	3.41425675	0.02150838	Upregulated
Ildr2	3.4159572	0.00489063	Upregulated
Gm44549	3.41737394	0.02089663	Upregulated
Gm41077	3.42243382	0.04227408	Upregulated
Itgbl1	3.43802293	0.02796679	Upregulated
Ppp1r3c	3.43958743	0.00496403	Upregulated
Wnt2	3.44717587	0.04397306	Upregulated
Gm43186	3.46107255	0.00662571	Upregulated
Gm45540	3.46566522	0.03795408	Upregulated
C4b	3.49319602	0.00937034	Upregulated
Gm44068	3.50433926	0.01687594	Upregulated
Tdrd5	3.5084721	0.03597864	Upregulated
Snord11	3.51543133	0.00928671	Upregulated
Ccdc3	3.55465776	0.00209527	Upregulated
Gm49784	3.56357902	0.0098301	Upregulated
Camp	3.56451689	0.00905675	Upregulated
Ogn	3.56504927	0.00452332	Upregulated
F830208F22Rik	3.56606836	0.0297587	Upregulated
Ednrb	3.58961832	0.01749098	Upregulated
Gm16897	3.61588165	0.01840761	Upregulated
Acta1	3.66544482	0.00150093	Upregulated
2410004P03Rik	3.67812739	0.00496874	Upregulated
Rorc	3.68021463	0.00610101	Upregulated
Aqp11	3.68027661	0.00162136	Upregulated
Gm10702	3.76212702	0.01310364	Upregulated
Cbr2	3.76888057	0.00851381	Upregulated
Plin5	3.78090562	0.01230415	Upregulated
ENSMUSG00002076731	3.78534854	0.01284385	Upregulated
Retnla	3.8009208	0.04995363	Upregulated
Gm47373	3.84248849	0.03175293	Upregulated
Gm13625	3.86546047	0.02851525	Upregulated
Gm37548	3.91575066	0.03519108	Upregulated
H19	3.99156555	0.0185644	Upregulated

Pck1	4.02306928	0.00244378	Upregulated
Foxa1	4.02883165	0.04010943	Upregulated
Ankef1	4.05272024	0.00079796	Upregulated
Gsta3	4.06431238	0.00248776	Upregulated
Pdk4	4.08127561	0.00104853	Upregulated
Rbp4	4.09179162	0.00084567	Upregulated
Msln	4.20055516	0.01593297	Upregulated
Cidea	4.27299354	0.0032347	Upregulated
Gm43787	4.32909532	0.00036586	Upregulated
C1qb	4.36225337	0.00010981	Upregulated
Cyp2f2	4.39486737	0.01972194	Upregulated
Sec14l3	4.48240402	0.00232963	Upregulated
BB031773	4.51895385	0.01670322	Upregulated
C1s1	4.59899677	0.00278752	Upregulated
Myl1	4.61484147	0.0048259	Upregulated
Cidec	4.69124795	0.037581	Upregulated
Dntt	4.70125584	0.04180464	Upregulated
Plin1	4.75827206	0.00148829	Upregulated
Gm26930	4.81227338	0.00065882	Upregulated
Thrsp	4.84735135	0.00022969	Upregulated
7SK	4.87829218	0.00568799	Upregulated
Myh4	5.02712302	0.00105939	Upregulated
Cyp2e1	5.09524117	0.01779625	Upregulated
Prg4	5.10140494	0.00017457	Upregulated
Car3	5.11902798	3.10E-07	Upregulated
Bpifb1	5.5069735	0.02909013	Upregulated
Arsi	5.55093479	0.00057032	Upregulated
Ces1d	5.76266691	0.00013452	Upregulated
H2-Q2	6.17731034	0.00018326	Upregulated
Cyp2a5	6.30298748	0.00168417	Upregulated
Ucp1	6.80669401	0.00012484	Upregulated
Scgb3a1	7.20743917	0.00432585	Upregulated
Rag1	7.46341832	1.88E-08	Upregulated
Bpifa1	7.86830248	0.00257126	Upregulated
Scgb1a1	8.02938572	0.00663262	Upregulated

Table of Contents Top of Current Table

Supplemental Table 10. List of all overlap of significant (P < 0.05) differentially expressed genes in whole blood of B6129 CBD (week 0 to 8) and B6129 vehicle (week 0 to 8) animals. &Commonly downregulated in 3xTg-AD (vehicle only) and B6129 (vehicle & CBD); @Commonly downregulated in 3xTg-AD (CBD only) and B6129 (vehicle & CBD); #Commonly downregulated in 3xTg-AD (vehicle & CBD) but upregulated in B6129 (vehicle only); *Commonly upregulated in 3xTg-AD (vehicle & CBD) and B6129 groups (vehicle & CBD); **Commonly upregulated in 3xTg-AD (vehicle only) and B6129 (vehicle & CBD); ***Commonly upregulated in 3xTg-AD (CBD only) and B6129 (vehicle & CBD); ‡Commonly upregulated in 3xTg-AD (vehicle only) and B6129 groups (vehicle & CBD); ^Commonly upregulated in 3xTg-AD (vehicle only) and B6129 (CBD only) but downregulated in B6129 (vehicle only); +Commonly upregulated in 3xTg-AD (vehicle only) but downregulated in B6129 (vehicle & CBD); ++Commonly upregulated in 3xTg-AD (CBD only) but downregulated in B6129 (vehicle & CBD)

Common Genes Dowregulated	Common Genes Upregulated	Common but Opposite: Downregulated in B6129 CBD & Upregulated in B6129 Vehicle	Common but Opposite: Upregulated in B6129 CBD & Downregulated in B6129 Vehicle
Abcc8	Ackr3**	Gm43868; lncRNA	Prkd1
Adamts18	C1qa*	Tnfaip6	Rbp4^
Adgrb3	C1qb*		S100b
C130074G19Rik&	C1qc*		
Cntnap3	C4b*		
Col24a1	Car3*		
Col4a2	Cbr2‡		
Csrnp3	Ccdc153		
Cux2	Cfh		
Dgkb	Ednrb*		
Dnah14	Fabp3‡		
Dpp10	Gpd1*		
Esyt3	Igfbp6***		
Faim2	Islr		
Fam110d&	Ogn		
Fibcd1	Pdk4*		
Frmd3	Prg4*		
Gm10717	Ptgis*		
Gm10718	Rag1		

Gm10722	Retnla*			
Gm10800	Ucp1*			
Gm10801				
Gpld1				
Igdcc3				
Kcnj6				
Lhx6				
Lrp1b				
Mapk10				
Mgat4c				
Mir100hg				
mt-Co3				
Myrip				
Myt1				
Myt1l[+]				
Nkain3				
Nkd1				
Nrxn3				
Pcdh17				
Phkg1				
Rab15[@]				
Rasgef1c				
Robo4[@]				
Sgsm1				
Slc35f4				
Snord13				
Sorbs2[++]				
Srgap1[+]				
Tenm3				

Tmem178b			
Wnk2			
Zfp133-ps			
Zfp423			
Zfp618			
Zfp786			

Supplemental Table 11. List of all overlaps of significant (P < 0.05) differentially expressed genes in whole blood of 3xTg-AD CBD (week 0 to 8) and B6129 CBD (week 0 to 8) animals. [%]Commonly downregulated in 3xTg-AD (vehicle & CBD) and B6129 (CBD only); [@]Commonly downregulated in in 3xTg-AD (CBD only) and B6129 (vehicle & CBD); [##]Commonly downregulated in 3xTg-AD (vehicle and CBD) but upregulated in B6129 (CBD only); *Commonly upregulated in 3xTg-AD (vehicle & CBD) and B6129 groups (vehicle & CBD); **Commonly upregulated in 3xTg-AD (vehicle only) and B6129 (vehicle & CBD); ***Commonly upregulated in 3xTg-AD (CBD only) and B6129 (vehicle & CBD); [††]Commonly upregulated in 3xTg-AD (vehicle & CBD) and B6129 (CBD only) groups; [++]Commonly upregulated in 3xTg-AD (CBD only) but downregulated in B6129 (vehicle & CBD); [+++]Commonly upregulated in 3xTg-AD (CBD only) and B6129 (CBD only) but downregulated in 3xTg-AD (vehicle only); [$$]Commonly upregulated in the 3xTg-AD animal groups (vehicle and CBD) but downregulated in the B6129 (CBD only)

**Common Genes Dowregulated**	**Common Genes Upregulated**	**Common but Opposite: Downregulated in 3xTg-AD CBD & Upregulated in B6129 CBD**	**Common but Opposite: Upregulated in 3xTg-AD CBD & Downregulated in B6129 CBD**
Fuom[%]	7SK	2010310C07Rik; lncRNA	Adcyap1r1
Galnt15[%]	Ackr3**	ENSMUSG00002075075 or Gm56228; miRNA	Fbxl15
Grm1	Acta1	Tdrd5[##]	Fgd1
Kcnj12	Aqp11		Fhod3
Paqr5	Atp1a2		Gm27252; lncRNA
Rab15[@]	Bpifa1		Htr1b
Robo4[@]	C1qa*		L3mbtl1
Susd4[%]	C1qb*		Mpdz
	C1qc*		Pex11g
	C1s1		Proca1
	C4b*		Pth1r[$$]
	Car3*		Slc26a1
	Cdo1		Sorbs2[++]
	Ces1d[††]		Tmem132a
	Cidea[††]		Tmem132e
	Cidec[††]		
	Clec1a		

	Copz2		
	Cyp1b1		
	Cyp2a5		
	Cyp2f2††		
	Dpt††		
	Ednrb*		
	F3		
	Gm12669		
	Gpd1*		
	Gpx3		
	H19		
	Igfbp6***		
	Lyz1		
	Msln		
	Myh4††		
	Myl1		
	Pck1††		
	Pdk4*		
	Peg3+++		
	Plin1††		
	Plin5		
	Ppp1r3c††		
	Prg4*		
	Ptgis*		
	Retnla*		
	Scgb3a1		
	Sec14l3††		
	Sfrp1††		
	Thrsp††		

	Ucp1*		

Supplemental Table 12. List of all differentially expressed genes (P<0.05) in whole blood of wild-type B6129 vehicle (week 0 to 8) that were reversed in expression in wild-type B6129 CBD (week 0 to 8) animals in comparison. *P<0.05, significant expression in the opposite direction in wild-type B6129 CBD

DEG (P<0.05): B6129	log2FoldChange: B6129	log2FoldChange: B6129 CBD	P-value: B6129 CBD
Oprm1	-5.461105358	0.861042415	0.819399425
Mir6399	-5.096844701	2.074543504	0.335410276
3425401B19Rik	-4.98214822	0.978540621	0.783605633
Gm16372	-4.952393223	1.169960914	0.725349447
Akap6	-4.924306888	0.362314396	1
Sntg1	-4.923299155	0.365142124	1
Mycbpap	-4.882904577	0.223086815	1
Gm4813	-4.865814784	1.806878409	0.420221049
Gm2106	-4.865232434	0	1
Alox12e	-4.847309557	0.631335469	0.968545064
A830009L08Rik	-4.804088683	0.626677797	0.969537465
Cnmd	-4.767627288	0	1
Cbx3-ps6	-4.722590199	2.558312972	0.168451365
D630023F18Rik	-4.696595823	0.932488882	0.787009355
Nav3	-4.682328754	0.044501459	1
Gabrg3	-4.681151108	0	1
Gm13446	-4.663450885	0	1
Tpo	-4.604547678	0.460446657	1
Ccl27a	-4.588106381	0.538827121	1
Kcnq2	-4.572222937	1.579343251	0.414415005
Gm44144	-4.568746913	2.129571745	0.116618256
Gm31107	-4.550208116	0.286844601	1
Shc3	-4.54475567	1.833412458	0.284974404
Negr1	-4.544723293	0.358994919	1
Col11a1	-4.527649144	1.444521969	0.30196505
Gm49192	-4.518936364	0.687691198	0.922445962
Otx1	-4.51867069	0.373025697	1
Gm42597	-4.490946951	0	1
Gm26756	-4.482305341	0.360229584	1
ENSMUSG00002076530	-4.427794192	0.811528948	0.808907642
Syt4	-4.393271443	0.950103248	0.777575015
Slc17a7	-4.39271933	1.425397157	0.491818778
Gm48743	-4.372400939	0	1
Sostdc1	-4.366289522	1.471077058	0.511268046
Avp	-4.338074954	0	1
Cilp2	-4.326378105	0.086007637	1
Ttc6	-4.319226751	0.178640347	1
Egflam	-4.31491737	0.320303514	1

D330050G23Rik	-4.237261231	1.426596135	0.506974132
Igsf11	-4.223375974	0	1
Lrfn5	-4.223156676	0	1
Gm35040	-4.221875088	1.504554922	0.467990964
Kndc1	-4.22163886	0.379420062	1
Gm26777	-4.214168075	0	1
2900027M19Rik	-4.213757926	0	1
Etv4	-4.21355592	0.831104464	0.77718373
Kcns3	-4.21031224	0	1
Rbp4*	-4.199403011	4.091791624	0.000845669
Gm45470	-4.184789995	0	1
Rpl29-ps2	-4.168495009	0	1
Rab3c	-4.168365165	0.147677808	1
Lypd1	-4.159026933	2.531183313	0.080217257
Cfap65	-4.074317496	1.65146257	0.25304772
Tmco5	-4.052030107	0.687231268	0.921424275
Gm29340	-4.047440174	0.053413943	1
Vmn2r53	-4.042854391	0.529045657	1
Cntnap5b	-4.029779469	1.55182625	0.266088692
Tmem200a	-4.028515268	0.927923138	0.786933142
Ptprn2	-4.028029922	0.349039329	1
S100b*	-4.027448898	3.006116841	0.027437612
Gm5067	-4.016237182	0.933303136	0.787034006
Cda	-4.013147309	0.950103155	0.789299212
Gm23995	-3.993208288	1.908263299	0.314742322
Wif1	-3.991812966	2.051105064	0.262706224
Gm48996	-3.985696281	1.450113424	0.501035776
Cd63-ps	-3.983392105	0.374763174	1
AU022754	-3.982897583	0.396698569	1
Gm45496	-3.978673205	0.930953593	0.798244352
Trp53cor1	-3.975802058	0.409456638	1
Gm5454	-3.961698414	0	1
Mir7052	-3.932459922	1.893368295	0.313689393
A330094K24Rik	-3.870008591	2.320097869	0.130756312
Zfp973	-3.866815167	0	1
Gm19196	-3.861639674	0.902920247	0.75398398
Gm44907	-3.85950003	0.362096275	1
A730098A19Rik	-3.839731168	0.813392301	0.820318724
Fsip1	-3.831499272	1.044057199	0.656028774
Dok5	-3.810041073	0	1
Rem2	-3.809897587	1.4980173	0.473943499
Grid1	-3.809578661	0.858173287	0.820473012
Gm37805	-3.809009081	0	1
Ajap1	-3.808479795	0.360628149	1

Tmem178	-3.80840628	0	1
Pcsk2	-3.807157027	0	1
Mro	-3.806577165	0.774326555	0.86198884
Cdh15	-3.80121813	2.528120269	0.063152097
Gm11855	-3.799343951	0	1
Gm43789	-3.798765891	0	1
Gm37928	-3.798757996	0	1
Gm14413	-3.797160651	0.579413439	1
Gm43175	-3.794078855	0	1
Pla2g4e	-3.793564497	0.950103212	0.782282846
Gm44021	-3.792897297	0.878472656	0.81242778
Gm37818	-3.784766946	0.945971331	0.722944684
Slc9a4	-3.783302053	0.401476952	1
Tdgf1	-3.776832505	2.198899323	0.217625621
Gm45737	-3.772234337	1.149946046	0.649932182
Erich2	-3.758102308	1.613534188	0.378152424
En1	-3.75759657	0.498048765	1
4930507D05Rik	-3.75619531	0.947518148	0.791712983
Gm49077	-3.755445185	1.689181785	0.322865614
Mab21l2	-3.691213894	0	1
2310040G24Rik	-3.587926753	2.464875812	0.09802732
Kcnmb4os2	-3.587097669	2.427530238	0.130432054
Kcnma1	-3.583340045	0.36275424	1
Aifm3	-3.580315001	2.443556764	0.067326058
Gm5628	-3.57473549	1.532742137	0.415662671
Foxb1	-3.546478731	1.005070848	0.642042503
St8sia3	-3.546249302	0.373025714	1
Slitrk3	-3.54594186	0.373025713	1
Phyhipl	-3.545822931	0.925018886	0.788201237
Mlip	-3.545806678	0.950103243	0.778302833
Prkg2	-3.545672278	0.359996449	1
Galnt18	-3.545620739	0.045697644	1
Pcdhac2	-3.54556722	1.07939638	0.634117658
Eef1a2	-3.545452688	0.950103246	0.777799004
Actl6b	-3.545296341	0.369868728	1
Slc7a10	-3.54504743	0.860699242	0.819161787
Atp13a5	-3.54437065	1.505328937	0.468629351
Chrna4	-3.541900634	0.350172689	1
Gm38260	-3.54041967	0	1
Nsun7	-3.540388067	0	1
Gm48542	-3.540341445	0	1
Gm37345	-3.538435366	0	1
Gm49411	-3.537040427	0.950103224	0.780712765
Gm43953	-3.537033511	0.373025704	1

1700086D15Rik	-3.536268542	0	1
Gm42443	-3.536167273	1.485670588	0.490432163
Fibin	-3.532944586	0.3730257	1
Gm45200	-3.531838576	0	1
Gm37393	-3.531298431	0.95010315	0.789870214
Slc13a4	-3.528511655	0.398855207	1
Gm45257	-3.528288456	2.469805107	0.117569036
Gm23787	-3.517567259	0	1
Pax7	-3.512819869	1.983749913	0.263061588
Mir3473g	-3.495091839	0.95318036	0.738304934
Fstl4	-3.429731818	0.381511846	1
Vwc2	-3.426003269	2.391593558	0.100294824
Ano4	-3.261712521	1.854371836	0.234484591
Gm3739	-3.251398987	0.639891917	0.956287247
Esrrb	-3.250055483	0	1
Gm20544	-3.190042996	1.058749871	0.595880036
B4galt2	-3.18490995	0.595804906	0.989329809
Gm22488	-3.178930879	1.530613309	0.21111373
A4galt	-3.134142991	0.070975349	1
Abi3bp	-3.130809693	0.274125583	1
Pth2r	-3.097852777	1.456831394	0.489923883
Gm42568	-3.080306346	0.233825828	1
H1f10	-2.972852488	2.426724767	0.09338221
Prkd1*	-2.955490911	2.988197185	0.028011305
Sgtb	-2.953681171	1.876436674	0.062398595
Grik1	-2.949403986	0.373025713	1
Gm43513	-2.89444683	0.437236405	1
Myo5c	-2.869398956	1.754313496	0.069480475
Gm25926	-2.858264467	0.950103111	0.794148071
Gm49871	-2.791342344	0.811964712	0.770157719
Ndrg4	-2.687715117	0.383250482	1
Fbxl13	-2.633919624	1.293044478	0.241713466
Gm17017	-2.615650954	0.501587157	1
Il34	-2.5199069	0.804763418	0.675242475
Gm43807	-2.518667925	0.720179014	0.835807452
Slco5a1	-2.489573989	0.299249292	1
Mir6236	-2.327355888	0.724236056	0.83804081
Gm22973	-2.289483441	0.25590439	1
Gm23297	-2.2766164	0.396472979	1
Bnip5	-2.256418193	0.288338039	1
Gm23444	-2.250378212	0.067657957	1
Gm23472	-2.203341977	0.164576264	1
Gm43627	-2.107728321	0.601744639	0.972672155
Nr1i2	-2.020179539	0.477700399	1

Fzd3	-2.019047711	1.621699006	0.091403702
Map9	-1.95453812	0.082364717	1
Unc5c	-1.840861057	0.032512313	1
Elp6	-1.797132033	0.112154736	1
A630072L19Rik	-1.743131214	0.221672746	1
Reep1	-1.723939035	0.774871729	0.64708163
Nsg1	-1.721791252	0.056872137	1
Gm49420	-1.699689014	0.357641223	1
Gm38366	-1.533298128	0.219435404	1
Senp8	1.70307933	-0.094704621	1
Rundc3a	1.807108289	-0.523306138	1
Hspa12a	2.337687069	-0.149886802	1
Gm38235	2.416076036	-0.474572548	1
Slc7a2	2.712276377	-0.733682339	0.894237387
Gm25821	2.791979812	-0.221616074	1
Cplane2	2.864279417	-0.294226039	1
E230020D15Rik	2.895039865	-0.293776453	1
Osbpl6	2.939901695	-0.0106346	1
Gm44967	3.016063234	-0.130841592	1
Htra1	3.023117068	-0.215439876	1
Bco2	3.222394769	-0.755398995	0.83621432
Gm13689	3.284240438	-1.672274646	0.367768777
Gm48623	3.284473242	-0.260102743	1
Ednra	3.288111174	-1.32019603	0.383221977
1700001L19Rik	3.291183791	-1.259976878	0.584600763
Gm13270	3.311475068	-0.070924637	1
Gm43868*	3.463823206	-3.46032639	0.010766957
Tnfaip6*	3.476157632	-2.661580913	0.04899922
Plppr2	3.479781049	-0.38394854	1
Pex11a	3.482769776	-0.008770797	1
Gm17893	3.492479278	-0.699339112	0.887067668
AV099323	3.524894389	-0.324280098	1
Adamtsl5	3.549656329	-0.342719256	1
Gjc1	3.640251673	-1.548228964	0.351001983
Zfp37	3.651045797	-1.705973164	0.262925407
Gm43800	3.659114813	-0.535610057	1
Gm48582	3.674181341	-0.282590644	1
Myl6b	3.684389031	-1.070797102	0.330941971
Ocln	3.688896639	-0.976159544	0.765713134
Hsd17b1	3.71075796	-1.065901524	0.565358466
Wfikkn1	3.757889582	-0.422140227	1
Nhlrc1	3.788901106	-0.093241405	1
Fzd1	3.789000194	-0.915446323	0.639904245
Gm9929	3.796835899	0	1

2310002F09Rik	3.805844018	-1.393794805	0.456495885
ENSMUSG00002075672	3.82477807	-1.654803242	0.252040193
Gm48673	3.843992817	-0.84282947	0.680668264
Gm43365	3.848289597	-0.026895517	1
Gm1604a	3.924231771	-0.614126656	0.976342936
Grem2	3.940146657	-1.663554448	0.356448545
Gm20125	3.956326551	-1.372432423	0.465118448
Prss57	4.058342674	-0.954745083	0.777097113
Gdpd2	4.074065136	-0.849072481	0.793867124
Smtnl2	4.085539925	-0.779147301	0.849256705
Gm13786	4.101137649	-1.137010596	0.509127333
Nox1	4.132743889	-2.254037084	0.140201846
Adamts2	4.194555882	-0.772914238	0.784043088
Gm42780	4.201824051	-0.077271395	1
Plaat5	4.237240575	-1.390000981	0.483418224
Gm12708	4.294807514	-0.349767197	1
Tcaf1	4.355127415	-1.052124963	0.700294067
Hspb8	4.368708402	-0.408926846	1
4833408A19Rik	4.399739409	-0.352413119	1
9630028I04Rik	4.621600996	-0.640618988	0.963714857
Gm9830	4.723605222	-0.451569512	1
Gm8850	4.741453304	-0.548065963	1
Gm47326	5.137859244	-2.77383189	0.178328393
Bcam	6.148880969	-1.407755766	0.375035085

Table of Contents Top of Current Table

Supplemental Table 13. List of all differentially expressed genes (P<0.05) in whole blood of 3xTg-AD CBD (week 0 to 8) that were reversed in expression in wild-type B6129 CBD (week 0 to 8) animals in comparison. *P<0.05, significant expression in the opposite direction in wild-type B6129 CBD

DEG (P<0.05): 3xTg-AD CBD	log2FoldChange: 3xTg-AD CBD	log2FoldChange: B6129 CBD	P-value: B6129 CBD
Tdrd5*	-16.26321405	3.508472105	0.035978635
Gm49376	-4.64582962	0.899574793	0.704563832
Gm34342	-4.393390297	0.919902456	0.852751246
Jazf1	-4.2620144	0.052894811	1
Gm20276	-4.236593311	0	1
Gm13710	-4.228989917	2.813133743	0.131665209
Gm42433	-4.175319922	0.354742179	1
Gm43728	-4.148896205	0.667967123	0.941248987
Gm42975	-4.123860756	2.469694706	0.247721109
Gm42728	-3.998332824	1.425818207	0.291132933
Gm12543	-3.905086719	1.745041031	0.445417269
Kdm4d	-3.899383542	0.530469118	1
C330008A17Rik	-3.898541893	1.083152514	0.736395305
Gm12737	-3.803074924	0.396251058	1
Gm6630	-3.802787641	0.120759376	1
Rasl11b	-3.742452666	0.67260913	0.868478024
Gm12971	-3.679046471	0.986433633	0.622459374
Gm14021	-3.664959888	0	1
Prokr2	-3.533809711	0.196713287	1
ENSMUSG00002074935	-3.471161787	2.555341349	0.112791079
Gm26737	-3.448312879	0	1
Gm43909	-3.431770203	1.073737813	0.577128368
Gm47594	-3.375900992	0.798074445	0.843644741
Gm49616	-3.332891392	0.193477715	1
Mycl	-3.327945899	0.279017693	1
A930035D04Rik	-3.310437036	1.57605742	0.268761242
Gm14373	-3.287489453	1.179561501	0.674297191
Gm23887	-3.275474714	0.105228046	1
5830487J09Rik	-3.246712808	0.984316527	0.595571256
ENSMUSG00002075075*; Gm56228, miRNA	-3.242362932	2.776380203	0.027538644
Sox17	-3.240758214	1.194406775	0.494572156
2810407A14Rik	-3.229697619	1.55816083	0.377647113
Nostrin	-3.199939677	0.068803792	1
Gm48254	-3.198932437	0.698529133	0.910203656
Gm43437	-3.194724342	0.28038961	1
Gm45807	-3.191478818	0.041864619	1
Map2k3os	-3.163123208	0.224820054	1
Gm20732	-3.103104926	1.277774085	0.295840277

Gm37536	-3.079044129	0.683103593	0.901738463
Gm44695	-3.074260275	1.423413519	0.310786172
C030032O16Rik	-3.072290607	2.952287413	0.070645068
5S_rRNA	-3.062553892	0.849189134	0.790087384
Ushbp1	-3.05758952	0.167762168	1
Rgs7bp	-3.056265575	0.029048031	1
Gm44899	-3.048480954	0.090416173	1
Gm17509	-2.93979351	0.530614199	1
Aldob	-2.903768669	0.375335228	1
Tas1r3	-2.86011782	0.28718	1
Gm45203	-2.848838361	0.305186337	1
Fjx1	-2.745819617	0.950103245	0.777984838
Gm43412	-2.706428193	0.085924345	1
Gm44045	-2.673455265	1.692772653	0.083151285
Pwwp2b	-2.639288755	0.251604359	1
8430422M14Rik	-2.614064152	0.482218835	1
Hic2	-2.590382151	0.229463334	1
Slc12a5	-2.562230924	0.180201107	1
Gm37663	-2.556382726	0.220887797	1
Reep1	-2.501898904	0.774871729	0.64708163
Slc16a9	-2.48837585	1.211220567	0.346291104
Gm47324	-2.459061504	0.238052713	1
2010310C07Rik*	-2.442927565	1.959935631	0.022867717
B930036N10Rik	-2.419847222	0.447552668	1
Edn3	-2.377061917	0.481645306	1
Zfyve28	-2.357275608	0.400618315	1
Gm17203	-2.346179799	1.361425585	0.277147922
Gm47121	-2.320309043	0.668725953	0.886830667
6230400D17Rik	-2.318402013	0.806216547	0.66305434
Tuba-rs1	-2.304438189	0.185327819	1
C230037L18Rik	-2.200247999	0.098851379	1
Gm37570	-2.196280073	0.7821392	0.720822731
Cited4	-2.149236683	0.271993877	1
Gm48961	-2.146386244	0.295760674	1
A730081D07Rik	-2.131005251	0.84115487	0.72696405
Gm44888	-2.083701783	0.031922195	1
Gm43254	-2.080474071	0.27075774	1
Gm13357	-1.979898279	0.942609959	0.605093455
Gm40309	-1.771849251	0.214761247	1
Ndnf	-1.726027025	0.212365524	1
Tnfsf13b	-1.587843711	0.920747189	0.42133913
Ptpdc1	-1.54599945	0.182669609	1
BC048403	-1.434022859	0.141367432	1
Armcx1	-1.297865759	0.07382669	1

Phactr2	-1.00190365	0.053863299	1
Commd6	1.046672226	-0.203599605	1
Med28	1.062295875	-0.022462676	1
Cdc34	1.110491007	-0.110059544	1
Atp6v0b	1.124760216	-0.154018709	1
Rps15	1.138112234	-0.606997219	0.931368207
Rpl22l1	1.169537933	-0.758930761	0.554170888
Bola2	1.183034216	-0.328827596	1
Use1	1.192426262	-0.657485465	0.801290689
Rpl32	1.202926182	-0.306934272	1
Coa3	1.213690371	-0.089779491	1
Glrx3	1.218362821	-0.042768467	1
Mrpl41	1.233431528	-0.266871294	1
Mrps14	1.236143206	-0.011399907	1
Lancl1	1.266649606	-0.324743179	1
Pycard	1.271333058	-0.351344338	1
Rpl9	1.281376427	-0.095002271	1
Chrac1	1.283618539	-0.068840605	1
Rps13	1.285779044	-0.346423585	1
Gm23442	1.293532738	-0.362800803	1
Rps21	1.316861277	-0.287792238	1
Psmb4	1.316939357	-0.159479379	1
Rpl5	1.321269174	-0.12633076	1
Rpl35a	1.322000891	-0.15449854	1
Rack1	1.346273588	-0.946304717	0.249000232
Ssr4	1.346518821	-0.090660694	1
Med11	1.349267977	-0.13804881	1
Rps19	1.349887272	-0.543849943	1
Nudt14	1.35069169	-0.44239512	1
Mrps24	1.36427952	-0.330533533	1
Cenpx	1.377740776	-0.200822279	1
Mea1	1.399139982	-0.257067846	1
Ubl4a	1.400655228	-0.071904145	1
Rpsa-ps10	1.426843207	-0.405126311	1
Cyba	1.439513787	-0.166050318	1
Cox16	1.44502316	-0.27096011	1
Itgb1bp1	1.445965956	-0.336538763	1
Tesc	1.454643574	-0.147886578	1
Rpl9-ps6	1.469868035	-0.031283784	1
Rps3	1.474603002	-0.524026285	1
Fam162a	1.482787224	-0.278089696	1
Rps18	1.483020023	-0.107763631	1
Hadh	1.488313703	-0.017014298	1
Gm25117	1.501655916	-0.270269884	1

Sptssa	1.505314223	-0.053091188	1
Rps25	1.512443952	-0.403753017	1
Gm48226	1.524162808	-0.468408986	1
A430005L14Rik	1.524662119	-0.399678778	1
Lsm4	1.539664427	-0.332051139	1
Echs1	1.546002049	-0.273453344	1
Bloc1s1	1.551298502	-0.182919469	1
Pfdn1	1.554254198	-0.550567352	1
Rps27rt	1.559960263	-0.772418841	0.616582805
Pafah1b3	1.563363055	-0.449329127	1
Rpl27	1.596936121	-0.065230414	1
Nsmce1	1.602788851	-0.028159466	1
AI413582	1.614030652	-0.160404084	1
Dctn3	1.625023792	-0.356819015	1
Gm19585	1.640193852	-0.747455663	0.729321442
Bbln	1.646359459	-0.672707597	0.844618025
Snrpd2	1.651834256	-0.303727982	1
Rps15a-ps6	1.66811781	-0.643960855	0.903641548
Mrpl13	1.686149542	-0.018841947	1
1810037I17Rik	1.706521454	-0.057967018	1
Zfp85	1.707502786	-0.196924179	1
Dctpp1	1.717631546	-0.119316393	1
Slirp	1.729028984	-0.580805631	1
Mtln	1.740805662	-0.560105768	1
Gpx4	1.742617187	-0.063001355	1
3110001I22Rik	1.744439973	-0.66954234	0.834722904
Mrpl28	1.763331031	-0.872612026	0.37281845
Rpl36	1.789912376	-0.120849458	1
Snx21	1.810300085	-1.040790093	0.387017097
Fzd1	1.81582892	-0.915446323	0.639904245
Pcbp4	1.849224113	-0.74326016	0.668249479
Tspo	1.865309933	-0.120476049	1
Plac8	1.889824521	-0.04196319	1
Fbxl15*	1.914338062	-1.465670628	0.04398074
Mansc1	1.937131642	-0.424661076	1
Gm15500	1.969746377	-0.100902942	1
Timm10	1.985081886	-0.029827635	1
Rpl10a	1.988024719	-0.141895968	1
Gm8186	2.041676492	-0.300556605	1
4933431K14Rik	2.042353406	-0.569422635	1
Htr1b*	2.051487814	-2.116564066	0.001838178
Tmtc1	2.052352747	-0.034158803	1
Axl	2.057916148	-0.087707461	1
Rab3a	2.07687884	-0.271575024	1

Mrpl57	2.111697953	-0.138830184	1
Pls3	2.12540058	-0.686331151	0.828168997
Hcfc1r1	2.153205619	-0.656785702	0.843453935
Hlf	2.202509201	-0.121661975	1
Gm13611	2.21048142	-0.388406426	1
Acot6	2.212368634	-0.344458267	1
Dennd2a	2.248888747	-1.492420857	0.279580518
Gpx4-ps2	2.249876189	-0.003946095	1
Dctd	2.273050167	-0.020350086	1
Map6	2.314214344	-1.958918759	0.085562997
Rps10-ps1	2.340445031	-0.787729668	0.788128718
Tfpi	2.370236601	-0.941421957	0.447110709
Cxcl12	2.381484739	-0.467975453	1
Cib2	2.394395595	-0.073796739	1
Mapk8ip1	2.428786252	-1.635970719	0.194893214
Gm9531	2.451749299	-0.075162203	1
Btf3-ps1	2.460449174	-1.351646143	0.365443869
Map1b	2.483107995	-0.015892629	1
Parm1	2.526352642	-0.287263145	1
Cstb	2.538617135	-0.149566771	1
BC034090	2.548008368	-1.684006554	0.280261075
Mpdz*	2.615862538	-2.926844893	0.011919111
Rbms3	2.617225647	-0.92115539	0.60754804
Kcnk10	2.628348402	-0.374690355	1
Dagla	2.631191554	-1.668349923	0.322143844
Nme4	2.634311601	-0.627015947	0.942415004
Gm42456	2.636415689	-0.395016401	1
Ptprd	2.65049284	-0.719243307	0.829485255
Mok	2.656376139	-0.092010753	1
Sntg2	2.659499815	-2.225958982	0.138928174
Aox1	2.680129862	-1.301464118	0.279059396
Ryr1	2.69972699	-0.217978474	1
Lurap1	2.751645102	-0.170602729	1
Gm12254	2.76041269	-0.547272295	1
Nol3	2.78085326	-1.675502125	0.349942253
Cd34	2.788164834	-0.878619114	0.628674907
Tbx3	2.802857571	-2.206599269	0.154915626
Cdkn1c	2.809097096	-0.747791487	0.833903614
Pnma3	2.812299205	0	1
Shisa2	2.813573031	-2.26027717	0.1272961
Nhs	2.826278109	-1.676751369	0.316574121
Cdh20	2.828543507	-1.989263694	0.210447249
Jam3	2.836642954	-0.640620945	0.949979445
Sox21	2.838460296	-0.020480935	1

Adcyap1r1*	2.838753572	-3.570195939	0.002522265
Clip4	2.841133438	-1.378611923	0.151507294
Cgref1	2.845970776	0	1
Fgd1*	2.846604526	-3.237124058	0.008917846
Gm17276	2.854182015	0	1
Tmc3	2.854415709	-0.139513992	1
Npr3	2.864360335	-0.261538703	1
Proca1*	2.882144993	-2.992794057	0.022831766
Togaram2	2.882481791	-2.165190297	0.173982574
4933412O06Rik	2.886370972	-0.758486482	0.895359281
Fgf2	2.918168013	-0.371717273	1
Kctd15	2.943263964	-2.126062351	0.100055595
1700007L15Rik	2.949974827	-0.257080641	1
Ccbe1	2.960286899	-0.348528641	1
Slc4a3	2.961208744	-1.229105943	0.516035683
Pla2g5	2.965280446	-0.539965039	1
Mfsd4b1	2.99555632	-2.093284326	0.156292813
Gm32282	2.998308486	-1.978725531	0.217241885
Calr3	3.005966637	-1.286615464	0.50711201
Lgr5	3.018276683	-1.615806018	0.26012425
Pdgfra	3.021142282	-0.103584476	1
Slc2a13	3.024367615	-1.862316944	0.196244133
Pth1r*	3.025491334	-3.955841785	0.000711651
Syn2	3.028438613	-1.670283538	0.350667603
Ntm	3.028934096	-2.479886602	0.075026399
Gm45605	3.032794841	-0.257149773	1
Pcdh18	3.040320279	-0.76829684	0.886438133
Dclk1	3.041253305	-1.621046382	0.256994456
Prox1	3.04125547	-1.325375571	0.375198962
Snx7	3.041394909	-0.051979958	1
Nexn	3.048105853	-2.245060884	0.13104215
4833417C18Rik	3.050036921	-0.844181359	0.731646273
Vwa5b2	3.063885148	-1.879664507	0.229479569
Gm44806	3.084328377	0	1
Cftr	3.089928164	-0.177706179	1
Sod3	3.095341737	-1.035534318	0.699472627
Hs3st3a1	3.095733553	-0.731250977	0.920009969
P2rx5	3.10333299	-0.897468279	0.804046688
8030456M14Rik	3.122525369	-0.990794503	0.655459161
A730063M14Rik	3.129109669	-1.084900685	0.684953924
Tmem132a*	3.131821369	-2.663672872	0.030571033
Tmem91	3.142662167	-0.885915505	0.633286584
Frmpd4	3.155827645	-0.771090485	0.883894979
Mme	3.165519989	-0.526478917	1

Gm16287	3.182931115	-2.224332527	0.187420602
Grb14	3.185941748	-2.24857664	0.127634509
Gm7429	3.196410679	-0.082195082	1
Cntn1	3.200684876	-2.093214541	0.149993259
Gm47079	3.211703519	-1.097703314	0.662855525
Sorbs2*	3.213415556	-1.695544681	0.016757493
Tmem132e*	3.216922517	-3.352449043	0.006011019
Aif1	3.227649204	-1.062417702	0.45120864
L3mbtl1	3.230478448	-3.051797297	0.013959082
Trp73	3.238175019	-1.178854176	0.530955341
Kcnn3	3.2468278	-0.644157828	0.951832658
Acss3	3.248836495	-0.704151016	0.887462652
Aldh3a1	3.262385646	-0.016204636	1
Tnnc1	3.267193306	-0.557823023	1
Arhgef26	3.271459708	-1.617161814	0.260591475
Gm49417	3.275202369	-1.142254493	0.706284994
Unc45b	3.284932374	-0.638263466	0.939056641
Sapcd2	3.310509776	-1.980428929	0.222325345
Hyal2	3.316735754	-0.362549614	1
Adgrb1	3.319217737	-1.721850344	0.217808663
Gm42716	3.322415054	-0.715831894	0.933860982
Kif26b	3.325974857	-2.033624317	0.071562228
Camsap3	3.326969581	-0.766577681	0.802062948
Bnc2	3.344631866	-0.401153533	1
Astn1	3.351818517	-1.899360634	0.217667194
Mmp15	3.35593409	-1.337973439	0.231164555
Gnai1	3.35733578	-1.352416681	0.457061177
Kcnj16	3.365882066	-0.780670488	0.706859367
Cyyr1	3.366167435	-2.403418373	0.062779198
9630028I04Rik	3.367286973	-0.640618988	0.963714857
Klhl33	3.373248389	-0.085215633	1
4930570G19Rik	3.379311007	-1.098599538	0.632449058
Tcap	3.391672461	-1.962488361	0.317057339
Gm26787	3.41175436	-2.815955571	0.119158262
Gm13830	3.431531691	-0.268906189	1
Lrp2	3.431531691	-0.637187571	0.964323006
Gm44524	3.436889042	-0.506173151	1
6030407O03Rik	3.457902822	-2.42151325	0.152810028
Kcnt2	3.458825796	-0.374534426	1
Amotl1	3.460947363	-0.784171776	0.616476908
Gm48765	3.475407898	-1.803930262	0.403488668
Peg3*	3.481369977	-2.897175788	0.020178263
Armh4	3.48896243	-0.626379096	0.947494509
Gm5621	3.514142323	-0.072811584	1

Gm27252*	3.545245376	-3.018795735	0.037194423
Rxfp2	3.552030939	-2.388867815	0.179032594
Insl6	3.565864504	-0.142504986	1
Col22a1	3.567835496	-3.050194166	0.070883772
Flnc	3.575809434	-0.656414473	0.943191581
Atoh8	3.579191703	-0.547274581	1
Cma1	3.609426653	-1.299848614	0.390244911
Kif17	3.6401914	-1.095774327	0.662988405
Fhod3*	3.647620532	-3.079724648	0.012575082
Tle6	3.649956684	-1.303456389	0.360810619
Mcc	3.652613774	-0.654390759	0.945775872
Bdh2	3.708716267	-2.048245282	0.116926115
Gm12657	3.715122367	-0.491279589	1
Gm48511	3.717812954	-0.124512215	1
Slc23a1	3.721742853	-0.918674104	0.746905172
Gm12917	3.737600463	-0.709667954	0.926866041
1810019N24Rik	3.780195704	-0.648525297	0.950592038
Gm5499	3.795083761	0	1
Pex11g*	3.853664828	-3.750409775	0.00554073
Slc26a1*	3.854370441	-2.954115138	0.015864413
Gm47861	3.889191814	-2.044663685	0.35535896
Lamb1	3.896520413	-0.260489172	1
Prok2	3.918224584	-1.663207621	0.533672694
Hspb8	3.976463301	-0.408926846	1
Vmn1r6	4.019478773	-0.228873777	1
Trpm1	4.032312283	-1.436688564	0.470561591
Pgam2	4.034285879	0	1
Gm42928	4.074014273	-1.778031866	0.347470626
Podn	4.081908122	-0.319213007	1
Gm14327	4.090442038	-0.650717236	0.953617872
Kcnab3	4.105459305	-0.309214627	1
Usp13	4.129555776	-1.013332479	0.576136148
Ms4a7	4.133975058	-0.609255537	0.987764998
Tln2	4.15078302	-0.873121697	0.771904974
Dnah9	4.169596871	-0.298329409	1
Zfp385c	4.181094191	-1.570876771	0.389906084
Cmya5	4.354072959	-1.373045774	0.219562815
Gm26670	4.397836165	-0.485211631	1
Gm10247	4.40243375	-1.967920925	0.430840549
Bcam	4.469850284	-1.407755766	0.375035085
Capn12	4.583910265	-1.526169934	0.571566969
Gpm6a	4.685023233	-0.050344019	1
Arhgdig	4.753265779	-2.190281362	0.253601043
Rapsn	5.32621318	-2.921760816	0.085905009

Scrn1	5.496589826	-1.174205347	0.684602422

Supplemental Table 14. List of all differentially expressed genes (P<0.05) in whole blood of wild-type B6129 CBD (week 0 to 8) that were reversed in expression in 3xTg-AD CBD (week 0 to 8) animals in comparison. *P<0.05, significant expression in the opposite direction in 3xTg-AD CBD

DEG (P<0.05): B6129 CBD	log2FoldChange: B6129 CBD	log2FoldChange: 3xTg-AD CBD	P-value: 3xTg-AD CBD
Lor	-6.402927876	0.612177976	0.985368087
Amn	-4.419033064	0.099640875	1
Krt25	-4.395865065	0.61500844	0.985797637
Postn	-4.323455833	0.73620094	0.890357988
Syt15	-4.320839379	0	1
Tchh	-4.263666872	1.841640342	0.215459661
Dnah14	-4.24914896	2.4573173	0.149794462
Gm38009	-4.247262258	1.329958641	0.505879911
ENSMUSG00000115801	-4.224632368	1.935078858	0.395123848
Arpin	-4.185358702	1.864954654	0.15520717
Grip2	-4.171795411	0	1
Trp63	-4.153229907	1.194569693	0.668255679
Gsg1l	-4.144347721	1.070147519	0.689698286
Galnt3	-4.138562205	0.832762284	0.780086094
Dcst1	-4.105908944	0	1
Csmd1	-4.096341375	0.899287195	0.781669342
Calcb	-4.084476991	2.559384383	0.121148459
Hmcn1	-4.069006801	0.521785837	1
Zfp786	-4.049131169	0.613172677	0.982680391
Slc12a8	-3.964443427	1.138006122	0.628607674
Pth1r*	-3.955841785	3.025491334	0.022708068
A930029G22Rik	-3.897829707	0	1
Kcnk9	-3.893577094	2.038968481	0.210957491
Il20ra	-3.865505837	0.098340656	1
Pex11g*	-3.750409775	3.853664828	0.005316492
Zfp13	-3.747168159	0.587879077	0.998180582
Gm46409	-3.745091794	0	1
A930037H05Rik	-3.739776464	0.046835321	1
Gm9967	-3.737715003	0.237681365	1
Slc5a1	-3.735730092	0	1
Abcc8	-3.706860548	0.613056909	0.982774019
Cyp26b1	-3.706063644	0.59838049	0.991778594
Fam83h	-3.68438193	1.328834876	0.493045235
4632428C04Rik	-3.67819733	0.249357486	1
Gm10719	-3.667114024	1.678690963	0.484602116
Asic1	-3.656847209	2.041179583	0.065561902
Slc9a2	-3.602441083	0.61322689	0.982760311
Radil	-3.592678902	0	1
Adcyap1r1*	-3.570195939	2.838753572	0.034305827

Lrrc18	-3.562824189	0	1
Dpp10	-3.561539048	0.024121965	1
Cdsn	-3.534825956	2.579995605	0.193538088
Dct	-3.5261141	1.227596701	0.658654104
Ecscr	-3.481937314	0.614145286	0.98421601
Kcnk2	-3.475449163	0	1
Esyt3	-3.475181486	2.026518318	0.257727151
Adgrb3	-3.474099031	0.61312603	0.98261213
Fras1	-3.472537485	1.563710909	0.30315531
Gpr63	-3.469174587	0	1
Pkdrej	-3.450123045	0.613668858	0.98343568
Gm14279	-3.444523882	0.614903013	0.985592213
Gm26788	-3.439713565	2.271446729	0.235846039
Gm5086	-3.437211853	0.08875417	1
Iqank1	-3.407644788	0.035602279	1
Fam110d	-3.394994043	0	1
Gabrb1	-3.367855626	0.613112767	0.982619429
Tmem72	-3.365784929	3.183560937	0.054099138
Kl	-3.364947791	0	1
Tmem132e*	-3.352449043	3.216922517	0.011379132
Chrm1	-3.348135007	0	1
Crhr1	-3.342122234	0	1
Zfhx2os	-3.318071007	0	1
Scel	-3.309785573	0.27398808	1
Vdr	-3.306931811	2.025216682	0.302092115
Nr2f2	-3.301440638	1.733167274	0.124491206
Sncg	-3.276921205	0	1
Tymp	-3.27127258	0.100002992	1
Thsd1	-3.260798321	1.951278754	0.237736484
Barx2	-3.259604558	0	1
Grip1	-3.254916083	0.613146603	0.982642175
Pcdhgc5	-3.253341585	1.160908083	0.649508046
Gabbr2	-3.250059891	0.329961394	1
Gm48600	-3.246024394	0	1
Nrxn3	-3.244468539	2.21406391	0.100189937
Pnma2	-3.237435041	0	1
Fgd1*	-3.237124058	2.846604526	0.034824214
Gpr3	-3.236382764	0	1
Lhx6	-3.236068243	0	1
Mirg	-3.225353274	2.606934447	0.065327355
Scarf2	-3.222069563	0.029600719	1
Ccdc63	-3.212969427	1.759380143	0.272440466
Tnnt2	-3.207954254	2.234440898	0.172835479
Gm10801	-3.184291808	1.132661267	0.546577468

Dnah7c	-3.169491361	1.172532048	0.648357315
Ror2	-3.162624212	1.190105407	0.65456514
Catsperg2	-3.145909125	2.984143293	0.06671358
Gm43609	-3.143262404	1.513796893	0.447708571
Papln	-3.142341354	0.946660272	0.734354918
Dipk1b	-3.131131805	0	1
Sox9	-3.130365781	0	1
Lrrc4	-3.12660807	0	1
Mapk10	-3.12392318	0.036054255	1
Grid2	-3.123498078	1.642269027	0.381174624
Bmper	-3.122607481	1.166047713	0.646319324
Nell1	-3.11734882	2.03090402	0.213652801
Frem2	-3.11714329	0.72649331	0.89061229
Gpc5	-3.116888426	0	1
Vat1l	-3.115089515	0	1
Gm5421	-3.114924426	0.485811105	1
Prr15	-3.112370846	0	1
C130075A20Rik	-3.111965109	2.346426964	0.119654419
Plekhb1	-3.111362156	0.441001719	1
Cntnap3	-3.110996222	2.59117719	0.108231061
Slco1a4	-3.106057901	1.651031699	0.379931352
Sez6	-3.08964791	0.021160603	1
Alk	-3.088112183	0.613240509	0.982780475
Lama2	-3.085713113	1.165275572	0.646085513
Fhod3*	-3.079724648	3.647620532	0.002652108
Tmem151a	-3.074488761	2.342545563	0.118035741
Frmd3	-3.065066444	0	1
B230206H07Rik	-3.058726777	0	1
L3mbtl1*	-3.051797297	3.230478448	0.011675021
Aspn	-3.041067775	0.612096446	0.983972615
Gm16570	-3.04043366	0.99393643	0.776238528
Gm27252*	-3.018795735	3.545245376	0.010965766
Serinc2	-2.993280309	0	1
Proca1*	-2.992794057	2.882144993	0.036085803
Myt1l	-2.990237585	1.164708001	0.645914887
Bcar1	-2.9877417	0.005105597	1
Dab1	-2.987562772	0	1
Myt1	-2.986600955	2.344470365	0.117445168
Asphd2	-2.984651558	0	1
Adgra1	-2.982157088	0	1
Lgi3	-2.979531043	0	1
Ntng1	-2.978826161	0.61314771	0.982643795
Igsf10	-2.978716488	0	1
Srrm4	-2.978033875	1.165563215	0.646172436

Hhipl1	-2.977654839	1.870883796	0.2074598
Celf6	-2.976940302	0	1
Nkain3	-2.973339034	0	1
Cdh12	-2.971254494	0.613162818	0.982665924
Col24a1	-2.962894879	0	1
Slc26a1*	-2.954115138	3.854370441	0.001499275
Nr2e1	-2.942870773	0	1
Pitpnm3	-2.929508188	0.613103279	0.982645614
Ano5	-2.927006559	1.648680137	0.386815771
Mpdz*	-2.926844893	2.615862538	0.049681437
Slc22a17	-2.922635784	1.034499399	0.482554035
Igkv1-133	-2.917405528	0.089388056	1
Serpina3f	-2.915152123	1.230772051	0.483202959
Peg3*	-2.897175788	3.481369977	0.004354363
Gm20501	-2.879740922	0	1
Tead2	-2.87706565	0.489620478	1
Zfp133-ps	-2.874690681	1.428060833	0.474744109
Nkd1	-2.873079843	1.640723396	0.384495171
Gm48691	-2.872393472	0.613601173	0.983329416
Igfbp3	-2.87158018	1.519979044	0.329007799
Igdcc3	-2.868256295	1.50453618	0.447503461
Apod	-2.843882264	0.613049405	0.982794848
Tmem178b	-2.838345201	0.61310543	0.982639668
Elfn1	-2.837357433	0.613090749	0.982680258
Rims1	-2.832973882	1.639212856	0.383353799
Omg	-2.83207612	0	1
Hpca	-2.829078154	0	1
Ano3	-2.827174519	0	1
Trank1	-2.824112811	1.161366403	0.648974362
Kcnh7	-2.82250027	0	1
P4ha2	-2.821933301	1.061951052	0.698505032
Tmem132c	-2.821623246	0	1
Elovl4	-2.821160607	0	1
Pcdhb13	-2.817558323	0	1
Tmem266	-2.814955732	1.166883658	0.646574675
Lca5l	-2.813317518	1.180146865	0.650922187
Slc35f4	-2.812199365	0	1
C2cd4c	-2.810035796	1.159048166	0.651677735
Gm43517	-2.804556854	0	1
Sema5b	-2.803535519	0	1
Fkbp10	-2.799584332	1.14873742	0.663838891
Agbl4	-2.790499395	0.029072242	1
Snx24	-2.787464275	1.459434817	0.454870206
Rasgrf1	-2.787441364	1.639550149	0.382447813

Ppfia3	-2.782559928	0.587502855	0.998416765
Cabyr	-2.779606136	1.970719291	0.229806983
Vasn	-2.771667096	0.421268336	1
Adamts18	-2.766715566	0	1
Mgat4c	-2.763731607	0	1
Zfp57	-2.725167931	0.613203676	0.982726015
Tfap2a	-2.702783519	0.59556069	0.993461472
Grin1	-2.693718756	0.036079337	1
Gm10800	-2.688388308	1.131555276	0.489855858
Chrd	-2.681077272	0	1
Ccdc85a	-2.678522531	0	1
Fam189a1	-2.674655637	0	1
Fxyd7	-2.672354129	0	1
Cobl	-2.670302129	2.354344652	0.114081355
Ajm1	-2.670246998	0	1
Lrp1b	-2.668828355	0	1
Sox8	-2.668222262	0	1
Map6d1	-2.666860178	0	1
Pcdhgb1	-2.666249474	0	1
Phkg1	-2.6661671	1.161245934	0.649114825
Sh3gl2	-2.665318046	0	1
Tmem132a*	-2.663672872	3.131821369	0.012414975
Lrrc7	-2.663538392	0	1
Cux2	-2.662730679	0.585055655	0.999964881
Tnfaip6	-2.661580913	0.586480301	0.999067712
Sez6l	-2.658338698	1.165844987	0.646257914
Dbx2	-2.656854011	0	1
Tenm3	-2.655898324	0.588861311	0.997566185
Gm10718	-2.635267656	1.148607707	0.480318471
Lrrc24	-2.620627024	0.03022931	1
Lamc2	-2.613983705	1.398192047	0.396793736
Slc6a7	-2.601234249	0	1
Ak4	-2.561194048	0.986934706	0.68273294
Spsb4	-2.550274965	0	1
Fat4	-2.539556261	0.019958978	1
Gm44067	-2.525988326	0.448680074	1
2810414N06Rik	-2.514433057	0.773397697	0.814531176
Myrip	-2.497826693	0	1
Gm10722	-2.497030268	2.171088698	0.110844528
Srgap1	-2.496886845	1.777390789	0.28766573
Wnk2	-2.492446398	0.937900964	0.631392149
Rgs5	-2.454326898	1.315794285	0.394806714
Grik4	-2.449661998	1.067168663	0.690586737
Zfp366	-2.419171056	0.6315891	0.953967598

Srcin1	-2.278062433	1.03221802	0.606285068
Gli3	-2.264298063	0.836676488	0.780044007
Lama4	-2.208470165	0.793674739	0.749111464
Snord13	-2.203699298	0.45109153	1
Col1a2	-2.140362916	0.720381146	0.829927272
Htr1b*	-2.116564066	2.051487814	0.036836417
Nuak1	-1.947390046	0.387953787	1
Gm48632	-1.911676303	1.081986896	0.539028374
Gm2415	-1.760386429	0.048429769	1
Sorbs2*	-1.695544681	3.213415556	0.005160917
Thtpa	-1.660093812	0.829869049	0.643428646
Fndc10	-1.657948431	0.274676958	1
Igkv4-59	-1.602078274	0.236073436	1
Zfp593	-1.596231205	0.950674713	0.364130357
Arl4d	-1.579836535	0.896393976	0.597207107
Fbxl15*	-1.465670628	1.914338062	0.020505529
Dcxr	-1.43121455	0.100980175	1
Zdhhc2	-1.353659295	0.945431691	0.363625009
Pald1	-1.326472487	0.763979749	0.721636462
Ddr1	-1.27992462	0.492674027	1
Poglut2	1.416036123	-0.378412615	1
Myb	1.609285849	-0.18707048	1
Lrrc27	1.708181112	-1.028984168	0.375000694
Gm37463	1.777038108	-0.22263275	1
5033421B08Rik	1.891920984	-0.053545442	1
2010310C07Rik*	1.959935631	-2.442927565	0.010689563
Gm14399	1.993646955	-0.528889236	1
Lama3	2.020014205	-0.196170797	1
Sall2	2.031764027	-0.001574344	1
Spats2l	2.065257301	-1.922287943	0.279364602
P3h3	2.114440683	-0.828393519	0.830655367
Morc4	2.136996362	-1.726929859	0.200038122
D130019J16Rik	2.168476208	-0.138399478	1
Slc16a2	2.3033124	-1.897581164	0.271890808
Spp1	2.332764756	-1.823594401	0.243179762
Gm37524	2.414793048	-0.744456753	0.788483401
Tmem169	2.432660804	-2.248416798	0.18795243
Gm37534	2.58799505	-2.227227104	0.09030488
Ltf	2.625916944	-0.190611398	1
Gm44175	2.633885348	-0.086848343	1
Gm15663	2.749111162	-1.8026746	0.233033852
ENSMUSG00002075075*	2.776380203	-3.242362932	0.013780806
Hmgcs2	2.782394207	0	1
Gm9042	2.815183629	-2.635916651	0.076049062

Cacng4	2.829538193	0	1
Spag6l	2.863958736	-1.107098885	0.681398193
Gm15743	2.952909423	-1.551363224	0.453922766
Vsnl1	2.979164975	0	1
Gm14681	2.984093519	-0.954667243	0.788409326
Gm14296	3.007353613	0	1
Zfp449	3.036725204	-0.965063642	0.622386321
Gxylt2	3.037319675	0	1
Ecrg4	3.039818528	0	1
Gm42729	3.171623965	0	1
Fabp3	3.193188349	0	1
Cstad	3.202375105	-1.907333255	0.269154385
Tas2r126	3.221718279	-2.203917951	0.166920245
Fbl-ps2	3.279156682	-0.506456969	1
Gm26571	3.287563591	-0.541006302	1
ENSMUSG00002075203	3.298923284	-0.799339238	0.840644976
Ccdc153	3.323950958	0	1
4930466K18Rik	3.358612899	-1.695918219	0.168467954
Chia1	3.376415036	-0.5410064	1
Gm37632	3.402119781	-1.168010253	0.715385506
Gm38318	3.414256753	-0.170705788	1
Gm44549	3.417373943	-0.722536441	0.886220488
Gm41077	3.422433824	-0.934096865	0.818569592
Gm43186	3.461072555	0	1
Gm45540	3.465665223	-0.541006232	1
Tdrd5*	3.508472105	-16.26321405	7.59E-18
Ccdc3	3.554657764	0	1
Gm49784	3.563579017	-1.601944793	0.420857898
Gm16897	3.615881646	-1.527281532	0.510291777
2410004P03Rik	3.678127393	0	1
Rorc	3.68021463	-2.472219943	0.207115684
Gm10702	3.762127019	-0.541006296	1
Gm47373	3.842488486	-0.900515772	0.850877291
Gm37548	3.915750665	0	1
Gsta3	4.064312381	-1.134021701	0.695134625
Gm43787	4.329095321	-0.441955691	1
BB031773	4.518953852	0	1
Dntt	4.701255844	-1.511920549	0.683866275
Gm26930	4.812273381	-0.054411886	1
7SK	4.87829218	-1.489022393	0.599423511
H2-Q2	6.177310338	0	1

Table of Contents
Top of Current Table

Supplemental Table 15. IPA Canonical Pathways associated with differentially expressed (P<0.05) genes in whole blood of 3xTg-AD vehicle (week 0 to 8) that were reversed in expression in wild-type B6129 vehicle (week 0 to 8) animals in comparison. Note that the -log(p-value) threshold was set at 1.30 for either comparison group. Pathways in italicized text indicate opposite z-scores from upregulated to downregulated from 3xTg-AD to B6129 animals respectively; some fields represent insufficient information to determine a z-score.

**Canonical Pathways**	**Gene Name**	**3xTg-AD VEH wk 8 vs. wk 0**		**WT VEH wk 8 vs. wk 0**	
		**-log(p-value)**	**z-score**	**-log(p-value)**	**z-score**
Endocannabinoid Neuronal Synapse Pathway	CACNA2D3, PRKAR1B	1.31	0.816	2.23	-1.667
Breast Cancer Regulation by Stathmin1	PRKAR1B	1.34	2.683	2.75	-2.449
G-Protein Coupled Receptor Signaling	PRKAR1B	1.36	1.46	3.98	-1.768
Netrin Signaling	CACNA2D3, PRKAR1B	1.38	0.707	1.39	-0.707
cAMP-mediated signaling	PRKAR1B	1.77	0	1.43	-1.667
BMP signaling pathway	PRKAR1B	1.8	2	1.81	-1
Neurotransmitter release cycle	LIN7A, PPFIA2, SLC17A7	1.93	2	1.94	-1
Potassium Channels	KCNQ4	2.09	1.134	7.21	-2.673
Molecular Mechanisms of Cancer	PRKAR1B	2.25	1.257	2.29	-1.976
CREB Signaling in Neurons	CACNA2D3, PRKAR1B	2.29	3.128	4.79	-3.157
S100 Family Signaling Pathway	CACNA2D3, ETV4, PRKAR1B	2.35	2.785	3.93	-2.744
Neurovascular Coupling Signaling Pathway	CACNA2D3, PRKAR1B	2.66	2.496	3.17	-2.138
Synaptogenesis Signaling Pathway	PRKAR1B	3.1	3	2.71	-3
Extracellular matrix organization	NCAN	4.61	3.317	3.25	-1.667
Axonal Guidance Signaling	PRKAR1B, SRGAP1	3.65	---	3.7	---
Signaling by FGFR1	FRS3	1.61	1	1.62	2
Acute Phase Response Signaling	RBP4	2.53	---	2.09	0

Table of Contents

Supplemental Table 16. IPA Canonical Pathways associated with differentially expressed (P<0.05) genes in whole blood of wild-type B6129 vehicle (week 0 to 8) that were reversed in expression in *3xTg-AD* vehicle (week 0 to 8) animals in comparison. Note that the -log(p-value) threshold was set at 1.30 for either comparison group; pathways in italicized text indicate opposite z-scores from wild-type B6129 vehicle to *3xTg-AD* vehicle animals respectively & some fields represent insufficient information to determine a z-score.

**Canonical Pathways**	**Gene Name**	**WT VEH wk 8 vs. wk 0**		**3xTg-AD VEH wk 8 vs. wk 0**	
		**-log(p-value)**	**z-score**	**-log(p-value)**	**z-score**
Neurotransmitter release cycle	LIN7A, PPFIA2, SLC17A7	1.94	-1	1.93	2
Endocannabinoid Neuronal Synapse Pathway	PRKAR1B	2.23	-1.667	1.31	0.816
Molecular Mechanisms of Cancer	PRKAR1B	2.29	-1.976	2.25	1.257
Synaptogenesis Signaling Pathway	PRKAR1B	2.71	-3	3.1	3
Breast Cancer Regulation by Stathmin1	PRKAR1B	2.75	-2.449	1.34	2.683
Neurovascular Coupling Signaling Pathway	PRKAR1B	3.17	-2.138	2.66	2.496
Extracellular matrix organization	NCAN	3.25	-1.667	4.61	3.317
S100 Family Signaling Pathway	ETV4, PRKAR1B	3.93	-2.744	2.35	2.785
CREB Signaling in Neurons	PRKAR1B	4.79	-3.157	2.29	3.128
Potassium Channels	KCNQ4	7.21	-2.673	2.09	1.134
Transport of inorganic cations/anions & amino acids/oligopeptides	SLC17A7	2.67	-2.121	0.697	2
Class B/2 (Secretin family receptors)	PTH1R	1.23	-1.342	1.72	0.816
Osteoarthritis Pathway	RBP4	1.1	0.378	1.41	1.134
Glycosaminoglycan metabolism	NCAN	0.998	0	2.06	1.633
Acute Phase Response Signaling	RBP4	2.09	0	2.53	---
Neurexins and neuroligins	LIN7A	0.883	---	1.42	2
Glutamate Receptor Signaling	SLC17A7	3.21	-2.236	1.23	---
Axonal Guidance Signaling	PRKAR1B, SRGAP1	3.7	---	3.65	---
Transcriptional Regulation by NPAS4	IQSEC3	0.266	---	1.37	---

Table of Contents

Supplemental Table 17. IPA Canonical Pathways associated with differentially expressed (P<0.05) genes in whole blood of *3xTg-AD* vehicle (week 0 to 8) that were muted in expression in *3xTg-AD* CBD (week 0 to 8) animals in comparison. Note that the -log(p-value) threshold was set at 1.30 for either comparison group; some fields represent insufficient information to determine a z-score.

**Canonical Pathways**	**Gene Name**	**3xTg-AD VEH wk 8 vs. wk 0**		**3xTg-AD CBD wk 8 vs. wk 0**	
		**-log(p-value)**	**z-score**	**-log(p-value)**	**z-score**
Transcriptional Regulatory Network in Embryonic Stem Cells	SMAD9	1.93	-0.333	0.589	1.633
Osteoarthritis Pathway	SMAD9	1.41	1.134	0.791	0.378
Human Embryonic Stem Cell Pluripotency	SMAD9	1.8	1.265	0.56	1.89
S100 Family Signaling Pathway	SMAD9	2.35	2.785	0.556	3.128
Adipogenesis pathway	SMAD9	1.9	0.707	0.533	0.447
ID1 Signaling Pathway	SMAD9	2.69	0.577	0.366	1.633
Myelination Signaling Pathway	SMAD9	1.49	0.277	0	2.828
Molecular Mechanisms of Cancer	SMAD9	2.25	1.257	0	2.524
Cardiomyocyte Differentiation via BMP Receptors	SMAD9	1.67	---	0.294	---
Role of NANOG in Mammalian Embryonic Stem Cell Pluripotency	SMAD9	1.65	---	0	---
BMP signaling pathway	SMAD9	1.8	2	0	---

Table of Contents

Supplemental Table 18. IPA Canonical Pathways associated with differentially expressed (P<0.05) genes in whole blood of B6129 vehicle (week 0 to 8) that were muted or reversed in expression in B6129 CBD (week 0 to 8) animals in comparison. Note that the -log(p-value) threshold was set at 1.30 for either comparison group; pathways in italicized text indicate opposite z-scores from B6129 vehicle to B6129 CBD animals respectively.

**Ingenuity Canonical Pathways**	**Gene Name**	**WT VEH wk 8 vs. wk 0**		**WT CBD wk 8 vs. wk 0**	
		**-log(p-value)**	**z-score**	**-log(p-value)**	**z-score**
Breast Cancer Regulation by Stathmin1	PRKD1	2.75	-2.449	2	-2.065
CREB Signaling in Neurons	PRKD1	4.79	-3.157	2.89	-2.558
DHCR24 Signaling Pathway	PRKD1, RBP4	0.714	-1.342	1.93	-0.378
Phagosome Formation	PRKD1, S100B	3.37	-1.826	2.19	-2.132
S100 Family Signaling Pathway	PRKD1, S100B	3.93	-2.744	2.87	-1.961
Glutaminergic Receptor Signaling Pathway (Enhanced)	PRKD1	5.38	-2.558	1.86	0
GP6 Signaling Pathway	PRKD1	2.64	-2.333	5.5	-1.732
Hepatic Fibrosis Signaling Pathway	PRKD1	1.87	0.577	2.43	-0.258
Neutrophil Extracellular Trap Signaling Pathway	PRKD1	2.09	-0.243	1.91	1.069

Table of Contents

Supplemental Table 19. IPA Canonical Pathways associated with differentially expressed (P<0.05) genes in whole blood of *3xTg-AD* CBD (week 0 to 8) that were reversed in expression in wild-type B6129 CBD (week 0 to 8) animals in comparison. Note that the -log(p-value) threshold was set at 1.30 for either comparison group. Some fields represent insufficient information to determine a z-score.

**Canonical Pathways**	**Gene Name**	**3xTg-AD CBD wk 8 vs. wk 0**		**WT CBD wk 8 vs. wk 0**	
		**-log(p-value)**	**z-score**	**-log(p-value)**	**z-score**
Phagosome Formation	ADCYAP1R1, HTR1B, PTH1R	1.01	3.674	2.19	-2.132
CREB Signaling in Neurons	ADCYAP1R1, HTR1B, PTH1R	0.94	2.524	2.89	-2.558
S100 Family Signaling Pathway	ADCYAP1R1, HTR1B, PTH1R	0.556	3.128	2.87	-1.961
G-Protein Coupled Receptor Signaling	ADCYAP1R1, HTR1B, PTH1R	0.429	3.13	1.59	-2.236
Breast Cancer Regulation by Stathmin1	ADCYAP1R1, HTR1B, PTH1R	0.422	3.153	2	-2.065
Molecular Mechanisms of Cancer	ADCYAP1R1, HTR1B, PTH1R	0	2.524	1.29	-2.558
Class B/2 (Secretin family receptors)	ADCYAP1R1, PTH1R	0.996	2.236	3.56	-1.414
Tight Junction Signaling	MPDZ	1.34	---	0.673	---

Table of Contents

Supplemental Table 20. IPA Canonical Pathways associated with differentially expressed (P<0.05) genes in whole blood of wild-type B6129 CBD (week 0 to 8) that were also upregulated in expression with an enhanced z-score in *3xTg-AD* CBD (week 0 to 8) animals in comparison. Note that the -log(p-value) threshold was set at 1.30 for either comparison group.

**Canonical Pathways**	**Gene Name**	**WT CBD wk 8 vs. wk 0**		**3xTg-AD CBD wk 8 vs. wk 0**	
		**-log(p-value)**	**z-score**	**-log(p-value)**	**z-score**
Regulation of Insulin-like Growth Factor (IGF) transport & uptake by IGFBPs	TMEM132A	2.19	1.134	1.81	2.828
Post-translational protein phosphorylation	TMEM132A	1.93	0.816	1.67	2.646

Table of Contents

Supplemental Table 21. List of all significant ($P < 0.05$) differentially expressed genes in whole blood of 3xTg-AD vehicle (week 8) versus wild-type B6129 vehicle (week 8) animals; 523 genes downregulated & 494 genes upregulated. Italicized genes have a recognized association with Alzheimer's disease pathology as exact matches with *3xTg-AD* vehicle (week 8) versus *3xTg-AD* vehicle (week 0) (Figure 1A & Supplemental Table 1)

Gene Name	Log2FoldChange	P-value	Regulation
Tdrd5	-20.6002354	3.429E-30	Downregulated
H2-Q2	-19.7539260	2.054E-31	Downregulated
Sult4a1	-9.4788028	6.068E-06	Downregulated
Gm35082	-8.7024703	1.978E-07	Downregulated
Dntt	-8.5399013	1.395E-03	Downregulated
Gm49980	-7.8094354	1.435E-02	Downregulated
H2af-ps2	-7.5365688	4.048E-04	Downregulated
Rpl31-ps15	-7.2837249	4.707E-06	Downregulated
Rps3a3	-7.0387034	1.072E-07	Downregulated
Gm6916	-6.9260428	4.688E-04	Downregulated
4930447F24Rik	-6.7046262	4.353E-05	Downregulated
Cib3	-6.0055537	1.725E-03	Downregulated
Loxl2	-5.9961341	5.863E-04	Downregulated
Cpne4	-5.8096450	2.666E-05	Downregulated
Ighv2-9	-5.7927991	1.301E-04	Downregulated
Col6a4	-5.7760198	7.162E-05	Downregulated
Zfp951	-5.4652379	5.914E-05	Downregulated
Eps8l1	-5.3965996	5.085E-03	Downregulated
Ighv1-14	-5.1768400	3.805E-02	Downregulated
Gm13307	-5.0725112	7.094E-04	Downregulated
Fam71b	-5.0330270	9.793E-03	Downregulated
Traj22	-4.9798536	1.342E-02	Downregulated
Ramp3	-4.9437792	9.343E-06	Downregulated
Rag1	-4.9433504	1.669E-03	Downregulated

Gm10548	-4.8302239	9.871E-05	Downregulated
Adgrl4	-4.7985238	6.540E-04	Downregulated
Gm48720	-4.7903669	1.171E-02	Downregulated
Gm15459	-4.7787944	1.295E-06	Downregulated
Gm26930	-4.6933894	5.786E-03	Downregulated
Tamalin	-4.6764364	2.257E-05	Downregulated
H4c17	-4.6561397	2.582E-03	Downregulated
Kcnh3	-4.6446399	6.395E-04	Downregulated
Gm36266	-4.6321557	1.194E-02	Downregulated
Gm34590	-4.6166380	3.619E-03	Downregulated
Sema3c	-4.5842067	7.259E-05	Downregulated
Pawr	-4.5612391	5.563E-04	Downregulated
D130019J16Rik	-4.5422715	1.290E-03	Downregulated
1810059H22Rik	-4.5145861	2.042E-02	Downregulated
Gm42743	-4.4845248	4.578E-03	Downregulated
Pbx4	-4.4378659	1.790E-04	Downregulated
Mtus2	-4.4317921	7.132E-04	Downregulated
2700054A10Rik	-4.4230613	2.078E-03	Downregulated
A130050O07Rik	-4.4146962	3.293E-03	Downregulated
Eno1b	-4.4001848	3.628E-02	Downregulated
Gm3650	-4.3941297	2.040E-02	Downregulated
Fgfrl1	-4.3717457	7.783E-07	Downregulated
Arntl2	-4.3401052	6.459E-04	Downregulated
Gm11695	-4.3253242	3.793E-02	Downregulated
Gm37766	-4.3171137	1.853E-03	Downregulated
Gm44888	-4.3132611	4.854E-04	Downregulated
Sema4c	-4.3010901	4.937E-04	Downregulated
Gbp2b	-4.3001068	2.132E-02	Downregulated

Prtn3	-4.2914802	6.228E-03	Downregulated
Prrt1	-4.2268248	6.879E-03	Downregulated
Pcp4l1	-4.2169935	7.318E-09	Downregulated
Ppp1r3e	-4.1666691	4.662E-03	Downregulated
Rpl21-ps15	-4.1432427	1.416E-02	Downregulated
Gask1b	-4.1361223	1.724E-03	Downregulated
Prss57	-4.1183649	1.194E-02	Downregulated
Jam2	-4.0988692	3.212E-04	Downregulated
Ubb-ps	-4.0853896	1.672E-04	Downregulated
Gm9008	-4.0811611	4.456E-02	Downregulated
Gm28707	-4.0798532	5.591E-03	Downregulated
Gm20186	-4.0674196	1.271E-03	Downregulated
Angptl6	-4.0366819	6.418E-04	Downregulated
Gvin-ps1	-4.0356588	1.045E-03	Downregulated
Hunk	-4.0331630	1.577E-03	Downregulated
Gm15708	-4.0326540	3.213E-03	Downregulated
A3galt2	-4.0272723	2.558E-03	Downregulated
BC016579	-4.0249143	6.366E-03	Downregulated
Eif3j2	-3.9888583	1.143E-06	Downregulated
Slc23a4	-3.9867987	7.959E-03	Downregulated
Trpm1	-3.9413975	2.666E-02	Downregulated
Ephb6	-3.9159841	7.344E-05	Downregulated
Trbj1-6	-3.9140603	3.235E-02	Downregulated
Gm37062	-3.9085315	1.498E-03	Downregulated
Snora41	-3.9024964	4.709E-03	Downregulated
Rimkla	-3.9021042	7.196E-04	Downregulated
ENSMUSG00002075672	-3.8848009	8.161E-03	Downregulated
Gm19427	-3.8688466	1.050E-02	Downregulated

E230020D15Rik	-3.8603811	1.661E-03	Downregulated
Gm15503	-3.8599524	5.808E-03	Downregulated
Cfap126	-3.8563852	9.867E-03	Downregulated
Map2k3os; lncRNA	-3.8509052	2.148E-03	Downregulated
Gm43062	-3.8387274	2.394E-03	Downregulated
Gm17072	-3.8308460	6.602E-03	Downregulated
Gm7285	-3.8254761	4.887E-03	Downregulated
Siah3	-3.8249819	3.406E-03	Downregulated
Lims2	-3.8235591	1.214E-03	Downregulated
Gm16092	-3.8188738	7.382E-03	Downregulated
Zfp365	-3.8074497	7.956E-04	Downregulated
Homer2	-3.8072482	1.903E-02	Downregulated
Gm44835	-3.7867874	1.504E-02	Downregulated
Rps13-ps1	-3.7807765	4.470E-04	Downregulated
Gm43173	-3.7762026	1.076E-02	Downregulated
Gm45640	-3.7621985	1.774E-02	Downregulated
Whrn	-3.7577882	1.786E-05	Downregulated
Endou	-3.7495285	1.031E-02	Downregulated
Ankrd45	-3.7366642	1.431E-03	Downregulated
Gm9493	-3.7358855	1.675E-02	Downregulated
Rangrf	-3.7311296	1.796E-03	Downregulated
Kcnip3	-3.7310046	1.399E-03	Downregulated
Kcnmb1	-3.7298044	1.500E-02	Downregulated
Gm14287	-3.7183893	4.269E-02	Downregulated
3110080O07Rik	-3.7062522	6.034E-03	Downregulated
Gm49703	-3.7060671	3.989E-03	Downregulated
Gfra1	-3.6863843	6.454E-03	Downregulated
Gm10044	-3.6760998	4.748E-02	Downregulated

Gm44174	-3.6563616	4.219E-02	Downregulated
Gm11868	-3.6561674	6.721E-03	Downregulated
Gm37124	-3.6556422	3.598E-02	Downregulated
Mir6999	-3.6545841	1.799E-02	Downregulated
Synm	-3.6483325	1.513E-03	Downregulated
Tma7-ps	-3.6455862	5.226E-04	Downregulated
Rpl34-ps1	-3.6415661	1.311E-03	Downregulated
Fbln2	-3.6365428	3.874E-03	Downregulated
Cma1	-3.6354194	9.745E-03	Downregulated
Gssos2	-3.6351927	4.275E-02	Downregulated
Gm20768	-3.6261172	1.633E-02	Downregulated
Gm43307	-3.6196797	1.675E-02	Downregulated
Gm29112	-3.6194142	1.150E-02	Downregulated
Gm42727	-3.6154698	1.770E-02	Downregulated
A430105J06Rik	-3.6036716	1.182E-02	Downregulated
Camk2a	-3.6031715	2.006E-02	Downregulated
Wtip	-3.5977220	7.261E-03	Downregulated
Gm12312	-3.5834070	1.371E-02	Downregulated
2310044K18Rik	-3.5781417	4.262E-02	Downregulated
Rnf227	-3.5779353	2.625E-03	Downregulated
Efr3b	-3.5764892	2.371E-03	Downregulated
Ggn	-3.5599326	3.611E-02	Downregulated
Rin1	-3.5399997	1.072E-02	Downregulated
Gm44975	-3.5297334	1.131E-02	Downregulated
Slc7a2	-3.5229259	8.088E-03	Downregulated
Gm38304	-3.5078047	1.236E-02	Downregulated
Unc5a	-3.5074792	3.883E-03	Downregulated
Gm17191	-3.5070651	5.560E-03	Downregulated

Gxylt2	-3.5003233	7.537E-03	Downregulated
Filip1	-3.4958292	5.202E-03	Downregulated
Mir181a-1hg	-3.4844780	4.962E-02	Downregulated
Gm42866	-3.4776020	6.538E-03	Downregulated
Dhrs13	-3.4764350	1.146E-03	Downregulated
Gm26850	-3.4599728	3.364E-02	Downregulated
Gm48582	-3.4564215	7.320E-03	Downregulated
Rhbdl1	-3.4556702	1.109E-02	Downregulated
F12	-3.4443323	3.242E-02	Downregulated
C920006O11Rik	-3.4233802	8.325E-03	Downregulated
Acan	-3.4223788	4.284E-02	Downregulated
Olfr1033	-3.4063299	1.457E-02	Downregulated
Gm42433	-3.3866158	5.339E-03	Downregulated
Gm38257	-3.3748054	7.094E-03	Downregulated
6030442K20Rik	-3.3744773	1.969E-02	Downregulated
Pcsk4	-3.3742578	8.733E-03	Downregulated
Ldhd	-3.3682008	5.042E-03	Downregulated
Gm16794	-3.3673240	2.093E-02	Downregulated
Pex11g	-3.3617145	2.303E-02	Downregulated
Laptm4b	-3.3591380	1.526E-02	Downregulated
Gm37160	-3.3576184	1.399E-02	Downregulated
Trbv13-1	-3.3548451	6.000E-03	Downregulated
Slc25a2	-3.3255278	1.604E-02	Downregulated
Tmem198	-3.3247103	5.000E-03	Downregulated
AI847159	-3.3222571	2.691E-02	Downregulated
Rarb	-3.3173540	1.703E-02	Downregulated
Rorc	-3.3143074	3.945E-02	Downregulated
Trim34b	-3.3135416	1.702E-02	Downregulated

Gm15943	-3.3085990	1.630E-02	Downregulated
4930481B07Rik	-3.3068562	9.042E-03	Downregulated
Map1a	-3.3039320	1.946E-10	Downregulated
Acvr1	-3.3030351	7.742E-03	Downregulated
Star	-3.2986442	4.812E-04	Downregulated
Gm45570	-3.2857931	2.094E-02	Downregulated
Ksr2	-3.2826524	4.649E-03	Downregulated
Gm15859	-3.2798341	1.798E-02	Downregulated
Glt8d2	-3.2601149	8.353E-03	Downregulated
Gm17096	-3.2437719	2.420E-02	Downregulated
Gm45356	-3.2423326	2.483E-02	Downregulated
Mir670hg	-3.2383002	1.941E-02	Downregulated
Lcn4	-3.2226162	4.621E-02	Downregulated
Cpa3	-3.2148894	6.236E-03	Downregulated
Gm31323	-3.2099585	4.198E-02	Downregulated
Zfp82	-3.2091247	3.164E-02	Downregulated
Iqck	-3.2079737	2.000E-02	Downregulated
Snord35a	-3.1987205	4.405E-02	Downregulated
P4ha2	-3.1934617	1.008E-02	Downregulated
Gm37537	-3.1913036	2.283E-02	Downregulated
Mmp15	-3.1872096	1.632E-02	Downregulated
Atg9b	-3.1861466	1.813E-02	Downregulated
Runx2os1	-3.1830624	4.529E-02	Downregulated
Gm37607	-3.1736784	1.251E-02	Downregulated
Cc2d2a	-3.1688488	8.376E-03	Downregulated
Gipr	-3.1668377	1.325E-02	Downregulated
Pcdhga9	-3.1666718	1.097E-02	Downregulated
Lrig3	-3.1652975	1.205E-02	Downregulated

Gabra3	-3.1650791	1.050E-02	Downregulated
Gm48279	-3.1631567	4.687E-02	Downregulated
Gm47113	-3.1567974	3.660E-02	Downregulated
Acot1	-3.1470557	1.396E-02	Downregulated
Gm4787	-3.1429941	1.426E-02	Downregulated
Galnt9	-3.1230997	9.842E-03	Downregulated
Gm43024	-3.1218217	8.815E-03	Downregulated
Gm49164	-3.1215239	3.712E-02	Downregulated
Gm13270	-3.1149760	2.521E-02	Downregulated
Notch3	-3.0921072	4.036E-02	Downregulated
2810429I04Rik	-3.0897181	1.494E-02	Downregulated
Gm34655	-3.0888698	3.099E-02	Downregulated
Gm45534	-3.0822418	2.662E-02	Downregulated
Gm43420	-3.0789282	2.954E-02	Downregulated
F2rl2	-3.0777342	2.212E-03	Downregulated
Gm28941	-3.0768487	3.673E-02	Downregulated
Gm42372	-3.0670312	1.050E-02	Downregulated
Tead1	-3.0606093	3.400E-02	Downregulated
Gm49197	-3.0593801	3.396E-02	Downregulated
Ace2	-3.0592669	2.980E-02	Downregulated
Gm37973	-3.0579513	3.927E-02	Downregulated
Gm37672	-3.0524241	3.140E-02	Downregulated
Clec4g	-3.0442206	1.719E-02	Downregulated
Ablim3	-3.0423883	2.102E-02	Downregulated
Slc39a2	-3.0418287	2.344E-02	Downregulated
Gm25821	-3.0410239	1.391E-02	Downregulated
Gm15667	-3.0407840	4.019E-02	Downregulated
Apln	-3.0406419	1.700E-02	Downregulated

Tmem179	-3.0403950	1.632E-02	Downregulated
Phldb2	-3.0402836	1.736E-02	Downregulated
Grem2	-3.0383756	1.644E-02	Downregulated
6330409D20Rik	-3.0380221	2.471E-02	Downregulated
Tagln3	-3.0369849	1.578E-02	Downregulated
Pcdhga8	-3.0329226	1.694E-02	Downregulated
Rac3	-3.0327175	1.828E-02	Downregulated
Lefty1	-3.0308590	4.436E-02	Downregulated
Mmp2	-3.0224728	2.148E-02	Downregulated
Gm14269	-3.0200719	3.330E-02	Downregulated
1700048O20Rik	-3.0084561	2.567E-02	Downregulated
Gm33195	-3.0061599	4.825E-02	Downregulated
Nrg1	-2.9925493	3.430E-02	Downregulated
Slc6a19	-2.9728206	3.208E-02	Downregulated
A230103J11Rik	-2.9712057	3.216E-02	Downregulated
0610038B21Rik	-2.9597607	4.067E-02	Downregulated
Cst7	-2.9566885	6.113E-03	Downregulated
Gm26064	-2.9449230	4.680E-02	Downregulated
Ubc	-2.9446632	1.220E-05	Downregulated
Mpzl2	-2.9208613	2.952E-02	Downregulated
Mmp28	-2.9203355	3.015E-02	Downregulated
Gm43412	-2.9161095	4.593E-02	Downregulated
Rnf150	-2.9142621	1.119E-05	Downregulated
Kazn	-2.9135354	2.741E-02	Downregulated
Gm43072	-2.9132464	3.082E-02	Downregulated
4933406I18Rik	-2.9071825	2.974E-02	Downregulated
Cd276	-2.9056495	2.792E-02	Downregulated
Adamtsl1	-2.9046338	1.956E-02	Downregulated

Clip3	-2.9039753	2.418E-02	Downregulated
Anks6	-2.9028560	2.541E-02	Downregulated
Neil2	-2.9024285	2.827E-02	Downregulated
Baiap2l1	-2.9018556	4.754E-02	Downregulated
Celf4	-2.8989761	2.353E-02	Downregulated
U6	-2.8969537	1.221E-02	Downregulated
4833412K13Rik	-2.8957275	3.021E-02	Downregulated
Htra3	-2.8956224	3.135E-02	Downregulated
Gm4247	-2.8943796	2.910E-02	Downregulated
Gm43187	-2.8937817	2.894E-02	Downregulated
Adcy1	-2.8917907	2.525E-02	Downregulated
Gm7666	-2.8907818	6.497E-03	Downregulated
Gm42615	-2.8879778	3.166E-02	Downregulated
Gm42688	-2.8852506	6.069E-03	Downregulated
Mpdz	-2.8821048	1.742E-02	Downregulated
Camk4	-2.8723835	5.276E-04	Downregulated
5830468F06Rik	-2.8668451	2.407E-02	Downregulated
Aqp11	-2.8499471	4.794E-02	Downregulated
F2rl1	-2.8446965	6.822E-03	Downregulated
Gm37298	-2.8370355	3.524E-02	Downregulated
Col23a1	-2.8231291	1.945E-02	Downregulated
Col8a2	-2.8204062	3.676E-02	Downregulated
Sncaip	-2.8137305	2.664E-02	Downregulated
Gm42883	-2.8136822	3.255E-02	Downregulated
Etv1	-2.8131591	2.592E-02	Downregulated
Schip1	-2.8074449	2.537E-02	Downregulated
Mrps12	-2.8031625	1.075E-04	Downregulated
Capza1-ps1	-2.7999903	1.867E-03	Downregulated

Stxbp6	-2.7947973	1.012E-02	Downregulated
Garnl3	-2.7929913	1.366E-02	Downregulated
Gm47512	-2.7811292	4.273E-02	Downregulated
Rapgef3	-2.7771491	2.100E-02	Downregulated
Zfp811	-2.7704984	1.324E-02	Downregulated
Lypd6b	-2.7691865	4.825E-03	Downregulated
Oasl2	-2.7631404	4.700E-02	Downregulated
Cybrd1	-2.7624855	4.764E-02	Downregulated
4931415C17Rik	-2.7623482	4.326E-02	Downregulated
Igf1	-2.7571656	4.192E-02	Downregulated
Spp1	-2.7569206	4.845E-02	Downregulated
Rab40b	-2.7520304	3.757E-02	Downregulated
Pcdhgb7	-2.7501380	3.764E-02	Downregulated
C430019N01Rik	-2.7493355	2.010E-02	Downregulated
Fam171a1	-2.7488872	3.635E-02	Downregulated
Slc7a14	-2.7488437	3.678E-02	Downregulated
Fhod3	-2.7480934	3.996E-02	Downregulated
Gm15513	-2.7463488	4.334E-02	Downregulated
Slit3	-2.7445325	2.701E-02	Downregulated
Spsb4	-2.7402339	4.047E-02	Downregulated
Tmem121	-2.7398662	3.895E-02	Downregulated
Gjc1	-2.7384793	4.230E-02	Downregulated
Ccr9	-2.7302813	2.693E-02	Downregulated
Bco2	-2.7053402	4.306E-02	Downregulated
Pfn2	-2.7041149	1.593E-02	Downregulated
Gm37666	-2.7021197	4.021E-02	Downregulated
Trnp1	-2.7002116	3.475E-02	Downregulated
Tgfb2	-2.6995132	1.470E-02	Downregulated

Scg5	-2.6988194	1.608E-02	Downregulated
Wfs1	-2.6950015	6.968E-03	Downregulated
Cacna2d4	-2.6843586	1.467E-03	Downregulated
Gm49396	-2.6815419	6.886E-03	Downregulated
Gm29670	-2.6645447	2.663E-02	Downregulated
Maoa	-2.6511985	1.884E-02	Downregulated
Klhl20	-2.6502797	2.768E-07	Downregulated
Gm43017	-2.6491945	4.760E-02	Downregulated
Lbhd1	-2.6491480	1.680E-02	Downregulated
Trat1	-2.6226559	1.118E-03	Downregulated
Adamts14	-2.6130479	2.137E-04	Downregulated
Ltk	-2.6099745	2.950E-04	Downregulated
Auts2	-2.5917967	1.196E-02	Downregulated
Mcam	-2.5917609	1.324E-02	Downregulated
Maneal	-2.5886261	4.663E-02	Downregulated
Gprasp2	-2.5864547	4.724E-02	Downregulated
Npl	-2.5853887	2.660E-02	Downregulated
Tmem108	-2.5847266	8.122E-03	Downregulated
Lama2	-2.5822848	4.788E-02	Downregulated
Mycl	-2.5817410	4.653E-02	Downregulated
Igkv3-12	-2.5464689	3.372E-02	Downregulated
Rgs11	-2.5406556	1.368E-02	Downregulated
Ikzf4	-2.5385120	5.198E-03	Downregulated
P3h4	-2.5347582	2.744E-02	Downregulated
4930417O13Rik	-2.5081412	9.371E-03	Downregulated
Gm37534	-2.5044611	4.700E-02	Downregulated
Tanc1	-2.4891178	2.111E-09	Downregulated
Zfp286	-2.4841898	4.341E-03	Downregulated

Krt222	-2.4828802	3.812E-02	Downregulated
Eomes	-2.4659793	4.979E-03	Downregulated
Nhsl1	-2.4631745	1.880E-02	Downregulated
Them4	-2.4513886	1.353E-02	Downregulated
Gm30648	-2.4511480	3.814E-03	Downregulated
Gm2629	-2.4403065	7.128E-03	Downregulated
Rpl3-ps1	-2.4361469	2.970E-02	Downregulated
Gm4489	-2.4327184	4.116E-03	Downregulated
Fosb	-2.4167685	2.116E-02	Downregulated
Atp9a	-2.4138933	2.777E-03	Downregulated
Kank2	-2.4126033	1.236E-02	Downregulated
Serpine2	-2.4125724	1.793E-05	Downregulated
Nlrp1b	-2.4029290	2.412E-02	Downregulated
Gm42869	-2.3893306	4.560E-02	Downregulated
Clgn	-2.3844938	1.819E-02	Downregulated
Tmem254a	-2.3839273	4.065E-03	Downregulated
A930005H10Rik	-2.3837377	4.898E-04	Downregulated
Tlcd3b	-2.3813726	1.392E-03	Downregulated
Spred3	-2.3770876	2.163E-02	Downregulated
Zfp775	-2.3738667	1.269E-03	Downregulated
Kcng2	-2.3719598	8.151E-03	Downregulated
Lbp	-2.2928663	4.016E-02	Downregulated
4930412F12Rik	-2.2786379	1.808E-02	Downregulated
Gm36931	-2.2762640	2.378E-03	Downregulated
Tmie	-2.2754596	2.743E-03	Downregulated
Ncr1	-2.2702806	1.547E-03	Downregulated
Styk1	-2.2623049	1.437E-02	Downregulated
Themis	-2.2521473	2.847E-03	Downregulated

Gm43667	-2.2499474	3.591E-02	Downregulated
Hk1os	-2.2492891	1.566E-02	Downregulated
6230400D17Rik	-2.2469819	2.819E-02	Downregulated
Pvr	-2.2278053	2.496E-03	Downregulated
Ust	-2.2248859	6.140E-04	Downregulated
Eef1a1-ps1	-2.2223630	4.243E-02	Downregulated
Gm42462	-2.1928197	2.544E-02	Downregulated
Lix1l	-2.1813541	4.403E-02	Downregulated
Ptgfrn	-2.1731498	8.909E-05	Downregulated
Trbc1	-2.1396671	1.430E-02	Downregulated
Dpysl3	-2.1388760	1.437E-02	Downregulated
Mir5125	-2.1381291	2.746E-02	Downregulated
1700006J14Rik	-2.1285129	6.265E-03	Downregulated
Cbx2	-2.1267509	4.249E-02	Downregulated
Tdrp	-2.1230601	1.732E-03	Downregulated
Ntrk3	-2.1216760	2.479E-02	Downregulated
4833445I07Rik	-2.1189932	3.094E-02	Downregulated
D630033A02Rik	-2.1173868	3.788E-02	Downregulated
Efna5	-2.1166074	7.596E-03	Downregulated
Pea15a	-2.1163491	1.270E-07	Downregulated
Trbv13-2	-2.0714064	4.346E-02	Downregulated
Plekha7	-2.0702191	7.964E-03	Downregulated
Gm37115	-2.0647449	3.356E-02	Downregulated
Tm4sf19	-2.0624669	2.170E-02	Downregulated
Trbc2	-2.0615791	1.968E-03	Downregulated
Zfp521	-2.0585232	4.862E-03	Downregulated
Klrk1	-2.0571202	4.934E-03	Downregulated
Atf3	-2.0559547	3.133E-02	Downregulated

Raver2	-2.0504863	5.947E-03	Downregulated
Asprv1	-2.0476499	1.788E-02	Downregulated
Myo6	-2.0465788	1.684E-05	Downregulated
Ldhb	-2.0307373	3.764E-02	Downregulated
Lef1	-2.0261132	2.740E-03	Downregulated
Gm38200	-2.0254084	4.295E-02	Downregulated
Klrb1c	-2.0232143	3.281E-02	Downregulated
Ccr5	-2.0153357	4.388E-03	Downregulated
Clstn1	-2.0129456	1.029E-02	Downregulated
Gbp8	-2.0106885	7.633E-03	Downregulated
Sh2d1a	-2.0104294	1.507E-02	Downregulated
Cd5	-2.0067885	1.427E-02	Downregulated
Arl4d	-2.0040712	2.845E-02	Downregulated
Arhgef17	-2.0003844	1.311E-02	Downregulated
Inka2	-1.9792149	4.403E-02	Downregulated
Atp10a	-1.9706273	1.721E-02	Downregulated
Cd4	-1.9357325	7.519E-03	Downregulated
Esr1	-1.9344353	3.170E-02	Downregulated
Vipr1	-1.9262335	1.002E-03	Downregulated
Zfp429	-1.9230597	4.921E-02	Downregulated
Nomo1	-1.9212707	5.152E-04	Downregulated
Dph2	-1.8833263	7.315E-04	Downregulated
Apoe	-1.8817427	1.116E-02	Downregulated
Enc1	-1.8791043	1.356E-03	Downregulated
Lysmd2	-1.8738995	6.455E-03	Downregulated
Cd28	-1.8518578	6.248E-04	Downregulated
St8sia1	-1.8450473	1.567E-02	Downregulated
Tlr12	-1.8403489	4.509E-03	Downregulated

Gpm6b	-1.8391810	3.214E-03	Downregulated
Acpp	-1.8326652	2.803E-02	Downregulated
Lancl3	-1.8299447	1.120E-02	Downregulated
Cnih3	-1.8263133	4.975E-02	Downregulated
Cdh1	-1.8239447	2.493E-02	Downregulated
4930598N05Rik	-1.8238022	5.023E-03	Downregulated
Rpgrip1	-1.8229583	3.813E-02	Downregulated
Ccdc61	-1.8130883	4.072E-03	Downregulated
Sytl2	-1.7960499	1.476E-03	Downregulated
Hpgd	-1.7946428	3.905E-02	Downregulated
6030458C11Rik	-1.7892118	3.843E-02	Downregulated
Pde7b	-1.7811919	3.028E-03	Downregulated
Itm2a	-1.7764981	1.353E-02	Downregulated
Tcf7	-1.7739232	1.046E-02	Downregulated
Nsg2	-1.7721010	1.116E-02	Downregulated
Ar	-1.7634491	4.800E-03	Downregulated
Slc35e4	-1.7562993	3.890E-02	Downregulated
Gm28373	-1.7547174	4.521E-02	Downregulated
Ddr1	-1.7512673	3.664E-02	Downregulated
Tyro3	-1.7288368	4.660E-02	Downregulated
C230085N15Rik	-1.7285875	3.302E-02	Downregulated
Tmbim1	-1.7080198	2.770E-03	Downregulated
Bicdl1	-1.7009174	7.937E-03	Downregulated
Btbd11	-1.6977989	2.358E-02	Downregulated
Tssc4	-1.6944268	2.645E-04	Downregulated
Pcbp4	-1.6924746	2.768E-02	Downregulated
Ccdc32	-1.6777050	1.949E-03	Downregulated
Tle1	-1.6759890	5.550E-03	Downregulated

Hs3st3b1	-1.6730344	2.440E-02	Downregulated
A430033K04Rik	-1.6725290	3.273E-02	Downregulated
Flnb	-1.6564025	4.075E-02	Downregulated
Adgrl1	-1.6481756	1.573E-02	Downregulated
Zfp763	-1.6418815	1.506E-02	Downregulated
Cfp	-1.6367302	2.638E-02	Downregulated
Cd247	-1.6324531	4.533E-02	Downregulated
Tox	-1.6267125	7.505E-03	Downregulated
Intu	-1.6253334	1.059E-02	Downregulated
Faah	-1.6209901	1.315E-03	Downregulated
Lpar6	-1.6170059	1.388E-02	Downregulated
Scamp5	-1.6112876	1.593E-02	Downregulated
Gprin3	-1.6112770	2.012E-02	Downregulated
Wls	-1.6074329	1.043E-02	Downregulated
BC043934	-1.6067162	2.498E-02	Downregulated
Zfp772	-1.5858341	4.745E-02	Downregulated
Gnb4	-1.5813571	1.762E-02	Downregulated
Als2cl	-1.5746275	4.647E-02	Downregulated
Spock2	-1.5717890	4.028E-02	Downregulated
Atp1b1	-1.5651606	5.092E-03	Downregulated
Kremen1	-1.5611153	3.979E-02	Downregulated
Sfn	-1.5540881	2.889E-02	Downregulated
Skap1	-1.5540423	2.426E-02	Downregulated
Sh2d2a	-1.5475824	2.553E-02	Downregulated
Tecpr1	-1.5465552	2.698E-03	Downregulated
Klhl3	-1.5456314	4.719E-02	Downregulated
Peg10	-1.5436062	2.001E-02	Downregulated
Zfp619	-1.5397912	2.366E-02	Downregulated

Impdh1	-1.5335233	1.692E-02	Downregulated
Emb	-1.5327135	3.533E-03	Downregulated
Il7r	-1.5286593	1.688E-02	Downregulated
Gtf2h4	-1.5217634	3.896E-02	Downregulated
Dmac1	-1.5112193	2.239E-02	Downregulated
Magee1	-1.5109354	3.118E-02	Downregulated
Rpusd1	-1.5048764	4.673E-02	Downregulated
Abhd6	-1.4976042	1.521E-02	Downregulated
Gm43387	-1.4961066	4.201E-02	Downregulated
Zfp719	-1.4954260	8.962E-03	Downregulated
Pfdn2	-1.4916724	1.899E-03	Downregulated
2010016I18Rik	-1.4898841	4.500E-02	Downregulated
Sez6l2	-1.4709191	3.526E-02	Downregulated
Cd81	-1.4664781	1.508E-02	Downregulated
Ctsw	-1.4628519	1.288E-02	Downregulated
Hgsnat	-1.4594833	5.771E-03	Downregulated
Zfp354c	-1.4468308	2.186E-02	Downregulated
St8sia6	-1.4366001	2.141E-02	Downregulated
Enah	-1.4156754	4.886E-02	Downregulated
Satb1	-1.4148209	2.569E-02	Downregulated
Plcg1	-1.3978180	1.685E-02	Downregulated
Wdr83os	-1.3873991	2.729E-02	Downregulated
Cryl1	-1.3758634	3.463E-02	Downregulated
Zfp157	-1.3604156	1.064E-02	Downregulated
Fam98c	-1.3404864	1.096E-02	Downregulated
Dlg4	-1.3404643	1.161E-03	Downregulated
Mrpl23	-1.3346217	2.632E-02	Downregulated
Lpin1	-1.3314749	1.547E-02	Downregulated

Ccdc86	-1.3238044	4.702E-02	Downregulated
Akap17b	-1.3143369	3.445E-02	Downregulated
Plekha5	-1.3125561	3.706E-02	Downregulated
Ubap1	-1.3101786	3.039E-02	Downregulated
Bcap31	-1.2954442	3.349E-02	Downregulated
Ms4a4b	-1.2929975	4.041E-02	Downregulated
Aven	-1.2855559	3.977E-02	Downregulated
Saraf	-1.2703557	3.635E-03	Downregulated
D16Ertd472e	-1.2630855	4.745E-03	Downregulated
Rab37	-1.2630770	1.662E-02	Downregulated
Sh3bgrl3	-1.2500329	2.372E-02	Downregulated
Pros1	-1.2482396	4.619E-02	Downregulated
Pdp1	-1.2447345	2.251E-02	Downregulated
Golga3	-1.2386336	4.970E-03	Downregulated
Sidt1	-1.1989929	4.010E-02	Downregulated
Panx1	-1.1593458	3.506E-02	Downregulated
Ergic1	-1.1554457	2.121E-02	Downregulated
As3mt	-1.1550357	2.096E-02	Downregulated
Il6st	-1.1449815	3.796E-02	Downregulated
Phtf2	-1.1093584	4.315E-02	Downregulated
Arl4c	-1.1000025	4.785E-02	Downregulated
Pde3b	-1.0777025	3.466E-02	Downregulated
Laptm5	-1.0210776	2.031E-02	Downregulated
Nrd1	0.9518618	2.947E-02	Upregulated
Nbr1	0.9669436	4.859E-02	Upregulated
Mindy2	0.9964422	4.871E-02	Upregulated
Add1	1.0273154	4.667E-02	Upregulated
Oip5os1	1.0338381	2.327E-02	Upregulated

Agpat4	1.0848780	3.651E-02	Upregulated
Tmcc2	1.0915244	4.654E-02	Upregulated
Fbxo3	1.0955160	3.136E-02	Upregulated
Abhd16a	1.1013284	7.308E-03	Upregulated
Hipk2	1.1082268	2.751E-02	Upregulated
Hbq1b	1.1274358	1.901E-02	Upregulated
Atp6v0d1	1.1431066	3.141E-02	Upregulated
Lmo2	1.1449616	1.927E-02	Upregulated
Wwp2	1.1488290	1.514E-02	Upregulated
Marchf8	1.1512643	4.416E-02	Upregulated
Psip1	1.1578116	2.796E-02	Upregulated
Nars	1.1632340	1.645E-02	Upregulated
Pold1	1.1672101	3.367E-02	Upregulated
Carhsp1	1.1776237	2.122E-02	Upregulated
Hras	1.1790537	2.229E-02	Upregulated
Napa	1.1930188	1.361E-02	Upregulated
Acsl1	1.2053400	4.950E-02	Upregulated
Rex1bd	1.2114361	3.641E-02	Upregulated
Rnasel	1.2148972	1.992E-02	Upregulated
Pck2	1.2460777	3.616E-02	Upregulated
Mki67	1.2546725	4.525E-02	Upregulated
Ahi1	1.2633362	1.846E-02	Upregulated
Vwa5a	1.2646412	5.493E-03	Upregulated
Ptpa	1.2748317	7.372E-03	Upregulated
Dop1b	1.2936455	1.920E-02	Upregulated
2500002B13Rik	1.3004423	3.806E-03	Upregulated
Tceal8	1.3061985	2.268E-02	Upregulated
Fam160b1	1.3076065	3.090E-03	Upregulated

Tsen15	1.3086782	4.451E-03	Upregulated
Bag2	1.3133127	3.332E-02	Upregulated
Fn3krp	1.3141480	3.376E-03	Upregulated
Exosc9	1.3212016	3.861E-03	Upregulated
Pnpo	1.3243258	4.846E-02	Upregulated
Pyurf	1.3316482	3.479E-02	Upregulated
Add2	1.3353350	4.262E-02	Upregulated
Nt5c3	1.3358367	4.515E-02	Upregulated
Acp1	1.3415011	2.215E-02	Upregulated
Hectd4	1.3435751	2.837E-02	Upregulated
Micos13	1.3504283	4.535E-02	Upregulated
Ranbp10	1.3506783	8.612E-03	Upregulated
Asf1b	1.3563935	3.047E-02	Upregulated
Bcas2	1.3608203	2.972E-02	Upregulated
Hscb	1.3624131	3.681E-02	Upregulated
Pitrm1	1.3631588	8.496E-03	Upregulated
Tgm2	1.3683973	3.878E-02	Upregulated
Cbr1	1.3731931	4.747E-02	Upregulated
Psmd10	1.3767266	7.871E-03	Upregulated
Rnf123	1.3794789	1.261E-02	Upregulated
Ilrun	1.3886169	9.692E-03	Upregulated
Polr2c	1.3886957	9.725E-03	Upregulated
Tspan33	1.3892785	6.932E-03	Upregulated
Nipa1	1.3904591	6.511E-03	Upregulated
Mettl27	1.3904986	2.024E-02	Upregulated
2310022B05Rik	1.3955498	1.840E-03	Upregulated
Hebp1	1.4128982	3.100E-02	Upregulated
Samd4	1.4129602	9.301E-03	Upregulated

Dmtn	1.4150140	1.593E-02	Upregulated
H2bc22	1.4207151	4.949E-02	Upregulated
Dnajb2	1.4209189	5.294E-03	Upregulated
Tjp1	1.4223962	5.402E-03	Upregulated
Aim2	1.4239481	9.828E-03	Upregulated
Tusc1	1.4243858	3.248E-02	Upregulated
Timm8b	1.4273453	4.598E-02	Upregulated
Snx32	1.4282268	2.750E-02	Upregulated
Zfpm1	1.4325627	2.493E-02	Upregulated
Mcm10	1.4326439	2.540E-02	Upregulated
Ank1	1.4342815	2.142E-02	Upregulated
Cars	1.4411366	2.278E-02	Upregulated
Palb2	1.4593902	4.989E-02	Upregulated
Adk	1.4607024	7.235E-03	Upregulated
Foxm1	1.4652861	3.371E-02	Upregulated
Btnl10	1.4711599	3.772E-02	Upregulated
Fgfr1op2	1.4722544	2.015E-02	Upregulated
Ntn4	1.4770038	2.455E-02	Upregulated
Kdr	1.4773039	2.687E-02	Upregulated
Plscr3	1.4883612	4.761E-02	Upregulated
Ppp2r5b	1.4974471	3.639E-02	Upregulated
Fahd1	1.5038834	2.331E-02	Upregulated
Dele1	1.5080481	8.383E-05	Upregulated
Nectin1	1.5158633	4.473E-02	Upregulated
Mgll	1.5204386	2.583E-03	Upregulated
G3bp2	1.5266906	2.265E-05	Upregulated
Ifrd2	1.5269713	4.331E-03	Upregulated
Slc4a1	1.5334059	1.660E-02	Upregulated

Pkd1l1	1.5341683	1.875E-02	Upregulated
Usp46	1.5553532	2.795E-02	Upregulated
Cdk8	1.5570293	3.578E-02	Upregulated
Cmas	1.5585958	1.550E-02	Upregulated
Txnrd2	1.5630226	2.626E-02	Upregulated
Mpc2	1.5666237	4.320E-02	Upregulated
Ndufb4	1.5671980	1.736E-02	Upregulated
E2f1	1.5684085	5.013E-03	Upregulated
Tmod1	1.5717287	5.052E-03	Upregulated
Nusap1	1.5895352	7.246E-03	Upregulated
Gm8189	1.5899186	3.270E-02	Upregulated
Tlr7	1.6054537	3.833E-02	Upregulated
Atg4a	1.6238463	3.667E-02	Upregulated
Rab3il1	1.6254098	1.465E-02	Upregulated
Rapgefl1	1.6276921	1.906E-02	Upregulated
7SK	1.6317919	4.607E-02	Upregulated
Fam214b	1.6363643	7.618E-04	Upregulated
Slc6a20a	1.6618233	2.116E-02	Upregulated
Etl4	1.6682879	4.798E-02	Upregulated
Sh3bp4	1.6718718	3.129E-02	Upregulated
Dnajc12	1.6725305	3.068E-02	Upregulated
Rundc3a	1.6739149	7.484E-03	Upregulated
Gcnt1	1.6759391	7.030E-03	Upregulated
Slc12a2	1.6859510	1.522E-02	Upregulated
Pink1	1.6915873	8.122E-04	Upregulated
Stk11	1.6980266	6.101E-03	Upregulated
Bhlha15	1.7027526	3.671E-02	Upregulated
Carnmt1	1.7031785	4.866E-03	Upregulated

Chp1	1.7035329	9.799E-04	Upregulated
Cript	1.7041280	4.847E-06	Upregulated
Bckdhb	1.7057029	3.796E-03	Upregulated
AW011738	1.7107526	3.395E-05	Upregulated
Ephb4	1.7149644	4.249E-02	Upregulated
Auh	1.7182614	2.616E-06	Upregulated
Mrpl20	1.7183127	1.148E-02	Upregulated
9830144P21Rik	1.7192077	2.865E-02	Upregulated
Reep6	1.7221193	3.618E-02	Upregulated
Ak7	1.7345712	2.989E-02	Upregulated
Slc25a42	1.7435171	3.948E-03	Upregulated
Pak4	1.7525515	3.615E-04	Upregulated
Vaultrc5	1.7534932	3.722E-02	Upregulated
Slit1	1.7546816	2.180E-02	Upregulated
Ndufa7	1.7561112	2.432E-02	Upregulated
Crat	1.7567762	4.343E-05	Upregulated
Rcl1	1.7580230	1.415E-02	Upregulated
Gm11280	1.7602933	3.829E-02	Upregulated
Psrc1	1.7741816	3.730E-03	Upregulated
Cox7a2	1.7745769	1.951E-02	Upregulated
Snx22	1.7770493	7.212E-04	Upregulated
Rmc1	1.8052900	4.556E-04	Upregulated
Ret	1.8066799	4.956E-03	Upregulated
Pparg	1.8275482	1.515E-02	Upregulated
5_8S_rRNA	1.8366935	4.457E-02	Upregulated
Atp5e	1.8498202	9.745E-03	Upregulated
Acacb	1.8673159	3.609E-02	Upregulated
Trim10	1.8707171	1.656E-02	Upregulated

Sptb	1.8756109	1.425E-05	Upregulated
Gngt2	1.8813865	3.006E-02	Upregulated
H2bu2	1.8863451	8.135E-04	Upregulated
Rn7s6	1.8882267	2.217E-03	Upregulated
Gm5148	1.9021773	2.422E-02	Upregulated
1810053B23Rik	1.9123540	3.865E-02	Upregulated
Tmem91	1.9197723	3.610E-02	Upregulated
Gm26461	1.9387484	1.202E-03	Upregulated
Fam13a	1.9423074	1.720E-02	Upregulated
Pet100	1.9446551	1.362E-02	Upregulated
mmu-mir-1194	1.9458975	1.876E-02	Upregulated
Vash1	1.9462421	1.349E-02	Upregulated
Xndc1	1.9466435	1.449E-03	Upregulated
Rph3al	1.9764802	7.870E-04	Upregulated
Fam161b	1.9852244	2.241E-02	Upregulated
Gm37194	1.9866720	5.614E-03	Upregulated
Mindy4	2.0113368	1.999E-02	Upregulated
Rhobtb1	2.0237167	8.913E-03	Upregulated
Sv2c	2.0249114	1.853E-02	Upregulated
Pih1d2	2.0332744	1.358E-02	Upregulated
Gm14305	2.0360182	4.437E-02	Upregulated
Gm20632	2.0361147	1.333E-02	Upregulated
A930006K02Rik	2.0478818	6.225E-03	Upregulated
Kcns2	2.0489545	7.785E-03	Upregulated
Rn7s1	2.0508825	2.448E-03	Upregulated
Alad	2.0517535	1.508E-03	Upregulated
Gls2	2.0527938	3.256E-02	Upregulated
Rad51	2.0595408	1.313E-03	Upregulated

Gm43627	2.0624165	3.398E-02	Upregulated
Amd2	2.0659538	2.495E-02	Upregulated
Gm49420	2.0750524	2.762E-03	Upregulated
Mpp2	2.0813885	9.869E-06	Upregulated
Gm13375	2.0910255	1.002E-02	Upregulated
Septin10	2.0928750	7.467E-03	Upregulated
Bbs7	2.0936139	7.662E-04	Upregulated
Rn18s-rs5	2.0980860	1.665E-02	Upregulated
A630072L19Rik	2.0988390	2.426E-03	Upregulated
Satb2	2.1050569	3.143E-02	Upregulated
Obscn	2.1086432	1.046E-02	Upregulated
Ctsf	2.1090220	2.090E-03	Upregulated
Nr1i2	2.1122393	5.309E-03	Upregulated
ENSMUSG00002075501	2.1122543	3.654E-03	Upregulated
Cyp4b1-ps2	2.1171743	3.143E-02	Upregulated
Narf	2.1180634	9.499E-09	Upregulated
Tbr1	2.1247780	3.022E-02	Upregulated
ENSMUSG00002075647	2.1346562	3.155E-02	Upregulated
Glrx	2.1445597	7.980E-03	Upregulated
Col4a1	2.1507557	4.457E-02	Upregulated
Ston1	2.1577452	1.069E-02	Upregulated
Pcx	2.1629204	3.944E-06	Upregulated
Aarsd1	2.1644500	6.530E-03	Upregulated
Mir451a	2.1710126	4.365E-03	Upregulated
Ube2e2	2.1806997	1.773E-02	Upregulated
Icam5	2.1837416	2.199E-02	Upregulated
Sh2d1b1	2.1868365	4.803E-02	Upregulated
Asns	2.1885469	9.484E-03	Upregulated

Ctcflos	2.1907442	1.379E-02	Upregulated
Phyhip	2.1979258	8.801E-03	Upregulated
Rn7s2	2.2049477	2.331E-03	Upregulated
Gm48161	2.2189897	2.781E-02	Upregulated
Zfp982	2.2328012	4.139E-02	Upregulated
Gm11973	2.2415023	1.600E-02	Upregulated
Serinc3	2.2421048	4.697E-05	Upregulated
Gm16793	2.2444163	8.209E-03	Upregulated
Arhgef40	2.2505170	8.036E-04	Upregulated
Tm6sf2	2.2962588	4.938E-02	Upregulated
9830132P13Rik	2.3014717	3.302E-02	Upregulated
Gm867	2.3287364	1.141E-03	Upregulated
Rnf26	2.3404822	5.398E-05	Upregulated
Calr3	2.3483335	4.708E-02	Upregulated
Gm5815	2.3632396	2.841E-02	Upregulated
Serpine1	2.3664829	4.523E-02	Upregulated
Syt14	2.4063100	1.383E-05	Upregulated
Bub1b	2.4167111	2.302E-03	Upregulated
BC065403	2.4249447	3.631E-02	Upregulated
4933431K14Rik	2.4571734	8.824E-03	Upregulated
5_8S_rRNA	2.4677718	2.555E-03	Upregulated
Sytl5	2.4732683	4.174E-02	Upregulated
Gm9522	2.4740714	3.579E-02	Upregulated
ENSMUSG00002076173	2.4783363	6.050E-03	Upregulated
Snrnp25	2.5024330	1.256E-05	Upregulated
Pycr1	2.5127522	1.155E-03	Upregulated
Apol10b	2.5355904	2.465E-02	Upregulated
Lockd	2.5410373	3.241E-04	Upregulated

Tmem17	2.5722748	2.963E-02	Upregulated
ENSMUSG00002075188	2.5772766	1.132E-03	Upregulated
Acat3	2.5831874	2.334E-02	Upregulated
H2bu1-ps	2.5838385	6.987E-03	Upregulated
Myo5c	2.6155572	4.092E-02	Upregulated
Gpi-ps	2.6463700	3.340E-02	Upregulated
Plxnb3	2.6550075	4.506E-03	Upregulated
Gm37265	2.6730945	3.746E-02	Upregulated
Gm18180	2.6851580	2.191E-02	Upregulated
6330549D23Rik	2.6866383	4.288E-02	Upregulated
Bmp4	2.6920701	9.545E-03	Upregulated
ENSMUSG00002075551	2.6996422	3.297E-03	Upregulated
Rnf165	2.7007424	1.474E-02	Upregulated
Cdh5	2.7174500	5.994E-03	Upregulated
Gm38381	2.7406753	7.482E-03	Upregulated
Josd2	2.7473046	1.162E-09	Upregulated
Gm47171	2.7602862	8.226E-03	Upregulated
Scart1	2.7651772	4.200E-02	Upregulated
Exph5	2.7699471	6.611E-03	Upregulated
Rims2	2.7712704	6.805E-04	Upregulated
Hoxb3os	2.7735062	1.937E-02	Upregulated
Rtn2	2.7844144	3.209E-02	Upregulated
Gm25813	2.7888099	1.767E-02	Upregulated
Gm40578	2.7900670	4.353E-03	Upregulated
Apol11b	2.7902618	1.117E-06	Upregulated
Gm15564	2.7994430	3.476E-03	Upregulated
Mgat3	2.8456877	4.376E-02	Upregulated
Srl	2.8550617	7.426E-03	Upregulated

Gm43751	2.8656668	3.712E-03	Upregulated
Gm29724	2.9047280	4.153E-03	Upregulated
Nhlrc4	2.9058849	5.558E-04	Upregulated
Ppp1r3c	2.9367083	1.267E-03	Upregulated
Gm8113	2.9837299	2.538E-02	Upregulated
Gm45315	3.0018319	1.182E-02	Upregulated
Vmn2r96	3.0073936	1.892E-02	Upregulated
ENSMUSG00002075524	3.0091735	1.922E-03	Upregulated
Plekhh2	3.0134214	8.065E-03	Upregulated
6330403K07Rik	3.0292517	7.541E-03	Upregulated
Rgs22	3.0646618	3.190E-02	Upregulated
Wasf1	3.0744689	3.437E-05	Upregulated
Ccl22	3.0809497	1.644E-02	Upregulated
6530409C15Rik	3.0820342	2.185E-02	Upregulated
Klk1b22	3.0976511	3.565E-02	Upregulated
Cerox1	3.1044234	2.318E-02	Upregulated
3300005D01Rik	3.1056370	4.237E-02	Upregulated
Gm11223	3.1565551	2.474E-02	Upregulated
4931422A03Rik	3.1821855	2.537E-02	Upregulated
Ppfia2	3.2168734	4.326E-02	Upregulated
Dab1	3.2199890	4.275E-02	Upregulated
Dscaml1	3.2214916	4.358E-02	Upregulated
Gm16213	3.2291243	3.400E-02	Upregulated
Zfp449	3.2447658	2.320E-02	Upregulated
Gm7897	3.2492056	1.670E-02	Upregulated
Mir6236	3.2538377	6.914E-04	Upregulated
Lars2	3.2852333	1.279E-03	Upregulated
Ighg2c	3.2933623	4.121E-02	Upregulated

Elavl2	3.3277672	7.536E-04	Upregulated
Nacad	3.3441169	2.011E-02	Upregulated
Vgf	3.3645310	4.749E-02	Upregulated
Trpm6	3.3664375	2.391E-02	Upregulated
Gpr6	3.3675221	4.624E-02	Upregulated
Hapln2	3.3687351	4.574E-02	Upregulated
A830018L16Rik	3.3711862	4.476E-02	Upregulated
Oprd1	3.3712485	4.622E-02	Upregulated
Iqsec3	3.3723679	4.429E-02	Upregulated
Lgi2	3.3726853	4.440E-02	Upregulated
Ntsr1	3.3728913	4.701E-02	Upregulated
Hdx	3.3728938	4.483E-02	Upregulated
Necab2	3.3733512	4.444E-02	Upregulated
Cdh10	3.3734300	4.438E-02	Upregulated
Rab15	3.3736750	4.417E-02	Upregulated
Ppp4r4	3.3739498	4.395E-02	Upregulated
Masp1	3.3745040	4.471E-02	Upregulated
Lrfn5	3.3747770	4.391E-02	Upregulated
Nap1l2	3.3747822	4.392E-02	Upregulated
Lrp1b	3.3748916	4.391E-02	Upregulated
Gpld1	3.3749001	4.423E-02	Upregulated
Kndc1	3.3749077	4.426E-02	Upregulated
Rnf208	3.3749139	4.427E-02	Upregulated
Pcsk2	3.3749462	4.436E-02	Upregulated
Cdh12	3.3750664	4.468E-02	Upregulated
Cckbr	3.3750867	4.473E-02	Upregulated
Negr1	3.3751821	4.384E-02	Upregulated
Zfp423	3.3752085	4.443E-02	Upregulated

Mab21l1	3.3753193	4.539E-02	Upregulated
Gm48822	3.3761882	4.811E-02	Upregulated
Gm5067	3.3762468	4.832E-02	Upregulated
4833412C15Rik	3.3772965	4.824E-02	Upregulated
Sat2	3.3827546	4.408E-02	Upregulated
Gm45274	3.4008467	2.361E-03	Upregulated
Nptxr	3.4094793	2.839E-02	Upregulated
Tmem63c	3.4114878	2.828E-02	Upregulated
Mycn	3.4117507	3.038E-02	Upregulated
4933412O06Rik	3.4209407	4.755E-02	Upregulated
Kirrel3os	3.4306249	3.242E-02	Upregulated
Apol10a	3.4324303	4.605E-04	Upregulated
Slc15a2	3.4469647	8.080E-06	Upregulated
Gm43909	3.4777276	2.298E-02	Upregulated
Hsd3b6	3.4893775	1.271E-02	Upregulated
Azin2	3.5292648	1.014E-02	Upregulated
Itgad	3.5368467	9.629E-04	Upregulated
Gm48684	3.5488238	4.369E-02	Upregulated
Gm45104	3.5658833	5.417E-03	Upregulated
Pkia	3.5753033	1.969E-02	Upregulated
Prkar1b	3.5789366	1.917E-02	Upregulated
Atcay	3.5808392	1.902E-02	Upregulated
Gm29514	3.5943579	2.677E-02	Upregulated
Mir144; miRNA	3.5943895	5.465E-03	Upregulated
Gm29340	3.5944960	3.058E-02	Upregulated
Hey2	3.5951689	2.034E-02	Upregulated
Spire2	3.5992416	2.009E-02	Upregulated
B930036N10Rik	3.6396228	1.714E-02	Upregulated

9430087J23Rik	3.6500584	3.144E-04	Upregulated
Gm45151	3.6549365	4.733E-02	Upregulated
Spag8	3.6640511	3.898E-02	Upregulated
Gm19196	3.6656313	4.524E-02	Upregulated
Gm34237	3.6807916	2.393E-02	Upregulated
Mei1	3.6855912	2.566E-02	Upregulated
Tacr3	3.6875319	2.361E-02	Upregulated
Mtag2	3.6879174	2.575E-02	Upregulated
7SK	3.6918156	2.526E-02	Upregulated
Apol10c-ps	3.6932762	3.112E-04	Upregulated
Klhdc8a	3.6950262	2.149E-02	Upregulated
Kif6	3.6953004	2.430E-02	Upregulated
Nova1	3.6954030	2.099E-02	Upregulated
Ndst4	3.6965455	2.148E-02	Upregulated
Lin7a	3.6967360	2.081E-02	Upregulated
Gria4	3.6967672	2.068E-02	Upregulated
Gm26814	3.6973852	2.173E-02	Upregulated
Slc6a8	3.6976505	2.066E-02	Upregulated
Hhatl	3.6985322	2.192E-02	Upregulated
Cttnbp2	3.6990905	2.106E-02	Upregulated
D930036K23Rik	3.6995103	2.268E-02	Upregulated
Npsr1	3.7002331	2.801E-02	Upregulated
Tmem74	3.7005134	2.074E-02	Upregulated
Syngr3	3.7016087	2.131E-02	Upregulated
Gm8615	3.7020786	3.916E-02	Upregulated
6030407O03Rik	3.7024024	4.709E-02	Upregulated
Gal3st1	3.7060710	2.089E-02	Upregulated
Krt77	3.7080566	2.146E-02	Upregulated

Lmcd1	3.7092348	2.222E-02	Upregulated
Fam107a	3.7147997	2.165E-02	Upregulated
Etnk2	3.7201999	2.273E-02	Upregulated
Scara3	3.7280963	1.391E-02	Upregulated
Apol11a	3.7333224	1.349E-07	Upregulated
Ccn2	3.7339425	1.359E-02	Upregulated
Kcnq4	3.7381726	1.523E-02	Upregulated
Iqub	3.7394445	1.693E-02	Upregulated
Lypd1	3.7408188	4.542E-02	Upregulated
Ankrd36	3.8117457	1.864E-02	Upregulated
A330094K24Rik	3.8307114	3.664E-02	Upregulated
Gm43566	3.8617343	3.183E-02	Upregulated
Gm6278	3.8647109	5.525E-04	Upregulated
Cdcp1	3.8812634	1.694E-02	Upregulated
Robo4	3.8989076	2.016E-02	Upregulated
Gm11653	3.9206297	1.573E-02	Upregulated
Gm47438	3.9220975	4.134E-02	Upregulated
Gm26797	3.9393931	4.519E-02	Upregulated
Zfp879	3.9553611	1.188E-02	Upregulated
Sfrp1	3.9556241	1.090E-02	Upregulated
Chga	3.9556300	1.130E-02	Upregulated
Insm1	3.9565102	1.156E-02	Upregulated
Arxes2	3.9577852	1.089E-02	Upregulated
Ptgs2	3.9583875	2.113E-02	Upregulated
Tunar	3.9586069	1.068E-02	Upregulated
Snap91	3.9598131	1.050E-02	Upregulated
Cbx3-ps6	3.9612437	4.016E-02	Upregulated
Gm37363	3.9615496	1.207E-02	Upregulated

St8sia3	3.9626020	1.048E-02	Upregulated
Asic4	3.9644811	1.181E-02	Upregulated
Depp1	3.9652657	3.398E-02	Upregulated
Gdnf	3.9657250	3.616E-02	Upregulated
Gm42899	3.9658340	1.172E-02	Upregulated
Gm44848	3.9695695	1.268E-02	Upregulated
Tmem88b	3.9717712	1.591E-03	Upregulated
Il17re	3.9935046	4.587E-02	Upregulated
Gm6344	4.0091671	4.227E-02	Upregulated
Pde6a	4.0181948	3.177E-02	Upregulated
9630001P10Rik	4.0238144	1.387E-02	Upregulated
Gm6937	4.0348685	3.922E-02	Upregulated
Gm12846	4.0399428	1.708E-02	Upregulated
Gm20449	4.0916387	3.315E-02	Upregulated
Commd5	4.0990294	3.547E-02	Upregulated
Gm45737	4.1033258	2.110E-02	Upregulated
Ttll9	4.1303834	9.988E-03	Upregulated
Gm15956	4.1527989	3.603E-02	Upregulated
Ighv5-16	4.1566228	2.475E-02	Upregulated
Gm38026	4.1587562	3.831E-02	Upregulated
Gm32950	4.1704928	3.426E-02	Upregulated
Ntn5	4.1714666	7.692E-03	Upregulated
Gm49069	4.1754012	6.452E-03	Upregulated
Gm37818	4.1754092	8.644E-03	Upregulated
Pcdh9	4.1821407	5.662E-03	Upregulated
Lrtm2	4.1875675	5.722E-03	Upregulated
Hjv	4.1876581	7.996E-03	Upregulated
F830115B05Rik	4.1877023	1.257E-02	Upregulated

Oas1e	4.1963982	1.358E-04	Upregulated
Gm13832	4.2013428	3.899E-02	Upregulated
Gm16439	4.2081789	3.410E-02	Upregulated
Spink10	4.2190466	1.006E-02	Upregulated
Rgs7	4.2191110	3.428E-03	Upregulated
Gm15206	4.2903885	4.759E-02	Upregulated
Klhl10	4.3139488	3.944E-02	Upregulated
Gm10516	4.3244402	2.915E-03	Upregulated
Rbp4	4.3456406	4.982E-03	Upregulated
Chrna2	4.3573036	3.519E-02	Upregulated
Etv4	4.3765093	3.653E-03	Upregulated
Pld6	4.3858157	6.880E-03	Upregulated
Gm14086	4.3884720	4.454E-02	Upregulated
Olfr374	4.3957814	4.324E-02	Upregulated
Hsd17b2	4.4003805	4.105E-02	Upregulated
Rpe65	4.4278949	1.171E-02	Upregulated
Adamts13	4.4301044	3.885E-02	Upregulated
Lipg	4.4466991	2.920E-02	Upregulated
Alx4	4.4610206	7.434E-03	Upregulated
Gm8423	4.4761497	4.491E-02	Upregulated
Grin3b	4.4934127	2.030E-02	Upregulated
Wnt6	4.5038351	1.723E-02	Upregulated
Mir6939	4.5040116	4.306E-02	Upregulated
Edn1	4.5382039	1.154E-02	Upregulated
Gm26756	4.5406257	7.075E-03	Upregulated
Kcnk2	4.5423715	1.993E-03	Upregulated
Ppp1r26	4.5454509	1.907E-03	Upregulated
Chil5	4.5498171	1.383E-02	Upregulated

Vamp9	4.5813080	1.572E-02	Upregulated
Kcnq1	4.5909725	4.440E-02	Upregulated
Loxhd1	4.5969922	1.946E-02	Upregulated
Ebf2	4.6154690	2.545E-03	Upregulated
Tmco5	4.6456296	5.143E-03	Upregulated
Rsph6a	4.6691497	1.175E-02	Upregulated
Slc17a7	4.6960835	1.175E-03	Upregulated
Gucy2g	4.6983503	1.828E-03	Upregulated
Tspan11	4.7010622	1.550E-03	Upregulated
Gm37833	4.7359978	4.753E-02	Upregulated
Gm49192	4.7508144	7.145E-03	Upregulated
4933424M12Rik	4.7527244	4.109E-02	Upregulated
Gm37827	4.7538096	8.528E-03	Upregulated
Pln	4.8033887	2.927E-03	Upregulated
Gjb2	4.8110845	1.393E-02	Upregulated
Gm23330	4.8282585	6.341E-03	Upregulated
Gm35101	4.9219111	3.062E-02	Upregulated
Gm9530	4.9323960	6.341E-03	Upregulated
Gm47175	4.9522861	1.145E-02	Upregulated
Cilp	4.9648023	3.093E-02	Upregulated
Prok2	4.9768343	2.294E-02	Upregulated
Gm16343	5.1127910	4.687E-02	Upregulated
Kat2b-ps	5.1266261	1.051E-02	Upregulated
Slc13a4	5.1821292	3.978E-04	Upregulated
Copz2	5.1886589	2.692E-04	Upregulated
Gm18963	5.2658864	2.930E-02	Upregulated
Gm8730	5.3311024	6.394E-08	Upregulated
Gm47493	5.3707647	5.228E-04	Upregulated

Gm9353	5.3718445	1.901E-03	Upregulated
Gm7324	5.4429254	1.838E-04	Upregulated
Scnn1g	5.4624181	7.875E-03	Upregulated
Ncan	5.5327557	6.036E-05	Upregulated
Gm49497	5.6224619	4.019E-02	Upregulated
mt-Th	5.6726434	4.082E-02	Upregulated
A730098A19Rik	5.7284304	4.841E-04	Upregulated
Nme8	5.9087627	1.225E-02	Upregulated
Gm33100	5.9416878	1.305E-02	Upregulated
Gm9625	6.0079103	5.530E-08	Upregulated
Cxcl14	6.1875575	4.527E-06	Upregulated
Erfe	6.3658256	5.795E-04	Upregulated
Gm14165	7.1700416	4.860E-08	Upregulated

Table of Contents Top of Current Table

Supplemental Table 22. List of all significant (P < 0.05) differentially expressed genes in whole blood of 3xTg-AD CBD (week 8) versus 3xTg-AD vehicle (week 8) animals; 134 genes downregulated & 337 genes are upregulated.

Gene Name	Log2FoldChange	P-value	Regulation
Gm49980	-17.8413797	2.919E-10	Downregulated
Gm14863	-4.5753081	1.637E-02	Downregulated
Gm45148	-4.3168841	2.791E-04	Downregulated
Gm33474	-4.2902737	3.409E-02	Downregulated
Gm20432	-4.1041931	3.269E-03	Downregulated
Gm7962	-4.0939952	1.688E-02	Downregulated
Cabcoco1	-4.0334482	2.262E-03	Downregulated
Slc13a4	-3.9468626	1.302E-03	Downregulated
Fam217a	-3.8254733	2.188E-02	Downregulated
Gm12846	-3.7664702	7.346E-03	Downregulated
Btnl7-ps	-3.7261127	2.514E-02	Downregulated
Ncan	-3.7204114	1.371E-03	Downregulated
Scn2b	-3.7005254	1.247E-03	Downregulated
Scnn1g	-3.6517971	4.533E-02	Downregulated
Clstn3	-3.6514794	9.157E-04	Downregulated
ENSMUSG00002075075	-3.6373219	4.235E-03	Downregulated
Gm37844	-3.6277253	9.710E-03	Downregulated
Lrfn1	-3.5660539	6.222E-03	Downregulated
Gm43728	-3.5542419	2.661E-02	Downregulated
Gm43909	-3.5493871	9.661E-03	Downregulated
Fam71f2	-3.5469885	3.420E-02	Downregulated
Gm49894	-3.5415331	1.370E-02	Downregulated
5830487J09Rik	-3.4874874	3.467E-03	Downregulated
Chmp4c	-3.4777803	4.630E-02	Downregulated
Psd	-3.4686633	3.475E-03	Downregulated

Tspan11	-3.4657943	6.306E-03	Downregulated
Mgat3	-3.4618251	4.919E-03	Downregulated
Zfp612	-3.4603082	4.947E-03	Downregulated
Chst7	-3.4473963	5.875E-03	Downregulated
Spata6l	-3.4384423	9.770E-03	Downregulated
Meiob	-3.4364433	3.813E-02	Downregulated
5S_rRNA	-3.4350813	3.619E-02	Downregulated
Rsph6a	-3.4338807	3.455E-02	Downregulated
Gm5909	-3.3894055	6.623E-03	Downregulated
Gm15853	-3.3667907	1.246E-02	Downregulated
Cldn10	-3.3347605	8.479E-03	Downregulated
Gm12037	-3.3308180	4.915E-02	Downregulated
Fgfr3	-3.3177819	8.444E-03	Downregulated
Flrt3	-3.3116171	8.457E-03	Downregulated
Kcnk12	-3.3112385	9.156E-03	Downregulated
Kcnk2	-3.3071059	8.778E-03	Downregulated
Prkar1b	-3.3054679	8.701E-03	Downregulated
Slco2b1	-3.2998568	9.215E-03	Downregulated
Elovl4	-3.2871207	2.369E-02	Downregulated
Rsph14	-3.2864766	4.976E-02	Downregulated
Sema3g	-3.2780821	1.296E-02	Downregulated
Jazf1	-3.2776471	9.565E-03	Downregulated
Cpeb1	-3.2663919	7.177E-03	Downregulated
Tmod2	-3.2626344	7.279E-03	Downregulated
Antxr1	-3.2522283	4.383E-03	Downregulated
Gm40332	-3.2484390	3.909E-02	Downregulated
Gm14681	-3.2429243	2.620E-02	Downregulated
Gm22571	-3.2045897	3.524E-02	Downregulated

Syt16	-3.1457800	1.469E-02	Downregulated
Chadl	-3.1406757	1.572E-02	Downregulated
Cdk18	-3.1386318	1.557E-02	Downregulated
Mycn	-3.1382787	1.657E-02	Downregulated
Tmem63c	-3.1380173	1.504E-02	Downregulated
Nr4a2	-3.1367931	1.548E-02	Downregulated
Map7d2	-3.1305244	2.601E-02	Downregulated
Scn7a	-3.1178284	2.060E-02	Downregulated
Prokr2	-3.1158941	3.049E-02	Downregulated
A930035D04Rik	-3.1037569	4.932E-02	Downregulated
C8g	-3.0971322	3.620E-02	Downregulated
Gm10827	-3.0557716	2.804E-02	Downregulated
Arl11	-3.0360356	2.214E-02	Downregulated
Aox4	-2.9887179	3.743E-02	Downregulated
Tmem88b	-2.9811584	4.910E-03	Downregulated
Otoa	-2.9747740	4.300E-02	Downregulated
Ak9	-2.9703201	3.592E-02	Downregulated
Gm20732	-2.9698738	2.572E-02	Downregulated
Aldob	-2.9655387	3.351E-02	Downregulated
Spats2l	-2.9591371	3.908E-02	Downregulated
Gm31332	-2.9551051	4.476E-02	Downregulated
Dscaml1	-2.9480213	2.730E-02	Downregulated
Fam135b	-2.9469715	2.652E-02	Downregulated
Dab1	-2.9465193	2.663E-02	Downregulated
Coro2b	-2.9451599	2.699E-02	Downregulated
Cdh22	-2.9418896	2.777E-02	Downregulated
Gm37818	-2.9401412	3.657E-02	Downregulated
Gm49069	-2.9401353	2.944E-02	Downregulated

Gm13205	-2.9319277	3.301E-02	Downregulated
Gm38241	-2.9302729	3.201E-02	Downregulated
Hic1	-2.9091402	4.434E-02	Downregulated
Gm23205	-2.8963578	3.005E-02	Downregulated
Ttll9	-2.8951152	4.194E-02	Downregulated
Gucy2g	-2.8857747	3.176E-02	Downregulated
Adgrl3	-2.8840705	4.609E-02	Downregulated
Gm46210	-2.8481202	1.846E-02	Downregulated
Gm37953	-2.7828923	4.329E-02	Downregulated
Hey2	-2.7445271	4.035E-02	Downregulated
Kcng3	-2.7337651	4.936E-02	Downregulated
Osbpl10	-2.7308380	3.844E-02	Downregulated
Slc8a2	-2.7304402	4.901E-02	Downregulated
Gm50433	-2.7296686	3.233E-02	Downregulated
Bmp6	-2.7264891	4.012E-02	Downregulated
Kcnc2	-2.7237814	4.979E-02	Downregulated
Gpr85	-2.7233087	4.977E-02	Downregulated
Lrp11	-2.7229612	4.968E-02	Downregulated
Plekhf1	-2.7146240	4.992E-02	Downregulated
Ankef1	-2.7099916	4.584E-02	Downregulated
Plekhh2	-2.6033618	2.052E-02	Downregulated
Cerox1	-2.5925311	3.853E-02	Downregulated
AI854703	-2.5901904	1.111E-02	Downregulated
Slc26a10	-2.5774134	3.683E-02	Downregulated
Nos3	-2.5627039	4.131E-02	Downregulated
Phf24	-2.5578766	2.353E-03	Downregulated
Gm42878	-2.5456099	1.815E-02	Downregulated
St6galnac5	-2.5361890	2.536E-02	Downregulated

Myadml2	-2.5177632	7.453E-03	Downregulated
Gm6560	-2.4749486	1.787E-02	Downregulated
Ston1	-2.4122112	6.219E-03	Downregulated
Pwwp2b	-2.3961613	1.890E-02	Downregulated
Pdlim4	-2.2973684	2.490E-02	Downregulated
Elavl2	-2.2596352	1.499E-02	Downregulated
Gm47121	-2.2499997	3.372E-02	Downregulated
Zfp462	-2.2426242	3.903E-02	Downregulated
6330403K07Rik	-2.2265105	4.105E-02	Downregulated
Cntnap1	-2.0889260	3.840E-03	Downregulated
Nek6	-2.0877223	1.740E-02	Downregulated
C230037L18Rik	-2.0524201	1.756E-02	Downregulated
Zcchc14	-1.9283250	3.198E-02	Downregulated
Tmem8b	-1.8831505	3.896E-02	Downregulated
Vash1	-1.8815227	2.845E-02	Downregulated
Pdzd3	-1.7420180	4.718E-02	Downregulated
Spats2	-1.7267650	4.256E-02	Downregulated
Ccdc173	-1.6896497	4.483E-02	Downregulated
Zfp473	-1.6642390	4.515E-02	Downregulated
Ccsap	-1.4906796	2.758E-02	Downregulated
Sgsm2	-1.4605537	2.715E-02	Downregulated
Gm11696	-1.3962252	4.573E-02	Downregulated
Srpk2	-1.3436871	5.403E-03	Downregulated
Ssbp4	-1.1750369	6.744E-03	Downregulated
Pde4a	-1.1200335	4.779E-02	Downregulated
Rps8	1.0811586	4.297E-02	Upregulated
Vps72	1.1206330	4.685E-02	Upregulated
Rpl5	1.1517787	3.078E-02	Upregulated

Tceal9	1.1864467	4.731E-02	Upregulated
Rpsa	1.1969873	3.154E-02	Upregulated
Rps13	1.2017553	3.574E-02	Upregulated
Rps25	1.2347540	1.825E-02	Upregulated
B9d2	1.2469764	3.194E-02	Upregulated
Rpl32	1.2525499	2.281E-02	Upregulated
Pdcd6	1.2597283	2.388E-02	Upregulated
Pno1	1.2656426	2.988E-02	Upregulated
Nfu1	1.2845753	3.629E-02	Upregulated
Lsm4	1.3153775	4.908E-02	Upregulated
Rps5	1.3247914	1.003E-02	Upregulated
Dctn3	1.3303363	1.965E-02	Upregulated
Cfap298	1.3421300	2.523E-02	Upregulated
Rpl10a	1.3481053	1.757E-02	Upregulated
2410004B18Rik	1.3711571	2.703E-02	Upregulated
Mtln	1.3780609	4.930E-02	Upregulated
Thoc6	1.4032021	4.567E-02	Upregulated
Rpsa-ps10	1.4267939	4.201E-02	Upregulated
Rps3	1.4280110	1.040E-02	Upregulated
Rack1	1.4294936	7.077E-03	Upregulated
Dtwd1	1.4477377	4.935E-02	Upregulated
Timm10	1.4651879	3.802E-03	Upregulated
Gm37906	1.4761553	4.411E-02	Upregulated
Atp5o	1.4982743	2.951E-02	Upregulated
Med29	1.5004807	4.910E-02	Upregulated
Rps15a	1.5009362	3.279E-02	Upregulated
Ndufc2	1.5207462	4.521E-02	Upregulated
Zfp85	1.5519791	1.568E-02	Upregulated

Ndufb6	1.5778235	4.934E-02	Upregulated
Zfp383	1.5779061	3.206E-02	Upregulated
Ndufc1	1.5831630	1.669E-02	Upregulated
Cox16	1.5994261	1.372E-02	Upregulated
2700062C07Rik	1.6039109	4.232E-02	Upregulated
Txndc12	1.6293583	9.663E-04	Upregulated
Dynlt1f	1.6827396	2.205E-02	Upregulated
Gm17655	1.6865422	2.269E-02	Upregulated
Oxld1	1.6998278	4.912E-02	Upregulated
Smim26	1.7216814	4.583E-02	Upregulated
Fbxo6	1.7286039	1.502E-02	Upregulated
Kcnc3	1.7296099	3.024E-02	Upregulated
Tst	1.7360034	4.017E-02	Upregulated
Anxa1	1.7424238	2.523E-02	Upregulated
Ust	1.7438044	1.905E-02	Upregulated
Tigd5	1.7508164	3.570E-02	Upregulated
Mvk	1.7724765	2.000E-02	Upregulated
Grin2d	1.7769405	3.472E-02	Upregulated
Rpp38	1.8049449	4.353E-02	Upregulated
Neb	1.8168994	1.156E-02	Upregulated
Fbxw17	1.8642915	3.191E-02	Upregulated
Tspo	1.8861356	1.436E-02	Upregulated
Gm37139	1.9024751	4.613E-02	Upregulated
Gm38365	1.9087474	1.410E-02	Upregulated
Cacna2d4	1.9103776	4.013E-02	Upregulated
Zfp775	1.9610352	1.403E-02	Upregulated
Pcbp4	1.9816364	5.293E-03	Upregulated
1700094M23Rik	2.1090956	4.905E-02	Upregulated

4833445I07Rik	2.1106596	3.124E-02	Upregulated
Mcam	2.1137352	4.412E-02	Upregulated
Trim46	2.1412803	3.145E-02	Upregulated
Gm45206	2.1415457	3.990E-02	Upregulated
Eef1a1-ps1	2.1770983	4.590E-02	Upregulated
Mgst3	2.1807869	1.355E-02	Upregulated
Wfdc21	2.1828675	3.586E-02	Upregulated
Gm9531	2.2084357	4.279E-02	Upregulated
Snora78	2.2177599	1.097E-02	Upregulated
Dync2i2	2.2472457	3.697E-02	Upregulated
Mapk8ip1	2.2500607	3.513E-02	Upregulated
Kank2	2.2869935	1.819E-02	Upregulated
Msl3l2	2.3014135	4.360E-02	Upregulated
Nudt12	2.3150013	1.722E-02	Upregulated
Ifnlr1	2.3295141	3.831E-02	Upregulated
Chil3	2.3420160	2.389E-02	Upregulated
Gm11423	2.3560528	3.488E-02	Upregulated
Gm37390	2.3603705	4.101E-02	Upregulated
Gm45137	2.3772399	1.013E-02	Upregulated
Scg5	2.4194726	4.150E-02	Upregulated
Phactr1	2.4284899	3.951E-02	Upregulated
Gm32051	2.4379552	2.223E-02	Upregulated
Lbhd1	2.4464170	3.000E-02	Upregulated
Asb17os	2.4468282	2.433E-02	Upregulated
C030017G13Rik	2.4599041	2.641E-02	Upregulated
Gm47246	2.4756853	4.373E-02	Upregulated
Galnt9	2.4916966	4.743E-02	Upregulated
Gpc3	2.5033722	3.115E-02	Upregulated

Ulk4	2.5284994	4.979E-02	Upregulated
Cst7	2.5358050	1.747E-02	Upregulated
Ptp4a1	2.5359981	1.549E-02	Upregulated
Dhrs13	2.5425968	3.274E-02	Upregulated
Fth-ps2	2.5429700	4.860E-02	Upregulated
Gm15417	2.5489581	3.356E-02	Upregulated
Trbv13-1	2.5507856	4.396E-02	Upregulated
Nkain4	2.5589083	2.725E-02	Upregulated
Gm5621	2.5605883	5.823E-03	Upregulated
U6	2.5640565	2.592E-02	Upregulated
Alpk3	2.5723899	4.551E-02	Upregulated
Cdc42ep1	2.5726385	4.946E-02	Upregulated
Actn3	2.5859404	7.585E-03	Upregulated
Lonrf3	2.6007383	4.319E-02	Upregulated
Stum	2.6050734	4.231E-02	Upregulated
Ighv5-9-1	2.6065556	3.384E-02	Upregulated
Gpr27	2.6160546	4.132E-02	Upregulated
Arhgef26	2.6183639	4.101E-02	Upregulated
Zfp442	2.6786157	4.082E-02	Upregulated
Nrxn3	2.7016368	3.893E-02	Upregulated
Gnai1	2.7042457	3.824E-02	Upregulated
Cpa3	2.7108855	1.732E-02	Upregulated
Bco2	2.7155901	4.441E-02	Upregulated
Trib3	2.7345263	4.645E-02	Upregulated
Pnma3	2.7362867	4.542E-02	Upregulated
Gli3	2.7487091	4.431E-02	Upregulated
Nhs	2.7502656	4.259E-02	Upregulated
Cdh20	2.7525310	4.224E-02	Upregulated

Neto1	2.7526218	4.269E-02	Upregulated
Pcdhga8	2.7544493	4.345E-02	Upregulated
Sgpp2	2.7548694	4.222E-02	Upregulated
Anks6	2.7551506	4.386E-02	Upregulated
C730002L08Rik	2.7565113	4.691E-02	Upregulated
Grid2ip	2.7578616	4.457E-02	Upregulated
Vwa3b	2.7600611	4.866E-02	Upregulated
Gm44888	2.7607303	3.878E-02	Upregulated
Gm42688	2.7609247	9.069E-03	Upregulated
Adcyap1r1	2.7627411	4.085E-02	Upregulated
Cgref1	2.7699583	4.205E-02	Upregulated
Fgd1	2.7705920	4.140E-02	Upregulated
Ect2l	2.7724040	2.837E-02	Upregulated
Tacc2	2.7765449	1.954E-04	Upregulated
Sema3c	2.7808097	4.692E-02	Upregulated
Gm43844	2.7824914	3.821E-02	Upregulated
Gm48353	2.7991999	4.961E-02	Upregulated
Faxc	2.8008133	2.411E-02	Upregulated
1700047M11Rik	2.8054843	3.001E-02	Upregulated
Proca1	2.8061325	4.270E-02	Upregulated
Togaram2	2.8064693	4.898E-02	Upregulated
C1qtnf4	2.8210340	3.100E-02	Upregulated
2810429I04Rik	2.8212396	3.357E-02	Upregulated
Gm37115	2.8214820	7.843E-04	Upregulated
Cadm4	2.8281384	2.698E-02	Upregulated
Ano1	2.8300687	3.498E-02	Upregulated
Atg9b	2.8378577	4.654E-02	Upregulated
Des	2.8427327	3.408E-02	Upregulated

Sh3bgr	2.8434962	3.306E-02	Upregulated
Gm43024	2.8458487	1.884E-02	Upregulated
Gm37255	2.8561711	1.148E-02	Upregulated
Kctd15	2.8672515	3.712E-02	Upregulated
Ldhd	2.8804218	2.325E-02	Upregulated
Shmt1	2.8808349	1.341E-02	Upregulated
Tmem240	2.8982253	1.902E-02	Upregulated
Gm44907	2.9036172	3.603E-02	Upregulated
Mettl5os	2.9174853	4.577E-02	Upregulated
Gm32282	2.9222960	3.762E-02	Upregulated
Angptl6	2.9288612	3.052E-02	Upregulated
Lrig3	2.9353245	2.750E-02	Upregulated
Zkscan4	2.9375649	2.675E-02	Upregulated
Lrrc3b	2.9426353	2.523E-02	Upregulated
Gm5805	2.9429142	1.294E-02	Upregulated
Pcdhgb7	2.9472496	2.502E-02	Upregulated
Noxred1	2.9474855	5.585E-03	Upregulated
Lamc2	2.9544423	2.711E-02	Upregulated
Adamtsl1	2.9549263	1.891E-02	Upregulated
St18	2.9587223	2.433E-02	Upregulated
Stamos	2.9628507	3.982E-02	Upregulated
Pcdh18	2.9643078	2.448E-02	Upregulated
Dclk1	2.9652408	1.793E-02	Upregulated
Nexn	2.9720933	2.387E-02	Upregulated
Gm5881	2.9759616	3.884E-02	Upregulated
Gm36949	2.9790942	3.522E-02	Upregulated
Vwa5b2	2.9878726	2.277E-02	Upregulated
Gm17096	3.0015413	4.084E-02	Upregulated

Gm20109	3.0017869	1.904E-02	Upregulated
Htra3	3.0081498	2.344E-02	Upregulated
Gm44806	3.0083159	3.495E-02	Upregulated
Gm48335	3.0121634	2.108E-02	Upregulated
Ackr3	3.0138738	6.209E-03	Upregulated
Ptges3l	3.0296895	3.298E-02	Upregulated
Casq2	3.0335778	2.272E-02	Upregulated
Gm17072	3.0363399	4.254E-02	Upregulated
Gm4787	3.0620478	1.846E-02	Upregulated
Ace2	3.0862876	2.575E-02	Upregulated
Iqck	3.0896709	2.493E-02	Upregulated
Gm49706	3.0924483	2.392E-02	Upregulated
Ckmt1	3.1050684	1.532E-02	Upregulated
Gm37584	3.1060055	4.397E-02	Upregulated
Dync2li1	3.1114634	1.484E-02	Upregulated
Gm45407	3.1156536	2.278E-02	Upregulated
Mid2	3.1196983	1.447E-02	Upregulated
Gjc2	3.1215462	1.603E-02	Upregulated
Cntn1	3.1246724	1.400E-02	Upregulated
Gm26287	3.1261443	2.939E-02	Upregulated
Dsp	3.1377028	2.636E-02	Upregulated
Gm9903	3.1435552	4.120E-02	Upregulated
Gm43259	3.1438351	2.519E-02	Upregulated
Mycbpap	3.1517985	4.181E-02	Upregulated
Atp13a5	3.1532327	1.340E-02	Upregulated
Gm43111	3.1666682	1.541E-02	Upregulated
Ak1	3.1697046	1.291E-02	Upregulated
Trim34b	3.1717410	1.787E-02	Upregulated

Gm42992	3.1726612	1.623E-02	Upregulated
6430710M23Rik	3.1770457	1.876E-02	Upregulated
Ablim3	3.1871025	1.499E-02	Upregulated
Il18	3.1922510	8.329E-03	Upregulated
Gm43684	3.1936336	3.945E-02	Upregulated
Tagln	3.1993563	3.050E-02	Upregulated
Gm38376	3.2078093	2.072E-02	Upregulated
Camk2a	3.2135065	3.594E-02	Upregulated
Gm47112	3.2151607	2.667E-02	Upregulated
Acvr1	3.2200120	9.735E-03	Upregulated
Myh14	3.2205372	1.933E-02	Upregulated
Gm38257	3.2317842	1.035E-02	Upregulated
Gm37062	3.2377663	1.434E-02	Upregulated
2700029L08Rik	3.2396901	1.363E-02	Upregulated
Tmem88	3.2492351	2.356E-02	Upregulated
Camsap3	3.2509571	9.580E-03	Upregulated
Tmem144	3.2660104	9.272E-03	Upregulated
Gm44210	3.2681143	1.081E-02	Upregulated
Astn1	3.2758060	8.545E-03	Upregulated
Olfml2b	3.2782151	4.196E-02	Upregulated
Syt2	3.2819750	8.924E-03	Upregulated
Gm37766	3.2944625	2.200E-02	Upregulated
Gm16794	3.2970662	2.062E-02	Upregulated
4930570G19Rik	3.3032985	8.784E-03	Upregulated
Zbbx	3.3072106	1.052E-02	Upregulated
Rgsl1	3.3126351	2.417E-02	Upregulated
Gm9974	3.3145708	3.200E-02	Upregulated
Slit2	3.3156913	1.932E-02	Upregulated

Gm48965	3.3161729	2.618E-02	Upregulated
Prss23	3.3189203	1.238E-02	Upregulated
Tnnt3	3.3284807	1.172E-02	Upregulated
Gm8437	3.3383160	1.651E-02	Upregulated
Gm26064	3.3502978	1.629E-02	Upregulated
Tacr1	3.3507888	3.091E-02	Upregulated
Jsrp1	3.3563167	7.955E-03	Upregulated
Ttn	3.3644326	1.035E-05	Upregulated
4930579K19Rik	3.3649926	1.487E-02	Upregulated
Trpv4	3.3701577	1.940E-02	Upregulated
Gm24198	3.3836199	3.004E-02	Upregulated
Gm50070	3.3885563	3.123E-02	Upregulated
Dach2	3.3893225	7.623E-03	Upregulated
C920006O11Rik	3.3905336	8.619E-03	Upregulated
Gm48765	3.3993954	3.839E-02	Upregulated
Mobp	3.4015760	6.083E-03	Upregulated
Pcsk4	3.4031319	8.360E-03	Upregulated
Peg3	3.4053575	5.496E-03	Upregulated
Gm43389	3.4085042	7.732E-03	Upregulated
Jam2	3.4093683	5.777E-03	Upregulated
ENSMUSG00002075696	3.4112307	2.140E-02	Upregulated
Celf4	3.4124602	5.212E-03	Upregulated
Sdk1	3.4125964	5.356E-03	Upregulated
Eef1a2	3.4175009	5.270E-03	Upregulated
Rnf227	3.4236886	5.424E-03	Upregulated
Serp2	3.4356706	3.059E-03	Upregulated
Prss36	3.4442634	4.657E-03	Upregulated
Gpm6a	3.4548414	2.966E-03	Upregulated

Six4	3.4552130	1.049E-02	Upregulated
Gm9754	3.4609610	1.147E-02	Upregulated
Efna1	3.4636446	6.989E-03	Upregulated
Gm27252	3.4692329	1.319E-02	Upregulated
Rxfp2	3.4760185	2.532E-02	Upregulated
Amt	3.4789846	3.223E-03	Upregulated
Col22a1	3.4918230	3.591E-02	Upregulated
Zfp105	3.4923645	4.341E-03	Upregulated
Atoh8	3.5031792	9.651E-03	Upregulated
Gm37607	3.5235425	4.385E-03	Upregulated
Zfp385c	3.5280042	8.597E-03	Upregulated
Rps18-ps6	3.5301056	1.130E-02	Upregulated
ENSMUSG00002075672	3.5307237	1.574E-02	Upregulated
Mir342	3.5341385	3.320E-02	Upregulated
Gm37672	3.5387597	7.950E-03	Upregulated
Gm11725	3.5400901	1.250E-02	Upregulated
Gfra1	3.5645807	8.266E-03	Upregulated
Xirp2	3.5652721	4.380E-02	Upregulated
Rpsa-ps7	3.5665244	8.617E-03	Upregulated
Fhod3	3.5716080	3.381E-03	Upregulated
Tekt5	3.6001485	2.849E-03	Upregulated
Rin1	3.6070934	7.845E-03	Upregulated
Rangrf	3.6261317	2.981E-03	Upregulated
Gm28707	3.6297465	1.330E-02	Upregulated
Gm22270	3.6307075	1.624E-03	Upregulated
1700113A16Rik	3.6379422	7.302E-03	Upregulated
Frmpd4	3.6568927	2.078E-03	Upregulated
Gm8146	3.7072648	4.924E-02	Upregulated

Gm15569	3.7138540	1.077E-02	Upregulated
Gm17249	3.7175844	5.471E-03	Upregulated
Gm5499	3.7190713	1.920E-02	Upregulated
Ramp3	3.7483339	2.022E-03	Upregulated
Synm	3.7650583	1.074E-03	Upregulated
Flnc	3.7677030	2.409E-03	Upregulated
Pex11g	3.7776523	6.481E-03	Upregulated
Bmp7	3.7975667	1.868E-03	Upregulated
Adamtsl3	3.7989221	8.862E-03	Upregulated
Gm20163	3.8057766	1.488E-03	Upregulated
Nol3	3.8451605	1.045E-03	Upregulated
Gm25636	3.8528542	1.206E-02	Upregulated
Ppp1r42	3.8583160	1.314E-02	Upregulated
Gm7054	3.8927305	1.572E-02	Upregulated
Atp2a1	3.8948770	4.090E-04	Upregulated
Gm26132	3.9039999	1.970E-02	Upregulated
Myh4	3.9158271	1.156E-03	Upregulated
Capn12	3.9308207	3.284E-02	Upregulated
Lrp2	3.9366590	1.300E-02	Upregulated
Tagln3	3.9516852	6.335E-04	Upregulated
Trpm1	3.9562998	1.715E-02	Upregulated
Asic1	3.9569226	6.269E-04	Upregulated
Gm15672	3.9711502	8.085E-03	Upregulated
Fcrlb	3.9786297	8.722E-03	Upregulated
Gm42928	3.9980018	5.659E-03	Upregulated
F3	4.0051424	5.510E-04	Upregulated
Lefty1	4.0085755	2.809E-03	Upregulated
Lox	4.0115512	1.647E-02	Upregulated

Sema4c	4.0345850	1.196E-03	Upregulated
3110080O07Rik	4.0486331	1.716E-03	Upregulated
Usp13	4.0535433	4.038E-04	Upregulated
3425401B19Rik	4.0666313	1.358E-02	Upregulated
Gstt1	4.0774048	2.370E-06	Upregulated
Rapsn	4.0966534	6.715E-03	Upregulated
Gm10687	4.0998431	4.325E-03	Upregulated
Traj22	4.1021166	3.159E-02	Upregulated
Crmp1	4.1910304	4.133E-03	Upregulated
Acta1	4.2519975	2.369E-05	Upregulated
Gm10247	4.3264213	3.699E-02	Upregulated
Gm28941	4.3549506	7.324E-04	Upregulated
Ckm	4.5767110	4.108E-02	Upregulated
Speg	4.5811708	2.994E-03	Upregulated
A3galt2	4.6693725	1.942E-04	Upregulated
Arhgdig	4.6772533	1.621E-03	Upregulated
BC016579	4.7903941	4.008E-04	Upregulated
Scrn1	4.8435000	9.124E-04	Upregulated
Myh1	4.9665303	1.600E-06	Upregulated
Snora41	5.0121556	6.734E-05	Upregulated
Mylk4	5.0889852	3.170E-03	Upregulated
Myl1	5.1493659	1.204E-03	Upregulated
Mb	5.4430740	1.399E-02	Upregulated
Tcap	5.9102321	7.375E-05	Upregulated
Tchh	5.9470051	4.540E-05	Upregulated
Myh2	6.1448910	8.106E-05	Upregulated

Table of Contents Top of Current Table

Supplemental Table 23. List of all significant (P < 0.05) differentially expressed genes in whole blood of wild-type B6129 CBD (week 8) versus wild-type B6129 vehicle (week 8) animals; 179 genes downregulated & 182 genes upregulated.

Gene Name	Log2FoldChange	P-value	Regulation
Tex11	-5.5927361	1.290E-03	Downregulated
Gm47326	-4.9279619	1.190E-02	Downregulated
Ocln	-4.7322858	3.305E-04	Downregulated
Loxl2	-4.6739310	5.925E-03	Downregulated
Ccm2l	-4.6115259	2.553E-04	Downregulated
Gm47861	-4.3250549	2.047E-02	Downregulated
Gm44430	-4.2899292	7.188E-04	Downregulated
Hmcn1	-4.1879350	2.369E-03	Downregulated
Slc7a2	-4.1864463	1.088E-03	Downregulated
Gm26850	-4.1518502	8.399E-03	Downregulated
Gm38009	-4.1151914	3.247E-03	Downregulated
Gm28592	-4.1048642	1.252E-02	Downregulated
Cyp26b1	-4.0987396	8.659E-04	Downregulated
Gpr161	-4.0784206	4.455E-04	Downregulated
Gm26631	-4.0593072	1.576E-02	Downregulated
Calcb	-4.0380831	6.958E-03	Downregulated
Bcam	-4.0365805	3.754E-04	Downregulated
Gm26788	-3.9007245	1.778E-02	Downregulated
Cxcl13	-3.8790595	4.200E-02	Downregulated
Scarf2	-3.8480655	1.940E-03	Downregulated
Gm12798	-3.8009080	3.502E-02	Downregulated
Gm17949	-3.7958540	1.584E-02	Downregulated
A730063M14Rik	-3.7895500	3.068E-03	Downregulated
Lct	-3.7864264	3.028E-02	Downregulated
Gm16570	-3.7760246	1.531E-02	Downregulated
Gbgt1	-3.7691251	1.763E-02	Downregulated

Klra13-ps	-3.7366115	4.518E-03	Downregulated
Gm47484	-3.7366099	3.114E-03	Downregulated
Apod	-3.7344522	2.219E-03	Downregulated
Tmem179	-3.7322726	2.082E-03	Downregulated
Grem2	-3.7302532	2.100E-03	Downregulated
A430105J06Rik	-3.7184716	8.970E-03	Downregulated
A230103J11Rik	-3.6630832	5.716E-03	Downregulated
Mme	-3.6596011	1.099E-03	Downregulated
Igfbp3	-3.6094433	4.208E-03	Downregulated
Mpdz	-3.5971586	1.193E-03	Downregulated
3222401L13Rik	-3.5956755	7.464E-03	Downregulated
Hhipl1	-3.5949791	3.409E-03	Downregulated
Gm9929	-3.5869396	2.085E-02	Downregulated
Gm4247	-3.5862572	4.570E-03	Downregulated
Gm42615	-3.5798554	5.204E-03	Downregulated
Il20ra	-3.5673286	1.064E-02	Downregulated
Pex11g	-3.4765146	1.794E-02	Downregulated
Gm35248	-3.4692979	4.249E-02	Downregulated
Gm25632	-3.4643406	4.234E-02	Downregulated
Fgf18	-3.4408058	1.963E-02	Downregulated
Slc7a14	-3.4407214	5.853E-03	Downregulated
Syt7	-3.4383966	5.772E-03	Downregulated
Gm10912	-3.3988768	3.824E-02	Downregulated
Ephb1	-3.3968676	5.067E-03	Downregulated
Zfp558	-3.3337572	2.157E-02	Downregulated
Gm29670	-3.3175522	3.109E-03	Downregulated
Otoa	-3.3175397	2.791E-02	Downregulated
Gm27252	-3.3174786	2.652E-02	Downregulated
Crb2	-3.3103231	1.592E-02	Downregulated

Edil3	-3.2859342	7.726E-03	Downregulated
Medag	-3.2810353	1.270E-02	Downregulated
Gli2	-3.2785520	1.153E-02	Downregulated
Lama2	-3.2741624	7.727E-03	Downregulated
Gm44735	-3.2735480	2.749E-02	Downregulated
Kansl2-ps	-3.2726790	3.038E-02	Downregulated
Pcdhb22	-3.2715170	1.091E-02	Downregulated
ENSMUSG00002075052	-3.2694088	4.731E-02	Downregulated
Trpm3	-3.2663489	1.071E-02	Downregulated
Tnfaip6	-3.2662643	1.116E-02	Downregulated
Hs3st3a1	-3.2656172	4.287E-02	Downregulated
6430573P05Rik	-3.2644399	3.078E-02	Downregulated
Meox1	-3.2637505	4.768E-02	Downregulated
Ttc34	-3.2597271	1.431E-02	Downregulated
Gm43868	-3.2539271	2.774E-02	Downregulated
A930037H05Rik	-3.2529578	3.661E-02	Downregulated
Grip2	-3.2458159	1.795E-02	Downregulated
Gm15853	-3.2132338	1.362E-02	Downregulated
Sec16b	-3.2056897	1.330E-02	Downregulated
Gm34655	-3.2036699	2.406E-02	Downregulated
Gm50323	-3.1857389	3.747E-02	Downregulated
Grm1	-3.1520283	1.149E-02	Downregulated
Cryab	-3.1492009	7.915E-03	Downregulated
C4b	-3.1477408	3.741E-02	Downregulated
Prg4	-3.1282547	3.189E-02	Downregulated
Gm19972	-3.0888850	2.627E-02	Downregulated
Amn	-3.0850209	3.886E-02	Downregulated
Hrh1	-3.0827121	2.053E-02	Downregulated
1700001L19Rik	-3.0812893	2.145E-02	Downregulated

Crispld1	-3.0791558	2.124E-02	Downregulated
Rgma	-3.0783224	1.958E-02	Downregulated
Gm13689	-3.0743458	2.737E-02	Downregulated
Prrt3	-3.0721119	2.096E-02	Downregulated
Tmem132a	-3.0674705	1.098E-02	Downregulated
Slc22a17	-3.0486745	1.017E-02	Downregulated
Gm17068	-3.0408818	3.825E-02	Downregulated
Fras1	-3.0385749	2.488E-02	Downregulated
Gm43072	-3.0280464	2.346E-02	Downregulated
4632411P08Rik	-3.0191242	3.071E-02	Downregulated
L3mbtl1	-3.0181086	1.988E-02	Downregulated
Baiap2l1	-3.0166557	3.756E-02	Downregulated
Gm45629	-2.9994693	1.063E-02	Downregulated
Cldn10	-2.9794819	7.704E-03	Downregulated
Col8a2	-2.9465715	2.275E-02	Downregulated
Lrrc24	-2.9446241	1.443E-02	Downregulated
C1qa	-2.9105946	8.055E-03	Downregulated
Adgrl4	-2.9103186	1.263E-02	Downregulated
Gm16861	-2.8894420	4.867E-02	Downregulated
4931415C17Rik	-2.8771483	3.336E-02	Downregulated
Wfdc1	-2.8680726	4.020E-02	Downregulated
Fam171a1	-2.8636873	2.752E-02	Downregulated
Fhod3	-2.8628934	3.053E-02	Downregulated
Gm44433	-2.8576865	3.971E-02	Downregulated
Phpt1	-2.8566030	3.882E-02	Downregulated
Sox1	-2.8557377	3.779E-02	Downregulated
Omg	-2.8552706	3.778E-02	Downregulated
Gabbr2	-2.8550027	3.799E-02	Downregulated
Gabrd	-2.8544969	3.721E-02	Downregulated

Mpped2	-2.8537301	3.739E-02	Downregulated
Asic2	-2.8528273	3.750E-02	Downregulated
Ptchd1	-2.8523869	3.848E-02	Downregulated
Susd4	-2.8521185	3.746E-02	Downregulated
Pfn4	-2.8519886	4.200E-02	Downregulated
Vstm2b	-2.8495780	3.832E-02	Downregulated
Ramp2	-2.8476072	4.593E-02	Downregulated
Gm45623	-2.8329698	4.581E-02	Downregulated
P4ha2	-2.8148291	1.541E-02	Downregulated
Enpp2	-2.7795795	1.315E-03	Downregulated
Sod3	-2.7764448	4.699E-02	Downregulated
Gm44067	-2.7333348	1.775E-02	Downregulated
Gabra3	-2.7280894	2.760E-02	Downregulated
4732496C06Rik	-2.7269642	4.612E-02	Downregulated
Thbs4	-2.7081078	4.999E-02	Downregulated
Plcxd1	-2.6962465	4.797E-02	Downregulated
Peg3	-2.6943572	4.466E-02	Downregulated
Smim10l2a	-2.6906345	4.576E-02	Downregulated
Kif17	-2.6862694	4.799E-02	Downregulated
Rarres2	-2.6683049	2.952E-02	Downregulated
Thsd1	-2.6603258	3.652E-02	Downregulated
Ankrd45	-2.6456220	6.041E-03	Downregulated
Zfp366	-2.6352142	1.457E-02	Downregulated
Fzd1	-2.6095332	2.601E-03	Downregulated
Panct2	-2.5348139	2.436E-02	Downregulated
Gm43062	-2.4889804	3.809E-02	Downregulated
Pcdhgb8	-2.4785394	4.350E-02	Downregulated
Zfp503	-2.4482931	2.342E-02	Downregulated
Slamf9	-2.4283859	4.592E-02	Downregulated

Maoa	-2.4128110	6.154E-03	Downregulated
Tgfb2	-2.4022370	2.811E-02	Downregulated
Sema3c	-2.3503184	9.966E-03	Downregulated
Galnt9	-2.3296029	2.644E-02	Downregulated
Aplp1	-2.3149985	1.742E-02	Downregulated
Gm37062	-2.3051614	4.047E-02	Downregulated
Tmeff1	-2.3021130	3.818E-02	Downregulated
E230020D15Rik	-2.2523114	4.500E-02	Downregulated
Fgf2	-2.2362727	2.205E-02	Downregulated
Fn1	-2.2331562	2.078E-02	Downregulated
Lims2	-2.1989851	3.725E-02	Downregulated
C1qb	-2.1757803	4.704E-02	Downregulated
Serpinb8	-2.1023433	3.719E-02	Downregulated
Rilpl1	-2.0938844	1.013E-02	Downregulated
Fgfr1	-2.0748512	6.323E-03	Downregulated
Tspan4	-2.0695976	3.387E-02	Downregulated
Sdc3	-2.0348368	2.101E-02	Downregulated
Ikzf4	-2.0344705	3.602E-03	Downregulated
Il33	-2.0331552	4.953E-02	Downregulated
Gm16793	-1.9093041	4.337E-02	Downregulated
Pcp4l1	-1.8887740	3.591E-02	Downregulated
Gm3055	-1.8854764	4.903E-02	Downregulated
Zfp365	-1.8804745	2.283E-02	Downregulated
Nphp3	-1.8601802	1.956E-02	Downregulated
Gm47644	-1.8222778	1.817E-02	Downregulated
Dpysl3	-1.8222270	3.629E-02	Downregulated
BC037704	-1.7730992	3.119E-02	Downregulated
Zfp661	-1.7546054	2.058E-02	Downregulated
Cttnbp2nl	-1.6374913	4.385E-02	Downregulated

Lysmd2	-1.6190245	5.237E-03	Downregulated
Tyro3	-1.5825706	2.933E-02	Downregulated
Tmem176b	-1.5813879	2.510E-02	Downregulated
Dhrs13	-1.5516021	4.120E-02	Downregulated
Klhl20	-1.5065653	1.988E-02	Downregulated
Niban2	-1.4375339	4.138E-02	Downregulated
Pfdn2	-1.2449896	2.009E-02	Downregulated
Pea15a	-1.2208420	2.399E-02	Downregulated
G3bp2	1.1361063	1.314E-02	Upregulated
Gm34220	1.3895131	3.154E-02	Upregulated
Piga	1.4941379	7.569E-03	Upregulated
Serinc3	1.5049389	2.385E-02	Upregulated
Adam22	1.6870586	3.443E-02	Upregulated
Syt14	1.7864029	4.142E-03	Upregulated
Gm50069	1.8704626	2.532E-02	Upregulated
Slc15a2	1.8941698	4.135E-02	Upregulated
Rn18s-rs5	1.9158249	3.520E-02	Upregulated
Pik3r3	1.9310273	2.521E-02	Upregulated
Il17rd	2.0115762	3.865E-02	Upregulated
Gm43627	2.0630722	2.850E-02	Upregulated
Cracdl	2.0717751	2.444E-02	Upregulated
Efnb3	2.1257201	3.091E-02	Upregulated
Sdc2	2.1871699	1.738E-02	Upregulated
Gm38381	2.3165080	3.050E-02	Upregulated
Gm43807	2.3284037	4.938E-02	Upregulated
Slco5a1	2.3308806	4.560E-02	Upregulated
Exph5	2.3613401	2.459E-02	Upregulated
Itgad	2.3725191	4.722E-02	Upregulated
Lmbr1	2.3767700	4.520E-02	Upregulated

Gm8091	2.4654241	2.592E-02	Upregulated
3110062G12Rik	2.5119059	4.094E-02	Upregulated
Morc4	2.5397937	3.748E-02	Upregulated
Gm25813	2.5767454	3.133E-02	Upregulated
Gm48718	2.7032879	3.267E-02	Upregulated
Gm42551	2.7083835	4.696E-02	Upregulated
Gm45274	2.7201419	2.117E-02	Upregulated
Fbxl13	2.7345318	7.660E-03	Upregulated
Gm44175	2.7576427	4.679E-02	Upregulated
Myo5c	2.7879927	2.110E-02	Upregulated
Plscr2	2.8445345	4.229E-02	Upregulated
Sgtb	2.8660410	3.556E-02	Upregulated
Sall2	2.9078531	4.103E-03	Upregulated
Hif3a	2.9312135	1.897E-02	Upregulated
6720482G16Rik	2.9591712	4.587E-02	Upregulated
Arhgef33	2.9749919	3.040E-02	Upregulated
Abi3bp	3.0098368	4.480E-03	Upregulated
Olfr552	3.0257719	3.004E-02	Upregulated
Gm6061	3.0307998	3.686E-02	Upregulated
Zfp449	3.0433525	3.278E-02	Upregulated
Ccdc3	3.0534511	2.397E-02	Upregulated
Gm6278	3.0628514	9.125E-03	Upregulated
Ccn2	3.0893193	4.867E-02	Upregulated
1700094M23Rik	3.1064690	3.090E-02	Upregulated
1700029H14Rik	3.1081104	3.953E-02	Upregulated
Fstl4	3.1228020	4.431E-02	Upregulated
Dbn1	3.1646396	1.597E-02	Upregulated
Rgs22	3.1820976	2.059E-02	Upregulated
Bex2	3.1994399	4.794E-02	Upregulated

Chgb	3.2007411	4.776E-02	Upregulated
Lin7b	3.2027893	4.777E-02	Upregulated
Arfgef3	3.2040942	4.716E-02	Upregulated
Fmn2	3.2067382	4.697E-02	Upregulated
Jph1	3.2068087	4.702E-02	Upregulated
S100b	3.2164193	4.791E-02	Upregulated
Cacng4	3.2210650	3.600E-02	Upregulated
Ccnjl	3.2679294	2.439E-02	Upregulated
5730408A14Rik	3.2723023	4.704E-02	Upregulated
Gm48335	3.2919909	1.633E-02	Upregulated
Gm18180	3.2953680	2.027E-03	Upregulated
Foxj1	3.3297781	2.522E-02	Upregulated
Vsnl1	3.3727584	2.561E-02	Upregulated
Gm7008	3.3870658	3.960E-02	Upregulated
Fbxl21	3.3925383	3.337E-02	Upregulated
Hjv	3.4190217	3.720E-02	Upregulated
Etv4	3.4196434	3.175E-02	Upregulated
Marchf4	3.4199284	3.006E-02	Upregulated
Tmem35a	3.4230737	2.953E-02	Upregulated
Gm27253	3.4233418	4.817E-02	Upregulated
Akap6	3.4242100	2.894E-02	Upregulated
Vmn2r53	3.4270300	4.782E-02	Upregulated
Msi1	3.4291603	2.916E-02	Upregulated
Tmem25	3.4296105	2.908E-02	Upregulated
Kndc1	3.4332710	2.890E-02	Upregulated
Ptprz1	3.4352548	2.876E-02	Upregulated
Gm32200	3.4399104	4.580E-02	Upregulated
Entpd2	3.4399585	3.022E-02	Upregulated
Rimbp3	3.4422343	4.954E-02	Upregulated

Ccn3	3.4510332	2.921E-02	Upregulated
Meg3	3.4580310	2.204E-02	Upregulated
Gm14413	3.4601951	3.125E-02	Upregulated
D630045J12Rik	3.4666615	2.043E-02	Upregulated
Greb1l	3.4760706	2.038E-02	Upregulated
Gm42685	3.4792605	3.217E-02	Upregulated
Ptpru	3.4840054	2.054E-02	Upregulated
Klf15	3.4843195	2.009E-02	Upregulated
Ndn	3.4894804	2.103E-02	Upregulated
Zc2hc1c	3.5101317	2.952E-02	Upregulated
Kcnh8	3.5494607	4.538E-02	Upregulated
C230071H17Rik	3.5653718	2.525E-02	Upregulated
Foxb1	3.5729142	2.674E-02	Upregulated
Gm43787	3.5776062	2.179E-02	Upregulated
Gm10516	3.5856420	1.693E-02	Upregulated
Galnt18	3.6088785	1.913E-02	Upregulated
Nav3	3.6156214	1.852E-02	Upregulated
Lrrc3b	3.6211129	1.870E-02	Upregulated
Gcnt4	3.6217075	1.898E-02	Upregulated
Stum	3.6233263	1.834E-02	Upregulated
Ntn5	3.6469807	2.138E-02	Upregulated
Fsip1	3.6479664	3.681E-02	Upregulated
E230025N22Rik	3.6519827	4.537E-02	Upregulated
Plch2	3.6603838	1.328E-02	Upregulated
Fbl-ps2	3.6614666	3.432E-02	Upregulated
B930036N10Rik	3.6897755	1.396E-02	Upregulated
Klhl35	3.6965997	4.459E-02	Upregulated
Gm19196	3.7336741	3.344E-02	Upregulated
Angptl1	3.7364649	3.256E-02	Upregulated

Ccl27a	3.7384816	1.064E-02	Upregulated
Calb2	3.7499107	1.397E-02	Upregulated
Dll4	3.7698664	2.644E-02	Upregulated
Lhfpl3	3.7948685	1.194E-02	Upregulated
Cntnap5b	3.8026701	1.175E-02	Upregulated
Rassf10	3.8386479	2.434E-02	Upregulated
Gm49077	3.8557096	2.860E-02	Upregulated
Acot12	3.8796506	4.281E-02	Upregulated
Cdh15	3.8803480	1.198E-02	Upregulated
Gm13523	3.8876768	4.724E-02	Upregulated
4930509G22Rik	3.8952700	4.188E-02	Upregulated
4930593A02Rik	3.8967340	3.503E-02	Upregulated
ENSMUSG00002074935	3.8980218	2.941E-02	Upregulated
Gm48333	3.9043969	4.677E-02	Upregulated
Gm49192	3.9131271	3.246E-02	Upregulated
Col9a2	3.9141489	1.289E-02	Upregulated
B3gat1	3.9320577	8.200E-03	Upregulated
Arxes2	3.9378833	8.366E-03	Upregulated
Mybpc1	3.9555680	4.076E-02	Upregulated
Gm26756	3.9789756	2.026E-02	Upregulated
Col11a1	3.9852746	8.523E-03	Upregulated
Gm44144	3.9942587	9.820E-03	Upregulated
Tbx15	3.9944830	3.745E-02	Upregulated
A730098A19Rik	3.9992579	2.382E-02	Upregulated
1700028K03Rik	4.0217876	1.019E-02	Upregulated
ENSMUSG00002076530	4.0241743	1.805E-02	Upregulated
Edn1	4.0345626	2.668E-02	Upregulated
Gm36757	4.0394368	1.423E-02	Upregulated

Cfap99	4.0491518	2.048E-02	Upregulated
Gm38301	4.0541602	3.747E-02	Upregulated
Gucy2g	4.0742595	7.944E-03	Upregulated
Bcat1	4.0791097	5.579E-03	Upregulated
Hmgcll1	4.0804514	5.534E-03	Upregulated
Gm37827	4.0877608	2.706E-02	Upregulated
Lvrn	4.0945165	3.614E-02	Upregulated
Gm48684	4.1034515	1.476E-02	Upregulated
Tspear	4.1693885	2.364E-02	Upregulated
Egflam	4.1829031	2.866E-02	Upregulated
Gm39822	4.1902431	2.194E-02	Upregulated
Gm49975	4.1957895	3.139E-02	Upregulated
A330094K24Rik	4.2038982	1.502E-02	Upregulated
Atp2b2	4.2070946	3.810E-03	Upregulated
Gm8738	4.2793058	1.161E-02	Upregulated
Gm38026	4.2834602	2.839E-02	Upregulated
2610528A11Rik	4.3018378	4.374E-02	Upregulated
Rbp4	4.3020972	4.290E-03	Upregulated
Palmd	4.3309924	3.013E-03	Upregulated
Synpo2l	4.3362992	2.496E-02	Upregulated
Mir6939	4.3876790	4.735E-02	Upregulated
Gm14086	4.4217550	3.984E-02	Upregulated
Gm13349	4.4388560	6.267E-03	Upregulated
Ptgs2	4.4410207	5.718E-03	Upregulated
Gm17981	4.4424026	5.849E-03	Upregulated
Lypd1	4.5151532	1.148E-02	Upregulated
Chrm5	4.5242455	2.561E-02	Upregulated
Alox12e	4.5494239	2.081E-02	Upregulated
Cstad	4.5697898	3.411E-03	Upregulated

Gm8730	4.6027648	4.704E-06	Upregulated
Gm48780	4.6093745	2.022E-03	Upregulated
Dpp6	4.6204594	1.084E-03	Upregulated
Sfrp1	4.7229310	8.115E-04	Upregulated
Chil5	4.7239190	8.737E-03	Upregulated
Gm38560	4.7422790	4.410E-02	Upregulated
Gm11934	4.7551048	1.873E-02	Upregulated
Gm20449	4.8508497	7.079E-03	Upregulated
Slc5a2	4.8643909	3.022E-02	Upregulated
Gm16558	4.8936597	2.679E-03	Upregulated
Copz2	4.9747021	4.262E-04	Upregulated
1700003G18Rik	5.0249418	2.673E-02	Upregulated
U6	5.1695711	4.685E-02	Upregulated
Gm9625	5.3299537	1.994E-06	Upregulated
Cfap65	5.3700860	1.431E-03	Upregulated
Gm33100	5.4593842	2.374E-02	Upregulated
Gm14165	6.7491200	3.256E-07	Upregulated

Table of Contents Top of Current Table

Supplemental Table 24. List of all significant (P < 0.05) differentially expressed genes in whole blood of 3xTg-AD CBD (week 8) versus wild-type B6129 CBD (week 8) animals; 569 genes downregulated & 696 genes upregulated.

Gene Name	Log2FoldChange	P-value	Regulation
Gm49980	-26.32259102	1.756E-22	Downregulated
Tdrd5	-21.11831210	2.617E-38	Downregulated
H2-Q2	-20.57201189	8.988E-41	Downregulated
Gm35082	-11.41202994	3.093E-14	Downregulated
Sult4a1	-9.64809418	3.583E-07	Downregulated
H2af-ps2	-8.78715989	6.042E-06	Downregulated
Dntt	-7.77474757	1.035E-03	Downregulated
Gm43305	-7.04677704	1.178E-02	Downregulated
4930447F24Rik	-6.80514843	6.094E-06	Downregulated
Rorc	-6.71363506	1.466E-05	Downregulated
Col6a4	-6.54699417	3.075E-06	Downregulated
Rpl31-ps15	-6.51747741	1.115E-05	Downregulated
Gvin-ps1	-6.39308760	9.789E-07	Downregulated
Eno1b	-6.22125554	1.102E-03	Downregulated
Cpne4	-6.10994626	2.485E-06	Downregulated
Rps3a3	-5.99500031	1.398E-07	Downregulated
Gm6916	-5.83099572	1.380E-03	Downregulated
Gm14287	-5.78270396	6.739E-04	Downregulated
Rag1	-5.60366064	3.314E-05	Downregulated
Gm12312	-5.46923605	9.307E-05	Downregulated
Klrc3	-5.40536053	7.134E-04	Downregulated
Aox4	-5.38300359	8.567E-06	Downregulated

Gm42743	-5.37367803	1.635E-04	Downregulated
Gm23123	-5.32681312	9.328E-03	Downregulated
Olfr552	-5.31505218	1.416E-04	Downregulated
Gm49975	-5.22777950	3.671E-03	Downregulated
Gm33474	-5.19631962	7.540E-03	Downregulated
Gm26881	-5.10722708	1.303E-04	Downregulated
Zfp125	-5.09934164	8.531E-05	Downregulated
Hunk	-5.01984467	2.325E-05	Downregulated
BB031773	-4.92719283	9.506E-03	Downregulated
1810059H22Rik	-4.86266590	5.388E-03	Downregulated
Slc6a19	-4.74331272	3.964E-04	Downregulated
Gm26510	-4.66245174	1.679E-03	Downregulated
Ighv1-14	-4.65495174	3.959E-02	Downregulated
Gm42752	-4.63532235	2.825E-02	Downregulated
Gm34590	-4.59655779	1.275E-03	Downregulated
Ighv3-2	-4.53115991	1.303E-02	Downregulated
Arsi	-4.52409108	6.245E-03	Downregulated
Traj44	-4.35423481	4.612E-02	Downregulated
Arl11	-4.30981313	2.812E-04	Downregulated
Gm37534	-4.30686445	7.189E-05	Downregulated
Dgkeos	-4.27261754	3.075E-02	Downregulated
Pawr	-4.22976323	3.470E-04	Downregulated
Gm46210	-4.20202244	9.769E-05	Downregulated
A930035D04Rik	-4.19079025	2.771E-03	Downregulated
Endou	-4.16556129	1.012E-03	Downregulated

Gm40841	-4.14942067	4.345E-02	Downregulated
Fgfrl1	-4.13487821	5.782E-07	Downregulated
Kcnh3	-4.12968863	1.922E-03	Downregulated
Lvrn	-4.10509491	1.637E-02	Downregulated
Spats2l	-4.10023634	8.559E-04	Downregulated
Cib3	-4.08028508	2.775E-02	Downregulated
Gm45640	-4.07615056	4.701E-03	Downregulated
Ubb-ps	-4.06474182	1.582E-05	Downregulated
Nr4a2	-4.05099193	3.333E-04	Downregulated
Gm31323	-4.00049569	4.778E-03	Downregulated
Whrn	-3.92381190	1.693E-05	Downregulated
Scn2b	-3.90544230	4.470E-04	Downregulated
Tmem169	-3.88750207	3.480E-03	Downregulated
Scgb1c1	-3.87918571	1.477E-02	Downregulated
3110062G12Rik	-3.87660371	2.668E-03	Downregulated
Gm14382	-3.86367967	1.653E-02	Downregulated
H4c17	-3.85537368	5.361E-03	Downregulated
7SK	-3.85333102	3.528E-02	Downregulated
Rasl11b	-3.84028609	1.085E-03	Downregulated
Platr26	-3.81798919	4.619E-02	Downregulated
Cpa2	-3.76290051	4.266E-03	Downregulated
Sall2	-3.74388645	1.218E-03	Downregulated
Gpr162	-3.73510101	1.959E-02	Downregulated
Cd1d2	-3.73332463	3.784E-02	Downregulated
Lmbr1	-3.73017818	5.126E-05	Downregulated

Hmgcs2	-3.70363445	4.401E-03	Downregulated
Pcdhga12	-3.69490830	1.461E-03	Downregulated
Jazf1	-3.69454291	1.817E-03	Downregulated
Gm26766	-3.68796027	4.159E-03	Downregulated
Eps8l1	-3.67953414	4.391E-02	Downregulated
Gm37844	-3.67410770	6.973E-03	Downregulated
Ccdc3	-3.67004796	1.786E-03	Downregulated
Gm35147	-3.66967551	4.125E-02	Downregulated
Gm15459	-3.63516361	6.669E-05	Downregulated
Gsta3	-3.61642809	8.403E-03	Downregulated
Gm42728	-3.60940150	7.985E-03	Downregulated
Gm16155	-3.60367700	3.164E-02	Downregulated
5S_rRNA	-3.59836980	2.302E-02	Downregulated
Gm37536	-3.58528540	1.009E-02	Downregulated
Gm49376	-3.58045453	9.853E-03	Downregulated
Gm20695	-3.57528363	4.159E-02	Downregulated
Gm10400	-3.57164868	9.128E-03	Downregulated
Slc16a2	-3.52738686	6.070E-03	Downregulated
Gm23887	-3.49640735	1.304E-02	Downregulated
Gm43143	-3.49424286	7.690E-03	Downregulated
Gask1b	-3.48491032	3.769E-03	Downregulated
Mir652	-3.47855235	3.109E-02	Downregulated
Gm11868	-3.46992413	6.069E-03	Downregulated
Ccl27a	-3.46500950	3.387E-03	Downregulated
Gm37632	-3.46230721	3.875E-02	Downregulated

Gm22786	-3.44708619	3.185E-02	Downregulated
Gm10037	-3.42862961	1.505E-02	Downregulated
Ighv2-9	-3.42308134	1.126E-02	Downregulated
Gm6630	-3.39927343	4.396E-02	Downregulated
Gm29596	-3.38269936	3.637E-02	Downregulated
Samd13	-3.37996846	2.587E-02	Downregulated
Gm973	-3.37358304	4.924E-03	Downregulated
Gm20732	-3.37177557	7.159E-03	Downregulated
Gm15708	-3.36756357	1.391E-02	Downregulated
Gm11948	-3.35149903	1.429E-02	Downregulated
Btf3-ps13	-3.33394198	3.545E-02	Downregulated
Map1a	-3.32584003	6.774E-13	Downregulated
Evc	-3.30670287	6.385E-03	Downregulated
Gm22571	-3.29212661	2.603E-02	Downregulated
Mycl	-3.28048530	3.797E-03	Downregulated
Kirrel	-3.27664212	7.011E-03	Downregulated
F12	-3.27110771	3.330E-02	Downregulated
Gm45356	-3.26823421	1.798E-02	Downregulated
Cabp5	-3.26090602	4.349E-02	Downregulated
Gm40663	-3.23440050	3.580E-02	Downregulated
2410004P03Rik	-3.23024307	1.629E-02	Downregulated
Gm44936	-3.22206234	2.418E-02	Downregulated
Acan	-3.21570033	4.547E-02	Downregulated
Ptgs2	-3.20575459	2.109E-02	Downregulated
Gm13349	-3.20358700	2.275E-02	Downregulated

Gm13307	-3.19715447	1.411E-02	Downregulated
Cdh22	-3.19322136	9.036E-03	Downregulated
Fut10	-3.19216522	2.527E-04	Downregulated
Prokr2	-3.19156856	2.203E-02	Downregulated
Lrp11	-3.18719771	8.872E-03	Downregulated
Mir1927	-3.18340096	4.282E-02	Downregulated
Gfra2	-3.17001159	3.638E-03	Downregulated
Gm12971	-3.14966540	2.196E-02	Downregulated
Cracdl	-3.14475201	5.220E-03	Downregulated
Gm43560	-3.13909635	4.503E-02	Downregulated
Gm45148	-3.12433155	1.544E-02	Downregulated
Reep1	-3.11896651	5.457E-03	Downregulated
Sox17	-3.11718508	2.867E-02	Downregulated
Sbsn	-3.11294148	1.897E-02	Downregulated
Vsnl1	-3.09928949	1.199E-02	Downregulated
Mal	-3.09678141	1.230E-02	Downregulated
Matn2	-3.09677497	1.440E-02	Downregulated
Gm6061	-3.09523500	2.666E-02	Downregulated
Bsn	-3.09243220	4.020E-03	Downregulated
Gm14681	-3.09207835	3.279E-02	Downregulated
Il17rd	-3.08166136	7.430E-03	Downregulated
Mxra8	-3.08095821	1.787E-02	Downregulated
F2rl2	-3.08065188	4.244E-04	Downregulated
Capza1-ps1	-3.07128187	8.020E-05	Downregulated
Il34	-3.07070536	1.107E-02	Downregulated

Gm33056	-3.06103380	8.091E-03	Downregulated
Trdv2-2	-3.06011737	2.729E-02	Downregulated
Gm26930	-3.05630632	3.302E-02	Downregulated
Gm12737	-3.05458118	1.531E-02	Downregulated
Gm38223	-3.05307699	3.658E-02	Downregulated
Gm8738	-3.04403797	4.065E-02	Downregulated
Gm15667	-3.03180946	2.909E-02	Downregulated
H2-Bl	-3.02193069	4.784E-02	Downregulated
Adamts14	-3.01787977	2.676E-06	Downregulated
Gm49894	-3.01711082	4.110E-02	Downregulated
Gm43186	-3.01318820	2.189E-02	Downregulated
Zfp462	-3.01161351	1.639E-03	Downregulated
Car9	-3.00610132	4.771E-02	Downregulated
1810010H24Rik	-2.99860177	2.048E-02	Downregulated
4633401B06Rik	-2.99161413	2.073E-02	Downregulated
Rassf10	-2.98893255	4.119E-02	Downregulated
D130019J16Rik	-2.98784519	4.142E-03	Downregulated
Spag6l	-2.98474417	1.787E-02	Downregulated
Ubxn10	-2.98454084	4.028E-02	Downregulated
P3h3	-2.98274077	1.092E-02	Downregulated
Gm20432	-2.98267875	4.871E-02	Downregulated
Opcml	-2.97505059	1.754E-02	Downregulated
Atp2b2	-2.97183128	1.758E-02	Downregulated
Gm44043	-2.95967341	4.921E-02	Downregulated
Nrn1	-2.95664615	1.866E-02	Downregulated

Laptm4b	-2.95195893	1.706E-02	Downregulated
Ccr9	-2.94962901	5.024E-03	Downregulated
Cacng4	-2.94759436	1.939E-02	Downregulated
Gm13423	-2.93849784	4.673E-02	Downregulated
C1ql3	-2.93659543	2.061E-02	Downregulated
A130050O07Rik	-2.90694048	1.814E-02	Downregulated
Clvs1	-2.89985479	2.362E-03	Downregulated
Adgrl3	-2.89464384	3.874E-02	Downregulated
Styk1	-2.89067434	5.710E-04	Downregulated
Cpeb1	-2.89028639	1.851E-02	Downregulated
Gm45807	-2.87405302	2.911E-02	Downregulated
Lhx1	-2.86759174	1.998E-02	Downregulated
Cdr2l	-2.86220759	2.082E-02	Downregulated
Gm37298	-2.85938998	1.340E-02	Downregulated
Ldb2	-2.85730488	2.530E-02	Downregulated
Rec8	-2.84733929	2.910E-02	Downregulated
Ccn1	-2.83720095	2.444E-02	Downregulated
2010310C07Rik	-2.83604055	1.611E-03	Downregulated
Gm47583	-2.83047408	3.747E-02	Downregulated
Gm37537	-2.80673654	4.243E-02	Downregulated
Gm50433	-2.80419801	2.317E-02	Downregulated
Gm48780	-2.79735193	3.477E-02	Downregulated
Tanc1	-2.79668196	8.159E-10	Downregulated
1700028K03Rik	-2.78651916	4.313E-02	Downregulated
ENSMUSG00002075075	-2.78326240	4.063E-02	Downregulated

C230035I16Rik	-2.76776780	3.374E-02	Downregulated
Plekhf1	-2.76164211	3.655E-02	Downregulated
Cstad	-2.75797673	4.861E-02	Downregulated
Gm26782	-2.75210144	2.640E-02	Downregulated
Col11a1	-2.75000821	3.866E-02	Downregulated
Myadml2	-2.74870015	2.188E-03	Downregulated
Fabp3	-2.74530398	3.622E-02	Downregulated
Sema3g	-2.74173687	4.502E-02	Downregulated
Lrfn1	-2.73999185	4.942E-02	Downregulated
Rasl10a	-2.73411588	3.121E-02	Downregulated
Mroh7	-2.72978906	4.511E-02	Downregulated
Gm6475	-2.72061171	3.894E-02	Downregulated
Nectin3	-2.71500695	3.195E-02	Downregulated
Rgs7bp	-2.71326684	3.109E-02	Downregulated
Rimkla	-2.70843443	3.281E-02	Downregulated
Mtcl1	-2.70410422	3.939E-02	Downregulated
Hsf4	-2.69819606	4.314E-02	Downregulated
Gm37780	-2.69246119	8.077E-05	Downregulated
Gm42433	-2.68131711	4.254E-02	Downregulated
Adh7	-2.67895096	4.104E-02	Downregulated
Gm43702	-2.67362647	2.415E-02	Downregulated
P3h4	-2.66981793	3.615E-02	Downregulated
Gfi1	-2.66386389	2.705E-02	Downregulated
Gm44045	-2.65597362	1.558E-02	Downregulated
Ndn	-2.63887308	4.147E-02	Downregulated

Arhgef15	-2.63123768	4.083E-02	Downregulated
Rapgef3	-2.62815456	1.486E-02	Downregulated
Zfp951	-2.62377382	2.086E-02	Downregulated
Slc16a14	-2.62350474	1.463E-02	Downregulated
Nlgn3	-2.60747354	4.130E-02	Downregulated
Mir7048	-2.60417349	3.325E-03	Downregulated
Gm15283	-2.60275683	2.679E-02	Downregulated
Gm15943	-2.59721033	4.209E-02	Downregulated
Gm48670	-2.58746542	4.339E-02	Downregulated
Ly6g6c	-2.57665234	9.500E-06	Downregulated
Gm4202	-2.57257966	5.091E-03	Downregulated
Pcp4l1	-2.57171621	2.945E-04	Downregulated
Gm10548	-2.56852039	4.133E-02	Downregulated
Emp1	-2.56689784	4.706E-03	Downregulated
Gm43820	-2.55964260	1.543E-02	Downregulated
Cracr2b	-2.55793715	3.521E-02	Downregulated
Clba1	-2.55295502	4.137E-02	Downregulated
Gm10451	-2.51435784	1.663E-02	Downregulated
Ntrk2	-2.50625974	3.918E-02	Downregulated
Pde6h	-2.47063619	3.152E-02	Downregulated
C1qtnf7	-2.46349765	4.421E-02	Downregulated
Pde7b	-2.43893546	1.830E-07	Downregulated
Gm13577	-2.43169884	1.085E-02	Downregulated
Gm527	-2.42652113	2.733E-03	Downregulated
Abca6	-2.42329180	4.643E-02	Downregulated

Gsta2	-2.41907386	3.952E-03	Downregulated
Ptk7	-2.40327886	3.224E-02	Downregulated
Hspd1-ps4	-2.40288545	5.478E-05	Downregulated
4930598N05Rik	-2.38638391	6.244E-06	Downregulated
Gm37498	-2.38173960	4.711E-02	Downregulated
Serpine2	-2.37354891	1.562E-06	Downregulated
Arpp21	-2.37112228	5.537E-04	Downregulated
Sytl2	-2.36084608	2.683E-06	Downregulated
Spa17	-2.35181318	1.168E-02	Downregulated
Lancl3	-2.34679747	6.126E-05	Downregulated
Rps13-ps1	-2.34224953	1.570E-02	Downregulated
Mcoln3	-2.33736287	3.269E-02	Downregulated
Eif3j2	-2.32383748	2.846E-03	Downregulated
Nim1k	-2.31605065	2.054E-02	Downregulated
Gm38414	-2.30980549	7.344E-03	Downregulated
Gm27019	-2.29110789	2.731E-02	Downregulated
Ndnf	-2.28330964	1.565E-05	Downregulated
Star	-2.28108197	2.202E-02	Downregulated
Mir8116	-2.27615322	3.792E-02	Downregulated
Asprv1	-2.26516070	1.320E-03	Downregulated
Pdlim4	-2.24953025	2.584E-02	Downregulated
Gm11274	-2.24864032	2.377E-04	Downregulated
Hic2	-2.24567292	1.719E-02	Downregulated
Clstn1	-2.22964498	9.825E-04	Downregulated
Hpse2	-2.21365270	4.967E-02	Downregulated

Mest	-2.21348803	5.569E-04	Downregulated
Zbtb8a	-2.21227231	3.650E-02	Downregulated
Yap1	-2.20622023	7.332E-07	Downregulated
Zfyve28	-2.20349265	1.330E-02	Downregulated
Islr2	-2.20176503	3.906E-02	Downregulated
Ksr2	-2.18521646	3.700E-02	Downregulated
Clgn	-2.16127022	3.502E-02	Downregulated
Gm10286	-2.15330325	6.798E-04	Downregulated
Peg10	-2.15316275	1.176E-05	Downregulated
Ccr5	-2.14613139	7.560E-04	Downregulated
Rdh5	-2.14313408	4.404E-02	Downregulated
Triqk	-2.13530823	1.223E-04	Downregulated
Gm37570	-2.12707241	4.665E-02	Downregulated
Ephb6	-2.12328498	1.477E-02	Downregulated
Myo6	-2.11336751	7.222E-07	Downregulated
Cntnap1	-2.09059064	2.682E-03	Downregulated
Nhsl1	-2.08720149	3.549E-02	Downregulated
Plp1	-2.08714257	1.366E-06	Downregulated
Tle2	-2.07616934	2.250E-02	Downregulated
Sytl4	-2.07190749	7.397E-06	Downregulated
6230400D17Rik	-2.06755159	2.782E-02	Downregulated
Poglut3	-2.06100678	9.290E-03	Downregulated
Gm9403	-2.05204800	3.524E-02	Downregulated
Pyroxd2	-2.04887511	1.542E-02	Downregulated
Tmprss7	-2.04284639	2.606E-02	Downregulated

Armcx6	-2.02063723	4.124E-03	Downregulated
Nomo1	-2.00612975	2.157E-05	Downregulated
Enah	-2.00173624	3.384E-04	Downregulated
Tfec	-1.99167295	2.784E-02	Downregulated
Atp10a	-1.96697038	1.004E-02	Downregulated
C230037L18Rik	-1.95854022	2.231E-02	Downregulated
Tox	-1.94413558	1.037E-04	Downregulated
Klrb1c	-1.94394049	2.999E-02	Downregulated
Klhdc8b	-1.91702788	1.574E-02	Downregulated
Hsd17b14	-1.90465001	4.275E-02	Downregulated
P3h2	-1.89722102	3.786E-02	Downregulated
Camk4	-1.89692084	2.051E-02	Downregulated
Prodh	-1.89075546	6.702E-03	Downregulated
Gm36328	-1.88539703	7.459E-03	Downregulated
Intu	-1.87362478	5.338E-04	Downregulated
Ncr1	-1.86461419	6.018E-03	Downregulated
Arhgef17	-1.84090587	9.400E-03	Downregulated
Exoc3l2	-1.83358173	3.110E-02	Downregulated
Ubc	-1.83259223	8.335E-03	Downregulated
Nek6	-1.83127724	4.479E-02	Downregulated
Ubap1	-1.82777358	2.026E-05	Downregulated
Tmem158	-1.82155966	2.381E-04	Downregulated
Gp5	-1.81472684	6.590E-05	Downregulated
Slc18a2	-1.81186649	1.161E-03	Downregulated
Prkaa2	-1.81121813	3.991E-05	Downregulated

C5ar2	-1.79908218	3.287E-02	Downregulated
Pros1	-1.79062692	3.035E-05	Downregulated
Cd101	-1.78637686	1.621E-02	Downregulated
Cfap97d2	-1.78387728	6.010E-03	Downregulated
Tmem14a	-1.78355329	1.225E-03	Downregulated
Cxcl5	-1.76849732	1.903E-02	Downregulated
Tubb2b	-1.76845572	1.244E-03	Downregulated
A930005H10Rik	-1.76678518	7.838E-03	Downregulated
Adam22	-1.76264164	2.294E-02	Downregulated
Loxl1	-1.75435342	2.621E-02	Downregulated
Zdhhc12	-1.75190136	4.881E-02	Downregulated
Mpig6b	-1.74037504	1.144E-03	Downregulated
Phtf2	-1.73771471	3.343E-07	Downregulated
Pafah2	-1.73689809	2.305E-02	Downregulated
Dlg4	-1.72893051	2.088E-07	Downregulated
Gm30648	-1.72461639	3.452E-02	Downregulated
Ufsp1	-1.71692486	1.067E-02	Downregulated
Tpi1	-1.71501822	2.139E-06	Downregulated
Gfod2	-1.70340363	1.508E-02	Downregulated
Stau2	-1.69515184	3.381E-03	Downregulated
Sez6l2	-1.68984215	2.716E-02	Downregulated
Glis3	-1.68820285	6.385E-03	Downregulated
2310034O05Rik	-1.67748742	2.292E-02	Downregulated
Cd81	-1.66804630	5.755E-04	Downregulated
Tdrp	-1.66590780	1.592E-02	Downregulated

Mr1	-1.65331255	2.533E-02	Downregulated
Sh3bgrl3	-1.64826641	3.241E-05	Downregulated
Mfap3l	-1.64078557	7.271E-05	Downregulated
Nefh	-1.64074931	1.977E-02	Downregulated
Tmbim1	-1.64058377	1.499E-03	Downregulated
Nav2	-1.64035987	2.601E-02	Downregulated
Gm11639	-1.63600319	3.841E-02	Downregulated
Trim9	-1.63466729	3.771E-02	Downregulated
Zscan2	-1.62978551	6.982E-03	Downregulated
Trp53i13	-1.62270236	4.214E-02	Downregulated
Nudt18	-1.62004370	3.765E-03	Downregulated
Inafm2	-1.61829312	6.411E-05	Downregulated
Clec1b	-1.61220497	1.024E-03	Downregulated
Tmem151b	-1.60947461	1.164E-02	Downregulated
Plekha7	-1.60843652	4.145E-02	Downregulated
Cd27	-1.60790187	4.189E-02	Downregulated
Pf4	-1.60590038	4.035E-03	Downregulated
Fgl2	-1.60296549	2.050E-02	Downregulated
Ppbp	-1.59998675	1.767E-02	Downregulated
6030458C11Rik	-1.59570162	4.521E-02	Downregulated
Camkk1	-1.58766151	9.661E-03	Downregulated
Hpse	-1.57289704	2.168E-03	Downregulated
Klrk1	-1.56272179	3.718E-02	Downregulated
Mras	-1.56235701	1.883E-03	Downregulated
Cdk5rap1	-1.55597360	1.312E-02	Downregulated

Tlcd3b	-1.55208361	2.856E-02	Downregulated
Nlrp3	-1.55173930	2.521E-02	Downregulated
Slc39a4	-1.55063973	6.159E-03	Downregulated
Tlr12	-1.55035055	1.176E-02	Downregulated
Scamp1	-1.55018852	4.636E-04	Downregulated
Slc16a12	-1.54862556	5.120E-03	Downregulated
Nckap5l	-1.54705145	2.243E-02	Downregulated
Cd28	-1.54432758	3.083E-03	Downregulated
Apoe	-1.54400022	3.331E-02	Downregulated
Layn	-1.54225433	8.114E-03	Downregulated
Muc13	-1.53318084	3.289E-02	Downregulated
Dusp14	-1.53102551	4.433E-02	Downregulated
Fam219a	-1.52501301	2.673E-03	Downregulated
Csgalnact1	-1.52474679	1.064E-03	Downregulated
Gm36279	-1.52175314	1.582E-02	Downregulated
Rab36	-1.51737940	7.347E-03	Downregulated
Dapk1	-1.51668070	6.942E-03	Downregulated
Sox4	-1.50049452	1.273E-02	Downregulated
Ifit2	-1.49779882	8.451E-03	Downregulated
Rab37	-1.49177124	6.667E-04	Downregulated
Lhfp	-1.48223698	1.744E-03	Downregulated
Aldh1l1	-1.48122991	4.989E-02	Downregulated
Gpm6b	-1.47673116	1.872E-02	Downregulated
9330175E14Rik	-1.47658457	4.336E-02	Downregulated
Slc24a3	-1.47078871	1.281E-03	Downregulated

Ar	-1.46943044	1.862E-02	Downregulated
Cdc42ep5	-1.46807504	8.606E-03	Downregulated
Nbdy	-1.45987476	2.136E-02	Downregulated
Cd4	-1.44922324	4.912E-02	Downregulated
Sh3bgrl2	-1.44688622	1.339E-03	Downregulated
Rnf180	-1.44332579	1.266E-02	Downregulated
Bicd2	-1.44316948	8.683E-05	Downregulated
Angpt1	-1.43973615	3.922E-03	Downregulated
Gm11696	-1.43881993	2.860E-02	Downregulated
Elovl7	-1.43828552	1.517E-02	Downregulated
Lpin1	-1.43351728	2.542E-03	Downregulated
Rtl8c	-1.43238221	4.077E-03	Downregulated
Tmc1	-1.42638722	3.910E-02	Downregulated
Tspan9	-1.42338323	2.328E-03	Downregulated
Gp1ba	-1.40526703	6.748E-03	Downregulated
Gm15706	-1.40423813	4.914E-02	Downregulated
Gucy1a1	-1.40354522	1.341E-02	Downregulated
Cavin1	-1.40040945	1.252E-02	Downregulated
Anxa4	-1.39531238	3.873E-02	Downregulated
Litaf	-1.39503054	6.782E-03	Downregulated
Pik3c3	-1.39196632	1.459E-03	Downregulated
Itga2	-1.39104772	4.900E-03	Downregulated
Dclre1b	-1.38712652	4.749E-03	Downregulated
Gm28609	-1.38579644	4.055E-02	Downregulated
Sgce	-1.38191424	1.418E-02	Downregulated

Cep126	-1.37767208	2.663E-02	Downregulated
Ccdc92	-1.37224344	2.478E-03	Downregulated
Mctp1	-1.37217643	1.056E-02	Downregulated
P2ry12	-1.36958694	2.095E-02	Downregulated
Wnk4	-1.36935156	4.529E-02	Downregulated
Maged2	-1.36913910	2.827E-03	Downregulated
Mpl	-1.36849500	1.668E-02	Downregulated
Piga	-1.36194276	9.726E-03	Downregulated
Abi2	-1.35891361	1.124E-03	Downregulated
Gucy1b1	-1.35752605	3.156E-03	Downregulated
Tmem245	-1.35631849	2.738E-05	Downregulated
Tollip	-1.35340443	8.671E-04	Downregulated
Rida	-1.35310754	1.606E-02	Downregulated
Tle1	-1.35139656	2.592E-02	Downregulated
Nudt3	-1.35017448	5.041E-03	Downregulated
Itgb1	-1.34801225	6.744E-04	Downregulated
Stx11	-1.34458963	1.321E-02	Downregulated
Fads2	-1.33901377	1.089E-02	Downregulated
Hacd4	-1.33696408	4.704E-03	Downregulated
Ergic1	-1.33352082	7.279E-04	Downregulated
Mmd	-1.33325958	1.036E-02	Downregulated
Panx1	-1.33063814	2.073E-03	Downregulated
Dip2c	-1.33004021	2.929E-02	Downregulated
Mcf2l	-1.32850937	1.486E-02	Downregulated
Pea15a	-1.32218269	4.984E-03	Downregulated

Pkd1l3	-1.31523535	1.559E-02	Downregulated
Acer2	-1.31404027	1.940E-02	Downregulated
Fam118a	-1.31086024	2.741E-02	Downregulated
Prdx6	-1.30883903	4.070E-03	Downregulated
Gad1	-1.30770822	2.431E-02	Downregulated
Dhcr24	-1.29632832	5.791E-03	Downregulated
Acss2	-1.29523435	1.160E-02	Downregulated
Esam	-1.29252358	1.330E-02	Downregulated
Fkbp1a	-1.29241700	2.295E-03	Downregulated
Dusp3	-1.29039878	4.090E-03	Downregulated
F5	-1.28821480	2.237E-02	Downregulated
Tsc22d1	-1.28644366	1.122E-02	Downregulated
Itgb5	-1.28500208	7.104E-03	Downregulated
Ormdl3	-1.28386043	2.191E-02	Downregulated
Plxna4	-1.28369407	4.772E-03	Downregulated
Ecm1	-1.28288489	1.010E-02	Downregulated
Dgke	-1.28080025	2.582E-02	Downregulated
Ramp1	-1.28068364	2.765E-02	Downregulated
Gp6	-1.27888373	1.353E-02	Downregulated
Coa5	-1.27831235	4.108E-03	Downregulated
Dnal4	-1.27644438	4.223E-02	Downregulated
Vwf	-1.27563855	3.581E-02	Downregulated
Dhrs3	-1.26662267	1.354E-02	Downregulated
Lrrn3	-1.26354586	2.296E-02	Downregulated
Itga6	-1.25950006	5.938E-03	Downregulated

Nol4l	-1.25906926	1.769E-02	Downregulated
Ltbp1	-1.25759555	1.297E-02	Downregulated
Ppp3r1	-1.25608918	1.752E-04	Downregulated
Mfsd6	-1.25549106	1.095E-03	Downregulated
Nrgn	-1.25392086	1.653E-02	Downregulated
Rap2a	-1.25307484	7.825E-03	Downregulated
Dut	-1.25288620	2.449E-02	Downregulated
Derl1	-1.24808881	1.354E-03	Downregulated
Cnst	-1.24429083	4.730E-03	Downregulated
Gata2	-1.24190334	1.462E-02	Downregulated
Dap	-1.24059200	2.805E-02	Downregulated
Clic4	-1.23797968	6.211E-03	Downregulated
Snx10	-1.23514623	9.997E-03	Downregulated
Mast2	-1.23278968	5.836E-03	Downregulated
Hpcal1	-1.23182865	6.434E-03	Downregulated
Lrrc29	-1.22714744	4.431E-02	Downregulated
Itgb3	-1.21676465	2.729E-02	Downregulated
Dedd	-1.21269540	1.261E-03	Downregulated
Nptn	-1.21100415	8.046E-03	Downregulated
Cd7	-1.20105037	3.799E-02	Downregulated
Pdk1	-1.19772399	8.253E-03	Downregulated
Ndrg1	-1.19133375	1.049E-02	Downregulated
Adcy9	-1.19004792	2.020E-02	Downregulated
Prokr1	-1.18467112	2.411E-02	Downregulated
Nacc2	-1.18335208	1.532E-02	Downregulated

Pcdh7	-1.18295560	4.091E-02	Downregulated
Gas2l1	-1.17850073	2.059E-02	Downregulated
Tpm4	-1.17762250	2.257E-02	Downregulated
Septin9	-1.17172794	1.897E-03	Downregulated
Cd44	-1.17169980	3.391E-03	Downregulated
Cd9	-1.17165572	4.703E-02	Downregulated
Rnf11	-1.17095116	3.912E-02	Downregulated
Parvb	-1.16654342	1.633E-02	Downregulated
Fam217b	-1.16624359	1.861E-02	Downregulated
Clu	-1.16596582	2.741E-02	Downregulated
Srxn1	-1.16382183	4.562E-02	Downregulated
As3mt	-1.16378742	9.398E-03	Downregulated
Card19	-1.16017156	4.975E-02	Downregulated
Nqo2	-1.16014982	3.691E-02	Downregulated
Itpr2	-1.15994641	5.852E-04	Downregulated
Arl4c	-1.15501502	1.294E-02	Downregulated
Lat	-1.15352821	3.213E-02	Downregulated
Kcna3	-1.14652149	2.848E-02	Downregulated
Zfp236	-1.14020776	1.947E-02	Downregulated
Tspan14	-1.13920351	3.380E-03	Downregulated
Ctif	-1.13452858	3.414E-02	Downregulated
Ro60	-1.13414401	3.507E-02	Downregulated
Otud7b	-1.13020567	1.898E-02	Downregulated
Ctnna1	-1.12932642	1.640E-02	Downregulated
Gatad1	-1.12748391	3.533E-02	Downregulated

Pgm2l1	-1.12639246	2.404E-02	Downregulated
Slc2a3	-1.12267901	2.226E-02	Downregulated
Rtn4ip1	-1.11937706	3.061E-02	Downregulated
Bcr	-1.11764914	1.849E-02	Downregulated
Dzip1	-1.11608455	3.789E-02	Downregulated
Ptms	-1.11573309	2.977E-02	Downregulated
Pear1	-1.11551847	1.532E-02	Downregulated
Tmem170b	-1.11513509	7.012E-03	Downregulated
Atp2c1	-1.11401247	6.474E-03	Downregulated
Ywhaz	-1.11108202	1.509E-02	Downregulated
Bbx	-1.11096669	3.305E-03	Downregulated
Cfl1	-1.10568622	1.115E-02	Downregulated
Tmod3	-1.10472936	9.854E-03	Downregulated
Ccdc171	-1.10181325	3.076E-02	Downregulated
Abhd2	-1.10110903	3.096E-03	Downregulated
Aldh2	-1.09972744	2.557E-02	Downregulated
AI504432	-1.09215295	2.225E-02	Downregulated
Gsn	-1.08822740	3.607E-02	Downregulated
Tmem9b	-1.08687654	4.801E-02	Downregulated
Ssbp4	-1.08256110	1.913E-02	Downregulated
Atp2a3	-1.08245801	3.206E-02	Downregulated
2510009E07Rik	-1.08079906	4.915E-02	Downregulated
Tmem43	-1.07764054	2.066E-02	Downregulated
Mapre2	-1.07449739	3.410E-02	Downregulated
Fyco1	-1.07269908	9.170E-03	Downregulated

Gab2	-1.07240494	3.491E-02	Downregulated
Laptm5	-1.06723782	3.142E-03	Downregulated
Mob3a	-1.05992396	1.550E-02	Downregulated
Slc39a6	-1.05647804	3.473E-03	Downregulated
Galnt7	-1.05322438	2.438E-02	Downregulated
Pygb	-1.05301090	4.229E-02	Downregulated
Pacsin2	-1.04825350	4.026E-02	Downregulated
Sntb1	-1.04494632	4.081E-02	Downregulated
Afg3l2	-1.04448059	4.274E-02	Downregulated
Cap1	-1.02930127	9.799E-03	Downregulated
Fyn	-0.99564385	7.500E-03	Downregulated
6430548M08Rik	-0.99391624	1.691E-02	Downregulated
Slmap	-0.98478627	2.341E-02	Downregulated
Pkm	-0.98086535	4.495E-02	Downregulated
Gnaq	-0.98067302	4.095E-02	Downregulated
Mbnl2	-0.97071788	3.327E-02	Downregulated
Cds2	-0.96503107	3.025E-02	Downregulated
Cmtm6	-0.95635075	1.044E-02	Downregulated
Atp6v0a2	-0.93277941	4.488E-02	Downregulated
Tpm3	-0.93155495	2.999E-02	Downregulated
Peak1	-0.92852706	4.785E-02	Downregulated
Stim1	-0.90717340	4.219E-02	Downregulated
Arpc4	1.00084177	1.574E-02	Upregulated
Get3	1.01426751	3.287E-02	Upregulated
Psmb7	1.07021381	3.476E-02	Upregulated

Sf3b6	1.08152703	4.729E-02	Upregulated
Hras	1.08186632	2.929E-02	Upregulated
Eef1d	1.09761999	4.925E-02	Upregulated
Rps27	1.11252465	1.049E-02	Upregulated
Bsg	1.11531921	4.202E-02	Upregulated
Tmem234	1.12063090	1.069E-02	Upregulated
Isca2	1.12312763	2.938E-02	Upregulated
Nars	1.12336953	1.030E-02	Upregulated
Rpl12	1.12595387	2.510E-02	Upregulated
Naa38	1.12741784	4.170E-02	Upregulated
Dele1	1.12825925	8.547E-03	Upregulated
Elof1	1.12868819	3.519E-02	Upregulated
Nfs1	1.13240009	9.505E-03	Upregulated
Pck2	1.13325255	4.514E-02	Upregulated
Psip1	1.13364984	1.553E-02	Upregulated
Mrpl13	1.15984137	4.634E-02	Upregulated
Polr1d	1.17076032	1.489E-02	Upregulated
Vps29	1.17388730	2.603E-02	Upregulated
Polr1e	1.17398002	2.494E-02	Upregulated
Med28	1.17410508	7.090E-03	Upregulated
7SK	1.17865854	2.516E-02	Upregulated
Mrpl41	1.18306953	3.809E-02	Upregulated
Eef1b2	1.18881857	3.010E-02	Upregulated
Mrps14	1.19525340	4.183E-02	Upregulated
Ran	1.19741931	9.860E-03	Upregulated

2310022B05Rik	1.20030177	9.791E-03	Upregulated
Rbm3	1.20491369	2.228E-02	Upregulated
Atp5g3	1.20497556	4.589E-02	Upregulated
Sptssa	1.21763173	2.035E-02	Upregulated
Fam160b1	1.21781620	2.858E-03	Upregulated
Pcx	1.22512993	3.057E-02	Upregulated
Ndufaf4	1.22605464	4.523E-02	Upregulated
Rpl26	1.22694328	1.698E-02	Upregulated
Sec13	1.23769210	2.263E-02	Upregulated
Cd83	1.23934682	1.452E-02	Upregulated
Aimp2	1.24347366	3.041E-02	Upregulated
Rps8	1.24959494	6.647E-03	Upregulated
Snrpe	1.25791048	8.840E-03	Upregulated
Commd6	1.25950457	3.211E-03	Upregulated
Psmb4	1.26054365	1.304E-02	Upregulated
Cript	1.26748622	1.287E-03	Upregulated
Rps21	1.26793690	2.884E-02	Upregulated
Mrps21	1.26921286	4.570E-02	Upregulated
Rpl10	1.26966081	4.427E-02	Upregulated
Rmc1	1.27355548	2.252E-02	Upregulated
Wdr61	1.27649967	2.208E-02	Upregulated
Atp5pb	1.27740153	1.009E-02	Upregulated
Tbca	1.27814089	4.665E-02	Upregulated
Cenpx	1.27835776	4.166E-02	Upregulated
Ifrd2	1.28069517	1.512E-02	Upregulated

Pde6d	1.28108602	1.632E-02	Upregulated
Atp5k	1.28593814	2.807E-02	Upregulated
Rps11	1.28621133	3.615E-03	Upregulated
Gm24336	1.28907113	4.843E-02	Upregulated
Tceal8	1.29024042	1.055E-02	Upregulated
Cyba	1.29263404	2.060E-02	Upregulated
Gm7964	1.29456775	2.268E-02	Upregulated
Zfand2b	1.29541965	1.715E-02	Upregulated
Rps24	1.29781671	4.154E-02	Upregulated
Fau	1.30426304	3.199E-03	Upregulated
Btf3	1.30548269	1.331E-02	Upregulated
2410006H16Rik	1.31221780	1.707E-02	Upregulated
Rpl9-ps6	1.31296383	3.798E-02	Upregulated
Slirp	1.32034016	2.015E-02	Upregulated
Rpl13a	1.32304270	1.240E-02	Upregulated
Ndufb11	1.32793522	3.361E-02	Upregulated
Hint3	1.33355659	2.540E-03	Upregulated
Atp5h	1.33388562	3.803E-02	Upregulated
Polr2k	1.33401640	2.882E-02	Upregulated
Tomm5	1.33667832	1.981E-02	Upregulated
Rps16	1.33789821	9.600E-03	Upregulated
Acot13	1.34395636	1.148E-02	Upregulated
H2bu2	1.34937563	2.206E-02	Upregulated
Cdc34	1.35002095	8.168E-04	Upregulated
Tmem160	1.35354130	9.544E-03	Upregulated

Rps15	1.35611475	2.545E-03	Upregulated
Gm25117	1.35909915	1.564E-02	Upregulated
Calcoco1	1.36001721	2.475E-02	Upregulated
Uqcr11	1.36161606	1.871E-02	Upregulated
Gm12174	1.36344772	4.486E-02	Upregulated
Mrpl28	1.36448431	1.816E-02	Upregulated
Fam234b	1.36507816	1.554E-02	Upregulated
Hspe1	1.36573485	4.854E-03	Upregulated
Rpl27	1.37827705	8.467E-03	Upregulated
Rpl27a	1.38000798	5.062E-03	Upregulated
Hsd17b10	1.38996373	9.467E-03	Upregulated
1110051M20Rik	1.39362096	4.890E-02	Upregulated
Pitrm1	1.39416000	1.682E-03	Upregulated
7SK	1.39655664	4.711E-02	Upregulated
Bud31	1.39776907	2.107E-02	Upregulated
Uros	1.39857671	4.871E-02	Upregulated
Rpl32	1.41246854	4.731E-03	Upregulated
Chchd1	1.41268147	1.985E-03	Upregulated
Srp14	1.41875240	7.716E-03	Upregulated
Use1	1.42274446	3.700E-03	Upregulated
Zdhhc2	1.42558836	3.160E-02	Upregulated
Rps3a1	1.42704914	3.264E-02	Upregulated
Rps5	1.43333002	3.119E-03	Upregulated
Coa4	1.43645856	4.654E-02	Upregulated
Bcas2	1.43721705	5.769E-03	Upregulated

Rpl5	1.44433650	1.053E-03	Upregulated
Rpl36	1.44703139	1.751E-03	Upregulated
Rpl9	1.44719413	3.680E-03	Upregulated
Rpl22	1.44805968	8.378E-04	Upregulated
Pam16	1.45049950	2.835E-02	Upregulated
Rpsa	1.45077232	2.345E-03	Upregulated
Znhit1	1.45121759	3.952E-02	Upregulated
Igkv1-135	1.46039243	3.204E-02	Upregulated
S100a6	1.47160657	3.393E-02	Upregulated
Rps27l	1.47272056	6.376E-03	Upregulated
Atp5j2	1.47465322	1.576E-02	Upregulated
Tmem147	1.47741588	2.789E-03	Upregulated
Pcbd2	1.47907807	3.364E-02	Upregulated
Rps27rt	1.48469564	1.736E-02	Upregulated
Shank1	1.48699631	3.307E-02	Upregulated
Rdm1	1.48738421	1.430E-02	Upregulated
Car2	1.48790303	2.987E-02	Upregulated
Rps27a	1.49131245	3.465E-03	Upregulated
Pfdn1	1.49142067	2.401E-02	Upregulated
Polr2c	1.49199078	7.556E-04	Upregulated
Coa6	1.49613787	3.823E-02	Upregulated
Pcbp4	1.49875692	1.566E-02	Upregulated
Gale	1.49995053	1.666E-02	Upregulated
Apol11b	1.50323507	1.921E-02	Upregulated
Swi5	1.50943524	1.241E-03	Upregulated

Asxl3	1.51362577	4.921E-02	Upregulated
Gm9843	1.51927862	4.345E-03	Upregulated
Cox16	1.52621473	1.622E-02	Upregulated
Atp6v0b	1.52640018	3.764E-05	Upregulated
Rpl35	1.53362476	1.239E-02	Upregulated
Bola2	1.53384282	1.186E-03	Upregulated
Ssr4	1.53553115	1.461E-03	Upregulated
Trim10	1.54503279	3.884E-02	Upregulated
Uqcc2	1.54826802	6.492E-03	Upregulated
Gm2000	1.55180226	3.116E-02	Upregulated
Frg1	1.55825109	8.325E-03	Upregulated
Ndufb9	1.56271574	9.053E-03	Upregulated
Nqo1	1.56730366	1.314E-02	Upregulated
Pparg	1.56798383	3.663E-02	Upregulated
Ubl4a	1.57858871	8.247E-03	Upregulated
Sv2c	1.58019453	4.820E-02	Upregulated
Timm17b	1.58119173	4.759E-03	Upregulated
Sertad3	1.58830347	2.322E-02	Upregulated
BC028528	1.58903967	1.452E-02	Upregulated
Nuak1	1.58951875	4.238E-02	Upregulated
Rps18	1.60163485	6.579E-04	Upregulated
Cops9	1.60349490	6.848E-03	Upregulated
H4c18	1.60402250	8.561E-03	Upregulated
Serpini1	1.60497409	1.782E-03	Upregulated
Ndufs6	1.60724713	3.225E-03	Upregulated

Rps25	1.60764849	2.010E-04	Upregulated
Ndufs5	1.60807693	1.220E-02	Upregulated
Cnnm1	1.60855108	3.942E-02	Upregulated
Gpx4	1.61121910	2.281E-02	Upregulated
Dusp12	1.61168459	1.732E-02	Upregulated
Mir451a	1.61199818	3.162E-02	Upregulated
Cstb	1.61466214	9.789E-03	Upregulated
Uqcrh	1.62149796	1.849E-02	Upregulated
Ndufb4	1.62304005	3.500E-03	Upregulated
Timm8b	1.62818913	4.229E-03	Upregulated
Chac2	1.62826595	1.680E-02	Upregulated
Eif3i	1.62963949	1.202E-04	Upregulated
Gm28437	1.63099797	4.205E-02	Upregulated
Mtln	1.64037199	5.637E-03	Upregulated
Cks1b	1.64317139	4.358E-02	Upregulated
Chn1	1.64328903	1.399E-02	Upregulated
Hcfc1r1	1.64493923	3.637E-03	Upregulated
Snx21	1.64682366	4.928E-02	Upregulated
Tspo	1.64738431	4.549E-02	Upregulated
H2ac21	1.65463030	3.180E-02	Upregulated
BC035044	1.65718390	1.202E-03	Upregulated
Shmt2	1.65832572	4.285E-03	Upregulated
Gm5617	1.66098320	2.596E-02	Upregulated
Cenpq	1.66681854	1.427E-02	Upregulated
Prdx3	1.66793491	1.396E-02	Upregulated

Nsmce1	1.66991236	6.779E-04	Upregulated
Rps13	1.67029127	2.185E-04	Upregulated
Pcna-ps2	1.67461063	1.509E-02	Upregulated
Gm4737	1.67666434	4.046E-02	Upregulated
Atp5e	1.67792255	9.853E-03	Upregulated
Psmd10	1.68126533	2.335E-05	Upregulated
Smim4	1.68223254	1.686E-02	Upregulated
Milr1	1.68270939	1.648E-02	Upregulated
Nudt5	1.68430264	5.285E-04	Upregulated
Rpsa-ps10	1.68582907	6.214E-03	Upregulated
Hax1	1.68644685	2.802E-04	Upregulated
Nucb2	1.68654515	9.980E-03	Upregulated
Gm8186	1.68658087	3.913E-02	Upregulated
Josd2	1.69066470	3.151E-04	Upregulated
Hebp1	1.69658884	8.267E-04	Upregulated
Itgb1bp1	1.70113372	6.469E-06	Upregulated
Rps14	1.70241617	6.913E-03	Upregulated
K230015D01Rik	1.70557335	2.823E-02	Upregulated
Rack1	1.70766096	3.416E-04	Upregulated
Snora47	1.71113881	3.535E-02	Upregulated
Ndufa11	1.71148827	1.080E-02	Upregulated
Dnajc12	1.72117845	4.206E-03	Upregulated
Mpc2	1.72760926	6.582E-03	Upregulated
Map1b	1.72901126	4.168E-02	Upregulated
Snrpc	1.74204064	1.447E-04	Upregulated

Fmc1	1.74353401	3.795E-03	Upregulated
Rn7s1	1.74503273	5.602E-03	Upregulated
Gpx4-ps2	1.74869048	1.510E-02	Upregulated
Col27a1	1.75006123	2.603E-02	Upregulated
Ube2b	1.75407177	1.298E-03	Upregulated
Lage3	1.76309345	9.864E-04	Upregulated
Rps19	1.76449059	3.269E-04	Upregulated
Cox4i1	1.76677793	9.558E-04	Upregulated
Rps13-ps2	1.76726350	4.661E-03	Upregulated
Rps3	1.77429410	3.004E-04	Upregulated
Cbr1	1.78027790	5.201E-04	Upregulated
Tmem126a	1.78088468	4.665E-03	Upregulated
Rab9	1.78565616	4.443E-05	Upregulated
Gm14305	1.78666246	2.888E-02	Upregulated
Atp5o	1.78920415	3.857E-03	Upregulated
Nudt12	1.79435244	2.330E-02	Upregulated
Gm15501	1.79634726	8.304E-03	Upregulated
Prdx2	1.79817902	9.669E-03	Upregulated
Atg4a	1.79959076	4.786E-03	Upregulated
Fam83d	1.80164329	3.469E-02	Upregulated
A930006K02Rik	1.81321685	7.943E-03	Upregulated
Timm10	1.81456342	3.136E-05	Upregulated
Rps10-ps2	1.81962381	5.200E-03	Upregulated
Tmem205	1.82399939	2.334E-02	Upregulated
Tspo2	1.82817843	3.429E-02	Upregulated

Ifi27	1.83008085	6.290E-03	Upregulated
Asns	1.83104963	1.891E-02	Upregulated
Rpl22l1	1.83113998	2.253E-05	Upregulated
Isg15	1.83283810	4.832E-02	Upregulated
Cttnbp2nl	1.83443096	1.821E-02	Upregulated
Pet100	1.84193253	8.350E-03	Upregulated
Etl4	1.84380869	7.745E-03	Upregulated
Smim1	1.84422320	1.776E-02	Upregulated
Acsl6	1.84637898	1.611E-02	Upregulated
Ptprd	1.84655747	3.972E-02	Upregulated
1700007L15Rik	1.85013492	4.659E-02	Upregulated
Uxt	1.85190220	1.967E-03	Upregulated
Micos13	1.85936679	1.219E-04	Upregulated
Phyhip	1.86714175	1.958E-02	Upregulated
Slfn4	1.86841691	3.472E-02	Upregulated
Vamp5	1.87252414	4.988E-04	Upregulated
Gm45872	1.87284505	3.957E-02	Upregulated
Tlr7	1.87553008	2.586E-03	Upregulated
Nt5c3	1.87814846	6.805E-05	Upregulated
Lsm4	1.88188698	4.681E-04	Upregulated
Uqcr10	1.89142395	1.207E-03	Upregulated
Fpr2	1.89174865	4.612E-02	Upregulated
Ndufb2	1.89411035	8.920E-04	Upregulated
Rundc3a	1.89460924	3.090E-04	Upregulated
Gm10221	1.89856148	1.622E-02	Upregulated

Mrpl57	1.90380231	1.537E-04	Upregulated
Rn7s2	1.90600909	4.133E-03	Upregulated
Obscn	1.91497333	6.655E-03	Upregulated
Gm44510	1.91924887	3.761E-02	Upregulated
Cox7b	1.92138172	2.707E-03	Upregulated
Arl6	1.92466506	2.787E-02	Upregulated
Icam4	1.93596092	1.097E-02	Upregulated
Bbln	1.93662013	2.446E-03	Upregulated
Cenpw	1.95217501	4.458E-02	Upregulated
Ifi27l2a	1.95551827	3.619E-02	Upregulated
Rpl10a	1.96182634	1.833E-05	Upregulated
Smagp	1.96797462	4.434E-02	Upregulated
Prdx1	1.97888435	2.297E-04	Upregulated
Gm9945	1.98831979	1.853E-02	Upregulated
4933431K14Rik	1.99950773	9.763E-03	Upregulated
Lockd	2.00061701	2.636E-03	Upregulated
Uqcrq	2.00231044	1.462E-03	Upregulated
Gm23346	2.00733125	2.210E-02	Upregulated
Ogdhl	2.01131751	6.558E-03	Upregulated
Gm34961	2.01550541	2.378E-02	Upregulated
Aspa	2.02613297	1.543E-02	Upregulated
Grin2d	2.03428043	4.480E-03	Upregulated
Rbbp4	2.03586156	2.410E-04	Upregulated
Bend6	2.04306450	3.640E-02	Upregulated
Ndufa7	2.04685321	1.161E-03	Upregulated

Blvrb	2.05406696	3.530E-03	Upregulated
7SK	2.05544570	3.858E-03	Upregulated
Cox5b	2.06160700	6.607E-06	Upregulated
Cd177	2.06308360	3.468E-02	Upregulated
Fgf2	2.06716517	4.446E-02	Upregulated
Pls3	2.07405235	9.133E-04	Upregulated
Psma5	2.08142110	3.682E-04	Upregulated
Rexo2	2.09178090	4.355E-05	Upregulated
Gm867	2.09639227	1.083E-03	Upregulated
Gm5805	2.09825210	4.196E-02	Upregulated
Rpl34	2.10686876	3.656E-04	Upregulated
Nkain4	2.10755996	1.915E-02	Upregulated
Gpm6a	2.10773096	8.601E-03	Upregulated
Hint1	2.10891866	6.439E-05	Upregulated
Ryr1	2.11845187	2.348E-03	Upregulated
Gm15564	2.11960635	1.904E-02	Upregulated
Dnph1	2.12463238	3.694E-02	Upregulated
AA465934	2.12537551	1.121E-02	Upregulated
Hfe	2.12742935	7.341E-03	Upregulated
Apol11a	2.14388439	2.538E-03	Upregulated
Gngt2	2.15325882	2.418E-03	Upregulated
Gpi-ps	2.15924419	3.871E-02	Upregulated
Gm6212	2.15945553	7.312E-03	Upregulated
Wfdc17	2.16458955	9.419E-03	Upregulated
Mpst	2.17161354	2.056E-03	Upregulated

Gins2	2.18503881	1.495E-02	Upregulated
Phactr1	2.18725670	1.966E-02	Upregulated
Hs6st2	2.18834825	1.913E-02	Upregulated
Lancl1	2.19197452	2.948E-06	Upregulated
Ddt	2.19347936	1.116E-02	Upregulated
Gm37357	2.19567437	4.890E-02	Upregulated
Lama4	2.20041347	3.938E-02	Upregulated
Apol10c-ps	2.20057374	3.008E-02	Upregulated
Gm7335	2.20566991	4.413E-02	Upregulated
Rpl10a-ps1	2.21033887	2.557E-03	Upregulated
Ndufb8	2.21075153	1.847E-05	Upregulated
Uchl1	2.22231114	3.134E-02	Upregulated
Ms4a6c	2.23311155	7.345E-03	Upregulated
Pyurf	2.23612757	3.749E-07	Upregulated
Dctn3	2.24313629	2.443E-07	Upregulated
Tmtc1	2.24711197	2.709E-03	Upregulated
Lcn2	2.25046966	4.611E-02	Upregulated
Mrpl20	2.25213283	1.866E-05	Upregulated
Snrpd2	2.25220920	2.525E-06	Upregulated
Ifitm6	2.25438251	1.245E-02	Upregulated
Cldn13	2.25558752	1.620E-02	Upregulated
Snrnp25	2.25612850	1.096E-05	Upregulated
Cd59a	2.25934750	1.065E-02	Upregulated
Plac8	2.27501274	5.127E-03	Upregulated
Rps15a-ps7	2.27861722	4.035E-03	Upregulated

Ak1	2.28481167	3.246E-02	Upregulated
Ctsf	2.28500820	7.276E-05	Upregulated
Mrpl32	2.30303998	1.501E-05	Upregulated
Ighv1-26	2.31624422	5.400E-03	Upregulated
Il33	2.31811873	1.613E-02	Upregulated
Actn3	2.32329919	9.540E-03	Upregulated
Tppp3	2.33101172	1.066E-03	Upregulated
Pklr	2.33792409	1.377E-02	Upregulated
Gm22303	2.35660702	2.445E-02	Upregulated
Kcnab3	2.35715475	1.746E-02	Upregulated
Supt4a	2.35802971	3.259E-05	Upregulated
Vmn2r96	2.35826337	4.275E-02	Upregulated
Spire2	2.36444095	3.877E-02	Upregulated
Btf3-ps1	2.39224368	3.282E-02	Upregulated
Usp13	2.39516366	1.443E-02	Upregulated
B230317F23Rik	2.40030522	1.608E-02	Upregulated
Rps15a-ps5	2.40565531	3.485E-03	Upregulated
Lars2	2.40589548	1.215E-02	Upregulated
Scin	2.40949175	2.865E-02	Upregulated
Ect2l	2.41407251	2.324E-02	Upregulated
Oas1e	2.42298653	1.934E-02	Upregulated
Gm21188	2.42477601	2.102E-02	Upregulated
Gm49024	2.42782022	1.217E-02	Upregulated
Smc2os	2.44523283	2.455E-02	Upregulated
Gm29650	2.44764587	1.373E-02	Upregulated

Gm13375	2.45427431	2.518E-04	Upregulated
Sh2d1b1	2.45710953	7.795E-03	Upregulated
Myo18b	2.45746931	2.848E-02	Upregulated
Celf4	2.47654830	3.987E-02	Upregulated
Wnk2	2.47939597	4.042E-02	Upregulated
Tmem107	2.49243171	4.314E-02	Upregulated
Cox7a2	2.49631980	1.460E-05	Upregulated
Mvk	2.49637386	1.891E-04	Upregulated
Ulk4	2.49857149	3.383E-02	Upregulated
Map6	2.52272568	1.819E-02	Upregulated
Gm9484	2.52657720	4.352E-02	Upregulated
Gpnmb	2.53096913	4.565E-02	Upregulated
Abca13	2.53188949	3.408E-02	Upregulated
Rps15a-ps6	2.53752417	4.673E-05	Upregulated
Gpc3	2.55382457	1.095E-02	Upregulated
Acta1	2.55441821	1.102E-02	Upregulated
7SK	2.57338024	1.098E-02	Upregulated
4833417C18Rik	2.57607325	6.550E-03	Upregulated
Fcgr4	2.58049069	5.707E-03	Upregulated
Ttc26	2.59340293	2.970E-02	Upregulated
Gm37716	2.60189187	3.502E-02	Upregulated
Ndufb6	2.60326228	6.752E-05	Upregulated
Dclk1	2.60622600	2.688E-02	Upregulated
Mme	2.64825775	4.173E-02	Upregulated
Dctpp1	2.65730605	3.272E-04	Upregulated

Pdgfc	2.66364606	3.857E-02	Upregulated
Mpdz	2.67783988	3.638E-02	Upregulated
Fcrl5	2.67808803	9.965E-04	Upregulated
Alad	2.69215418	1.404E-07	Upregulated
Gm11870	2.69650454	3.391E-02	Upregulated
Myorg	2.70965232	1.942E-02	Upregulated
Atp13a5	2.71280708	3.407E-02	Upregulated
S100a9	2.71889154	4.898E-03	Upregulated
Gm1866	2.73581745	5.519E-03	Upregulated
Gm36161	2.74975670	8.583E-03	Upregulated
Gm4419	2.76071274	1.371E-02	Upregulated
Igkv1-133	2.76680579	2.088E-02	Upregulated
Rps18-ps6	2.78662443	3.300E-02	Upregulated
Bcl2a1d	2.79013036	5.478E-03	Upregulated
Fam229b	2.79029332	6.241E-04	Upregulated
Myom3	2.80343675	3.867E-02	Upregulated
Cdh11	2.81102564	1.369E-02	Upregulated
Cd302	2.82088132	4.019E-03	Upregulated
Thsd7a	2.82802066	2.325E-02	Upregulated
Tmem144	2.83085329	2.414E-02	Upregulated
Arhgef26	2.83678033	1.260E-02	Upregulated
Gnai1	2.84305028	2.224E-02	Upregulated
Sema4g	2.85015001	3.391E-02	Upregulated
Slc23a1	2.86039011	2.525E-02	Upregulated
Map2	2.86081033	1.913E-04	Upregulated

Rps7-ps3	2.86087375	3.057E-02	Upregulated
4930570G19Rik	2.86094790	2.323E-02	Upregulated
Nhs	2.86506569	3.274E-02	Upregulated
Sgpp2	2.86966945	3.245E-02	Upregulated
Megf11	2.87107338	1.153E-02	Upregulated
Wfdc21	2.88337804	2.552E-03	Upregulated
Gfra4	2.88594670	3.134E-02	Upregulated
Gm17354	2.89007078	6.116E-03	Upregulated
Gm17396	2.89333245	4.891E-02	Upregulated
Lilra5	2.89550393	2.496E-03	Upregulated
Fam135a	2.89551211	3.796E-04	Upregulated
Slamf9	2.89623720	5.819E-03	Upregulated
Fzd1	2.89681118	4.731E-04	Upregulated
Adgrb1	2.89780384	1.114E-02	Upregulated
Nr1h3	2.90433922	1.225E-03	Upregulated
Samd15	2.90836512	2.709E-02	Upregulated
Gm17244	2.91254530	4.938E-02	Upregulated
Ltf	2.91324219	1.615E-02	Upregulated
Pcdhb10	2.92066198	4.230E-02	Upregulated
4921504A21Rik	2.92397522	4.156E-02	Upregulated
Serpina3f	2.92674164	1.900E-02	Upregulated
Gm37769	2.93310300	4.274E-02	Upregulated
4930579K19Rik	2.93485851	3.458E-02	Upregulated
Egfl6	2.93593710	4.280E-02	Upregulated
Gm37060	2.93762417	3.972E-02	Upregulated

Cacng8	2.94795235	3.654E-02	Upregulated
Stk33	2.95024923	4.820E-02	Upregulated
Zkscan2	2.95642395	3.513E-02	Upregulated
Iqsec3	2.95849455	3.452E-02	Upregulated
Stk32a	2.95973392	3.540E-02	Upregulated
Shc2	2.96017547	3.440E-02	Upregulated
Myt1	2.96033548	3.455E-02	Upregulated
Ccdc87	2.96057784	4.675E-02	Upregulated
Elmod1	2.96127696	3.411E-02	Upregulated
C130075A20Rik	2.96229207	3.570E-02	Upregulated
Rab39b	2.96392669	3.436E-02	Upregulated
Slc19a3	2.96445286	4.029E-02	Upregulated
Prrg3	2.96658858	3.349E-02	Upregulated
Sphkap	2.96836887	3.307E-02	Upregulated
Lrtm2	2.96850052	3.379E-02	Upregulated
Pcdhb22	2.97008950	3.396E-02	Upregulated
Cobl	2.97020977	3.316E-02	Upregulated
Rps15a-ps3	2.97039795	3.237E-02	Upregulated
Abhd12b	2.97197850	4.590E-02	Upregulated
Amer3	2.97202517	3.332E-02	Upregulated
Gpr176	2.97219095	3.406E-02	Upregulated
Sgcd	2.97261651	3.381E-02	Upregulated
Gm19410	2.97267788	3.415E-02	Upregulated
Gm19439	2.97335784	3.427E-02	Upregulated
Adamtsl5	2.97438123	5.203E-04	Upregulated

Dusp15	2.97554392	3.297E-02	Upregulated
Olfm3	2.97585452	3.296E-02	Upregulated
Crym	2.97739584	3.294E-02	Upregulated
Gm42635	2.98162080	3.588E-02	Upregulated
Gm24727	2.98229790	3.752E-06	Upregulated
Efna3	2.98348470	3.286E-02	Upregulated
Pitx2	2.98374169	4.675E-02	Upregulated
Cldn11	2.98549844	3.283E-02	Upregulated
Aspg	3.00313142	4.125E-02	Upregulated
S100a8	3.00324423	1.206E-03	Upregulated
Mapk8ip1	3.00906163	2.184E-03	Upregulated
Nr2f2	3.01178936	7.013E-03	Upregulated
Gm45200	3.01266581	3.530E-02	Upregulated
Tmem232	3.01331052	3.999E-02	Upregulated
Stc1	3.01993522	3.246E-02	Upregulated
Gm14327	3.02829627	1.834E-02	Upregulated
Fras1	3.02831207	2.424E-02	Upregulated
Mettl5os	3.03228535	3.610E-02	Upregulated
Amn	3.03644942	4.515E-02	Upregulated
Gm32282	3.03709606	2.918E-02	Upregulated
Zkscan4	3.05236502	2.016E-02	Upregulated
Zfp105	3.05484732	1.210E-02	Upregulated
Diras1	3.05643971	1.861E-02	Upregulated
Gm42912	3.06031845	3.110E-02	Upregulated
Plcxd1	3.06456655	1.977E-02	Upregulated

Clmn	3.07034501	5.371E-03	Upregulated
Gm47693	3.08451563	3.579E-02	Upregulated
Rps10-ps1	3.08734131	5.910E-04	Upregulated
Gm5881	3.09076165	3.038E-02	Upregulated
Spink10	3.09590434	1.892E-02	Upregulated
Ccser1	3.09931531	9.438E-03	Upregulated
Hjv	3.10104679	6.816E-03	Upregulated
Vwa5b2	3.10267273	1.703E-02	Upregulated
Clmp	3.10995646	9.435E-03	Upregulated
Gm34776	3.12200339	3.241E-02	Upregulated
Cwh43	3.12671833	2.959E-02	Upregulated
Gm10941	3.13229444	3.586E-02	Upregulated
A130077B15Rik	3.13502902	3.237E-02	Upregulated
Dennd2a	3.14547714	1.516E-03	Upregulated
Casq2	3.14837790	1.708E-02	Upregulated
Tnfrsf11b	3.16571522	2.502E-02	Upregulated
BC034090	3.17412494	8.442E-03	Upregulated
Calcb	3.17524934	4.201E-02	Upregulated
Ocln	3.17901718	2.945E-02	Upregulated
Tmem132a	3.19590232	7.960E-03	Upregulated
Scel	3.19622940	2.062E-02	Upregulated
Hs6st3	3.19963859	1.786E-02	Upregulated
Snora20	3.20545932	2.321E-02	Upregulated
Cntnap3	3.20704220	3.579E-02	Upregulated
Cmya5	3.21486145	2.841E-05	Upregulated

Mid1-ps1	3.21666884	3.014E-02	Upregulated
Syt17	3.21886996	1.597E-02	Upregulated
D930020B18Rik	3.21941392	1.954E-02	Upregulated
9330111N05Rik	3.21951954	3.394E-02	Upregulated
Gm16287	3.22171869	3.425E-02	Upregulated
Mirg	3.22279956	1.620E-02	Upregulated
Gabrg3	3.22660166	1.541E-02	Upregulated
Ighv1-39	3.22702354	4.880E-03	Upregulated
Pde1a	3.22782238	1.559E-02	Upregulated
Gabrd	3.23290169	1.498E-02	Upregulated
Spock1	3.23336552	1.506E-02	Upregulated
Scarf2	3.23390638	1.830E-02	Upregulated
Cntn1	3.23947246	1.022E-02	Upregulated
Gm37297	3.24060814	1.399E-02	Upregulated
Gabbr2	3.24129924	1.493E-02	Upregulated
Pou3f1	3.25173109	1.462E-02	Upregulated
4632428C04Rik	3.25227563	1.549E-02	Upregulated
L3mbtl1	3.26926603	1.050E-02	Upregulated
2610001A08Rik	3.27348423	2.595E-02	Upregulated
Cutal	3.27479667	4.472E-02	Upregulated
Chil3	3.28219270	5.369E-04	Upregulated
Kansl2-ps	3.28552022	2.619E-02	Upregulated
6430710M23Rik	3.29184578	1.412E-02	Upregulated
Zfp558	3.30088294	2.365E-02	Upregulated
Gm52965	3.30953092	1.444E-02	Upregulated

Klhl41	3.31390494	3.288E-02	Upregulated
7SK	3.31945447	1.434E-02	Upregulated
Ngp	3.32448742	9.079E-03	Upregulated
Gm45104	3.32703127	4.182E-03	Upregulated
Gm49326	3.33194013	2.106E-02	Upregulated
Gm44067	3.33391812	1.189E-03	Upregulated
Klhl40	3.35696837	1.439E-02	Upregulated
Ccdc63	3.37597768	1.631E-02	Upregulated
Mir144	3.38430748	2.120E-03	Upregulated
Plxna4os1	3.38956781	1.634E-03	Upregulated
Astn1	3.39060610	6.108E-03	Upregulated
Syt2	3.39677512	6.405E-03	Upregulated
Tbx3	3.41872266	1.119E-02	Upregulated
Slco6c1	3.42580824	3.650E-02	Upregulated
Pnma3	3.42816432	8.189E-03	Upregulated
Shisa2	3.42943814	8.374E-03	Upregulated
Tcim	3.43534289	2.346E-02	Upregulated
Cdh20	3.44440862	7.374E-03	Upregulated
Neto1	3.44449944	7.503E-03	Upregulated
C730002L08Rik	3.44838890	8.786E-03	Upregulated
Grid2ip	3.44973917	8.089E-03	Upregulated
Mir5119	3.45086979	2.056E-02	Upregulated
Adcyap1r1	3.45461869	7.043E-03	Upregulated
Gm37897	3.45678834	1.503E-02	Upregulated
Cgref1	3.46183589	7.433E-03	Upregulated

Fgd1	3.46246964	7.251E-03	Upregulated
Klra13-ps	3.46788697	4.734E-03	Upregulated
Ankrd22	3.46805932	2.262E-02	Upregulated
Gm17276	3.47004712	7.294E-03	Upregulated
Jsrp1	3.47111683	5.711E-03	Upregulated
Gm11628	3.47922681	2.296E-02	Upregulated
Slc22a17	3.48305024	2.380E-03	Upregulated
Cxcl13	3.48359418	4.974E-02	Upregulated
Ttn	3.49208641	3.667E-06	Upregulated
Proca1	3.49801009	7.863E-03	Upregulated
Togaram2	3.49834686	9.825E-03	Upregulated
4933412O06Rik	3.50223605	8.935E-03	Upregulated
Peg3	3.52015756	3.859E-03	Upregulated
Dscam	3.52675884	3.787E-03	Upregulated
Rep15	3.53046948	2.268E-02	Upregulated
Eef1a2	3.53230097	3.695E-03	Upregulated
Tomm6os	3.53627624	2.451E-02	Upregulated
4732496C06Rik	3.53720629	4.200E-03	Upregulated
Tmem44	3.54133884	8.902E-04	Upregulated
Fam241b	3.54140866	2.804E-03	Upregulated
Gm14168	3.54515114	1.081E-02	Upregulated
Gdnf	3.55536810	2.622E-02	Upregulated
Des	3.56140151	5.360E-03	Upregulated
Gm38009	3.56259121	1.412E-02	Upregulated
Dkk3	3.58344721	1.570E-03	Upregulated

4930517E14Rik	3.58521873	1.704E-02	Upregulated
Gm9885	3.59493691	1.895E-02	Upregulated
3300005D01Rik	3.59654830	1.250E-02	Upregulated
Gm37166	3.60083348	6.960E-03	Upregulated
Rpsa-ps12	3.60091221	2.926E-02	Upregulated
Ms4a6d	3.60476719	7.242E-04	Upregulated
Col22a1	3.60662306	2.920E-02	Upregulated
Bcar1	3.62292938	4.032E-03	Upregulated
Hhipl1	3.62724843	3.870E-03	Upregulated
Olfm4	3.63948739	1.026E-05	Upregulated
Zfp385c	3.63974706	4.992E-03	Upregulated
Gm2999	3.64111119	2.482E-02	Upregulated
Pth1r	3.64135643	4.327E-03	Upregulated
Syn2	3.64430373	3.507E-03	Upregulated
Ntm	3.64479921	3.467E-03	Upregulated
Gm10912	3.64725050	1.643E-02	Upregulated
Pcdh18	3.65618539	3.689E-03	Upregulated
Nexn	3.66397096	3.568E-03	Upregulated
Kif17	3.67897898	2.315E-03	Upregulated
Pfn4	3.68021111	3.700E-03	Upregulated
Fhod3	3.68640811	2.339E-03	Upregulated
Lpin3	3.69886710	2.826E-02	Upregulated
Gm44806	3.70019342	6.706E-03	Upregulated
Gm30124	3.70650714	1.736E-02	Upregulated
Flnc	3.70864393	9.271E-04	Upregulated

Gm16793	3.70973797	5.004E-08	Upregulated
Hs3st3a1	3.71159852	1.334E-02	Upregulated
Pla2r1	3.72079903	3.197E-02	Upregulated
Mgst3	3.72402751	1.248E-06	Upregulated
Atp2a1	3.73159004	5.705E-04	Upregulated
Il20ra	3.73417438	6.319E-03	Upregulated
Gm5086	3.73539309	7.391E-03	Upregulated
Sod3	3.74165845	2.609E-03	Upregulated
A730063M14Rik	3.74497474	3.447E-03	Upregulated
Igfbp3	3.74647503	3.044E-03	Upregulated
Ankrd36	3.74934471	6.827E-03	Upregulated
Tmem72	3.79942583	1.719E-02	Upregulated
Grb14	3.80180686	1.938E-03	Upregulated
Itgb2l	3.82496784	7.075E-03	Upregulated
Tmem132e	3.83278763	1.790E-03	Upregulated
Gm9903	3.83543263	9.498E-03	Upregulated
Sntg2	3.85244244	1.853E-03	Upregulated
Gm18113	3.85559955	3.267E-02	Upregulated
Lpar3	3.85608655	3.009E-02	Upregulated
Gpr84	3.86402936	3.753E-02	Upregulated
Gm49417	3.89106728	1.193E-02	Upregulated
Pex11g	3.89245240	4.798E-03	Upregulated
Tc2n	3.89704379	1.039E-02	Upregulated
Myh4	3.91419332	9.508E-04	Upregulated
Hdhd3	3.91706038	1.726E-03	Upregulated

Gm42716	3.93827991	1.568E-02	Upregulated
Mrln	3.94977335	4.176E-02	Upregulated
Gm32051	3.95154722	3.352E-05	Upregulated
Galnt3	3.95600388	2.656E-03	Upregulated
Myh2	3.96539934	3.878E-03	Upregulated
Nrxn3	3.97059198	9.570E-04	Upregulated
Hmcn1	3.97246344	3.494E-03	Upregulated
1700047M11Rik	3.97967270	1.574E-03	Upregulated
Slc26a1	4.00099764	5.038E-04	Upregulated
6030407O03Rik	4.07376779	4.922E-03	Upregulated
Dcdc2a	4.08253052	6.282E-04	Upregulated
Tex11	4.08719661	1.358E-02	Upregulated
Slc6a12	4.09026141	3.813E-03	Upregulated
Gm48765	4.09127277	9.889E-03	Upregulated
Gm4841	4.09338763	2.782E-02	Upregulated
Arpin	4.09956851	5.529E-04	Upregulated
Postn	4.13933463	6.617E-03	Upregulated
Gm27252	4.16111037	2.119E-03	Upregulated
Rxfp2	4.16789586	5.577E-03	Upregulated
Ms4a7	4.17276262	1.021E-02	Upregulated
Gm12394	4.18537422	3.196E-02	Upregulated
Gm42928	4.20193812	2.801E-03	Upregulated
Plekhs1	4.20650657	3.928E-02	Upregulated
C1qtnf4	4.21089477	1.479E-03	Upregulated
Ighv4-1	4.25009807	1.842E-02	Upregulated

9130604C24Rik	4.25562103	5.312E-03	Upregulated
Gng13	4.28292843	1.102E-02	Upregulated
Inhba	4.29055074	7.368E-03	Upregulated
Dagla	4.29245880	1.537E-04	Upregulated
Lct	4.29255653	7.103E-03	Upregulated
Nacad	4.29725924	2.706E-04	Upregulated
Grin3b	4.30480097	7.302E-03	Upregulated
Plekhb1	4.32624561	2.187E-04	Upregulated
Tmem45a2	4.34006385	1.660E-02	Upregulated
Frmpd4	4.34877030	1.618E-04	Upregulated
Camp	4.37452537	7.605E-04	Upregulated
Hsd17b2	4.39008002	1.580E-02	Upregulated
Arg1	4.39535297	3.547E-02	Upregulated
Gm5499	4.41094864	4.257E-03	Upregulated
Gm47861	4.50505655	8.688E-03	Upregulated
Prok2	4.53408920	1.469E-02	Upregulated
Nol3	4.53703810	7.073E-05	Upregulated
Gm20110	4.60398807	7.577E-03	Upregulated
Capn12	4.62269782	1.001E-02	Upregulated
Asic1	4.64880026	3.766E-05	Upregulated
Pgam2	4.65015075	2.393E-03	Upregulated
Ffar3	4.72300078	3.892E-02	Upregulated
Gm30641	4.72646591	6.760E-03	Upregulated
Saa3	4.76050575	2.893E-02	Upregulated
Myh1	4.89095770	1.040E-06	Upregulated

Mmp13	4.97602155	4.379E-03	Upregulated
Oas1g	4.99211258	7.763E-03	Upregulated
4631405J19Rik	5.07596543	4.652E-02	Upregulated
Gstt1	5.17000535	8.235E-10	Upregulated
Gm26787	5.17579982	8.711E-04	Upregulated
Gm7324	5.23907204	5.915E-06	Upregulated
Arhgdig	5.36913066	2.287E-04	Upregulated
Tcap	5.40764050	2.508E-04	Upregulated
Gm10300	5.46319331	1.194E-02	Upregulated
Ighv5-16	5.48623220	4.712E-04	Upregulated
Tchh	5.52779961	1.295E-04	Upregulated
4930438A08Rik	5.52973228	8.558E-03	Upregulated
Scrn1	5.53537739	1.157E-04	Upregulated
Gm12609	5.64777025	2.262E-02	Upregulated
Erfe	5.65546835	1.994E-04	Upregulated
Tnfsf15	5.93445634	4.592E-02	Upregulated
Rapsn	5.94207806	3.568E-05	Upregulated
Upp1	6.05011223	5.307E-05	Upregulated

Table of Contents Top of Current Table

Supplemental Table 25. List of all significant (P < 0.05) differentially expressed genes in whole brain of *3xTg-AD* vehicle (week 8) versus wild-type B6129 vehicle (week 8) animals; 72 genes downregulated & 28 upregulated. Italicized genes have a recognized association with Alzheimer's disease (AD) pathology.

Gene Name	Identity/Role	Log2FoldChange	P-value	Regulation
Gm49980	LncRNA; unknown, upstream from Pik3c3 (phosphatidylinositol-3-kinase; autophagy & protein lipidation)	-21.8998093	1.34E-12	Downregulated
H2af-ps2	H2A histone family, pseudogene 2; nucleosome structure	-8.99746407	3.94E-05	Downregulated
H4c17	H4 clustered histone 17; nucleosome structure	-7.37836034	9.46E-06	Downregulated
Gm6166	Fatty acid binding protein 5, epidermal pseudogene; may predict lipid binding activity	-7.08481173	0.00095271	Downregulated
Gm13410	DEAD (Asp-Glu-Ala-Asp) box polypeptide 6 pseudogene; may be involved in pancreatic cancer	-6.48395328	0.00023842	Downregulated
Gm35082	LncRNA; unknown	-6.30889326	0.00026678	Downregulated
Eif3j2	Eukaryotic translation initiation factor 3, subunit J2; protein synthesis from the 40s ribosome, enables identical protein binding activity in the cytoplasm	-6.21671891	1.21E-14	Downregulated
Gm9008	Ring finger protein 26, retrotransposed (Rnf26rt); may enable ubiquitin protein ligase activity, protein localization to perinuclear region of cytoplasm	-6.19587158	0.00083808	Downregulated
Gm50350	LncRNA; unknown	-6.16264818	0.01405864	Downregulated
Gm3650	Pseudogene;	-6.12377956	0.00068657	Downregulated

	Unknown, upstream from Eif3j2 (see above)			
Gbp2b	Guanylate binding protein 2; interferon-induced protein that can bind to guanine nucleotides (GMP, GDP, & GTP)	-6.06188464	0.00247467	Downregulated
Gm7240	Pseudogene; unknown	-5.72847824	0.01717091	Downregulated
Rps3a3	Ribosomal protein S3A;<br>RNA binding and structural constituent of ribosome, component of the cytoplasmic ribosomal 40S subunit	-5.63606904	1.65E-05	Downregulated
Eno1b	Enolase 1b, retrotransposed; evolving pseudogene of enolase 1, widespread expression during embryonic development	-5.4700907	0.00417283	Downregulated
Gm13841	Pseudogene, orthologous to ribosomal protein L29 of the cytoplasmic ribosomal 60S subunit	-5.24968885	0.00805568	Downregulated
Gm6916	Pseudogene of coiled-coil domain containing 72; involved in cytoplasmic translation	-5.11239187	0.01302779	Downregulated
Gm20186	LncRNA; involved in response to wounding, upstream from LSM8 as a U6 small nucleolar RNA associate protein	-5.10420129	2.68E-08	Downregulated

Gm12312	Pseudogene of FGFR1 oncogene partner 2; involved in wound healing	-5.0403284	0.00099632	Downregulated
Tuba1c	Tubulin alpha 1c; enables GTP binding activity & structural constituent of the cytoskeleton	-4.96308288	4.25E-09	Downregulated
Gm11868	Pseudogene of cytochrome b5 reductase 4; involved in endoplasmic reticulum stress response and protection against reactive oxygen species buildup	-4.83411155	0.00018424	Downregulated
Gm6685	Pseudogene of cytochrome B5 domain-containing protein 1; binds heme under oxidizing conditions	-4.81017722	0.01293622	Downregulated
Gm3555	Pseudogene; unknown	-4.76169396	0.00110019	Downregulated
Rpl34-ps1	Ribosomal protein L34, pseudogene 1; ribosomal protein as a cytoplasmic component of the 60S subunit	-4.51536012	3.77E-05	Downregulated
Rec8	REC8 meiotic recombination protein; homologous recombination & chromosome synapsis	-4.50276556	0.00013231	Downregulated
Evi2	Ecotropic viral integration site 2; transmembrane protein to function as a cell surface receptor, positive regulation of granulocyte differentiation	-4.40045399	0.00096087	Downregulated

Tma7-ps	Translation machinery associated 7 homolog pseudogene; cytoplasmic translation	-4.3402753	2.38E-05	Downregulated
Capza1-ps1	Capping Protein (Actin Filament) Muscle Z-Line, Alpha 1 Pseudogene; F-actin binding and growth of actin filaments	-4.18397995	6.11E-07	Downregulated
Gm15459	Pseudogene of heat shock protein 8; chaperone of protein folding	-4.04683779	2.97E-05	Downregulated
Gm20075	Pseudogene of basic transcription factor 3-like 4; transcription	-3.95726669	0.03845495	Downregulated
Rnase6	Ribonuclease A Family Member K6; broad spectrum antimicrobial activity against pathogenic bacteria	-3.923827	0.00152764	Downregulated
Gm16092	LncRNA; unknown	-3.77046578	0.00468533	Downregulated
Rps13-ps1	Ribosomal protein S13 pseudogene; cytoplasmic ribosomal protein of the 40S subunit	-3.56850169	0.00046447	Downregulated
Ubb-ps	Ubiquitin B pseudogene; degradation of cellular proteins via 26S proteasome	-3.54945728	0.0015465	Downregulated
Cxcr5	C-X-C Motif Chemokine Receptor 5; binds to B-lymphocyte chemoattractant, B-cell migration	-3.50444572	0.01138474	Downregulated
Ankrd7	Ankyrin repeat domain 7; blastocyst hatching, in	-3.49163438	0.03545281	Downregulated

	centrosome & nucleoplasm			
A230056P14Rik	lncRNA; unknown	-3.47772166	0.02651132	Downregulated
Gm8130	Pseudogene of ribosomal protein S3; cytoplasmic & nuclear ribosomal protein of the 40S subunit	-3.35777404	0.04394441	Downregulated
4833407H14Rik	lncRNA, myocardial infarction associated transcript 1 (Mirt1); response to wounding	-3.25142982	0.04075686	Downregulated
Gm26881	lncRNA; may attenuate cell death and inflammation following myocardial infarction	-3.22451731	0.00355774	Downregulated
Zfp125	Zinc finger protein 125; Foxo1-inducible transcriptional repressor involved in lipoprotein structure, lipid binding & transport, may be involved in schizophrenia & memory impairment	-3.16937406	0.00130628	Downregulated
4933432K03Rik	lncRNA; unknown	-3.14889889	0.02858355	Downregulated
Ms4a1	Membrane Spanning 4-Domains A1, or CD20; development & differentiation of B-cells into plasma cells	-3.14868712	0.01956612	Downregulated
Osgin1	Oxidative Stress Induced Growth Inhibitor 1; regulates inflammation & cell death, involved in executive function and memory	-3.02731101	0.00653493	Downregulated

Gm8116	Pseudogene; unknown	-2.93933497	0.00377289	Downregulated
Ccr6	C-C Motif Chemokine Receptor 6; seven transmembrane protein, recruitment of dendritic & T cells for immunity	-2.87349352	0.00855086	Downregulated
Rps3a2	Ribosomal protein S3A2; Orthologous to human ribosomal protein S3A (RPS3A), cytoplasmic ribosomal protein of the 40S subunit	-2.81964543	0.00261599	Downregulated
C5ar2	Complement C5a Receptor 2; G-protein coupled receptor 1 family member involved in the complement system of the innate immune response	-2.81147111	0.00941902	Downregulated
Gm45844	Pseudogene; unknown	-2.658559	0.03258743	Downregulated
Pla2g4e	Phospholipase A2 Group IVE; regulation of membrane-tubule-mediated transport; candidate for resilience to AD	-2.57608189	3.58E-09	Downregulated
Gm10548	Pseudogene of ribosomal protein L29; cytoplasmic ribosomal protein of the 60S subunit	-2.54877717	0.00518799	Downregulated
Nlrc4	NLR Family CARD Domain Containing 4; caspase recruitment & formation of the inflammasome	-2.54604907	0.00979818	Downregulated
3110053B16Rik	LncRNA; unknown	-2.51819901	0.04356234	Downregulated
Gm26782	LncRNA; unknown	-2.46795054	0.01901529	Downregulated

Gm48678	LncRNA; unknown	-2.46016468	0.00388008	Downregulated
Clec3b	C-Type Lectin Domain Family 3 Member B; calcium signaling, platelet degranulation	-2.36411157	0.00114466	Downregulated
Ccl27a	C-C motif chemokine ligand 27A; positive regulation of T cell chemotaxis & actin cytoskeleton reorganization	-2.34473608	2.94E-36	Downregulated
Ubc	Ubiquitin C; polyubiquitin precursor for protein degradation	-2.27676951	0.00110893	Downregulated
Rpl3-ps1	Ribosomal protein L3, pseudogene 1; cytoplasmic ribosomal protein of the 60S subunit	-2.27652523	0.04089918	Downregulated
Myo7a	Myosin VIIA; binds actin, cellular motor movement, involved in non-syndromic hearing loss	-2.21531483	5.00E-05	Downregulated
Gm14292	Pseudogene of Wilms' tumor 1-associating protein; transcriptional and posttranscriptional regulation	-2.20364874	0.03149488	Downregulated
Xaf1	XIAP Associated Factor 1; negative regulation of the inhibitors of apoptosis	-2.1184126	0.00130901	Downregulated
Morf4l1-ps1	Mortality Factor 4 Like 1 Pseudogene 1; double-strand break repair via homologous recombination and histone modification	-2.06701933	0.01004768	Downregulated
Gm5898	Pseudogene of the thyroid hormone receptor associated	-2.00032094	0.04688543	Downregulated

	protein 3; thyroid hormone receptor binding, positive regulation of circadian rhythms			
Gm42756	lncRNA; unknown	-1.99851837	0.00175485	Downregulated
Serpina3n	Serine (or cysteine) peptidase inhibitor, clade A, member 3N; molecular chaperone, cytokine response	-1.88695136	0.02506104	Downregulated
Tagap	T Cell Activation RhoGTPase Activating Protein; guanyl-nucleotide exchange factor activity	-1.86544884	0.01220089	Downregulated
Il15	Interleukin 15; immune response, cell proliferation & differentiation, protective & anti-apoptotic	-1.68591204	0.01409431	Downregulated
Gm14418	Krüppel-associated box (KRAB) & zinc finger with C2H2 domain; DNA-binding transcription activity, RNA polymerase II specific	-1.41478328	0.01857114	Downregulated
Mrps12	Mitochondrial Ribosomal Protein S12; mitochondrial protein synthesis	-1.39618529	0.04692054	Downregulated
2900052N01Rik	lncRNA, lnc-290; B cell proliferation, activation, and differentiation	-1.38745033	0.02660802	Downregulated
Aqp6	Aquaporin 6; water-specific transport across the plasma membrane along osmotic gradient	-1.19802001	0.03332111	Downregulated
Pcdhgb6	Protocadherin Gamma Subfamily B, 6; calcium-dependent	-0.98020393	0.02723525	Downregulated

	cell adhesion, establishment & function of brain cell-cell connections			
Msl3	Male-Specific Lethal (MSL) Complex Subunit 3; chromatin remodeling & transcription regulation	0.84914042	0.03042235	Upregulated
Frmpd4	FERM And PDZ Domain Containing 4; positive regulation of dendritic spine morphogenesis and density, excitatory synaptic transmission	0.96419943	2.07E-08	Upregulated
Uba7	Ubiquitin Like Modifier Activating Enzyme 7; promyelocytic leukemia retinoic acid receptor alpha degradation & apoptosis; conjugation of interferon-stimulated gene 15; involved in intellectual disability	1.40573685	0.03576964	Upregulated
Gm42970	Putative piRNA (piR-22850)	1.47107628	0.02211182	Upregulated
ENSMUSG00000095041	AC149090.1, Phosphatidylserine Decarboxylase (PISD); catalyzes conversion of phosphatidylserine to phosphatidylethanolamine in the inner mitochondrial membrane, phospholipid metabolism	1.84938173	0.00643967	Upregulated
Bub1b	Budding Uninhibited By Benzimidazoles 1 Mitotic Checkpoint Serine/Threonine	2.14719692	0.01493231	Upregulated

	Kinase B; delays onset of anaphase and ensures proper chromosome segregation			
Ftl1-ps1	Ferritin light polypeptide 1, pseudogene 1; may be involved in intracellular iron storage	2.16563777	0.01545174	Upregulated
Exoc3l2	Exocyst Complex Component 3 Like 2; upregulated by VEGFA and governs cell membrane dynamics, mutations associated with AD	2.20433819	0.03387985	Upregulated
Usp18	Ubiquitin Specific Peptidase 18; cleave ubiquitin from ubiquitinated protein substrates	2.43954377	0.01825035	Upregulated
Ccr1	C-C Motif Chemokine Receptor 1; immunity with ligands as include macrophage inflammatory protein 1 alpha (MIP-1α), regulated on activation normal T expressed and secreted protein (RANTES), monocyte chemoattractant protein 3 (MCP-3), and myeloid progenitor inhibitory factor-1 (MPIF-1).	2.54902182	0.0124348	Upregulated
Col6a4	Collagen, type VI, alpha 4; enables collagen binding activity & involved in extracellular matrix organization	2.7744053	0.04153367	Upregulated

Gm5518	Pseudogene; unknown	2.81453729	0.01399876	Upregulated
Ly6d	Lymphocyte Antigen 6 Family Member D; marks the earliest stage of B-cell specification, paralog of Ly6/Neurotoxin 1 (LYNX1)	3.50585018	0.02263615	Upregulated
Apol11b	Apolipoprotein L 11b; enables chloride channel and lipid binding activity	3.5226559	0.00990964	Upregulated
Osm	Oncostatin M; member of the leukemia inhibitory factor/oncostatin-M (LIF/OSM) family of proteins & interleukin-6 (IL-6) family member, cytokine and growth regulator that inhibits proliferation of tumor cell lines	3.62968453	0.02815339	Upregulated
Plac8	Placenta Associated 8; positive regulation of cold-induced thermogenesis & positive regulation of transcription by RNA polymerase II, brown fat cell differentiation	3.6697891	0.03072898	Upregulated
Gm21188	Annexin A2 receptor 1 (Anxa2r1); enable signaling receptor activity, may induce osteoclast formation	3.78859161	0.03649573	Upregulated
Kcnn4	Potassium Calcium-Activated Channel Subfamily N Member 4; membrane hyperpolarization and Ca^{2+} influx	3.97235167	0.00753031	Upregulated
Ifi204	Interferon activated gene 204; enables double-stranded DNA	3.9898778	0.01900668	Upregulated

	binding activity & transcription coregulator activity, involved in cellular response to interferon-α; common marker of SARS-CoV-2 infection and AD			
Gm16439	BCL2/adenovirus E1B interacting protein 3 (BNIP3), pseudogene; pro-apoptosis	4.06436357	0.00515787	Upregulated
Gm26793	LncRNA; resides on fibroblast growth factor 15 (FGF15)	4.37876839	7.62E-05	Upregulated
Cxcl13	C-X-C Motif Chemokine Ligand 13 or "Angie"; B lymphocyte chemoattractant, B lymphocyte migration, a paralog of CXCL3	4.5585492	0.03085745	Upregulated
Gm7324	Pseudogene of RNA Binding Motif Protein X-Linked (RBMX); positive regulation of mRNA splicing (e.g., low density lipoprotein receptor, LDLR) via spliceosome, involved in intellectual development disorder	4.6543399	1.45E-07	Upregulated
Gm8730	Pseudogene of ribosomal protein, large, P0 (RPLP0); RPLP0 is a ribosomal protein of the component of the 60S subunit & is associated with AD	4.82931219	2.23E-06	Upregulated
Gm6278	Pseudogene of RPLP0; see above for Gm8730	4.9634558	0.00303561	Upregulated

Gm9625	Pseudogene; upstream from zinc finger protein 273 (Zfp273)	5.10097292	9.55E-06	Upregulated
Gm14165	Pseudogene of RPLP0; see above for Gm8730 & Gm6278	6.85295393	4.49E-05	Upregulated
Gm27177	LncRNA; unknown	32.9616958	4.72E-37	Upregulated

Table of Contents

Top of Current Table

Supplemental Table 26. List of all overlap of significant (P < 0.05) differentially expressed genes in both whole blood and brain of *3xTg-AD* vehicle (week 8) versus wild-type B6129 vehicle (week 8) animals. Italicized genes have a recognized association with Alzheimer's disease (AD) pathology.

**Common Genes Downregulated**	**Common Genes Upregulated**	**Common but Opposite: Downregulated in 3xTg-AD Blood & Upregulated in 3xTg-AD Brain versus B6129 vehicle**
Capza1-ps1; Capping Protein (Actin Filament) Muscle Z-Line, Alpha 1 Pseudogene; growth of actin filaments	Apol11b; Apolipoprotein L 11b; chloride channel & lipid binding	Col6a4; Collagen, type VI, alpha 4; extracellular matrix organization
Eif3j2; Eukaryotic translation initiation factor 3, subunit J2; protein synthesis	Bub1b; Budding Uninhibited By Benzimidazoles 1 Mitotic Checkpoint Serine/Threonine Kinase B; chromosome segregation	
Eno1b; Enolase 1b, retrotransposed evolving pseudogene of enolase 1; embryonic development	Gm14165; Pseudogene of ribosomal protein, large, P0 (RPLP0); protein synthesis, RPLP0 is associated with AD	
Gbp2b; Guanylate binding protein 2; interferon-induced, immunity	Gm16439; BCL2/adenovirus E1B interacting protein 3 (BNIP3), pseudogene, pro-apoptosis	
Gm10548; Pseudogene of ribosomal protein L29; protein synthesis	Gm6278; Pseudogene of RPLP0; see above for Gm14165	
Gm11868; Pseudogene of cytochrome b5 reductase 4; endoplasmic reticulum stress response, protection against reactive oxygen species	Gm7324; Pseudogene of RNA Binding Motif Protein X-Linked (RBMX); positive regulation of mRNA splicing (e.g., low density lipoprotein receptor, LDLR)	
Gm12312; Pseudogene of FGFR1 oncogene partner 2; wound healing	Gm8730; Pseudogene of RPLP0; see above for Gm14165 & Gm6278	
Gm15459;	Gm9625;	

Pseudogene of heat shock protein 8; protein folding	Pseudogene; within proximity of zinc finger protein 273 (Zfp273)
Gm16092;<br>lncRNA	
Gm20186;<br>lncRNA; wounding response	
Gm35082;<br>lncRNA	
Gm3650;<br>Pseudogene, in proximity of Eif3j2	
Gm49980;<br>lncRNA;<br>In proximity of Pik3c3 (phosphatidylinositol-3-kinase; autophagy & protein lipidation)	
Gm6916;<br>Pseudogene of coiled-coil domain containing 72; cytoplasmic translation	
Gm9008;<br>Ring finger protein 26, retrotransposed (Rnf26rt); ubiquitin protein ligase activity	
H2af-ps2;<br>H2A histone family, pseudogene 2;<br>nucleosome structure	
H4c17;<br>H4 clustered histone 17; nucleosome structure	
Mrps12;<br>Mitochondrial Ribosomal Protein S12; mitochondrial protein synthesis	
Rpl34-ps1;<br>Ribosomal protein L34, pseudogene 1; protein synthesis	
Rpl3-ps1;<br>Ribosomal protein L3, pseudogene 1; protein synthesis	
Rps13-ps1;<br>Ribosomal protein S13 pseudogene; protein synthesis	

Rps3a3;<br>Ribosomal protein S3A;<br>protein synthesis
Tma7-ps;<br>Translation machinery associated 7 homolog pseudogene; cytoplasmic translation
Ubb-ps;<br>Ubiquitin B pseudogene;<br>protein degradation
Ubc;<br>Ubiquitin C; protein degradation

Supplemental Table 27. List of all significant (P < 0.05) differentially expressed genes in whole brain of *3xTg-AD* CBD versus *3xTg-AD* vehicle animals; 5 genes downregulated & 38 upregulated. Italicized genes have a recognized association with Alzheimer's disease (AD) pathology; *Prior recognition for sensitivity to CBD treatment.

Gene Name	Identity/Role	Log2FoldChange	P-value	Regulation
Rag2	V(D)J Recombination-Activating Protein 2; develops B & T lymphocytes	-11.1552185	0.01422445	Downregulated
Gm27177	LncRNA; unknown	-10.7853403	3.72E-06	Downregulated
Mrps36-ps1	Mitochondrial ribosomal protein S36 (Mrps36), pseudogene 1; Mrps36 is also known as Alpha-Ketoglutarate Dehydrogenase Subunit 4 (KGD4), consists of a small 28S subunit and a large 39S subunit, involved in 2-oxoglutarate metabolic process	-3.43368106	0.03285365	Downregulated
Pilrb2	Paired Immunoglobin Like Type 2 Receptor Beta, also known as PILRB; immunity, associates with immunoreceptor tyrosine-based activation motif (ITAM) molecules on the cell surface	-3.1737281	0.02304744	Downregulated
1600010M07Rik	LncRNA; unknown	-2.90547654	0.04991688	Downregulated
Gbp3	Guanylate Binding Protein 3; Interferon (IFN)-inducible GTPase that plays important roles in innate immunity	1.62110803	0.00785419	Upregulated
*Rsad2**	Radical S-Adenosyl Methionine Domain Containing 2; interferon-inducible antiviral protein of the S-adenosyl-L-methionine (SAM) superfamily of enzymes, innate immunity,	1.67640876	0.017952	Upregulated

	antiproliferative activity in T-cells			
Nlrc5	NOD-like receptor (NLR) family caspase recruitment domain (CARD) domain containing 5; cytokine response & antiviral immunity	1.8865627	0.00284546	Upregulated
Phf11b	Plant homeodomain (PHD) Finger Protein 11; positive regulation of Th1-type cytokine gene expression	1.95000507	0.00775493	Upregulated
Ifi209	Interferon activated gene 209 with human ortholog as interferon gamma inducible protein 16 (IFI16); <u>may interact with the apolipoprotein E2 allele</u>	2.00711644	0.00694702	Upregulated
*Oas1b**	2'-5'-Oligoadenylate Synthetase 1; innate cellular antiviral response, cell growth & apoptosis; genetic variants associated with AD & in some cases linked to severe SARS-CoV-2 infection	2.05386459	0.00921522	Upregulated
Bst2	Bone Marrow Stromal Cell Antigen 2; growth & development of B-cells, maybe be enriched in reactive astrocytes and associated with glioma & multiple sclerosis	2.05596679	0.00652201	Upregulated
Gm5526	Pseudogene; unknown	2.2055758	0.0304474	Upregulated
Irf7	Interferon Regulatory Factor 7; transcriptional activation of virus-inducible cellular genes, such as interferon beta chain genes, shared among AD& SARS-CoV-2	2.23655886	0.02667674	Upregulated

Usp18	Ubiquitin Specific Peptidase 18; cleave ubiquitin from ubiquitinated protein substrates, upregulated with exercise during AD	2.2384876	0.00673232	Upregulated
Iigp1	Interferon inducible GTPase 1; involved in defense response to Gram-negative bacterium, regulation of autophagy	2.25680851	0.01163375	Upregulated
Ifi27l2a	Interferon, alpha-inducible protein 27 like 2A; acts upstream of or within aging and response to virus, pro-apoptotic, located in mitochondrial membrane, human orthologs include interferon alpha inducible protein 27 like 2 (IFI27L2)	2.29889884	0.0106573	Upregulated
Zbp1	Z-DNA Binding Protein 1; innate immune response & type-I interferon production	2.38053694	0.02861004	Upregulated
Oasl2	2'-5'-Oligoadenylate Synthetase Like 2; interleukin-27-mediated signaling pathway, negative regulation of viral genome replication, and positive regulation of retinoic acid-inducible gene I (RIG-I) signaling pathway	2.4199821	0.04899194	Upregulated
*Oas2**	2'-5'-Oligoadenylate Synthetase 2; innate cellular antiviral response, cell growth & apoptosis; associated with AD & SARS-CoV-2 infection	2.46707829	0.04115153	Upregulated
Ifit1	Interferon Induced Protein With Tetratricopeptide Repeats 1; inhibits viral	2.53496588	0.00073252	Upregulated

	replication translational initiation, paralog of IFIT1B			
Isg15	Interferon-stimulated protein, 15KDa, Ubiquitin Like Modifier; chemotactic activity towards neutrophils, direction of ligated target proteins to intermediate filaments, cell-to-cell signaling, & antiviral activity, upregulated with exercise during AD	2.60858905	0.00177691	Upregulated
*Oasl1**	2'-5' oligoadenylate synthetase-like 1; double-stranded RNA binding activity, acts upstream of or within interleukin-27-mediated signaling pathway, orthologous to human OASL, associated with AD and/or SARS-CoV-2 infection	2.69959902	0.01605691	Upregulated
H2-Q6	Histocompatibility 2, Q region locus 6; 14-3-3 protein, transporter associated with antigen processing (TAP) & signaling receptor binding activity, human orthologs include HLA-E, HLA-F, & HLA-G	2.74740632	0.00270784	Upregulated
H2-Q7	Histocompatibility 2, Q region locus 7; 14-3-3 protein, transporter associated with antigen processing (TAP) & signaling receptor binding activity, human orthologs include HLA-E, HLA-F, & HLA-G	2.9170156	4.49E-05	Upregulated
Mx1	Myxovirus Dynamin Like GTPase 1; cellular antiviral response, associated with AD and SARS-CoV-2 infection	2.96383319	0.00262559	Upregulated

Il18r1	Interleukin 18 Receptor 1; binding of the pro-inflammatory cytokine IL18, but not IL1A nor IL1B	2.98981091	0.02274497	Upregulated
Oas3*	2'-5'-oligoadenylate synthetase 3; induced by interferons and catalyzes the 2', 5' oligomers of adenosine in order to bind and activate RNase L, inhibition of cellular protein synthesis and viral infection resistance	3.00045718	0.00092426	Upregulated
Gm30411	LncRNA; unknown	3.06798463	0.02813666	Upregulated
Ly6c2	Lymphocyte antigen 6 family member C2; acetylcholine receptor inhibitor activity, ortholog of human LY6H, reduced by amyloid β during AD	3.21707033	0.0206694	Upregulated
G430095P16Rik	LncRNA; unknown	3.22020433	0.03687573	Upregulated
Rufy4	RUN & FYVE Domain-Containing Protein 4; phosphatidylinositol-3-phosphate binding activity, autophagy	3.2249072	0.04019921	Upregulated
Epb42	Erythrocyte membrane protein band 4.2; erythrocyte shape and mechanical property regulation	3.34655051	0.0135967	Upregulated
Klrb1b	Killer cell lectin-like receptor subfamily B member 1B; Acts upstream of or within negative regulation of natural killer cell mediated cytotoxicity, ortholog of human KLRB1	3.42453072	0.04430671	Upregulated
Gm12185	Predicted gene 12185; involved in cellular response to interferon-beta and defense response	3.46679933	0.01434039	Upregulated

Ifi44	Interferon induced protein 44; immunity	3.55246652	0.00295076	Upregulated
Ifi206	Interferon activated gene 206; regulation of cysteine-type endopeptidase activity, regulation of gene expression, & and regulation of innate immune response, ortholog of human IFI16; may interact with the apolipoprotein E2 allele	3.57029299	0.00425405	Upregulated
Pigr	Polymeric Immunoglobulin Receptor; mediates selective transcytosis of polymeric IgA and IgM across mucosal epithelial cells	3.85993707	0.03860169	Upregulated
Igkv12-46	Immunoglobulin kappa variable 12-46; immunity	3.90141146	0.00170527	Upregulated
*Ccl5**	C-C Motif Chemokine Ligand 5; immunoregulation & inflammation, release of histamine from basophils and activates eosinophils, ligand of CC receptor 5 (CCR5)	4.96302762	8.43E-05	Upregulated
*Cxcl10**	C-X-C motif chemokine ligand 10; binds to CXCR3 & stimulates monocytes, natural killer and T-cell migration, and modulation of adhesion molecule expression, key regulator of the 'cytokine storm' immune response to SARS-CoV-2 infection	5.09837018	0.00240486	Upregulated
Dntt	DNA nucleotidylexotransferase; member of the DNA polymerase type-X family, generates antigen receptor diversity by synthesizing non-germ	14.6876652	3.32E-09	Upregulated

	line elements (N-regions) at the junctions of rearranged Ig heavy chain and T cell receptor gene segments			
Gm49980	LncRNA; unknown	16.4882161	1.22E-08	Upregulated

Supplemental Table 28. List of all overlap of significant (P < 0.05) differentially expressed genes in both whole blood and brain of 3xTg-AD CBD (week 8) versus 3xTg-AD vehicle (week 8) animals.

**Common but Opposite: Downregulated in 3xTg-AD CBD Blood & Upregulated in 3xTg-AD CBD Brain versus 3xTg-AD vehicle**
Gm49980; LncRNA; In proximity of Pik3c3 (phosphatidylinositol-3-kinase; autophagy & protein lipidation)

Table of Contents

Supplemental Table 29. List of all significant (P < 0.05) differentially expressed genes in whole brain of wild-type B6129 CBD versus wild-type B6129 vehicle animals; 7 downregulated genes & 10 upregulated genes.

Gene Name	Identity/Role	Log2FoldChange	P-value	Regulation
Gm8670	Twist family bHLH transcription factor (TWIST) neighbor pseudogene; unknown	-3.85585945	0.04388542	Downregulated
2610206C17Rik	RIKEN cDNA 2610206C17 gene; lncRNA	-3.45151822	0.03272903	Downregulated
Cxcr5	C-X-C Motif Chemokine Receptor 5; binds to B-lymphocyte chemoattractant, B-cell migration	-3.42884542	0.01369637	Downregulated
Mid1-ps1	Midline 1, pseudogene 1; Mid1 is broadly associated with cancer & neurodegenerative disease	-3.3513001	0.03871813	Downregulated
Gm38431	LncRNA; represents a read-through transcript composed of ribonucleoprotein, PTB binding 1 (Raver1) and ferredoxin 2 (Fdx1l) sequence	-2.45727324	0.04330679	Downregulated
Gm24727	Predicted gene, 24727; unknown	-1.98438765	0.02934841	Downregulated
Mid1	Midline 1; a member of the tripartite motif (TRIM) family, also known as the 'RING-B box-coiled coil' (RBCC) subgroup of RING finger proteins, formation of multiprotein structures acting as anchor points to microtubules; broadly associated with cancer & neurodegenerative disease	-1.43949516	0.00487552	Downregulated
Nrm	Nurim; shares homology with isoprenylcysteine	1.77314549	0.02497355	Upregulated

	carboxymethyltransferase enzymes			
Lax1	Lymphocyte transmembrane adaptor 1; SH2 domain & protein kinase binding activity, involved in B-cell activation, negative regulation of MAP kinase activity, & negative regulation of T cell activation	2.78588943	0.04593249	Upregulated
Slamf6	SLAM family member 6; of the CD2 subfamily of the immunoglobulin superfamily, a coreceptor in the process of Natural Killer (NK) cell activation	2.79494329	0.04980721	Upregulated
Igkc	Immunoglobulin kappa constant; antigen & immunoglobulin receptor binding activity, involved in retina homeostasis	2.87894631	0.03792317	Upregulated
Gm26793	LncRNA; resides on fibroblast growth factor 15 (FGF15)	3.31001592	0.00500883	Upregulated
Il2rg	Interleukin 2 receptor subunit gamma; signaling component of many interleukin receptors, including those of interleukin -2, -4, -7 and -21, referred to as the common gamma chain	3.45377526	0.02858967	Upregulated
Igkv12-41	Immunoglobulin kappa chain variable 12-41; act upstream of or within adaptive immune response, ortholog of human IGKV1-27	3.49262364	0.03240489	Upregulated
Gm9625	Pseudogene; upstream from zinc finger protein 273 (Zfp273)	3.75177526	0.00193368	Upregulated
Gm8730	Pseudogene of ribosomal protein, large, P0 (RPLP0); RPLP0 is a	3.89893975	0.00022242	Upregulated

	ribosomal protein of the component of the 60S subunit & is associated with AD			
Gm14165	Pseudogene of RPLP0; see above for Gm8730	6.27434542	0.00021422	Upregulated

Supplemental Table 30. List of all overlap of significant ($P < 0.05$) differentially expressed genes in both whole blood and brain of wild-type B6129 CBD (week 8) versus B6129 vehicle (week 8) animals.

**Common Genes Upregulated**
Gm14165;<br>Pseudogene of ribosomal protein, large, P0 (RPLP0); protein synthesis, RPLP0 is associated with AD
Gm8730;<br>Pseudogene of RPLP0; see above for Gm14165
Gm9625;<br>Pseudogene; within proximity of zinc finger protein 273 (Zfp273)

Table of Contents

Supplemental Table 31. List of all significant (P < 0.05) differentially expressed genes in whole brain of 3xTg-AD CBD versus wild-type B6129 CBD animals; 73 genes downregulated & 98 upregulated genes. Italicized genes have a recognized association with Alzheimer's disease (AD) pathology; *Prior recognition for sensitivity to CBD treatment.

Gene Name	Identity/Role	Log2FoldChange	P-value	Regulation
Rag2	V(D)J Recombination-Activating Protein 2; develops B & T lymphocytes	-12.6610633	0.00497159	Downregulated
H4c17	H4 clustered histone 17; nucleosome structure	-8.53376288	7.55E-08	Downregulated
H2af-ps2	H2A histone family, pseudogene 2; nucleosome structure	-7.58321805	0.00013181	Downregulated
Gm43305	LncRNA; unknown, aldosterone-induced	-7.39226502	0.00797284	Downregulated
Gm35082	LncRNA; unknown	-7.24819869	3.06E-06	Downregulated
Tdg-ps2	Thymine DNA glycosylase, pseudogene 2	-6.98017569	0.00872547	Downregulated
Gm20186	LncRNA; involved in response to wounding, upstream from LSM8 as a U6 small nucleolar RNA associate protein	-6.92314072	6.16E-09	Downregulated
Gm49980	LncRNA; unknown, upstream from Pik3c3 (phosphatidylinositol-3-kinase; upstream of/within autophagy & protein lipidation)	-6.75980092	0.01932761	Downregulated
Rps3a3	Ribosomal protein S3A; RNA binding and structural constituent of ribosome, component of the cytoplasmic ribosomal 40S subunit	-6.68316432	2.62E-09	Downregulated
Gm9008	Ring finger protein 26, retrotransposed; may enable ubiquitin protein ligase activity, protein localization to perinuclear region of cytoplasm	-6.6110698	0.00010916	Downregulated

Zfp990	Zinc finger protein 990; regulation of transcription by RNA polymerase II	-6.55788744	0.03130775	Downregulated
Gm13410	DEAD (Asp-Glu-Ala-Asp) box polypeptide 6 pseudogene; may be involved in pancreatic cancer	-6.44836988	8.62E-05	Downregulated
Gm6166	Fatty acid binding protein 5, epidermal pseudogene; may predict lipid binding activity	-6.18469398	0.00085522	Downregulated
Gm3650	Pseudogene; Unknown, upstream from Eif3j2	-5.85675088	0.00039526	Downregulated
Gm7240	Pseudogene; unknown	-5.6642435	0.00834279	Downregulated
Eif3j2	Eukaryotic translation initiation factor 3, subunit J2; protein synthesis from the 40s ribosome, enables identical protein binding activity in the cytoplasm	-5.59697072	1.44E-13	Downregulated
Gm6916	Pseudogene of coiled-coil domain containing 72; involved in cytoplasmic translation	-5.54745797	0.00257299	Downregulated
Gm16092	LncRNA; unknown	-5.31011974	0.00013257	Downregulated
Gm50350	LncRNA; unknown	-5.27100565	0.02026238	Downregulated
Gm11868	Pseudogene of cytochrome b5 reductase 4; involved in endoplasmic reticulum stress response and protection against reactive oxygen species buildup	-5.13973846	3.12E-05	Downregulated
Gm5801	Ubiquitin-conjugating enzyme E2, J2 homolog pseudogene	-4.93520785	0.00274821	Downregulated
Eno1b	Enolase 1b, retrotransposed; evolving pseudogene of enolase 1, widespread expression during embryonic development	-4.88452126	0.00359254	Downregulated

Rec8	REC8 meiotic recombination protein; homologous recombination & chromosome synapsis	-4.7910754	4.46E-06	Downregulated
Gm6685	Pseudogene of cytochrome B5 domain-containing protein 1; binds heme under oxidizing conditions	-4.76994373	0.0066921	Downregulated
Gbp2b	Guanylate binding protein 2; interferon-induced protein that can bind to guanine nucleotides (GMP, GDP, & GTP)	-4.76810362	0.01090576	Downregulated
Gm3555	Serine/threonine kinase 38 like (STK38L) pseudogene; STK38L has ATP binding & magnesium ion binding activity, protein serine/threonine kinase activity	-4.72314751	0.00028356	Downregulated
Gm12312	FGFR1 oncogene partner 2 FGFR1OP2) pseudogene; FGFR1OP2 is involved in wound healing	-4.66485562	0.00028677	Downregulated
Gm13841	Pseudogene, orthologous to ribosomal protein L29 of the cytoplasmic ribosomal 60S subunit	-4.55739286	0.01281374	Downregulated
Rps13-ps1	Ribosomal protein S13 pseudogene; cytoplasmic ribosomal protein of the 40S subunit	-4.35251361	5.64E-07	Downregulated
Capza1-ps1	Capping Protein (Actin Filament) Muscle Z-Line, Alpha 1 Pseudogene; F-actin binding and growth of actin filaments	-4.29627552	7.83E-09	Downregulated
Tma7-ps	Translation machinery associated 7 homolog pseudogene; cytoplasmic translation	-4.15174217	2.36E-05	Downregulated
Gm14287	Pseudogene; unknown	-3.88996037	0.01217946	Downregulated

Gm20075	Pseudogene of basic transcription factor 3-like 4; transcription	-3.84745101	0.02319227	Downregulated
Tuba1c	Tubulin alpha 1c; enables GTP binding activity & structural constituent of the cytoskeleton	-3.79086377	9.89E-07	Downregulated
Gm15459	Pseudogene of heat shock protein 8; chaperone of protein folding	-3.7633964	1.03E-05	Downregulated
Gm28403	Pseudogene; unknown	-3.74542819	0.0414225	Downregulated
Gm10548	Pseudogene of ribosomal protein L29; cytoplasmic ribosomal protein of the 60S subunit	-3.71829654	6.02E-05	Downregulated
Trim34b	Tripartite Motif Containing 43B; ubiquitin protein ligase activity, innate immunity	-3.66957871	0.00470664	Downregulated
Gm8116	Pseudogene; unknown	-3.64573824	0.00051928	Downregulated
Rpl34-ps1	Ribosomal protein L34, pseudogene 1; ribosomal protein as a cytoplasmic component of the 60S subunit	-3.55530846	0.0003627	Downregulated
Igkv12-41	Immunoglobulin kappa chain variable 12-41; act upstream of or within adaptive immune response, ortholog of human IGKV1-27	-3.42866215	0.01027682	Downregulated
Gm35021	LncRNA; unknown	-3.42446139	0.01067126	Downregulated
Gm16548	LncRNA; unknown	-3.37040163	0.0243256	Downregulated
Gm26881	LncRNA; unknown	-3.32612104	0.00056394	Downregulated
Ubb-ps	Ubiquitin B pseudogene; degradation of cellular proteins via 26S proteasome	-3.27310844	0.00098231	Downregulated
Gm8524	Histone H3.3-like pseudogene; nucleosome structure of the chromosomal fiber	-3.21084674	0.02475622	Downregulated
Rps3a2	Ribosomal protein S3A2; Orthologous to human	-3.14831873	7.23E-05	Downregulated

	ribosomal protein S3A (RPS3A), cytoplasmic ribosomal protein of the 40S subunit			
Dynlt2a3	Dynein light chain Tctex-type 2A3;	-3.06218517	0.03816571	Downregulated
Zfp125	Zinc finger protein 125; Foxo1-inducible transcriptional repressor involved in lipoprotein structure, lipid binding & transport, may be involved in schizophrenia & memory impairment	-3.0580949	0.00041555	Downregulated
Xlr4b	X-linked lymphocyte-regulated 4B; positive regulation of dendritic spine morphogenesis and positive regulation of synapse assembly, ortholog of human family with sequence similarity 9 member B (FAM9B) & FAM9C	-3.02136312	0.00546424	Downregulated
Slc6a20b	Solute carrier family 6 (neurotransmitter transporter), member 20B; L-proline transmembrane transporter, amino-acid betaine transmembrane transporter; and proline:sodium symporter activity, amino-acid betaine and cation transport, ortholog of human SLC6A20	-2.8330196	0.04742955	Downregulated
Ccr6	C-C Motif Chemokine Receptor 6; seven transmembrane protein, recruitment of dendritic & T cells for immunity	-2.55623878	0.00342502	Downregulated
Gm20383	LncRNA; unknown	-2.43289251	0.01496405	Downregulated
Ccl27a	C-C motif chemokine ligand 27A; positive regulation of T cell chemotaxis & actin	-2.28661162	2.37E-41	Downregulated

	cytoskeleton reorganization			
Gm48678	LncRNA; unknown	-2.21488401	0.00437731	Downregulated
Gm26782	LncRNA; unknown	-2.15153175	0.02460143	Downregulated
Serpina3n	Serine (or cysteine) peptidase inhibitor, clade A, member 3N; molecular chaperone, cytokine response	-2.13137127	0.00211725	Downregulated
Gm10074	U6 snRNA-associated Sm-like protein (LSm5); pre-miRNA splicing	-2.1257176	0.01907219	Downregulated
9030025P20Rik	Endoplasmic reticulum (ER) membrane associated RNA degradation like 2 (Ermardl2 or Ermard); neuronal migration	-2.10260111	0.02642957	Downregulated
Gm5898	Pseudogene of the thyroid hormone receptor associated protein 3; thyroid hormone receptor binding, positive regulation of circadian rhythms	-2.08961125	0.0183461	Downregulated
Gm14292	Pseudogene of Wilms' tumor 1-associating protein; transcriptional and posttranscriptional regulation	-2.05768601	0.0252093	Downregulated
Akr1c14	Aldo-keto reductase family 1, member C14; androsterone dehydrogenase activity, involved in glycoside, progesterone, and prostaglandin metabolic process, ortholog of human AKR1C1/C2/C3	-2.04440839	0.00834004	Downregulated
Pla2g4e	Phospholipase A2 Group IVE; regulation of membrane-tubule-mediated transport; candidate for resilience to AD	-2.00748922	1.62E-06	Downregulated

Clec3b	C-Type Lectin Domain Family 3 Member B; calcium signaling, platelet degranulation	-1.97569996	0.00347118	Downregulated
4930481B07Rik	LncRNA; unknown	-1.97421273	0.01776139	Downregulated
Morf4l1-ps1	Mortality Factor 4 Like 1 Pseudogene 1; double-strand break repair via homologous recombination and histone modification	-1.8596927	0.01175496	Downregulated
Gm5089	LncRNA; unknown	-1.80019353	0.02211007	Downregulated
Tagap	T Cell Activation RhoGTPase Activating Protein; guanyl-nucleotide exchange factor activity	-1.7667891	0.00836992	Downregulated
Gm14418	Krüppel-associated box (KRAB) & zinc finger with C2H2 domain; DNA-binding transcription activity, RNA polymerase II specific	-1.70431366	0.00074572	Downregulated
Gm14403	LncRNA; regulation of transcription by RNA polymerase II	-1.47882311	0.02640515	Downregulated
Myo7a	Myosin VIIA; binds actin, cellular motor movement, involved in non-syndromic hearing loss	-1.39400656	0.0209076	Downregulated
Pcdhga11	Protocadherin gamma subfamily A, 11; establishment & function of cell-cell connections in the brain	-1.01600113	0.00404492	Downregulated
Pcdhgb6	Protocadherin Gamma Subfamily B, 6; calcium-dependent cell adhesion, establishment & function of brain cell-cell connections	-0.9970708	0.00845952	Downregulated
Msl3	Male-Specific Lethal (MSL) Complex Subunit 3; chromatin remodeling & transcription regulation	0.79889336	0.04228065	Upregulated

Frmpd4	FERM And PDZ Domain Containing 4; positive regulation of dendritic spine morphogenesis and density, excitatory synaptic transmission	0.96361123	9.61E-11	Upregulated
H2-T23	Histocompatibility 2, T region locus 23; antigen processing and presentation of endogenous peptide antigen via MHC class Ib via ER pathway, Transporter associated with antigen processing (TAP)-dependent, inner ear development, & positive regulation of T cell mediated cytotoxicity, ortholog of human HLA-E/F/G	1.25021992	0.02726812	Upregulated
H2-K1	Histocompatibility 2, K1, K region; antigen processing and presentation of endogenous peptide antigen via MHC class I via ER pathway, TAP-dependent, ortholog of human HLA-A/E/F	1.27917051	0.00493821	Upregulated
Psmb8	Proteasome 20S subunit beta 8; induced by gamma interferon and replaces catalytic subunit 3 (proteasome beta 5 subunit) in the immunoproteasome	1.28848109	0.02474348	Upregulated
Irgm2	Immunity-related GTPase family M member 2; interferon-gamma response	1.33073287	0.01612201	Upregulated
H2-T24	Histocompatibility 2, T region locus 24; antigen processing and presentation of endogenous peptide antigen via MHC class I	1.40045193	0.04985761	Upregulated

	via ER pathway, TAP-independent, antigen processing and presentation of endogenous peptide antigen via MHC class Ib, & positive regulation of T cell mediated cytotoxicity			
Slfn2	Schlafen 2; negative regulation of cell population proliferation, ortholog of human SLFN12L	1.40590447	0.04870389	Upregulated
Gbp5	Guanylate Binding Protein 5; activator of NLRP3 inflammasome assembly, innate immunity & inflammation	1.43071945	0.03446396	Upregulated
Ddx58	Asp-Glu-Ala-Asp (DEAD) box 58 or RNA sensor RIG-I; double-stranded RNA recognition & regulation of the antiviral innate immune response	1.44230841	0.01414687	Upregulated
Igtp	Interferon gamma induced GTPase; interferon-beta response, ortholog of immunity related GTPase M (IRGM)	1.46936741	0.01931592	Upregulated
ENSMUSG00000095041	AC149090.1, Phosphatidylserine Decarboxylase (PISD); catalyzes conversion of phosphatidylserine to phosphatidylethanolamine in the inner mitochondrial membrane, phospholipid metabolism	1.48106533	0.0257486	Upregulated
Trim30a	Tripartite motif-containing 30A; Negative regulation of NLRP3 inflammasome complex assembly, cytokine production, & protein autoubiquitination,	1.50326153	0.030023	Upregulated

	ortholog of human TRIM5			
Lgals3bp	Galectin 3 binding protein (GAL3BP); beta-galactoside-binding protein, immune response associated with natural killer (NK) and lymphokine-activated killer (LAK) cell cytotoxicity	1.54982782	0.00923647	Upregulated
Mid1	Midline 1; a member of the tripartite motif (TRIM) family, also known as the 'RING-B box-coiled coil' (RBCC) subgroup of RING finger proteins, formation of multiprotein structures acting as anchor points to microtubules; broadly associated with cancer & neurodegenerative disease	1.56480398	0.00022335	Upregulated
Cd52	CD52 molecule; positive regulation of cytosolic calcium	1.56710548	0.01255217	Upregulated
Rnf213	Ring finger protein 213; protein-protein interactions, ATPase activity, susceptibility gene for Moyamoya disease, a vascular disorder of intracranial arteries	1.58981172	0.00288099	Upregulated
Parp14	Poly (ADP-ribose) polymerase family member 14; anti-apoptosis, aerobic glycolysis regulation	1.59836206	0.00272254	Upregulated
Ifi47	Interferon gamma inducible protein 47; GTPase activity, defense response	1.60287742	0.02905513	Upregulated
Parp10	Poly (ADP-ribose) polymerase family member 10; gene transcription by altering	1.61424684	0.00551468	Upregulated

	chromatin organization by adding ADP-ribose to histones, transcriptional cofactor			
Uba7	Ubiquitin Like Modifier Activating Enzyme 7; promyelocytic leukemia retinoic acid receptor alpha degradation & apoptosis; conjugation of interferon-stimulated gene 15; involved in intellectual disability	1.66159332	0.00088999	Upregulated
Gm4737	Adenosylhomocysteinase like (Ahcyl); S-adenosylhomocysteine catabolic process, chronic inflammatory response to antigenic stimulus, & circadian sleep/wake cycle	1.66877525	0.02153567	Upregulated
Itgax	Integrin subunit alpha X; combines with beta 2 chain (ITGB2) to form leukocyte-specific integrin as inactivated-C3b (iC3b) receptor 4 (CR4) adherence of neutrophils and monocytes to stimulated endothelium cells	1.69233659	0.04530491	Upregulated
Ifit3	Interferon induced protein with tetratricopeptide repeats 3; negative regulation of apoptosis & cell proliferation	1.7085472	0.03228812	Upregulated
Ifi209	Interferon activated gene 209 (also known as Ifix or Pyhin1); double-stranded DNA binding activity, cellular response to interferon-beta	1.72252028	0.0262553	Upregulated
*Cybb**	Cytochrome b-245 beta chain; primary component of the microbicidal oxidase system (NADPH	1.7757256	0.01197482	Upregulated

	oxidase complex) of phagocytes			
Tap1	Transporter 1, ATP binding cassette subfamily B member, member of multidrug resistance (MDR)/TAP subfamily	1.78777725	0.00037158	Upregulated
Clec7a	C-type lectin domain containing 7A; member of the C-type lectin/C-type lectin-like domain (CTL/CTLD) superfamily, innate immunity	1.82199948	0.0261512	Upregulated
*Mx2**	MX dynamin like GTPase 2; involved in interferon-alpha/beta, anti-proliferation of T-cells	1.91325641	0.02860071	Upregulated
Gbp3	Guanylate Binding Protein 3; Interferon (IFN)-inducible GTPase, innate immunity	1.94626246	0.0005096	Upregulated
Gm22513	A small nuclear RNA (snRNA); unknown	1.97684762	0.01787355	Upregulated
Gbp6	Guanylate binding protein family member 6; induced by interferon	2.01056874	0.01786311	Upregulated
Adgrg5	Adhesion G protein-coupled receptor G5; immunity & central nervous system function	2.05456791	0.02157012	Upregulated
Apol11b	Apolipoprotein L 11b; enables chloride channel and lipid binding activity	2.07696079	0.02521993	Upregulated
Phf11b	Plant homeodomain (PHD) Finger Protein 11; positive regulation of Th1-type cytokine gene expression	2.07713894	0.00499437	Upregulated
H2-Q4	Histocompatibility 2, Q region locus 4; 14-3-3 protein, transporter associated with antigen processing (TAP) & signaling receptor binding	2.11312036	0.00022449	Upregulated

	activity, human orthologs include HLA-E, HLA-F, & HLA-G			
Cd300lf	CD300 molecule like family member f; cell surface glycoprotein with single IgV-like extracellular domain, involved in immunity	2.14819305	0.02173225	Upregulated
Nlrc5	NOD-like receptor (NLR) family caspase recruitment domain (CARD) domain containing 5; cytokine response & antiviral immunity	2.1976673	0.00031727	Upregulated
ENSMUSG00002076091	Gm54573 as a miRNA; unknown	2.21241082	0.0030355	Upregulated
*Oas1b**	2'-5'-Oligoadenylate Synthetase 1; innate cellular antiviral response, cell growth & apoptosis; genetic variants associated with AD & in some cases linked to severe SARS-CoV-2 infection	2.22371584	0.0042633	Upregulated
Trim5	Tripartite motif containing 5; E3 ubiquitin ligase	2.28827272	0.03261839	Upregulated
Ddx60	DExD/H-box helicase 60; Asp-Glu-Ala-Asp (DEAD) protein, RNA helicase, RIG-I-like receptor-mediated signaling	2.30321276	0.00088103	Upregulated
Gm24727	Predicted gene, 24727; unknown	2.30747535	0.00222333	Upregulated
Gpr65	G protein-coupled receptor 65 (also known as G protein-coupled receptor T-cell death-associated gene 8, TDAG8); actin cytoskeleton reorganization, positive regulation of stress fiber	2.31562363	0.02689341	Upregulated

	assembly, acidification sensor			
Gm12250	Interferon-gamma-inducible GTPase 10 (IRGB10); immunity	2.32532066	0.00250391	Upregulated
Milr1	Mast cell immunoglobulin like receptor 1; cell-cell adhesion via plasma-membrane adhesion molecules, mast cell degranulation and deactivation	2.32815134	0.03557371	Upregulated
Gm38431	LncRNA; represents a read-through transcript composed of ribonucleoprotein, PTB binding 1 (Raver1) and ferredoxin 2 (Fdx1l) sequence	2.42861836	0.02851569	Upregulated
Gvin3	GTPase, very large interferon inducible, family member 3; immunity	2.51884409	0.00872253	Upregulated
*Rsad2**	Radical S-Adenosyl Methionine Domain Containing 2; interferon-inducible antiviral protein of the S-adenosyl-L-methionine (SAM) superfamily of enzymes, innate immunity, antiproliferative activity in T-cells	2.56688006	2.91E-05	Upregulated
Ifi213	Interferon activated gene 213; double-stranded DNA binding activity, cellular response to interferon-beta	2.62657514	0.03117219	Upregulated
I730030J21Rik	RIKEN cDNA I730030J21 gene; lncRNA	2.64668116	0.04113708	Upregulated
Ms4a4b	Membrane-spanning 4-domain family, subfamily A (also known as Chandra); CD20 homologue in T cells	2.76912003	0.04165959	Upregulated

Bst2	Bone Marrow Stromal Cell Antigen 2; growth & development of B-cells, maybe be enriched in reactive astrocytes and associated with glioma & multiple sclerosis	2.79058731	6.60E-05	Upregulated
Gm12185	Predicted gene 12185; involved in cellular response to interferon-beta and defense response	2.84825539	0.04291158	Upregulated
H2-Q6	Histocompatibility 2, Q region locus 6; 14-3-3 protein, transporter associated with antigen processing (TAP) & signaling receptor binding activity, human orthologs include HLA-E, HLA-F, & HLA-G	2.85255027	0.0020352	Upregulated
Ifi208	Interferon activated gene 208; double-stranded DNA binding activity, cellular response to interferon-beta	2.86773136	0.04423117	Upregulated
Troap	Trophinin associated protein (also known as TASTIN); cell adhesion	2.96510479	0.03564332	Upregulated
Igkv3-2	Immunoglobulin kappa variable 3-2; immunity	3.03388016	0.04105357	Upregulated
*Oasl1**	2'-5' oligoadenylate synthetase-like 1; double-stranded RNA binding activity, acts upstream of or within interleukin-27-mediated signaling pathway, orthologous to human OASL, associated with AD and/or SARS-CoV-2 infection	3.04400545	0.00757733	Upregulated
Fcgr4	Fc receptor, IgG, low affinity IV; IgE and IgG receptor activity, neutrophil activation & positive regulation of bone resorption, ortholog of human FCGR3A/B	3.0515848	0.04168559	Upregulated

mt-Tv	Mitochondrially encoded tRNA valine; mitochondrial protein building	3.1032799	0.03592835	Upregulated
G430095P16Rik	RIKEN cDNA G430095P16 gene; lncRNA	3.14460404	0.0426358	Upregulated
Ifi204	Interferon activated gene 204; enables double-stranded DNA binding activity & transcription coregulator activity, involved in cellular response to interferon-α; common marker of SARS-CoV-2 infection and AD	3.18440953	0.00192825	Upregulated
Igkv12-46	Immunoglobulin kappa variable 12-46; immunity	3.24873365	0.01174048	Upregulated
Gm45620	Predicted gene 45620; lncRNA	3.32916144	0.01891422	Upregulated
Isg15	Interferon-stimulated protein, 15KDa, Ubiquitin Like Modifier; chemotactic activity towards neutrophils, direction of ligated target proteins to intermediate filaments, cell-to-cell signaling, & antiviral activity, upregulated with exercise during AD	3.33303052	2.91E-05	Upregulated
Gm5841	Ubiquitin specific peptidase 1 pseudogene	3.35501284	0.04049448	Upregulated
Zbp1	Z-DNA Binding Protein 1; innate immune response & type-I interferon production	3.44586846	0.00348033	Upregulated
Oas3*	2'-5'-oligoadenylate synthetase 3; induced by interferons and catalyzes the 2', 5' oligomers of adenosine in order to bind and activate RNase L, inhibition of cellular protein synthesis and viral infection resistance	3.54299512	0.00020383	Upregulated

Gm19585	Predicted gene, 19585; lncRNA	3.55926343	0.00466479	Upregulated
Ifi27l2a	Interferon, alpha-inducible protein 27 like 2A; aging, response to virus, pro-apoptotic, located in mitochondrial membrane, human orthologs include interferon alpha inducible protein 27 like 2 (IFI27L2)	3.5718259	1.71E-05	Upregulated
Oas1g	2'-5' oligoadenylate synthetase 1G; induced by interferon, immunity	3.5780495	0.04644078	Upregulated
Iigp1	Interferon inducible GTPase 1; involved in defense response to Gram-negative bacterium, regulation of autophagy	3.58815314	1.56E-05	Upregulated
Gm5518	Pseudogene; unknown	3.59803493	0.00036327	Upregulated
Ifi44	Interferon induced protein 44; immunity	3.65032413	0.00219821	Upregulated
Ifit1	Interferon Induced Protein With Tetratricopeptide Repeats 1; inhibits viral replication translational initiation, paralog of IFIT1B	3.70000039	1.02E-07	Upregulated
Irf7	Interferon Regulatory Factor 7; transcriptional activation of virus-inducible cellular genes, such as interferon beta chain genes, shared among AD& SARS-CoV-2	3.71093672	4.90E-05	Upregulated
Slfn4	Schlafen 4; activation by Toll-like receptor agonists & repressed during macrophage colony-stimulating factor-mediated differentiation	3.74753927	0.00512636	Upregulated
*Ccl4**	C-C motif chemokine ligand 4; mitogen-	3.76576208	0.02248828	Upregulated

	inducible monokine, chemokinetic & inflammatory functions			
Gm35101	Predicted gene, 35101; lncRNA	3.76923408	0.04090537	Upregulated
*Oas1a**	2'-5' oligoadenylate synthetase 1A; innate cellular antiviral response, cell growth & apoptosis; genetic variants associated with AD & in some cases linked to severe SARS-CoV-2 infection	3.78022214	0.00016011	Upregulated
Mx1	Myxovirus Dynamin Like GTPase 1; cellular antiviral response, associated with AD and SARS-CoV-2 infection	3.80330404	8.63E-05	Upregulated
Cst7	Cystatin F; glycosylated cysteine protease inhibitor; immune regulation	3.82371554	0.00326436	Upregulated
Ms4a4c	Membrane-spanning 4-domains, subfamily A, member 4C; regulates lipid metabolism & immunity, ortholog of human MS4A4A	3.84147978	0.00108668	Upregulated
Usp18	Ubiquitin Specific Peptidase 18; cleave ubiquitin from ubiquitinated protein substrates, upregulated with exercise during AD	3.87593652	2.22E-07	Upregulated
H2-Q7	Histocompatibility 2, Q region locus 7; 14-3-3 protein, transporter associated with antigen processing (TAP) & signaling receptor binding activity, human orthologs include HLA-E, HLA-F, & HLA-G	3.91480233	4.71E-07	Upregulated
Oasl2	2'-5'-Oligoadenylate Synthetase Like 2;	4.0552608	0.00021807	Upregulated

	interleukin-27-mediated signaling pathway, negative regulation of viral genome replication, and positive regulation of retinoic acid-inducible gene I (RIG-I) signaling pathway			
Ifi211	Interferon activated gene 211; DNA damage response, regulation of cell survival	4.20183458	0.01306063	Upregulated
*Cxcl10**	C-X-C motif chemokine ligand 10; binds to CXCR3 & stimulates monocytes, natural killer and T-cell migration, and modulation of adhesion molecule expression, key regulator of the 'cytokine storm' immune response to SARS-CoV-2 infection	4.28192363	0.01015046	Upregulated
Dnase1l3	Deoxyribonuclease 1L3; mediates breakdown of DNA during apoptosis	4.30574918	0.00449648	Upregulated
Gm4951	Interferon inducible GTPase 1C (Ligp1c); response to interferon-beta, immunity	4.40336851	0.01127939	Upregulated
*Gm16439**	BCL2/adenovirus E1B interacting protein 3 (BNIP3), pseudogene; pro-apoptosis	4.42038727	0.00048323	Upregulated
*Oas2**	2'-5'-Oligoadenylate Synthetase 2; innate cellular antiviral response, cell growth & apoptosis; associated with AD & SARS-CoV-2 infection	4.47620762	0.00032353	Upregulated
Ifi206	Interferon activated gene 206; regulation of cysteine-type endopeptidase activity, regulation of gene expression, & and regulation of innate	4.6156588	0.00027311	Upregulated

	immune response, ortholog of human IFI16; may interact with the apolipoprotein E2 allele			
Cxcl13	C-X-C Motif Chemokine Ligand 13 or "Angie"; B lymphocyte chemoattractant, B lymphocyte migration, a paralog of CXCL3	5.00280435	0.00094533	Upregulated
Gm7324	Pseudogene of RNA Binding Motif Protein X-Linked (RBMX); positive regulation of mRNA splicing (e.g., low density lipoprotein receptor, LDLR) via spliceosome, involved in intellectual development disorder	6.03500758	2.25E-09	Upregulated
*Ccl5**	C-C motif chemokine ligand 5; chemoattractant for blood monocytes, memory T helper cells and eosinophils, release of histamine from basophils, activates eosinophils, ligand of CCR5	6.04158219	9.56E-07	Upregulated
Dntt	DNA nucleotidylexotransferase; member of the DNA polymerase type-X family, generates antigen receptor diversity by synthesizing non-germ line elements (N-regions) at the junctions of rearranged Ig heavy chain and T cell receptor gene segments	14.6148913	4.00E-09	Upregulated

Table of Contents Top of Current Table

Supplemental Table 32. List of all overlap of significant (P < 0.05) differentially expressed genes in both whole blood and brain of 3xTg-AD CBD (week 8) versus wild-type B6129 CBD (week 8) animals. Italicized genes have a recognized association with Alzheimer's disease (AD) pathology.

**Common Genes Downregulated**	**Common Genes Upregulated**	**Common but Opposite: Downregulated in 3xTg-AD CBD Blood & Upregulated in 3xTg-AD CBD versus B6129 CBD**
Capza1-ps1; Capping Protein (Actin Filament) Muscle Z-Line, Alpha 1 Pseudogene; growth of actin filaments	Apol11b; Apolipoprotein L 11b; chloride channel & lipid binding	Dntt; DNA nucleotidylexotransferase; member of the DNA polymerase type-X family; antigen receptor diversity
Ccl27a; C-C motif chemokine ligand 27A; T cell chemotaxis, cytoskeleton reorganization	*Cxcl13*; C-X-C Motif Chemokine Ligand 13 or "Angie"; lymphocyte migration	
Eif3j2; Eukaryotic translation initiation factor 3, subunit J2; protein synthesis	Fcgr4; Fc receptor, IgG, low affinity IV; IgE and IgG receptor activity, neutrophil activation	
Eno1b; Enolase 1b, retrotransposed evolving pseudogene of enolase 1; embryonic development	Frmpd4; FERM And PDZ Domain Containing 4; dendritic spine morphogenesis and density, excitatory synaptic transmission	
Gm10548; Pseudogene of ribosomal protein L29; protein synthesis	Gm24727; Predicted gene, 24727	
Gm11868; Pseudogene of cytochrome b5 reductase 4; endoplasmic reticulum stress response, protection against reactive oxygen species	Gm4737; Adenosylhomocysteinase like (Ahcyl); chronic inflammatory response, circadian sleep/wake cycle	
Gm12312; Pseudogene of FGFR1 oncogene partner 2; wound healing	Gm7324; Pseudogene of RNA Binding Motif Protein X-Linked (RBMX); positive regulation of mRNA splicing (e.g.,	

	low density lipoprotein receptor, LDLR)
Gm14287;<br>Pseudogene	Ifi27l2a;<br>Interferon, alpha-inducible protein 27 like 2A; aging, response to virus, pro-apoptotic
Gm15459;<br>Pseudogene of heat shock protein 8; protein folding	*Isg15*;<br>Interferon-stimulated protein, 15KDa, Ubiquitin Like Modifier; chemotactic activity towards neutrophils, antiviral activity
Gm26782;<br>lncRNA	Milr1;<br>Mast cell immunoglobulin like receptor 1; cell-cell adhesion mast cell degranulation
Gm26881;<br>lncRNA	Oas1g;<br>2'-5' oligoadenylate synthetase 1G; induced by interferon, immunity
Gm35082;<br>lncRNA	Slfn4;<br>Schlafen 4; activation by Toll-like receptor agonists
Gm43305;<br>lncRNA, aldosterone-induced	
Gm49980;<br>lncRNA;<br>within proximity of Pik3c3 (phosphatidylinositol-3-kinase; upstream of/within autophagy & protein lipidation)	
Gm6916;<br>pseudogene; cytoplasmic translation	
H2af-ps2;<br>H2A histone family, pseudogene 2;<br>nucleosome structure	
H4c17;<br>H4 clustered histone 17;	

nucleosome structure
Rec8;<br>REC8 meiotic recombination protein; chromosome synapsis
Rps13-ps1;<br>Ribosomal protein S13 pseudogene; protein synthesis
Rps3a3;<br>Ribosomal protein S3A; protein synthesis
Ubb-ps;<br>Ubiquitin B pseudogene; protein degradation
Zfp125;<br>Zinc finger protein 125; transcriptional repression, lipoprotein structure, lipid binding & transport

Table of Contents Top of Current Table

Supplemental Table 33. List of all significant (P < 0.05) differentially expressed positive mode metabolites in whole brain of *3xTg-AD* and wild-type B6129 mice (6.5 mo old) following 8 weeks of cannabidiol (CBD) or vehicle treatment; *3xTg-AD* vehicle vs. B6129 vehicle (8 & 65 metabolites upregulated & downregulated respectively in comparison), *3xTg-AD* CBD vs. *3xTg-AD* vehicle (0 & 0), B6129 CBD vs. B6129 vehicle (3 & 9), & *3xTg-AD* CBD vs. B6129 CBD (5 & 9).

**Positive Mode Metabolites**	**Comparison Group**	**log2FoldChange**
1-(-docosahexaenoyl)-sn-glycero-3-phosphocholine	3xTg-AD VEH vs. WT VEH	-0.96358
1-Linoleoylglycerophosphocholine	3xTg-AD CBD vs. WT CBD	-0.63927
	3xTg-AD VEH vs. WT VEH	-0.55047
1,2-dihydroxyheptadec-16-yn-4-yl acetate	3xTg-AD VEH vs. WT VEH	-0.97121
10-Nitrooleate	3xTg-AD VEH vs. WT VEH	-0.71744
11-Aminoundecanoic acid	3xTg-AD VEH vs. WT VEH	0.932088
11-oxo-9-prosta tetraenoic acid	3xTg-AD VEH vs. WT VEH	-0.63517
13,16,19-Docosatrienoicacid	3xTg-AD CBD vs. WT CBD	1.270641
14-HDHA	3xTg-AD VEH vs. WT VEH	-0.8233
14'-apo-Carotenal	3xTg-AD VEH vs. WT VEH	-0.56492
16-HETE	3xTg-AD CBD vs. WT CBD	-0.72876
	3xTg-AD VEH vs. WT VEH	-0.54283
18-HETE	3xTg-AD VEH vs. WT VEH	-0.58004
2-(Acetamidomethylene)succinate	3xTg-AD VEH vs. WT VEH	-1.07941
2-(Diethoxymethyl)furan	3xTg-AD VEH vs. WT VEH	-0.94986
	WT CBD vs. WT VEH	-0.39979
2-arachidonyl-sn-glycero-3-phosphoethanolamine	3xTg-AD CBD vs. WT CBD	-0.72962
2-Butylfuran	3xTg-AD VEH vs. WT VEH	-1.00462
2-Methylserine	WT CBD vs. WT VEH	0.177988
2,4-Dimethylbenzaldehyde	3xTg-AD VEH vs. WT VEH	-1.01047
2,4-Pentadiynylbenzene	3xTg-AD VEH vs. WT VEH	-0.97031
	WT CBD vs. WT VEH	-0.55331
2,6-dimethylenaphthalene	3xTg-AD VEH vs. WT VEH	-1.07295
22-Oxodocosanoic acid	3xTg-AD VEH vs. WT VEH	1.030382
3-Hexenyl phenylacetate	3xTg-AD VEH vs. WT VEH	-0.48528
3-Methyl-1-phenyl-2-butene	3xTg-AD VEH vs. WT VEH	-1.03469
3-Methylcrotonylglycine	3xTg-AD VEH vs. WT VEH	-1.05765
3-Methylethcathinone	3xTg-AD VEH vs. WT VEH	-1.09274

4-(Trimethylammonio)-3-(undecanoyloxy)butanoate	3xTg-AD VEH vs. WT VEH	-0.59216
4-Methyl-2-phenyl-2-pentenal	3xTg-AD VEH vs. WT VEH	-1.13783
4-Methyl-2-propyltetrahydro-2H-pyran-4-yl acetate	WT CBD vs. WT VEH	-0.81858
4-(stearoyl amino)butanoic acid	3xTg-AD VEH vs. WT VEH	0.946177
	WT CBD vs. WT VEH	0.204144
5-Hydroxytryptophan	3xTg-AD VEH vs. WT VEH	-0.84414
9-Nitrooleate	3xTg-AD CBD vs. WT CBD	-1.43567
Abietic acid	3xTg-AD CBD vs. WT CBD	-0.55607
Aceclidine	3xTg-AD VEH vs. WT VEH	-1.00936
Acetyl-methylcholine	3xTg-AD VEH vs. WT VEH	0.644296
Androstenedione	3xTg-AD VEH vs. WT VEH	-0.74247
Anisole	3xTg-AD VEH vs. WT VEH	-0.94618
Arachidonic acid methyl ester	3xTg-AD CBD vs. WT CBD	-2.32178
	3xTg-AD VEH vs. WT VEH	-2.26856
Aspartame	3xTg-AD VEH vs. WT VEH	-0.50942
Benzene	3xTg-AD VEH vs. WT VEH	-0.96255
Benzyl isobutyl ketone	3xTg-AD VEH vs. WT VEH	-1.07695
Butopyronoxyl	WT CBD vs. WT VEH	-0.33013
Cedrene	WT CBD vs. WT VEH	0.490204
Cetrimonium	3xTg-AD VEH vs. WT VEH	1.671084
Cetyl Benzoate	3xTg-AD CBD vs. WT CBD	-1.72981
	3xTg-AD VEH vs. WT VEH	-1.9246
Choline	3xTg-AD VEH vs. WT VEH	-0.20403
Coniferyl alcohol	3xTg-AD VEH vs. WT VEH	-0.55919
Costunolide	3xTg-AD VEH vs. WT VEH	-0.51362
Cyclo(leucylprolyl)	3xTg-AD VEH vs. WT VEH	-0.90485
Cyclohex-2-enone	3xTg-AD VEH vs. WT VEH	-0.72207
dehydroretinaldehyde	3xTg-AD VEH vs. WT VEH	-0.67147
Docosahexaenoic acid	3xTg-AD VEH vs. WT VEH	-0.56372
Epoxy-eicosatetraenoic acid	3xTg-AD VEH vs. WT VEH	-0.73267
Esmolol	3xTg-AD VEH vs. WT VEH	1.143567
Fenestrel	3xTg-AD VEH vs. WT VEH	-0.54627
Fingolimod	3xTg-AD VEH vs. WT VEH	-0.99222

Glutamate	WT CBD vs. WT VEH	-0.27826
Glycero-3-Phosphoethanolamine	3xTg-AD CBD vs. WT CBD	1.314297
Hexylresorcinol	3xTg-AD VEH vs. WT VEH	-1.0892
Ibufenac	3xTg-AD VEH vs. WT VEH	-1.13585
Isocyanocyclohexane	3xTg-AD VEH vs. WT VEH	-0.97212
Jasmone	3xTg-AD VEH vs. WT VEH	-1.09584
Jasmonic acid	3xTg-AD VEH vs. WT VEH	-0.86928
Lariciresinol	3xTg-AD VEH vs. WT VEH	-0.82904
Lithocholic Acid	3xTg-AD VEH vs. WT VEH	0.828331
lysophosphatidylethanolamine (22:6/0:0)	3xTg-AD CBD vs. WT CBD	-0.70215
Megestrol	3xTg-AD VEH vs. WT VEH	-0.97663
Methyl 15-cyanopentadecanoate	3xTg-AD VEH vs. WT VEH	-1.01709
Metolachlor morpholinone	3xTg-AD VEH vs. WT VEH	-1.13208
N-(9-oxodecyl) acetamide	WT CBD vs. WT VEH	-0.31157
N-dodecanoylsphinganine	3xTg-AD CBD vs. WT CBD	0.706553
	3xTg-AD VEH vs. WT VEH	0.798726
N-Ethyl-2,6-nonadienamide	3xTg-AD VEH vs. WT VEH	-1.13885
N-hexadecyl-ethanolamine	3xTg-AD CBD vs. WT CBD	0.506007
N-Methylconiine	3xTg-AD VEH vs. WT VEH	-1.01721
N,N-Bis(2-hydroxyethyl)dodecanamide	3xTg-AD CBD vs. WT CBD	2.252767
Naphthalene	3xTg-AD VEH vs. WT VEH	-0.88012
Oglufanide	3xTg-AD VEH vs. WT VEH	-0.48643
p-Xylene	3xTg-AD VEH vs. WT VEH	-0.98759
Palmitoyl ethanolamide	WT CBD vs. WT VEH	-0.36967
Pantothenol	3xTg-AD VEH vs. WT VEH	-0.72523
Promegestone	3xTg-AD CBD vs. WT CBD	-0.99396
	3xTg-AD VEH vs. WT VEH	-0.74722
Pulegone	WT CBD vs. WT VEH	-0.55655
Rivastigmine	3xTg-AD VEH vs. WT VEH	-1.58467
Santene	3xTg-AD VEH vs. WT VEH	-0.97717
	WT CBD vs. WT VEH	-0.38917
Sedanolide	3xTg-AD VEH vs. WT VEH	-1.08557
Timonacic	3xTg-AD VEH vs. WT VEH	-0.4719
Toluene	3xTg-AD VEH vs. WT VEH	-0.96976
Triethylene glycol monomethyl ether	3xTg-AD VEH vs. WT VEH	-0.78901

Trimethadione	3xTg-AD VEH vs. WT VEH	-0.88333
Valethamate	3xTg-AD VEH vs. WT VEH	-0.714
Valpromide	3xTg-AD VEH vs. WT VEH	-0.81195

Supplemental Table 34. List of all significant (P < 0.05) differentially expressed negative mode metabolites in whole brain of *3xTg-AD* and wild-type B6129 mice (6.5 mo old) following 8 weeks of cannabidiol (CBD) or vehicle treatment; *3xTg-AD* vehicle vs. B6129 vehicle (19 & 20 metabolites upregulated & downregulated respectively in comparison), *3xTg-AD* CBD vs. *3xTg-AD* vehicle (0 & 3), B6129 CBD vs. B6129 vehicle (0 & 2), & *3xTg-AD* CBD vs. B6129 CBD (26 & 9).

**Negative Mode Metabolites**	**Comparison Group**	**log2FoldChange**
1-phenylpropane-1,2-dione	3xTg-AD CBD vs. WT CBD	0.39595
11-Eicosenoic acid	3xTg-AD CBD vs. WT CBD	0.420581
	3xTg-AD VEH vs. WT VEH	0.50032
11,14,17-Eicosatrienoic acid	3xTg-AD CBD vs. WT CBD	0.286312
	3xTg-AD VEH vs. WT VEH	0.377002
11(12)-EET	3xTg-AD CBD vs. WT CBD	-0.48464
13,16,19-Docosatrienoic acid	3xTg-AD CBD vs. WT CBD	0.496247
	3xTg-AD VEH vs. WT VEH	0.422802
14-HDoHE	3xTg-AD VEH vs. WT VEH	-1.05882
19-Hydroxy prostaglandin F2	3xTg-AD VEH vs. WT VEH	-0.33531
2-Aminomuconate	3xTg-AD CBD vs. WT CBD	-0.98902
	3xTg-AD VEH vs. WT VEH	-0.91333
2-Hydroxyethanesulfonate	3xTg-AD VEH vs. WT VEH	-0.84101
2-Hydroxyglutarate	3xTg-AD CBD vs. WT CBD	0.752532
3-Hydroxybutanoate	3xTg-AD VEH vs. WT VEH	-0.46454
3-Methylindole	3xTg-AD CBD vs. WT CBD	0.44524
3b-Hydroxy-5-cholenoic acid	3xTg-AD CBD vs. WT CBD	0.459593
4-Hydroxyphenyllactic acid	3xTg-AD VEH vs. WT VEH	-0.5215
4-Nitrophenyl-3-ketovalidamine	3xTg-AD VEH vs. WT VEH	0.499973
5-deoxy-J2-IsoP	3xTg-AD VEH vs. WT VEH	-0.47338
5,5-Dimethylhydantoin	3xTg-AD VEH vs. WT VEH	0.283515
5'-S-Methyl-5'-thioinosine	3xTg-AD VEH vs. WT VEH	-0.42892
Acetylphosphate	3xTg-AD VEH vs. WT VEH	-0.54261
Anatabine	3xTg-AD CBD vs. WT CBD	0.558253
Androstane	WT CBD vs. WT VEH	-0.15976
Bardoxolone methyl	3xTg-AD CBD vs. WT CBD	-0.37299
Carnitine	3xTg-AD CBD vs. WT CBD	0.357312
cis-5-Tetradecenoylcarnitine	3xTg-AD CBD vs. WT CBD	0.460989
	3xTg-AD VEH vs. WT VEH	0.509437

Eptapirone	3xTg-AD VEH vs. WT VEH	1.02533
Ethyl myristate	3xTg-AD VEH vs. WT VEH	0.148654
Fructose 6-phosphate	3xTg-AD CBD vs. WT CBD	0.242568
	3xTg-AD VEH vs. WT VEH	0.295924
Geosmin	3xTg-AD CBD vs. WT CBD	-1.25056
	3xTg-AD VEH vs. WT VEH	-0.7286
Glutaconate	3xTg-AD CBD vs. WT CBD	0.36941
Glutathione (reduced)	3xTg-AD VEH vs. WT VEH	-0.36672
Glutathione oxidized	3xTg-AD VEH vs. WT VEH	-0.46259
Glycerophosphoglycerol	3xTg-AD CBD vs. WT CBD	0.484098
Guanosine	3xTg-AD CBD vs. WT CBD	-1.16266
	3xTg-AD VEH vs. WT VEH	-1.12767
Icosadienoic acid	3xTg-AD CBD vs. WT CBD	0.362731
Indole;1-Benzazole	3xTg-AD CBD vs. WT CBD	0.286483
Inosine	3xTg-AD VEH vs. WT VEH	-0.26795
Leucine	3xTg-AD CBD vs. WT CBD	0.414598
Lysergic acid	3xTg-AD CBD vs. WT CBD	-0.88844
	3xTg-AD VEH vs. WT VEH	-0.54285
Lysophosphatidylethanolamine (22:6/0:0)	3xTg-AD CBD vs. WT CBD	-0.27304
	3xTg-AD VEH vs. WT VEH	-0.3894
Maleamate	3xTg-AD VEH vs. WT VEH	-0.34252
Malic acid	WT CBD vs. WT VEH	-0.18302
Margaric acid	3xTg-AD CBD vs. WT CBD	0.444417
	3xTg-AD VEH vs. WT VEH	0.277499
Metaphosphoric acid (HPO3)	3xTg-AD CBD vs. 3xTg-AD VEH	-0.05971
	3xTg-AD VEH vs. WT VEH	0.0683
Myristic acid	3xTg-AD VEH vs. WT VEH	0.244028
N-Acetyl-DL-methionine	3xTg-AD VEH vs. WT VEH	-0.4678
N-Acetylaspartic acid	3xTg-AD VEH vs. WT VEH	-1.53188
N-Arachidonoyl taurine	3xTg-AD CBD vs. WT CBD	-0.38323
N(2)-Acetyl-L-aminoadipate	3xTg-AD VEH vs. WT VEH	-0.65913
Naphthalen-2-amine	3xTg-AD CBD vs. WT CBD	0.455446
Oleic acid	3xTg-AD CBD vs. WT CBD	0.229334
	3xTg-AD VEH vs. WT VEH	0.203385

Orthophosphate	3xTg-AD CBD vs. 3xTg-AD VEH	-0.06085
	3xTg-AD VEH vs. WT VEH	0.068918
Pantetheine	3xTg-AD CBD vs. WT CBD	0.348084
	3xTg-AD VEH vs. WT VEH	0.445071
Phenylalanine	3xTg-AD CBD vs. WT CBD	0.394814
Phytomonic acid	3xTg-AD CBD vs. WT CBD	0.542073
	3xTg-AD VEH vs. WT VEH	0.513407
Pyrophosphoric acid	3xTg-AD CBD vs. 3xTg-AD VEH	-0.12775
	3xTg-AD CBD vs. WT CBD	-0.17522
sn-glycero-3-Phosphoethanolamine	3xTg-AD CBD vs. WT CBD	1.911593
	3xTg-AD VEH vs. WT VEH	1.145419
Stearic acid	3xTg-AD CBD vs. WT CBD	0.213134
Tetraacetylethylenediamine	3xTg-AD VEH vs. WT VEH	0.956204
Tryptophan	3xTg-AD CBD vs. WT CBD	0.430918
Xanthosine	3xTg-AD CBD vs. WT CBD	0.538977
	3xTg-AD VEH vs. WT VEH	0.557439

Table of Contents Top of Current Table
